# Supplementary material for: Direct deoxygenative borylation of carboxylic acids
Source: Nat Commun. 2021 Aug 17;12:4970. doi: 10.1038/s41467-021-25229-8 (PMC8370987; doi:10.1038/s41467-021-25229-8)
Supplement: Supplementary file 1 — Supplementary Information [file 41467_2021_25229_MOESM1_ESM.pdf]

Supplementary information for

Direct Deoxygenative Borylation of Carboxylic Acids

Jianbin Li[1,2,3], Chia-Yu Huang[1,2,3], Mohamad Ataya[1,3], Rustam Z. Khaliullin[1,*], Chao-Jun Li[1,2,*]

[1]Department of Chemistry, McGill University, 801 Sherbrooke Street W, Montreal, Quebec H3A 0B8, Canada.

[2]FRQNT Centre for Green Chemistry and Catalysis.

[3]These authors contributed equally

1. Supplementary Notes

1.1. Solvents and chemicals

Liquid chemicals (e.g., Et_3N) used in this work are pre-treated over 4 Å molecular sieves (beads, 8-12 mesh), and water is distilled. The 4 Å molecular sieves were purchased from Sigma-Aldrich chemical company and were freshly activated in the oven for 12 hours at 380 ℃ before use. Most of the solvents and reagents used in this work were purchased from Sigma-Aldrich, Alfa Aesar Chemical and Combi-Blocks companies and used without further purification unless otherwise specified. For the rest, they were well documented and readily accessed by laboratory synthesis according to the literature reports.

1.2. Spectroscopies (NMR, GC-MS, HRMS)

Nuclear magnetic resonance (NMR) spectra, including ^{1}H, ^{13}C and ^{19}F (CCl_3F set at 0 ppm) and ^{11}B NMR were recorded on Bruker 400 MHz or 500 MHz spectrometers, which used the deuterium lock signal to reference the spectra. The solvent residual peaks, e.g., of chloroform ($CDCl_3$: δ 7.28 ppm for ^{1}H NMR and δ 77.0 ppm for ^{13}C NMR), were used as references. Data were reported as follows: multiplicity (s = singlet, d = doublet, t = triplet, q = quartet, quint = quintet, m = multiplet, dd = doublet of doublet, etc), coupling constant (*J*/Hz) and integration. All NMR spectra were recorded at room temperature. For ^{11}B NMR, the broad singlet peak at around 0 ppm arises from the borosilicate glass NMR tube. EI-MS was obtained from the Agilent gas chromatography-mass spectroscopy (GC-MS) system with helium (He) as the carrier gas. H_2 detection was performed on an Agilent gas chromatography-thermal conductivity detector (GC-TCD) system with argon (Ar) as the carrier gas. High-resolution mass spectrometry (HRMS) was conducted by using atmospheric pressure chemical ionization (APCI) or electro-spraying ionization (ESI) and was performed by McGill University on a Thermo-Scientific Exactive Orbitrap. Protonated/deprotonated molecular ions $(M\pm H)^+$ or sodium adducts $(M+Na)^+$ were used for empirical formula confirmation.

1.3. Compound purification

Short packed column chromatography was performed with E. Merck silica gel 60 (230–400 mesh) or SORBENT silica gel 30-60 μm. Flash column chromatography was performed with the Isolera™ Prime advanced automatic flash purification system. Analytical thin-layer chromatography (TLC) was performed using Merck silica gel 60 F254 pre-coated plates (0.25 mm). ***After purification, all the boronates were stored at -20 ℃ to prevent decomposition.***

1.4. Terminology and compound name abbreviation

PTFE, polytetrafluoroethylene;
ND, not detected;
B_2cat_2, bis(catecholato)diboron;
B_2pin_2, bis(pinacolato)diboron
Et_3N, triethylamine;
EtOAc, ethyl acetate;
Et_2O, diethyl ether;
Na_2SO_4, sodium sulfate;
DMA, *N*,*N*-dimethylacetamide;
DMF, *N*,*N*-dimethylformamide;
NMP, *N*-methylpyrrolidone;
DMPU, *N*,*N*-dimethylpropyleneurea;
DMEU, *N*,*N*-dimethylethyleneurea;
HMPA, hexamethylphosphoramide;
DMSO, dimethylsulfoxide;
THF, tetrahydrofuran;
DMC, dimethyl carbonate;
DMAP, 4-(dimethylamino)pyridine;
H-Bcat, catecholborane;
$NaB(cat)_2$, sodium bis(catecholato)borate;

2. Supplementary Methods

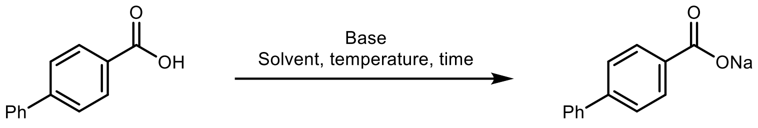

1a-CO_2H → **1a**

Method A: $NaOH_s$, toluene, 75 °C, 1 h; *then*, air drying, 12 h;
Method B: $NaOH_s$, toluene, 75 °C, 1 h; *then*, dried at 70 °C, 12 h;
Method C: $NaOH_{aq}$, toluene, 75 °C, 1 h; *then*, air drying, 12 h;
Method D: $NaO\textit{t}\text{-}Bu_{EtOH}$, EtOH, rt, 1 h; *then*, dried at 70 °C, 12 h;

The preparation of 4-biphenylcarboxylic acid salt is representative of all the metal carboxylates used in this work. Simple carboxylate, e.g., sodium benzoate ($PhCO_2Na$) and potassium benzoate ($PhCO_2K$), were commercially available and used directly without further treatment.

Method A: To a flame-dried round bottom flask (50 mL) equipped with a Teflon-coated magnetic stirring bar was added 4-biphenylcarboxylic acid (**1a**, 0.495 g, 2.5 mmol, 1.0 equiv), which was suspended on 6.25 mL toluene (0.40 M). To the suspension was added finely ground NaOH powder (0.0999 g, 2.5 mmol, 1.0 equiv), and the mixture was stirred at 75 ℃ for 1.0 hour (colour change from pale yellow to white was observed). After the reaction, the white and powdery precipitate was filtered and dried by airflow overnight, which was used directly for other reactions without further purification.

Method B: To a flame-dried round bottom flask (50 mL) equipped with a Teflon-coated magnetic stirring bar was added 4-biphenylcarboxylic acid (**1a**, 0.495 g, 2.5 mmol, 1.0 equiv), which was suspended on 6.25 mL toluene (0.40 M). To the suspension was added finely ground NaOH powder (0.0999 g, 2.5 mmol, 1.0 equiv), and the mixture was stirred at 75 ℃ for 1.0 hour (colour change from pale yellow to white was observed). After the reaction, the white and powdery precipitate was filtered and dried at 70 ℃ under a high vacuum overnight, which was used directly for other reactions without further purification.

Method C: To a flame-dried round bottom flask (50 mL) equipped with a Teflon-coated magnetic stirring bar was added 4-biphenylcarboxylic acid (**1a**, 0.495 g, 2.5 mmol, 1.0 equiv), which was suspended on 6.25 mL toluene (0.40 M). To the suspension was added 20 M NaOH aqueous solution (0.125 mL, 2.5 mmol, 1.0 equiv), and the mixture was stirred at 75 ℃ for 1.0 hour (colour change from pale yellow to white was observed). After the reaction, the white and powdery precipitate was filtered and dried by airflow overnight, which was used directly for other reactions without further purification.

Method D: At room temperature, to 5.0 mL EtOH solution of 4-biphenylcarboxylic acid (**1a**, 0.396 g, 2.0 mmol, 1.0 equiv) was added the 0.40 M NaO*t*-Bu (5.0 mL in EtOH, 2.0 mmol, 1.0 equiv) solution dropwise, and the mixture was kept stirred for 1.0 hour (colour change from pale yellow to white was observed). After the reaction, the white and powdery precipitate was filtered and dried at 70 ℃ under a high vacuum overnight, which was used directly for other reactions without further purification.

Cautions:

- *As shown in the following optimization, proper preparation of the carboxylate alkaline salts is key to the high-yield deoxy-borylation.*
- *The excessive base could cause a reduction of reaction yield. Therefore, the scale of synthesizing the carboxylate should be sufficiently large to avoid the imprecision of weighing reagents.*

3. Supplementary Discussion

3.1. Alkali carboxylate deoxygenative borylation

3.1.1. Examination of the effect of different solvents

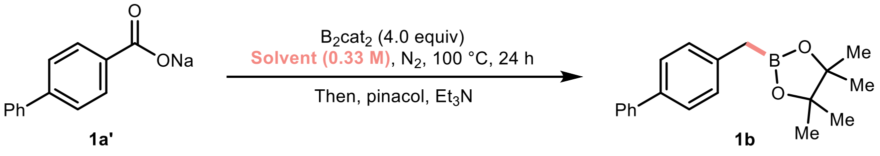

The procedure for examining the effect of different solvents was described below.

To a flame-dried reaction tube (10 mL) equipped with a Teflon-coated magnetic stirring bar were added 4-biphenylcarboxylic acid sodium salt (**1a'**, 22.0 mg, 0.10 mmol, 1.0 equiv) and B_2cat_2 (95.1 mg, 0.40 mmol, 4.0 equiv). The resulting mixture was degassed and back-filled with nitrogen (N_2, high purity 4.8, >99.998%) before being transferred to the nitrogen-filled glovebox. Shortly after, 0.30 mL designated solvent (0.33 M) was syringed into the reaction tube, which was capped by an aluminium seal with PTFE/silicone septum. The reaction tube was moved out of the glovebox and stirred at 100 °C for 24 hours.

Upon completion of the reaction, to the resulted crude was added pinacol (94.5 mg, 0.80 mmol, 8.0 equiv) and 0.35 mL Et_3N. Then, the mixture was stirred at ambient temperature for 1.0 hour. After adding the 1,3,5-trimethoxybenzene (16.8 mg, 0.10 mmol, 1.0 equiv) as internal standard, the reaction was quenched by 3.0 mL brine and 1.0 mL distilled H_2O and supplemented with 5.0 mL EtOAc for extraction. With vigorous shaking followed by unperturbed standing for a while to allow the two layers to separate, the upper organic layer was transferred and passed through a short-packed pipette column filled with Na_2SO_4 (3.0 cm) and silica gel (0.50 cm). The above-mentioned extraction process was repeated for another four times with EtOAc (5.0*4 mL). The filtered anhydrous organic solution (~25 mL) was collected in a 100 mL round bottom flask and concentrated on a rotary evaporator. The crude was subjected to ^1H NMR and GC-MS analysis for the reaction yield, and the results were summarized in the following table.

Supplementary Table 3.1.1. Effect of different solvents

entry	*solvent*	*NMR yield (**1b**)*
1	DMA	36%
2	NMP	10%
3	DMPU	38%
4	DMEU	20%
5	HMPA	43%
6	MeOH	ND
7	*i*-PrOH	ND
8	*t*-BuOH	ND
9	diethyl ether	ND
10	THF	ND
11	dioxane	ND
12	DME	ND
13	diglyme	ND
14	hexane	ND
15	cyclohexane	ND

16	benzene	ND
17	toluene	ND
18	Ph-CF_3	ND
19	DCM	ND
20	$CHCl_3$	ND
21	DCE	ND
22	DMSO	ND
23	CH_3CN	ND
24	EtOAc	ND
25	acetone	ND

Note: Unless otherwise specified, all the solvents used in the optimization were purchased from the chemical vendors and used directly without further purification.

3.1.2. Examination of the effect of reagent and solvent quality

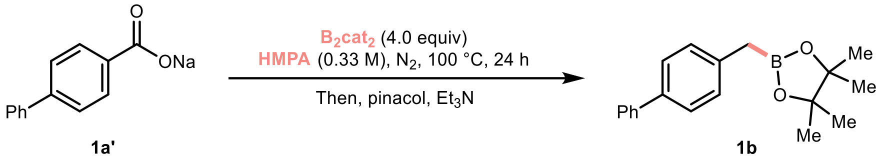

The procedure for examining the effect of reagent and solvent quality was described below.

To a flame-dried reaction tube (10 mL) equipped with a Teflon-coated magnetic stirring bar were added 4-biphenylcarboxylic acid sodium salt (**1a'**, 44.0 mg, 0.20 mmol, 1.0 equiv) and B_2cat_2 (190.2 mg, 0.80 mmol, 4.0 equiv) of specific quality. The resulting mixture was degassed and back-filled with nitrogen (N_2, high purity 4.8, >99.998%) before being transferred to the nitrogen-filled glovebox. Shortly after, 0.60 mL commercially available or distilled HMPA (0.33 M) was syringed into the reaction tube, which was capped by an aluminium seal with PTFE/silicone septum. The reaction tube was moved out of the glovebox and stirred at 100 °C for 24 hours.

Upon the completion of the reaction, to the resulted crude was added pinacol (189.1 mg, 1.6 mmol, 8.0 equiv) and 0.70 mL Et_3N. Then, the mixture was stirred at ambient temperature for 1.0 hour. After adding the 1,3,5-trimethoxybenzene (16.8 mg, 0.10 mmol, 1.0 equiv) as internal standard, it was quenched by 3.0 mL brine and 1.0 mL distilled H_2O and supplemented with 5.0 mL EtOAc for extraction. With vigorous shaking followed by unperturbed standing for a while to allow the two layers to separate, the upper organic layer was transferred and passed through a short-packed pipette column filled with Na_2SO_4 (3.0 cm) and silica gel (0.50 cm). The above-mentioned extraction process was repeated for another four times with EtOAc (5.0*4 mL). The filtered anhydrous organic solution (~25 mL) was collected in a 100 mL round bottom flask and concentrated on a rotary evaporator. The crude was subjected to ^{1}H NMR and GC-MS analysis for the reaction yield, and the results were summarized in the following table.

Supplementary Table 3.1.2. Effect of reagent and solvent quality

entry	*B_2cat_2 (4.0 equiv)*	*HMPA (0.33M)*	*NMR yield (**1b**)*
1	Commercial	Commercial	43%
2	Commercial	Distilled	58%

3	Purified	Commercial	53%
4	Purified	Distilled	59%

Note: Purification of the reagent and solvent is necessary for both high reaction yield and reproducibility since some impurities in the reagent, for instance, moisture and amine residue, could easily impact the reaction efficiency. The B_2cat_2 was evacuated under a high vacuum overnight. Purification of HMPA was based on some reports from the literature, which distilled the HMPA over metallic sodium under reduced pressure, dried it over 4 Å molecular sieves and stored it in an N_2-filled glovebox.[1] Unless otherwise specified, both B_2cat_2 and HMPA used for the following optimization were purified before use.

3.1.3. Examination of the effect of sodium benzoates prepared by different methods

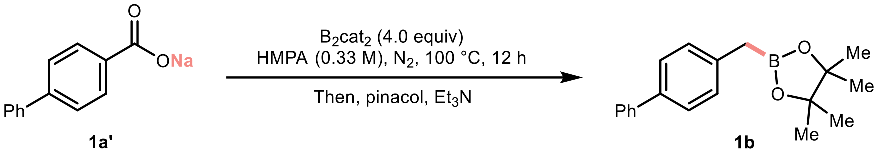

The procedure for examining the effect of sodium benzoates prepared by different methods was described below.

To a flame-dried reaction tube (10 mL) equipped with a Teflon-coated magnetic stirring bar were added B_2cat_2 (190.2 mg, 0.80 mmol, 4.0 equiv) and 4-biphenylcarboxylic acid sodium salt (**1a'**, 44.0 mg, 0.20 mmol, 1.0 equiv), which was prepared by different methods (see **Section 2** for details). The resulting mixture was degassed and back-filled with nitrogen (N_2, high purity 4.8, >99.998%) before being transferred to the nitrogen-filled glovebox. Shortly after, 0.60 mL HMPA (0.33 M) was syringed into the reaction tube, which was capped by an aluminium seal with PTFE/silicone septum. The reaction tube was moved out of the glovebox and stirred at 100 ℃ for 12 hours.

Upon the completion of the reaction, to the resulted crude was added pinacol (189.1 mg, 1.6 mmol, 8.0 equiv) and 0.70 mL Et_3N. Then, the mixture was stirred at ambient temperature for 1.0 hour. After adding the 1,3,5-trimethoxybenzene (16.8 mg, 0.10 mmol, 1.0 equiv) as internal standard, it was quenched by 3.0 mL brine and 1.0 mL distilled H_2O and supplemented with 5.0 mL EtOAc for extraction. With vigorous shaking followed by unperturbed standing for a while to allow the two layers to separate, the upper organic layer was transferred and passed through a short-packed pipette column filled with Na_2SO_4 (3.0 cm) and silica gel (0.50 cm). The above-mentioned extraction process was repeated for another four times with EtOAc (5.0*4 mL). The filtered anhydrous organic solution (~25 mL) was collected in a 100 mL round bottom flask and concentrated on a rotary evaporator. The crude was subjected to ^1H NMR and GC-MS analysis for the reaction yield, and the results were summarized in the following table.

Supplementary Table 3.1.3. Effect of sodium benzoates prepared by different methods

entry	*synthetic methods*	*NMR yield (**1b**)*
1	Method A	59%
2	Method B	53%
3	Method C	34%
4	Method D	45%

Note: For reaction efficiency and condition simplification, all the sodium benzoates used in this work were prepared by Method A.

3.1.4. Examination of the effect of different diboron reagents

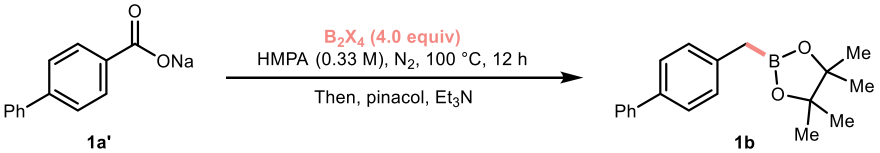

The procedure for examining the effect of different diboron reagents was described below.

To a flame-dried reaction tube (10 mL) equipped with a Teflon-coated magnetic stirring bar were added 4-biphenylcarboxylic acid sodium salt (**1a'**, 44.0 mg, 0.20 mmol, 1.0 equiv) and the corresponding diboron reagent or its equivalent (0.80 mmol, 4.0 equiv). The resulting mixture was degassed and back-filled with nitrogen (N_2, high purity 4.8, >99.998%) before being transferred to the nitrogen-filled glovebox. Shortly after, 0.60 mL HMPA (0.33 M) was syringed into the reaction tube, which was capped by an aluminium seal with PTFE/silicone septum. The reaction tube was moved out of the glovebox and stirred at 100 ℃ for 12 hours.

Upon the completion of the reaction, if necessary, to the resulted crude was added pinacol (189.1 mg, 1.6 mmol, 8.0 equiv) and 0.70 mL Et_3N. Then, the mixture was stirred at ambient temperature for 1.0 hour. After adding the 1,3,5-trimethoxybenzene (16.8 mg, 0.10 mmol, 1.0 equiv) as internal standard, it was quenched by 3.0 mL brine and 1.0 mL distilled H_2O and supplemented with 5.0 mL EtOAc for extraction. With vigorous shaking followed by unperturbed standing for a while to allow the two layers to separate, the upper organic layer was transferred and passed through a short-packed pipette column filled with Na_2SO_4 (3.0 cm) and silica gel (0.50 cm). The above-mentioned extraction process was repeated for another four times with EtOAc (5.0*4 mL). The filtered anhydrous organic solution (~25 mL) was collected in a 100 mL round bottom flask and concentrated on a rotary evaporator. The crude was subjected to ^1H NMR and GC-MS analysis for the reaction yield, and the results were summarized in the following table.

Supplementary Table 3.1.4. Effect of different diboron reagents

entry	*B_2X_4 (4.0 equiv)*	*NMR yield (**1b**)*
1	B_2cat_2	59%
2	B_2pin_2	0%
3	$B_2(OH)_4$	0%
4	$B_2(OH)_4$ +catechol (1:2)	0%

3.1.5. Examination of the effect of B_2cat_2 loading

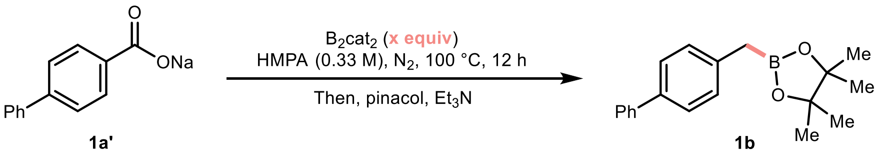

The procedure for examining the effect of B_2cat_2 loadings was described below.

To a flame-dried reaction tube (10 mL) equipped with a Teflon-coated magnetic stirring bar were added 4-biphenylcarboxylic acid sodium salt (**1a'**, 44.0 mg, 0.20 mmol, 1.0 equiv) and B_2cat_2 (*x* equiv). The resulting mixture was degassed and back-filled with nitrogen (N_2, high purity 4.8, >99.998%) before being transferred to the nitrogen-filled glovebox. Shortly after, 0.60 mL HMPA (0.33 M) was syringed into the

reaction tube, which was capped by an aluminium seal with PTFE/silicone septum. The reaction tube was moved out of the glovebox and stirred at 100 ℃ for 12 hours.

Upon the completion of the reaction, to the resulted crude was added pinacol (2*x* equiv) and 0.70 mL Et_3N. Then, the mixture was stirred at ambient temperature for 1.0 hour. After adding the 1,3,5-trimethoxybenzene (16.8 mg, 0.10 mmol, 1.0 equiv) as internal standard, it was quenched by 3.0 mL brine and 1.0 mL distilled H_2O and supplemented with 5.0 mL EtOAc for extraction. With vigorous shaking followed by unperturbed standing for a while to allow the two layers to separate, the upper organic layer was transferred and passed through a short-packed pipette column filled with Na_2SO_4 (3.0 cm) and silica gel (0.50 cm). The above-mentioned extraction process was repeated for another four times with EtOAc (5.0*4 mL). The filtered anhydrous organic solution (~25 mL) was collected in a 100 mL round bottom flask and concentrated on a rotary evaporator. The crude was subjected to ^{1}H NMR and GC-MS analysis for the reaction yield, and the results were summarized in the following table.

Supplementary Table 3.1.5. Effect of different B_2cat_2 loadings

entry	*B_2cat_2 (x equiv)*	*NMR yield (**1b**)*
1	x = 3	40%
2	x = 4	59%
3	x = 5	54%

3.1.6. Examination of the effect of counter cations of different carboxylates

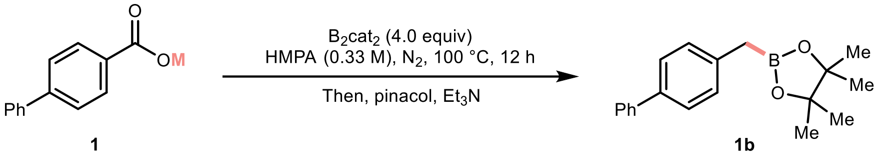

1 **1b**

The procedure for examining the effect of different alkaline carboxylates was described below.

To a flame-dried reaction tube (10 mL) equipped with a Teflon-coated magnetic stirring bar were added the corresponding 4-biphenylcarboxylate salt (0.20 mmol, 1.0 equiv based on B_2cat_2) and B_2cat_2 (190.2 mg, 0.80 mmol, 4.0 equiv). The resulting mixture was degassed and back-filled with nitrogen (N_2, high purity 4.8, >99.998%) before being transferred to the nitrogen-filled glovebox. Shortly after, 0.60 mL HMPA (0.33 M) was syringed into the reaction tube, which was capped by an aluminium seal with PTFE/silicone septum. The reaction tube was moved out of the glovebox and stirred at 100 ℃ for 12 hours.

Upon the completion of the reaction, to the resulted crude was added pinacol (189.1 mg, 1.6 mmol, 8.0 equiv) and 0.70 mL Et_3N. Then, the mixture was stirred at ambient temperature for 1.0 hour. After adding the 1,3,5-trimethoxybenzene (16.8 mg, 0.10 mmol, 1.0 equiv) as internal standard, it was quenched by 3.0 mL brine and 1.0 mL distilled H_2O and supplemented with 5.0 mL EtOAc for extraction. With vigorous shaking followed by unperturbed standing for a while to allow the two layers to separate, the upper organic layer was transferred and passed through a short-packed pipette column filled with Na_2SO_4 (3.0 cm) and silica gel (0.50 cm). The above-mentioned extraction process was repeated for another four times with EtOAc (5.0*4 mL). The filtered anhydrous organic solution (~25 mL) was collected in a 100 mL round bottom flask and concentrated on a rotary evaporator. The crude was subjected to ^{1}H NMR and GC-MS analysis for the reaction yield, and the results were summarized in the following table.

Supplementary Table 3.1.6. Effect of different counter cations of the carboxylates

entry	*4-Ph-C_6H_4-CO_2M*	*NMR yield (**1b**)*
1	M = Li	46%
2	M = Na	59%
3	M = K	53%
4	M = Cs	54%
5	M = ½ Mg	51%

3.1.7. Examination of the effect of different basic additives

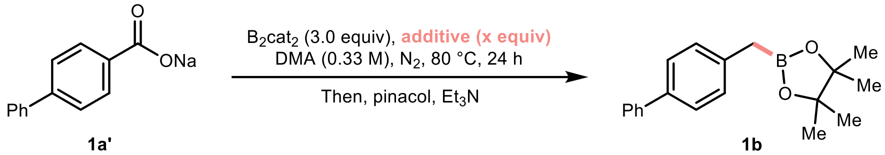

The procedure for examining the effect of different basic additives was described below.

To a flame-dried reaction tube (10 mL) equipped with a Teflon-coated magnetic stirring bar were added 4-biphenylcarboxylic acid sodium salt (**1a'**, 44.0 mg, 0.20 mmol, 1.0 equiv), B_2cat_2 (142.7 mg, 0.60 mmol, 3.0 equiv) and the corresponding additives. The resulting mixture was degassed and back-filled with nitrogen (N_2, high purity 4.8, >99.998%) before being transferred to the nitrogen-filled glovebox. Shortly after, 0.60 mL DMA (0.33 M) was syringed into the reaction tube, which was capped by an aluminium seal with PTFE/silicone septum. The reaction tube was moved out of the glovebox and stirred at 80 ℃ for 24 hours.

Upon completion of the reaction, to the resulted crude was added pinacol (141.8 mg, 1.2 mmol, 6.0 equiv) and 0.70 mL Et_3N. Then, the mixture was stirred at ambient temperature for 1.0 hour. After adding the 1,3,5-trimethoxybenzene (16.8 mg, 0.10 mmol, 1.0 equiv) as internal standard, the reaction was quenched by 3.0 mL brine and 1.0 mL distilled H_2O and supplemented with 5.0 mL EtOAc for extraction. With vigorous shaking followed by unperturbed standing for a while to allow the two layers to separate, the upper organic layer was transferred and passed through a short-packed pipette column filled with Na_2SO_4 (3.0 cm) and silica gel (0.50 cm). The above-mentioned extraction process was repeated for another four times with EtOAc (5.0*4 mL). The filtered anhydrous organic solution (~25 mL) was collected in a 100 mL round bottom flask and concentrated on a rotary evaporator. The crude was subjected to ^{1}H NMR and GC-MS analysis for the reaction yield, and the results were summarized in the following table.

Supplementary Table 3.1.7. Effect of different basic additives

entry	*additive (loading)*	*NMR yield (**1b**)*
1	none	27%
2	NaOMe (1.0 equiv)	2%
3	NaO*t*-Bu (1.0 equiv)	3%
4	Na_2CO_3 (1.0 equiv)	trace
5	$NaHCO_3$ (1.0 equiv)	4%
6	NaOAc (1.0 equiv)	2%
7	DIPEA (1.0 equiv)	11%

3.1.8. Examination of the effect of different reducing additives

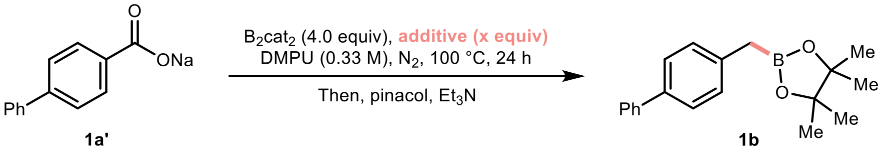

1a' 1b

The procedure for examining the effect of different reducing additives was described below.

To a flame-dried reaction tube (10 mL) equipped with a Teflon-coated magnetic stirring bar were added 4-biphenylcarboxylic acid sodium salt (**1a'**, 44.0 mg, 0.20 mmol, 1.0 equiv), B_2cat_2 (190.2 mg, 0.80 mmol, 4.0 equiv) and the corresponding additives. The resulting mixture was degassed and back-filled with nitrogen (N_2, high purity 4.8, >99.998%) before being transferred to the nitrogen-filled glovebox. Shortly after, 0.60 mL DMPU (0.33 M) was syringed into the reaction tube, which was capped by an aluminium seal with PTFE/silicone septum. The reaction tube was moved out of the glovebox and stirred at 100 ℃ for 24 hours.

Upon completion of the reaction, to the resulted crude was added pinacol (189.1 mg, 1.6 mmol, 8.0 equiv) and 0.70 mL Et_3N. Then, the mixture was stirred at ambient temperature for 1.0 hour. After adding the 1,3,5-trimethoxybenzene (16.8 mg, 0.10 mmol, 1.0 equiv) as internal standard, the reaction was quenched by 3.0 mL brine and 1.0 mL distilled H_2O and supplemented with 5.0 mL EtOAc for extraction. With vigorous shaking followed by unperturbed standing for a while to allow the two layers to separate, the upper organic layer was transferred and passed through a short-packed pipette column filled with Na_2SO_4 (3.0 cm) and silica gel (0.50 cm). The above-mentioned extraction process was repeated for another four times with EtOAc (5.0*4 mL). The filtered anhydrous organic solution (~25 mL) was collected in a 100 mL round bottom flask and concentrated on a rotary evaporator. The crude was subjected to ^{1}H NMR and GC-MS analysis for the reaction yield, and the results were summarized in the following table.

Supplementary Table 3.1.8. Effect of different reducing additives

entry	*additive (loading)*	*NMR yield (**1b**)*
1	none	38%
2	H-Bcat (1.0 equiv)	27%[a]
3	H-Bpin (1.0 equiv)	35%[a]
4	B_2pin_2 (1.0 equiv)	38%
5	B_2pin_2 (2.0 equiv)	40%
6	$B_2(OH)_4$ (1.0 equiv)	35%
7	$B_2(OH)_4$ (2.0 equiv)	18%
8	$NaBH_4$ (1.0 equiv)	ND
9	$PhSiH_3$ (1.0 equiv)	ND[a]
10	TMDS (1.0 equiv)	19%[a]
11	HCO_2Na (1.0 equiv)	14%
12	PPh_3 (1.0 equiv)	35%

[a]The additive was added in the nitrogen-filled glovebox.

3.1.9. Examination of the effect of different electrophilic additives

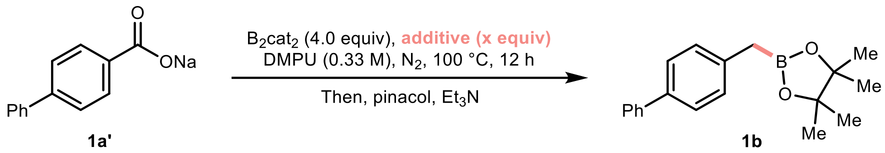

The procedure for examining the effect of different electrophilic additives was described below.

To a flame-dried reaction tube (10 mL) equipped with a Teflon-coated magnetic stirring bar were added 4-biphenylcarboxylic acid sodium salt (**1a'**, 44.0 mg, 0.20 mmol, 1.0 equiv), B_2cat_2 (190.2 mg, 0.80 mmol, 4.0 equiv) and the corresponding additives. The resulting mixture was degassed and back-filled with nitrogen (N_2, high purity 4.8, >99.998%) before being transferred to the nitrogen-filled glovebox. Shortly after, 0.60 mL DMPU (0.33 M) was syringed into the reaction tube, which was capped by an aluminium seal with PTFE/silicone septum. The reaction tube was moved out of the glovebox and stirred at 100 ℃ for 12 hours.

Upon completion of the reaction, to the resulted crude was added pinacol (189.1 mg, 1.6 mmol, 8.0 equiv) and 0.70 mL Et_3N. Then, the mixture was stirred at ambient temperature for 1.0 hour. After adding the 1,3,5-trimethoxybenzene (16.8 mg, 0.10 mmol, 1.0 equiv) as internal standard, the reaction was quenched by 3.0 mL brine and 1.0 mL distilled H_2O and supplemented with 5.0 mL EtOAc for extraction. With vigorous shaking followed by unperturbed standing for a while to allow the two layers to separate, the upper organic layer was transferred and passed through a short-packed pipette column filled with Na_2SO_4 (3.0 cm) and silica gel (0.50 cm). The above-mentioned extraction process was repeated for another four times with EtOAc (5.0*4 mL). The filtered anhydrous organic solution (~25 mL) was collected in a 100 mL round bottom flask and concentrated on a rotary evaporator. The crude was subjected to ^1H NMR and GC-MS analysis for the reaction yield, and the results were summarized in the following table.

Supplementary Table 3.1.9. Effect of different electrophilic additives

entry	*additive (loading)*	*NMR yield (**1b**)*
1	none	43%
2	DMC (1.0 equiv)	39%
3	$B(OMe)_3$ (1.0 equiv)	39%
4	TFAA (1.0 equiv)	29%
5	Tf_2O (1.0 equiv)	32%
6	Me_2N-CO-Cl (1.0 equiv)	35%

3.1.10. Examination of the effect of other additives

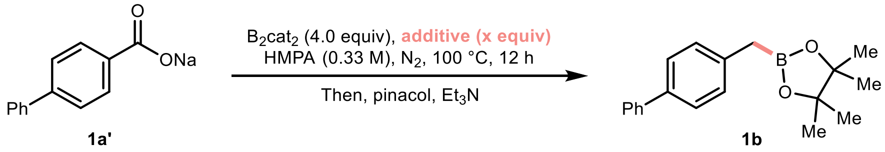

The procedure for examining the effect of other additives was described below.

To a flame-dried reaction tube (10 mL) equipped with a Teflon-coated magnetic stirring bar were added 4-biphenylcarboxylic acid sodium salt (**1a'**, 44.0 mg, 0.20 mmol, 1.0 equiv), B_2cat_2 (190.2 mg, 0.80 mmol, 4.0 equiv) and the corresponding additives. The resulting mixture was degassed and back-filled with nitrogen (N_2, high purity 4.8, >99.998%) before being transferred to the nitrogen-filled glovebox. Shortly

after, 0.60 mL HMPA (0.33 M) was syringed into the reaction tube, which was capped by an aluminum seal with PTFE/silicone septum. The reaction tube was moved out of the glovebox and stirred at 100 ℃ for 12 hours.

Upon the completion of the reaction, to the resulted crude was added pinacol (189.1 mg, 1.6 mmol, 8.0 equiv) and 0.70 mL Et_3N. Then, the mixture was stirred at ambient temperature for 1.0 hour. After adding the 1,3,5-trimethoxybenzene (16.8 mg, 0.10 mmol, 1.0 equiv) as internal standard, it was quenched by 3.0 mL brine and 1.0 mL distilled H_2O and supplemented with 5.0 mL EtOAc for extraction. With vigorous shaking followed by unperturbed standing for a while to allow the two layers to separate, the upper organic layer was transferred and passed through a short-packed pipette column filled with Na_2SO_4 (3.0 cm) and silica gel (0.50 cm). The above-mentioned extraction process was repeated for another four times with EtOAc (5.0*4 mL). The filtered anhydrous organic solution (~25 mL) was collected in a 100 mL round bottom flask and concentrated on a rotary evaporator. The crude was subjected to ^{1}H NMR and GC-MS analysis for the reaction yield, and the results were summarized in the following table.

Supplementary Table 3.1.10. Effect of other additives

entry	*additive (loading)*	*NMR yield (**1b**)*
1	none	59%
2	15-crown-5 (1.0 equiv)	51%
3	$B(C_6F_5)_3$ (5 mol%)	48%
4	NaI (1.0 equiv)	48%
5	NaI (3.0 equiv)	38%
6	NaI (5.0 equiv)	20%
7	$B(OH)_3$ (1.0 equiv)	48%
8	H_2O (2.0 equiv)	29%

3.1.11. Examination of the effect of drying agents

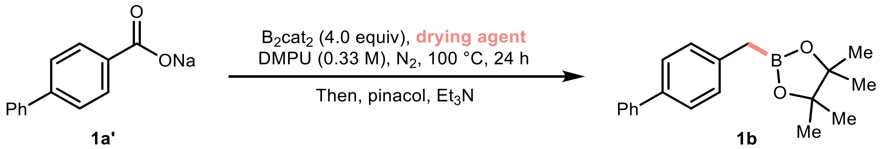

The procedure for examining the effect of drying agents was described below.

To a flame-dried reaction tube (10 mL) equipped with a Teflon-coated magnetic stirring bar were added 4-biphenylcarboxylic acid sodium salt (**1a'**, 44.0 mg, 0.20 mmol, 1.0 equiv), B_2cat_2 (190.2 mg, 0.80 mmol, 4.0 equiv) and the drying agent. The resulting mixture was degassed and back-filled with nitrogen (N_2, high purity 4.8, >99.998%) before being transferred to the nitrogen-filled glovebox. Shortly after, 0.60 mL DMPU (0.33 M) was syringed into the reaction tube, which was capped by an aluminium seal with PTFE/silicone septum. The reaction tube was moved out of the glovebox and stirred at 100 ℃ for 24 hours.

Upon completion of the reaction, to the resulted crude was added pinacol (189.1 mg, 1.6 mmol, 8.0 equiv) and 0.70 mL Et_3N. Then, the mixture was stirred at ambient temperature for 1.0 hour. After adding the 1,3,5-trimethoxybenzene (16.8 mg, 0.10 mmol, 1.0 equiv) as internal standard, the reaction was quenched by 3.0 mL brine and 1.0 mL distilled H_2O and supplemented with 5.0 mL EtOAc for extraction. With vigorous shaking followed by unperturbed standing for a while to allow the two layers to separate,

the upper organic layer was transferred and passed through a short-packed pipette column filled with Na_2SO_4 (3.0 cm) and silica gel (0.50 cm). The above-mentioned extraction process was repeated for another four times with EtOAc (5.0*4 mL). The filtered anhydrous organic solution (~25 mL) was collected in a 100 mL round bottom flask and concentrated on a rotary evaporator. The crude was subjected to ^1H NMR and GC-MS analysis for the reaction yield, and the results were summarized in the following table.

Supplementary Table 3.1.11. Effect of different drying agents

entry	*drying agent (loading)*	*NMR yield (**1b**)*
1	none	38%
2	Na_2SO_4 (1.0 equiv)	36%
3	$MgSO_4$ (0.50 equiv)	40%
4	$MgSO_4$ (1.0 equiv)	40%
5	$CaCl_2 \cdot 2H_2O$ (1.0 equiv)	trace
6	4 Å MS (25.0 mg)	32%

3.1.12. Examination of the effect of different temperature

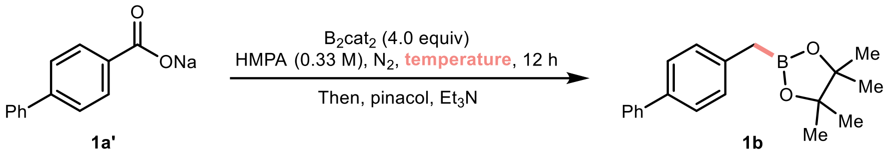

The procedure for examining the effect of different temperatures was described below.

To a flame-dried reaction tube (10 mL) equipped with a Teflon-coated magnetic stirring bar were added 4-biphenylcarboxylic acid sodium salt (**1a’**, 44.0 mg, 0.20 mmol, 1.0 equiv) and B_2cat_2 (190.2 mg, 0.80 mmol, 4.0 equiv). The resulting mixture was degassed and back-filled with nitrogen (N_2, high purity 4.8, >99.998%) before being transferred to the nitrogen-filled glovebox. Shortly after, 0.60 mL HMPA (0.33 M) was syringed into the reaction tube, which was capped by an aluminum seal with PTFE/silicone septum. The reaction tube was moved out of the glovebox and stirred at the designated temperature for 12 hours.

Upon the completion of the reaction, to the resulted crude was added pinacol (189.1 mg, 1.6 mmol, 8.0 equiv) and 0.70 mL Et_3N. Then, the mixture was stirred at ambient temperature for 1.0 hour. After adding the 1,3,5-trimethoxybenzene (16.8 mg, 0.10 mmol, 1.0 equiv) as internal standard, it was quenched by 3.0 mL brine and 1.0 mL distilled H_2O and supplemented with 5.0 mL EtOAc for extraction. With vigorous shaking followed by unperturbed standing for a while to allow the two layers to separate, the upper organic layer was transferred and passed through a short-packed pipette column filled with Na_2SO_4 (3.0 cm) and silica gel (0.50 cm). The above-mentioned extraction process was repeated for another four times with EtOAc (5.0*4 mL). The filtered anhydrous organic solution (~25 mL) was collected in a 100 mL round bottom flask and concentrated on a rotary evaporator. The crude was subjected to ^1H NMR and GC-MS analysis for the reaction yield, and the results were summarized in the following table.

Supplementary Table 3.1.12. Effect of different temperature

entry	*reaction temperature*	*NMR yield (**1b**)*
1	120 ℃	54%
2	100 ℃	59%

3	75 °C	42%
4	50 °C	0%
5	25 °C	0%

3.1.13. Examination of the effect of different reaction time

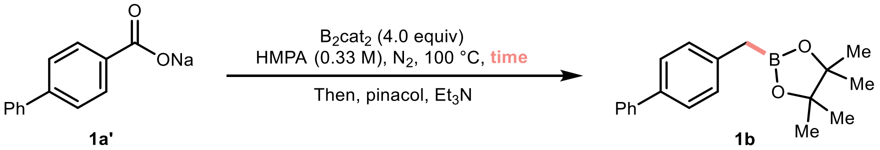

The procedure for examining the effect of different reaction time was described below.

To a flame-dried reaction tube (10 mL) equipped with a Teflon-coated magnetic stirring bar were added 4-biphenylcarboxylic acid sodium salt (**1a'**, 44.0 mg, 0.20 mmol, 1.0 equiv) and B_2cat_2 (190.2 mg, 0.80 mmol, 4.0 equiv). The resulting mixture was degassed and back-filled with nitrogen (N_2, high purity 4.8, >99.998%) before being transferred to the nitrogen-filled glovebox. Shortly after, 0.60 mL HMPA (0.33 M) was syringed into the reaction tube, which was capped by an aluminum seal with PTFE/silicone septum. The reaction tube was moved out of the glovebox and stirred at 100 °C for different reaction time.

Upon the completion of the reaction, to the resulted crude was added pinacol (189.1 mg, 1.6 mmol, 8.0 equiv) and 0.70 mL Et_3N. Then, the mixture was stirred at ambient temperature for 1.0 hour. After adding the 1,3,5-trimethoxybenzene (16.8 mg, 0.10 mmol, 1.0 equiv) as internal standard, it was quenched by 3.0 mL brine and 1.0 mL distilled H_2O and supplemented with 5.0 mL EtOAc for extraction. With vigorous shaking followed by unperturbed standing for a while to allow the two layers to separate, the upper organic layer was transferred and passed through a short-packed pipette column filled with Na_2SO_4 (3.0 cm) and silica gel (0.50 cm). The above-mentioned extraction process was repeated for another four times with EtOAc (5.0*4 mL). The filtered anhydrous organic solution (~25 mL) was collected in a 100 mL round bottom flask and concentrated on a rotary evaporator. The crude was subjected to ^{1}H NMR and GC-MS analysis for the reaction yield, and the results were summarized in the following table.

Supplementary Table 3.1.12. Effect of different reaction time

entry	*reaction time*	*NMR yield (**1b**)*
1	6 h	44%
2	9 h	54%
3	12 h	59%
4	24 h	55%

3.1.14. Control experiments

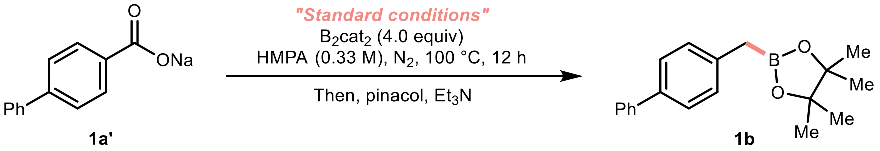

The procedure for the control experiments with sodium carboxylate was described below.

To a flame-dried reaction tube (10 mL) equipped with a Teflon-coated magnetic stirring bar were added 4-biphenylcarboxylic acid sodium salt (**1a'**, 44.0 mg, 0.20 mmol, 1.0 equiv), B_2cat_2 (190.2 mg, 0.80 mmol,

4.0 equiv) and the corresponding control reagent/catalyst. The resulting mixture was degassed and back-filled with nitrogen (N_2, high purity 4.8, >99.998%) before being transferred to the nitrogen-filled glovebox. Shortly after, 0.60 mL HMPA (0.33 M) was syringed into the reaction tube, which was capped by an aluminum seal with PTFE/silicone septum. The reaction tube was moved out of the glovebox and stirred at 100 ℃ for 12 hours under nitrogen or air.

Upon the completion of the reaction, to the resulted crude was added pinacol (189.1 mg, 1.6 mmol, 8.0 equiv) and 0.70 mL Et_3N. Then, the mixture was stirred at ambient temperature for 1.0 hour. After adding the 1,3,5-trimethoxybenzene (16.8 mg, 0.10 mmol, 1.0 equiv) as internal standard, it was quenched by 3.0 mL brine and 1.0 mL distilled H_2O and supplemented with 5.0 mL EtOAc for extraction. With vigorous shaking followed by unperturbed standing for a while to allow the two layers to separate, the upper organic layer was transferred and passed through a short-packed pipette column filled with Na_2SO_4 (3.0 cm) and silica gel (0.50 cm). The above-mentioned extraction process was repeated for another four times with EtOAc (5.0*4 mL). The filtered anhydrous organic solution (~25 mL) was collected in a 100 mL round bottom flask and concentrated on a rotary evaporator. The crude was subjected to ^1H NMR and GC-MS analysis for the reaction yield, and the results were summarized in the following table.

Supplementary Table 3.1.13. Effect of other reaction parameters

entry	*variations from standards conditions*	*NMR yield (**1b**)*
1	none	59%
2	under air	43%
3	+ ICyCuCl (5.0 mol%)	30%
4	+ $FeBr_2$ (10 mol%)	60%
5	$Ni(COD)_2$ (10 mol%)	46%
6	+ $[Rh(COD)Cl]_2$ (5.0 mol%)	20%
7	+ $Ru(PPh_3)_3Cl_2$ (10 mol%)	18%
8	+ $[Ir(COD)Cl]_2$ (5.0 mol%)	15%

Note:

- *For the under-air experiment, the reaction tube was filled with compressed air before being stirred with heat.*
- *The ICyCuCl and anhydrous $FeBr_2$ catalysts were shown effective in the deoxygenative borylation of aldehydes/ketones and esters, respectively.[2, 3] The former were prepared via in-situ mixing copper (I) chloride (CuCl, 1.1 mg, 0.011 mmol, 5.6 mol%), 1,3-dicyclohexylimidazolium chloride (ICy·HCl, 2.7 mg, 0.010 mmol, 5.0 mol%) and NaOt-Bu (1.0 mg, 0.011 mmol, 5.4 mol%), which was followed by the addition of 0.30 mL HMPA and stirred for 30 minutes to pre-form the metal complex ICyCuCl. The anhydrous $FeBr_2$ was purchased from Strem Chemicals and stored in the glovebox before use. Other representative transition metal catalysts (e.g., [Ni], [Rh], [Ru] and [Ir]), which have been shown effective in C-H/C-X borylation, were also examined to evaluate their potential involvement as trace residues in this deoxygenative borylation.*
- *Neither of the tested catalysts gave improved yields than the standard conditions, indicating that the carboxylic acid sodium salt deoxygenative borylation does not require transition metals.*

3.2. Free carboxylic acid deoxygenative borylation

3.2.1. Examination of the effect of different solvents

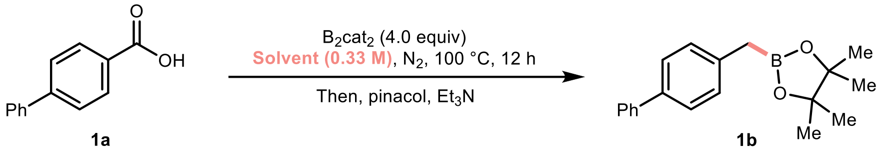

The procedure for examining the effect of different solvents was described below.

To a flame-dried reaction tube (10 mL) equipped with a Teflon-coated magnetic stirring bar were added 4-biphenylcarboxylic acid (**1a**, 39.6 mg, 0.20 mmol, 1.0 equiv) and B_2cat_2 (190.2 mg, 0.80 mmol, 4.0 equiv). The resulting mixture was degassed and back-filled with nitrogen (N_2, high purity 4.8, >99.998%) before being transferred to the nitrogen-filled glovebox. Shortly after, 0.60 mL designated solvent (0.33 M) was syringed into the reaction tube, which was capped by an aluminum seal with PTFE/silicone septum. The reaction tube was moved out of the glovebox and stirred at 100 ℃ for 12 hours.

Upon the completion of the reaction, to the resulted crude was added pinacol (189.1 mg, 1.6 mmol, 8.0 equiv) and 0.70 mL Et_3N. Then, the mixture was stirred at ambient temperature for 1.0 hour. After adding the 1,3,5-trimethoxybenzene (16.8 mg, 0.10 mmol, 1.0 equiv) as internal standard, it was quenched by 3.0 mL brine and 1.0 mL distilled H_2O and supplemented with 5.0 mL EtOAc for extraction. With vigorous shaking followed by unperturbed standing for a while to allow the two layers to separate, the upper organic layer was transferred and passed through a short-packed pipette column filled with Na_2SO_4 (3.0 cm) and silica gel (0.50 cm). The above-mentioned extraction process was repeated for another four times with EtOAc (5.0*4 mL). The filtered anhydrous organic solution (~25 mL) was collected in a 100 mL round bottom flask and concentrated on a rotary evaporator. The crude was subjected to ^{1}H NMR and GC-MS analysis for the reaction yield, and the results were summarized in the following table.

Supplementary Table 3.2.1. Effect of different solvents

entry	*solvent*	*NMR yield (**1b**)*
1	DMA	54%
2	NMP	45%
3	DMPU	45%
4	HMPA	61%[a], 55%[b]
5	MeOH	ND
6	THF	ND
7	hexane	ND
8	toluene	ND
9	Ph-CF_3	ND
10	DCM	ND
11	$CHCl_3$	ND
12	DMSO	ND
13	CH_3NO_2	ND
14	CH_3CN	ND
15	EtOAc	ND
16	acetone	ND

17	DMC	ND

[a]*Purification of HMPA was based on some reports from the literature, which distilled the HMPA over metallic sodium under reduced pressure, dried it over 4 Å molecular sieves and stored it in an N_2-filled glovebox.*[1] *Unless otherwise specified, both B_2cat_2 and HMPA used for the following optimization were purified before use.* [b]*Although HMPA from the commercial source could be used directly and give a similar yield, the ^{1}H NMR spectrum of its crude looked messier with some unidentified side products.*

3.2.2. Examination of the effect of water content

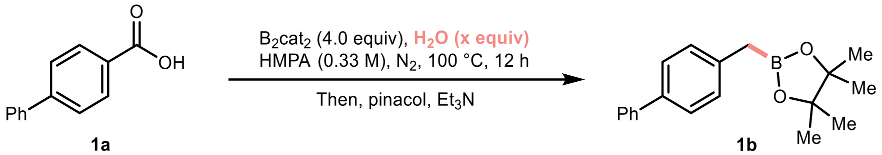

1a **1b**

The procedure for examining the effect of different water content was described below.

To a flame-dried reaction tube (10 mL) equipped with a Teflon-coated magnetic stirring bar were added 4-biphenylcarboxylic acid (**1a**, 39.6 mg, 0.20 mmol, 1.0 equiv) and B_2cat_2 (190.2 mg, 0.80 mmol, 4.0 equiv). The resulting mixture was degassed and back-filled with nitrogen (N_2, high purity 4.8, >99.998%) before being transferred to the nitrogen-filled glovebox. Shortly after, 0.60 mL HMPA (0.33 M), followed by the addition of a designated quantity of water, was syringed into the reaction tube, which was capped by an aluminum seal with PTFE/silicone septum. The reaction was moved out of the glovebox and stirred at 100 °C for 12 hours.

Upon the completion of the reaction, to the resulted crude was added pinacol (189.1 mg, 1.6 mmol, 8.0 equiv) and 0.70 mL Et_3N. Then, the mixture was stirred at ambient temperature for 1.0 hour. After adding the 1,3,5-trimethoxybenzene (16.8 mg, 0.10 mmol, 1.0 equiv) as internal standard, it was quenched by 3.0 mL brine and 1.0 mL distilled H_2O and supplemented with 5.0 mL EtOAc for extraction. With vigorous shaking followed by unperturbed standing for a while to allow the two layers to separate, the upper organic layer was transferred and passed through a short-packed pipette column filled with Na_2SO_4 (3.0 cm) and silica gel (0.50 cm). The above-mentioned extraction process was repeated for another four times with EtOAc (5.0*4 mL). The filtered anhydrous organic solution (~25 mL) was collected in a 100 mL round bottom flask and concentrated on a rotary evaporator. The crude was subjected to ^{1}H NMR and GC-MS analysis for the reaction yield, and the results were summarized in the following table.

Supplementary Table 3.2.2. Effect of water content

entry	*H_2O (x equiv)*	*NMR yield (**1b**)*
1	x = 0	61%
2	x = 2.5	26%
3	x = 5.0	13%
4	x = 10.0	0%
5	x = 20.0	0%

3.2.3. Examination of the effect of different diboron reagents

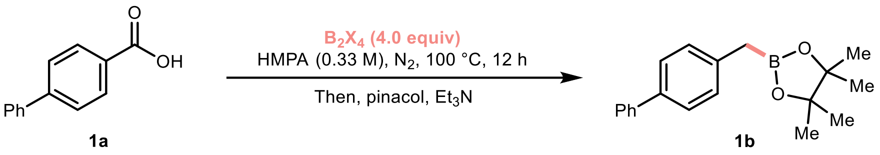

The procedure for examining the effect of different diboron reagents was described below.

To a flame-dried reaction tube (10 mL) equipped with a Teflon-coated magnetic stirring bar were added 4-biphenylcarboxylic acid (**1a**, 39.6 mg, 0.20 mmol, 1.0 equiv) and the corresponding diboron reagent or its equivalent (0.80 mmol, 4.0 equiv). The resulting mixture was degassed and back-filled with nitrogen (N_2, high purity 4.8, >99.998%) before being transferred to the nitrogen-filled glovebox. Shortly after, 0.60 mL HMPA (0.33 M) was syringed into the reaction tube, which was capped by an aluminum seal with PTFE/silicone septum. The reaction was moved out of the glovebox and stirred at 100 ℃ for 12 hours.

Upon the completion of the reaction, if necessary, to the resulted crude was added pinacol (189.1 mg, 1.6 mmol, 8.0 equiv) and 0.70 mL Et_3N. Then, the mixture was stirred at ambient temperature for 1.0 hour. After adding the 1,3,5-trimethoxybenzene (16.8 mg, 0.10 mmol, 1.0 equiv) as internal standard, it was quenched by 3.0 mL brine and 1.0 mL distilled H_2O and supplemented with 5.0 mL EtOAc for extraction. With vigorous shaking followed by unperturbed standing for a while to allow the two layers to separate, the upper organic layer was transferred and passed through a short-packed pipette column filled with Na_2SO_4 (3.0 cm) and silica gel (0.50 cm). The above-mentioned extraction process was repeated for another four times with EtOAc (5.0*4 mL). The filtered anhydrous organic solution (~25 mL) was collected in a 100 mL round bottom flask and concentrated on a rotary evaporator. The crude was subjected to ^1H NMR and GC-MS analysis for the reaction yield, and the results were summarized in the following table.

Supplementary Table 3.2.3. Effect of different diboron reagents

entry	*B_2X_4 (4.0 equiv)*	*NMR yield (**1b**)*
1	B_2cat_2	61%
2	B_2pin_2	0%
3	$B_2(OH)_4$	0%
4	$B_2(OH)_4$ +catechol (1:2)	0%

3.2.4. Examination of the effect of the loading of B_2cat_2

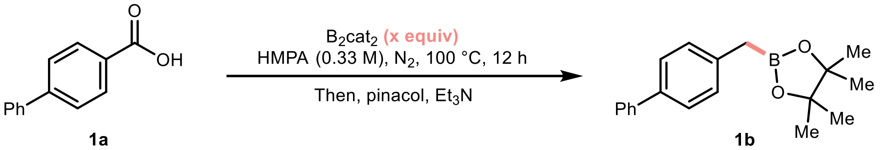

The procedure for examining the effect of different B_2cat_2 loadings was described below.

To a flame-dried reaction tube (10 mL) equipped with a Teflon-coated magnetic stirring bar were added 4-biphenylcarboxylic acid (**1a**, 39.6 mg, 0.20 mmol, 1.0 equiv) and B_2cat_2 of the corresponding amount (*x* equiv). The resulting mixture was degassed and back-filled with nitrogen (N_2, high purity 4.8, >99.998%) before being transferred to the nitrogen-filled glovebox. Shortly after, 0.60 mL HMPA (0.33 M) was syringed into the reaction tube, which was capped by an aluminum seal with PTFE/silicone septum. The reaction was moved out of the glovebox and stirred at 100 ℃ for 12 hours.

Upon the completion of the reaction, to the resulted crude was added pinacol (2*x* equiv) and 0.70 mL Et_3N. Then, the mixture was stirred at ambient temperature for 1.0 hour. After adding the 1,3,5-trimethoxybenzene (16.8 mg, 0.10 mmol, 1.0 equiv) as internal standard, it was quenched by 3.0 mL brine and 1.0 mL distilled H_2O and supplemented with 5.0 mL EtOAc for extraction. With vigorous shaking followed by unperturbed standing for a while to allow the two layers to separate, the upper organic layer was transferred and passed through a short-packed pipette column filled with Na_2SO_4 (3.0 cm) and silica gel (0.50 cm). The above-mentioned extraction process was repeated for another four times with EtOAc (5.0*4 mL). The filtered anhydrous organic solution (~25 mL) was collected in a 100 mL round bottom flask and concentrated on a rotary evaporator. The crude was subjected to ^1H NMR and GC-MS analysis for the reaction yield, and the results were summarized in the following table.

Supplementary Table 3.2.4. Effect of the B_2cat_2 loading

entry	*B_2cat_2 (x equiv)*	*NMR yield (**1b**)*
1	x = 3.0	55%
2	x = 4.0	61%
3	x = 5.0	53%

3.2.5. Examination of the effect of different temperatures

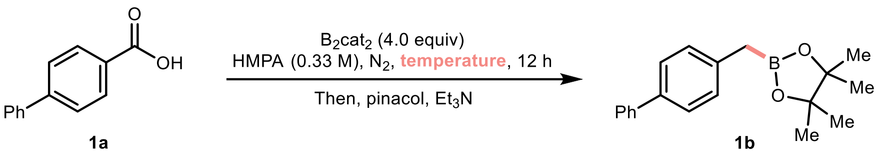

The procedure for examining the effect of different temperatures was described below.

To a flame-dried reaction tube (10 mL) equipped with a Teflon-coated magnetic stirring bar were added 4-biphenylcarboxylic acid (**1a**, 39.6 mg, 0.20 mmol, 1.0 equiv) and B_2cat_2 (190.2 mg, 0.80 mmol, 4.0 equiv). The resulting mixture was degassed and back-filled with nitrogen (N_2, high purity 4.8, >99.998%) before being transferred to the nitrogen-filled glovebox. Shortly after, 0.60 mL HMPA (0.33 M) was syringed into the reaction tube, which was capped by an aluminum seal with PTFE/silicone septum. The reaction tube was moved out of the glovebox and stirred at the designated temperature for 12 hours.

Upon the completion of the reaction, to the resulted crude was added pinacol (189.1 mg, 1.6 mmol, 8.0 equiv) and 0.70 mL Et_3N. Then, the mixture was stirred at ambient temperature for 1.0 hour. After adding the 1,3,5-trimethoxybenzene (16.8 mg, 0.10 mmol, 1.0 equiv) as internal standard, it was quenched by 3.0 mL brine and 1.0 mL distilled H_2O and supplemented with 5.0 mL EtOAc for extraction. With vigorous shaking followed by unperturbed standing for a while to allow the two layers to separate, the upper organic layer was transferred and passed through a short-packed pipette column filled with Na_2SO_4 (3.0 cm) and silica gel (0.50 cm). The above-mentioned extraction process was repeated for another four times with EtOAc (5.0*4 mL). The filtered anhydrous organic solution (~25 mL) was collected in a 100 mL round bottom flask and concentrated on a rotary evaporator. The crude was subjected to ^1H NMR and GC-MS analysis for the reaction yield, and the results were summarized in the following table.

Supplementary Table 3.2.5. Effect of different temperatures

entry	*temperature*	*NMR yield (**1b**)*

1	120 °C	65%
2	100 °C	61%
3	80 °C	43%
4	60 °C	trace
5	40 °C	0%

3.2.6. Examination of the effect of different reaction time

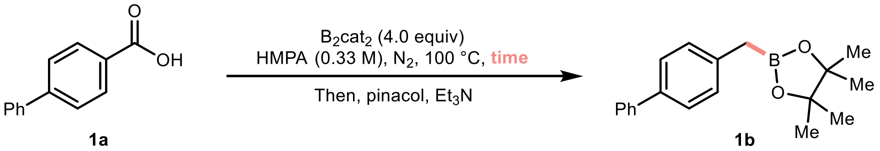

The procedure for examining the effect of different reaction time was described below.

To a flame-dried reaction tube (10 mL) equipped with a Teflon-coated magnetic stirring bar were added 4-biphenylcarboxylic acid (**1a**, 39.6 mg, 0.20 mmol, 1.0 equiv) and B_2cat_2 (190.2 mg, 0.80 mmol, 4.0 equiv). The resulting mixture was degassed and back-filled with nitrogen (N_2, high purity 4.8, >99.998%) before being transferred to the nitrogen-filled glovebox. Shortly after, 0.60 mL HMPA (0.33 M) was syringed into the reaction tube, which was capped by an aluminum seal with PTFE/silicone septum. The reaction tube was moved out of the glovebox and stirred at 100 °C for different reaction time.

Upon the completion of the reaction, to the resulted crude was added pinacol (189.1 mg, 1.6 mmol, 8.0 equiv) and 0.70 mL Et_3N. Then, the mixture was stirred at ambient temperature for 1.0 hour. After adding the 1,3,5-trimethoxybenzene (16.8 mg, 0.10 mmol, 1.0 equiv) as internal standard, it was quenched by 3.0 mL brine and 1.0 mL distilled H_2O and supplemented with 5.0 mL EtOAc for extraction. With vigorous shaking followed by unperturbed standing for a while to allow the two layers to separate, the upper organic layer was transferred and passed through a short-packed pipette column filled with Na_2SO_4 (3.0 cm) and silica gel (0.50 cm). The above-mentioned extraction process was repeated for another four times with EtOAc (5.0*4 mL). The filtered anhydrous organic solution (~25 mL) was collected in a 100 mL round bottom flask and concentrated on a rotary evaporator. The crude was subjected to ^1H NMR and GC-MS analysis for the reaction yield, and the results were summarized in the following table.

Supplementary Table 3.2.6. Effect of different reaction time

entry	*reaction time*	*NMR yield (**1b**)*
1	24 h	55%
2	12 h	61%
3	6 h	49%
4	3 h	43%

3.2.7. Examination of the effect of a catalytic amount of base additives

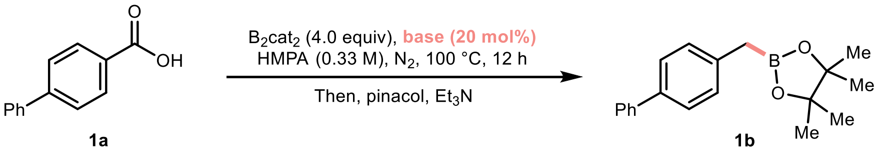

The procedure for examining the effect of different basic additives was described below.

To a flame-dried reaction tube (10 mL) equipped with a Teflon-coated magnetic stirring bar were added 4-biphenylcarboxylic acid (**1a**, 39.6 mg, 0.20 mmol, 1.0 equiv), B_2cat_2 (190.2 mg, 0.80 mmol, 4.0 equiv) and the corresponding base (0.040 mmol, 20 mol%). The resulting mixture was degassed and back-filled with nitrogen (N_2, high purity 4.8, >99.998%) before being transferred to the nitrogen-filled glovebox. Shortly after, 0.60 mL HMPA (0.33 M) was syringed into the reaction tube, which was capped by an aluminum seal with PTFE/silicone septum. The reaction tube was moved out of the glovebox and stirred at 100 °C for 12 hours.

Upon the completion of the reaction, to the resulted crude was added pinacol (189.1 mg, 1.6 mmol, 8.0 equiv) and 0.70 mL Et_3N. Then, the mixture was stirred at ambient temperature for 1.0 hour. After adding the 1,3,5-trimethoxybenzene (16.8 mg, 0.10 mmol, 1.0 equiv) as internal standard, it was quenched by 3.0 mL brine and 1.0 mL distilled H_2O and supplemented with 5.0 mL EtOAc for extraction. With vigorous shaking followed by unperturbed standing for a while to allow the two layers to separate, the upper organic layer was transferred and passed through a short-packed pipette column filled with Na_2SO_4 (3.0 cm) and silica gel (0.50 cm). The above-mentioned extraction process was repeated for another four times with EtOAc (5.0*4 mL). The filtered anhydrous organic solution (~25 mL) was collected in a 100 mL round bottom flask and concentrated on a rotary evaporator. The crude was subjected to ^1H NMR and GC-MS analysis for the reaction yield, and the results were summarized in the following table.

Supplementary Table 3.2.7. Effect of different basic additives

entry	*base (20 mol%)*	*NMR yield (**1b**)*
1	none	61%
2	NaOMe	42%
3	Na_2CO_3	39%
4	NaF	56%
5	NaF (3.0 equiv)	49%
6	Et_3N	50%
7	pyridine	44%

3.2.8. Control experiments

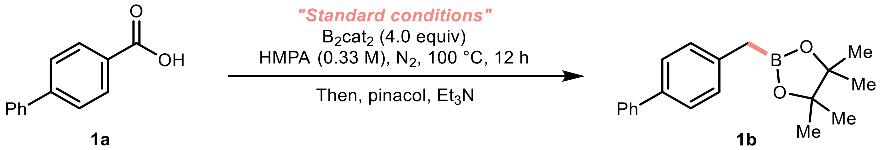

1a **1b**

The procedure for the control experiments with free carboxylic acid was described below.

To a flame-dried reaction tube (10 mL) equipped with a Teflon-coated magnetic stirring bar were added 4-biphenylcarboxylic acid (**1a**, 39.6 mg, 0.20 mmol, 1.0 equiv), B_2cat_2 (190.2 mg, 0.80 mmol, 4.0 equiv) and the corresponding control reagent/catalyst. The resulting mixture was degassed and back-filled with nitrogen (N_2, high purity 4.8, >99.998%) before being transferred to the nitrogen-filled glovebox. Shortly after, 0.60 mL HMPA (0.33 M) was syringed into the reaction tube, which was capped by an aluminum seal with PTFE/silicone septum. The reaction tube was moved out of the glovebox and stirred at 100 °C for 12 hours under nitrogen or air.

Upon the completion of the reaction, to the resulted crude was added pinacol (189.1 mg, 1.6 mmol, 8.0 equiv) and 0.70 mL Et_3N. Then, the mixture was stirred at ambient temperature for 1.0 hour. After adding the 1,3,5-trimethoxybenzene (16.8 mg, 0.10 mmol, 1.0 equiv) as internal standard, it was quenched by 3.0 mL brine and 1.0 mL distilled H_2O and supplemented with 5.0 mL EtOAc for extraction. With vigorous shaking followed by unperturbed standing for a while to allow the two layers to separate, the upper organic layer was transferred and passed through a short-packed pipette column filled with Na_2SO_4 (3.0 cm) and silica gel (0.50 cm). The above-mentioned extraction process was repeated for another four times with EtOAc (5.0*4 mL). The filtered anhydrous organic solution (~25 mL) was collected in a 100 mL round bottom flask and concentrated on a rotary evaporator. The crude was subjected to ^{1}H NMR and GC-MS analysis for the reaction yield, and the results were summarized in the following table.

Supplementary Table 3.2.8. Effect of other reaction parameters

entry	*variations from standards conditions*	*NMR yield (**1b**)*
1	none	61%
2	under air	56%
3	+ ICyCuCl (5.0 mol%)	21%
4	+ $FeBr_2$ (10 mol%)	60%
5	+ $Pd(PPh_3)_2Cl_2$ (10 mol%)	23%
6	+ $[Rh(COD)Cl]_2$ (5.0 mol%)	24%
7	+ $Ru(PPh_3)_3Cl_2$ (10 mol%)	19%
8	+ $[Ir(COD)Cl]_2$ (5.0 mol%)	13%

Note:

- *For the under-air experiment, the reaction tube was filled with compressed air before being stirred with heat.*
- *The ICyCuCl and anhydrous $FeBr_2$ catalysts were shown effective in the deoxygenative borylation of aldehydes/ketones and esters, respectively.[2, 3] The former were prepared via in-situ mixing copper (I) chloride (CuCl, 1.1 mg, 0.011 mmol, 5.6 mol%), 1,3-dicyclohexylimidazolium chloride (ICy·HCl, 2.7 mg, 0.010 mmol, 5.0 mol%) and NaOt-Bu (1.0 mg, 0.011 mmol, 5.4 mol%), which was followed by the addition of 0.30 mL HMPA and stirred for 30 minutes to pre-form the metal complex ICyCuCl. The anhydrous $FeBr_2$ and other transition metal catalysts used in the control experiments were purchased from chemical vendors, stored in the glovebox, and used without purification. Other representative transition metal catalysts (e.g., [Pd], [Rh], [Ru] and [Ir]), which have been shown effective in C-H/C-X borylation, were also examined to evaluate their potential involvement as trace residues in this deoxygenative borylation.*
- *A recent report by Su's group showed the capability of [Ni] in performing direct decarboxylative borylation of free carboxylic acids.[4] Such a divergent reaction outcome indicated the distinct reactivity of our B_2cat_2/HMPA deoxygenation system, which is complementary to Su's protocol.*
- *Neither of the tested catalysts gave improved yields than the standard conditions, indicating that the carboxylic acid deoxygenative borylation does not require transition metals.*

4. General procedure

4.1. General procedure for deoxy-monoboration of aromatic carboxylic acids and their sodium salts

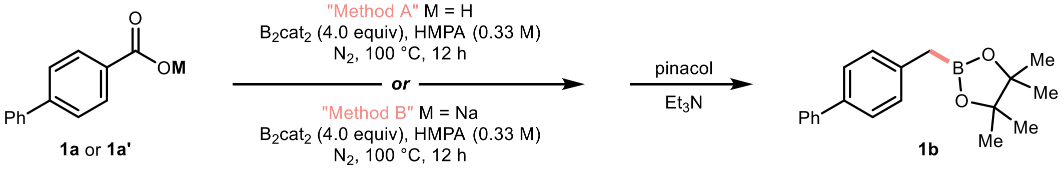

The preparation of **1b** from **1a** (M = H) or **1a'** (M = Na) is representative and applicable to all boronic acid pinacol monoester synthesis from aromatic carboxylic acids or their sodium salts in this work unless otherwise specified.

To a flame-dried reaction tube (10 mL) equipped with a Teflon-coated magnetic stirring bar were added 4-biphenylcarboxylic acid (**1a**, 39.6 mg, 0.20 mmol, 1.0 equiv) or 4-biphenylcarboxylic acid sodium salt (**1a'**, 44.0 mg, 0.20 mmol, 1.0 equiv) and B_2cat_2 (190.2 mg, 0.80 mmol, 4.0 equiv). The resulting mixture was degassed and back-filled with nitrogen (N_2, high purity 4.8, >99.998%) before being transferred to the nitrogen-filled glovebox. Shortly after, 0.60 mL HMPA (0.33 M) was syringed into the reaction tube, which was capped by an aluminum seal with PTFE/silicone septum. The reaction tube was moved out of the glovebox and stirred at 100 °C for 12 hours.

Upon the completion of the reaction, to the resulted crude was added pinacol (189.1 mg, 1.6 mmol, 8.0 equiv) and 0.70 mL Et_3N. Then, the mixture was stirred at ambient temperature for 1.0 hour, which was quenched by 3.0 mL brine and 1.0 mL distilled H_2O and supplemented with 5.0 mL EtOAc for extraction. With vigorous shaking followed by unperturbed standing for a while to allow the two layers to separate, the upper organic layer was transferred and passed through a short-packed pipette column filled with Na_2SO_4 (3.0 cm) and silica gel (0.50 cm). The above-mentioned extraction process was repeated for another four times with EtOAc (5.0*4 mL). The filtered anhydrous organic solution (~25 mL) was collected in a 100 mL round bottom flask and concentrated on a rotary evaporator. The crude was subjected to flash column chromatography on silica gel to furnish the titled compound **1b**.

Comments:

1. *Significant hydrogen evolution could occur when adding the diol for transesterification. In large-scale synthesis, sufficient chilling and slow diol addition were needed for safety concerns.*
2. *During the substrate scope exploration, it was found that the boronic acid pinacol esters mostly showed weak ultra-violet (UV) responses and common stainings, for instance, phosphomolybdic acid with heat activation, generally do not assist the visualization on TLC. Besides, the extraction with EtOAc inevitably rendered some HMPA irremovable from the reaction crude. These issues complicated the isolation of desired products and affected their isolated yield and purity. With these problems, flash column chromatography performed by Isolera™ Prime advanced automatic flash purification system was suggested. The system was established based on a double-beam collection mode with both 220 nm and 254 nm, in which the "collect all" mode was turned off, and the threshold was defaulted to be 15 mAU. In light of the relatively high solvent cut-off of EtOAc, Et_2O was preferred to combine with hexane as eluents.*
3. *It was very important to know that this type of boronic acid esters might decompose on silica gel (generally true when purifying the alkylboronic esters); therefore, the eluent flow rate was well-*

controlled, and the overall chromatographic process was completed within 10 minutes. However, the column should not be too short since the highly polar HMPA residue in the crude could easily elute the product.

4. *In the case of very challenging product isolation, DMA could replace HMPA as the solvent, which simplified the purification but gave similar productivity. Otherwise, the residue obtained after solvent removal could be dissolved in 5.0 mL Et_2O, which was washed by 5.0 mL distilled water three times to remove most of the HMPA.*
5. *To be noticed, some ketone side product was occasionally observed during the substrate scope exploration, especially when using sodium salts or electronically neutral benzoic acids as the starting materials. The ketone side product could have very close polarity to the desired benzylboronate product and cause significant difficulty in the purification. In those cases, transesterification with pinanediol could help since the corresponding boronate have varied polarity.*
6. *It was observed that B_2cat_2 supplied by Combi Blocks contained ~5% catechol impurities, which was found inconsequential to the reaction efficiency. This chemical was stored in a cold room (4 ℃). Before using it, it was purified according to Section 3.1.2. When the purified product was contaminated by catechol, basic treatment by washing the reaction mixture before the column chromatography with 5.0 mL Na_2CO_3 aqueous solution (0.50 M) three times could help remove most of the catechol contaminant.*
7. *The water content of pinacol could affect transesterification's efficiency; therefore, azeotroping the commercial pinacol with toluene, drying it under the high vacuum and storing it under argon atmosphere was suggested.[5]*

4.2. General procedure for deoxy-triboration of aliphatic carboxylic acid and their sodium salts

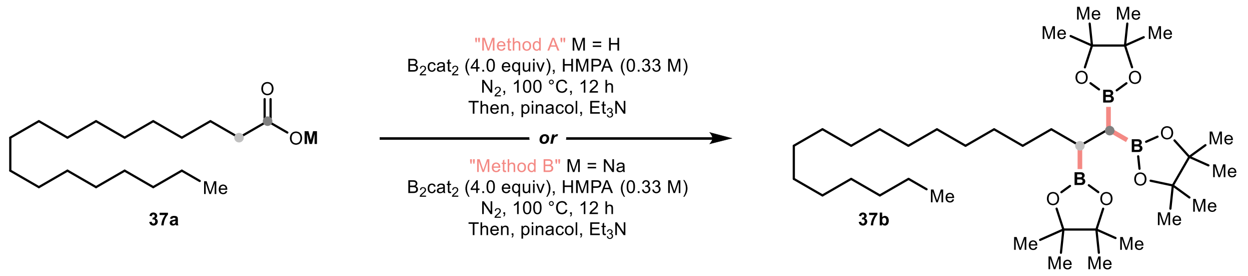

The preparation of **37b** from **37a** or **37a'** is representative and applicable to tris(boronate) synthesis from aliphatic carboxylic acids or their sodium salts in this work unless otherwise specified.

To a flame-dried reaction tube (10 mL) equipped with a Teflon-coated magnetic stirring bar were added octadecanoic acid (**37a**, 56.8 mg, 0.20 mmol, 1.0 equiv) or sodium octadecenoate (**37a'**, 61.2 mg, 0.20 mmol, 1.0 equiv) and B_2cat_2 (190.2 mg, 0.80 mmol, 4.0 equiv). The resulting mixture was degassed and back-filled with nitrogen (N_2, high purity 4.8, >99.998%) before being transferred to the nitrogen-filled glovebox. Shortly after, 0.60 mL HMPA (0.33 M) was syringed into the reaction tube, which was capped by an aluminum seal with PTFE/silicone septum. The reaction tube was moved out of the glovebox and stirred at 100 ℃ for 12 hours.

Upon the completion of the reaction, to the resulted crude was added pinacol (189.1 mg, 1.6 mmol, 8.0 equiv) and 0.70 mL Et_3N. Then, the mixture was stirred at ambient temperature for 1.0 hour, which was quenched by 3.0 mL brine and 1.0 mL distilled H_2O and supplemented with 5.0 mL EtOAc for extraction.

With vigorous shaking followed by unperturbed standing for a while to allow the two layers to separate, the upper organic layer was transferred and passed through a short-packed pipette column filled with Na_2SO_4 (3.0 cm) and silica gel (0.50 cm). The above-mentioned extraction process was repeated for another four times with EtOAc (5.0*4 mL). The filtered anhydrous organic solution (~25 mL) was collected in a 100 mL round bottom flask and concentrated on a rotary evaporator. The crude was subjected to flash column chromatography on silica gel to furnish the titled compound **37b**.

Comments:

1. *Significant hydrogen evolution would occur when adding the diol for transesterification. In large-scale synthesis, sufficient chilling and slow diol addition were needed for safety concerns.*
2. *The polarity of desired tris(boronate) was found very close to the unreacted B_2pin_2 (transformed from B_2cat_2 during the transesterification workup), and both of them are difficult to be visualized by common methods. Due to the high response factors of both tris(boronate) and B_2pin_2 in GC-MS, it could be used as a convenient method to check the purity of the isolated product.*
3. *During the substrate scope exploration, it was found that the tris(boronates) mostly showed no ultra-violet (UV) responses, especially for the ones without aromatic moiety. Common stainings, for instance, phosphomolybdic acid with heat activation, generally do not assist the visualization on TLC. Besides, the extraction with EtOAc inevitably rendered some HMPA irremovable from the reaction crude. These issues complicated the isolation of desired products and affected their isolated yield and purity. With these problems, flash column chromatography performed by Isolera™ Prime advanced automatic flash purification system was suggested. In this system, the "collect all" mode was turned on. In light of the relatively high solvent cut-off of EtOAc, Et_2O was preferred to combine with hexane as eluents.*
4. *It was very important to know that this type of multiborylated compounds might decompose on silica gel (generally true when purifying the alkylboronic esters); therefore, the eluent flow rate was well-controlled, and the overall chromatographic process was completed within 15 minutes. However, the column should not be too short since the highly polar HMPA residue in the crude could easily elute the product.*
5. *In the case of very challenging product isolation, DMA could replace HMPA as the solvent, which simplified the purification but gave similar productivity. Otherwise, the residue obtained after solvent removal could be dissolved in 5.0 mL Et_2O, which was washed by 5.0 mL distilled water three times to remove most of the HMPA.*
6. *When the purified product was contaminated by catechol, basic treatment by washing the reaction mixture before the column chromatography with 5.0 mL Na_2CO_3 aqueous solution (0.50 M) three times could help remove most of the catechol contaminant.*
7. *The water content of pinacol could affect transesterification's efficiency; therefore, azeotroping the commercial pinacol with toluene, drying it under the high vacuum and storing it under argon atmosphere was suggested.*[5]

4.3. General procedure for the synthetic application

4.3.1. Selective protodeboronation

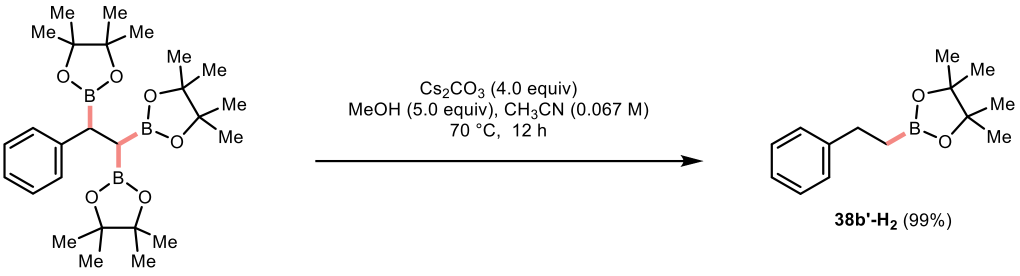

38b → **38b'-H₂** (99%)

The preparation of **38b'-H$_2$** from **38b** via selective protodeboronation was based on a slight modification of Song's protocol.[6]

To a flame-dried reaction tube (10 mL) equipped with a Teflon-coated magnetic stirring bar were added **38b** (96.8 mg, 0.20 mmol, 1.0 equiv) and Cs_2CO_3 (260 mg, 0.80 mmol, 4.0 equiv). Shortly after, 3.0 mL CH_3CN (0.33 M) and MeOH (32 mg, 1.0 mmol, 5.0 equiv) were syringed into the reaction tube. The resulting mixture was degassed by three freeze-pump-thaw cycles and back-filled with nitrogen (N_2, high purity 4.8, >99.998%), which was capped an aluminium seal with PTFE/silicone septum. The reaction was stirred at 70 °C for 12 hours.

Upon completing the reaction, the crude was diluted by EtOAc, filtered through a silica gel plug, and concentrated on a rotary evaporator. The crude was subjected to flash column chromatography on silica gel to furnish the titled compound **38b'-H$_2$**.

4.3.2. Sequential carboxylation and deoxygenative borylation

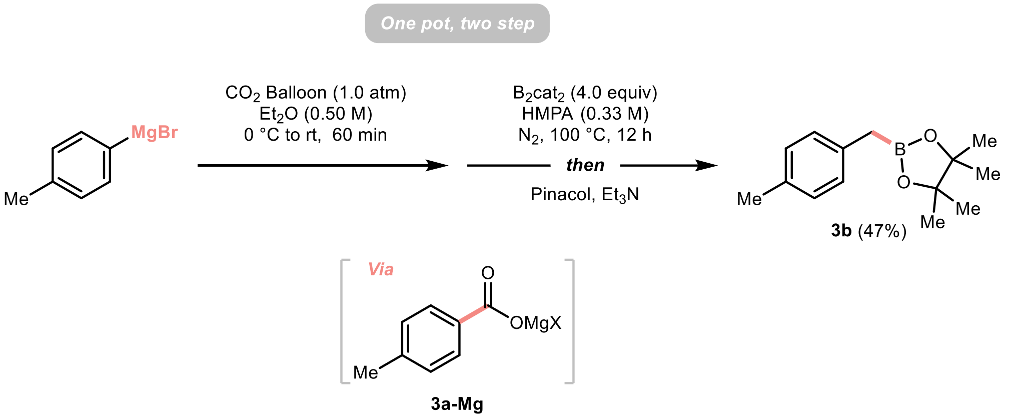

3b (47%)

3a-Mg

The preparation of **3b** from Grignard reagent via sequential carboxylation and deoxygenative borylation was described below.

At 0 °C (ice bath), to a flame-dried reaction tube (10 mL) equipped with a Teflon-coated magnetic stirring bar were added 0.40 mL *p*-tolylmagnesium bromide ethereal solution (0.20 mmol, 1.0 equiv, 0.50 M in Et_2O) under argon atmosphere. The reaction tube was covered by a rubber septum and charged with carbon dioxide (CO_2, high purity 3.0, >99.9%) by a balloon. The ice bath was removed to allow the temperature to gradually resume to room temperature. After 60 minutes, the solvent was removed under vacuum, and a white foamy solid (**3a-Mg**) was obtained, which was not purified and directly subjected to the next step.

To the dried reaction crude was added B_2cat_2 (190.2 mg, 0.80 mmol, 4.0 equiv). The resulting mixture was degassed and back-filled with nitrogen (N_2, high purity 4.8, >99.998%) before being transferred to the nitrogen-filled glovebox. Shortly after, 0.60 mL HMPA (0.33 M) was syringed into the reaction tube, which was capped by an aluminum seal with PTFE/silicone septum. The reaction tube was moved out of the glovebox and stirred at 100 °C for 12 hours.

Upon the completion of the reaction, to the resulted crude was added pinacol (189.1 mg, 1.6 mmol, 8.0 equiv) and 0.70 mL Et_3N. Then, the mixture was stirred at ambient temperature for 1.0 hour. After adding the 1,3,5-trimethoxybenzene (16.8 mg, 0.10 mmol, 1.0 equiv) as internal standard, it was quenched by 3.0 mL brine and 1.0 mL distilled H_2O and supplemented with 5.0 mL EtOAc for extraction. With vigorous shaking followed by unperturbed standing for a while to allow the two layers to separate, the upper organic layer was transferred and passed through a short-packed pipette column filled with Na_2SO_4 (3.0 cm) and silica gel (0.50 cm). The above-mentioned extraction process was repeated for another four times with EtOAc (5.0*4 mL). The filtered anhydrous organic solution (~25 mL) was collected in a 100 mL round bottom flask and concentrated on a rotary evaporator. The crude was subjected to ^{1}H NMR and GC-MS analysis for the reaction yield. These analyses showed a 47% yield of the desired benzylboronate.

4.3.3. Deoxygenative borylation of mixed aldehydes and carboxylic acids

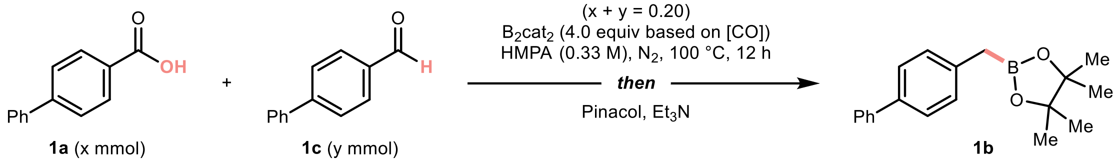

The preparation of **1b** from mixed carboxylic acid **1a** and aldehyde **1c** was described below.

To a flame-dried reaction tube (10 mL) equipped with a Teflon-coated magnetic stirring bar were added 4-biphenylcarboxylic acid (**1a**, x mmol) and 4-biphenylcarboxaldehyde (**1c**, y mmol) and B_2cat_2 (190.2 mg, 0.80 mmol, 4.0 equiv based on the overall amount of carbonyl-containing compounds). The resulting mixture was degassed and back-filled with nitrogen (N_2 high purity, >99.998%) before being transferred to the nitrogen-filled glovebox. Shortly after, 0.60 mL HMPA (0.33 M) was syringed into the reaction tube, which was capped by an aluminum seal with PTFE/silicone septum. The reaction tube was moved out of the glovebox and stirred at 100 °C for 12 hours.

Upon the completion of the reaction, to the resulted crude was added pinacol (189.1 mg, 1.6 mmol, 8.0 equiv) and 0.70 mL Et_3N. Then, the mixture was stirred at ambient temperature for 1.0 hour. After adding the 1,3,5-trimethoxybenzene (16.8 mg, 0.10 mmol, 1.0 equiv) as internal standard, it was quenched by 3.0 mL brine and 1.0 mL distilled H_2O and supplemented with 5.0 mL EtOAc for extraction. With vigorous shaking followed by unperturbed standing for a while to allow the two layers to separate, the upper organic layer was transferred and passed through a short-packed pipette column filled with Na_2SO_4 (3.0 cm) and silica gel (0.50 cm). The above-mentioned extraction process was repeated for another four times with EtOAc (5.0*4 mL). The filtered anhydrous organic solution (~25 mL) was collected in a 100 mL round bottom flask and concentrated on a rotary evaporator. The crude was subjected to ^{1}H NMR and GC-MS analysis for the reaction yield, and the results were summarized in the following table.

Supplementary Table 4.3.3. Deoxygenative borylation of mixed carboxylic acid and aldehyde

entry	$ArCO_2H$ (x) : ArCHO (y)	NMR yield (**1b**)
1	x : y = 3 : 1	62%
2	x : y = 1 : 1	77%
3	x : y = 1 : 3	83%

4.3.4. Deoxygenative borylation of alcohol oxidation product

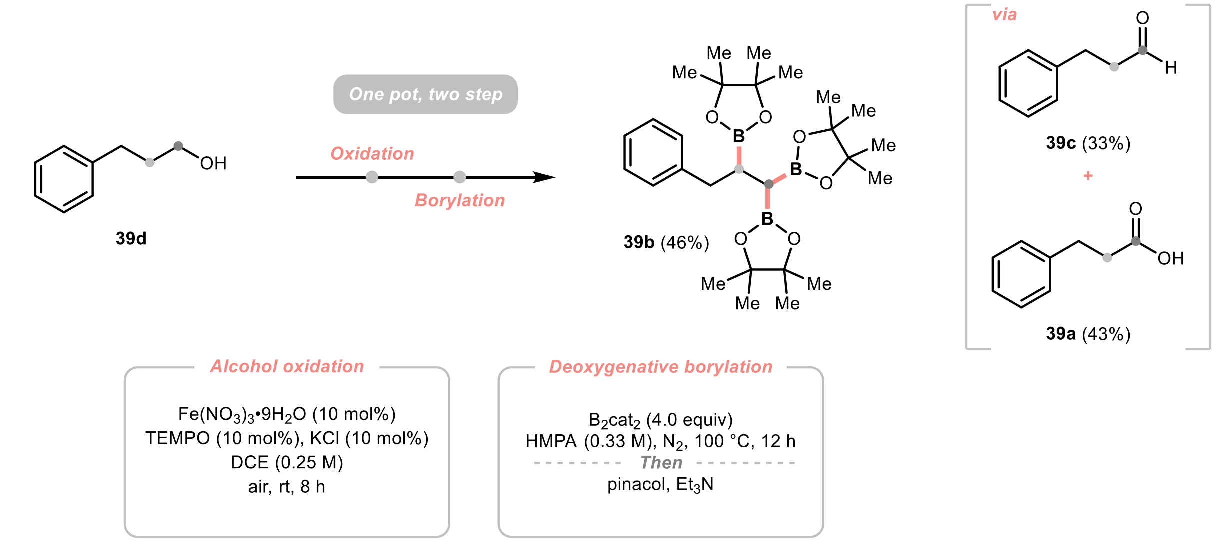

The preparation of **39b** from a one-pot, two-step sequence starting from **39d** was described below.

The alcohol oxidation conditions were based on a slight modification of Ma's protocol.[7] To a flame-dried reaction tube (10 mL) equipped with a Teflon-coated magnetic stirring bar were added iron(III) nitrate nonahydrate ($Fe(NO_3)\cdot 9H_2O$, 4.2 mg, 0.020 mmol, 10 mol%), (2,2,6,6-tetramethylpiperidin-1-yl)oxyl (TEMPO, 3.1 mg, 0.020 mmol, 10 mol%) and KCl (1.5 mg, 0.020 mmol, 10 mol%). Shortly after, 0.80 mL DCE (0.25 M) was syringed into the reaction tube, followed by 3-phenyl-1-propanol (**39d**, 27.2 mg, 0.020 mmol, 1.0 equiv). The reaction was stirred under air at room temperature for 8 hours. Two reactions were conducted in parallel at the same time.

After that, one of the reactions was intercepted for determining the reaction conversion and crude composition by being quenched with 3.0 mL brine, 1.0 mL distilled H_2O and supplemented with 5.0 mL EtOAc for extraction. With vigorous shaking followed by unperturbed standing for a while to allow the two layers to separate, the upper organic layer was transferred and passed through a short-packed pipette column filled with Na_2SO_4 (3.0 cm) and silica gel (0.50 cm). The above-mentioned extraction process was repeated for another four times with EtOAc (5.0*4 mL). The filtered anhydrous organic solution (~25 mL) was collected in a 100 mL round bottom flask and concentrated on a rotary evaporator. After the addition of dibromomethane (34.8 mg, 0.20 mmol, 1.0 equiv) as the internal standard, the crude was subjected to ^1H NMR and GC-MS analysis for the reaction yield. *^1H NMR showed a 76% conversion after the reaction, and a 33% yield of the corresponding aldehyde (**39c**) and 43% yield of the carboxylic acid (**39a**) were obtained.*

With this information, the solvent of the parallel reaction was removed under the vacuum. Then, to the dried reaction crude was added B_2cat_2 (190.2 mg, 0.80 mmol, 4.0 equiv). The resulting mixture was degassed and back-filled with nitrogen (N_2, high purity 4.8, >99.998%) before being transferred to the

nitrogen-filled glovebox. Shortly after, 0.60 mL HMPA (0.33 M) was syringed into the reaction tube, which was capped by an aluminum seal with PTFE/silicone septum. The reaction tube was moved out of the glovebox and stirred at 100 °C for 12 hours.

Upon the completion of the reaction, to the resulted crude was added pinacol (189.1 mg, 1.6 mmol, 8.0 equiv) and 0.70 mL Et_3N. Then, the mixture was stirred at ambient temperature for 1.0 hour. After adding the 1,3,5-trimethoxybenzene (16.8 mg, 0.10 mmol, 1.0 equiv) as internal standard, it was quenched by 3.0 mL brine and 1.0 mL distilled H_2O and supplemented with 5.0 mL EtOAc for extraction. With vigorous shaking followed by unperturbed standing for a while to allow the two layers to separate, the upper organic layer was transferred and passed through a short-packed pipette column filled with Na_2SO_4 (3.0 cm) and silica gel (0.50 cm). The above-mentioned extraction process was repeated for another four times with EtOAc (5.0*4 mL). The filtered anhydrous organic solution (~25 mL) was collected in a 100 mL round bottom flask and concentrated on a rotary evaporator. The crude was subjected to ^{1}H NMR and GC-MS analysis for the reaction yield. These analyses showed a 46% yield of the desired tris(boronate) (**39b**) and 61% yield based on the combined yield of alcohol oxidation product.

4.3.5. Deoxygenative borylation of methylarene oxidation product

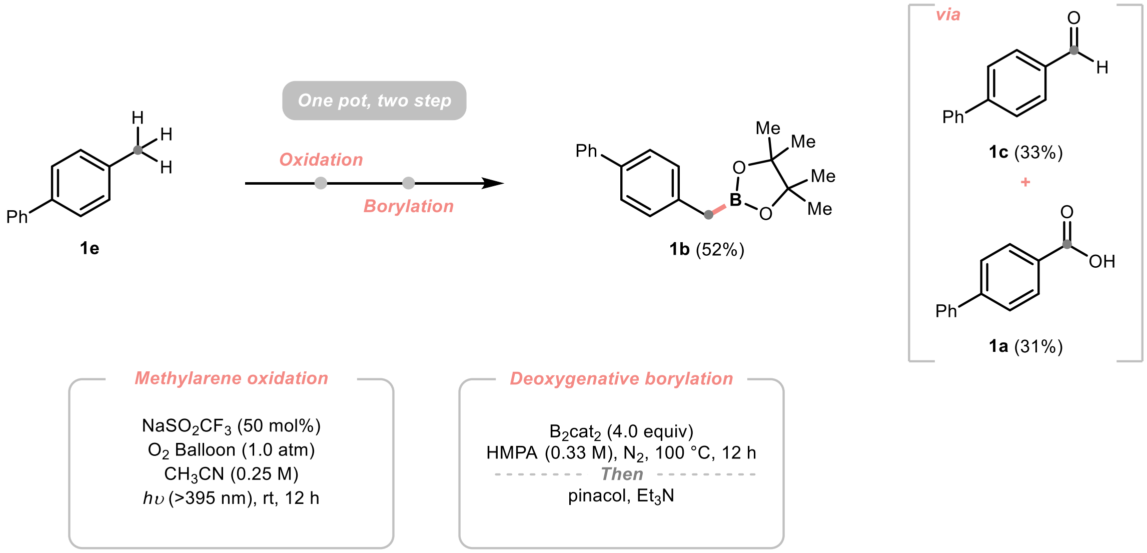

The preparation of **1b** from a one-pot, two-step sequence starting from **1e** was described below.

The alcohol oxidation conditions were based on a slight modification of Fu's protocol.[8] To a flame-dried reaction tube (10 mL) equipped with a Teflon-coated magnetic stirring bar were added 4-phenyltoluene (33.6 mg, 0.20 mmol, 1.0 equiv) and sodium triflinate ($NaSO_2CF_3$, 15.6 mg, 0.10 mmol, 50 mol%). Shortly after, 1.0 mL CH_3CN (0.20 M) was syringed into the reaction tube. The reaction tube was covered by a rubber septum, and the resulting mixture was degassed and back-filled with oxygen (O_2, extra dry, high purity 2.6, >99.6%). After installing an oxygen balloon (1.0 atm), the reaction tube was irradiated by a xenon lamp equipped with a 395 nm long-pass optical filter at room temperature for 12 hours. The reaction was stirred at room temperature for 8 hours. Two reactions were conducted in parallel at the same time.

Upon completion, one of the reactions was intercepted for determining the reaction conversion and crude composition. After removing the solvent under vacuum and adding dibromomethane (34.8 mg, 0.20

mmol, 1.0 equiv) as the internal standard, the crude was subjected to ^1H NMR and GC-MS analysis for the reaction yield. *^1H NMR showed a 71% conversion after the reaction, and a 33% yield of the corresponding aldehyde (**1c**) and a 31% yield of the carboxylic acid (**1a**) were obtained.*

With this information, the solvent of the parallel reaction was removed under the vacuum. Then, to the dried reaction crude was added B_2cat_2 (190.2 mg, 0.80 mmol, 4.0 equiv). The resulting mixture was degassed and back-filled with nitrogen (N_2, high purity 4.8, >99.998%) before being transferred to the nitrogen-filled glovebox. Shortly after, 0.60 mL HMPA (0.33 M) was syringed into the reaction tube, which was capped by an aluminum seal with PTFE/silicone septum. The reaction tube was moved out of the glovebox and stirred at 100 °C for 12 hours.

Upon the completion of the reaction, to the resulted crude was added pinacol (189.1 mg, 1.6 mmol, 8.0 equiv) and 0.70 mL Et_3N. Then, the mixture was stirred at ambient temperature for 1.0 hour. After adding the 1,3,5-trimethoxybenzene (16.8 mg, 0.10 mmol, 1.0 equiv) as internal standard, it was quenched by 3.0 mL brine and 1.0 mL distilled H_2O and supplemented with 5.0 mL EtOAc for extraction. With vigorous shaking followed by unperturbed standing for a while to allow the two layers to separate, the upper organic layer was transferred and passed through a short-packed pipette column filled with Na_2SO_4 (3.0 cm) and silica gel (0.50 cm). The above-mentioned extraction process was repeated for another four times with EtOAc (5.0*4 mL). The filtered anhydrous organic solution (~25 mL) was collected in a 100 mL round bottom flask and concentrated on a rotary evaporator. The crude was subjected to ^1H NMR and GC-MS analysis for the reaction yield. These analyses showed a 52% yield of the desired benzylboronate (**1b**) and a 72% yield based on the combined yield of methylarene oxidation product.

4.3.6. General procedure for R-Bpin to R-BF$_3$K conversion

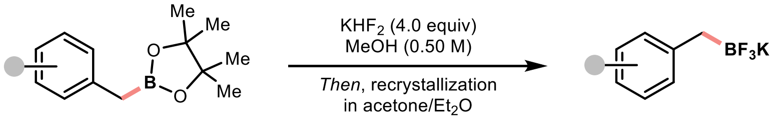

The procedure of converting the R-Bpin into R-BF$_3$K was based on a modified protocol reported by Shi[9] and Hooper & Hutton.[10]

To a stirring solution of R-Bpin in methanol (0.50 M) was added the saturated aqueous solution of KHF_2 (4.5 M, 4.0 equiv) dropwise via syringe. The mixture was kept stirred at room temperature overnight. After the reaction, the solvent was removed under vacuum, and the residue was dried to get the solidified residue. The dried solids were triturated with hot acetone and filtered to remove the excessive inorganic salts. The resulting filtrate was concentrated to a minimal volume, after which Et_2O was added to precipitate corresponding products.

4.3.7. General procedure for Suzuki-Miyaura coupling between R-BF$_3$K and Ar-Br

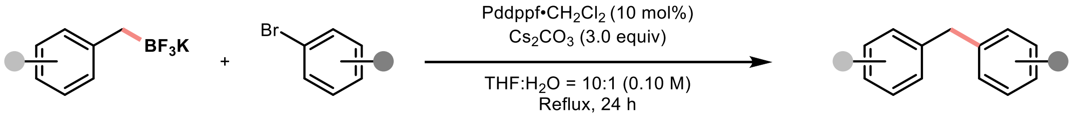

The procedure of Suzuki-Miyaura coupling between R-BK$_3$K and Ar-Br was described below.

To the mixed solution of THF and H_2O (10:1, 0.10 M) with the R-BF_3K (0.24 mmol, 1.2 equiv) and Ar-Br (0.20 mmol, 1.0 equiv) in a flame-dried reaction tube (10 mL) equipped with a Teflon-coated magnetic stirring bar was added [1,1'-bis(diphenylphosphino)ferrocene] dichloropalladium, dichloromethane complex (1:1) (Pddppf·CH_2Cl_2, 16.3 mg, 0.020 mmol, 10 mol%) and Cs_2CO_3 (195.5 mg, 0.60 mmol, 3.0 equiv). The mixture was heated to reflux and stirred at this temperature for 24 hours. After that, the volatiles were removed under vacuum, and the residue was treated with 5.0 mL H_2O. The resultant aqueous fraction was supplemented with 5.0 mL EtOAc for extraction. With vigorous shaking followed by unperturbed standing for a while to allow the two layers to separate, the upper organic layer was transferred and passed through a short-packed pipette column filled with Na_2SO_4 (3.0 cm) and silica gel (0.50 cm). The above-mentioned extraction process was repeated for another two times with EtOAc (5*2 mL). The filtered anhydrous organic solution (~15 mL) was collected in a 100 mL round bottom flask and concentrated on a rotary evaporator. The crude was subjected to ^{1}H NMR and GC-MS analysis for the reaction analysis. The desired product could be obtained by flash column chromatography on silica gel.

4.3.8. General procedure for Suzuki-Miyaura coupling between R-Bpin and Ar-Br

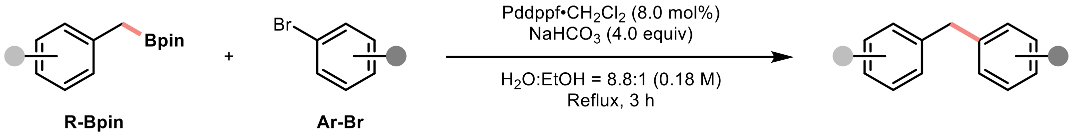

The procedure of Suzuki-Miyaura coupling between R-Bpin and Ar-Br was described below.

To a flame-dried reaction tube (10 mL) equipped with a Teflon-coated magnetic stirring bar was added Ar-Br (0.20 mmol, 1.0 equiv), R-Bpin (0.30 mmol, 1.5 equiv), [1,1'-bis(diphenylphosphino)ferrocene] dichloropalladium, dichloromethane complex (1:1) (Pddppf·CH_2Cl_2, 6.5 mg, 0.016 mmol, 8.0 mol%) and $NaHCO_3$ (33.6 mg, 0.80 mmol, 4.0 equiv). After adding a mixed solvent H_2O/EtOH (8.8:1, 0.18 M), the mixture was heated to reflux and stirred at this temperature for 3.0 hours.

After that, the hot solution was filtered over Celite and neutralized by HCl (1.0 M). Extra H_2O was supplemented to make the total volume of the aqueous layer 5.0 mL. The resultant aqueous fraction was supplemented with 5.0 mL EtOAc for extraction. With vigorous shaking followed by unperturbed standing for a while to allow the two layers to separate, the upper organic layer was transferred and passed through a short-packed pipette column filled with Na_2SO_4 (3.0 cm) and silica gel (0.50 cm). The above-mentioned extraction process was repeated for another two times with EtOAc (5*2 mL). The filtered anhydrous organic solution (~15 mL) was collected in a 100 mL round bottom flask and concentrated on a rotary evaporator. The crude was subjected to ^{1}H NMR and GC-MS analysis for the reaction analysis. The desired product could be obtained by flash column chromatography on silica gel.

4.5. Stability of benzylboronates

Referring to the storage conditions recommended by Combi Blocks, for all the benzylboronic pinacol esters sold by this vendor, it mentioned, "Store under -20 ℃ or -40 ℃ if to be stored for more than 3 months. Keep the container tightly closed in a dry and well-ventilated place. Containers which are opened must be carefully resealed and kept upright to prevent leakage" (4-Methylbenzylboronic acid pinacol ester as a representative example, please see https://www.combi-blocks.com/cgi-bin/find.cgi?PN-8008).

To provide more information about handling and storing this type of compounds, we examined their sensitivity towards light, air and moisture using benzylboronic acid pinacol ester. The results were shown below.

compound	***storage conditions at room temperature***	***recovery of 2b***	***other comments***
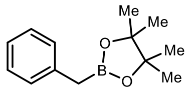 **2b** (0.20 mmol)	under nitrogen<br>with ambient light<br>30 days	91%	2% PhCHO<br>1% $PhCH_2OH$<br>0% $PhCH_3$
	under air<br>with ambient light<br>30 days	88%	trace PhCHO<br>1% $PhCH_2OH$<br>0% $PhCH_3$
	under nitrogen<br>in the dark<br>30 days	96%	trace PhCHO<br>1% $PhCH_2OH$<br>0% $PhCH_3$
	under air<br>in the dark<br>30 days	80%	trace PhCHO<br>1% $PhCH_2OH$<br>0% $PhCH_3$
	in 0.60 mL water<br>under air<br>with ambient light<br>12 h	95%	trace PhCHO<br>3% $PhCH_2OH$<br>0% $PhCH_3$

In all these stability test experiments, a high recovery of **2b** was obtained, and an insignificant amount of decomposition products, including oxidation and hydrolysis, was observed; therefore, at least in the timeframe from half a day to a month, we could conclude that **2b** was not susceptible under ambient light and atmosphere (O_2, H_2O, etc.).

To be noticed, we observed a relatively lower recovery rate when the samples were exposed to the air, which might attribute to the evaporation over time. Besides, due to the limited time, other functionalised benzylboronic acid pinacol esters were not tested, and the stability of **2b** should only serve as a standard for a part of the members in the boronate family.

Therefore, following the procedure of Combi Blocks to properly store these kinds of compounds is still highly advised for long-term storage.

4.5. Limitation of current work

4.5.1. Heteroaromatic substrates

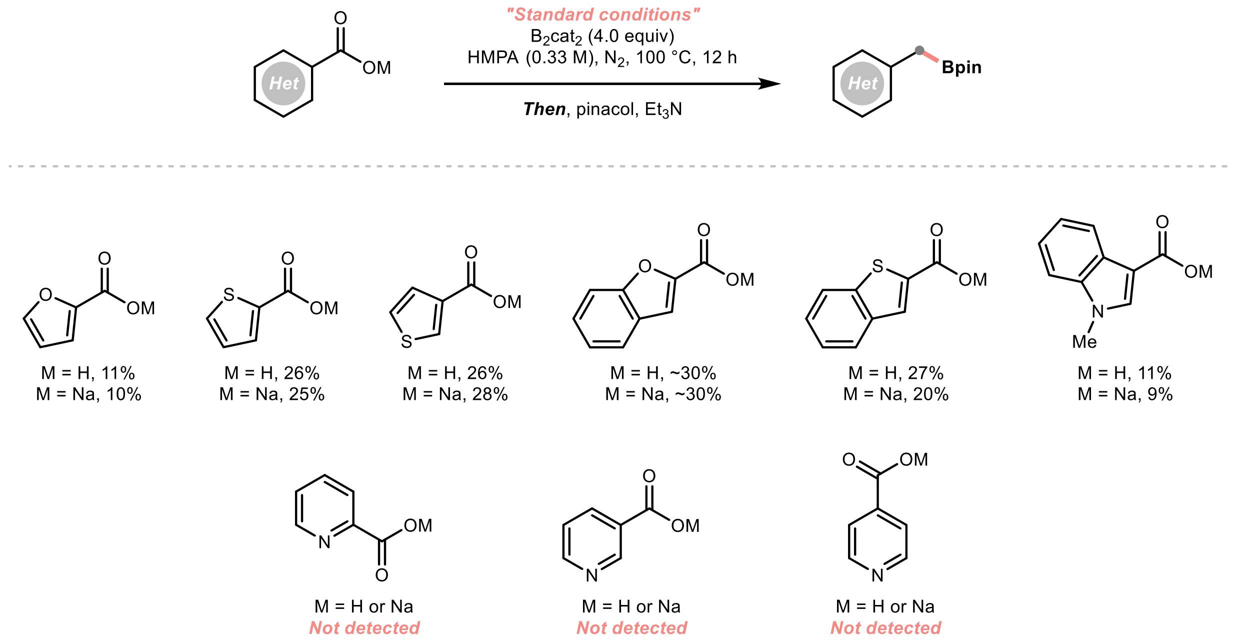

In general, the carboxylic/carboxylate groups on heteroaromatic rings showed lower reactivity compared to benzoic acid and its derivatives. This is possibly caused by the intrinsic instability of heteroaromatic rings, which could undergo hydrolytic ring opening or reductive dearomatisation under our conditions (B_2cat_2 could behave as strong Lewis acid and reductant). For those with five-membered heterocyclic moieties, their electron-rich carboxyl/carboxylate groups were more reluctant toward reduction.

To be noticed, all three isomers of the pyridinecarboxylic acids did not give the corresponding pyridylic boronates under our optimal conditions, whether in the free acid or sodium salt forms. This was in accordance with our results in testing the effect of basic additives, in which pyridine showed an inhibitory effect even in a catalytic amount.

4.5.2. Amino acids

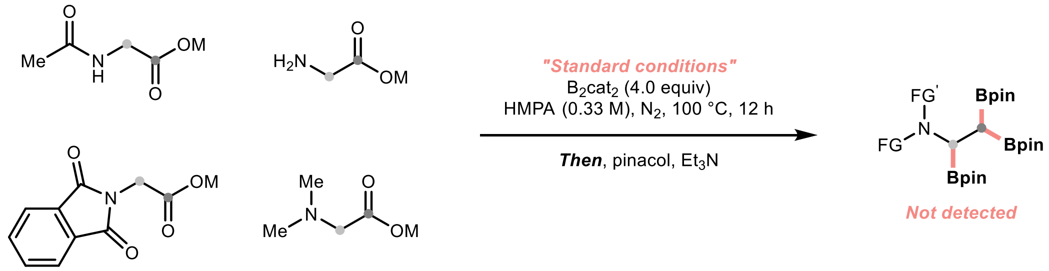

Although the amino group should, in principle, be compatible with our borylation conditions (see the example of 4-*N*,*N*-dimethylaminobenzoic acid), the desired boron products from glycines with or with various protecting groups were not observed in NMR and GC-MS analysis. We suspected that the basic and coordinating amino group adjacent to the carboxylic group would interfere with the complexation between the B_2cat_2 and the latter, inhibiting the desirable reactivities and remaining as one of the limitations of our methods.

4.5.3. Using HMPA as the solvent

In light of the detrimental effect of HMPA, we made several attempts to avoid its usage, including reducing its loading and testing other solvents. More than 25 common organic solvents in different concentrations and their combination in various ratios were examined in the deoxygenative borylation of 4-biphenylcarboxylic acid and its sodium salt.

Although most of our effort in minimising the involvement of HMPA or identifying a benign substituent of it was unsuccessful, DMA, which was commonly used as a chaperone with B_2X_4 in reductive borylation chemistry,[9, 11-13] and was often seen in other types of reductive transformations, could still give a reasonable yield of **1b** (54%).

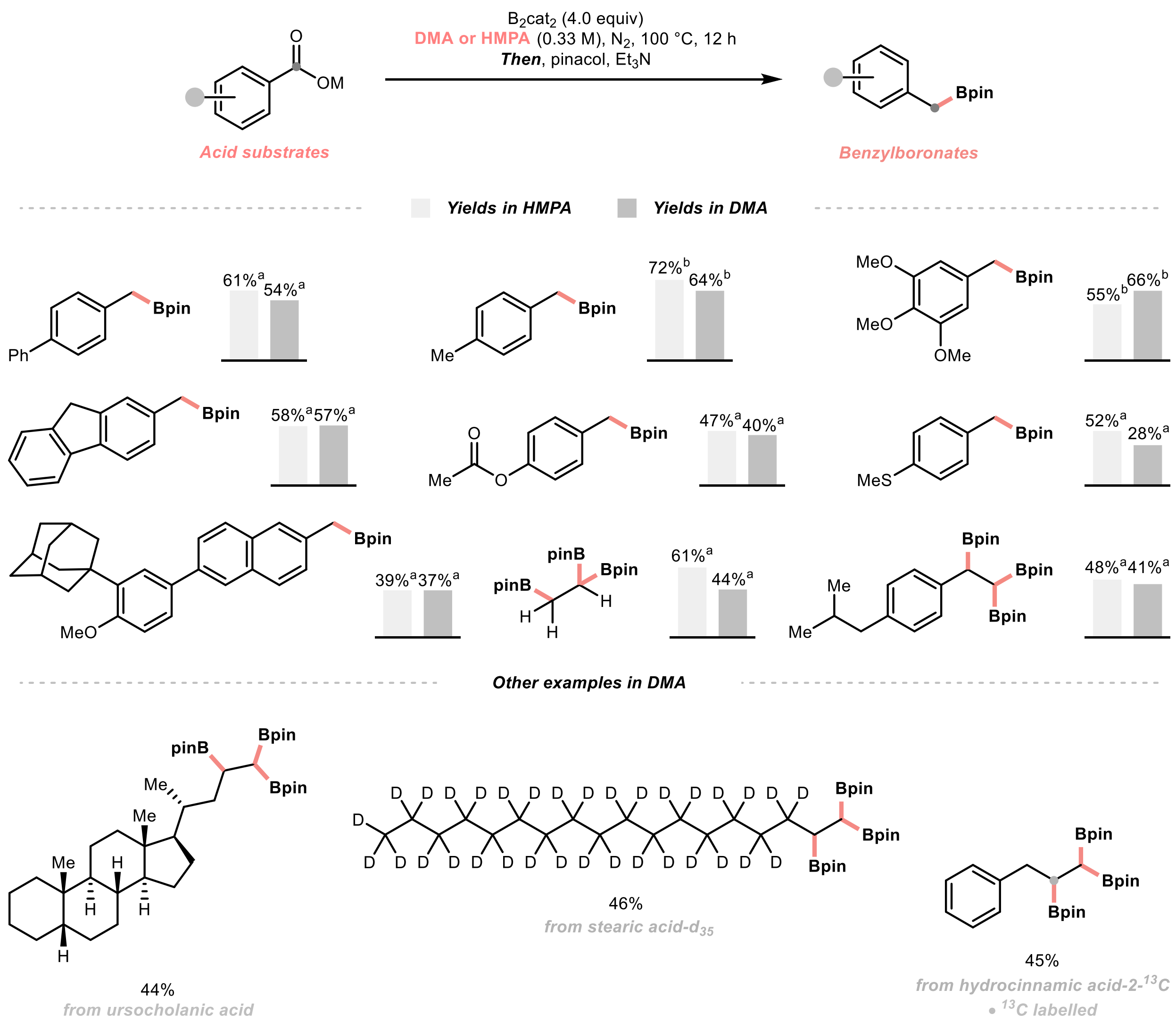

A quick spot check of some representative substrates showed that reactions in DMA could lead to comparable productivities to those in HMPA. Other examples involving complex carboxylic acid and some isotope-labelled substrates also showed promising results when DMA was employed as a solvent

During the substate scope exploration with DMA, 1,1,2-trisborylated ethane **35b** was occasionally observed, whose identity was confirmed by both NMR and GC-MS. The presence of such a side product

indicated the background reaction between B_2cat_2 and DMA solvent, which might account for the generally lower yields of reactions conducted in DMA. Armed with this information, a more robust amide solvent, *N*,*N*'-diethylacetamide (DEA), was evaluated; however, it yielded less desired product, possibly because it is too hindered to complex with B_2cat_2.

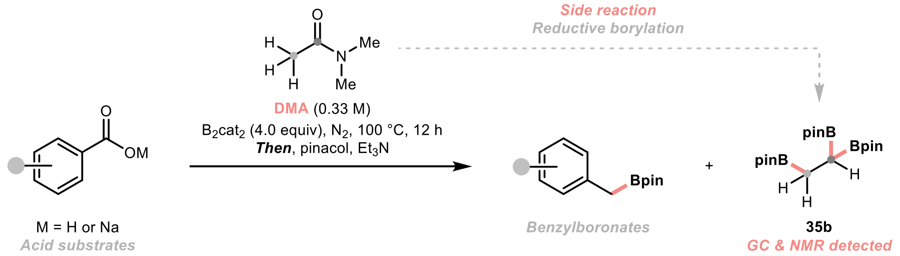

Taken together, if the harmful effect of HMPA was a major concern when using our acid deoxygenative borylation protocol, especially for large-scale synthesis, we showed that DMA served as a more user-friendly yet analogously efficient surrogate for the HMPA in our deoxygenative borylation chemistry. Besides, exploring a more robust coordinating solvent, which was more resistant toward hydrolysis and reduction, would be a promising way to further improve the current protocol.

5. Mechanistic study

5.1. Intermediate trapping experiment

5.1.1. Examination of radical intermediacy

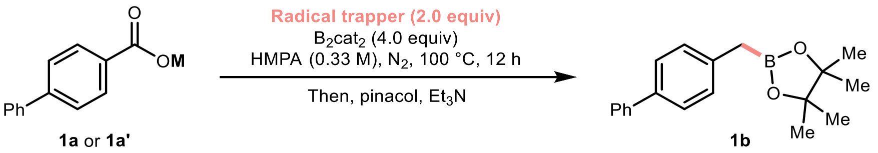

The procedure for examining radical intermediacy was described below.

To a flame-dried reaction tube (10 mL) equipped with a Teflon-coated magnetic stirring bar were added 4-biphenylcarboxylic acid (**1a**, 39.6 mg, 0.20 mmol, 1.0 equiv) or 4-biphenylcarboxylic acid sodium salt (**1a'**, 44.0 mg, 0.20 mmol, 1.0 equiv), B_2cat_2 (190.2 mg, 0.80 mmol, 4.0 equiv) and the corresponding radical trapping reagent, butylated hydroxytoluene (BHT, 44.0 mg, 0.40 mmol, 2.0 equiv) or 1,1-diphenylethylene (1,1-DPE, 36.0 mg, 0.40 mmol, 2.0 equiv). The resulting mixture was degassed and back-filled with nitrogen (N_2, high purity 4.8, >99.998%) before being transferred to the nitrogen-filled glovebox. Shortly after, 0.60 mL HMPA (0.33 M) was syringed into the reaction tube, which was capped by an aluminum seal with PTFE/silicone septum. The reaction tube was moved out of the glovebox and stirred at 100 ℃ for 12 hours under nitrogen.

Upon the completion of the reaction, to the resulted crude was added pinacol (189.1 mg, 1.6 mmol, 8.0 equiv) and 0.70 mL Et_3N. Then, the mixture was stirred at ambient temperature for 1.0 hour. After adding the 1,3,5-trimethoxybenzene (16.8 mg, 0.10 mmol, 1.0 equiv) as internal standard, it was quenched by 3.0 mL brine and 1.0 mL distilled H_2O and supplemented with 5.0 mL EtOAc for extraction. With vigorous shaking followed by unperturbed standing for a while to allow the two layers to separate, the upper organic layer was transferred and passed through a short-packed pipette column filled with Na_2SO_4 (3.0 cm) and silica gel (0.50 cm). The above-mentioned extraction process was repeated for another four times with EtOAc (5.0*4 mL). The filtered anhydrous organic solution (~25 mL) was collected in a 100 mL round bottom flask and concentrated on a rotary evaporator. The crude was subjected to ^1H NMR and GC-MS analysis for the reaction yield, and the results were summarized in the following table.

Supplementary Table 5.1.1. Examination of radical intermediacy

entry	*M*	*radical trapper*	*NMR yield (**1b**)*
1	M = H	none	64%
2	M = H	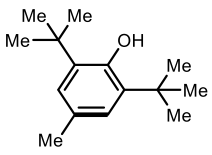 **BHT**	61%

3	M = H	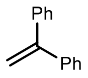<br>**1,1-DPE**	56%
4	M = Na	none	58%
5	M = Na	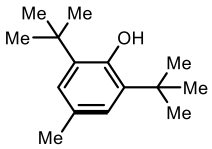<br>**BHT**	62%
6	M = Na	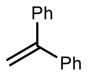<br>**1,1-DPE**	51%

These results collectively suggested that the radical mechanism might not be operative in our deoxygenative borylation system.

5.1.2. Examination of carbene intermediate

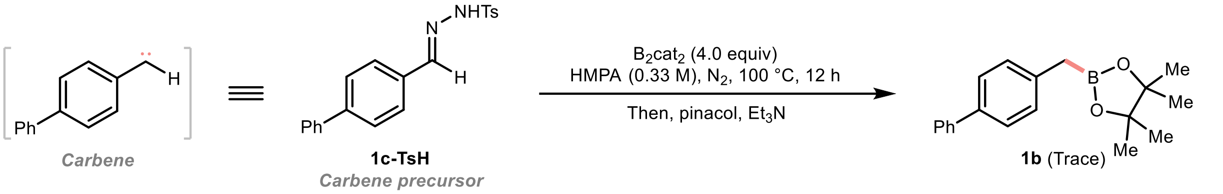

The procedure for examining carbene intermediate was described below.

To a flame-dried reaction tube (10 mL) equipped with a Teflon-coated magnetic stirring bar were added 4-biphenylcarboxaldehyde tosylhydrazone (**1c-TsH**, 70.0 mg, 0.20 mmol, 1.0 equiv) and B_2cat_2 (190.2 mg, 0.80 mmol, 4.0 equiv). The mixture was degassed and back-filled with nitrogen (N_2, high purity 4.8, >99.998%) before being transferred to the nitrogen-filled glovebox. Shortly after, 0.60 mL HMPA (0.33 M) was syringed into the reaction tube, which was capped by an aluminum seal with PTFE/silicone septum. The reaction tube was moved out of the glovebox and stirred at 100 ℃ for 12 hours under nitrogen.

Upon the completion of the reaction, to the resulted crude was added pinacol (189.1 mg, 1.6 mmol, 8.0 equiv) and 0.70 mL Et_3N. Then, the mixture was stirred at ambient temperature for 1.0 hour. After adding the 1,3,5-trimethoxybenzene (16.8 mg, 0.10 mmol, 1.0 equiv) as internal standard, it was quenched by 3.0 mL brine and 1.0 mL distilled H_2O and supplemented with 5.0 mL EtOAc for extraction. With vigorous shaking followed by unperturbed standing for a while to allow the two layers to separate, the upper organic layer was transferred and passed through a short-packed pipette column filled with Na_2SO_4 (3.0 cm) and silica gel (0.50 cm). The above-mentioned extraction process was repeated for another four times with EtOAc (5.0*4 mL). The filtered anhydrous organic solution (~25 mL) was collected in a 100 mL round

bottom flask and concentrated on a rotary evaporator. The crude was subjected to ^{1}H NMR and GC-MS analysis for the reaction yield.

A trace amount of desire benzylboronate was obtained as indicated by ^{1}H NMR and GC-MS, which suggested that carbene might not be an active intermediate in the current system.

5.1.3. Isolation of ketone side product from reductive dimerization of carboxylic acid

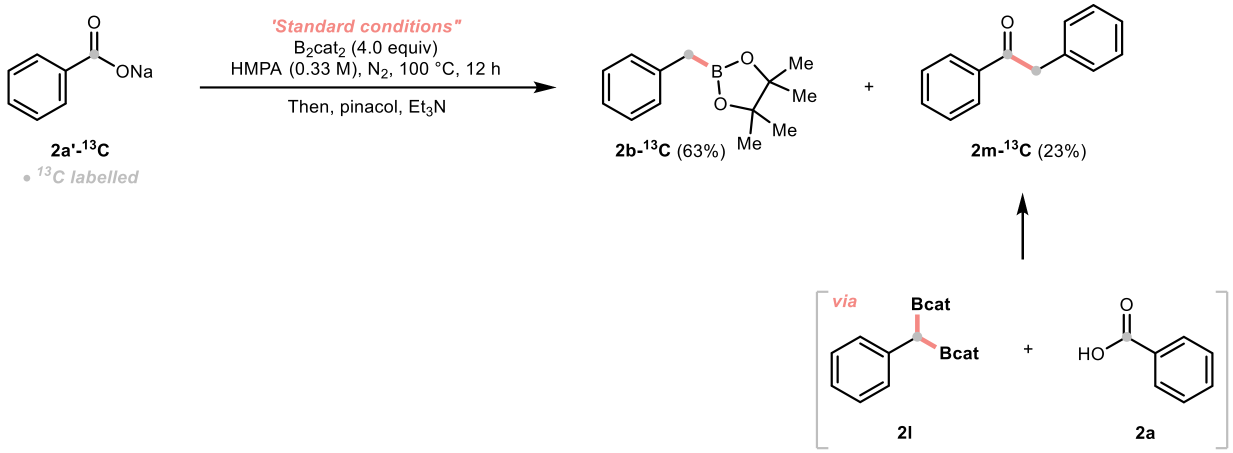

The procedure for isolating the ketone side product from the reductive dimerization of carboxylic acid was the same as the general procedure in **Section 4.1**.

The ketone side product could be derived from the reaction between the *gem*-diboronate and the carboxylic acid, which was reported by Liu's group.[14] This observation, combined with the result from **Section 5.2.1**, suggested that the *gem*-diboronate was the active intermediate in our deoxygenative borylation reaction.

5.2. Deoxygenated intermediates involved during carboxylic acid reduction

5.2.1. Reduction Intermediates involved for aromatic substrates

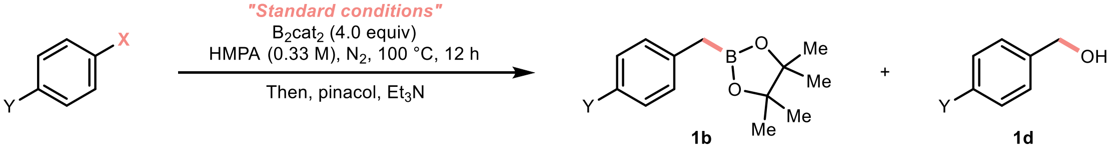

The procedure for examining the intermediates for aromatic carboxylic acids during the reaction was described below.

To a flame-dried reaction tube (10 mL) equipped with a Teflon-coated magnetic stirring bar were added the corresponding reduced forms of aromatic carboxylic acids (0.20 mmol, 1.0 equiv) and B_2cat_2 (190.2 mg, 0.80 mmol, 4.0 equiv). The resulting mixture was degassed and back-filled with nitrogen (N_2, high purity 4.8, >99.998%) before being transferred to the nitrogen-filled glovebox. Shortly after, 0.60 mL HMPA (0.33 M) was syringed into the reaction tube, which was capped by an aluminum seal with PTFE/silicone septum. The reaction tube was moved out of the glovebox and stirred at 100 °C for 12 hours under nitrogen.

Upon the completion of the reaction, to the resulted crude was added pinacol (189.1 mg, 1.6 mmol, 8.0 equiv) and 0.70 mL Et_3N. Then, the mixture was stirred at ambient temperature for 1.0 hour. After adding

the 1,3,5-trimethoxybenzene (16.8 mg, 0.10 mmol, 1.0 equiv) as internal standard, it was quenched by 3.0 mL brine and 1.0 mL distilled H_2O and supplemented with 5.0 mL EtOAc for extraction. With vigorous shaking followed by unperturbed standing for a while to allow the two layers to separate, the upper organic layer was transferred and passed through a short-packed pipette column filled with Na_2SO_4 (3.0 cm) and silica gel (0.50 cm). The above-mentioned extraction process was repeated for another four times with EtOAc (5.0*4 mL). The filtered anhydrous organic solution (~25 mL) was collected in a 100 mL round bottom flask and concentrated on a rotary evaporator. The crude was subjected to ^1H NMR and GC-MS analysis for the reaction yield, and the results were summarized in the following table.

Supplementary Table 5.2.1. Effect of some potential reduced forms of aromatic carboxylic acids

entry	*reduction intermediate*	*NMR yield of RCH_2Bpin*	*NMR yield RCH_2OH*
1	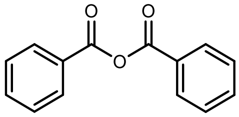 **2a$_2$**	49%	3%
2	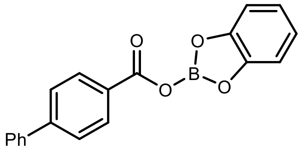 **1a-OBcat**	42%	trace
3	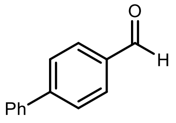 **1c**	70%	11%
4	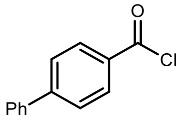 **1a-Cl**	trace	trace
5	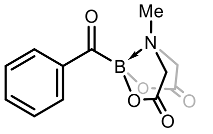 **2a-BMIDA**	60%	trace
6	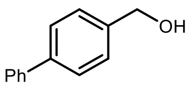 **1d**	trace	>99%

7	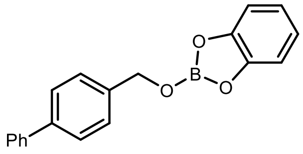<br>**1d-OBcat**	trace	>99%
8	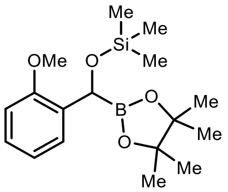<br>**52d-OTMs**	35%	trace
9	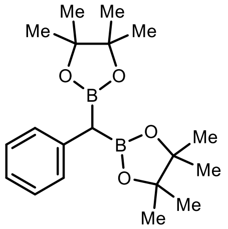<br>**2b'**	>99%	trace

Note:

1. *The OBcat alcohol **1d-OBcat** was prepared according to a modified procedure based on Hreczycho's method by using H-Bcat.*[15]
2. *The OBcat anhydride **1a-OBcat** was prepared according to a modified protocol based on Ma's work*[16] *by mixing an equal molar of the corresponding carboxylic acid and catecholborane (H-Bcat) in neat and stirring under nitrogen atmosphere for 30 minutes. The obtained solid crude was directly subjected to the next step without purification. Independent ^{1}H NMR analysis showed the full conversion of the carboxylic acid and H-Bcat, and ^{11}B indicated the formation of the OBcat anhydride (δ = 22.9 ppm in CD_2Cl_2). Besides, the formation of the corresponding aldehyde was not observed.*
3. *The acylboronic acid MIDA ester **2a-BMIDA** was synthesized according to Perrin's procedure.*[17]
4. *The bisborylated toluene compound **2b'** was prepared based on the method reported by Wang's group.*[18]
5. *Aldehyde and anhydride could give the desired boronate product in good yields. Although their possibility of being active intermediates in this transformation could not be completely excluded, they were less likely to be involved in this transformation since they were highly sensitive toward hydride reduction (H-Bcat was present if carboxylic acid is the starting material) and will be reduced to the unreactive alcohol. Besides, anhydride as the reaction intermediate could not explain the cases with reaction yields higher than 50%.*
6. *Comparing entries 5 and 6, although Cl^- is a better leaving group than $B(cat)O^-$, the acid chloride **1a-Cl** is unreactive under the standard condition, which demonstrated the importance of boron substituent in the OBcat anhydride **1a-OBcat**.*

5.2.2. Reduction Intermediates involved in aliphatic substrates

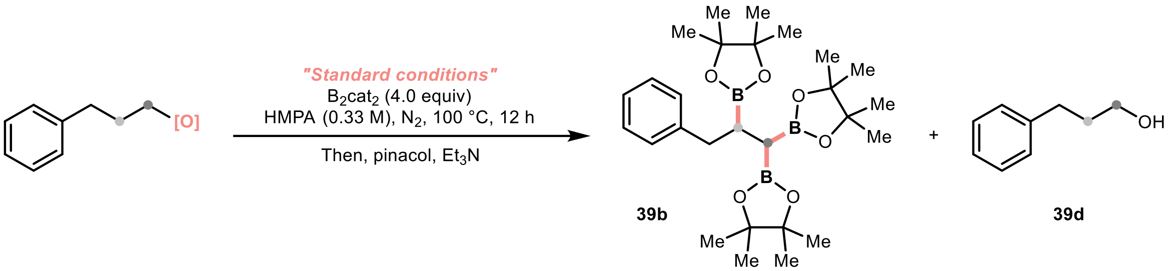

39b **39d**

The procedure for examining the intermediates for aliphatic carboxylic acids during the reaction was described below.

To a flame-dried reaction tube (10 mL) equipped with a Teflon-coated magnetic stirring bar were added the corresponding reduced forms of aliphatic carboxylic acids (0.20 mmol, 1.0 equiv) and B_2cat_2 (190.2 mg, 0.80 mmol, 4.0 equiv). The reaction tube was covered by a rubber septum, and the resulting mixture was degassed and back-filled with nitrogen (N_2, high purity 4.8, >99.998%) before being transferred to the nitrogen-filled glovebox. Shortly after, 0.60 mL HMPA (0.33 M) was syringed into the reaction tube, which was capped by an aluminum seal with PTFE/silicone septum. The reaction tube was moved out of the glovebox and stirred at 100 ℃ for 12 hours under nitrogen.

Upon the completion of the reaction, to the resulted crude was added pinacol (189.1 mg, 1.6 mmol, 8.0 equiv) and 0.70 mL Et_3N. Then, the mixture was stirred at ambient temperature for 1.0 hour. After adding the 1,3,5-trimethoxybenzene (16.8 mg, 0.10 mmol, 1.0 equiv) as internal standard, it was quenched by 3.0 mL brine and 1.0 mL distilled H_2O and supplemented with 5.0 mL EtOAc for extraction. With vigorous shaking followed by unperturbed standing for a while to allow the two layers to separate, the upper organic layer was transferred and passed through a short-packed pipette column filled with Na_2SO_4 (3.0 cm) and silica gel (0.50 cm). The above-mentioned extraction process was repeated for another four times with EtOAc (5.0*4 mL). The filtered anhydrous organic solution (~25 mL) was collected in a 100 mL round bottom flask and concentrated on a rotary evaporator. The crude was subjected to ^{1}H NMR and GC-MS analysis for the reaction yield, and the results were summarized in the following table.

Supplementary Table 5.2.2. Effect of some potential reduced forms of aliphatic carboxylic acids

entry	*reduction intermediate*	*NMR yield (**39b**)*	*NMR yield (**39d**)*
1	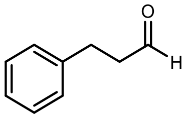 **39c**	60%	23%
2	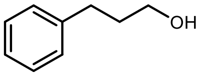 **39d**	ND	>99%
3	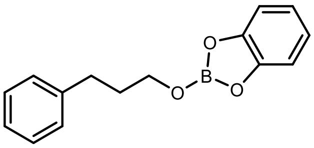 **39d-OBcat**	ND	90%

4	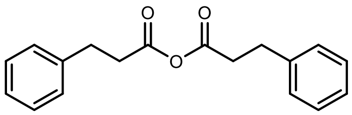 **39a₂**	47%	14%
5	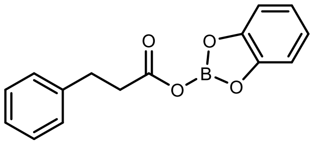 **39a-OBcat**	67%	13%

Note:

1. *The OBcat alcohol **39d-OBcat** was prepared according to a modified procedure based on Hreczycho's method by using H-Bcat.*[15]
2. *The OBcat anhydride **39a-OBcat** was prepared according to a modified protocol based on Ma's work*[16] *by mixing an equal molar of the corresponding carboxylic acid and catecholborane (H-Bcat) in neat and stirring under nitrogen atmosphere for 30 minutes. The obtained solid crude was directly subjected to the next step without purification. Independent* ^{1}*H NMR analysis showed the full conversion of the carboxylic acid and H-Bcat, and* ^{11}*B indicated the OBcat anhydride formation (*δ = 23.1 ppm in CD_2Cl_2*). Besides, the formation of the corresponding aldehyde was not observed.*
3. *Aldehyde and anhydride could give the desired boronate product in good yields. Although their possibility of being active intermediates in this transformation could not be completely excluded, they were less likely to be involved in this transformation since they were highly sensitive toward hydride reduction (H-Bcat was present if carboxylic acid is the starting material) and will be reduced to the unreactive alcohol. Besides, anhydride as the reaction intermediate could not explain the cases with reaction yields higher than 50%.*

5.2.3. Examination of the intermediacy of vinyl boronate

5.2.3.1. Reactivity of vinyl boronates

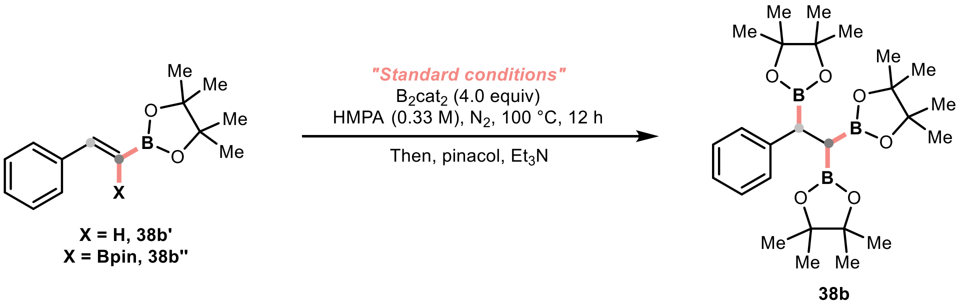

X = H, 38b'
X = Bpin, 38b''
38b

The procedure for examining the intermediacy for vinyl boronate was described below.

To a flame-dried reaction tube (10 mL) equipped with a Teflon-coated magnetic stirring bar were added *trans*-2-phenylvinylboronic acid pinacol ester (**38b'**, 46.0 mg, 0.20 mmol, 1.0 equiv) or 2,2'-(2-phenylethene-1,1-diyl)bis(4,4,5,5-tetramethyl-1,3,2-dioxaborolane) (**38b''**, 71.2 mg, 0.20 mmol, 1.0 equiv) and B_2cat_2 (190.2 mg, 0.80 mmol, 4.0 equiv). The resulting mixture was degassed and back-filled with nitrogen (N_2, high purity 4.8, >99.998%) before being transferred to the nitrogen-filled glovebox. Shortly

after, 0.60 mL HMPA (0.33 M) was syringed into the reaction tube, which was capped by an aluminum seal with PTFE/silicone septum. The reaction tube was moved out of the glovebox and stirred at 100 °C for 12 hours under nitrogen.

Upon the completion of the reaction, to the resulted crude was added pinacol (189.1 mg, 1.6 mmol, 8.0 equiv) and 0.70 mL Et_3N. Then, the mixture was stirred at ambient temperature for 1.0 hour. After adding the 1,3,5-trimethoxybenzene (16.8 mg, 0.10 mmol, 1.0 equiv) as internal standard, it was quenched by 3.0 mL brine and 1.0 mL distilled H_2O and supplemented with 5.0 mL EtOAc for extraction. With vigorous shaking followed by unperturbed standing for a while to allow the two layers to separate, the upper organic layer was transferred and passed through a short-packed pipette column filled with Na_2SO_4 (3.0 cm) and silica gel (0.50 cm). The above-mentioned extraction process was repeated for another four times with EtOAc (5.0*4 mL). The filtered anhydrous organic solution (~25 mL) was collected in a 100 mL round bottom flask and concentrated on a rotary evaporator. The crude was subjected to ^1H NMR and GC-MS analysis for the reaction yield.

Supplementary Table 5.2.3.1. Intermediacy of vinylboronates

entry	*vinylboronate*	*diboron*	*conversion*	*yield (**38b**)*
1	X = H	B_2cat_2	>99%	95%
2	X = Bpin	B_2cat_2	49%	19%
3	X = H	B_2pin_2	0%	0%

The significantly higher conversion and product yield of the reaction with vinylboronate **38b’** than the diboronate **38b’’** indicated that **38b’** was more likely to be the active intermediate in the deoxygenative triboration transformation.

Interestingly, vinylboronate **38b’** has been proposed as the key intermediate for the 1,1,2-tris(boronate) synthesis via subsequent vicinal diboration. However, in all cases, B_2pin_2 aided by bases or metal catalysts were used,[6, 19, 20] which were shown ineffective in our work.

5.2.3.2. Identification of vinyl boronates in reaction crude

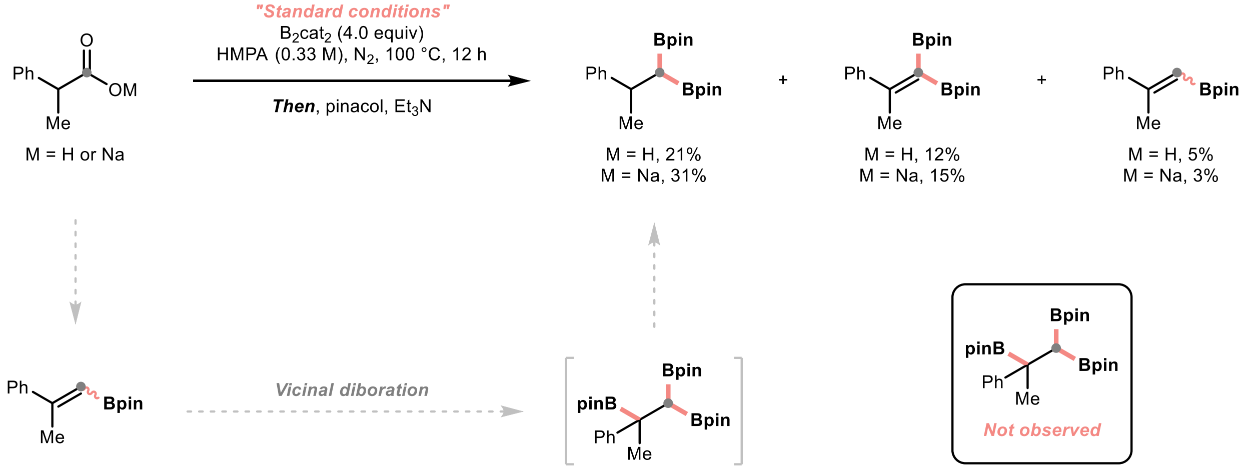

To study the reactivities of α-branched carboxylic acids, several representative members in this class, including 2-phenylpropionic acid, 2-methylbutanoic acid, ibuprofen, and naproxen, were utilised. We

carefully analysed the reaction mixture in the case of 2-phenylpropionic acid using NMR and HRMS. The results were shown below.

Although the desired tris(boronate) product was not observed in this reaction, several boron-containing products were identified.

1) 1,1-Alkyldiboronate: this side product could be attributed to the selective mono-protodeboronation of desired tris(boronate) product. We believed that the strained scaffold of tris(boronate) product promoted such a hydrolysis step during the reaction since it occurred to a much smaller extent in the case of linear acid examples. In addition, the internal chelation between Bpin groups was proposed to assist the deborylation step,[20] where the carbanion bearing α-phenyl ring was favoured.

2) Vinylboronate: Due in part to the steric hindrance of this branched substrate, the rate of the vicinal diborylation step was decreased, allowing the accumulation of vinylboronate and 1,1-vinyldiboronate in the reaction mixture. The formation of these intermediates was consistent with our mechanistic findings, which indicates the important role of vinyl(di)boronates in the deoxygenative borylation of aliphatic substrates.

Similar results were obtained in the cases of 2-methylbutanoic acid, ibuprofen, and naproxen (not shown above).

5.3. Isotope labelling experiment

5.3.1. D_2O experiment

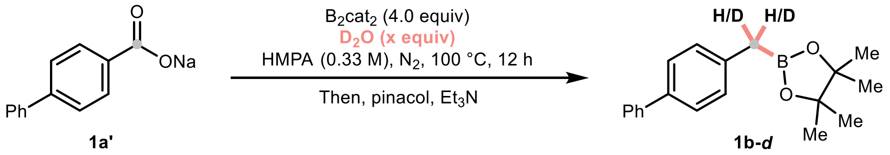

The procedure for examining the effect of the D_2O additive was described below.

To a flame-dried reaction tube (10 mL) equipped with a Teflon-coated magnetic stirring bar were added 4-biphenylcarboxylic acid sodium salt (**1a’**, 44.0 mg, 0.20 mmol, 1.0 equiv) and B_2cat_2 (190.2 mg, 0.80 mmol, 4.0 equiv). The resulting mixture was degassed and back-filled with nitrogen (N_2, high purity 4.8, >99.998%) before being transferred to the nitrogen-filled glovebox. Shortly after, 0.60 mL HMPA (0.33 M) was syringed into the reaction tube, followed by an autopipette spike of the corresponding amount of D_2O (*x* equiv). The reaction tube was capped by an aluminum seal with PTFE/silicone septum. The reaction tube was moved out of the glovebox and stirred at 100 °C for 12 hours under nitrogen.

Upon the completion of the reaction, to the resulted crude was added pinacol (189.1 mg, 1.6 mmol, 8.0 equiv) and 0.70 mL Et_3N. Then, the mixture was stirred at ambient temperature for 1.0 hour. After adding the 1,3,5-trimethoxybenzene (16.8 mg, 0.10 mmol, 1.0 equiv) as internal standard, it was quenched by 3.0 mL brine and 1.0 mL distilled H_2O and supplemented with 5.0 mL EtOAc for extraction. With vigorous shaking followed by unperturbed standing for a while to allow the two layers to separate, the upper organic layer was transferred and passed through a short-packed pipette column filled with Na_2SO_4 (3.0 cm) and silica gel (0.50 cm). The above-mentioned extraction process was repeated for another four times with EtOAc (5.0*4 mL). The filtered anhydrous organic solution (~25 mL) was collected in a 100 mL round bottom flask and concentrated on a rotary evaporator. The crude was subjected to ^1H NMR, ^2H NMR and GC-MS analysis, which was then subjected to flash column chromatography on silica gel to furnish the titled compound. The results were summarized in the following table.

Supplementary Table 5.3.1. Effect of D_2O additive

entry	*equivalence of D_2O*	*NMR yield (**1b-d**)*	*overall D content*
1	x = 1.0	47%	64%
2	x = 2.0	29%	73%

5.3.2. Experiment with $ArCO_2D$

5.3.2.1. Preparation of $ArCO_2D$

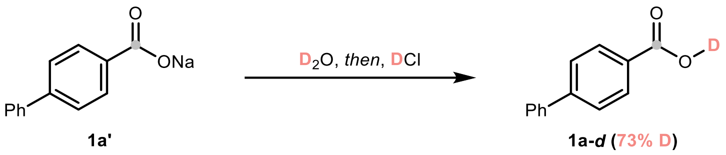

The procedure for preparing the 4-biphenylcarboxylic acid-*d* was described below.

Under argon, to a flame-dried round bottom flask (25 mL) equipped with a Teflon-coated magnetic stirring bar were added 4-biphenylcarboxylic acid sodium salt (**1a'**, 220.0 mg, 1.0 mmol, 1.0 equiv), which was followed by deuterium oxide (D_2O) until the carboxylate was fully dissolved under mild heating conditions. After cooling the clear solution to room temperature, to the carboxylate solution was added deuterium chloride aqueous solution (DCl, 35 wt. % in D_2O, ≥99 atom % D) and adjust the pH to around 2.0 for precipitating the carboxylic acid. The solid was obtained by vacuum filtration under argon, which was dried by a freeze dryer overnight to give off-white power. The deuterium content was determined by ^1H NMR with molecular sieve-dried $CDCl_3$ and confirmed by ^2H NMR.

5.3.2.2. Deoxygenative borylation of $ArCO_2D$

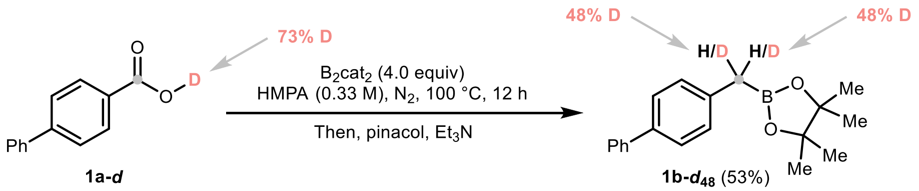

The procedure for examining the effect of the $ArCO_2D$ was described below.

To a flame-dried reaction tube (10 mL) equipped with a Teflon-coated magnetic stirring bar were added 4-biphenylcarboxylic acid-*d* (**1a-*d***, 39.8 mg, 0.20 mmol, 1.0 equiv) and B_2cat_2 (190.2 mg, 0.80 mmol, 4.0 equiv). The resulting mixture was degassed and back-filled with nitrogen (N_2, high purity 4.8, >99.998%) before being transferred to the nitrogen-filled glovebox. Shortly after, 0.60 mL HMPA (0.33 M) was syringed into the reaction tube. The reaction tube was capped by an aluminum seal with PTFE/silicone septum. The reaction tube was moved out of the glovebox and stirred at 100 ℃ for 12 hours under nitrogen.

Upon the completion of the reaction, to the resulted crude was added pinacol (189.1 mg, 1.6 mmol, 8.0 equiv) and 0.70 mL Et_3N. Then, the mixture was stirred at ambient temperature for 1.0 hour. After adding the 1,3,5-trimethoxybenzene (16.8 mg, 0.10 mmol, 1.0 equiv) as internal standard, it was quenched by 3.0 mL brine and 1.0 mL distilled H_2O and supplemented with 5.0 mL EtOAc for extraction. With vigorous shaking followed by unperturbed standing for a while to allow the two layers to separate, the upper organic layer was transferred and passed through a short-packed pipette column filled with Na_2SO_4 (3.0

cm) and silica gel (0.50 cm). The above-mentioned extraction process was repeated for another four times with EtOAc (5.0*4 mL). The filtered anhydrous organic solution (~25 mL) was collected in a 100 mL round bottom flask and concentrated on a rotary evaporator. The crude was subjected to ^{1}H NMR and GC-MS analysis, which was then subjected to flash column chromatography on silica gel to furnish the titled compound. The deuterium content of the purified product was determined by ^{1}H NMR and confirmed by ^{2}H NMR.

5.4. Role of B_2cat_2

5.4.1. Possible active forms of B_2cat_2 during the reactions of aromatic carboxylic acids

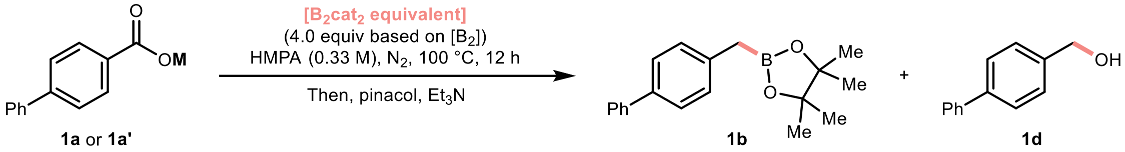

The procedure for examining the active forms of B_2cat_2 with aromatic carboxylic acids was described below.

To a flame-dried reaction tube (10 mL) equipped with a Teflon-coated magnetic stirring bar were added 4-biphenylcarboxylic acid (**1a**, 39.6 mg, 0.20 mmol, 1.0 equiv) or 4-biphenylcarboxylic acid sodium salt (**1a'**, 44.0 mg, 0.20 mmol, 1.0 equiv) and the corresponding borylating reagent (0.80 mmol, 4.0 equiv based on [B_2]). The resulting mixture was degassed and back-filled with nitrogen (N_2, high purity 4.8, >99.998%) before being transferred to the nitrogen-filled glovebox. Shortly after, 0.60 mL HMPA (0.33 M) was syringed into the reaction tube, which was capped by an aluminum seal with PTFE/silicone septum. The reaction tube was moved out of the glovebox and stirred at 100 °C for 12 hours under nitrogen.

Upon the completion of the reaction, to the resulted crude was added pinacol (189.1 mg, 1.6 mmol, 8.0 equiv) and 0.70 mL Et_3N. Then, the mixture was stirred at ambient temperature for 1.0 hour. After adding the 1,3,5-trimethoxybenzene (16.8 mg, 0.10 mmol, 1.0 equiv) as internal standard, it was quenched by 3.0 mL brine and 1.0 mL distilled H_2O and supplemented with 5.0 mL EtOAc for extraction. With vigorous shaking followed by unperturbed standing for a while to allow the two layers to separate, the upper organic layer was transferred and passed through a short-packed pipette column filled with Na_2SO_4 (3.0 cm) and silica gel (0.50 cm). The above-mentioned extraction process was repeated for another four times with EtOAc (5.0*4 mL). The filtered anhydrous organic solution (~25 mL) was collected in a 100 mL round bottom flask and concentrated on a rotary evaporator. The crude was subjected to ^{1}H NMR and GC-MS analysis for the reaction yield, and the results were summarized in the following table.

Supplementary Table 5.4.1. Effect of some potential active forms of B_2cat_2 for aromatic substrates

entry	*M*	*borylating reagent*	*NMR yield (**1b**)*	*NMR yield (**1d**)*
1	M = H	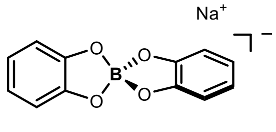 **NaB(cat)₂**	ND *(chaotic mixture)*	ND *(chaotic mixture)*

2	M = Na	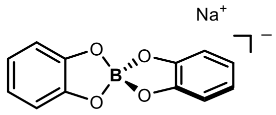 NaB(cat)$_2$	ND	ND
3	M = H	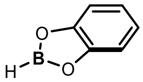 H-Bcat	ND	>99%
4	M = Na	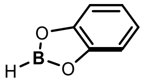 H-Bcat	ND	>99%

5.4.2. Possible active forms of B_2cat_2 during the reactions of aliphatic carboxylic acids

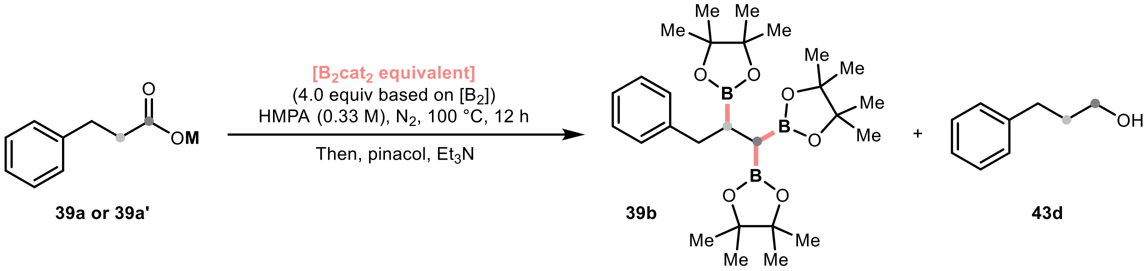

The procedure for examining the active forms of B_2cat_2 with aliphatic carboxylic acids was described below.

To a flame-dried reaction tube (10 mL) equipped with a Teflon-coated magnetic stirring bar were added hydrocinnamic acid (**39a**, 30.0 mg, 0.20 mmol, 1.0 equiv) or sodium hydrocinnamate (**39a'**, 34.4 mg, 0.20 mmol, 1.0 equiv) and the corresponding borylating reagent (0.80 mmol, 4.0 equiv based on [B_2]). The resulting mixture was degassed and back-filled with nitrogen (N_2, high purity 4.8, >99.998%) before being transferred to the nitrogen-filled glovebox. Shortly after, 0.60 mL HMPA (0.33 M) was syringed into the reaction tube, which was capped by an aluminum seal with PTFE/silicone septum. The reaction tube was moved out of the glovebox and stirred at 100 ℃ for 12 hours under nitrogen.

Upon the completion of the reaction, to the resulted crude was added pinacol (189.1 mg, 1.6 mmol, 8.0 equiv) and 0.70 mL Et_3N. Then, the mixture was stirred at ambient temperature for 1.0 hour. After adding the 1,3,5-trimethoxybenzene (16.8 mg, 0.10 mmol, 1.0 equiv) as internal standard, it was quenched by 3.0 mL brine and 1.0 mL distilled H_2O and supplemented with 5.0 mL EtOAc for extraction. With vigorous shaking followed by unperturbed standing for a while to allow the two layers to separate, the upper organic layer was transferred and passed through a short-packed pipette column filled with Na_2SO_4 (3.0 cm) and silica gel (0.50 cm). The above-mentioned extraction process was repeated for another four times with EtOAc (5.0*4 mL). The filtered anhydrous organic solution (~25 mL) was collected in a 100 mL round bottom flask and concentrated on a rotary evaporator. The crude was subjected to ^{1}H NMR and GC-MS analysis for the reaction yield, and the results were summarized in the following table.

Supplementary Table 5.4.2. Effect of some potential active forms of B_2cat_2 for aliphatic substrates

entry	*M*	*borylating reagent*	*NMR yield (**39b**)*	*NMR yield (**39d**)*
1	M = H	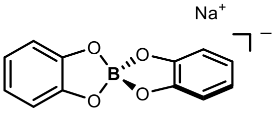 **NaB(cat)$_2$**	ND	ND
2	M = Na	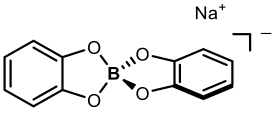 **NaB(cat)$_2$**	ND	ND
3	M = H	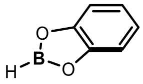 **H-Bcat**	ND	>99%
4	M = Na	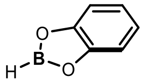 **H-Bcat**	ND	>99%

5.4.3. ^{11}B NMR experiments

5.4.3.1. ^{11}B NMR experiments with B_2cat_2 and HMPA

The procedure of recording ^{11}B NMR of B_2cat_2 in HMPA and DCM was described below.

To a **quartz** NMR tube was charged B_2cat_2 (23.7 mg, 0.10 mmol), which was followed by the injection of 0.40 mL solvent (0.25 M). The tube was shaken for homogeneity or sonicated if necessary. Then, the solution was submitted to ^{11}B NMR analysis, and the spectra were stacked below.

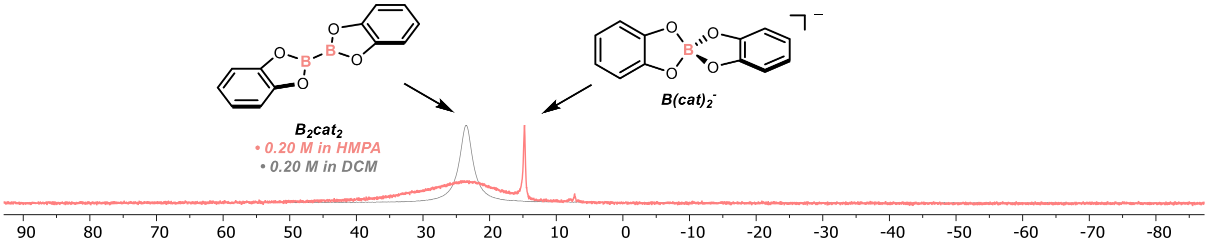

Supplementary Figure 5.4.3.1. Stacked ^{11}B NMR spectra of B_2cat_2 in HMPA (pink) and DCM (grey)

Compared to the sharp peak of the spectrum recorded in DCM (δ = 30.8 ppm), the broadened and upfielded signal in HMPA (δ = 23.8 ppm) might indicate the loose coordination of the basic phosphoramide oxygen of HMPA to the acidic boron centre in B_2cat_2. The significant broadening might be caused by the rapid interconversion of the on-and-off coordination status between these two species.

Another sharp peak (δ = 14.8 ppm) might be the $B(cat)_2^-$, whose identity was confirmed by independent synthesis.

All the experimental results above were consistent with the observation in related literature.[9, 11, 12]

5.4.3.2. ^{11}B NMR experiments with B_2cat_2 and NaOAc

The procedure of recording ^{11}B NMR of B_2cat_2 with NaOAc was described below.

To a vial was charged B_2cat_2 (23.7 mg, 0.10 mmol, 1.0 equiv), sodium acetate (NaOAc, 8.2 mg, 0.10 mmol, 1.0 equiv) and 15-crown-5 (22.0 mg, 0.10 mmol, 1.0 equiv), which was followed by the injection of 1.5 mL CD_2Cl_2 (0.067 M). The reaction mixture was sonicated for 2 hours for homogeneity. The solution was then transferred to a **quartz** NMR tube, which was submitted to ^{11}B NMR analysis. The spectra obtained were stacked with the blank one without NaOAc.

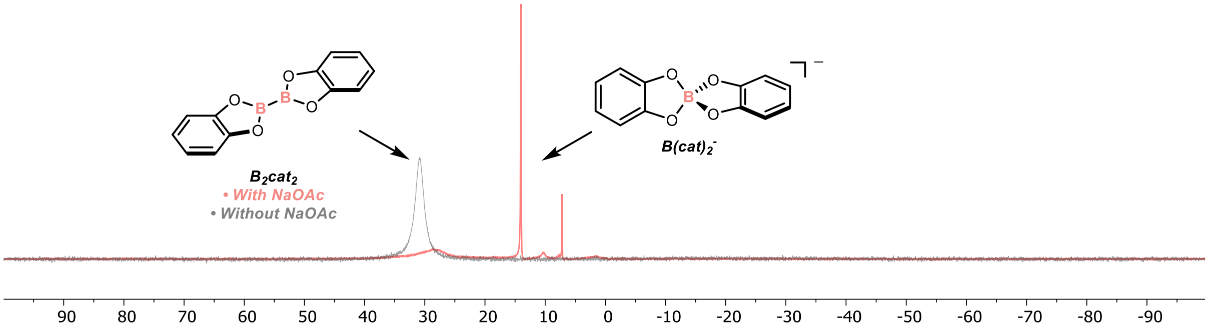

Supplementary Figure 5.4.3.2. Stacked ^{11}B NMR spectra of B_2cat_2 in CD_2Cl_2 with NaOAc (pink) and without NaOAc (grey)

Crown ether was added to the solution to enhance the solubility of the sodium salt, and it does not coordinate with the boron centre of B_2cat_2. This was confirmed by comparing the spectra of B_2cat_2 in CD_2Cl_2 with or without 15-crown-5. Adding the ether does not change the signal of B_2cat_2 in ^{11}B NMR.

Compared to the sharp peak of the spectrum recorded in CD_2Cl_2 (δ = 30.8 ppm), the broadened and upfielded signal in the presence of NaOAc (δ = 28.5 ppm) might indicate the loose coordination of the anionic acetate to the acidic boron centre in B_2cat_2. The significant broadening might be caused by the rapid interconversion of the on-and-off coordination status between these two species.

The sharp peak (δ = 14.0 ppm) might be the $B(cat)_2^-$, whose identity was confirmed by independent synthesis. However, the peak (δ = 7.2 ppm) remained unknown.

5.4.3.3. ^{11}B NMR experiments with B_2cat_2 and RCO_2H

The procedure of recording ^{11}B NMR of B_2cat_2 with benzoic acid and acetic acid was described below.

To a **quartz** NMR tube was charged B_2cat_2 (23.7 mg, 0.10 mmol, 1.0 equiv) and the corresponding carboxylic acid (benzoic acid, 12.2 mg, 0.10 mmol, 1.0 equiv; acetic acid, 6.0 mg, 0.10 mmol, 1.0 equiv), which was followed by the injection of 0.50 mL CD_2Cl_2 (0.20 M). The tube was shaken for homogeneity or sonicated if necessary. After standing at room temperature for 48 hours, the solution was submitted to ^{11}B NMR analysis, and the spectra were shown below.

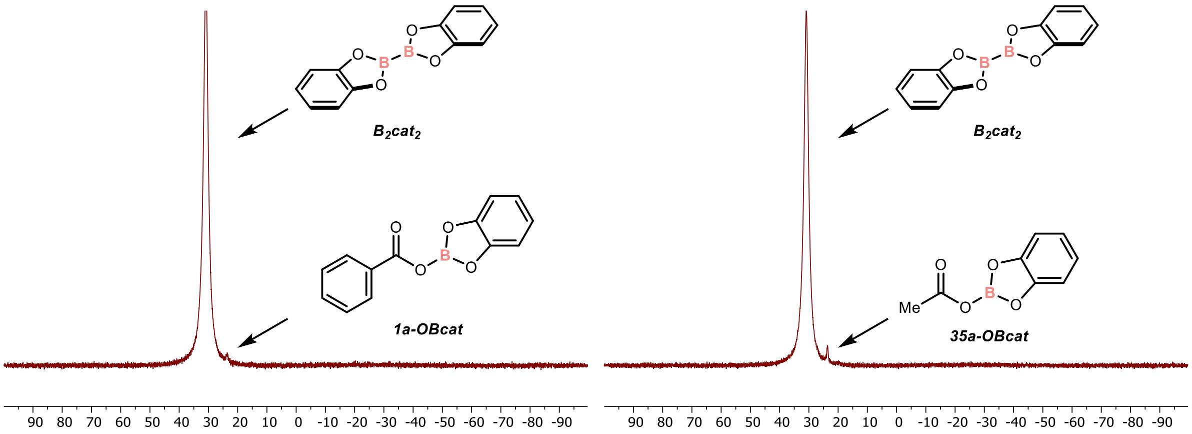

Supplementary Figure 5.4.3.3. ^{11}B NMR spectra of B_2cat_2 and $PhCO_2H$ (left) and HOAc (right) in CD_2Cl_2 after 48 hours

The peak (δ = 23.6 and 23.5 ppm in the case of benzoic and acetic acid, respectively) indicated the formation of the corresponding OBcat carboxylate,[16] which was essential for the success of subsequent C-O cleavage events and removal of the problematic proton in the free carboxylic acid. HMPA was not opted as the solvent in these experiments due to the significant broadening effect, which prevented clear visualization of the OBcat anhydride formation.

5.4.4. H_2 Evolution from H-Bcat by-product

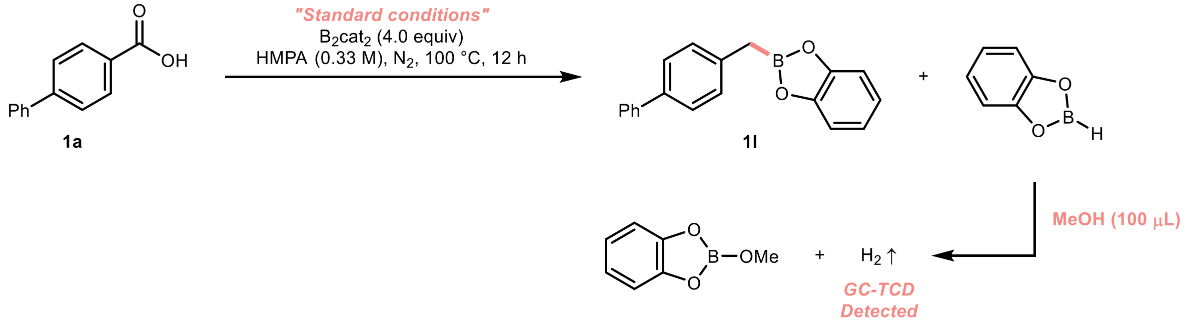

The procedure for detecting the H-Bcat by-product was described below.

To a flame-dried reaction tube (10 mL) equipped with a Teflon-coated magnetic stirring bar were added 4-biphenylcarboxylic acid (**1a**, 39.6 mg, 0.20 mmol, 1.0 equiv) and B_2cat_2 (190.2 mg, 0.80 mmol, 4.0 equiv). The resulting mixture was degassed and back-filled with nitrogen (N_2, high purity 4.8, >99.998%) before being transferred to the nitrogen-filled glovebox. Shortly after, 0.60 mL HMPA (0.33 M) was syringed into the reaction tube. The reaction tube was capped by an aluminum seal with PTFE/silicone septum. The reaction tube was moved out of the glovebox and stirred at 100 °C for 12 hours under nitrogen.

Upon the completion of the reaction, when the mixture was cooled down to room temperature, 100 μL MeOH was quickly injected inside. With vigorous shaking, significant bubbling and exothermic phenomena were observed, indicating the formation of some gaseous species. The headspace atmosphere was sampled by a gas syringe and submitted to GC-TCD analysis. The identity of the gaseous product was confirmed by comparing the authentic H_2 sample.

Note: Peaks at around 5 and 10 minutes arose from the system-default valve switch.

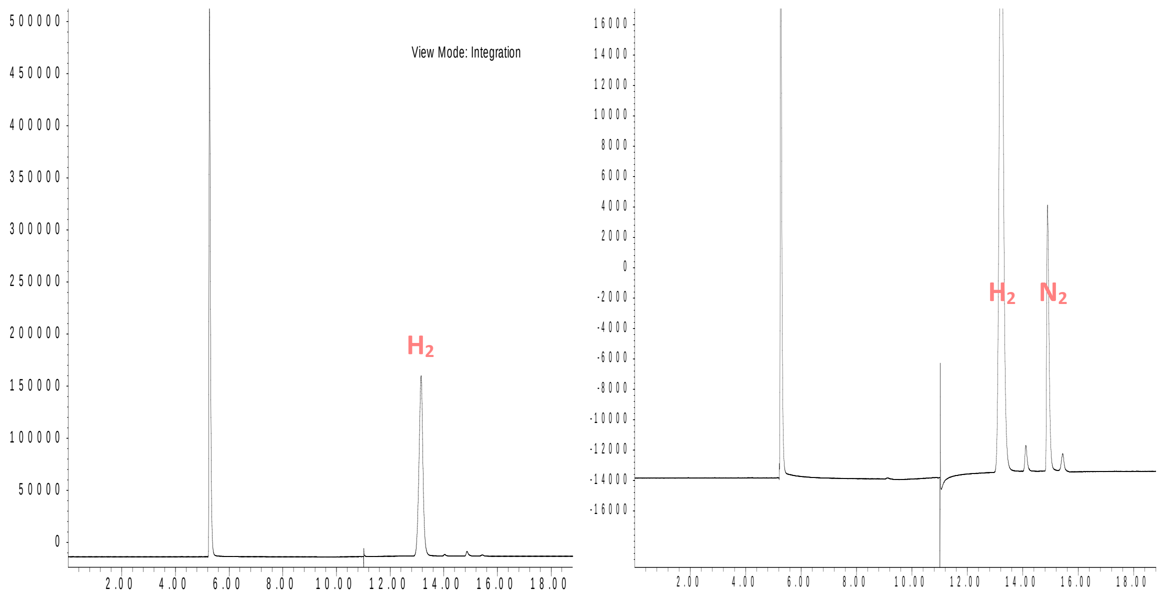

Supplementary Figure 5.4.4.GC-TCD Chromatographs of H_2 standard (left) and headspace sample of the deoxy-borylation reaction (right)

5.5. Role of HMPA

5.5.1. Deoxygenative borylation in non-coordinating DCM with Lewis base additive

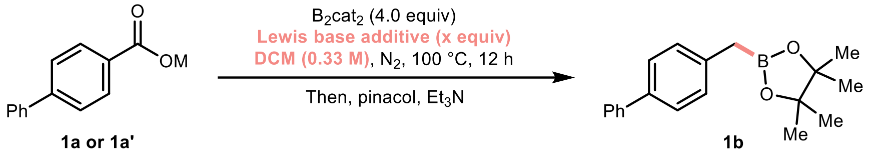

The procedure for examining the effect of the Lewis base additive in DCM was described below.

To a flame-dried reaction tube (10 mL) equipped with a Teflon-coated magnetic stirring bar were added 4-biphenylcarboxylic acid sodium salt (**1a'**, 44.0 mg, 0.20 mmol, 1.0 equiv) or 4-biphenylcarboxylic acid (**1a**, 39.6 mg, 0.20 mmol, 1.0 equiv), B_2cat_2 (190.2 mg, 0.80 mmol, 4.0 equiv) and the corresponding Lewis base additive. The resulting mixture was degassed and back-filled with nitrogen (N_2, high purity 4.8, >99.998%) before being transferred to the nitrogen-filled glovebox. Shortly after, 0.60 mL DCM (0.33 M) was syringed into the reaction tube. The reaction tube was capped by an aluminum seal with PTFE/silicone septum. The reaction tube was moved out of the glovebox and stirred at 100 ℃ for 12 hours under nitrogen.

Upon the completion of the reaction, to the resulted crude was added pinacol (189.1 mg, 1.6 mmol, 8.0 equiv) and 0.70 mL Et_3N. Then, the mixture was stirred at ambient temperature for 1.0 hour. After adding the 1,3,5-trimethoxybenzene (16.8 mg, 0.10 mmol, 1.0 equiv) as internal standard, it was quenched by 3.0 mL brine and 1.0 mL distilled H_2O and supplemented with 5.0 mL EtOAc for extraction. With vigorous shaking followed by unperturbed standing for a while to allow the two layers to separate, the upper organic layer was transferred and passed through a short-packed pipette column filled with Na_2SO_4 (3.0 cm) and silica gel (0.50 cm). The above-mentioned extraction process was repeated for another four times with EtOAc (5.0*4 mL). The filtered anhydrous organic solution (~25 mL) was collected in a 100 mL round bottom flask and concentrated on a rotary evaporator. The crude was subjected to ^1H NMR and GC-MS

analysis, which was then subjected to flash column chromatography on silica gel to furnish the titled compound. The results were summarized in the following table.

Supplementary Table 5.5.1. Effect of Lewis base additive

entry	*acid substrate*	*basic additive*	*NMR yield (**1b**)*
1	M = Na	DMAP (1.0 equiv)	5%
2	M = Na	HMPA (3.0 equiv)	32%
3	M = H	DMAP (1.0 equiv)	trace
4	M = H	HMPA (3.0 equiv)	23%

5.5.2. NMR experiments

5.5.2.1. ^{1}H NMR experiments

The procedure of recording ^{1}H NMR of B_2cat_2 and HMPA in CD_2Cl_2 was described below.

To an NMR tube was charged B_2cat_2 (23.8 mg, 0.10 mmol, 1.0 equiv), which was followed by the injection of 0.40 mL CD_2Cl_2 (0.25 M). The tube was shaken for homogeneity or sonicated if necessary. Then, the solution was submitted to ^{1}H NMR analysis, and the blank spectrum in the absence of HMPA was recorded.

By using the CH_2Cl_2 residue (δ = 5.36 ppm) in the deuterated solvent as internal standard, the corresponding amount of HMPA (17.9 to 89.5 mg, 0.10 to 0.50 mmol, 1.0 to 8.0 equiv) was injected inside the NMR tube with a gradient increment of HMPA loading (on 17.9 mg, 0.10 mmol, 1.0 equiv increment basis). After each injection, the ^{1}H NMR was recorded.

Upon completion, the spectra were stacked and shown below.

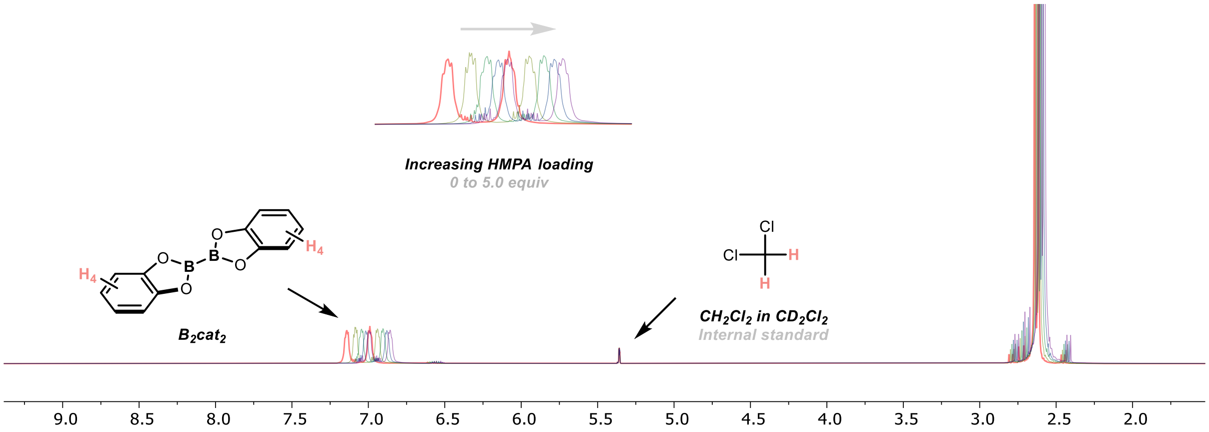

Supplementary Figure 5.5.2.1. Stacked ^{1}H NMR spectra of B_2cat_2 in CD_2Cl_2 with increasing loading of HMPA

CD_2Cl_2 was used in this experiment due to its non-coordinating property and good solubility for B_2cat_2, which is beneficial for signal acquisition in ^{1}H NMR. The upfield shift of the B_2cat_2 proton signal might indicate the coordination of the oxygen atom of HMPA to B_2cat_2, which gave a partial negative charge to the boron atom.

5.5.2.2. ^{31}P NMR experiments

The procedure of recording ^{31}P NMR of B_2cat_2 and HMPA in CD_2Cl_2 was described below.

To an NMR tube was charged B_2cat_2 (23.8 mg, 0.10 mmol, 1.0 equiv) and tetrabutylammonium hexafluorophosphate ($TBAPF_6$, 38.7 mg, 0.10 mmol, 1.0 equiv), which was followed by the injection of 0.40 mL CD_2Cl_2 (0.25 M). The tube was shaken for homogeneity or sonicated if necessary. Then, the solution was submitted to ^{31}P NMR analysis, and the blank spectrum in the absence of HMPA was recorded.

By using the peak of $TBAPF_6$ (δ = -144.4 ppm, hept, 1P) in the deuterated solvent as internal standard, the corresponding amount of HMPA (17.9 to 89.5 mg, 0.10 to 0.50 mmol, 1.0 to 8.0 equiv) was injected inside the NMR tube with a gradient increment of HMPA loading (on 17.9 mg, 0.10 mmol, 1.0 equiv increment basis). After each injection, the ^{31}P NMR was recorded.

Upon completion, the spectra were stacked and shown below.

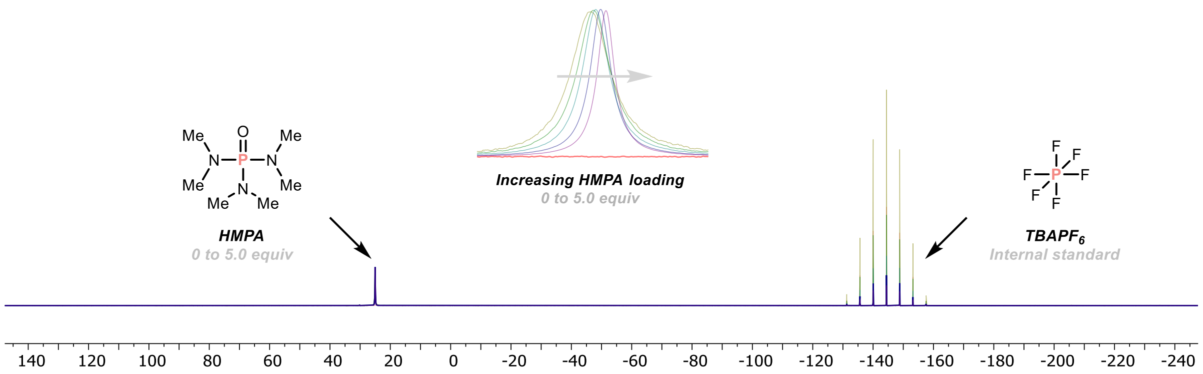

Supplementary Figure 5.5.2.2. Stacked ^{31}P NMR spectra of B_2cat_2 in CD_2Cl_2 with increasing loading of HMPA

CD_2Cl_2 was used in this experiment due to its non-coordinating property and good solubility for B_2cat_2, which is beneficial for signal acquisition in ^{31}P NMR. The change of HMPA ^{31}P signal might indicate the coordination of the oxygen atom of HMPA to B_2cat_2, which altered the chemical environment of the phosphorus centre in HMAP. Besides, the subtle shift might indicate the complexation was transient, or the on-and-off coordinating states were changed rapidly.

Note: $TBAPF_6$ was chosen as the internal standard since it is too weak to coordinate with B_2cat_2. This was confirmed in the blank ^1H and ^{11}B spectra without HMPA, in which the signals of B_2cat_2 remained unchanged.

5.6. Computational mechanistic study

5.6.1. Computational methods

Density functional theory (DFT) calculations were carried out in order to gain insights into the mechanism of the acylboronate formation from intermediates **B1** and **B2**. All calculations were performed using the Gaussian software package (version 16, revision B.01). The dispersion-corrected B3LYP approximation was used as the exchange-correlation functional.[21-23] The 6-31++G(d,p) basis set was employed to represent spin-polarized molecular orbitals. DMA solvent was represented implicitly using the polarizable continuum model in the integral equation formalism.[24] Structures of stable intermediates and transition states were fully optimized. Thermodynamic functions were calculated using the harmonic approximation for the standard-state temperature of 298.15 K and concentration of 1 mol×L^{-1}. The stable intermediate structures and transition states were shown together with their Gibbs free energies.

5.6.2. Results of DFT modelling

5.6.2.1. Formation of the acylboronate intermediate from free carboxylic acid

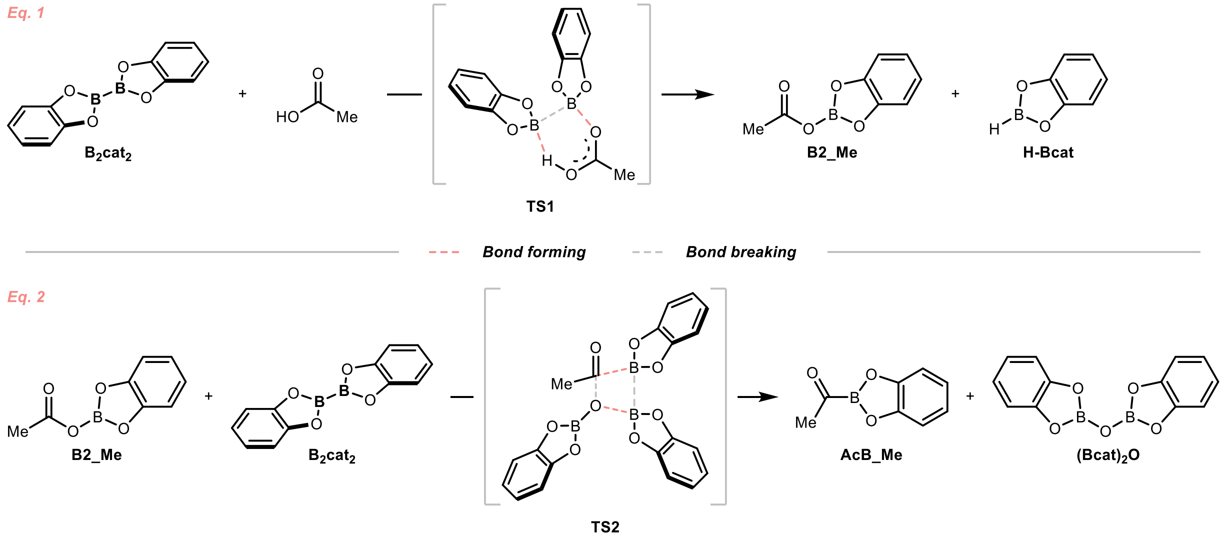

Acetic acid (HOAc) was used as a simple and representative acid to model the formation of acylboronate from free carboxylic acids. The proposed mechanism of the formation of acylboronate intermediate is shown in the equation above. The Gibbs energy profile of the reaction is shown below.

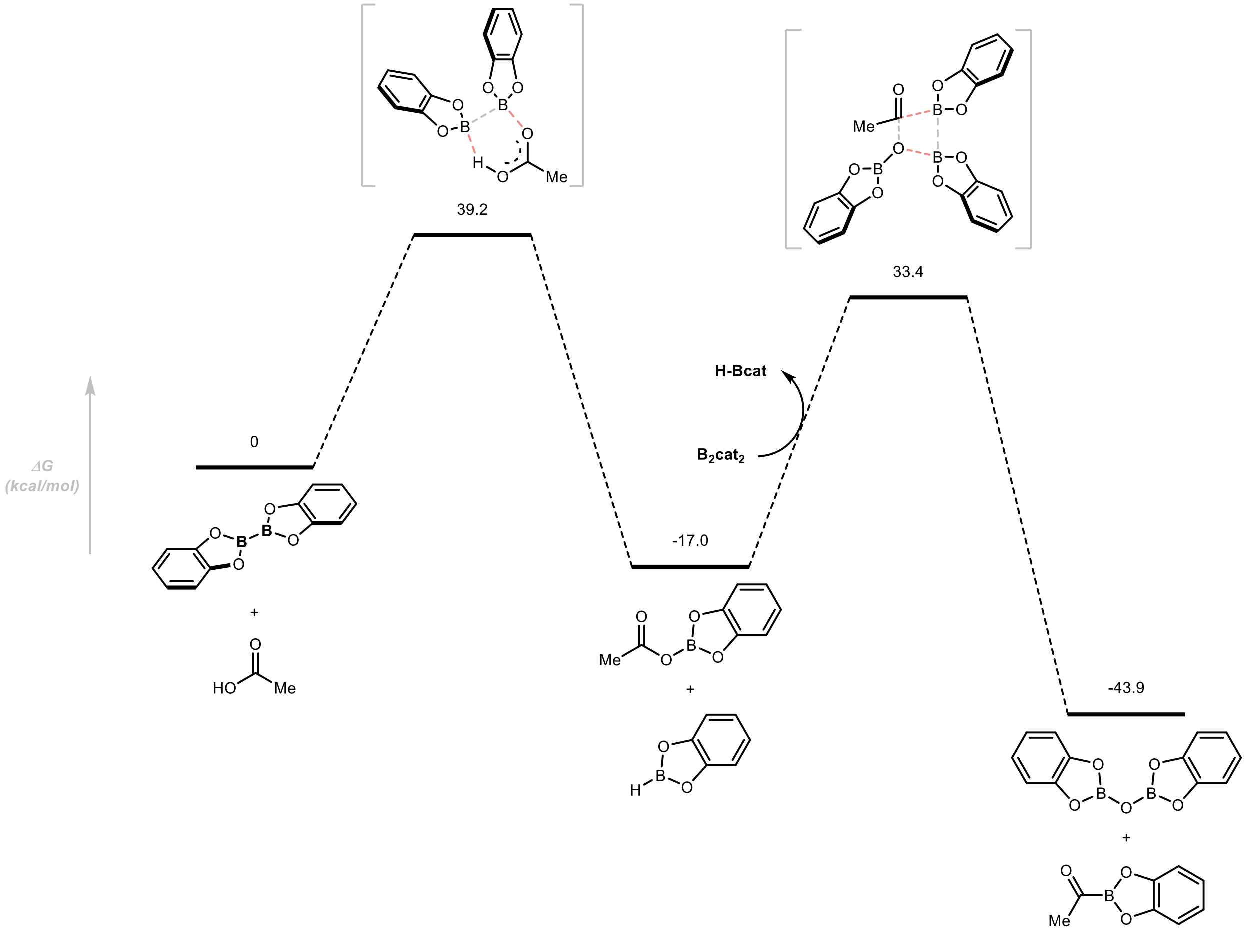

Supplementary Figure 5.6.2.1. Gibbs energy profile for acylboronate formation from acetic acid

5.6.2.2. Formation of the acylboronate intermediate from sodium carboxylate

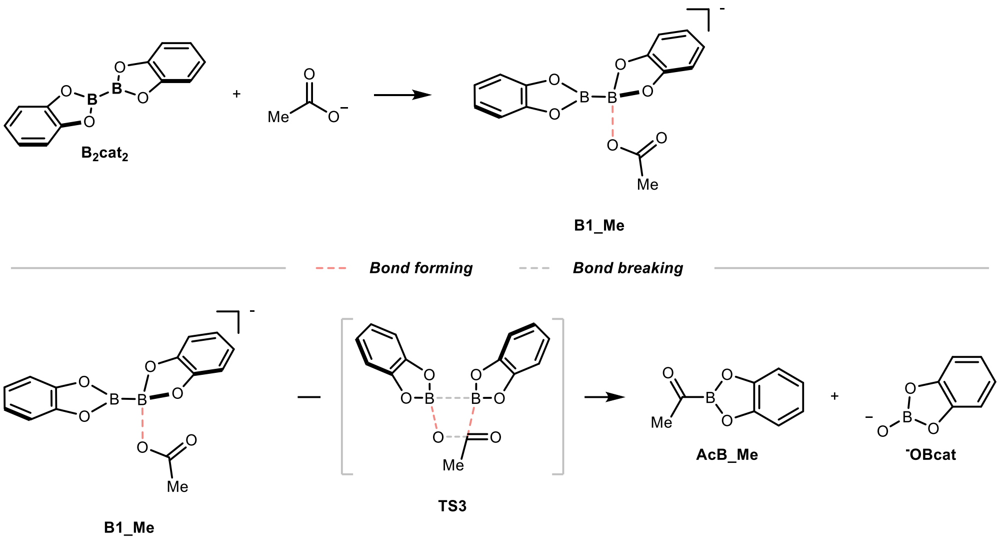

Acetate anion (AcO^-) was used as a simple and representative carboxylate to model the formation of acylboronate from sodium carboxylate. The proposed mechanism of the formation of acylboronate intermediate is shown in the equation above. The Gibbs energy profile of the reaction is shown below.

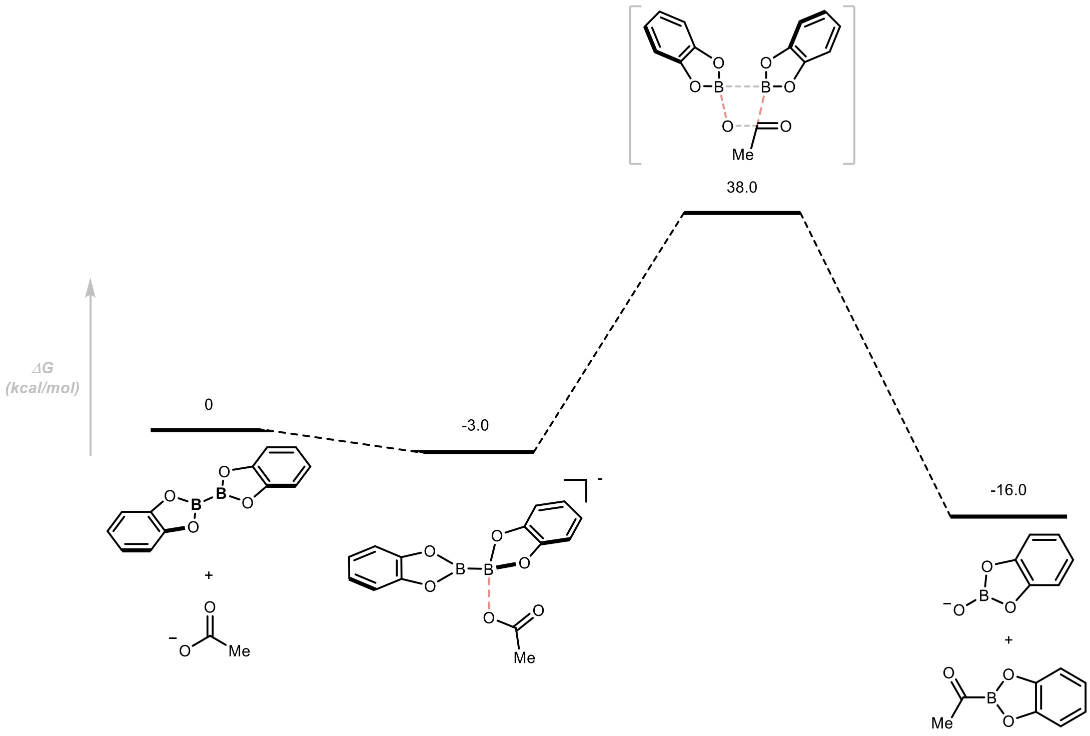

Supplementary Figure 5.6.2.2. Gibbs energy profile for acylboronate formation from acetate anion

5.6.3. Discussion of the DFT results

The calculations showed that all elementary steps are thermodynamically favourable in the cases of both the free acid and carboxylate anion. This indicated that the formation of the boron-oxygen bond is

an effective driving force of the acylboronate formation. The height of the kinetic barriers shows that the transformations are plausible. However, the activation energies appeared to be overestimated. Unlike stable molecules, transition state geometries and barrier heights are difficult to reproduce accurately using the exchange-correlation functionals, B3LYP in this case, that are fitted to experimental data collected for stable systems. Other possible sources of error are the Harmonic treatment of low-frequency vibrations and implicit treatment of solvent molecules. It can be speculated that polar HMPA molecules with negatively charged oxygen atom can form hydrogen or donor-acceptor bonds with the reacting species, which can be stronger in the transition state than reactants, thus lowering the activation energy.

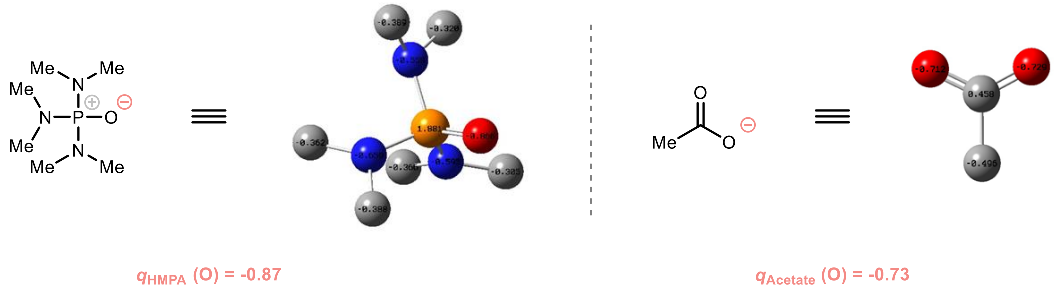

Supplementary Figure 5.6.3. DFT-computed Mulliken charge comparison between HMPA and AcO^- anion

Future more extensive simulations employing the quantum mechanics/molecular mechanics (QM/MM) method to describe solvent molecules explicitly and accelerated molecular dynamics to estimate the free energies beyond harmonic approximation can provide a more accurate description of the reaction and elucidate the nature of the observed solvent effect.

5.7. UV-vis experiments

The UV-vis spectra with benzoic acid (**2a**), sodium benzoate (**2a'**), bis(catecholato)diboron (B_2cat_2), and their mixtures in HMPA were collected on Agilent Cary 5000 series UV-vis-NIR spectrometer.

Note: Sonication was necessary to dissolve the poorly soluble $PhCO_2Na$ and B_2cat_2. HMPA used in these spectroscopic analyses was pre-dried overnight.

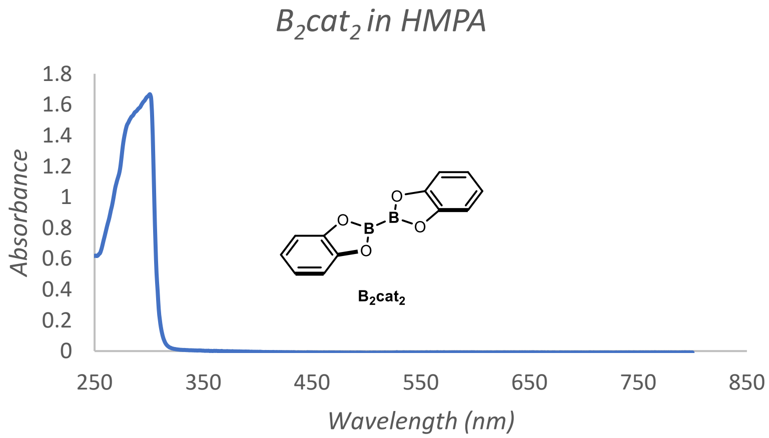

Supplementary Figure 5.7.1. UV-vis spectrum of B_2cat_2 in HMPA

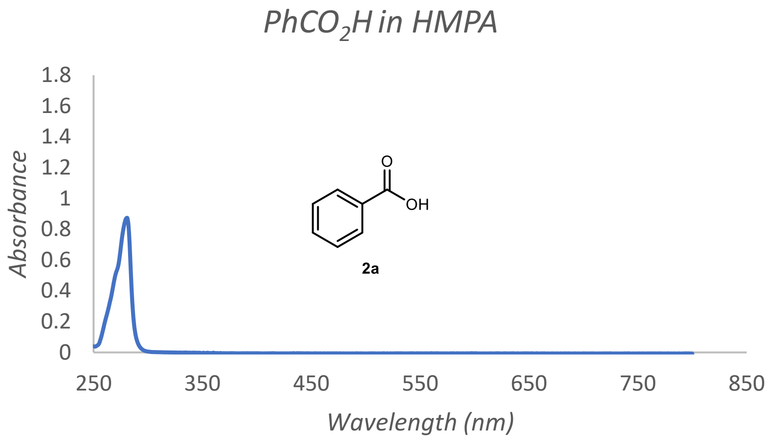

Supplementary Figure 5.7.2. UV-vis spectrum of $PhCO_2H$ in HMPA

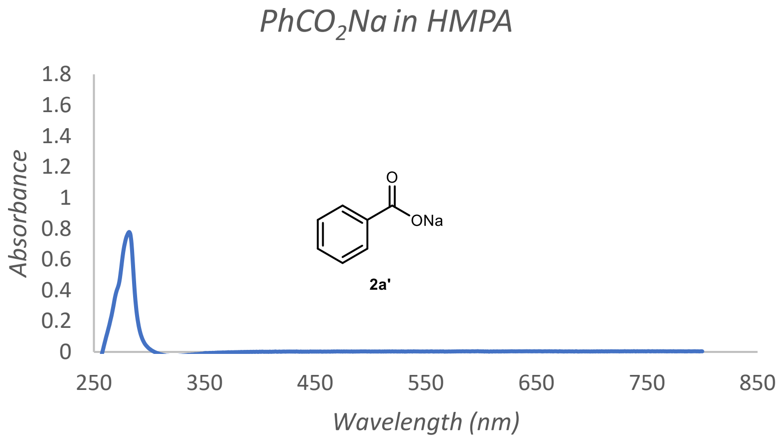

Supplementary Figure 5.7.3. UV-vis spectrum of $PhCO_2Na$ in HMPA

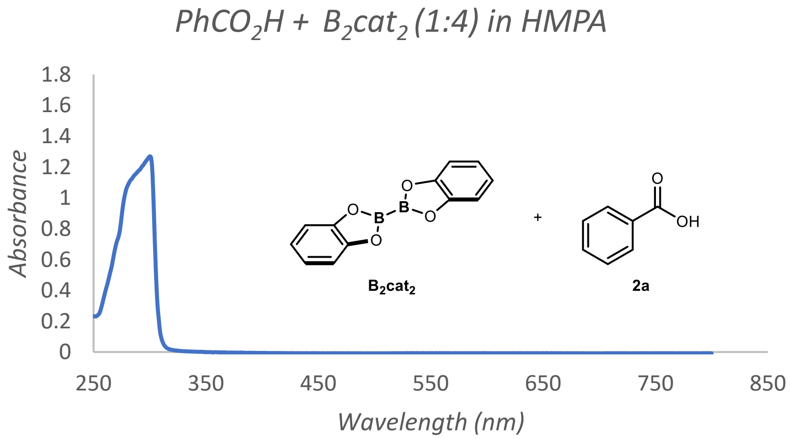

Supplementary Figure 5.7.4. UV-vis spectrum of B_2cat_2 and $PhCO_2H$ (4:1) in HMPA

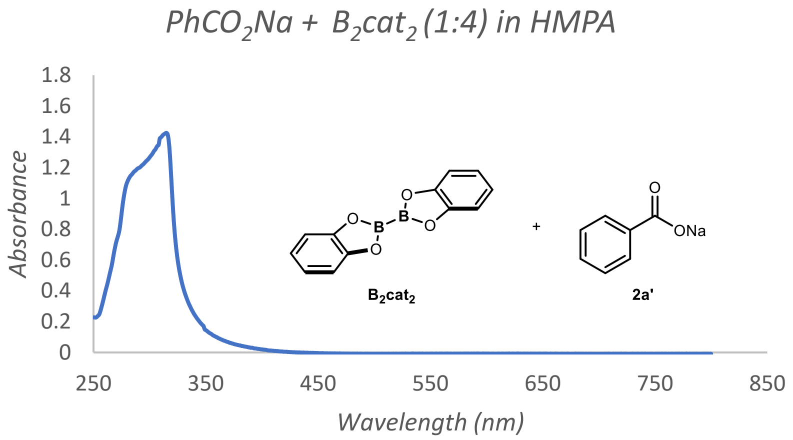

Supplementary Figure 5.7.5. UV-vis spectrum of B_2cat_2 and $PhCO_2Na$ (4:1) in HMPA

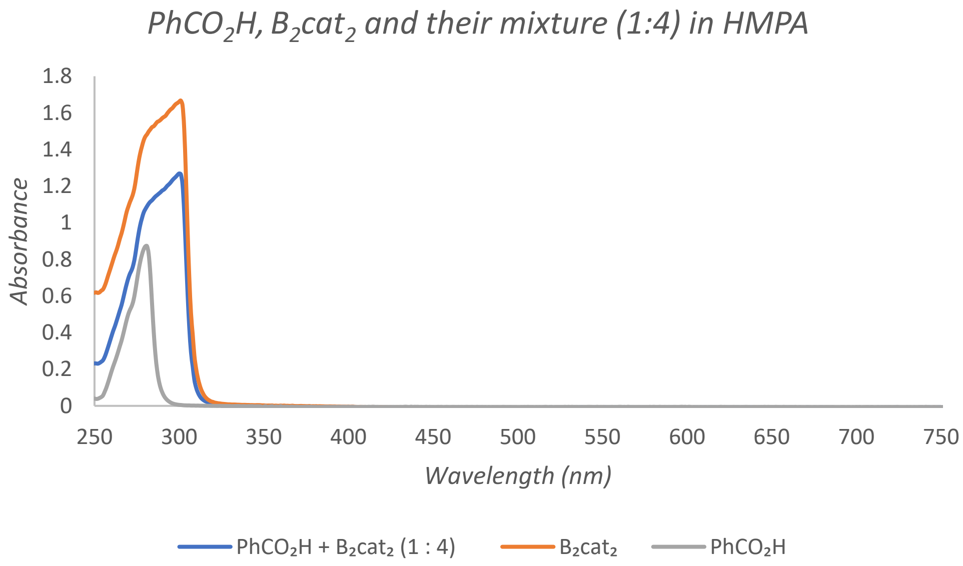

Supplementary Figure 5.7.6. Stacked UV-vis spectra of B_2cat_2, $PhCO_2H$, B_2cat_2 and $PhCO_2H$ (4:1) in HMPA

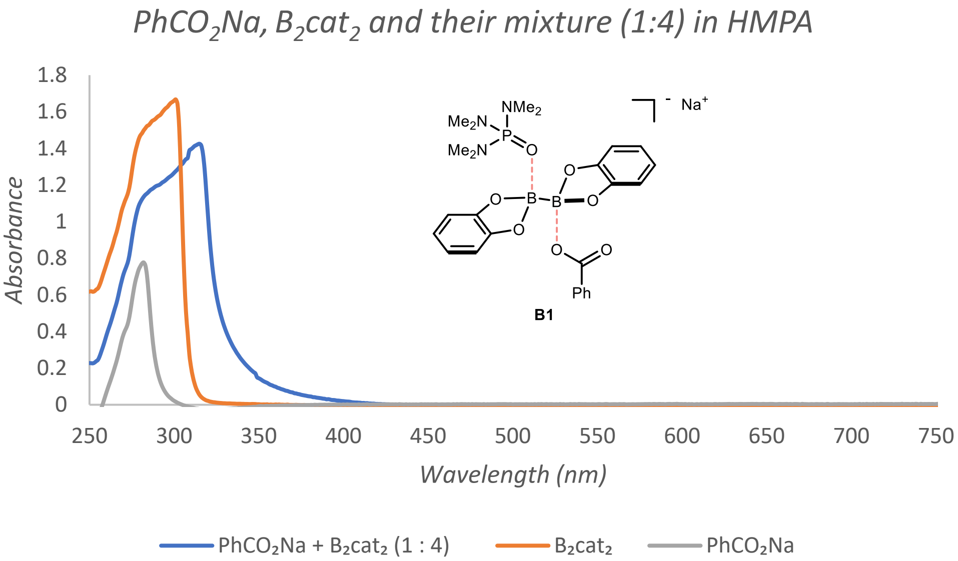

Supplementary Figure 5.7.7. Stacked UV-vis spectra of B_2cat_2, $PhCO_2Na$, B_2cat_2 and $PhCO_2Na$ (4:1) in HMPA

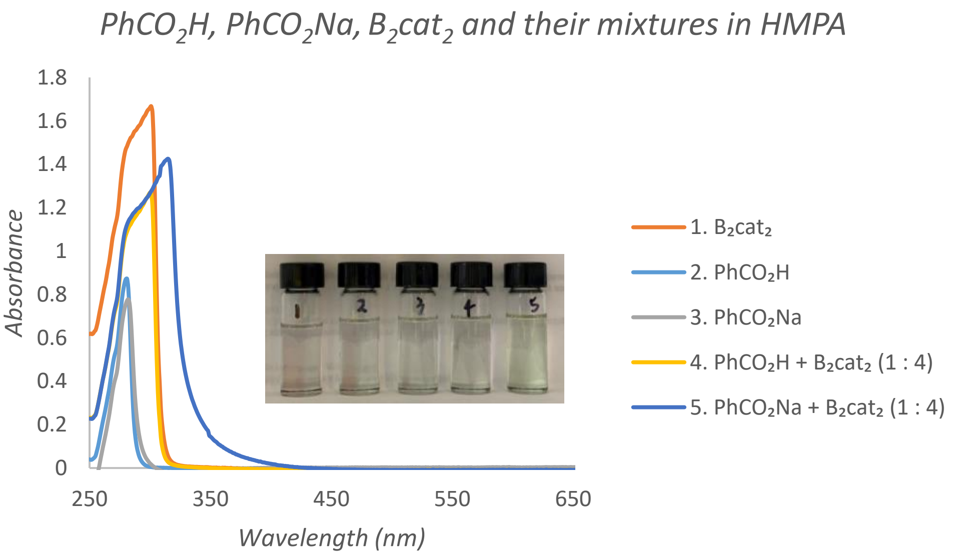

Supplementary Figure 5.7.8. Stacked UV-vis spectra of B_2cat_2, $PhCO_2H$, $PhCO_2Na$, B_2cat_2 and $PhCO_2H$ (4:1), B_2cat_2 and $PhCO_2Na$ (4:1) in HMPA

6. Compound characterization and copies of spectra

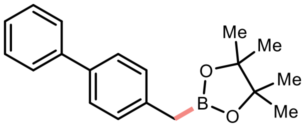

2-([1,1'-Biphenyl]-4-ylmethyl)-4,4,5,5-tetramethyl-1,3,2-dioxaborolane (**1b**, condition A: 56%, 32.9 mg; condition B: 54%, 31.8 mg) was prepared following the general procedure without modification and isolated by column chromatography as a coloerless oil. This compound is known, and its characterization data are consistent with the litrature report.[25]

^{1}H NMR (500 MHz, $CDCl_3$) δ (ppm) 7.62 – 7.60 (m, 2H), 7.51 (d, *J* = 8.2 Hz, 2H), 7.44 (t, *J* = 7.7 Hz, 2H), 7.34 (tt, *J* = 7.4, 1.3 Hz, 1H), 7.29 (d, *J* = 8.2 Hz, 2H), 2.37 (s, 2H), 1.28 (s, 12H);

^{13}C NMR (125 MHz, $CDCl_3$) δ (ppm) 141.3, 137.9, 137.8, 129.4, 128.7, 127.0, 126.9, 126.8, 83.5, 24.8 (*The carbon attached to boron was not observed due to quadrupolar relaxation*);

^{11}B NMR (161 MHz, $CDCl_3$) δ (ppm) 33.2 (bs, 1B);

GC-MS (EI) for $C_{19}H_{23}BO_2$ Calcd: 294.2, found: 294.1.

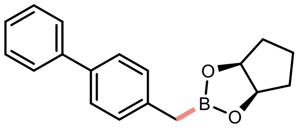

(3a*R*,6a*S*)-2-([1,1'-Biphenyl]-4-ylmethyl)tetrahydro-4*H*-cyclopenta[*d*][1,3,2]dioxaborole (**1f**, condition A: 56%, 31.3 mg) was prepared following the general procedure without modification and worked-up with *cis*-1,2-cyclopentanediol (163.4 mg, 1.6 mmol, 8.0 equiv) in 0.70 mL Et_3N at room temperature for 1.0 hour. It was isolated by column chromatography as a colorless oil. This compound is known, and its characterization data are consistent with the litrature report.[25]

^{1}H NMR (500 MHz, $CDCl_3$) δ (ppm) 7.62 – 7.60 (m, 2H), 7.53 (d, *J* = 8.2 Hz, 2H), 7.45 (t, *J* = 7.7 Hz, 2H), 7.35 (t, *J* = 7.4 Hz, 1H), 7.30 (d, *J* = 8.2 Hz, 2H), 4.88 (dd, *J* = 3.9, 1.7 Hz, 2H), 2.41 (s, 2H), 1.97 – 1.94 (m, 2H), 1.68 – 1.54 (m, 4H);

^{13}C NMR (125 MHz, $CDCl_3$) δ (ppm) 141.3, 137.9, 137.9, 129.3, 128.7, 127.1, 127.0, 126.9, 82.5, 34.6, 21.5 (*The carbon attached to boron was not observed due to quadrupolar relaxation*);

^{11}B NMR (161 MHz, $CDCl_3$) δ (ppm) 33.2 (bs, 1B);

GC-MS (EI) for $C_{18}H_{19}BO_2$ Calcd: 278.1, fond: 278.1.

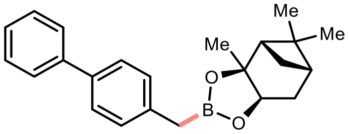

(3a*S*,4*S*,6*S*,7a*R*)-2-([1,1'-Biphenyl]-4-ylmethyl)-3a,5,5-trimethylhexahydro-4,6-methanobenzo [d][1,3,2]dioxaborole (**1g**, condition A: 49%, 34.0 mg) was prepared following the general procedure without modification and worked-up with (1*R*,2*R*,3*S*,5*R*)-(−)-pinanediol (272.4 mg, 1.6 mmol, 8.0 equiv) in 0.70 mL Et_3N at room temperature for 1.0 hour. This compound is known, and its characterization data are consistent with the litrature report.[25]

^{1}H NMR (500 MHz, $CDCl_3$) δ (ppm) 7.63 – 7.61 (m, 2H), 7.53 (d, *J* = 8.2 Hz, 2H), 7.45 (t, *J* = 7.7 Hz, 2H), 7.36 – 7.33 (m, 1H), 7.32 (d, *J* = 8.1 Hz, 2H), 4.34 (dd, *J* = 8.8, 2.0 Hz, 1H), 2.43 (s, 2H), 2.39 – 2.34 (m, 1H), 2.27

– 2.20 (m, 1H), 2.11 (t, J = 5.5 Hz, 1H), 1.96 – 1.92 (m, 1H), 1.91 – 1.87 (m, 1H), 1.44 (s, 3H), 1.32 (s, 3H), 1.14 (d, J = 10.9 Hz, 1H), 0.88 (s, 3H);
^{13}C NMR (125 MHz, $CDCl_3$) δ (ppm) 141.3, 138.0, 137.8, 129.4, 128.7, 127.1, 127.0, 126.9, 85.9, 78.0, 51.3, 39.5, 38.2, 35.5, 28.7, 27.1, 26.5, 24.0 (*The carbon attached to boron was not observed due to quadrupolar relaxation*);
^{11}B NMR (161 MHz, $CDCl_3$) δ (ppm) 32.7 (bs, 1B);
GC-MS (EI) for $C_{23}H_{27}BO_2$ Calcd: 346.2, found: 346.2.

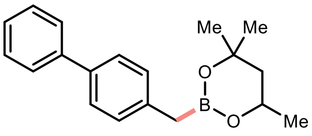

2-([1,1'-Biphenyl]-4-ylmethyl)-4,4,6-trimethyl-1,3,2-dioxaborinane (**1h**, condition A: 45%, 26.5 mg) was prepared following the general procedure without modification and worked-up with (±)-2-methyl-2,4-pentanediol (189.1 mg, 1.6 mmol, 8.0 equiv) in 0.70 mL Et_3N at room temperature for 1.0 hour. This compound is known, and its characterization data are consistent with the litrature report.[25]
^{1}H NMR (500 MHz, $CDCl_3$) δ 7.65 – 7.62 (m, 2H), 7.52 (d, J = 8.2 HZz, 2H), 7.46 (t, J = 7.7 Hz, 2H), 7.35 (tt, J = 7.2, 1.2 Hz, 1H), 7.31 (d, J = 8.3 Hz, 2H), 4.23 (dqd, J = 12.3, 6.2, 3.0 Hz, 1H), 2.26 (s, 2H), 1.81 (dd, J = 13.9, 3.0 Hz, 1H), 1.52 (dd, J = 13.6, 11.9 Hz, 1H), 1.33 (s, 3H), 1.32 (s, 3H), 1.28 (d, J = 6.2 Hz, 3H);
^{13}C NMR (125 MHz, $CDCl_3$) δ 141.5, 139.6, 137.2, 129.5, 128.7, 126.9, 126.7, 70.9, 64.9, 45.9, 31.2, 28.1, 23.2 (*The carbon attached to boron was not observed due to quadrupolar relaxation and one aromatic carbon was missing due to the overlapping*);
^{11}B NMR (161 MHz, $CDCl_3$) δ 29.4 (bs, 1B);
GC-MS (EI) for $C_{19}H_{23}BO_2$ Calcd: 294.2, found: 294.1.

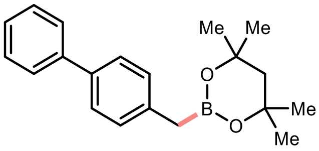

2-([1,1'-Biphenyl]-4-ylmethyl)-4,4,6,6-tetramethyl-1,3,2-dioxaborinane (**1i**, condition B: 59%, 36.3 mg) was prepared following the general procedure without modification and worked-up with 2,4-dimethyl-2,4-pentanediol (211.5 mg, 1.6 mmol, 8.0 equiv) in 0.70 mL Et_3N at room temperature for 1.0 hour. This compound is known, and its characterization data are consistent with the litrature report.[25]
^{1}H NMR (500 MHz, $CDCl_3$) δ 7.64 – 7.62 (m, 2H), 7.51 (d, J = 8.2 Hz, 2H), 7.45 (t, J = 7.7 Hz, 2H), 7.35 – 7.30 (m, 3H), 2.25 (s, 2H), 1.84 (s, 2H), 1.36 (s, 12H);
^{13}C NMR (125 MHz, $CDCl_3$) δ 141.5, 139.8, 137.1, 129.5, 128.7, 126.9, 126.7, 126.7, 70.7, 48.8, 31.8 (*The carbon attached to boron was not observed due to quadrupolar relaxation*);
^{11}B NMR (161 MHz, $CDCl_3$) δ 29.2 (bs, 1B);
GC-MS (EI) for $C_{20}H_{25}BO_2$ Calcd: 308.2, found: 308.2.

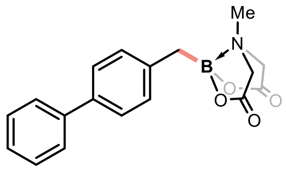

2-([1,1'-Biphenyl]-4-ylmethyl)-6-methyl-1,3,6,2-dioxazaborocane-4,8-dione (**1j**, condition A: 50%, 32.3 mg) was prepared following the general procedure without modification and worked-up with *N*-methyliminodiacetic acid (235.4 mg, 1.6 mmol, 8.0 equiv) at 90 °C for 4.0 hour. This compound is known, and its characterization data are consistent with the litrature report.[25]

^{1}H NMR (500 MHz, CD_3CN) δ (ppm) 7.67 – 7.64 (m, 2H), 7.55 (d, *J* = 8.2 Hz, 2H), 7.47 (t, *J* = 7.7 Hz, 2H), 7.36 (t, *J* = 7.4 Hz, 1H), 7.30 (d, *J* = 8.2 Hz, 2H), 3.98 (d, *J* = 16.9 Hz, 2H), 3.78 (d, *J* = 16.9 Hz, 2H), 2.95 (s, 3H), 2.19 (s, 2H);

^{13}C NMR (125 MHz, CD_3CN) δ (ppm) 168.0, 141.0, 139.9, 137.2, 129.7, 128.8, 127.0, 126.6, 126.6, 62.0, 45.8 (*The carbon attached to boron was not observed due to quadrupolar relaxation*);

^{11}B NMR (160 MHz, CD_3CN) (ppm) 12.3 (bs, 1B);

HRMS (ESI, M+Na^{+}) for $C_{18}H_{18}BNNaO_4$ Calcd: 346.1221, found: 346.1230.

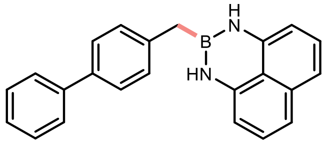

2-([1,1'-Biphenyl]-4-ylmethyl)-2,3-dihydro-1*H*-naphtho[1,8-*de*][1,3,2]diazaborinine (**1k**, condition A: 46%, 30.7 mg) was prepared following the general procedure without modification and worked-up with potassium carbonate (K_2CO_3, 442.3 mg, 3.2 mmol, 16.0 equiv) and 1,8-diaminonaphthalene (253.1 mg, 1.6 mmol, 8.0 equiv) in 4.0 mL CH_3CN (0.050 M) at room temperature for 1.0 hour. This compound is unknonw in the literature.

^{1}H NMR (500 MHz, $CDCl_3$) δ (ppm) 7.66 (d, *J* = 7.9 Hz, 2H), 7.60 (d, *J* = 8.1 Hz, 2H), 7.50 (t, *J* = 7.7 Hz, 2H), 7.39 (t, *J* = 7.3 Hz, 1H), 7.28 (d, *J* = 7.9 Hz, 2H), 7.13 (t, *J* = 7.8 Hz, 2H), 7.06 (d, *J* = 8.3 Hz, 2H), 6.30 (dd, *J* = 7.3, 1.1 Hz, 2H), 5.56 (s, 2H), 2.47 (s, 2H);

^{13}C NMR (125 MHz, $CDCl_3$) δ (ppm) 141.0, 140.9, 138.7, 138.2, 136.3, 129.3, 128.8, 127.6, 127.5, 127.1, 127.0, 119.6, 117.7, 105.8 (*The carbon attached to boron was not observed due to quadrupolar relaxation*);

^{11}B NMR (161 MHz, $CDCl_3$) δ (ppm) 31.6 (bs, 1B);

GC-MS (EI) for $C_{23}H_{19}BN_2$ Calcd: 334.2, found: 334.2;

HRMS (APCI, M+H^{+}) for $C_{23}H_{20}BN_2$ Calcd: 335.1714, found: 335.1710.

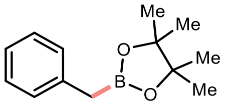

2-Benzyl-4,4,5,5-tetramethyl-1,3,2-dioxaborolane (**2b**, condition B: 65%, 28.3 mg) was prepared following the general procedure without modification and isolated by column chromatography as a coloerless oil. This compound is known, and its characterization data are consistent with the litrature report.[25]

^{1}H NMR (500 MHz, $CDCl_3$) δ (ppm) 7.26 (t, *J* = 7.5 Hz, 2H), 7.21 (d, *J* = 6.6 Hz, 2H), 7.14 (t, *J* = 7.2 Hz, 1H), 2.32 (s, 2H), 1.26 (s, 12H);
^{13}C NMR (125 MHz, $CDCl_3$) δ (ppm) 138.6, 129.0, 128.3, 124.8, 83.4, 24.7 (*The carbon attached to boron was not observed due to quadrupolar relaxation*);
^{11}B NMR (161 MHz, $CDCl_3$) δ (ppm) 33.1 (bs, 1B);
GC-MS (EI) for $C_{13}H_{19}BO_2$ Calcd: 218.1, found: 218.1.

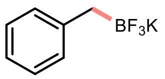

Benzyltrifluoro-λ^4-borane, potassium salt (**2l**) was prepared from the corresponding benzylboronic pinacol ester via the reaction with saturated KHF_2 aqueous solution and recrystallization in acetone and Et_2O.[10] This compound is known, and its characterization data are consistent with the literature report.[26]
^{1}H NMR (500 MHz, DMSO-d_6) δ 7.04 (t, *J* = 7.4 Hz, 2H), 6.98 (d, *J* = 7.5 Hz, 2H), 6.87 (t, *J* = 7.2 Hz, 1H), 1.45 (s, 2H);
^{13}C NMR (125 MHz, DMSO-d_6) δ 147.4, 129.1, 127.4, 122.4 (*The carbon attached to boron was not observed due to quadrupolar relaxation*);
^{11}B NMR (161 MHz, DMSO-d_6) δ 3.9 (broad q, *J* = 53.7 Hz, 1B);
^{19}F NMR (471 MHz, DMSO-d_6) δ 136.79 (bs, 3F);
HRMS (ESI, M+K$^+$) for $C_7H_7BF_3K_2$ Calcd: 236.9862, found: 236.9863.

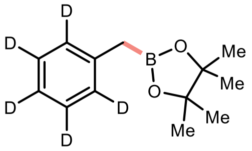

4,4,5,5-Tetramethyl-2-((phenyl-*d*$_5$)methyl)-1,3,2-dioxaborolane (**2b-*d***, condition B: 51%, 22.7 mg) was prepared following the general procedure without modification and isolated by column chromatography as a colourless oil. This compound is unknown in the literature.
^{1}H NMR (800 MHz, acetone-d$_6$) δ 2.23 (s, 2H), 1.22 (s, 12H);
^{13}C NMR (201 MHz, acetone-d_6) δ 138.8, 128.5 (t, *J* = 24.1 Hz), 127.5 (t, *J* = 24.1 Hz), 124.1 (t, *J* = 24.2 Hz), 83.1, 24.1 (*The carbon attached to boron was not observed due to quadrupolar relaxation*);
^{2}H NMR (123 MHz, acetone-d_6) δ 7.23 (d, *J* = 0.8 Hz, 2D), 7.18 (d, *J* = 0.8 Hz, 2D), 7.11 (d, *J* = 0.7Hz, 1D);
^{11}B NMR (161 MHz, acetone-d_6) δ 33.0 (bs, 1B);
GC-MS (EI) for $C_{13}H_{14}D_5BO_2$ Calcd: 223.2, found: 223.1;
HRMS (APCI, M+H$^+$) for $C_{13}H_{15}BD_5O_2$ Calcd: 224.1865, found: 224.1863.

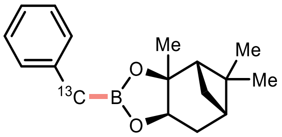

(3a*S*,4*S*,6*S*,7a*R*)-3a,5,5-trimethyl-2-(phenyl-λ^2-methyl-^{13}*C*)hexahydro-4,6-methanobenzo[*d*][1,3,2]dioxaborole (**2g-^{13}C**, condition B: 58%, 31.4 mg) was prepared following the

general procedure without modification and isolated by column chromatography as a coloerless oil. This compound is unknown in the literature.

^{1}H NMR (500 MHz, $CDCl_3$) δ 7.26 (d, *J* = 7.4 Hz, 2H), 7.24 – 7.21 (m, 2H), 7.15 (t, *J* = 7.1 Hz, 1H), 4.30 (dd, *J* = 8.8, 1.9 Hz, 1H), 2.36 (d, *J* = 118.0 Hz, 2H), 2.36 – 2.30 (m, 1H), 2.23 – 2.18 (m, 1H), 2.07 (dd, *J* = 5.6, 5.6 Hz, 1H), 1.93 – 1.89 (m, 1H), 1.87 – 1.Z83 (m, 1H), 1.41 (s, 3H), 1.30 (s, 3H), 1.09 (d, *J* = 10.9 Hz, 1H), 0.85 (s, 3H);

^{13}C NMR (125 MHz, $CDCl_3$) δ 143.4, 142.9, 142.3, 141.4, 140.9, 139.0, 138.6, 129.0, 128.9, 128.3, 128.3, 124.9, 85.8, 51.3, 39.5, 38.9, 38.8, 38.7, 38.6, 38.2, 35.5, 28.6, 27.1, 26.4, 24.0, 19.7 (bs) (*Additional peaks observed probably indicated the presence of rotamers*);

^{11}B NMR (161 MHz, $CDCl_3$) δ 32.6 (bs, 1B);

GC-MS (EI) for $C_{16}{}^{13}C_1H_{23}BO_2$ Calcd: 271.2, *not found possibly due to the instability or non-volatility of target compound;*

HRMS (APCI, M+H$^+$) for $C_{16}{}^{13}C_1H_{24}BO_2$ Calcd: 272.1897, found: 272.1893.

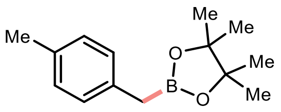

4,4,5,5-Tetramethyl-2-(4-methylbenzyl)-1,3,2-dioxaborolane (**3b**, condition B: 62%, 28.8 mg) was prepared following the general procedure without modification and isolated by column chromatography as a coloerless oil. This compound is known, and its characterization data are consistent with the litrature report.[25]

^{1}H NMR (500 MHz, $CDCl_3$) δ (ppm) 7.10 (d, *J* = 8.1 Hz, 2H), 7.07 (d, *J* = 8.1 Hz, 2H), 2.32 (s, 3H), 2.28 (s, 2H), 1.26 (s, 12H);

^{13}C NMR (125 MHz, $CDCl_3$) δ (ppm) 135.4, 134.1, 129.0, 128.9, 83.4, 24.7, 21.0 (*The carbon attached to boron was not observed due to quadrupolar relaxation*);

^{11}B NMR (161 MHz, $CDCl_3$) δ (ppm) 33.2 (bs, 1B);

GC-MS (EI) for $C_{14}H_{21}BO_2$ Calcd: 232.2, found: 232.2.

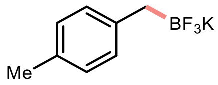

Trifluoro(4-methylbenzyl)- λ^4-borane, potassium salt (**3l**) was prepared from the corresponding benzylboronic pinacol ester via the reaction with saturated KHF_2 aqueous solution and recrystallization in acetone and Et_2O.[10] This compound is known, and its characterization data are consistent with the literature report.[27]

^{1}H NMR (500 MHz, acetone-*d*$_6$) δ 7.00 (d, *J* = 7.7 Hz, 2H), 6.87 (d, *J* = 7.8 Hz, 2H), 2.20 (s, 3H), 1.60 (s, 2H);

^{13}C NMR (125 MHz, acetone-*d*$_6$) δ (ppm) 143.9, 130.5, 128.7, 127.6, 20.1 (*The carbon attached to boron was not observed due to quadrupolar relaxation*);

^{11}B NMR (161 MHz, acetone-*d*$_6$) δ 4.4 (q, *J* = 61.1 Hz, 1B);

^{19}F NMR (471 MHz, acetone-*d*$_6$) δ -140.26 – -141.19 (m, 3F);

HRMS (ESI, M-K$^+$) for $C_8H_9BF_3$ Calcd: 173.0755, found: 173.0751.

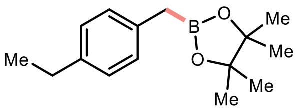

2-(4-Ethylbenzyl)-4,4,5,5-tetramethyl-1,3,2-dioxaborolane (**4b**, condition B: 65%, 32.0 mg) was prepared following the general procedure without modification and isolated by column chromatography as a coloerless oil. This compound is known, and its characterization data are consistent with the litrature report.[25]

^{1}H NMR (500 MHz, $CDCl_3$) δ (ppm) 7.13 (d, *J* = 8.1 Hz, 2H), 7.09 (d, *J* = 8.1 Hz, 2H), 2.62 (q, *J* = 7.5 Hz, 2H), 2.28 (s, 2H), 1.26 (s, 12H), 1.23 (d, *J* = 7.6 Hz, 3H);

^{13}C NMR (125 MHz, $CDCl_3$) δ (ppm) 140.6, 135.6, 128.9, 127.8, 83.4, 28.4, 24.7, 15.6 (*The carbon attached to boron was not observed due to quadrupolar relaxation*);

^{11}B NMR (161 MHz, $CDCl_3$) δ (ppm) 33.2 (bs, 1B);

GC-MS (EI) for $C_{15}H_{23}BO_2$ Calcd: 246.2, found: 246.1.

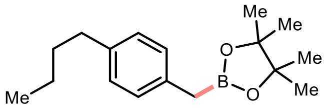

2-(4-Butylbenzyl)-4,4,5,5-tetramethyl-1,3,2-dioxaborolane (**5b**, condition B: 56%, 30.7 mg) was prepared following the general procedure without modification and isolated by column chromatography as a colourless oil. This compound is unknown in the literature.

^{1}H NMR (500 MHz, $CDCl_3$) δ 7.11 (d, *J* = 8.1 Hz, 2H), 7.07 (d, *J* = 8.1 Hz, 2H), 2.58 (t, *J* = 7.8 Hz, 2H), 2.28 (s, 2H), 1.63 – 1.56 (m, 2H), 1.41 – 1.33 (m, 2H), 1.26 (s, 12H), 0.94 (t, *J* = 7.4 Hz, 3H);

^{13}C NMR (125 MHz, $CDCl_3$) δ 139.2, 135.6, 128.8, 128.3, 83.4, 35.2, 33.7, 24.7, 22.4, 14.0 (*The carbon attached to boron was not observed due to quadrupolar relaxation*);

^{11}B NMR (161 MHz, $CDCl_3$) δ 33.2 (bs, 1B);

GC-MS (EI) for $C_{17}H_{27}BO_2$ Calcd: 274.2, found: 274.1.

HRMS (APCI, M+H$^+$) for $C_{17}H_{28}BO_2$ Calcd: 275.2177, found: 275.2175;

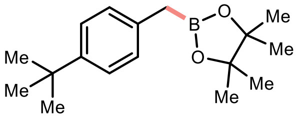

2-(4-(*Tert*-butyl)benzyl)-4,4,5,5-tetramethyl-1,3,2-dioxaborolane (**6b**, condition A: 55%, 30.1 mg) was prepared following the general procedure without modification and isolated by column chromatography as a coloerless oil. This compound is known, and its characterization data are consistent with the litrature report.[25]

^{1}H NMR (500 MHz, $CDCl_3$) δ 7.29 (d, *J* = 8.3 Hz, 2H), 7.15 (d, *J* = 8.4 Hz, 2H), 2.29 (s, 2H), 1.33 (s, 9H), 1.27 (s, 12H);

^{13}C NMR (125 MHz, $CDCl_3$) δ 147.5, 135.4, 128.7, 125.2, 83.4, 34.3, 31.4, 24.8 (*The carbon attached to boron was not observed due to quadrupolar relaxation*);

^{11}B NMR (161 MHz, $CDCl_3$) δ 33.2 (bs, 1B);

GC-MS (EI) for $C_{17}H_{27}BO_2$ Calcd: 274.2, found: 274.1.

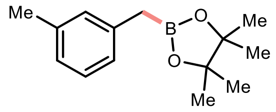

4,4,5,5-Tetramethyl-2-(3-methylbenzyl)-1,3,2-dioxaborolane (**7b**, condition B: 71%, 32.9 mg) was prepared following the general procedure without modification and isolated by column chromatography as a coloerless oil. This compound is known, and its characterization data are consistent with the litrature report.[25]

^{1}H NMR (500 MHz, $CDCl_3$) δ (ppm) 7.15 (t, *J* = 7.5 Hz, 1H), 7.02 – 7.00 (m, 2H), 6.96 (d, *J* = 7.5 Hz, 1H), 2.33 (s, 3H), 2.28 (s, 2H), 1.26 (s, 12H);

^{13}C NMR (125 MHz, $CDCl_3$) δ (ppm) 138.5, 137.7, 129.9, 128.1, 126.0, 125.6, 83.4, 24.7, 21.4 (*The carbon attached to boron was not observed due to quadrupolar relaxation*);

^{11}B NMR (161 MHz, $CDCl_3$) δ (ppm) 33.1 (bs, 1B);

GC-MS (EI) for $C_{14}H_{21}O_2$ Calcd: 232.1, found: 232.1.

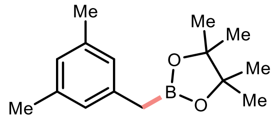

2-(3,5-Dimethylbenzyl)-4,4,5,5-tetramethyl-1,3,2-dioxaborolane (**8b**, condition A: 45%, 22.1 mg) was prepared following the general procedure without modification and isolated by column chromatography as a colourless oil. This compound is known, and its characterization data are consistent with the literature report.[25]

^{1}H NMR (500 MHz, $CDCl_3$) δ 6.82 (s, 2H), 6.78 (s, 1H), 2.29 (s, 6H), 2.24 (s, 2H), 1.26 (s, 12H);

^{13}C NMR (125 MHz, $CDCl_3$) δ 138.4, 137.6, 126.9, 126.6, 83.4, 24.7, 21.3 (*The carbon attached to boron was not observed due to quadrupolar relaxation*);

^{11}B NMR (161 MHz, $CDCl_3$) δ 33.2 (bs, 1B);

GC-MS (EI) for $C_{15}H_{23}BO_2$ Calcd: 246.2, found: 246.1;

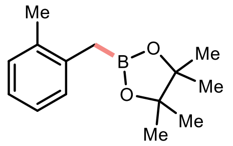

4,4,5,5-Tetramethyl-2-(2-methylbenzyl)-1,3,2-dioxaborolane (**9b**, condition B: 47%, 21.8 mg) was prepared following the general procedure without modification and isolated by column chromatography as a coloerless oil. This compound is known, and its characterization data are consistent with the litrature report.[28]

^{1}H NMR (500 MHz, $CDCl_3$) δ 7.18 – 7.06 (m, 4H), 2.31 (s, 3H), 2.29 (s, 2H), 1.26 (s, 12H);

^{13}C NMR (125 MHz, $CDCl_3$) δ 137.5, 135.9, 129.8, 129.5, 125.9, 125.2, 83.4, 24.8, 20.1 (*The carbon attached to boron was not observed due to quadrupolar relaxation*);

^{11}B NMR (161 MHz, $CDCl_3$) δ 33.2 (bs, 1B);

GC-MS (EI) for $C_{14}H_{21}O_2$ Calcd: 232.1, found: 232.1.

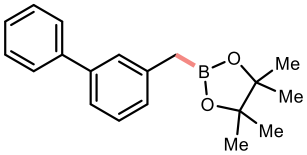

2-([1,1'-Biphenyl]-3-ylmethyl)-4,4,5,5-tetramethyl-1,3,2-dioxaborolane (**10b**, condition B: 53%, 31.2 mg) was prepared following the general procedure without modification and isolated by column chromatography as a coloerless oil. This compound is known, and its characterization data are consistent with the litrature report.[25]

^{1}H NMR (500 MHz, $CDCl_3$) δ 7.63 – 7.61 (m, 2H), 7.47 – 7.44 (m, 3H), 7.39 (tt, *J* = 7.6, 1.4 Hz, 1H), 7.36-7.33 (m, 2H), 7.21 (d, *J* = 7.6 Hz, 1H), 2.40 (s, 2H), 1.28 (s, 12H);

^{13}C NMR (125 MHz, $CDCl_3$) δ 141.5, 141.1, 139.1, 128.7, 128.6, 128.0, 127.9, 127.2, 127.0, 123.8, 83.5, 24.8 (*The carbon attached to boron was not observed due to quadrupolar relaxation*);

^{11}B NMR (161 MHz, $CDCl_3$) δ 33.2 (bs, 1B);

GC-MS (EI) for $C_{19}H_{23}BO_2$ Calcd: 294.2, found: 294.1.

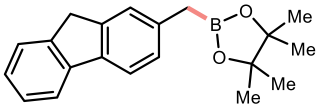

2-((9*H*-Fluoren-2-yl)methyl)-4,4,5,5-tetramethyl-1,3,2-dioxaborolane (**11b**, condition A: 56%, 34.3 mg) was prepared following the general procedure without modification and isolated by column chromatography as a coloerless oil. This compound is known, and its characterization data are consistent with the litrature report.[25]

^{1}H NMR (500 MHz, $CDCl_3$) δ 7.77 (d, *J* = 7.5 Hz, 1H), 7.71 (d, *J* = 7.8 Hz, 1H), 7.55 (d, *J* = 7.4 Hz, 1H), 7.42 (s, 1H), 7.38 (t, *J* = 7.2 Hz, 1H), 7.29 (td, *J* = 7.4, 1.1 Hz, 1H), 7.25 (d, *J* = 7.8 Hz, 1H), 3.90 (s, 2H), 2.43 (s, 2H), 1.29 (s, 12H);

^{13}C NMR (125 MHz, $CDCl_3$) δ 143.6, 143.1, 142.0, 138.7, 137.4, 127.5, 126.6, 126.1, 125.7, 124.9, 119.7, 119.5, 83.5, 36.8, 24.8 (*The carbon attached to boron was not observed due to quadrupolar relaxation*);

^{11}B NMR (161 MHz, $CDCl_3$) δ 33.2 (bs, 1B);

GC-MS (EI) for $C_{20}H_{23}BO_2$ Calcd: 306.2, found: 306.1.

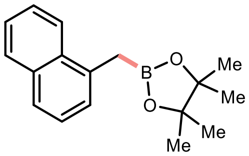

4,4,5,5-Tetramethyl-2-(naphthalen-1-ylmethyl)-1,3,2-dioxaborolane (**12b**, condition B: 44%, 23.6 mg) was prepared following the general procedure without modification and isolated by column chromatography as a coloerless oil. This compound is known, and its characterization data are consistent with the litrature report.[28]

^{1}H NMR (500 MHz, $CDCl_3$) δ (ppm) 8.04 (d, *J* = 8.3 Hz, 1H), 7.86 – 7.84 (m, 1H), 7.69 (d, *J* = 7.8 Hz, 1H), 7.52 – 7.46 (m, 2H), 7.41 – 7.36 (m, 2H), 2.72 (s, 2H), 1.22 (s, 12H);

^{13}C NMR (125 MHz, $CDCl_3$) δ (ppm) 135.6, 133.8, 132.5, 128.5, 126.5, 125.8, 125.4, 125.3, 124.5, 83.5, 24.7 (*The carbon attached to boron was not observed due to quadrupolar relaxation and one aromatic carbon was missing due to the overlapping*);
^{11}B NMR (161 MHz, $CDCl_3$) δ (ppm) 33.5 (bs, 1B);
GC-MS (EI) for $C_{17}H_{21}BO_2$ Calcd: 268.2 found: 268.1;

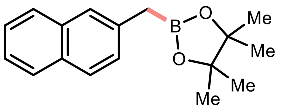

4,4,5,5-Tetramethyl-2-(naphthalen-2-ylmethyl)-1,3,2-dioxaborolane (**13b**, condition A: 58%, 31.1 mg) was prepared following the general procedure without modification and isolated by column chromatography as a coloerless oil. This compound is known, and its characterization data are consistent with the litrature report.[29]
^{1}H NMR (500 MHz, $CDCl_3$) δ 7.83 – 7.77 (m, 3H), 7.67 (s, 1H), 7.48 – 7.38 (m, 3H), 2.51 (s, 2H), 1.28 (s, 12H);
^{13}C NMR (125 MHz, $CDCl_3$) δ 136.4, 133.9, 131.5, 128.3, 127.7, 127.6, 127.3, 126.6, 125.7, 124.7, 83.5, 24.8 (*The carbon attached to boron was not observed due to quadrupolar relaxation*);
^{11}B NMR (161 MHz, $CDCl_3$) δ 33.1 (bs, 1B);
GC-MS (EI) for $C_{17}H_{21}BO_2$ Calcd: 268.2 found: 268.1.

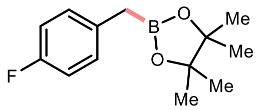

2-(4-Fluorobenzyl)-4,4,5,5-tetramethyl-1,3,2-dioxaborolane (**14b**, condition A: 49%, 23.1 mg based on ^{19}F NMR) was prepared following the general procedure without modification and isolated by column chromatography as a colorless oil. This compound is known, and its characterization data are consistent with the literature report.[25]
^{1}H NMR (500 MHz, $CDCl_3$) δ 7.17 – 7.13 (m, 2H), 6.97 – 6.92 (m, 2H), 2.28 (s, 2H), 1.25 (s, 12H);
^{13}C NMR (125 MHz, $CDCl_3$) δ 160.8 (d, J = 242.0 Hz), 134.1 (d, J = 3.2 Hz), 130.2 (d, J = 7.7 Hz), 114.9 (d, J = 21.0 Hz), 83.5, 24.7 (*The carbon attached to boron was not observed due to quadrupolar relaxation*);
^{11}B NMR (161 MHz, $CDCl_3$) δ 33.1 (bs, 1B);
^{19}F NMR (471 MHz, $CDCl_3$) δ -119.34 – -119.43 (m, 1F);
GC-MS (EI) for $C_{13}H_{18}BFO_2$ Calcd: 236.1, found: 236.1.

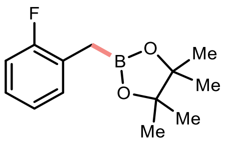

2-(2-Fluorobenzyl)-4,4,5,5-tetramethyl-1,3,2-dioxaborolane (**15b**, condition B: 50%, 23.6 mg based on ^{19}F NMR; 62%, 29.3 mg based on the recovered starting material) was prepared following the general procedure without modification and isolated by column chromatography as a coloerless oil. This compound is known, and its characterization data are consistent with the litrature report.[25]

^{1}H NMR (500 MHz, $CDCl_3$) δ 7.22 (td, *J* = 7.7, 1.6 Hz, 1H), 7.16 – 7.11 (m, 1H), 7.06 – 6.99 (m, 2H), 2.29 (s, 2H), 1.27 (s, 12H);
^{13}C NMR (125 MHz, $CDCl_3$) δ 161.0 (d, *J* = 243.5 Hz), 131.3 (d, *J* = 4.9 Hz), 126.6 (d, *J* = 7.9 Hz), 126.0 (d, *J* = 16.8 Hz), 123.8 (d, *J* = 3.6 Hz), 114.9 (d, *J* = 22.1 Hz), 83.6, 24.7 (*The carbon attached to boron was not observed due to quadrupolar relaxation*);
^{19}F NMR (471 MHz, $CDCl_3$) δ -116.80 – -116.88 (m, 1F);
^{11}B NMR (161 MHz, $CDCl_3$) δ 33.08 (bs, 1B);
GC-MS (EI) for $C_{13}H_{18}BFO_2$ Calcd: 236.1, found: 236.1.

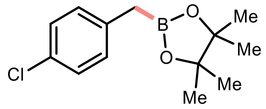

2-(4-Chlorobenzyl)-4,4,5,5-tetramethyl-1,3,2-dioxaborolane (**16b**, condition A: 56%, 28.3 mg) was prepared following the general procedure without modification and isolated by column chromatography as a colourless oil. This compound is known, and its characterization data are consistent with the litrature report.[29]
^{1}H NMR (500 MHz, $CDCl_3$) δ 7.22 (d, *J* = 8.4 Hz, 2H), 7.13 (d, *J* = 8.5 Hz, 2H), 2.28 (s, 2H), 1.25 (s, 12H);
^{13}C NMR (125 MHz, $CDCl_3$) δ 137.2, 130.6, 130.3, 128.3, 83.6, 24.7 (*The carbon attached to boron was not observed due to quadrupolar relaxation*);
^{11}B NMR (161 MHz, $CDCl_3$) δ 33.0 (bs, 1B);
GC-MS (EI) for $C_{13}H_{18}BClO_2$ Calcd: 252.1 (100%), 254.1 (33%), found: 252.1 (100%), 254.1 (33%).

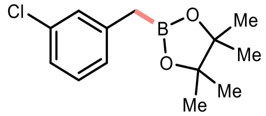

2-(3-Chlorobenzyl)-4,4,5,5-tetramethyl-1,3,2-dioxaborolane (**17b**, condition A: 48%, 24.2 mg) was prepared following the general procedure without modification and isolated by column chromatography as a coloerless oil. This compound is known, and its characterization data are consistent with the litrature report.[25]
^{1}H NMR (500 MHz, $CDCl_3$) δ 7.20 – 7.20 (m, 1H), 7.17 (d, *J* = 7.7 Hz, 1H), 7.13 – 7.11 (m, 1H), 7.09 – 7.07 (m, 1H), 2.29 (s, 2H), 1.26 (s, 12H);
^{13}C NMR (125 MHz, $CDCl_3$) δ 140.8, 133.9, 129.4, 129.1, 127.2, 125.1, 83.6, 24.7 (*The carbon attached to boron was not observed due to quadrupolar relaxation*);
^{11}B NMR (161 MHz, $CDCl_3$) δ 32.9 (bs, 1B);
GC-MS (EI) for $C_{13}H_{18}BClO_2$ Calcd: 252.1 (100%), 254.1 (33%), found: 252.1 (100%), 254.1 (33%).

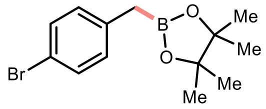

2-(4-Bromo-benzyl)-4,4,5,5-tetramethyl-1,3,2-dioxaborolane (**18b**, condition B: 47%, 27.9 mg) was prepared following the general procedure without modification and isolated by column chromatography

as a colourless oil. This compound is known, and its characterization data are consistent with the literature report.[25]

^{1}H NMR (500 MHz, $CDCl_3$) δ 7.37 (d, *J* = 8.3 Hz, 2H), 7.08 (d, *J* = 8.2 Hz, 2H), 2.26 (s, 2H), 1.25 (s, 12H);
^{13}C NMR (126 MHz, $CDCl_3$) δ 137.7, 131.3, 130.7, 118.6, 83.6, 24.7 (*The carbon attached to boron was not observed due to quadrupolar relaxation*);
^{11}B NMR (161 MHz, $CDCl_3$) δ 32.9 (bs, 1B);
GC-MS (EI) for $C_{13}H_{18}BBrO_2$ Calcd: 296.1 (100%), 298.1 (97%), found: 296.0 (100%), 298.0 (97%).

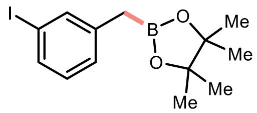

2-(3-Iodobenzyl)-4,4,5,5-tetramethyl-1,3,2-dioxaborolane (**19b**, condition B: 37%, 25.5 mg) was prepared following the general procedure without modification and isolated by column chromatography as a coloerless oil. This compound is known, and its characterization data are consistent with the litrature report.[30]

^{1}H NMR (500 MHz, $CDCl_3$) δ 7.57 (s, 1H), 7.48 (d, *J* = 7.9 Hz, 1H), 7.17 (dd, *J* = 7.7, 0.6 Hz, 1H), 6.99 (t, *J* = 7.8 Hz, 1H), 2.25 (s, 2H), 1.26 (s, 12H);
^{13}C NMR (125 MHz, $CDCl_3$) δ 141.2, 137.9, 134.0, 129.9, 128.3, 94.5, 83.6, 24.7 (*The carbon attached to boron was not observed due to quadrupolar relaxation*);
^{11}B NMR (161 MHz, $CDCl_3$) δ 32.9 (bs, 1B);
GC-MS (EI) for $C_{13}H_{18}BIO_2$ Calcd: 344.0, found: 344.0.

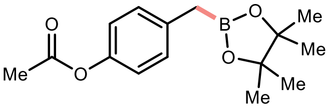

4-((4,4,5,5-Tetramethyl-1,3,2-dioxaborolan-2-yl)methyl)phenyl acetate (**20b**, condition A: 42%, 23.2 mg) was prepared following the general procedure without modification and isolated by column chromatography as a coloerless oil. This compound is known, and its characterization data are consistent with the litrature report.[25]

^{1}H NMR (500 MHz, $CDCl_3$) δ 7.20 (d, *J* = 8.6 Hz, 2H), 6.97 (d, *J* = 8.5 Hz, 2H), 2.30 (s, 5H), 1.26 (s, 12H);
^{13}C NMR (125 MHz, $CDCl_3$) δ 169.7, 148.1, 136.2, 129.9, 121.2, 83.5, 24.8, 21.2 (*The carbon attached to boron was not observed due to quadrupolar relaxation*);
^{11}B NMR (161 MHz, $CDCl_3$) δ 33.0 (bs, 1B);
GC-MS (EI) for $C_{15}H_{21}BO_4$ Calcd: 276.2, found: 276.1.

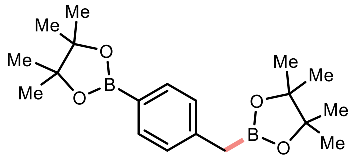

4,4,5,5-Tetramethyl-2-(4-(4,4,5,5-tetramethyl-1,3,2-dioxaborolan-2-yl)benzyl)-1,3,2-dioxaborolane (**21b**, condition A: 35%, 24.1 mg) was prepared following the general procedure without modification and

isolated by column chromatography as a coloerless oil. This compound is known, and its characterization data are consistent with the litrature report.[25]

^{1}H NMR (500 MHz, $CDCl_3$) δ 7.71 (d, *J* = 8.0 Hz, 2H), 7.21 (d, *J* = 8.1 Hz, 2H), 2.33 (s, 2H), 1.35 (s, 12H), 1.24 (s, 12H);

^{13}C NMR (125 MHz, $CDCl_3$) δ 142.3, 134.8, 128.4, 83.5, 83.5, 24.9, 24.7 (*The carbon attached to boron was not observed due to quadrupolar relaxation*);

^{11}B NMR (161 MHz, $CDCl_3$) δ 33.0 (bs, 1B), 31.1 (bs, 1B);

GC-MS (EI) for $C_{19}H_{30}B_2O_4$ Calcd:344.2, found: 344.2.

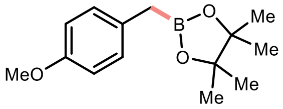

2-(4-Methoxybenzyl)-4,4,5,5-tetramethyl-1,3,2-dioxaborolane (**22b**, condition B: 53%, 26.3 mg) was prepared following the general procedure without modification and isolated by column chromatography as a colourless oil. This compound is known, and its characterization data are consistent with the literature report.[31]

^{1}H NMR (500 MHz, $CDCl_3$) δ 7.12 (d, *J* = 8.7 Hz, 2H), 6.81 (d, *J* = 8.7 Hz, 2H), 3.79 (s, 3H), 2.25 (s, 2H), 1.26 (s, 12H).

^{13}C NMR (125 MHz, $CDCl_3$) δ 157.1, 130.5, 129.8, 113.8, 83.4, 55.2, 24.7 (*The carbon attached to boron was not observed due to quadrupolar relaxation*);

^{11}B NMR (161 MHz, $CDCl_3$) δ 33.1 (bs, 1B);

GC-MS (EI) for $C_{14}H_{21}BO_3$ Calcd: 248.1, found: 248.2.

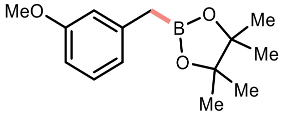

2-(3-Methoxybenzyl)-4,4,5,5-tetramethyl-1,3,2-dioxaborolane (**23b**, condition B: 49%, 24.3 mg) was prepared following the general procedure without modification and isolated by column chromatography as a coloerless oil. This compound is known, and its characterization data are consistent with the litrature report.[29]

^{1}H NMR (500 MHz, $CDCl_3$) δ 7.18 (t, *J* = 7.9 Hz, 1H), 6.80 (d, *J* = 7.5 Hz, 1H), 6.78 – 6.78 (m, 1H), 6.70 (dd, *J* = 8.2, 2.3 Hz, 1H), 3.81 (s, 3H), 2.30 (s, 2H), 1.26 (s, 12H);

^{13}C NMR (125 MHz, $CDCl_3$) δ 159.5, 140.2, 129.2, 121.5, 114.6, 110.4, 83.4, 55.1, 24.7 (*The carbon attached to boron was not observed due to quadrupolar relaxation*);

^{11}B NMR (161 MHz, $CDCl_3$) δ 33.1 (bs, 1B);

GC-MS (EI) for $C_{14}H_{21}BO_3$ Calcd: 248.1, found: 248.2.

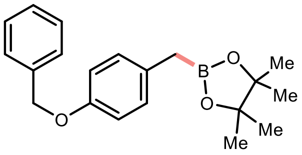

2-(4-(Benzyloxy)benzyl)-4,4,5,5-tetramethyl-1,3,2-dioxaborolane (**24b**, condition B: 65%, 42.1 mg) was prepared following the general procedure without modification and isolated by column chromatography as a colourless oil. This compound is unknown in the literature.

^{1}H NMR (500 MHz, $CDCl_3$) δ 7.45 (d, *J* = 7.6 Hz, 2H), 7.40 (t, *J* = 7.4 Hz, 2H), 7.34 (tt, *J* = 7.2, 1.2 Hz, 1H), 7.12 (d, *J* = 8.7 Hz, 2H), 6.89 (d, *J* = 8.7 Hz, 2H), 5.05 (s, 2H), 2.25 (s, 2H), 1.26 (s, 12H);

^{13}C NMR (125 MHz, $CDCl_3$) δ 156.4, 137.4, 130.8, 129.9, 128.5, 127.8, 127.5, 114.8, 83.4, 70.0, 24.8 (*The carbon attached to boron was not observed due to quadrupolar relaxation*);

^{11}B NMR (161 MHz, $CDCl_3$) δ 33.2 (bs, 1B);

GC-MS (EI) for $C_{20}H_{25}BO_3$ Calcd: 324.2, found: 324.1.

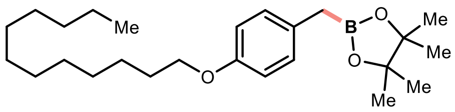

2-(4-(Dodecyloxy)benzyl)-4,4,5,5-tetramethyl-1,3,2-dioxaborolane (**25b**, condition B: 63%, 50.7 mg) was prepared following the general procedure without modification and isolated by column chromatography as a colourless oil. This compound is unknown in the literature.

^{1}H NMR (500 MHz, $CDCl_3$) δ 7.10 (d, *J* = 8.6 Hz, 2H), 6.80 (d, *J* = 8.6 Hz, 2H), 3.93 (t, *J* = 6.6 Hz, 2H), 2.24 (s, 2H), 1.77 (dt, *J* = 14.6, 6.7 Hz, 2H), 1.49-1.43 (m, 2H), 1.38 – 1.29 (m, 16H), 1.25 (s, 12H), 0.91 (t, *J* = 7.0 Hz, 3H);

^{13}C NMR (125 MHz, $CDCl_3$) δ 156.7, 130.2, 129.8, 114.4, 83.3, 68.0, 31.9, 29.7, 29.6, 29.6, 29.6, 29.4, 29.4, 29.4, 26.1, 24.7, 22.7, 14.1 (*The carbon attached to boron was not observed due to quadrupolar relaxation*);

^{11}B NMR (161 MHz, $CDCl_3$) δ 33.4 (bs, 1B);

GC-MS (EI) for $C_{25}H_{43}BO_3$ Calcd: 402.3, found: 402.3;

HRMS (APCI, M+H$^+$) for $C_{25}H_{44}BO_3$ Calcd: 403.3378, found: 403.3383.

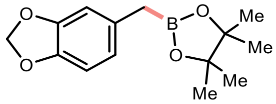

2-(4-(Dodecyloxy)benzyl)-4,4,5,5-tetramethyl-1,3,2-dioxaborolane (**26b**, condition B: 62%, 32.5 mg) was prepared following the general procedure without modification and isolated by column chromatography as a coloerless oil. This compound is known, and its characterization data are consistent with the litrature report.[32]

^{1}H NMR (500 MHz, $CDCl_3$) δ 6.72 (s, 1H), 6.71 (d, *J* = 6.0 Hz, 1H), 6.64 (dd, *J* = 8.0, 1.6 Hz, 1H), 5.92 (s, 2H), 2.24 (s, 2H), 1.26 (s, 12H);

^{13}C NMR (125 MHz, $CDCl_3$) δ 147.4, 145.0, 132.2, 121.5, 109.6, 108.1, 100.6, 83.4, 24.7 (*The carbon attached to boron was not observed due to quadrupolar relaxation*);

^{11}B NMR (161 MHz, $CDCl_3$) δ 32.9 (bs, 1B);

GC-MS (EI) for $C_{14}H_{19}BO_4$ Calcd:262.1, found: 262.1.

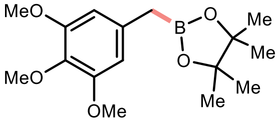

4,4,5,5-Tetramethyl-2-(3,4,5-trimethoxybenzyl)-1,3,2-dioxaborolane (**27b**, condition B: 48%, 29.6 mg based on ^{1}H NMR) was prepared following the general procedure without modification. For convenient isolation, the benzylboronate was converted to the corresponding alcohol (**27d**).
GC-MS (EI) for $C_{16}H_{25}BO_5$ Calcd: 308.2, found: 308.2.

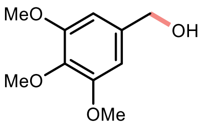

(3,4,5-Trimethoxyphenyl)methanol (**27d**) was prepared following the general procedure without modification. This compound is known, and its characterization data are consistent with the literature report.[33]
^{1}H NMR (500 MHz, $CDCl_3$) δ 6.53 (s, 2H), 4.55 (s, 2H), 3.79 (s, 6H), 3.78 (s, 3H), 2.71 (s, 1H);
^{13}C NMR (125 MHz, $CDCl_3$) δ 153.2, 137.0, 136.9, 103.7, 65.2, 60.8, 56.0;
GC-MS (EI) for $C_{10}H_{14}O_4$ Calcd: 198.1, *not found possibly due to the instability or non-volatility of the target compound.*

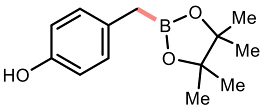

4-((4,4,5,5-Tetramethyl-1,3,2-dioxaborolan-2-yl)methyl)phenol (**28b**, condition A: 68%, 31.8 mg based on ^{1}H NMR) was prepared following the general procedure without modification. For convenient isolation, the benzylboronate was converted to the corresponding alcohol (**28d**).
GC-MS (EI) for $C_{13}H_{19}BO_3$ Calcd: 234.1, found: 234.2.

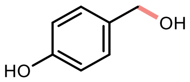

4-(Hydroxymethyl)phenol (**28d**) was prepared following the general procedure without modification. This compound is known, and its characterization data are consistent with the literature report.[33]
^{1}H NMR (500 MHz, DMSO-d_6) δ 9.32 (s, 1H), 7.10 (d, J = 8.5 Hz, 2H), 6.70 (d, J = 8.5 Hz, 2H), 4.94 (s, 1H), 4.36 (s, 2H);
^{13}C NMR (125 MHz, DMSO-d_6) δ 156.7, 133.2, 128.5, 115.2, 63.2;
GC-MS (EI) for $C_7H_8O_2$ Calcd: 124.1, *not found possibly due to the instability or non-volatility of the target compound.*

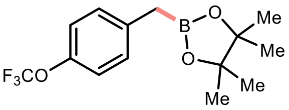

4,4,5,5-Tetramethyl-2-(4-(trifluoromethoxy)benzyl)-1,3,2-dioxaborolane (**29b**, condition A: 43%, 26.0 mg based on ^{19}F NMR; 52%, 31.4 mg based on recovered starting material) was prepared following the general procedure without modification and isolated by column chromatography as a coloerless oil. This compound is known, and its characterization data are consistent with the litrature report.[25]
^{1}H NMR (500 MHz, $CDCl_3$) δ 7.21 (d, J = 8.7 Hz, 2H), 7.10 (d, J = 7.9 Hz, 2H), 2.31 (s, 2H), 1.26 (s, 12H);

^{13}C NMR (125 MHz, $CDCl_3$) δ 146.8, 137.5, 130.1, 120.8, 120.6 (q, J = 256.2 Hz), 83.6, 24.7 (*The carbon attached to boron was not observed due to quadrupolar relaxation*);
^{19}F NMR (471 MHz, $CDCl_3$) δ -57.92 (s, 3F);
^{11}B NMR (161 MHz, $CDCl_3$) δ 33.0 (bs, 1B);
GC-MS (EI) for $C_{14}H_{18}BF_3O_3$ Calcd: 302.1, found: 302.1.

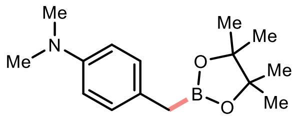

***N*,*N*-Dimethyl-4-((4,4,5,5-tetramethyl-1,3,2-dioxaborolan-2-yl)methyl)aniline** (**30b**, condition A: 47%, 24.5 mg based on ^{1}H NMR) was prepared following the general procedure without modification. For convenient isolation, the benzylboronate was converted to the corresponding alcohol (**30d**).
GC-MS (EI) for $C_{15}H_{24}BNO_2$ Calcd: 261.2, found: 261.1.

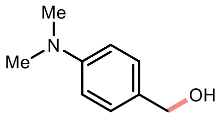

(4-(Dimethylamino)phenyl)methanol (**30d**) was prepared following the general procedure without modification. This compound is known, and its characterization data are consistent with the literature report.[34]
^{1}H NMR (500 MHz, $CDCl_3$) δ 7.26 (d, J = 8.7 Hz, 2H), 6.76 (d, J = 8.7 Hz, 2H), 4.56 (s, 2H), 2.98 (s, 6H);
^{13}C NMR (125 MHz, $CDCl_3$) δ 150.4, 129.2, 128.6, 112.8, 65.2, 40.8;
GC-MS (EI) for $C_9H_{13}NO$ Calcd: 151.1, *not found possibly due to the instability or non-volatility of the target compound.*

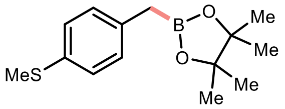

4,4,5,5-Tetramethyl-2-(4-(methylthio)benzyl)-1,3,2-dioxaborolane (**31b**, condition A: 45%, 23.8 mg) was prepared following the general procedure without modification and isolated by column chromatography as a coloerless oil. This compound is known, and its characterization data are consistent with the litrature report.[25]
^{1}H NMR (500 MHz, $CDCl_3$) δ 7.19 (d, J = 8.3 Hz, 2H), 7.13 (d, J = 8.3 Hz, 2H), 2.48 (s, 3H), 2.27 (s, 2H), 1.25 (s, 12H);
^{13}C NMR (125 MHz, $CDCl_3$) δ 135.9, 134.0, 129.5, 127.4, 83.5, 24.7, 16.5 (*The carbon attached to boron was not observed due to quadrupolar relaxation*);
^{11}B NMR (161 MHz, $CDCl_3$) δ 33.1 (bs, 1B);
GC-MS (EI) for $C_{14}H_{21}BO_2S$ Calcd: 264.1, found: 264.1.

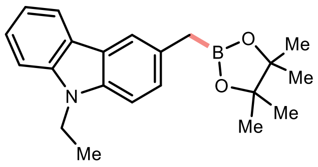

9-Ethyl-3-((4,4,5,5-tetramethyl-1,3,2-dioxaborolan-2-yl)methyl)-9*H*-carbazole (**32b**, condition A: 52%, 37.2 mg) was prepared following the general procedure without modification and isolated by column chromatography as a colourless oil. This compound is unknown in the literature.

^{1}H NMR (500 MHz, $CDCl_3$) δ 8.08 (d, *J* = 7.7 Hz, 1H), 7.93 (s, 1H), 7.45 (td, *J* = 7.6, 7.1, 1.1 Hz, 1H), 7.39 (d, *J* = 8.2 Hz, 1H), 7.34 – 7.30 (m, 2H), 7.22 – 7.19 (m, 1H), 4.36 (q, *J* = 7.2 Hz, 2H), 2.50 (s, 2H), 1.44 (t, *J* = 7.2 Hz, 3H), 1.27 (s, 12H);

^{13}C NMR (125 MHz, $CDCl_3$) δ 140.1, 138.1, 128.6, 127.0, 125.3, 123.1, 122.9, 120.4, 120.3, 118.3, 108.3, 108.2, 83.4, 37.5, 24.8, 13.9 (*The carbon attached to boron was not observed due to quadrupolar relaxation*);

^{11}B NMR (161 MHz, $CDCl_3$) δ 33.2 (bs, 1B);

GC-MS (EI) for $C_{21}H_{26}BNO_2$ Calcd: 335.2, found: 355.2;

HRMS (APCI, M+Na$^+$) for $C_{21}H_{26}BNNaO_2$ Calcd: 358.1949, found: 358.1948.

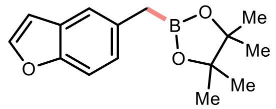

2-(Benzofuran-5-ylmethyl)-4,4,5,5-tetramethyl-1,3,2-dioxaborolane (**33b**, condition A: 45%, 23.2 mg) was prepared following the general procedure without modification and isolated by column chromatography as a colourless oil. This compound is unknown in the literature.

^{1}H NMR (500 MHz, $CDCl_3$) δ 7.58 (d, *J* = 2.2 Hz, 1H), 7.41 (d, *J* = 3.4 Hz, 1H), 7.39 (d, *J* = 8.4 Hz, 1H), 7.13 (dd, *J* = 8.4, 1.7 Hz, 1H), 6.71 (dd, *J* = 2.2, 0.9 Hz, 1H), 2.40 (s, 2H), 1.26 (s, 12H);

^{13}C NMR (125 MHz, $CDCl_3$) δ 153.2, 144.8, 132.8, 127.6, 125.5, 120.9, 110.9, 106.4, 83.4, 24.7 (*The carbon attached to boron was not observed due to quadrupolar relaxation*);

^{11}B NMR (161 MHz, $CDCl_3$) δ 33.2 (bs, 1B);

GC-MS (EI) for $C_{15}H_{19}BO_3$ Calcd: 258.1, found: 258.1;

HRMS (APCI, M+Na$^+$) for $C_{15}H_{19}BNaO_3$ Calcd: 281.1319, found: 281.1313.

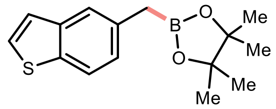

2-(Benzo[*b*]thiophen-5-ylmethyl)-4,4,5,5-tetramethyl-1,3,2-dioxaborolane (**34b**, condition A: 63%, 34.5 mg) was prepared following the general procedure without modification and isolated by column chromatography as a colourless oil. This compound is unknown in the literature.

^{1}H NMR (500 MHz, $CDCl_3$) δ 7.76 (d, *J* = 8.3 Hz, 1H), 7.65 (d, *J* = 0.8 Hz, 1H), 7.40 (d, *J* = 5.4 Hz, 1H), 7.27 (dd, *J* = 5.5, 0.5 Hz, 1H), 7.21 (dd, *J* = 8.2, 1.6 Hz, 1H), 2.43 (s, 2H), 1.26 (s, 12H);

^{13}C NMR (125 MHz, $CDCl_3$) δ 140.1, 136.5, 134.7, 126.1, 126.1, 123.6, 123.5, 122.1, 83.5, 24.8 (*The carbon attached to boron was not observed due to quadrupolar relaxation*);

^{11}B NMR (161 MHz, $CDCl_3$) δ 33.2 (bs, 1B);

GC-MS (EI) for $C_{15}H_{19}BO_2S$ Calcd: 274.1, found: 274.1.

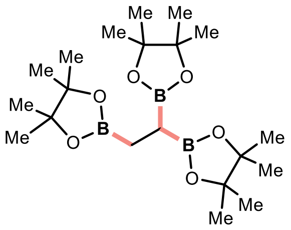

2,2',2''-(Ethane-1,1,2-triyl)tris(4,4,5,5-tetramethyl-1,3,2-dioxaborolane) (**35b**, condition A: 58%, 47.3 mg) was prepared following the general procedure without modification and isolated by column chromatography as a coloerless oil. This compound is unknown in the literature.

^{1}H NMR (500 MHz, $CDCl_3$) δ 1.24 (s, 12H), 1.23 (s, 12H), 1.22 (s, 12H), 0.97 (d, *J* = 7.6 Hz, 2H), 0.79 (t, *J* = 7.6 Hz, 1H);

^{13}C NMR (125 MHz, $CDCl_3$) δ 82.8, 82.8, 24.8, 24.8, 24.6 (*The carbons attached to borons were not observed due to quadrupolar relaxation and one aliphatic carbon was missing due to the overlapping*);

^{11}B NMR (161 MHz, $CDCl_3$) δ 34.3 (bs, 3B) (*One peak was missing due to the signal overlap*);

GC-MS (EI) for $C_{20}H_{39}B_3O_6$ Calcd: 408.3, found: 393.3 ($M^{\cdot+}$-Me);

HRMS (APCI, M+H$^+$) for $C_{20}H_{40}B_3O_6$ Calcd: 409.3099, found: 409.3105.

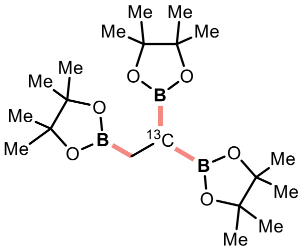

2,2',2''-(1λ^3-ethane-1,1,2-triyl-1-^{13}*C*)tris(4,4,5,5-tetramethyl-1,3,2-dioxaborolane) (**35b-^{13}C$_1$**, condition B: 57%, 46.6 mg) was prepared following the general procedure without modification and isolated by column chromatography as a coloerless oil. This compound is unknown in the literature.

^{1}H NMR (500 MHz, $CDCl_3$) δ 1.23 (s, 12H), 1.23 (s, 12H), 1.22 (s, 12H), 0.99 – 0.93 (m, 2H), 0.79 (dt, *J* = 112.0, 7.6 Hz, 1H);

^{13}C NMR (125 MHz, $CDCl_3$) δ 82.8, 82.8, 24.8, 24.8, 24.6, 3.4 (bs, 1^{13}C) (*One carbon attached to boron was not observed due to quadrupolar relaxation and one aliphatic carbon was missing due to the overlapping*);

^{11}B NMR (161 MHz, $CDCl_3$) δ 34.3 (bs, 3B) (*One peak was missing due to the signal overlap*);

GC-MS (EI) for $C_{19}{}^{13}CH_{39}B_3O_6$ Calcd: 409.3, *parent peak not found possibly due to the instability of the target compound and the fragmentation pattern is difficult to analyze;*

HRMS (APCI, M+H$^+$) for $C_{19}{}^{13}CH_{40}B_3O_6$ Calcd: 410.3132, found: 410.3141.

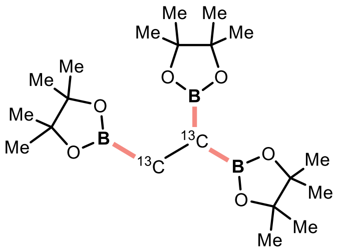

2,2',2''-(1λ[3],2λ[2]-ethane-1,1,2-triyl-^{13}*C*$_2$)tris(4,4,5,5-tetramethyl-1,3,2-dioxaborolane) (**35b-^{13}C$_2$**, condition B: 51%, 41.8 mg) was prepared following the general procedure without modification and isolated by column chromatography as a coloerless oil. This compound is unknown in the literature.

^{1}H NMR (500 MHz, $CDCl_3$) δ 1.23 (s, 12H), 1.22 (s, 12H), 1.22 (s, 12H), 1.10-0.83 (m, 2H), 0.92-0.65 (m, 1H);

^{13}C NMR (125 MHz, $CDCl_3$) δ 82.8, 82.8, 24.8, 24.8, 24.6, 6.1 (bs, 1^{13}C), 3.4 (bs, 1^{13}C) (*One aliphatic carbon was missing due to the overlapping*);

^{11}B NMR (161 MHz, $CDCl_3$) δ 34.3 (bs, 3B) (*One peak was missing due to the signal overlap*);

GC-MS (EI) for $C_{18}{}^{13}C_2H_{39}B_3O_6$ Calcd: 410.3, *parent peak not found possibly due to the instability of the target compound and the fragmentation pattern is difficult to analyze;*

HRMS (APCI, M+H$^+$) for $C_{18}{}^{13}C_2H_{40}B_3O_6$ Calcd: 411.3166, found:411.3177.

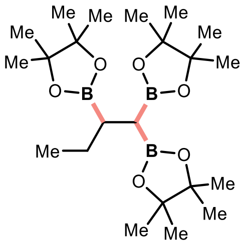

2,2',2''-(Butane-1,1,2-triyl)tris(4,4,5,5-tetramethyl-1,3,2-dioxaborolane) (**36b**, condition A: 57%, 49.7 mg) was prepared following the general procedure without modification and isolated by column chromatography as a coloerless oil. This compound is unknown in the literature.

^{1}H NMR (500 MHz, $CDCl_3$) δ 1.49 (dq, *J* = 7.3, 7.3 Hz, 2H), 1.34 – 1.31 (m, 1H), 1.23 (s, 24H), 1.23 (s, 12H), 0.90 (d, *J* = 10.2 Hz, 1H), 0.88 (t, *J* = 7.4 Hz, 3H);

^{13}C NMR (125 MHz, $CDCl_3$) δ 82.7, 82.7, 26.1, 25.1, 24.9, 24.8, 24.7, 24.7, 24.6, 13.2 (*The carbons attached to borons were not observed due to quadrupolar relaxation and one aliphatic carbon was missing due to the overlapping*);

^{11}B NMR (161 MHz, $CDCl_3$) δ 34.3 (bs, 3B) (*One peak was missing due to the signal overlap*);

GC-MS (EI) for $C_{22}H_{43}B_3O_6$ Calcd: 436.3, found: 421.4 ($M^{\cdot+}$-Me);

HRMS (APCI, M+H$^+$) for $C_{22}H_{44}B_3O_6$ Calcd: 437.3412, found: 437.3425.

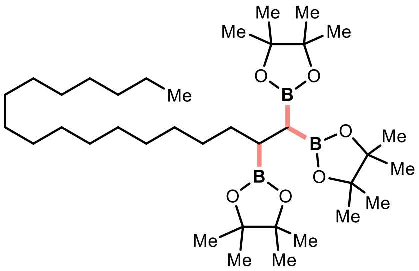

2,2',2''-(Octadecane-1,1,2-triyl)tris(4,4,5,5-tetramethyl-1,3,2-dioxaborolane) (**37b**, condition B: 63%, 79.6 mg) was prepared following the general procedure without modification and isolated by column chromatography as a coloerless oil. This compound is unknown in the literature.

^{1}H NMR (500 MHz, $CDCl_3$) δ 1.49 – 1.23 (m, 67H), 0.91 – 0.88 (m, 4H);

^{13}C NMR (125 MHz, $CDCl_3$) δ 82.7, 82.7, 82.6, 33.4, 31.9, 30.0, 29.7, 29.7, 29.6, 29.4, 28.7, 25.0, 24.9, 24.9, 24.7, 24.6, 22.7, 14.1 (*The carbons attached to borons were not observed due to quadrupolar relaxation and seven aliphatic carbons were missing due to the overlapping*);

^{11}B NMR (161 MHz, $CDCl_3$) δ 34.9 (bs, 3B) (*One peak was missing due to the signal overlap*);

GC-MS (EI) for $C_{36}H_{71}B_3O_6$ Calcd: 632.6, found: 574.6 ($M^{\cdot+}$-*n*-Bu);

HRMS (APCI, M+H$^+$) for $C_{36}H_{72}B_3O_6$ Calcd: 633.5603, found: 633.5626.

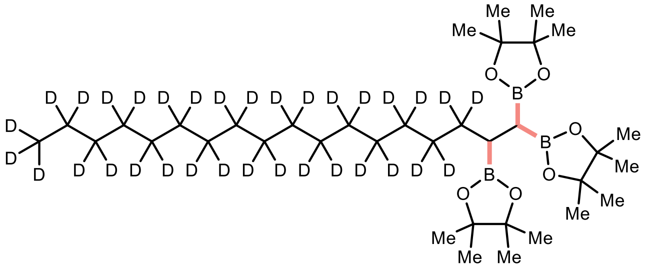

2,2',2''-(Octadecane-1,1,2-triyl-3,3,4,4,5,5,6,6,7,7,8,8,9,9,10,10,11,11,12,12,13,13,14,14,15,15,16,16,17,17,18,18,18-*d*$_{33}$)tris(4,4,5,5-tetramethyl-1,3,2-dioxaborolane) (**37b-*d***, condition A: 46%, 61.3 mg) was prepared following the general procedure without modification and isolated by column chromatography as a colourless oil. This compound is unknown in the literature.

^{1}H NMR (500 MHz, $CDCl_3$) δ 1.23 – 1.22 (m, 36H), 0.88 (d, *J* = 10.2 Hz, 1H);

^{13}C NMR (200 MHz, $CDCl_3$) δ 82.7, 32.3, 30.5, 28.7, 28.4, 28.3, 28.3, 28.2, 28.0, 27.4, 27.4, 26.0-23.7 (m), 21.4, 12.9 (*The spectra was too difficult to be analzyed*);

^{11}B NMR (161 MHz, $CDCl_3$) δ 34.3 (bs, 3B) (*One peak was missing due to the signal overlap*);

^{2}H NMR (77 MHz, $CDCl_3$) δ 1.16, 0.80 *(Peaks were missing due to the signal overlap*);

GC-MS (EI) for $C_{36}H_{38}D_{33}B_3O_6$ Calcd: 665.8, found: 664.9 ($M^{\cdot+}$-H);

HRMS (APCI, M+H$^+$) for $C_{36}H_{39}B_3D_{33}O_6$ Calcd: 666.7674, found: 666.7693.

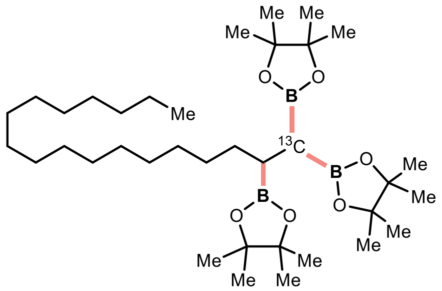

2,2',2''-(Octadecane-1,1,2-triyl-1-^{13}*C*)tris(4,4,5,5-tetramethyl-1,3,2-dioxaborolane) (**37b-^{13}C**, condition A: 67%, 85.0 mg) was prepared following the general procedure without modification and isolated by column chromatography as a coloerless oil. This compound is unknown in the literature.

^{1}H NMR (500 MHz, $CDCl_3$) δ 1.47 – 1.11 (m, 67H), 0.90 (t, *J* = 7.0 Hz, 3H), 0.88 (dd, *J* = 112.0, 10.0 Hz, 1H);

^{13}C NMR (125 MHz, $CDCl_3$) δ 82.7, 82.7, 82.6, 45.8, 33.4, 31.9, 30.0, 29.7, 29.7, 29.6, 29.4, 28.7, 28.6, 25.0, 24.9, 24.9, 24.7, 24.6, 22.7, 14.1, 11.6 (bs, 1^{13}C) (*One carbons attached to borons were not observed due to quadrupolar relaxation and five aliphatic carbons were missing due to the overlapping*);

^{11}B NMR (161 MHz, $CDCl_3$) δ 34.5 (bs, 3B) (*One peak was missing due to the signal overlap*);

GC-MS (EI) for $C_{35}{}^{13}CH_{71}B_3O_6$ Calcd: 633.6, *parent peak not found possibly due to the instability of the target compound, and the fragmentation pattern is difficult to analyze*;

HRMS (APCI, M+H$^+$) for $C_{35}H_{72}B_3O_6{}^{13}C$ Calcd: 634.5636, found: 634.5660.

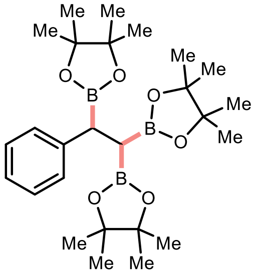

2,2',2''-(2-Phenylethane-1,1,2-triyl)tris(4,4,5,5-tetramethyl-1,3,2-dioxaborolane) (**38b**, condition A: 67%, 64.9 mg) was prepared following the general procedure without modification and isolated by column chromatography as a coloerless oil. This compound is known, and its characterization data are consistent with the litrature report.[25]

^{1}H NMR (500 MHz, $CDCl_3$) δ 7.23 (dd, *J* = 8.1, 1.2 Hz, 2H), 7.18 (t, *J* = 7.7 Hz, 2H), 7.07 – 7.03 (m, 1H), 2.69 (d, *J* = 12.7 Hz, 1H), 1.47 (d, *J* = 13.1 Hz, 1H), 1.26 (s, 6H), 1.25 (s, 6H), 1.17 (s, 6H), 1.15 (s, 6H), 0.97 (s, 6H), 0.95 (s, 6H);

^{13}C NMR (125 MHz, $CDCl_3$) δ 145.3, 128.5, 127.8, 124.7, 83.1, 83.0, 82.7, 24.9, 24.8, 24.7, 24.4, 24.3, 24.2. (*The carbons attached to borons were not observed due to quadrupolar relaxation*);

^{11}B NMR (161 MHz, $CDCl_3$) δ 33.8 (bs, 3B) (*One peak was missing due to the signal overlap*);

GC-MS (EI) for $C_{26}H_{43}B_3O_6$ Calcd: 484.3, found: 469.4 ($M^{\cdot+}$-Me).

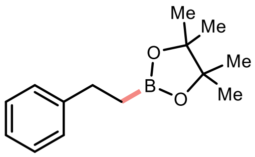

4,4,5,5-Tetramethyl-2-phenethyl-1,3,2-dioxaborolane (**38b'-H_2**, condition according to **Section 4.3.1.**: 66%, 30.6 mg) was prepared following the general procedure without modification and isolated by column chromatography as a coloerless oil. This compound is known, and its characterization data are consistent with the litrature report.[6]

^{1}H NMR (500 MHz, $CDCl_3$) δ 7.28 (t, *J* = 7.5 Hz, 2H), 7.24 (d, *J* = 6.9 Hz, 2H), 7.17 (t, *J* = 7.0 Hz, 1H), 2.77 (t, *J* = 8.3 Hz, 2H), 1.24 (s, 12H), 1.17 (t, *J* = 8.7 Hz, 2H);

^{13}C NMR (125 MHz, $CDCl_3$) δ 144.4, 128.2, 128.0, 125.5, 83.1, 30.0, 24.8 (*The carbon attached to boron was not observed due to quadrupolar relaxation*);

^{11}B NMR (161 MHz, $CDCl_3$) δ 34.0 (bs, 1B);

GC-MS (EI) for $C_{14}H_{21}BO_2$ Calcd: 232.2, *parent peak not found possibly due to the instability of the target compound, and the fragmentation pattern is difficult to analyze.*

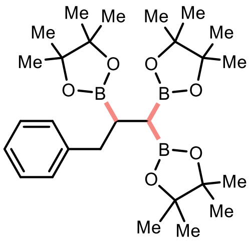

2,2',2''-(3-Phenylpropane-1,1,2-triyl)tris(4,4,5,5-tetramethyl-1,3,2-dioxaborolane) (**39b**, condition B: 49%, 48.8 mg) was prepared following the general procedure without modification and isolated by column chromatography as a coloerless oil. This compound is known, and its characterization data are consistent with the litrature report.[25]

^{1}H NMR (500 MHz, $CDCl_3$) δ 7.25 – 7.20 (m, 4H), 7.13 – 7.10 (m, 1H), 2.82 (dd, *J* = 13.3, 6.9 Hz, 1H), 2.74 (dd, *J* = 13.3, 8.7 Hz, 1H), 1.71 (td, *J* = 8.9, 7.0 Hz, 1H), 1.25 (s, 24H), 1.15 (s, 6H), 1.12 (s, 6H), 0.88 (d, *J* = 9.2 Hz, 1H);

^{13}C NMR (125 MHz, $CDCl_3$) δ 142.6, 129.3, 127.8, 125.3, 82.9, 82.8, 82.8, 39.2, 25.0, 24.9, 24.8, 24.8, 24.7 (*The carbons attached to borons were not observed due to quadrupolar relaxation and one aliphatic carbon was missing due to the overlapping*);

^{11}B NMR (161 MHz, $CDCl_3$) δ 34.0 (bs, 3B) (*One peak was missing due to the signal overlap*);

GC-MS (EI) for $C_{27}H_{45}B_3O_6$ Calcd: 498.3, found: 483.3 ($M^{\cdot+}$-Me).

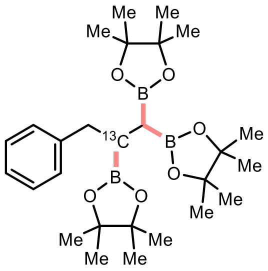

2,2',2''-(3-Phenylpropane-1,1,2-triyl-2-^{13}*C*)tris(4,4,5,5-tetramethyl-1,3,2-dioxaborolane) (**39b-^{13}C**, condition A: 45%, 44.9 mg) was prepared following the general procedure without modification and isolated by column chromatography as a coloerless oil. This compound is unknown in the literature.
^{1}H NMR (500 MHz, $CDCl_3$) δ 7.26 – 7.19 (m, 4H), 7.14 – 7.06 (m, 1H), 2.86 – 2.69 (m, 2H), 1.84 – 1.55 (m, 1H), 1.25 (s, 24H), 1.15 (s, 6H), 1.12 (s, 6H), 0.87 (dd, *J* = 9.1, 4.1 Hz, 1H);
^{13}C NMR (125 MHz, $CDCl_3$) δ 142.7, 129.3 (d, *J* = 1.2 Hz, 1C), 127.8, 125.3, 82.9, 82.8, 82.8, 39.2 (d, *J* = 30 Hz, 1C), 25.0, 25.0, 24.9, 24.8, 24.7, 22.1 (bs, ^{13}C) (*One carbons attached to borons were not observed due to quadrupolar relaxation and one aliphatic carbon was missing due to the overlapping*);
^{11}B NMR (161 MHz, $CDCl_3$) δ 34.1 (bs, 3B) (*One peak was missing due to the signal overlap*);
GC-MS (EI) for $C_{26}{}^{13}CH_{45}B_3O_6$ Calcd: 499.4, found: 499.4;
HRMS (APCI, M+H$^+$) for $C_{26}H_{46}B_3O_6{}^{13}C$ Calcd: 500.3602 found: 500.3620.

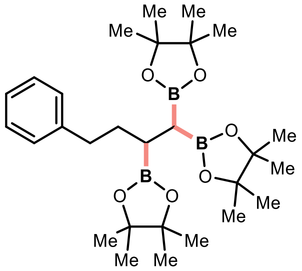

2,2',2''-(4-Phenylbutane-1,1,2-triyl)tris(4,4,5,5-tetramethyl-1,3,2-dioxaborolane) (**40b**, condition A: 61%, 62.5 mg) was prepared following the general procedure without modification and isolated by column chromatography as a coloerless oil. This compound is known, and its characterization data are consistent with the litrature report.[19]
^{1}H NMR (500 MHz, $CDCl_3$) δ 7.26 (t, *J* = 7.4 Hz, 2H), 7.19 (d, *J* = 6.9 Hz, 2H), 7.15 (t, *J* = 7.2 Hz, 1H), 2.68 (ddd, *J* = 13.4, 10.8, 6.0 Hz, 1H), 2.54 (ddd, *J* = 13.5, 10.7, 6.5 Hz, 1H), 1.82 – 1.71 (m, 2H), 1.50-1.45 (m, 1H), 1.27 (s, 6H), 1.26 (s, 6H), 1.24 (s, 18H), 1.23 (s, 6H), 0.97 (d, *J* = 10.0 Hz, 1H);
^{13}C NMR (125 MHz, $CDCl_3$) δ 143.8, 128.4, 128.1, 125.3, 82.8, 82.8, 82.8, 35.7, 35.3, 25.1, 25.0, 24.9, 24.8, 24.7, 24.6 (*The carbons attached to borons were not observed due to quadrupolar relaxation*);
^{11}B NMR (161 MHz, $CDCl_3$) δ 34.2 (bs, 3B) (*One peak was missing due to the signal overlap*);
GC-MS (EI) for $C_{28}H_{47}B_3O_6$ Calcd: 512.4, found: 512.4;
HRMS (APCI, M+H$^+$) for $C_{28}H_{48}B_3O_6$ Calcd: 513.3725, found: 513.3716.

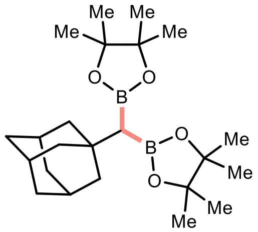

2,2'-(((3*r*,5*r*,7*r*)-Adamantan-1-yl)methylene)bis(4,4,5,5-tetramethyl-1,3,2-dioxaborolane) (**43b'**, condition B: 33%, 26.5 mg based on ^{1}H NMR) was prepared following the general procedure without modification and isolated by column chromatography as a coloerless oil. This compound is known, and its characterization data are consistent with the litrature report.[3]

^{1}H NMR (500 MHz, $CDCl_3$) δ 1.94 – 1.91 (m, 3H), 1.73 – 1.72 (m, 6H), 1.65 (s, 6H), 1.25 (s, 12H), 1.24 (s, 12H), 0.64 (s, 1H);
^{13}C NMR (125 MHz, $CDCl_3$) δ 82.6, 44.3, 37.1, 33.7, 29.3, 25.0, 24.6 (*The carbons attached to borons were not observed due to quadrupolar relaxation*);
^{11}B NMR (161 MHz, $CDCl_3$) δ 33.5 (bs, 2B);
GC-MS (EI) for $C_{23}H_{40}B_2O_4$ Calcd: 402.3, found: 402.2;
HRMS (APCI, M+H$^+$) for $C_{23}H_{41}B_2O_4$ Calcd: 403.3185, found: 403.3202.

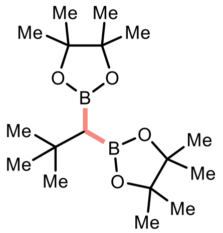

2,2'-(2,2-Dimethylpropane-1,1-diyl)bis(4,4,5,5-tetramethyl-1,3,2-dioxaborolane) (**44b'**, condition B: 29%, 18.8 mg based on ^{1}H NMR) was prepared following the general procedure without modification and isolated by column chromatography as a coloerless oil. This compound is known, and its characterization data are consistent with the litrature report.[2]
^{1}H NMR (500 MHz, $CDCl_3$) δ 1.25 (s, 12H), 1.24 (s, 12H), 1.08 (s, 9H), 0.79 (s, 1H);
^{13}C NMR (125 MHz, $CDCl_3$) δ 82.6, 31.9, 31.3, 24.9, 24.5 (*The carbons attached to borons were not observed due to quadrupolar relaxation*);
^{11}B NMR (161 MHz, $CDCl_3$) δ 33.4 (bs, 2B);
GC-MS (EI) for $C_{17}H_{34}B_2O_4$ Calcd: 324.3, found: 309.1 ($M^{\cdot+}$-Me).

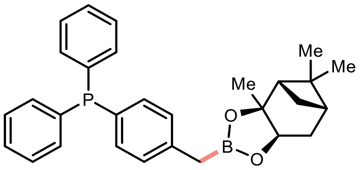

(Diphenyl(4-(((3a*S*,4*S*,6*S*,7a*R*)-3a,5,5-trimethylhexahydro-4,6-methanobenzo[*d*][1,3,2]dioxaborol-2-yl)methyl)phenyl)phosphane (**45g**, condition A: 44%, 40.0 mg) was prepared following the general procedure without modification and isolated by column chromatography as a coloerless oil. This compound is unknown in the literature.
^{1}H NMR (500 MHz, $CDCl_3$) δ 7.35 – 7.30 (m, 10H), 7.24 – 7.20 (m, 4H), 4.30 (dd, *J* = 8.7, 1.7 Hz, 1H), 2.37 (s, 2H), 2.36 – 2.31 (m, 1H), 2.22 – 2.17 (m, 1H), 2.06 (dd, *J* = 5.5, 5.5 Hz, 1H), 1.93 – 1.89 (m, 1H), 1.85 – 1.82 (m, 1H), 1.40 (s, 3H), 1.30 (s, 3H), 1.05 (d, *J* = 10.9 Hz, 1H), 0.85 (s, 3H);
^{13}C NMR (125 MHz, $CDCl_3$) δ 139.9, 137.6 (d, *J* = 10.5 Hz), 134.0 (d, *J* = 19.9 Hz), 133.7 (d, *J* = 19.2 Hz), 129.3 (d, *J* = 7.4 Hz), 128.5, 128.4 (d, *J* = 6.8 Hz), 85.9, 78.0, 51.3, 39.5, 38.2, 35.4, 28.6, 27.1, 26.4, 24.0 (*The carbon attached to boron was not observed due to quadrupolar relaxation and one aromatic carbon attaching to the phosphorus was missing due to the overlapping or low intensity*);
^{11}B NMR (161 MHz, $CDCl_3$) δ 32.5 (bs, 1B);
^{31}P NMR (202 MHz, $CDCl_3$) δ -6.2 (s, 1P);
GC-MS (EI) for $C_{29}H_{32}BO_2P$ Calcd: 454.2, found: 454.3;

HRMS (APCI, M+H$^+$) for $C_{29}H_{33}BO_2P$ Calcd: 455.2306, found: 455.2328.

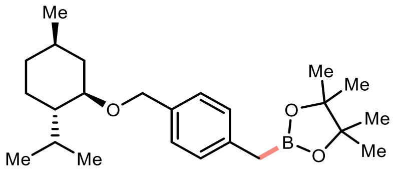

2-(4-((((1*R*,2*S*,5*R*)-2-Isopropyl-5-methylcyclohexyl)oxy)methyl)benzyl)-4,4,5,5-tetramethyl-1,3,2-dioxaborolane (**46b**, condition A: 40%, 30.9 mg) was prepared following the general procedure without modification and isolated by column chromatography as a colorless oil. This compound is known, and its characterization data are consistent with the litrature report.[25]

^{1}H NMR (500 MHz, $CDCl_3$) δ 7.24 (d, *J* = 8.1 Hz, 2H), 7.16 (d, *J* = 8.1 Hz, 2H), 4.62 (d, *J* = 11.2 Hz, 1H), 4.37 (d, *J* = 11.2 Hz, 1H), 3.17 (td, *J* = 10.6, 4.1 Hz, 1H), 2.34 – 2.30 (m, 1H), 2.29 (s, 2H), 2.23 – 2.18 (m, 1H), 1.69 – 1.61 (m, 2H), 1.41 – 1.26 (m, 2H), 1.25 (s, 12H), 0.96 – 0.90 (m, 9H), 0.71 (d, *J* = 6.9 Hz, 3H);

^{13}C NMR (125 MHz, $CDCl_3$) δ 137.8, 135.4, 128.9, 128.1, 83.4, 78.4, 70.4, 48.3, 40.3, 34.6, 31.6, 25.5, 24.7, 23.2, 22.4, 21.1, 16.0 (*The carbon attached to boron was not observed due to quadrupolar relaxation*);

^{11}B NMR (161 MHz, $CDCl_3$) δ 33.1 (bs, 1B);

GC-MS (EI) for $C_{24}H_{39}BO_3$ Calcd: 386.3, found: 386.2.

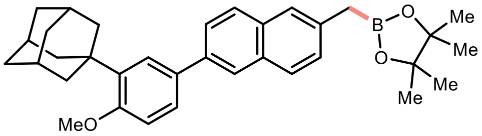

2-((6-(3-((3*r*,5*r*,7*r*)-Adamantan-1-yl)-4-methoxyphenyl)naphthalen-2-yl)methyl)-4,4,5,5-tetramethyl-1,3,2-dioxaborolane (**47b**, condition A: 39%, 39.6 mg) was prepared following the general procedure without modification and isolated by column chromatography as a colourless oil. This compound is unknown in the literature.

^{1}H NMR (500 MHz, $CDCl_3$) δ 7.95 (s, 1H), 7.81 (d, *J* = 5.5 Hz, 1H), 7.80 (d, *J* = 5.5 Hz, 1H), 7.70 (dd, *J* = 8.5, 1.8 Hz, 1H), 7.65 (s, 1H), 7.60 (d, *J* = 2.4 Hz, 1H), 7.54 (dd, *J* = 8.3, 2.3 Hz, 1H), 7.38 (dd, *J* = 8.4, 1.7 Hz, 1H), 7.01 (d, *J* = 8.4 Hz, 1H), 3.92 (s, 3H), 2.49 (s, 2H), 2.21 (s, 6H), 2.13 (s, 3H), 1.83 (s, 6H), 1.27 (s, 12H);

^{13}C NMR (125 MHz, $CDCl_3$) δ 158.4, 138.8, 137.9, 136.1, 133.5, 132.6, 131.8, 128.6, 127.9, 127.6, 126.4, 125.9, 125.6, 125.5, 124.8, 112.1, 83.5, 55.2, 52.4, 40.6, 37.2, 29.2, 24.8, 8.6 (*The carbon attached to boron was not observed due to quadrupolar relaxation*);

^{11}B NMR (161 MHz, $CDCl_3$) δ 33.4 (bs, 1B);

GC-MS (EI) for $C_{34}H_{41}BO_3$ Calcd: 508.3, *not found possibly due to the instability or non-volatility of the target compound*;

HRMS (APCI, M+H$^+$) for $C_{34}H_{42}BO_3$ Calcd: 509.3222, found: 509.3237.

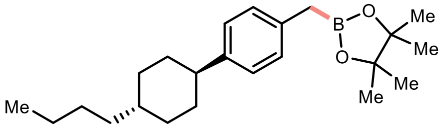

2-(4-((1*s*,4*r*)-4-Butylcyclohexyl)benzyl)-4,4,5,5-tetramethyl-1,3,2-dioxaborolane (**48b**, condition A: 47%, 33.5 mg) was prepared following the general procedure without modification and isolated by column chromatography as a colourless oil. This compound is unknown in the literature.

^{1}H NMR (500 MHz, $CDCl_3$) δ 7.13 (d, *J* = 8.2 Hz, 2H), 7.10 (d, *J* = 8.3 Hz, 2H), 2.43 (tt, *J* = 12.3, 3.3 Hz, 1H), 2.28 (s, 2H), 1.92 – 1.86 (m, 4H), 1.49 – 1.39 (m, 2H), 1.34 – 1.28 (m, 5H), 1.26 (s, 12H), 1.25 – 1.22 (m, 2H), 1.11 – 1.01 (m, 2H), 0.94 – 0.91 (m, 3H);

^{13}C NMR (125 MHz, $CDCl_3$) δ 144.3, 135.7, 128.9, 126.7, 83.4, 44.1, 37.4, 37.2, 34.4, 33.7, 29.3, 24.8, 23.0, 14.2 (*The carbon attached to boron was not observed due to quadrupolar relaxation*);

^{11}B NMR (161 MHz, $CDCl_3$) δ 33.2 (bs, 1B);

GC-MS (EI) for $C_{23}H_{37}BO_2$ Calcd: 356.3, found: 356.2;

HRMS (APCI, M+H$^+$) for $C_{23}H_{38}BO_2$ Calcd: 357.2959, found: 357.2968.

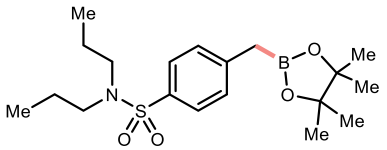

***N*,*N*-Dipropyl-4-((4,4,5,5-tetramethyl-1,3,2-dioxaborolan-2-yl)methyl)benzenesulfonamide** (**49b**, condition B: 33%, 25.1 mg based on ^{1}H NMR) was prepared following the general procedure without modification. For convenient isolation, the benzylboronate was converted to the corresponding alcohol (**43d**).

GC-MS (EI) for $C_{19}H_{32}BNO_4S$ Calcd: 381.2, found: 381.3.

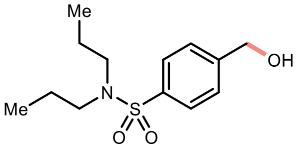

4-(Hydroxymethyl)-*N*,*N*-dipropylbenzenesulfonamide (**49d**) was prepared following the general procedure without modification. This compound is known, and its characterization data are consistent with the litrature report.[35]

^{1}H NMR (500 MHz, $CDCl_3$) δ 7.62 (d, *J* = 8.3 Hz, 2H), 7.38 (d, *J* = 8.4 Hz, 2H), 4.65 (s, 2H), 3.49 (s, 1H), 2.99 (t, *J* = 7.7 Hz, 4H), 1.48 (h, *J* = 7.4 Hz, 4H), 0.80 (t, *J* = 7.4 Hz, 6H);

^{13}C NMR (125 MHz, $CDCl_3$) δ 146.1, 138.3, 127.0, 126.9, 63.8, 50.0, 22.0, 11.1;

GC-MS (EI) for $C_{13}H_{21}NO_3S$ Calcd: 271.1, *not found possibily due to the instability or non-volatility of target compound.*

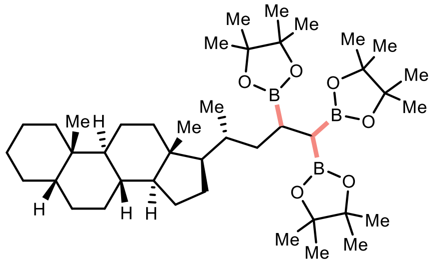

2,2',2''-((4*R*)-4-((5*S*,8*R*,9*S*,10*S*,13*R*,14*S*,17*R*)-10,13-Dimethylhexadecahydro-1*H*-cyclopenta[a]phenanthren-17-yl)pentane-1,1,2-triyl)tris(4,4,5,5-tetramethyl-1,3,2-dioxaborolane) (**50b**, condition A: 44%, 62.3 mg) was prepared following the general procedure without modification and isolated by column chromatography as a colourless oil. This compound is unknown in the literature.

^{1}H NMR (500 MHz, $CDCl_3$) δ 2.02 – 1.96 (m, 1H), 1.91 – 1.80 (m, 2H), 1.80 – 1.66 (m, 4H), 1.65 – 1.57 (m, 1H), 1.57 – 1.49 (m, 1H), 1.49 – 0.96 (m, 56H), 0.94 (d, *J* = 6.4 Hz, 3H), 0.92 (s, 3H), 0.80 (d, *J* = 10.7 Hz, 1H), 0.64 (s, 3H);

^{13}C NMR (125 MHz, $CDCl_3$) δ 82.8, 82.7, 82.5, 57.5, 56.7, 43.8, 42.8, 41.1, 40.5, 40.4, 37.6, 35.9, 35.7, 35.4, 28.2, 27.6, 27.3, 27.1, 26.6, 25.2, 25.0, 24.9, 24.9, 24.6, 24.5, 24.3, 24.3, 21.4, 20.9, 19.0, 12.1 (*The carbon attached to boron was not observed due to quadrupolar relaxation*);

^{11}B NMR (161 MHz, $CDCl_3$) δ 34.2 (bs, 3B) (*One peak was missing due to the signal overlap*);

GC-MS (EI) for $C_{42}H_{75}B_3O_6$ Calcd: 708.6, *not found possibily due to the instability or non-volatility of target compound;*

HRMS (APCI, M+H$^+$) for $C_{42}H_{76}B_3O_6$ Calcd: 709.5916, found: 709.5946.

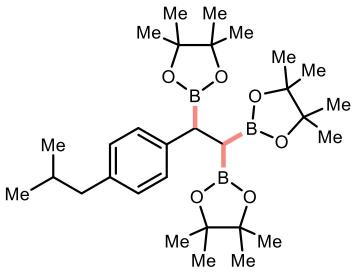

2,2',2''-(2-(4-Isobutylphenyl)ethane-1,1,2-triyl)tris(4,4,5,5-tetramethyl-1,3,2-dioxaborolane) (**51b**, condition A: 48%, 51.8 mg) was prepared following the general procedure without modification and isolated by column chromatography as a colourless oil. This compound is unknown in the literature.

^{1}H NMR (500 MHz, $CDCl_3$) δ 7.12 (d, *J* = 8.1 Hz, 2H), 6.96 (d, *J* = 8.0 Hz, 2H), 2.65 (d, *J* = 12.8 Hz, 1H), 2.39 (dd, *J* = 7.0, 1.8 Hz, 2H), 1.84 – 1.71 (m, 1H), 1.45 (d, *J* = 12.8 Hz, 1H), 1.26 (s, 6H), 1.24 (s, 6H), 1.17 (s, 6H), 1.15 (s, 6H), 0.96 (s, 6H), 0.95 (s, 6H), 0.86 (dd, *J* = 6.6, 4.7 Hz, 6H).

^{13}C NMR (125 MHz, $CDCl_3$) δ 142.4, 137.7, 128.6, 128.2, 83.0, 83.0, 82.6, 45.1, 30.3, 25.0, 24.8, 24.7, 24.4, 24.4, 24.2, 22.3, 22.2 (*The carbon attached to boron was not observed due to quadrupolar relaxation*);

^{11}B NMR (161 MHz, $CDCl_3$) δ 33.9 (bs, 3B) (*One peak was missing due to the signal overlap*);

GC-MS (EI) for $C_{30}H_{51}B_3O_6$ Calcd: 540.4, found: 525.5 ($M^{\cdot+}$-Me);

HRMS (APCI, M+H$^+$) for $C_{30}H_{52}B_3O_6$ Calcd: 514.4038, found:514.4038.

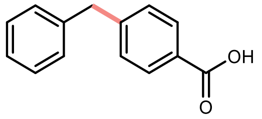

4-Benzylbenzoic acid (**52a**, 50%, 21.1 mg) was prepared following the general procedure **4.3.7** and isolated by column chromatography as a colourless oil. This compound is known, and its characterization data are consistent with the literature report.[36]

^{1}H NMR (500 MHz, CD_3OD) δ 7.95 (d, *J* = 8.3 Hz, 2H), 7.37 – 7.26 (m, 4H), 7.25 – 7.17 (m, 3H), 4.05 (s, 2H);

^{13}C NMR (125 MHz, CD_3OD) δ 168.5, 147.0, 140.4, 129.6, 128.6, 128.6, 128.3, 128.2, 125.9, 41.3;

HRMS (APCI, M-H⁺) for $C_{14}H_{11}O_2$ Calcd: 211.0765, found: 211.0759.

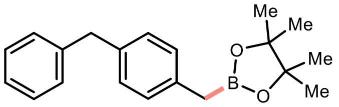

2-(4-Benzylbenzyl)-4,4,5,5-tetramethyl-1,3,2-dioxaborolane (**52b**, condition A: 50%, 30.8 mg) was prepared following the general procedure without modification and isolated by column chromatography as a colourless oil. This compound is unknown in the literature.

^{1}H NMR (500 MHz, $CDCl_3$) δ 7.33 – 7.28 (m, 2H), 7.25 – 7.19 (m, 3H), 7.14 (d, *J* = 8.0 Hz, 2H), 7.09 (d, *J* = 8.0 Hz, 2H), 3.96 (s, 2H), 2.29 (s, 2H), 1.26 (s, 12H);

^{13}C NMR (125 MHz, $CDCl_3$) δ 141.5, 137.5, 136.2, 129.1, 129.0, 128.9, 128.4, 125.9, 83.4, 41.5, 24.8 (*The carbon attached to boron was not observed due to quadrupolar relaxation*);

^{11}B NMR (161 MHz, $CDCl_3$) δ 33.1 (bs, 1B);

GC-MS (EI) for $C_{20}H_{25}BO_2$ Calcd: 308.2, found: 308.3;

HRMS (APCI, M+H⁺) for $C_{20}H_{26}BO_2$ Calcd: 309.2020, found: 309.2034.

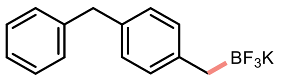

(4-Benzylbenzyl)trifluoro-λ^4-borane, potassium salt (**52l**) was prepared from the corresponding benzylboronic pinacol ester via the reaction with saturated KHF_2 aqueous solution and recrystallization in acetone and Et_2O.[10] This compound is unknown in the literature.

^{1}H NMR (500 MHz, DMSO-*d*$_6$) δ 7.26 (t, J = 2H), 7.19 (d, *J* = 6.6 Hz, 2H), 7.15 (t, *J* = 7.2 Hz, 1H), 6.89 (s, 4H), 3.81 (s, 2H), 1.40 (d, *J* = 6.3 Hz, 2H);

^{13}C NMR (125 MHz, DMSO-*d*$_6$) δ 144.9, 142.6, 134.9, 129.1, 129.0, 128.7, 127.8, 126.1, 41.3 (*The carbon attached to boron was not observed due to quadrupolar relaxation*);

^{11}B NMR (161 MHz, DMSO-*d*$_6$) δ 4.0 (bs, 1B);

^{19}F NMR (471 MHz, DMSO-*d*$_6$) δ 136.54 (bs, 3F);

HRMS (ESI, M-K⁺) for $C_{14}H_{13}BF_3$ Calcd: 249.1068, found: 249.1067.

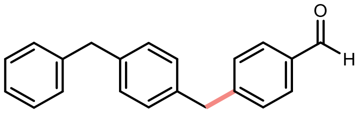

4-(4-Benzylbenzyl)benzaldehyde (**53c**, 66%, 37.8 mg) was prepared following the general procedure **4.3.7** and isolated by column chromatography as a colourless oil. This compound is known, and its characterization data are consistent with the literature report.[37]

^{1}H NMR (500 MHz, $CDCl_3$) δ 10.00 (s, 1H), 7.82 (d, *J* = 8.2 Hz, 2H), 7.37 (d, *J* = 8.0 Hz, 2H), 7.31 (t, *J* = 7.4 Hz, 2H), 7.26 – 7.19 (m, 3H), 7.16 (d, *J* = 8.2 Hz, 2H), 7.12 (d, *J* = 8.2 Hz, 2H), 4.05 (s, 2H), 3.98 (s, 2H);

^{13}C NMR (126 MHz, $CDCl_3$) δ 192.0, 148.6, 141.0, 139.4, 137.5, 134.7, 130.1, 129.6, 129.2, 129.1, 128.9, 128.5, 126.1, 41.7, 41.6;

GC-MS (EI) for $C_{21}H_{18}O$ Calcd: 286.1, found: 286.1;

HRMS (APCI, M+H⁺) for $C_{21}H_{19}O$ Calcd: 287.1430, found: 287.1441.

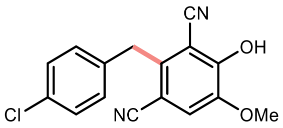

2-(4-Chlorobenzyl)-4-hydroxy-5-methoxyisophthalonitrile (**54f**, 48%, 28.6 mg) was prepared following the general procedure **4.3.8** and isolated by column chromatography as a colourless oil. This compound is known, and its characterization data are consistent with the litrature report.[38]

^{1}H NMR (500 MHz, CD_3OD) δ 7.46 (s, 1H), 7.31 (d, *J* = 8.6 Hz, 2H), 7.26 (d, *J* = 8.7 Hz, 2H), 4.26 (s, 2H), 3.95 (s, 3H) (*Phenolic proton was not observed*);

^{13}C NMR (125 MHz, CD_3OD) δ 147.4, 140.7, 136.7, 132.4, 129.7, 128.3, 128.1, 128.0, 117.3, 117.3, 114.2, 102.6, 55.8, 37.1;

HRMS (ESI, M-H$^+$) for $C_{16}H_{10}N_2O_2Cl$ Calcd: 297.0436 (100%), 299.0401 (32%), found: 297.0446 (100%), 299.0409 (32%).

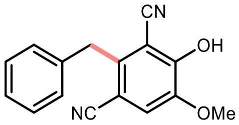

2-Benzyl-4-hydroxy-5-methoxyisophthalonitrile (**55f**, 46%, 24.3 mg) was prepared following the general procedure **4.3.8** and isolated by column chromatography as a colourless oil. This compound is known, and its characterization data are consistent with the litrature report.[38]

^{1}H NMR (500 MHz, CD_3OD) δ 7.45 (bs, 1H), 7.37 – 7.19 (m, 6H), 4.26 (s, 2H), 3.95 (bs, 3H);

^{13}C NMR (125 MHz, CD_3OD) δ 147.2, 141.4, 137.9, 128.2, 128.1, 127.9, 126.9, 126.6, 126.5, 117.3, 114.2, 102.6, 63.8, 37.9;

HRMS (ESI, M-H$^+$) for $C_{16}H_{11}N_2O_2$ Calcd: 263.0826, found: 263.0834.

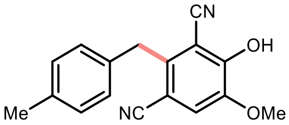

4-Hydroxy-5-methoxy-2-(4-methylbenzyl)isophthalonitrile (**56f**, 49%, 27.2 mg) was prepared following the general procedure **4.3.8** and isolated by column chromatography as a colourless oil. This compound is known, and its characterization data are consistent with the litrature report.[38]

^{1}H NMR (500 MHz, CD_3OD) δ 7.44 (s, 1H), 7.16 (d, *J* = 8.1 Hz, 2H), 7.11 (d, *J* = 8.0 Hz, 2H), 4.22 (s, 2H), 3.94 (s, 3H), 2.31 (s, 3H) (*Phenolic proton was not observed*);

^{13}C NMR (125 MHz, CD_3OD) δ 155.8, 147.1, 141.7, 136.2, 134.9, 128.8, 128.0, 117.4, 114.2, 102.6, 101.3, 55.8, 37.4, 19.6 (*One aromatic carbon was missing due to the overlapping*);

HRMS (ESI, M-H$^+$) for $C_{17}H_{13}N_2O_2$ Calcd: 277.0983, found: 277.0990.

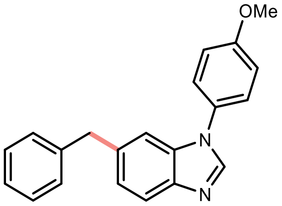

6-Benzyl-1-(4-methoxyphenyl)-1*H*-benzo[*d*]imidazole (**57f**, 53%, 33.3 mg) was prepared following the general procedure **4.3.8** but with 3.0 equiv R-Bpin and isolated by column chromatography as a colourless oil. This compound is unknown in the literature.

^{1}H NMR (500 MHz, $CDCl_3$) δ 8.03 (s, 1H), 7.80 (d, *J* = 8.2 Hz, 1H), 7.41 (d, *J* = 7.5 Hz, 2H), 7.33 – 7.25 (m, 3H), 7.23 – 7.17 (m, 4H), 7.09 (d, *J* = 7.5 Hz, 2H), 4.12 (s, 2H), 3.91 (s, 3H);

^{13}C NMR (125 MHz, $CDCl_3$) δ 159.3, 142.5, 141.5, 136.9, 134.5, 129.2, 128.8, 128.5, 126.1, 125.8, 125.7, 124.2, 120.3, 115.1, 110.4, 55.7, 42.3;

GC-MS (EI) for $C_{21}H_{18}N_2O$ Calcd: 314.1, found: 314.2;

HRMS (ESI, M+H$^+$) for $C_{21}H_{19}N_2O$ Calcd: 315.1492, found: 315.1492.

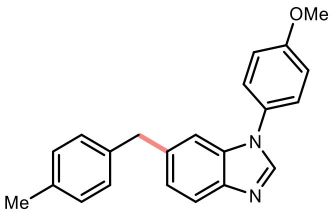

1-(4-Methoxyphenyl)-6-(4-methylbenzyl)-1*H*-benzo[*d*]imidazole (**58f**, 59%, 38.7 mg) was prepared following the general procedure **4.3.8** but with 3.0 equiv R-Bpin and isolated by column chromatography as a colourless oil. This compound is known, and its characterization data are consistent with the litrature report.[39]

^{1}H NMR (500 MHz, $CDCl_3$) δ 8.02 (s, 1H), 7.79 (d, *J* = 8.4 Hz, 1H), 7.42 (d, *J* = 8.9 Hz, 2H), 7.31 (dd, *J* = 1.6, 0.8 Hz, 1H), 7.19 (dd, *J* = 8.3, 1.6 Hz, 1H), 7.11 – 7.07 (m, 6H), 4.08 (s, 2H), 3.91 (s, 3H), 2.32 (s, 3H);

^{13}C NMR (125 MHz, $CDCl_3$) δ 159.3, 142.5, 138.5, 137.2, 135.6, 134.5, 129.3, 129.2, 128.6, 125.8, 125.7, 124.2, 120.3, 115.1, 110.3, 55.7, 41.9, 21.0;

GC-MS (EI) for $C_{22}H_{20}N_2O$ Calcd: 328.2, found: 328.3;

HRMS (ESI, M+H$^+$) for $C_{22}H_{21}N_2O$ Calcd: 328.1648, found: 328.1650.

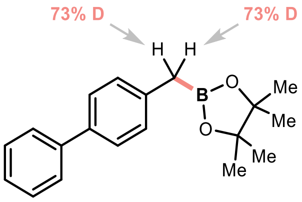

2-([1,1'-Biphenyl]-4-ylmethyl)-4,4,5,5-tetramethyl-1,3,2-dioxaborolane-*d*$_{73\%}$ (**1b-*d*$_{73\%}$**, condition according to **Section 5.3.1.**: 29%, 17.1 mg based on ^{1}H NMR) was prepared following the general procedure without modification and isolated by column chromatography as a colourless oil. This compound is unknown in the literature.

^{1}H NMR (500 MHz, $CDCl_3$) δ 7.61 – 7.59 (m, 2H), 7.50 (d, *J* = 8.3 Hz, 2H), 7.44 (t, *J* = 7.7 Hz, 2H), 7.33 (tt, *J* = 6.9, 1.2 Hz, 1H), 7.28 (d, *J* = 8.3 Hz, 2H), 2.36 – 2.34 (m, 0.55H), 1.28 (s, 12H);

^{13}C NMR (125 MHz, $CDCl_3$) δ 141.3, 137.8, 137.8, 129.4, 128.7, 127.0, 126.9, 126.8, 83.5, 24.8 (*The carbon attached to boron was not observed due to quadrupolar relaxation*);

^{11}B NMR (161 MHz, $CDCl_3$) δ 33.2 (bs, 1B);

^{2}H NMR (77 MHz, None) δ 2.29 (s, 1.46D);

GC-MS (EI) for $C_{19}H_{23}BO_2$ Calcd: 294.2, found: 294.1; for $C_{19}H_{22}DBO_2$ Calcd: 295.2, found: 295.1; for $C_{19}H_{21}D_2BO_2$ Calcd: 296.2, found: 296.1;
HRMS (APCI, M+H$^+$) for $C_{19}H_{24}BO_2$ Calcd: 295.1869, found: 295.1890; for $C_{19}H_{23}DBO_2$ Calcd: 296.1932, found: 296.1933; for $C_{19}H_{22}D_2BO_2$ Calcd: 297.1995, found: 297.1989.

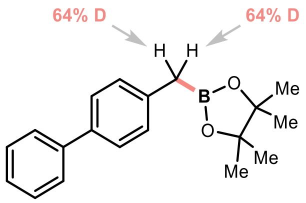

2-([1,1'-Biphenyl]-4-ylmethyl)-4,4,5,5-tetramethyl-1,3,2-dioxaborolane-*d*$_{64\%}$ (**1b-*d*$_{64\%}$,** condition according to **Section 5.3.1.**: 47%, 27.7 mg based on ^{1}H NMR) was prepared following the general procedure without modification and isolated by column chromatography as a colourless oil. This compound is unknown in the literature.
^{1}H NMR (500 MHz, $CDCl_3$) δ 7.61 – 7.59 (m, 2H), 7.50 (d, *J* = 8.3 Hz, 2H), 7.44 (t, *J* = 7.7 Hz, 2H), 7.33 (tt, *J* = 6.9, 1.2 Hz, 1H), 7.28 (d, *J* = 8.1 Hz, 2H), 2.36 – 2.34 (m, 0.72H), 1.28 (s, 12H);
^{13}C NMR (125 MHz, $CDCl_3$) δ 141.3, 137.8, 137.8, 129.4, 128.7, 127.0, 126.9, 126.8, 83.5, 24.8 (*The carbon attached to boron was not observed due to quadrupolar relaxation*);
^{11}B NMR (161 MHz, $CDCl_3$) δ 33.2 (bs, 1B);
^{2}H NMR (77 MHz, $CDCl_3$) δ 2.30 (s, 1.28D);
GC-MS (EI) for $C_{19}H_{23}BO_2$ Calcd: 294.2, found: 294.1; for $C_{19}H_{22}DBO_2$ Calcd: 295.2, found: 295.1; for $C_{19}H_{21}D_2BO_2$ Calcd: 296.2, found: 296.1;
HRMS (APCI, M+H$^+$) for $C_{19}H_{24}BO_2$ Calcd: 295.1869, found: 295.1872; for $C_{19}H_{23}DBO_2$ Calcd: 296.1932, found: 296.1923; for $C_{19}H_{22}D_2BO_2$ Calcd: 297.1995, found: 297.1978.

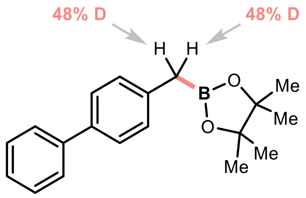

2-([1,1'-Biphenyl]-4-ylmethyl)-4,4,5,5-tetramethyl-1,3,2-dioxaborolane-*d*$_{48\%}$ (**1b-*d*$_{48\%}$,** condition according to **Section 5.3.2.2.**: 53%, 31.3 mg based on ^{1}H NMR) was prepared following the general procedure without modification and isolated by column chromatography as a coloerless oil. This compound is unknown in the literature.
^{1}H NMR (500 MHz, $CDCl_3$) δ 7.61 (dd, *J* = 8.2, 1.1 Hz, 2H), 7.51 (d, *J* = 8.2 Hz, 2H), 7.43 (d, *J* = 8.0 Hz, 2H), 7.35 – 7.32 (m, 1H), 7.28 (d, *J* = 8.1 Hz, 2H), 2.35 (m, 1H), 1.28 (s, 12H);
^{13}C NMR (125 MHz, $CDCl_3$) δ 141.3, 137.8, 137.8, 129.4, 128.7, 127.0, 126.9, 126.8, 83.5, 24.8 (*The carbon attached to boron was not observed due to quadrupolar relaxation*);
^{11}B NMR (161 MHz, $CDCl_3$) δ 33.2 (bs, 1B);
^{2}H NMR (77 MHz, $CDCl_3$) δ 2.30 (s, 0.96D);
GC-MS (EI) for $C_{19}H_{23}BO_2$ Calcd: 294.2, found: 294.1; for $C_{19}H_{22}DBO_2$ Calcd: 295.2, found: 295.1; for $C_{19}H_{21}D_2BO_2$ Calcd: 296.2, found: 296.1;

HRMS (APCI, M+H$^+$) for $C_{19}H_{24}BO_2$ Calcd: 295.1869, found: 295.1880; for $C_{19}H_{23}DBO_2$ Calcd: 296.1932, found: 296.1934; for $C_{19}H_{22}D_2BO_2$ Calcd: 297.1995, found: 297.1988.

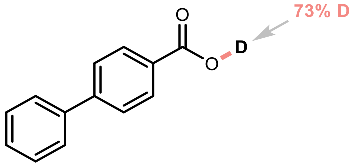

[1,1'-Biphenyl]-4-carboxylic acid-*d* (**1a-*d***) was prepared following the general procedure in **Section 5.3.2.1.**. This compound is unknown in the literature.

^{1}H NMR (500 MHz, $CDCl_3$) δ 11.88 (s, 0.27H), 8.22 (d, *J* = 8.1 Hz, 2H), 7.74 (d, *J* = 8.1 Hz, 2H), 7.67 (d, *J* = 7.1 Hz, 2H), 7.51 (t, *J* = 7.4 Hz, 2H), 7.44 (t, *J* = 7.4 Hz, 1H);

^{13}C NMR (125 MHz, $CDCl_3$) δ 171.1, 146.6, 139.9, 130.8, 129.0, 128.3, 127.9, 127.3, 127.2;

^{2}H NMR (77 MHz, $CDCl_3$) δ 11.6 (s, 0.73D);

HRMS (APCI, M+H$^+$) for $C_{13}H_9O_2D$ Calcd: 199.0754, found:199.0762.

Supplementary Figure 1b-1 ^{1}H NMR (500 mHz, $CDCl_3$) 2-([1,1'-Biphenyl]-4-ylmethyl)-4,4,5,5-tetramethyl-1,3,2-dioxaborolane

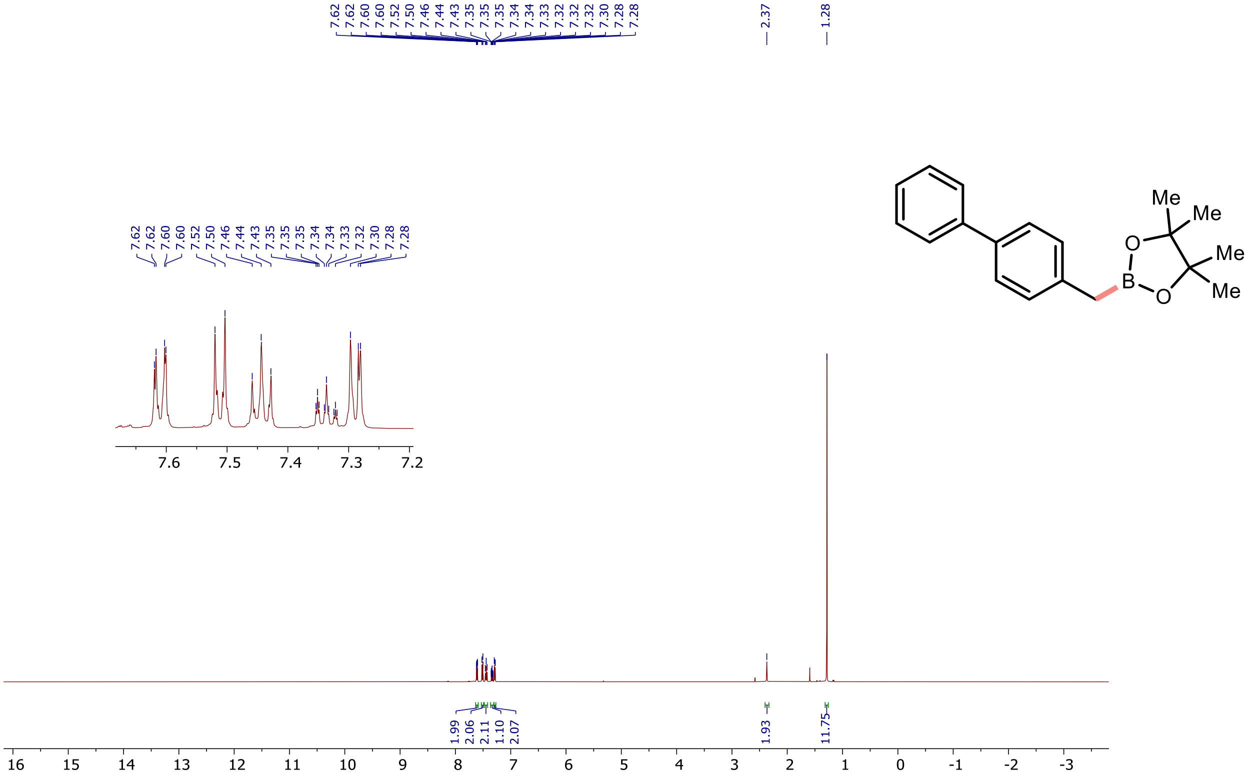

Supplementary Figure 1b-2 ^{13}C NMR (125 mHz, $CDCl_3$) 2-([1,1'-Biphenyl]-4-ylmethyl)-4,4,5,5-tetramethyl-1,3,2-dioxaborolane

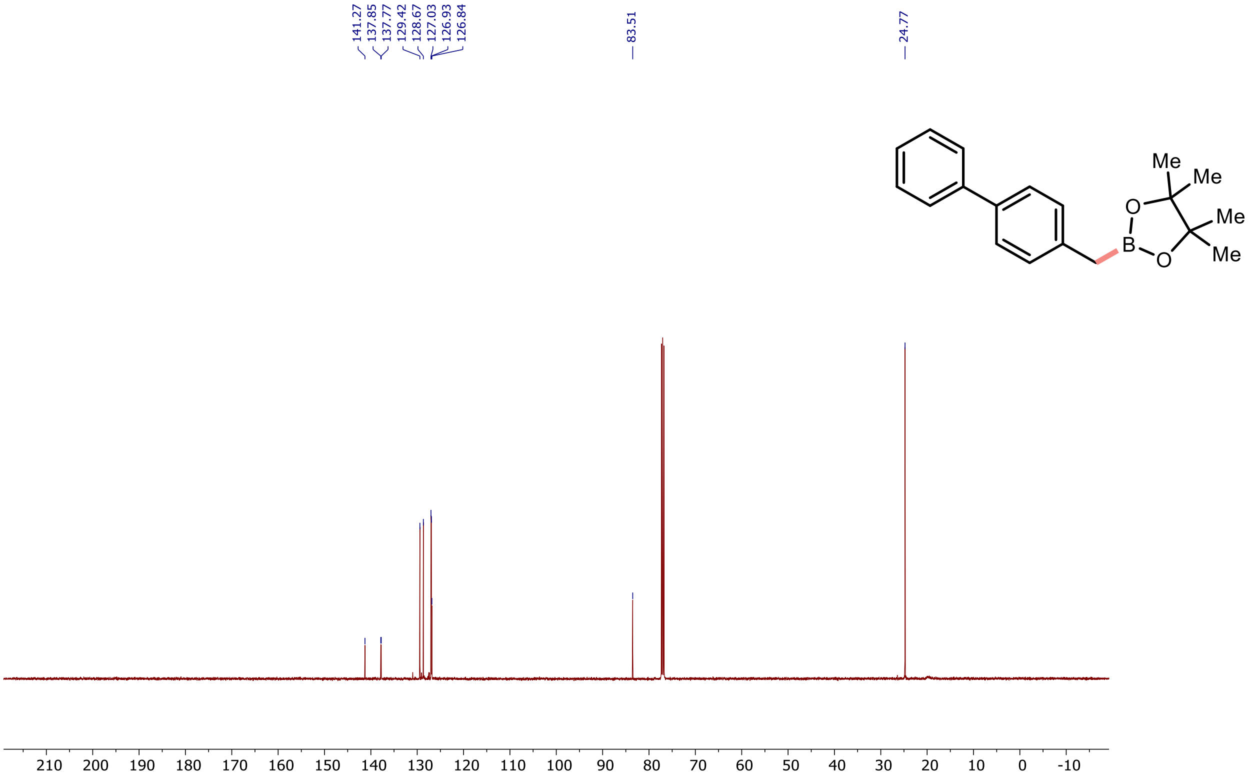

Supplementary Figure 1b-3 ^{11}B NMR (161 mHz, $CDCl_3$) 2-([1,1'-Biphenyl]-4-ylmethyl)-4,4,5,5-tetramethyl-1,3,2-dioxaborolane

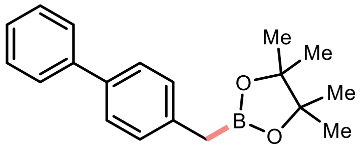

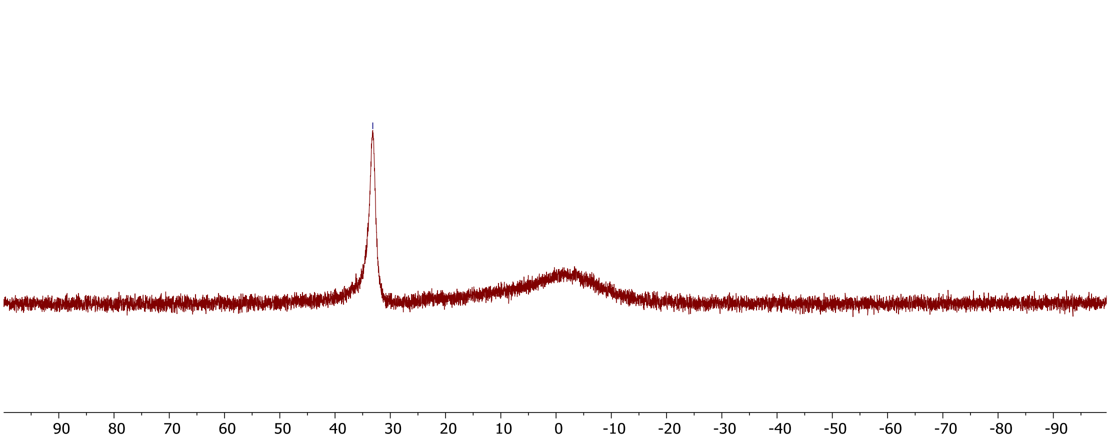

Supplementary Figure 1f-1 ^{1}H NMR (500 mHz, $CDCl_3$) (3a*R*,6a*S*)-2-([1,1'-Biphenyl]-4-ylmethyl)tetrahydro-4*H*-cyclopenta[*d*][1,3,2]dioxaborole

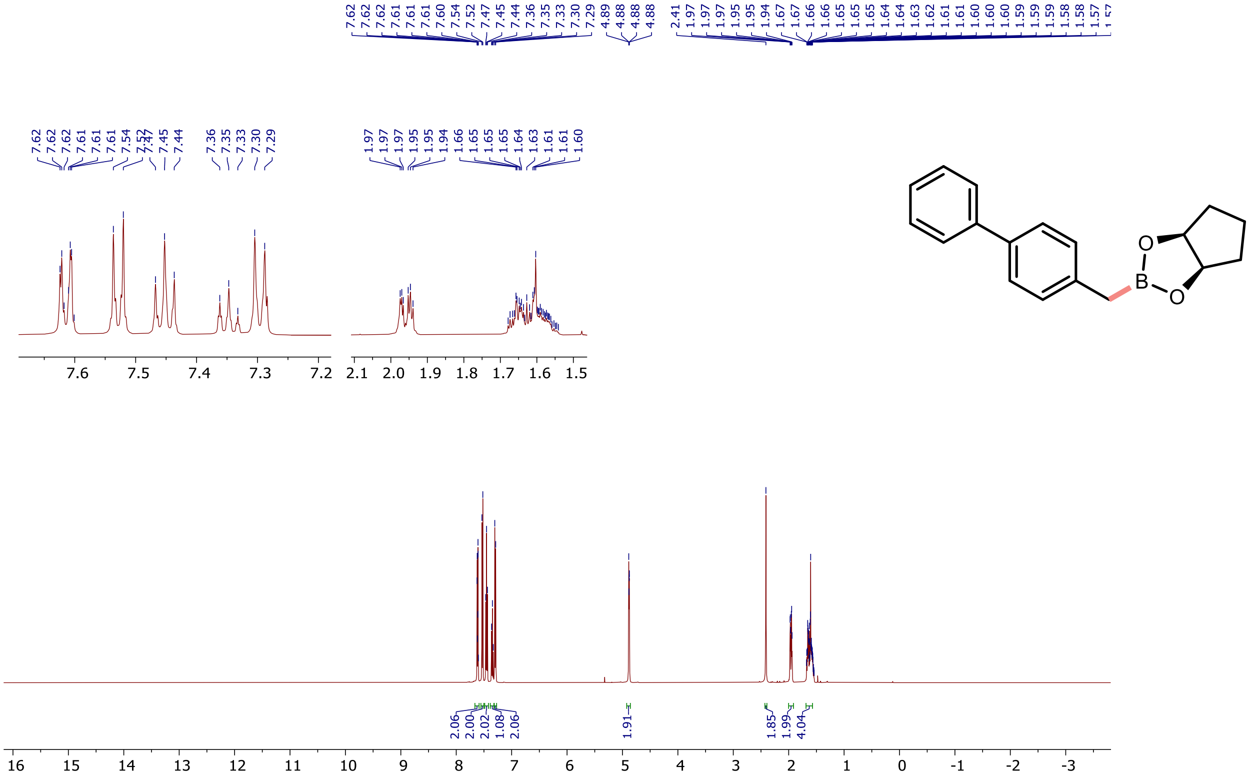

Supplementary Figure 1f-2 ^{13}C NMR (125 mHz, $CDCl_3$) (3a*R*,6a*S*)-2-([1,1'-Biphenyl]-4-ylmethyl)tetrahydro-4*H*-cyclopenta[*d*][1,3,2]dioxaborole

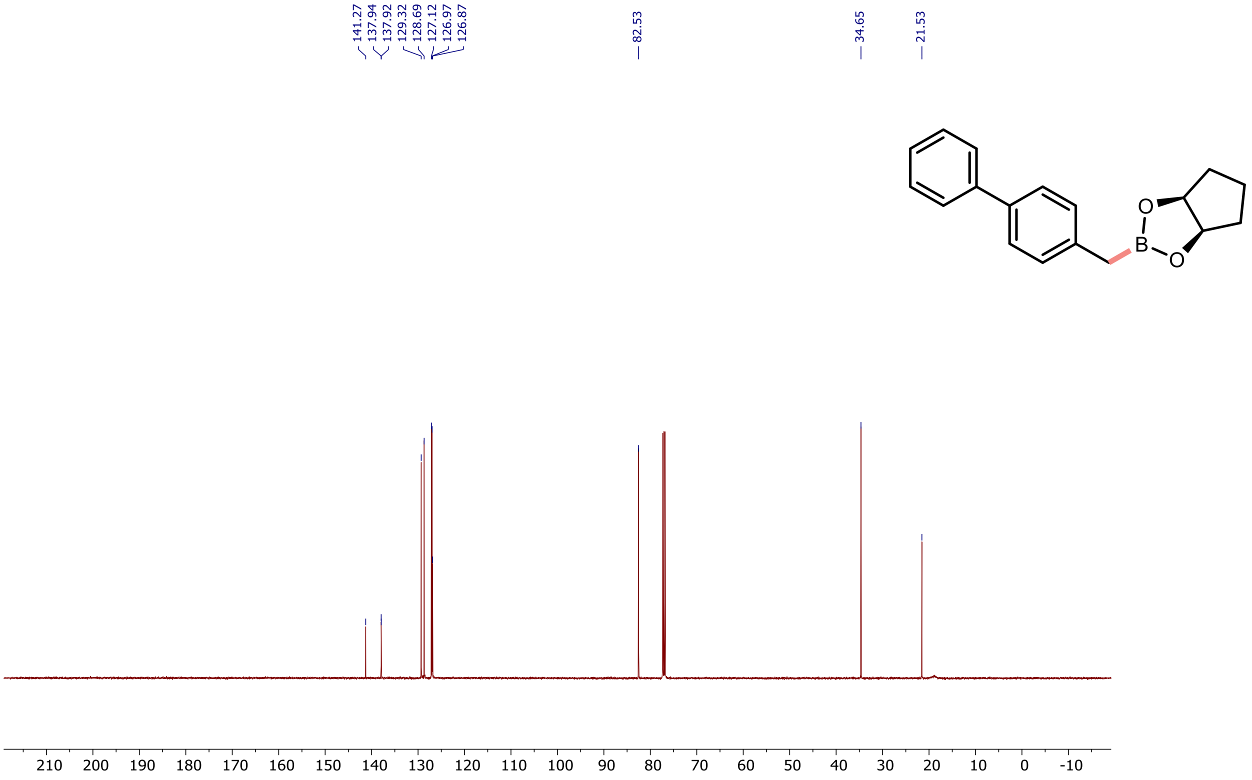

Supplementary Figure 1f-3 ^{11}B NMR (161 mHz, $CDCl_3$) (3a*R*,6a*S*)-2-([1,1'-Biphenyl]-4-ylmethyl)tetrahydro-4*H*-cyclopenta[*d*][1,3,2]dioxaborole

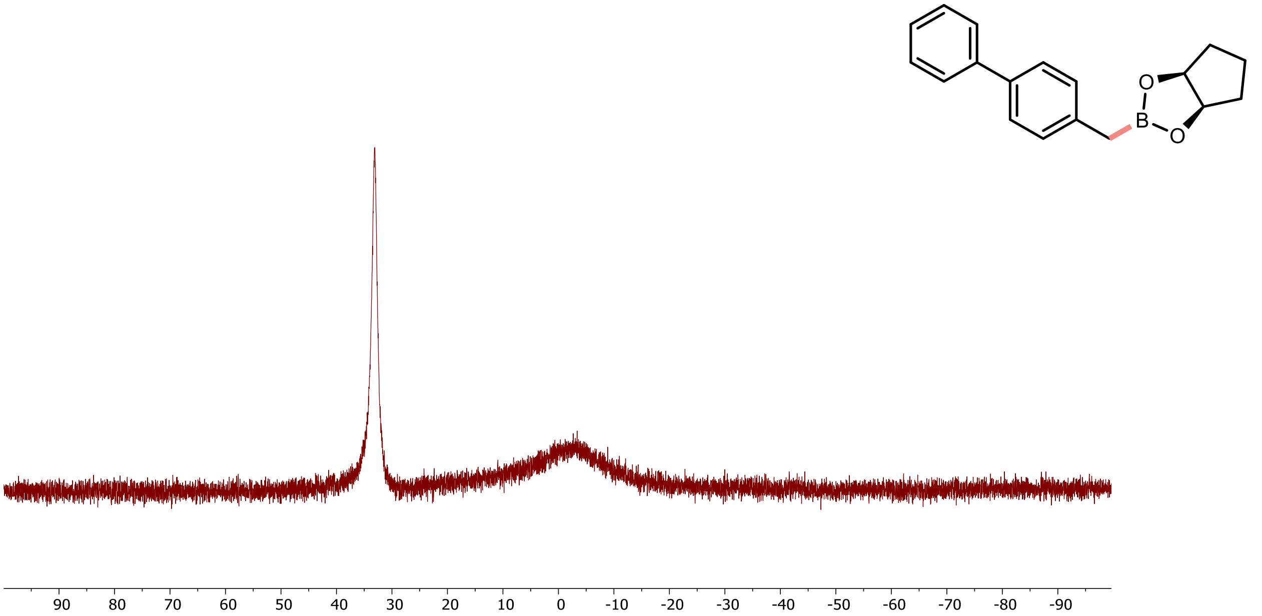

Supplementary Figure 1g-1 ^{1}H NMR (500 mHz, $CDCl_3$) (3a*S*,4*S*,6*S*,7a*R*)-2-([1,1'-Biphenyl]-4-ylmethyl)-3a,5,5-trimethylhexahydro-4,6-methanobenzo[d][1,3,2]dioxaborole

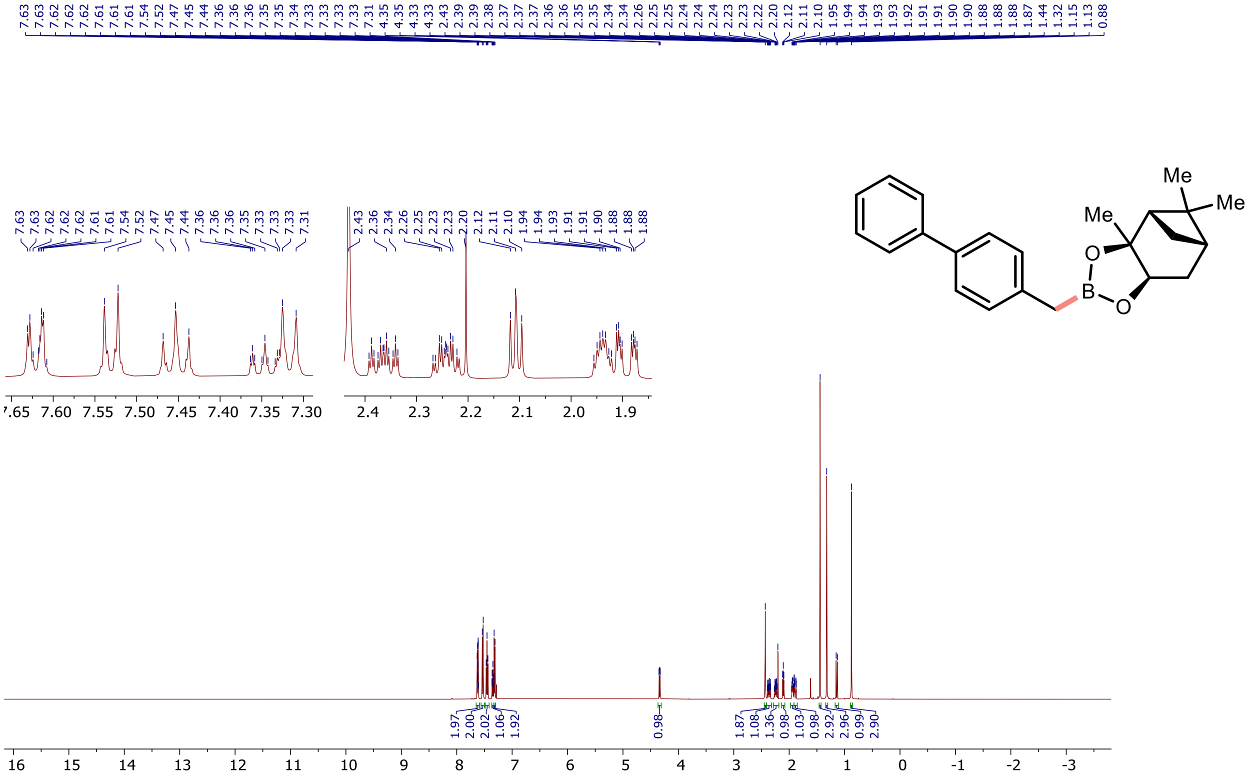

Supplementary Figure 1g-2 ^{13}C NMR (125 mHz, $CDCl_3$) (3a*S*,4*S*,6*S*,7a*R*)-2-([1,1'-Biphenyl]-4-ylmethyl)-3a,5,5-trimethylhexahydro-4,6-methanobenzo [d][1,3,2]dioxaborole

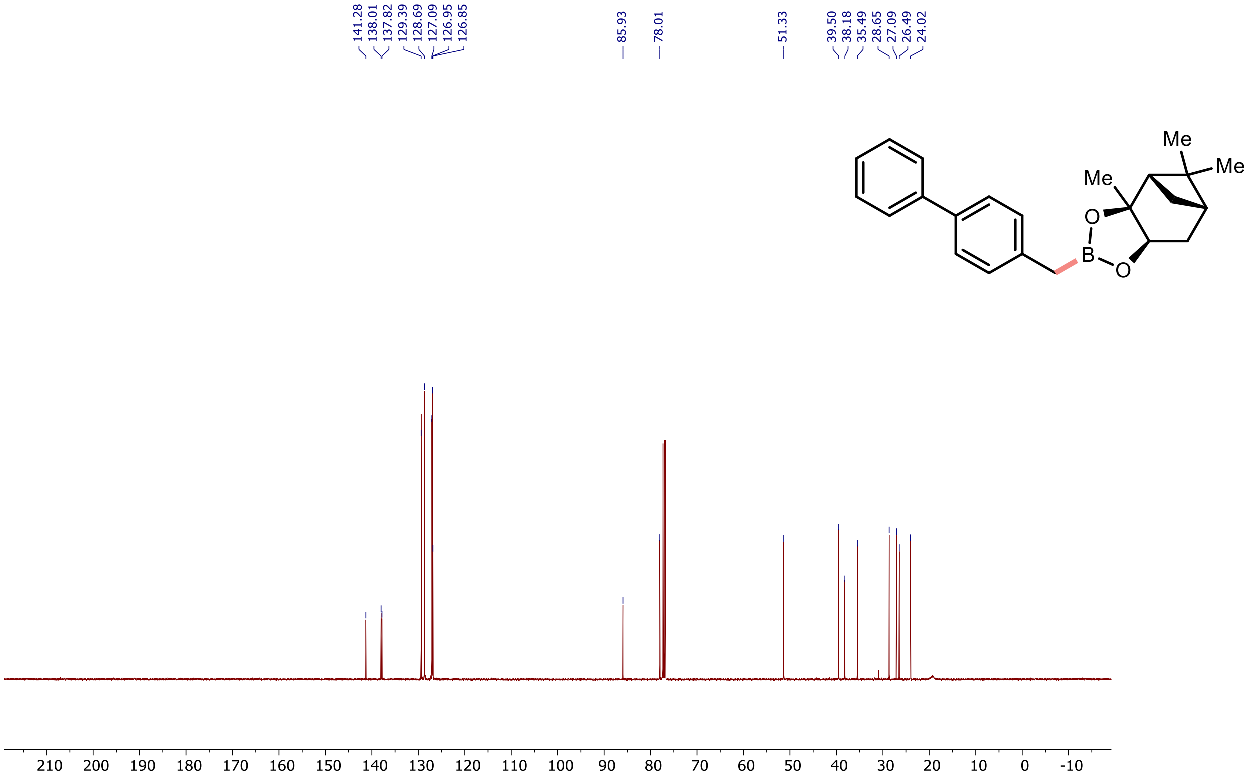

Supplementary Figure 1g-3 ^{11}B NMR (161 mHz, $CDCl_3$) (3a*S*,4*S*,6*S*,7a*R*)-2-([1,1'-Biphenyl]-4-ylmethyl)-3a,5,5-trimethylhexahydro-4,6-methanobenzo[d][1,3,2]dioxaborole

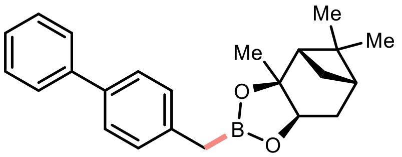

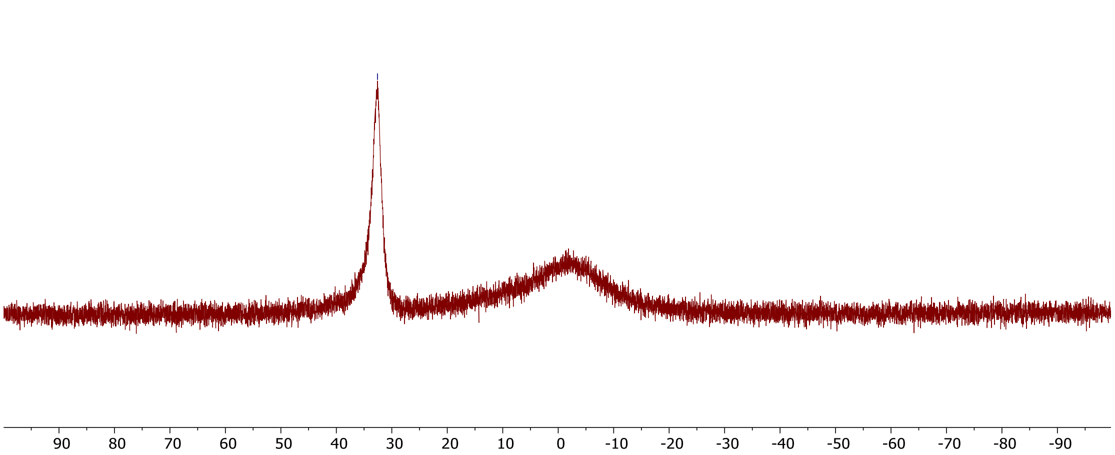

Supplementary Figure 1h-1 ^{1}H NMR (500 MHz, $CDCl_3$) 2-([1,1'-Biphenyl]-4-ylmethyl)-4,4,6-trimethyl-1,3,2-dioxaborinane

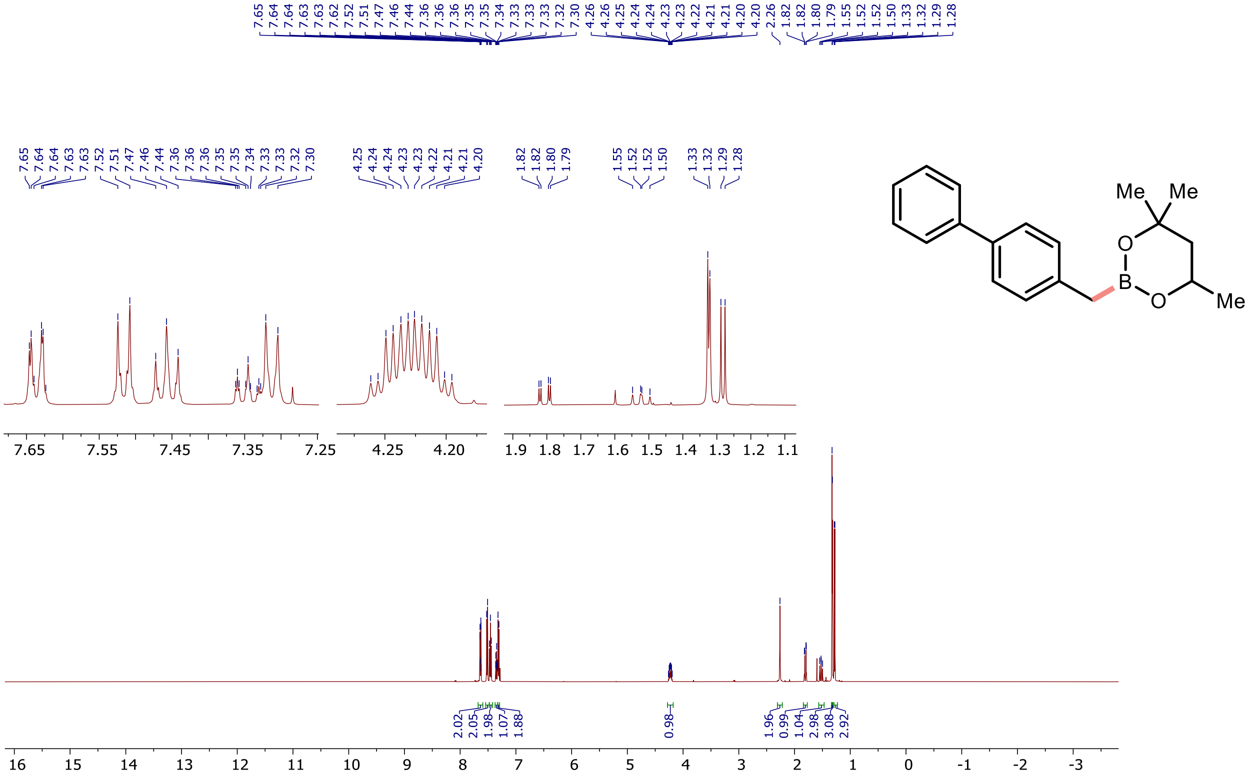

Supplementary Figure 1h-2 ^{13}C NMR (125 MHz, $CDCl_3$) 2-([1,1'-Biphenyl]-4-ylmethyl)-4,4,6-trimethyl-1,3,2-dioxaborinane

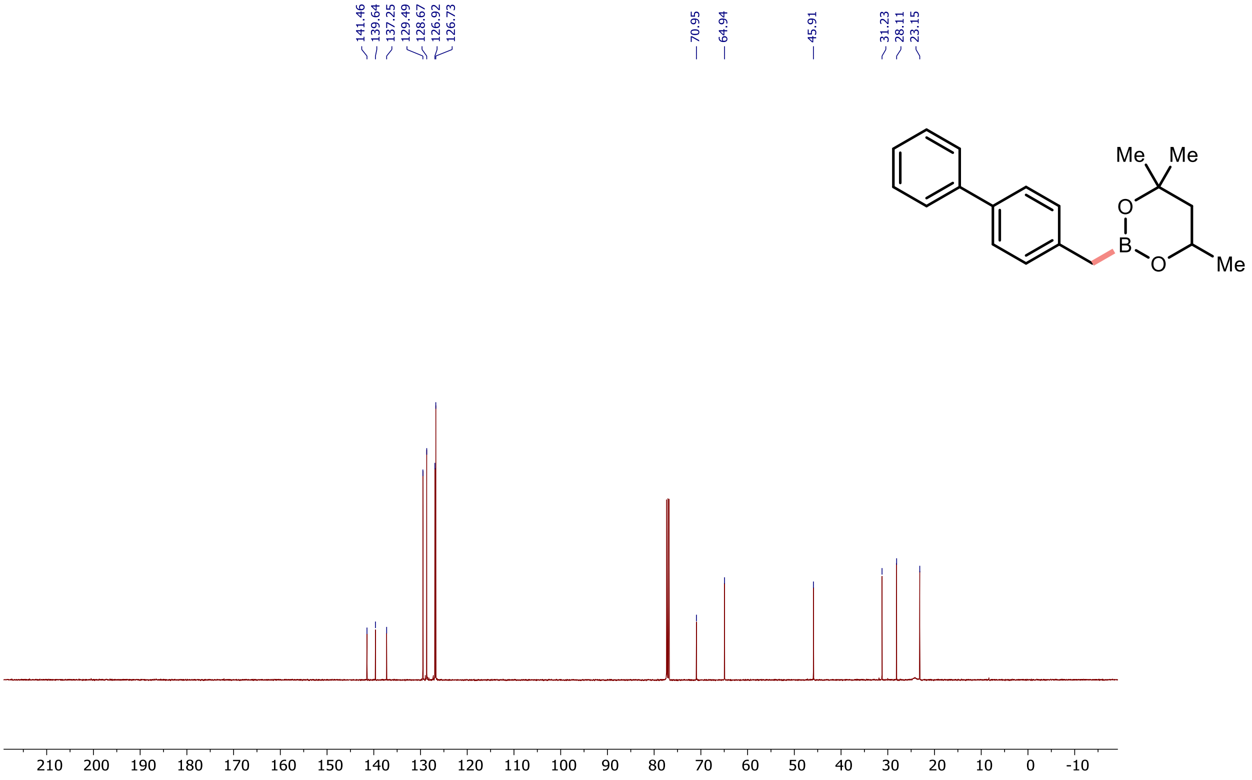

Supplementary Figure 1h-3 ^{11}B NMR (161 MHz, $CDCl_3$) 2-([1,1'-Biphenyl]-4-ylmethyl)-4,4,6-trimethyl-1,3,2-dioxaborinane

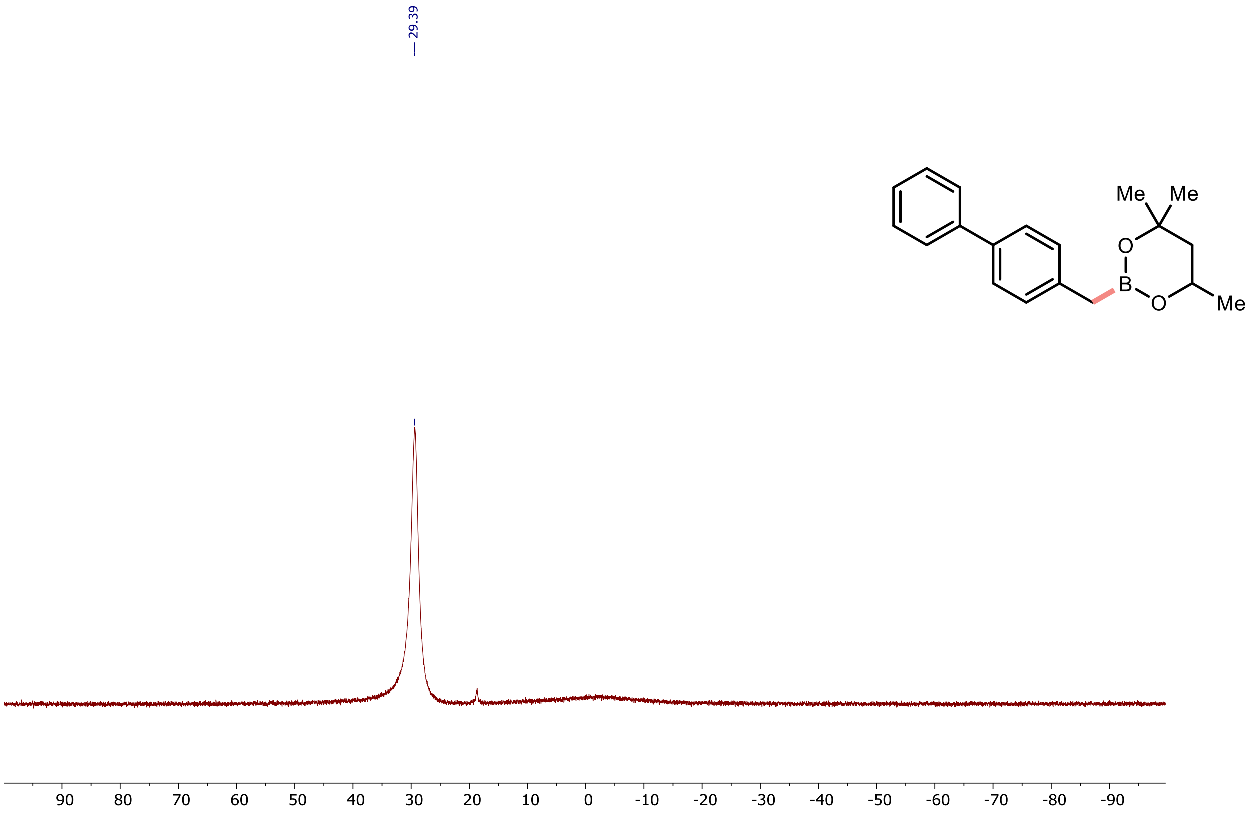

Supplementary Figure 1i-1 ^{1}H NMR (500 MHz, $CDCl_3$) 2-([1,1'-Biphenyl]-4-ylmethyl)-4,4,6,6-tetramethyl-1,3,2-dioxaborinane

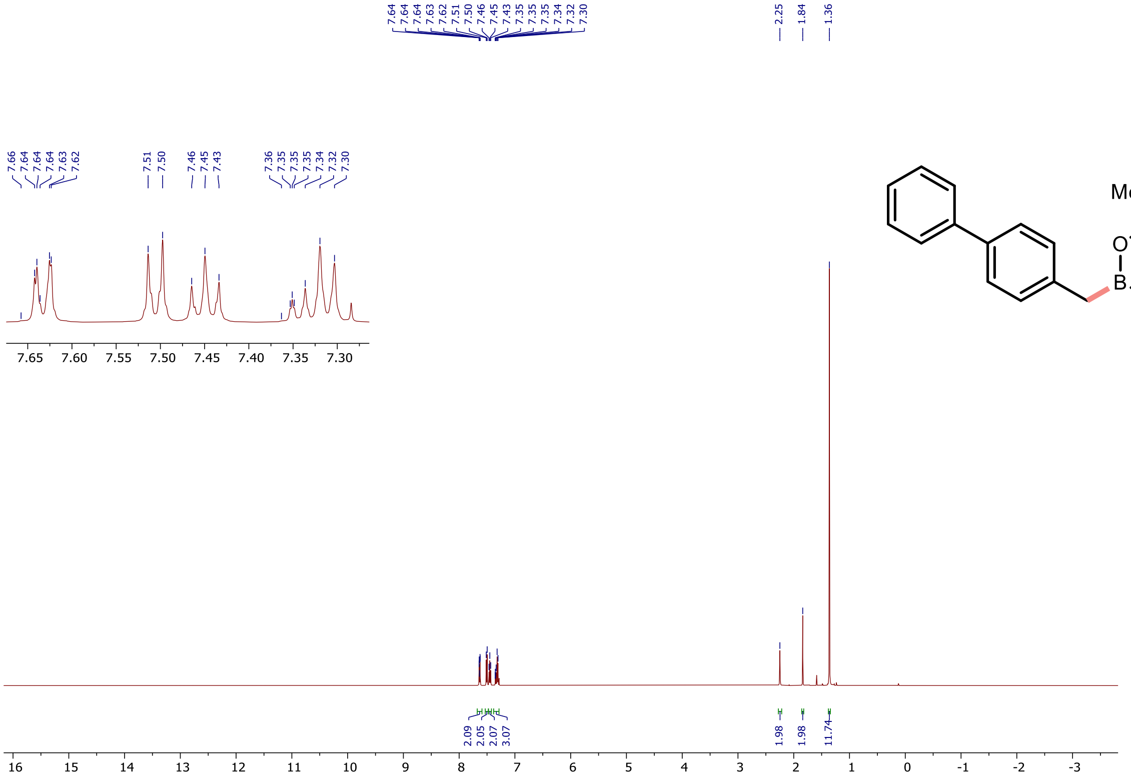

Supplementary Figure 1i-2 ^{13}C NMR (125 MHz, $CDCl_3$) 2-([1,1'-Biphenyl]-4-ylmethyl)-4,4,6,6-tetramethyl-1,3,2-dioxaborinane

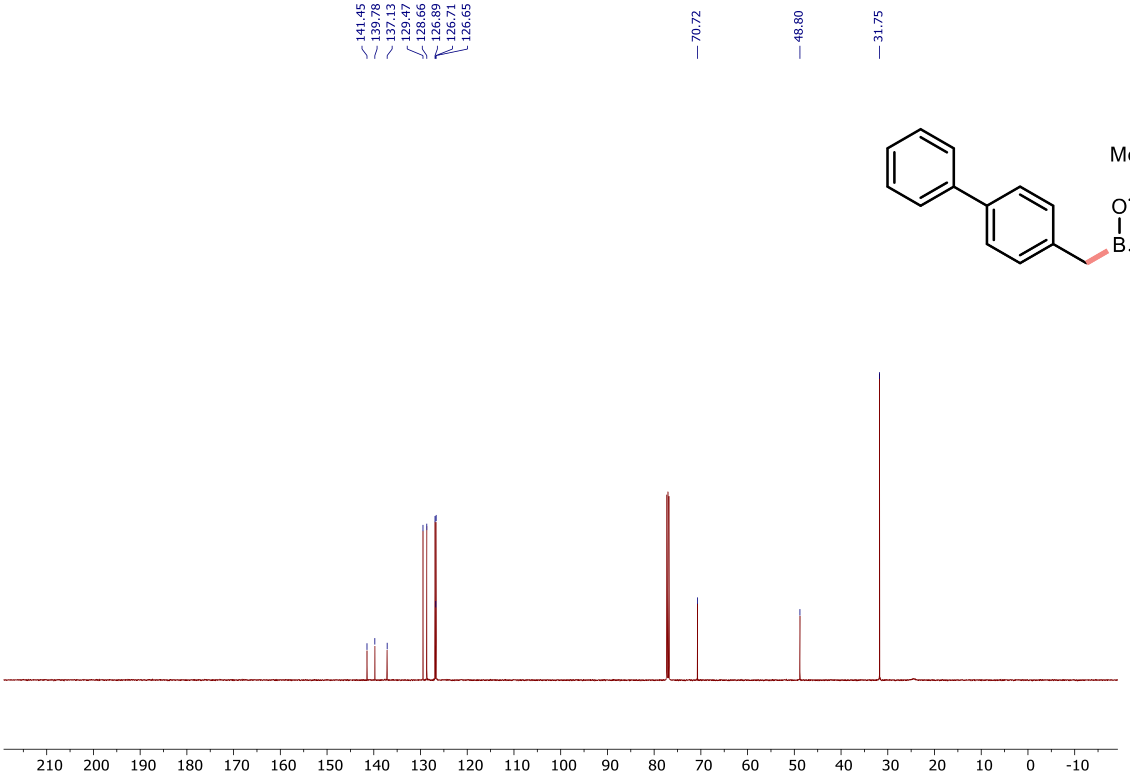

Supplementary Figure 1i-3 ^{11}B NMR (161 MHz, $CDCl_3$) 2-([1,1'-Biphenyl]-4-ylmethyl)-4,4,6,6-tetramethyl-1,3,2-dioxaborinane

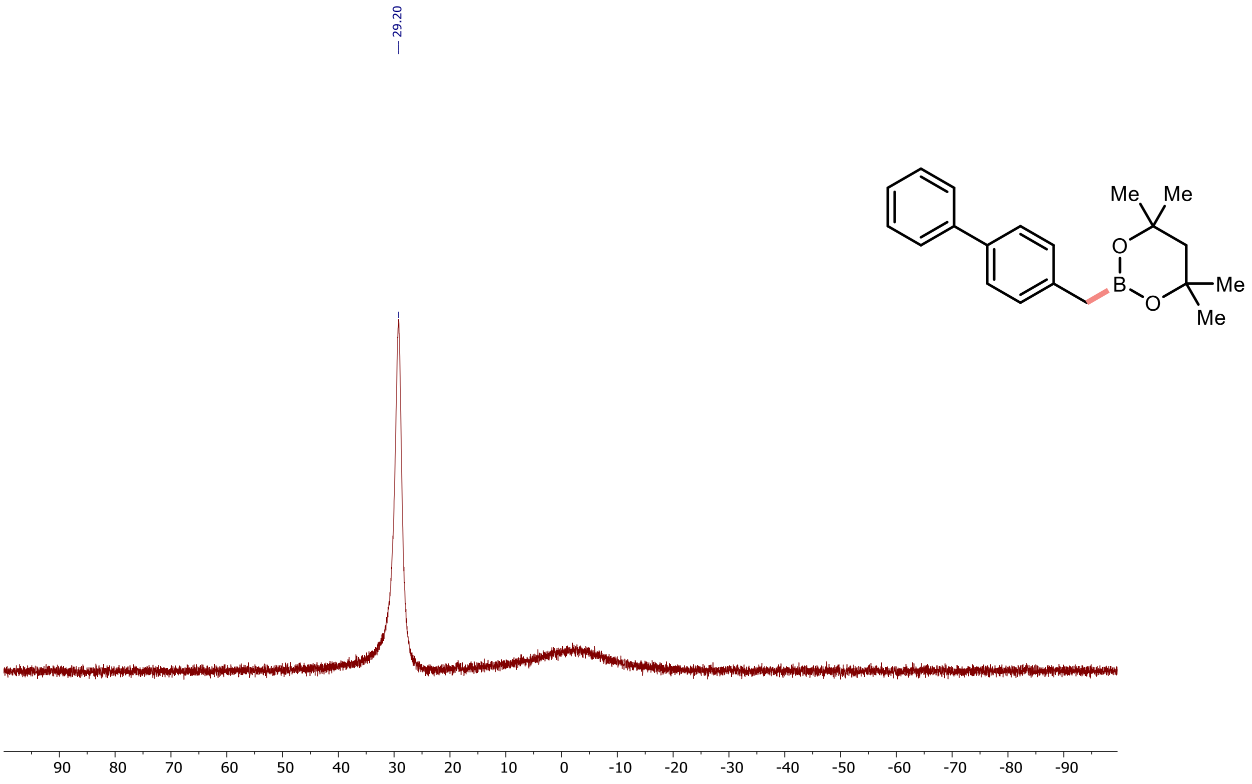

Supplementary Figure 1j-1 ^{1}H NMR (500 mHz, CD_3CN) 2-([1,1'-Biphenyl]-4-ylmethyl)-6-methyl-1,3,6,2-dioxazaborocane-4,8-dione

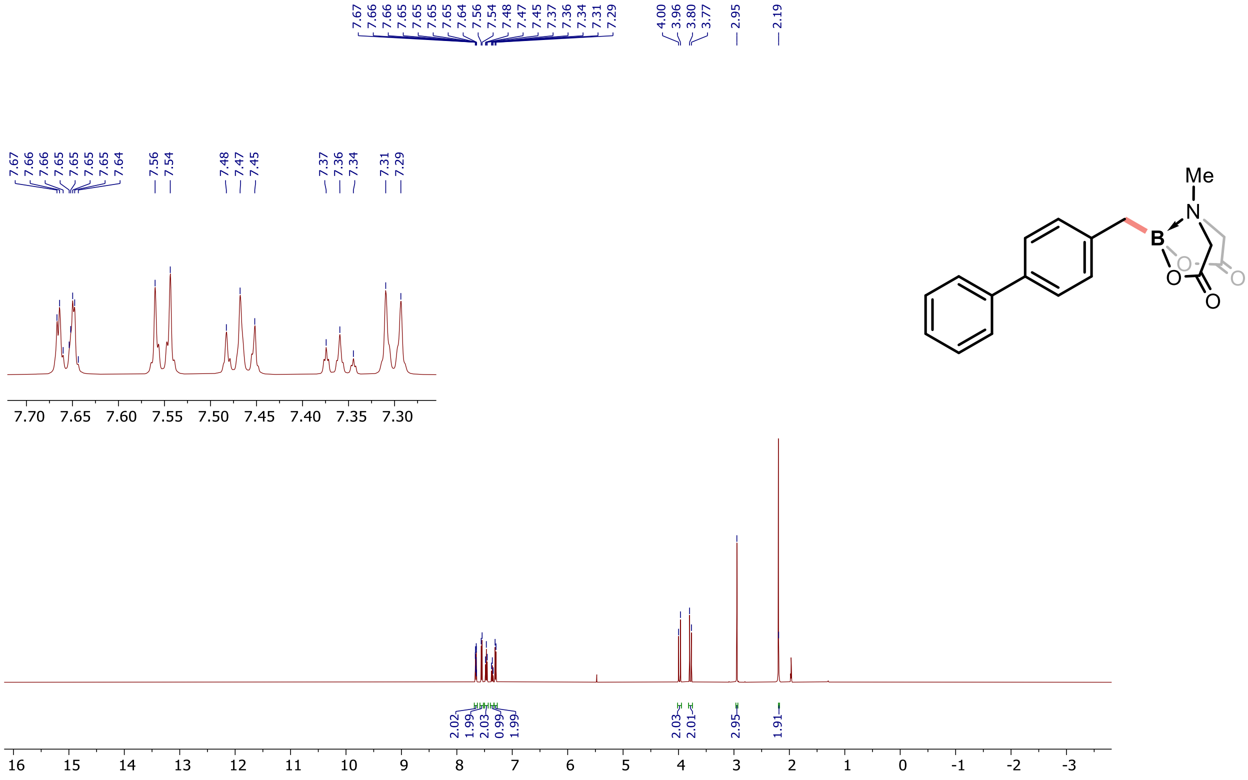

Supplementary Figure 1j-2 ^{13}C NMR (125 mHz, CD_3CN) 2-([1,1'-Biphenyl]-4-ylmethyl)-6-methyl-1,3,6,2-dioxazaborocane-4,8-dione

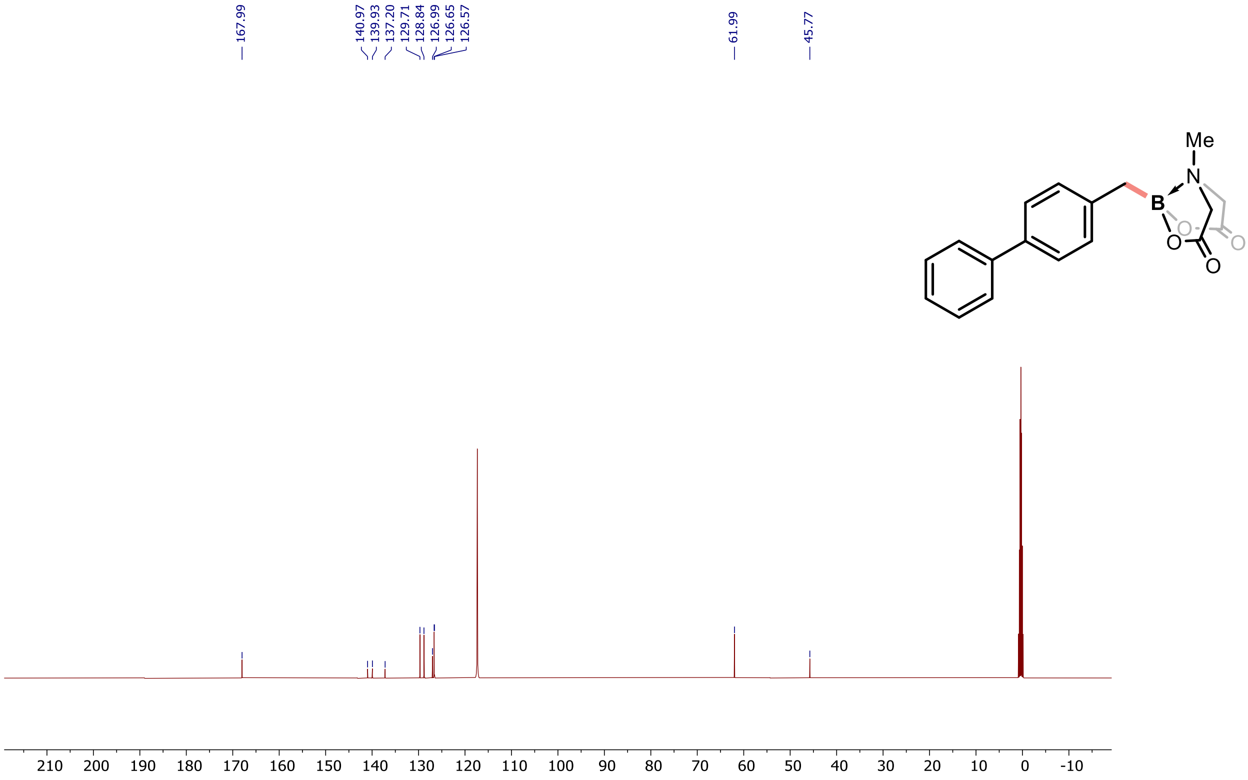

Supplementary Figure 1j-3 ^{11}B NMR (161 mHz, CD_3CN) 2-([1,1'-Biphenyl]-4-ylmethyl)-6-methyl-1,3,6,2-dioxazaborocane-4,8-dione

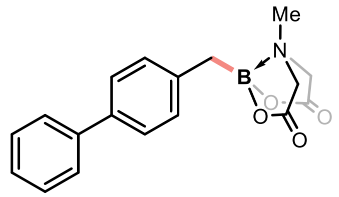

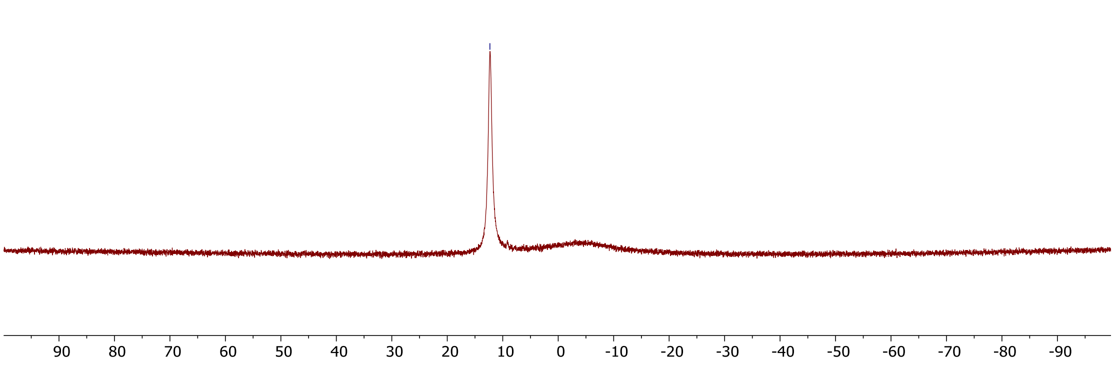

Supplementary Figure 1k-1 ^{1}H NMR (500 mHz, $CDCl_3$) 2-([1,1'-Biphenyl]-4-ylmethyl)-2,3-dihydro-1*H*-naphtho[1,8-*de*][1,3,2]diazaborinine

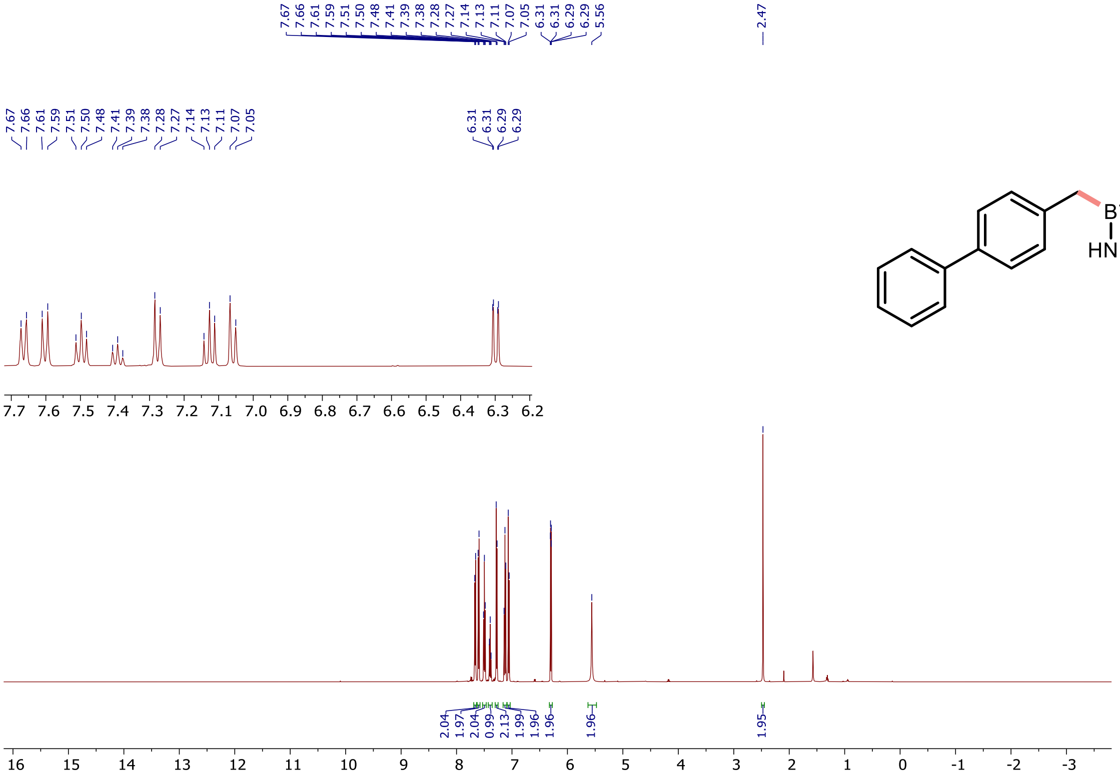

Supplementary Figure 1k-2 ^{13}C NMR (125 mHz, $CDCl_3$) 2-([1,1'-Biphenyl]-4-ylmethyl)-2,3-dihydro-1*H*-naphtho[1,8-*de*][1,3,2]diazaborinine

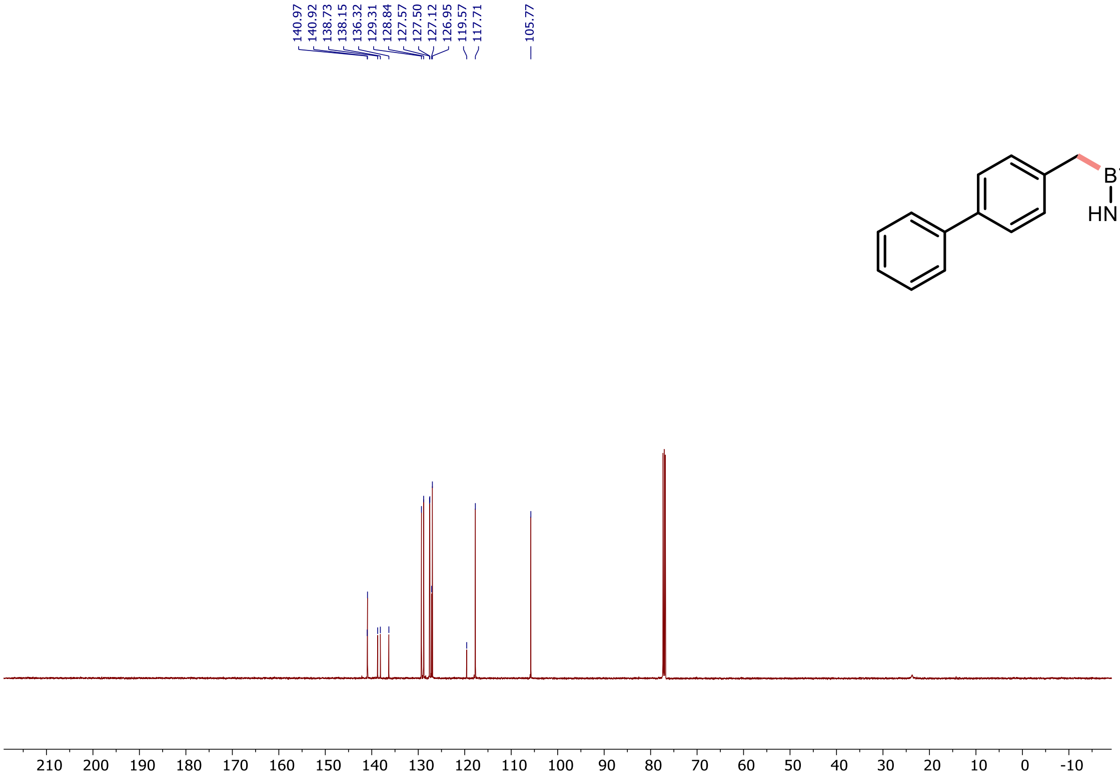

Supplementary Figure 1k-3 ^{11}B NMR (161 mHz, $CDCl_3$) 2-([1,1'-Biphenyl]-4-ylmethyl)-2,3-dihydro-1*H*-naphtho[1,8-*de*][1,3,2]diazaborinine

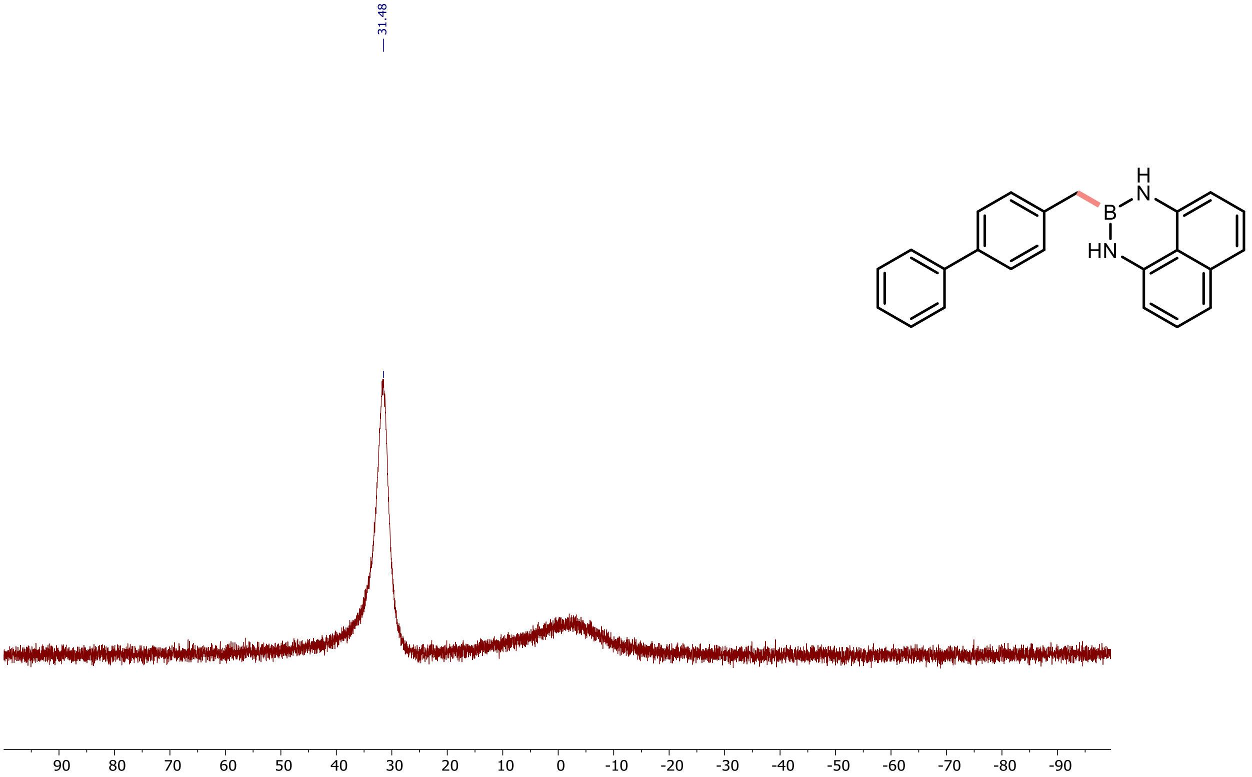

Supplementary Figure 2b-1 ^{1}H NMR (500 mHz, $CDCl_3$) 2-Benzyl-4,4,5,5-tetramethyl-1,3,2-dioxaborolane

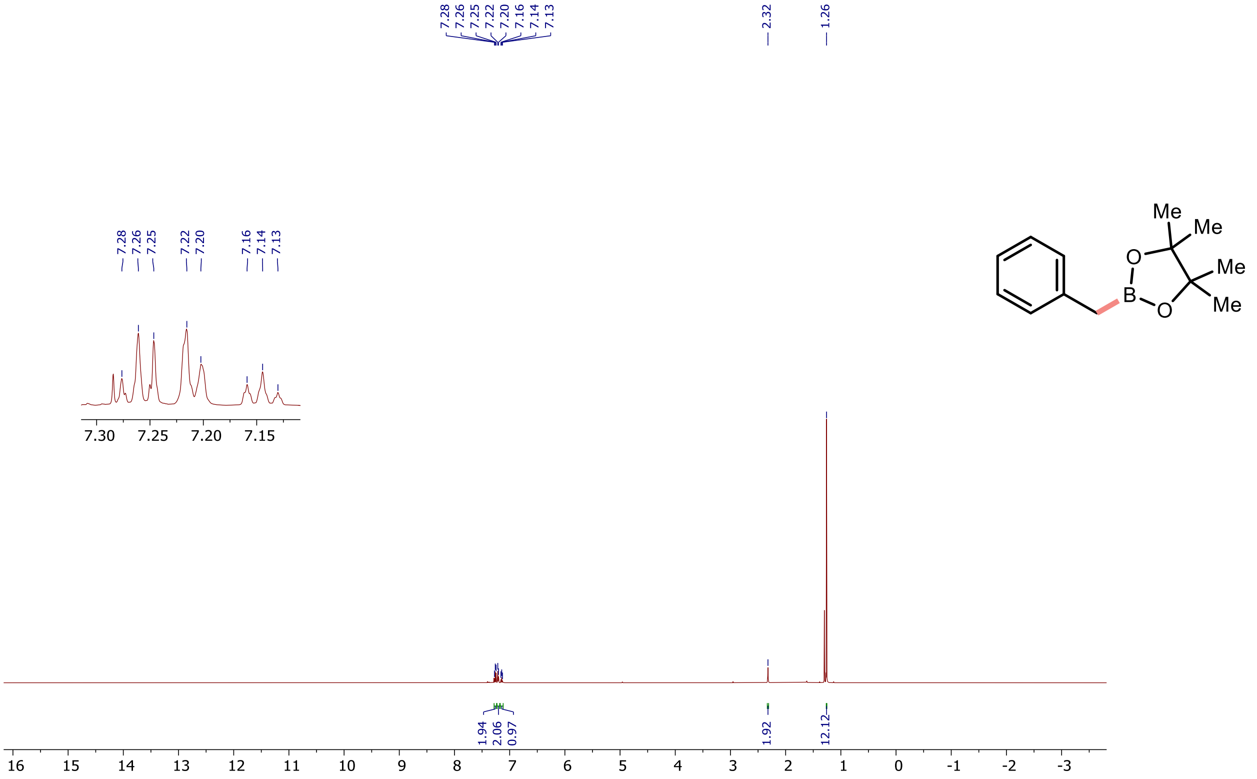

Supplementary Figure 2b-2 ^{13}C NMR (125 mHz, $CDCl_3$) 2-Benzyl-4,4,5,5-tetramethyl-1,3,2-dioxaborolane

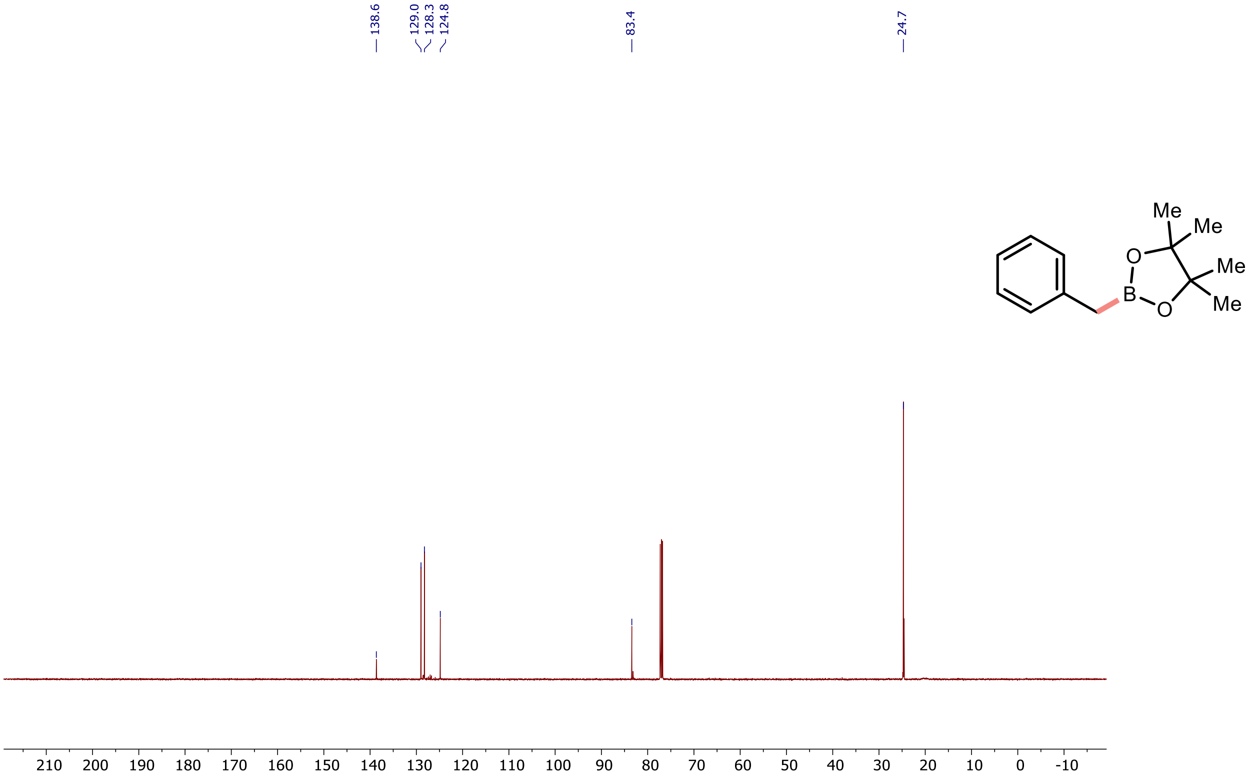

Supplementary Figure 2b-3 ^{11}B NMR (161 mHz, $CDCl_3$) 2-Benzyl-4,4,5,5-tetramethyl-1,3,2-dioxaborolane

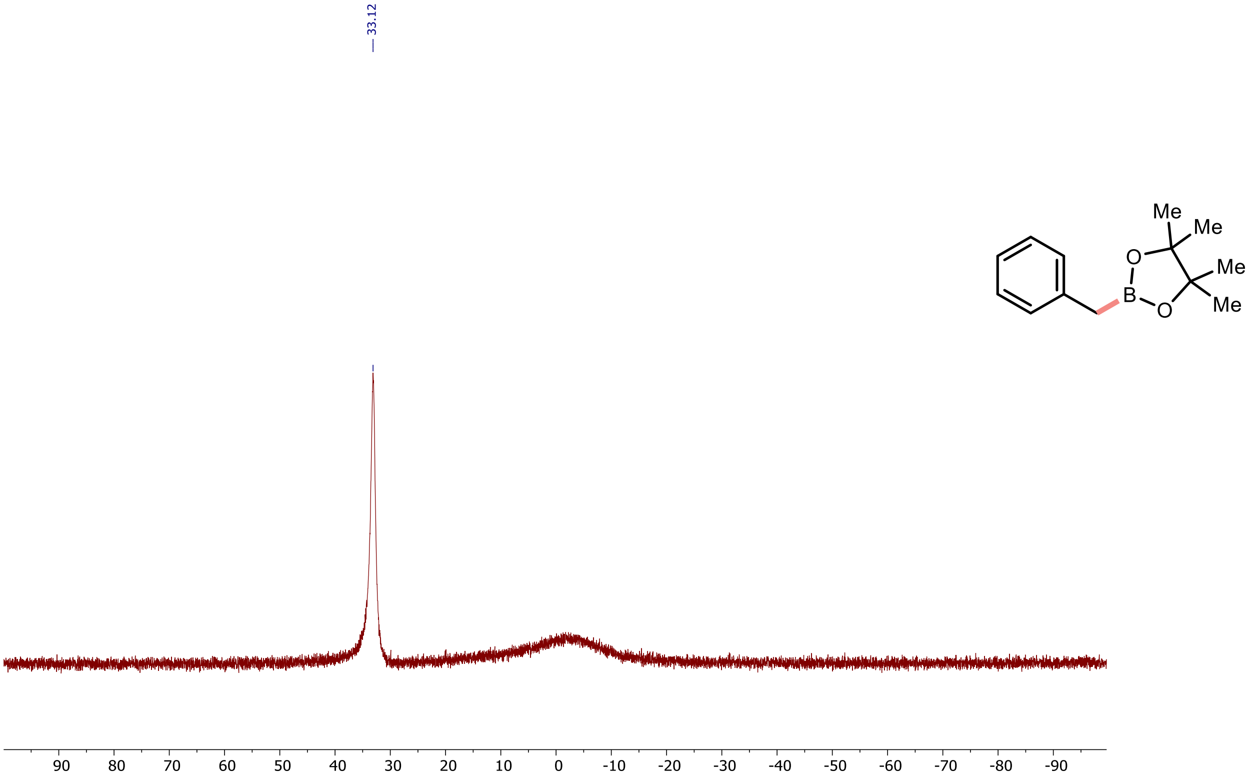

Supplementary Figure 2l-1 ^{1}H NMR (500 MHz, DMSO-d_6) Benzyltrifluoro-λ^4-borane, potassium salt

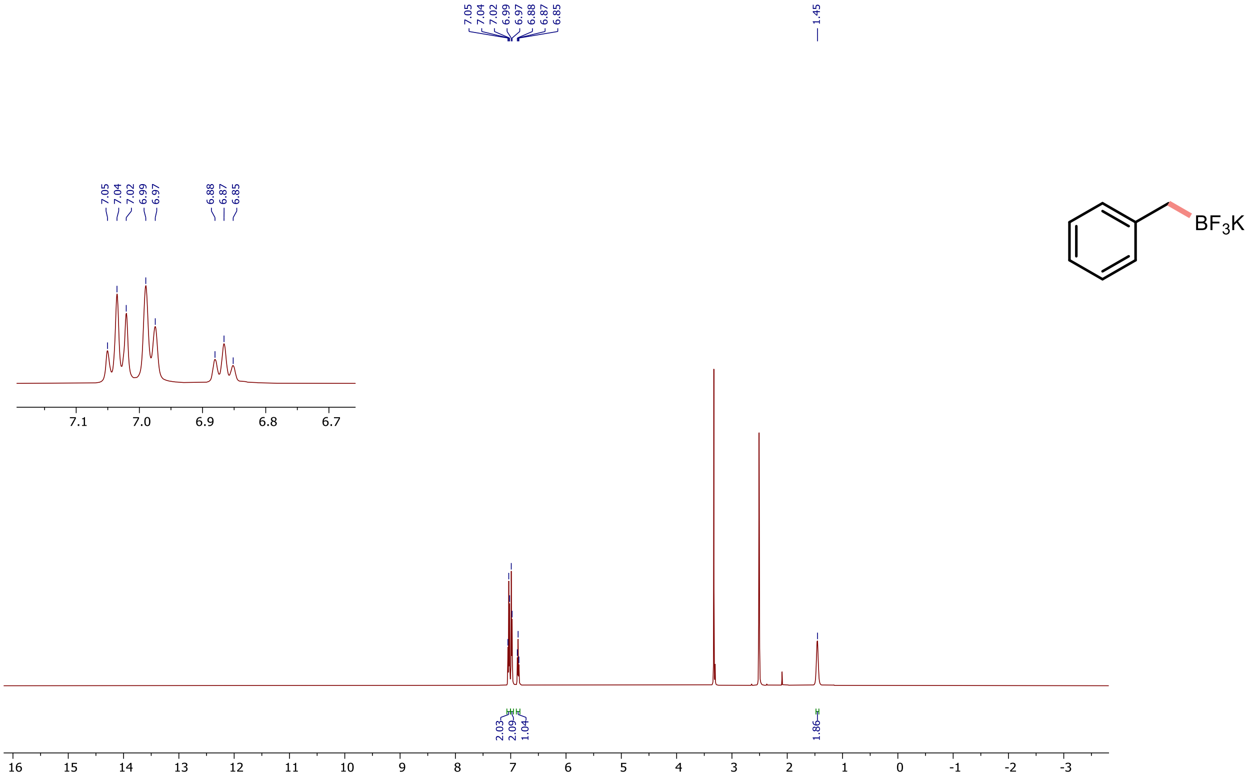

Supplementary Figure 2l-2 ^{13}C NMR (125 MHz, DMSO-d_6) Benzyltrifluoro-λ^4-borane, potassium salt

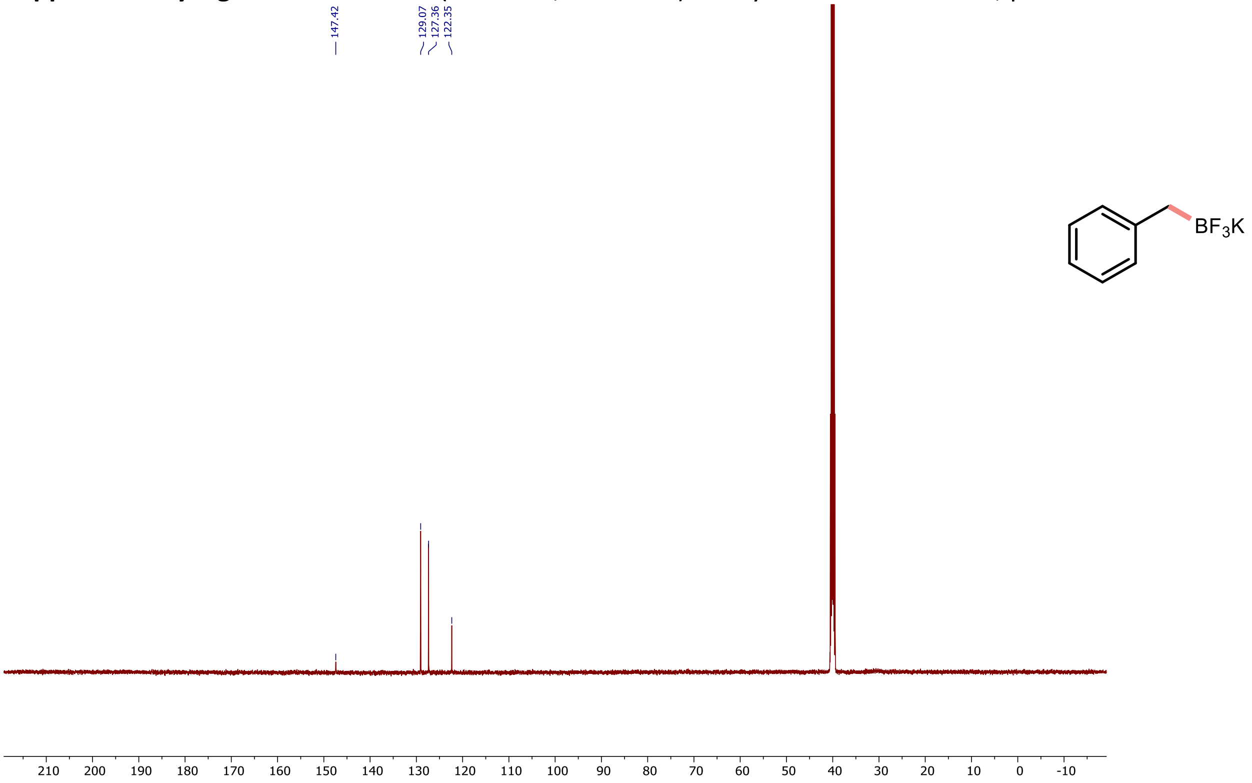

Supplementary Figure 2l-3 ^{11}B NMR (161 MHz, DMSO-d_6) Benzyltrifluoro-λ^4-borane, potassium salt

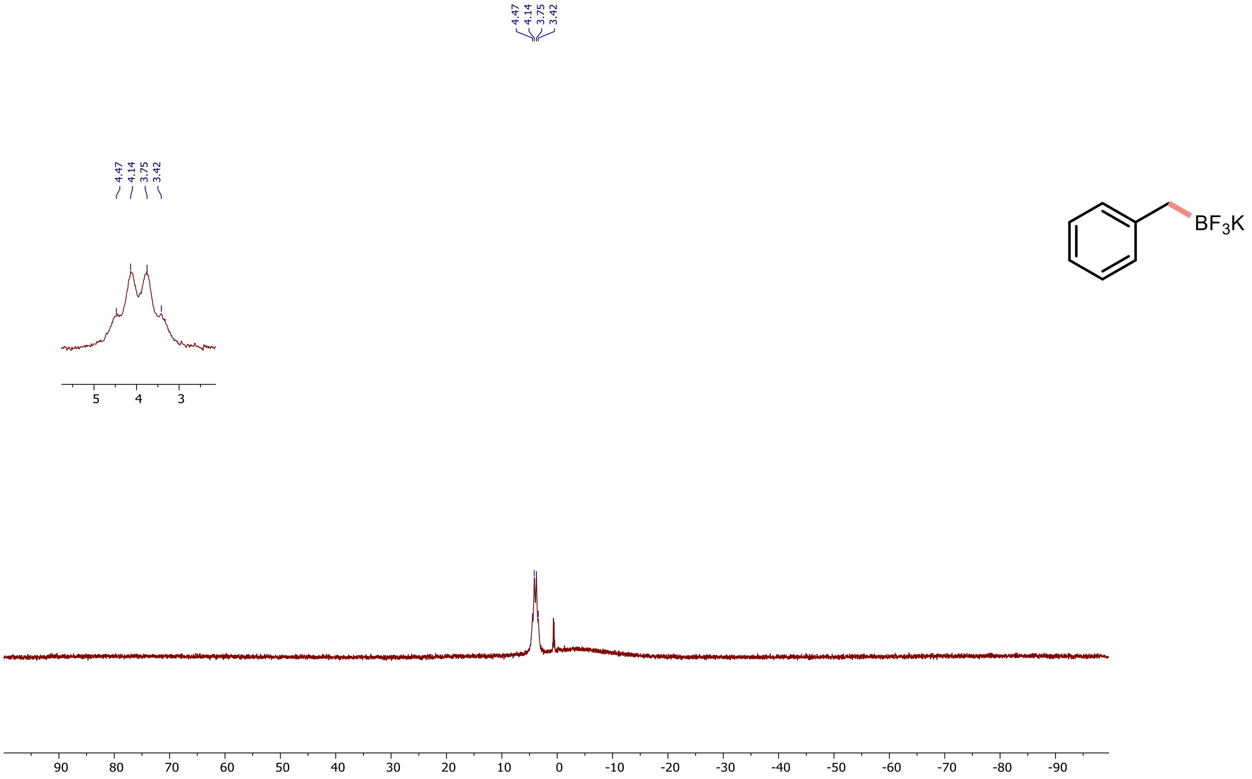

Supplementary Figure 2l-4 ^{19}F NMR (471 MHz, DMSO-d_6) Benzyltrifluoro-λ^4-borane, potassium salt

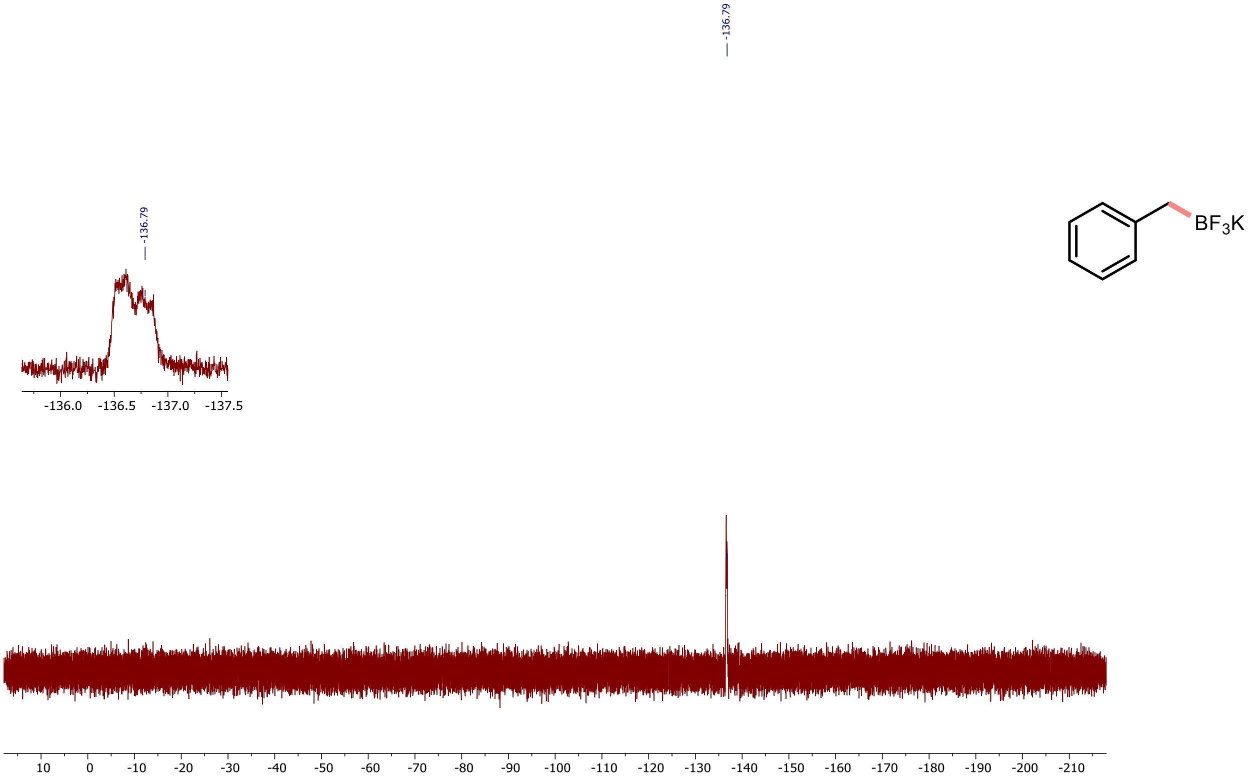

Supplementary Figure 2b-*d*-1 ^{1}H NMR (800 MHz, acetone-d_6) 4,4,5,5-Tetramethyl-2-((phenyl-d_5)methyl)-1,3,2-dioxaborolane

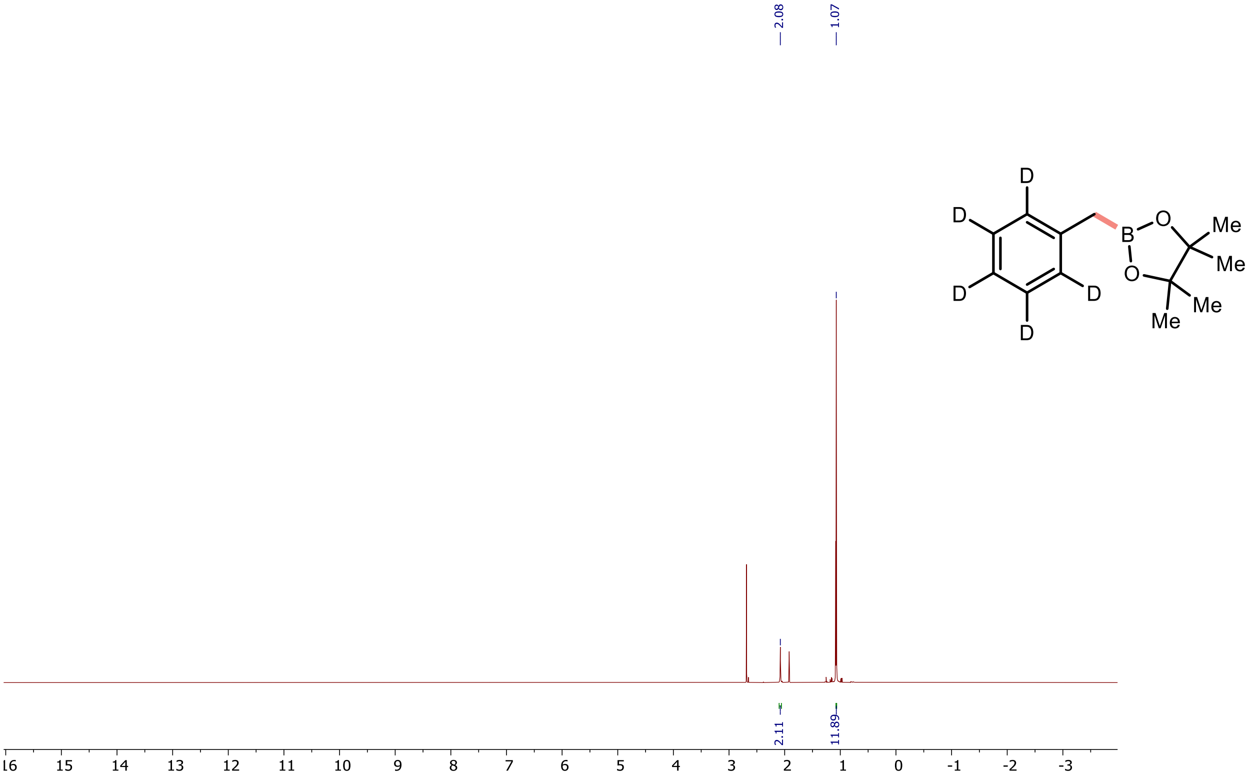

Supplementary Figure 2b-*d*-2 ^{13}C NMR (201 MHz, acetone-d_6) 4,4,5,5-Tetramethyl-2-((phenyl-d_5)methyl)-1,3,2-dioxaborolane

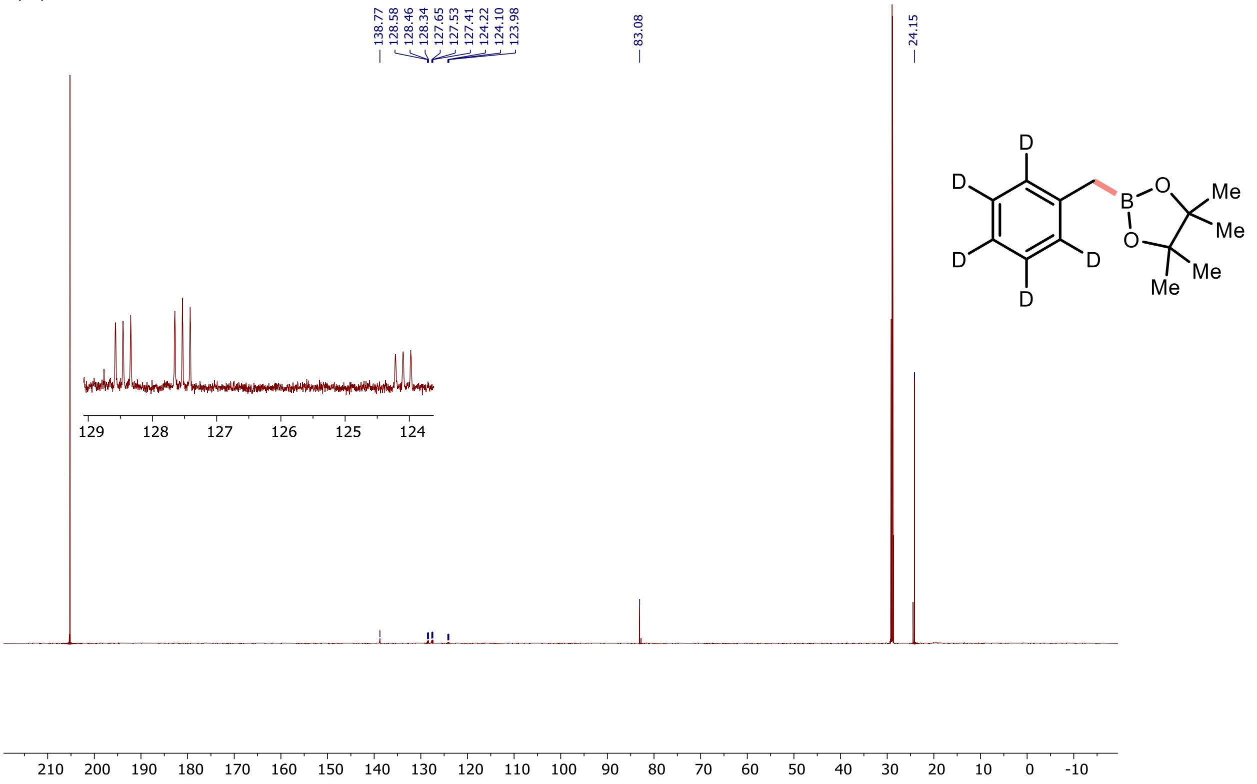

Supplementary Figure 2b-*d*-3 ^{2}H NMR (123 MHz, acetone-d_6) 4,4,5,5-Tetramethyl-2-((phenyl-d_5)methyl)-1,3,2-dioxaborolane

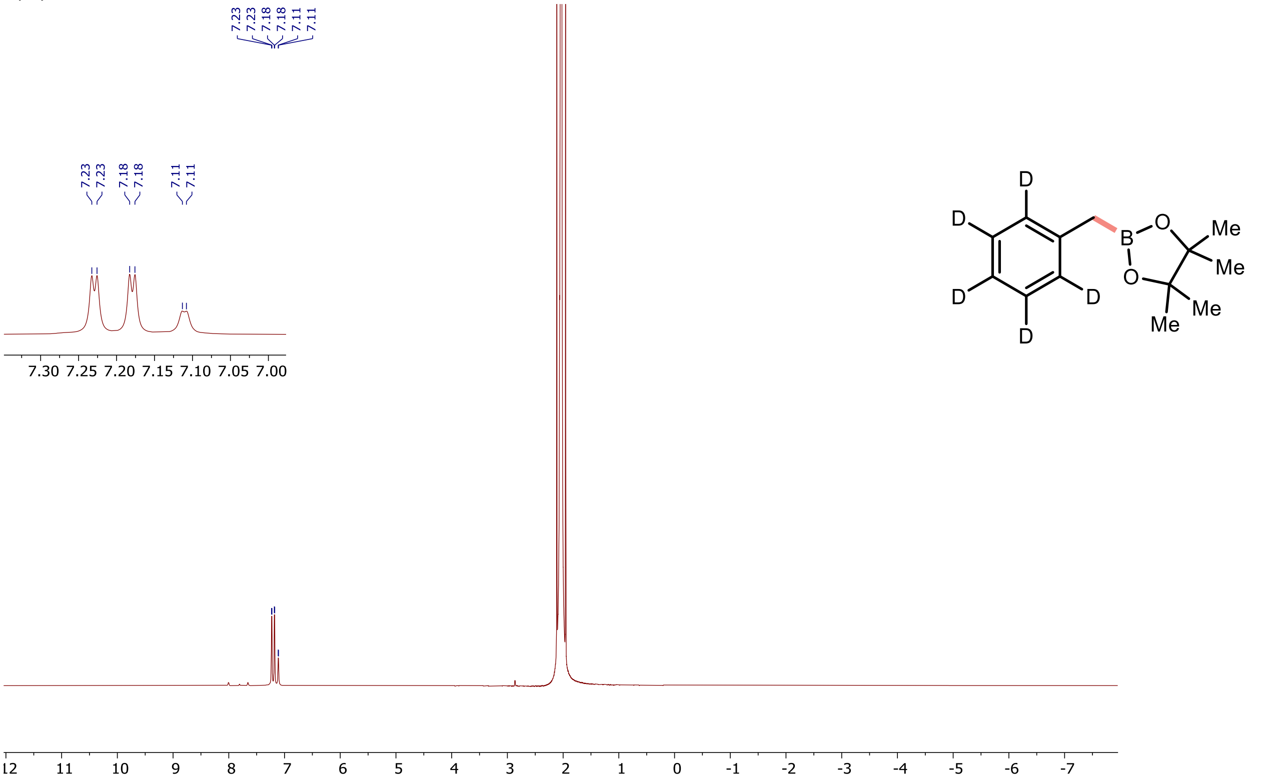

Supplementary Figure 2b-*d*-4 ^{11}B NMR (161 MHz, acetone-d_6) 4,4,5,5-Tetramethyl-2-((phenyl-d_5)methyl)-1,3,2-dioxaborolane

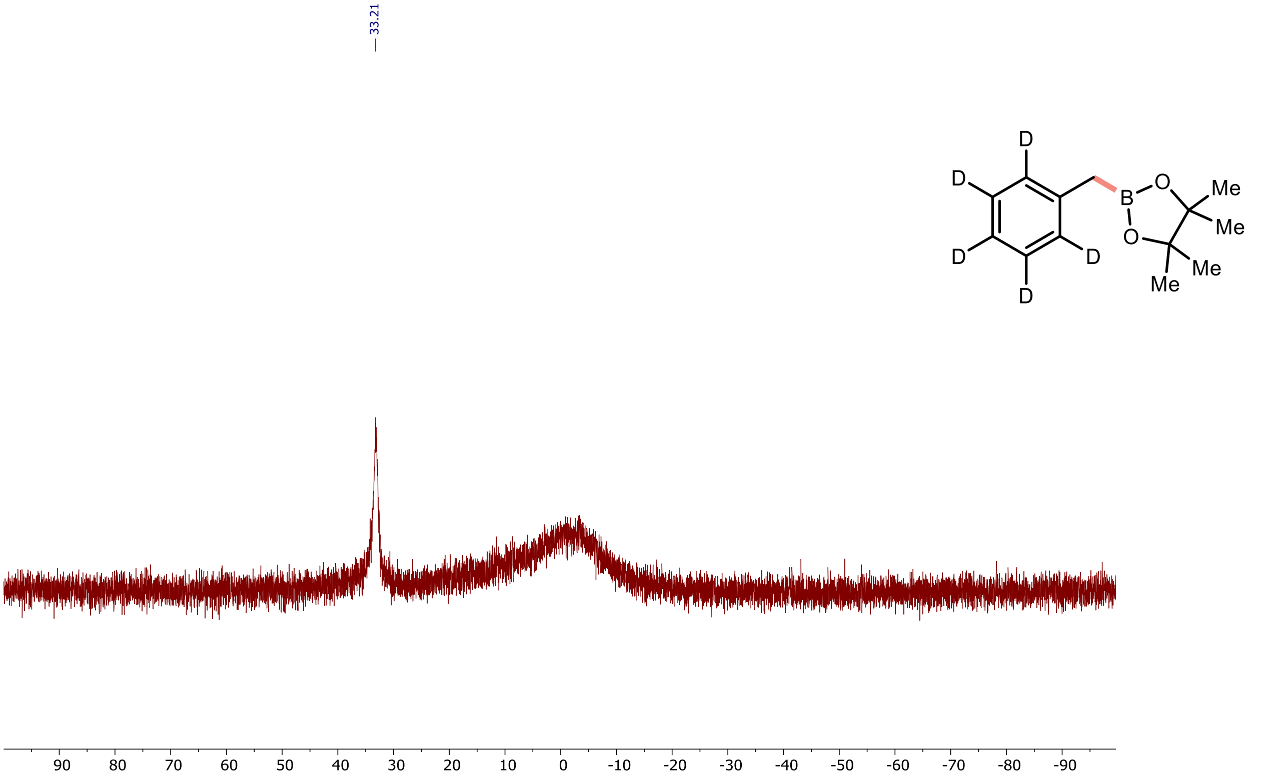

Supplementary Figure 2g-^{13}C-1 ^{1}H NMR (500 MHz, $CDCl_3$) (3a*S*,4*S*,6*S*,7a*R*)-3a,5,5-trimethyl-2-(phenyl-λ^{2}-methyl-^{13}*C*)hexahydro-4,6-methanobenzo[*d*][1,3,2]dioxaborole

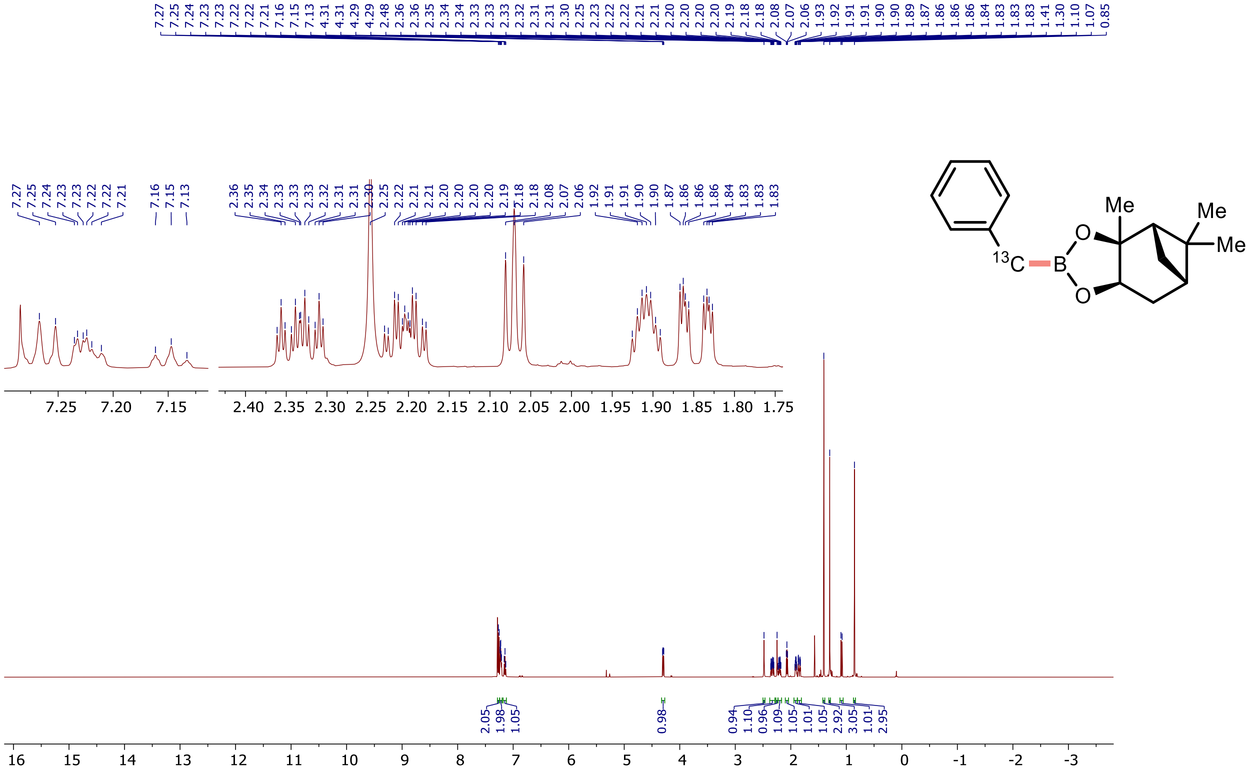

Supplementary Figure 2g-^{13}C-2 ^{13}C NMR (125 MHz, $CDCl_3$) (3a*S*,4*S*,6*S*,7a*R*)-3a,5,5-trimethyl-2-(phenyl-λ^{2}-methyl-^{13}*C*)hexahydro-4,6-methanobenzo[*d*][1,3,2]dioxaborole

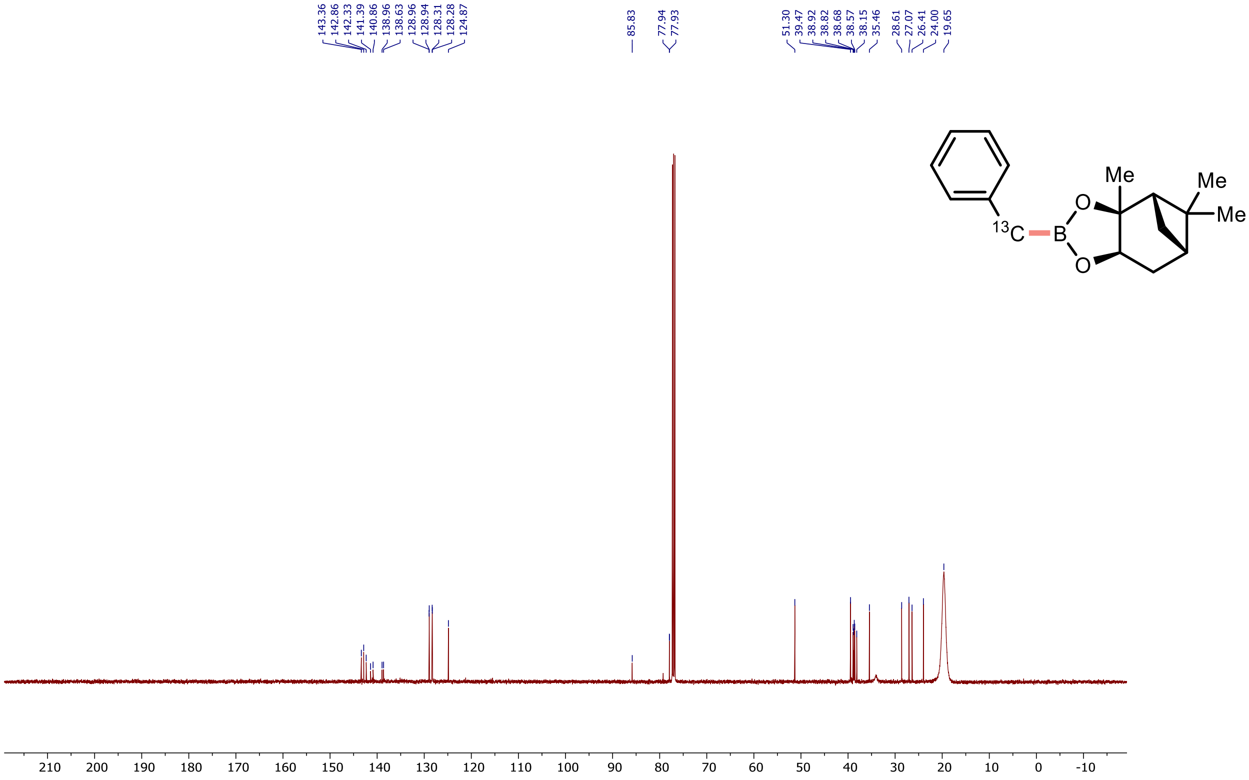

Supplementary Figure 2g-^{13}C-3 ^{11}B NMR (161 MHz, $CDCl_3$) (3a*S*,4*S*,6*S*,7a*R*)-3a,5,5-trimethyl-2-(phenyl-λ^2-methyl-^{13}*C*)hexahydro-4,6-methanobenzo[*d*][1,3,2]dioxaborole

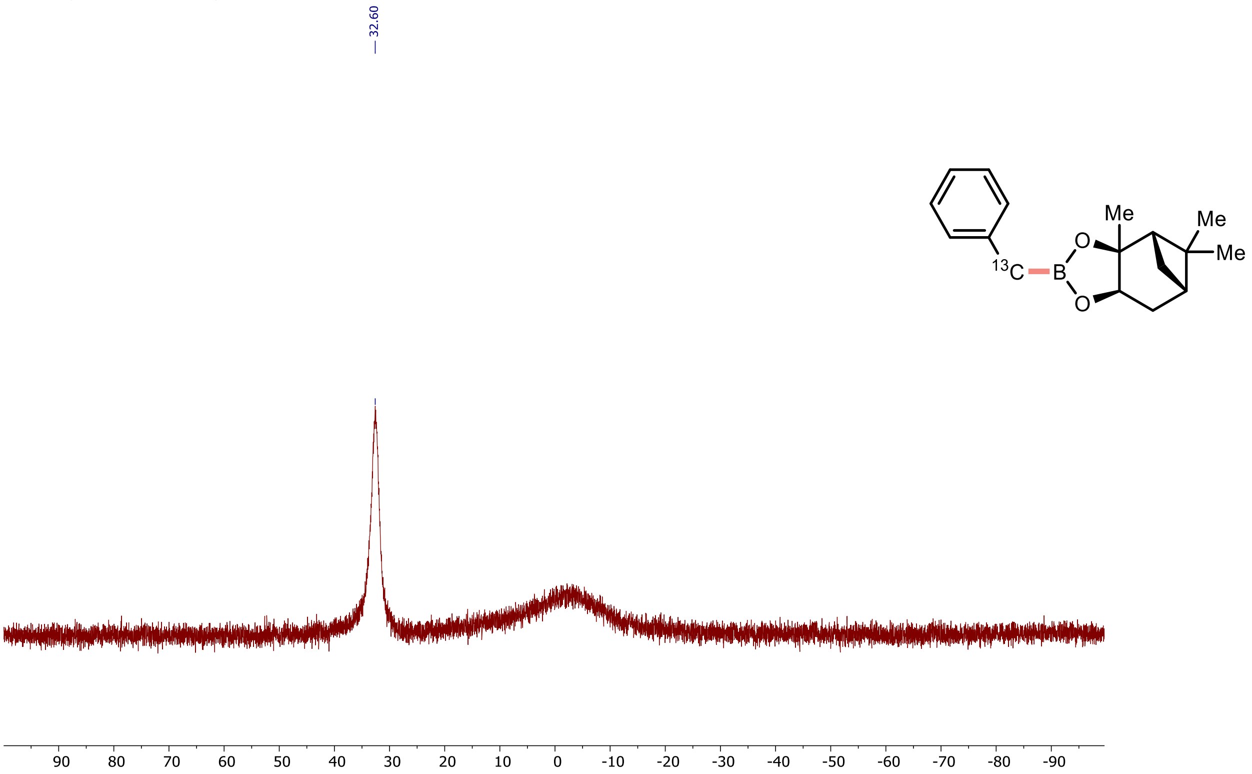

Supplementary Figure 3b-1 ^{1}H NMR (500 mHz, $CDCl_3$) 4,4,5,5-Tetramethyl-2-(4-methylbenzyl)-1,3,2-dioxaborolane

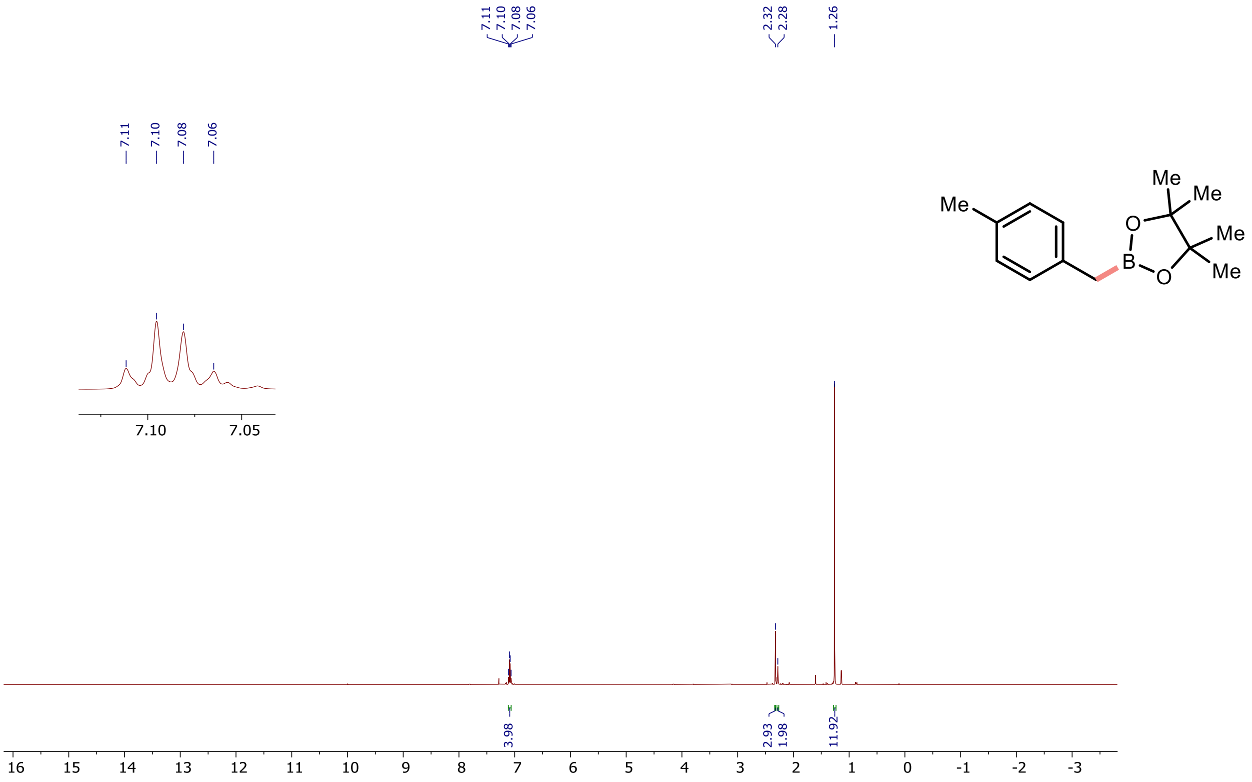

Supplementary Figure 3b-2 ^{13}C NMR (125 mHz, $CDCl_3$) 4,4,5,5-Tetramethyl-2-(4-methylbenzyl)-1,3,2-dioxaborolane

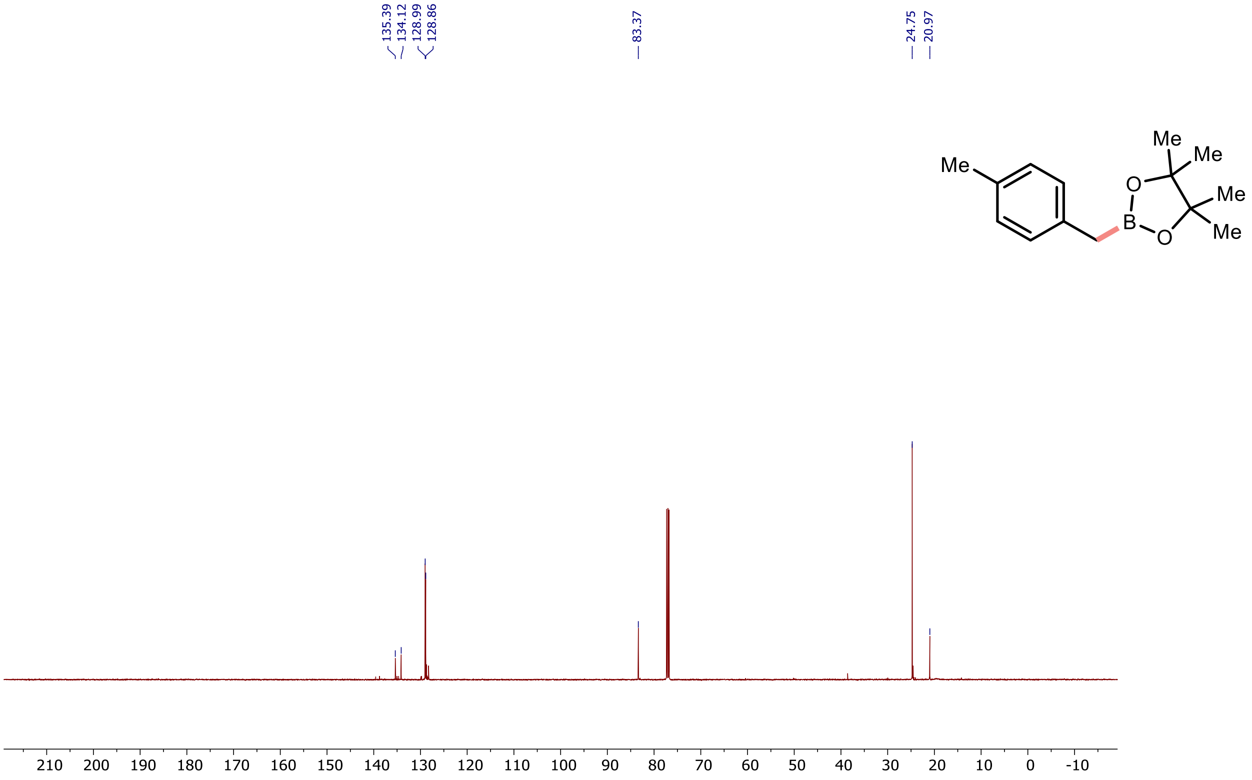

Supplementary Figure 3b-3 ^{11}B NMR (161 mHz, $CDCl_3$) 4,4,5,5-Tetramethyl-2-(4-methylbenzyl)-1,3,2-dioxaborolane

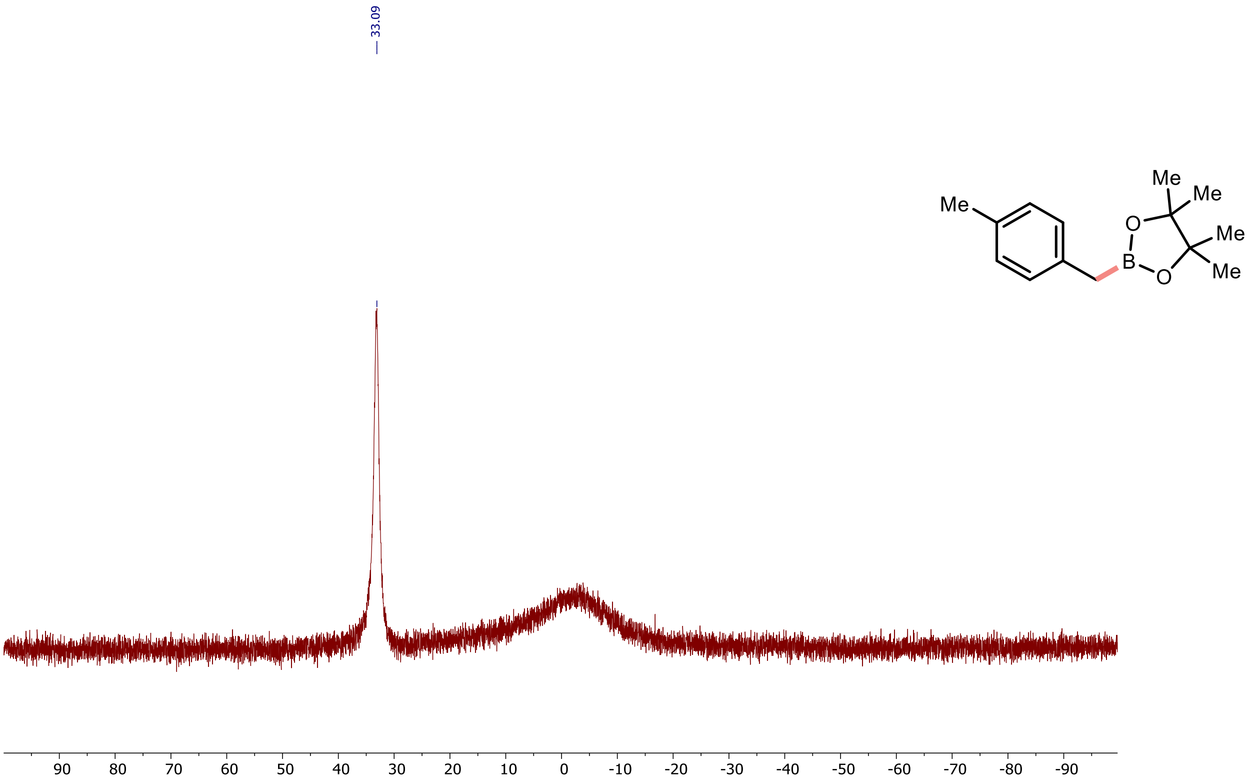

Supplementary Figure 3l-1 ^{1}H NMR (500 mHz, acetone-d_6) Trifluoro(4-methylbenzyl)- λ^{4}-borane, potassium salt

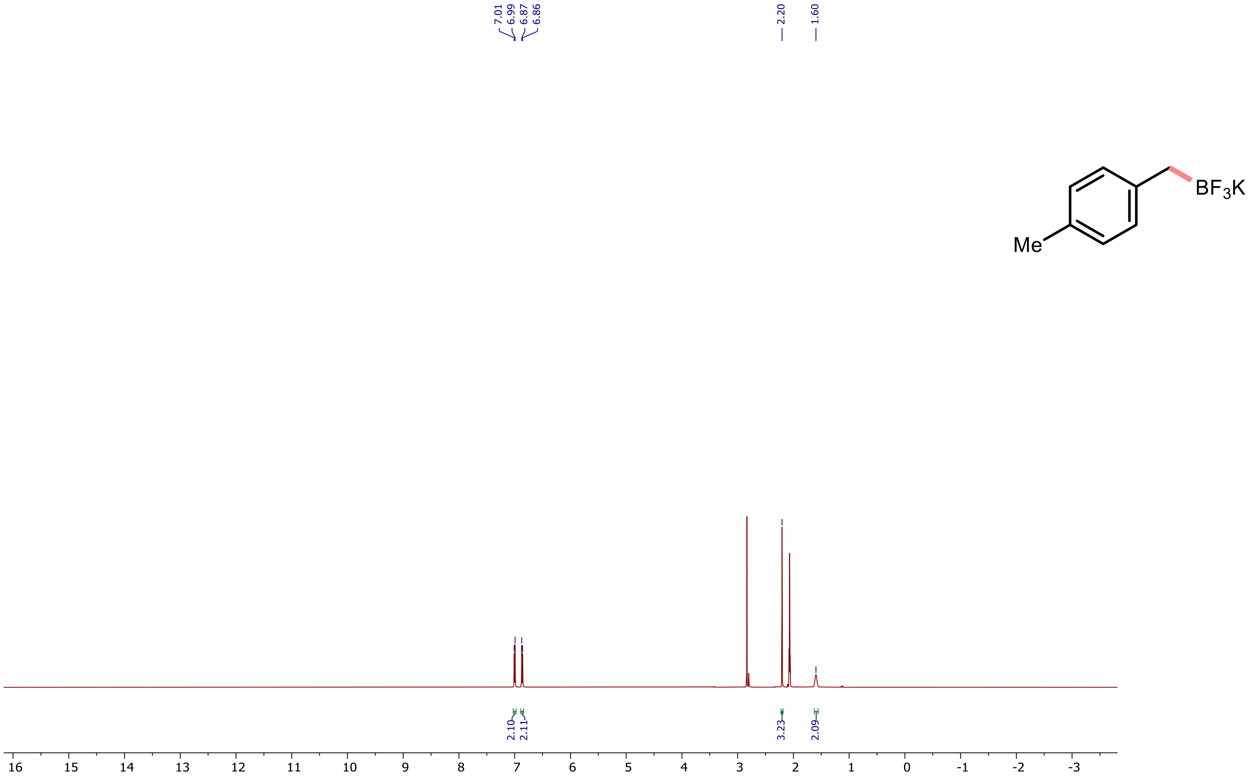

Supplementary Figure 3l-2 ^{13}C NMR (125 mHz, acetone-d_6) Trifluoro(4-methylbenzyl)- λ^{4}-borane, potassium salt

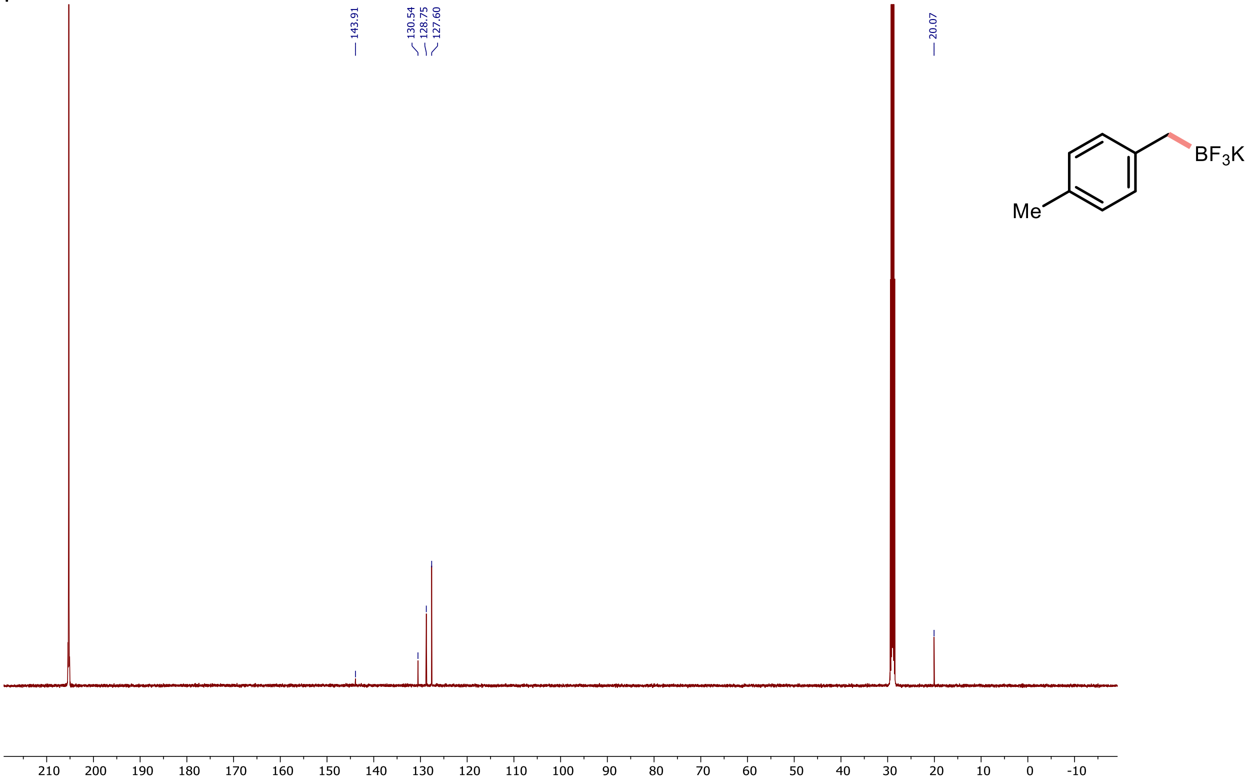

Supplementary Figure 3l-3 ^{11}B NMR (161 mHz, acetone-d_6) Trifluoro(4-methylbenzyl)- λ^4-borane, potassium salt

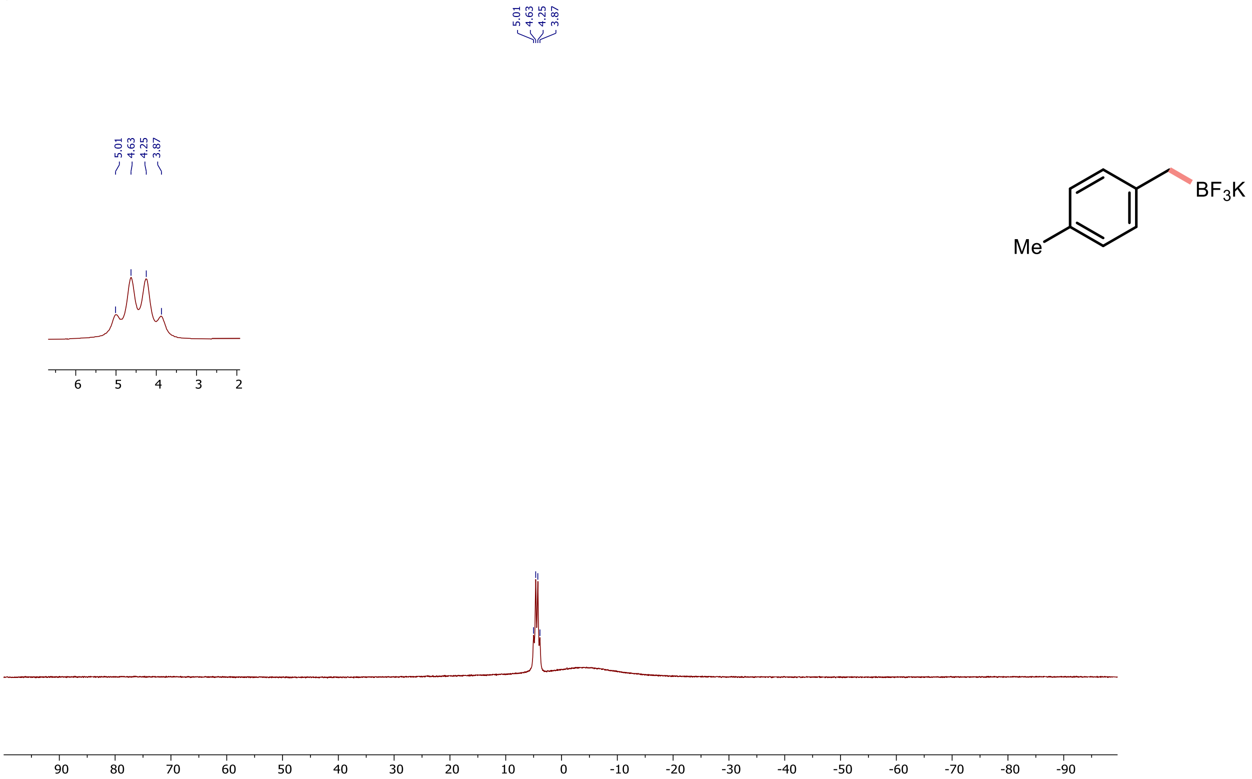

Supplementary Figure 3l-4 ^{19}F NMR (471 mHz, acetone-d_6) Trifluoro(4-methylbenzyl)- λ^4-borane, potassium salt

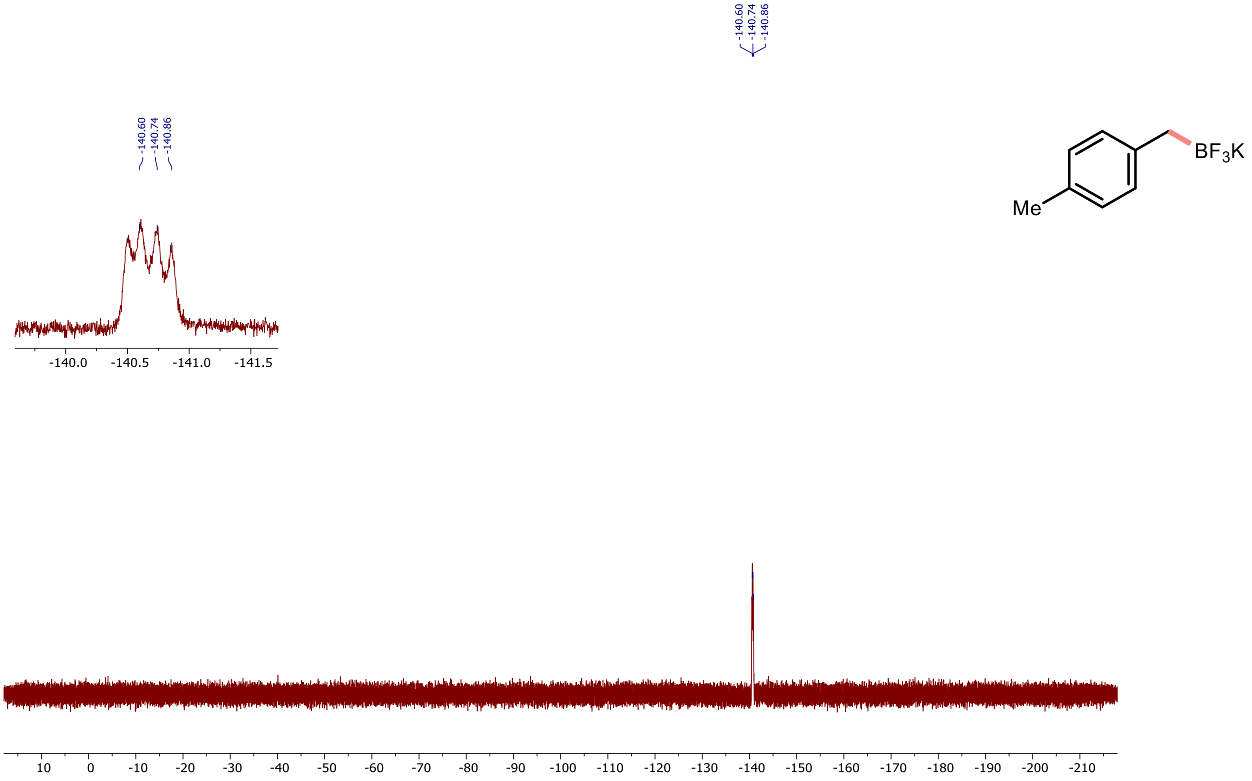

Supplementary Figure 4b-1 ^{1}H NMR (500 mHz, $CDCl_3$) 2-(4-Ethylbenzyl)-4,4,5,5-tetramethyl-1,3,2-dioxaborolane

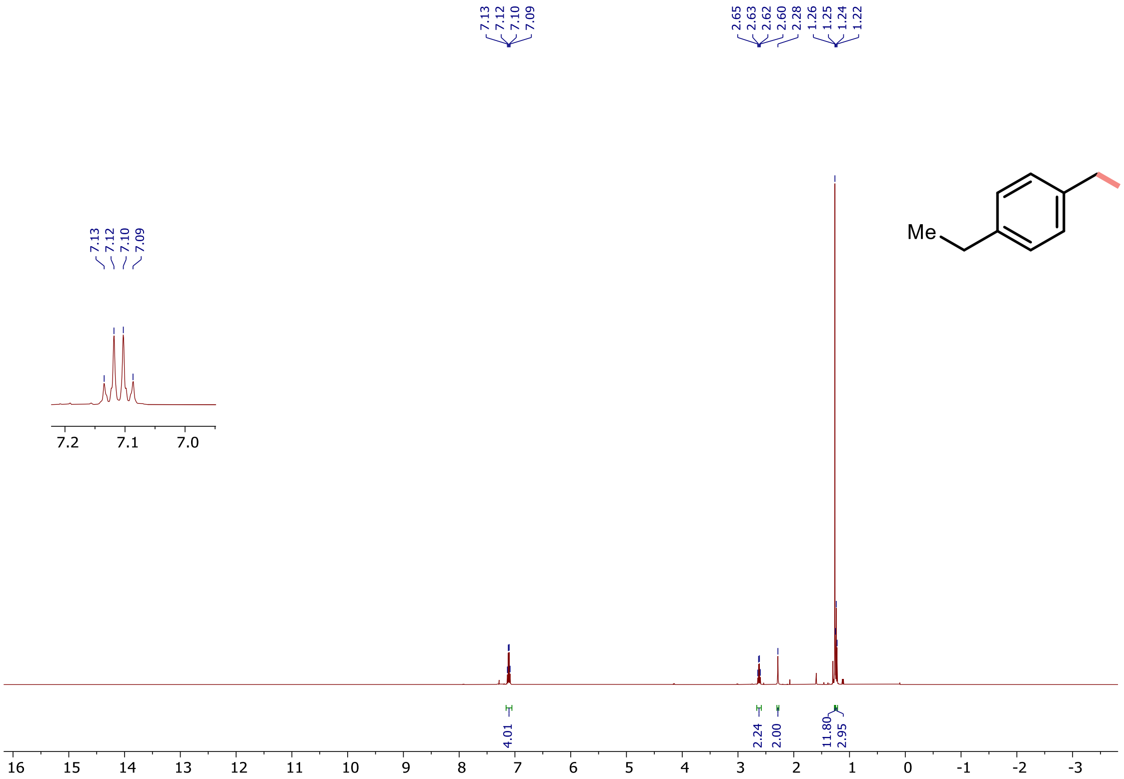

Supplementary Figure 4b-2 ^{13}C NMR (125 mHz, $CDCl_3$) 2-(4-Ethylbenzyl)-4,4,5,5-tetramethyl-1,3,2-dioxaborolane

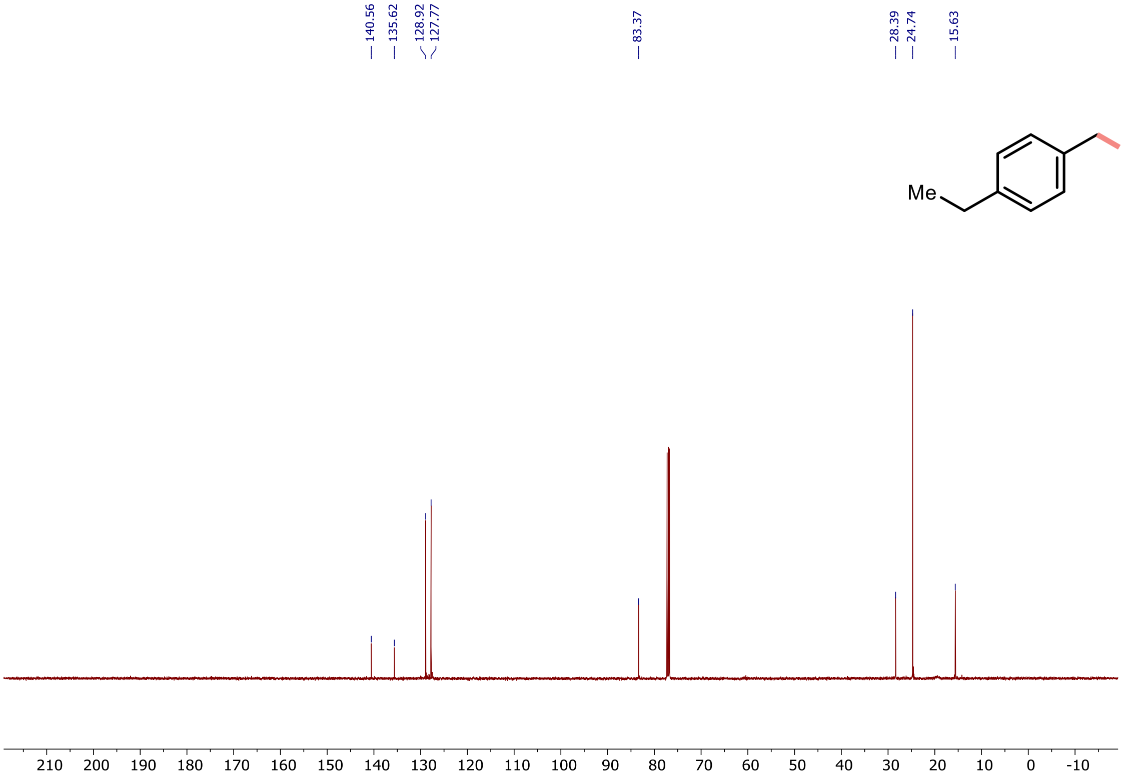

Supplementary Figure 4b-3 ^{11}B NMR (161 mHz, $CDCl_3$) 2-(4-Ethylbenzyl)-4,4,5,5-tetramethyl-1,3,2-dioxaborolane

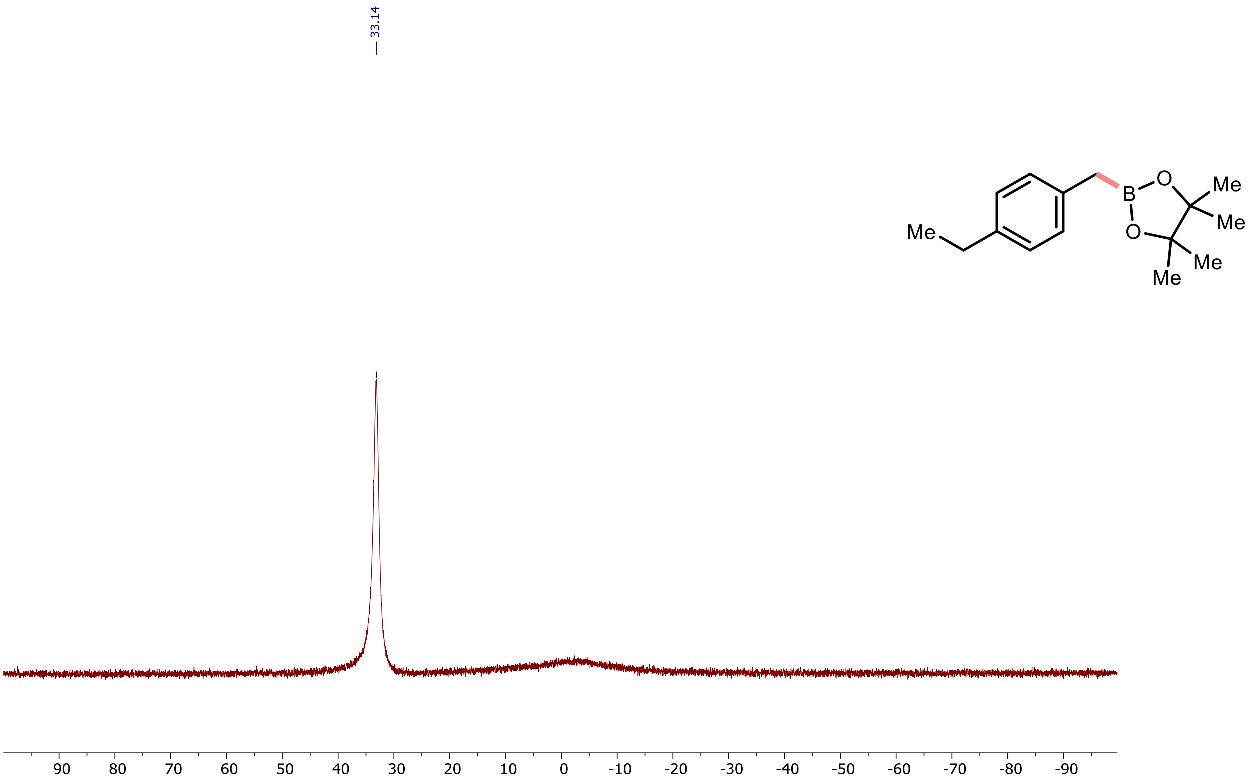

Supplementary Figure 5b-1 ^{1}H NMR (500 MHz, $CDCl_3$) 2-(4-Butylbenzyl)-4,4,5,5-tetramethyl-1,3,2-dioxaborolane

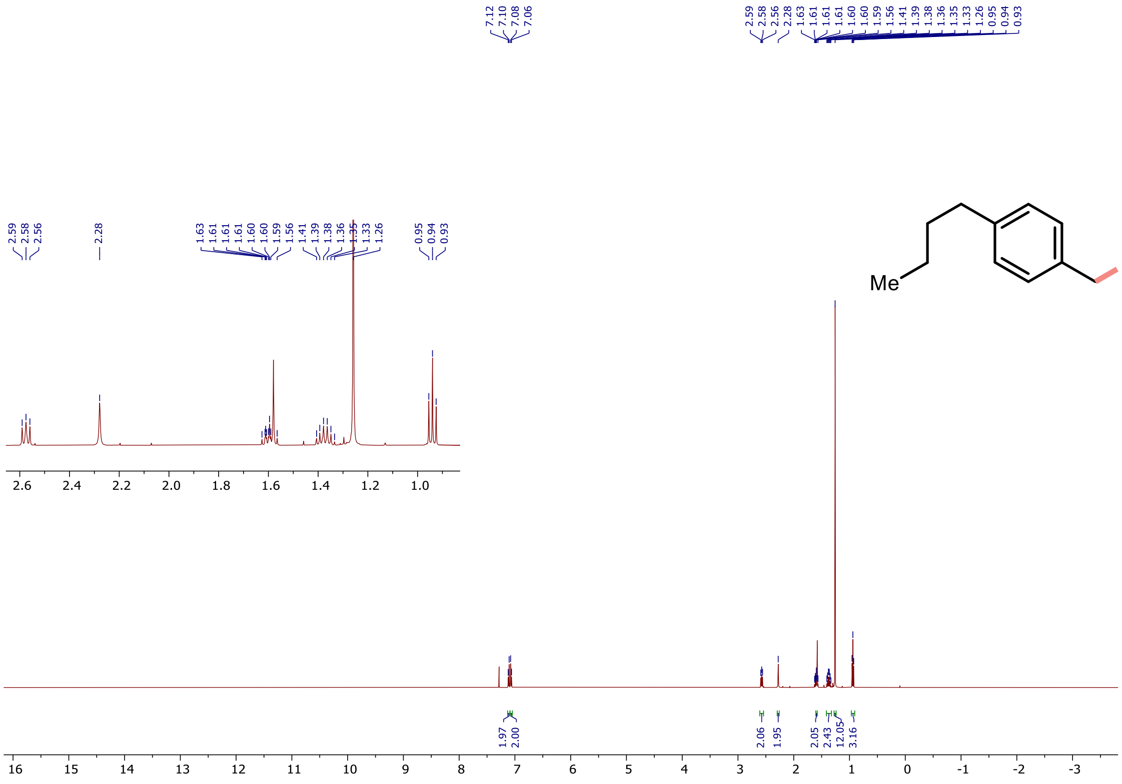

Supplementary Figure 5b-2 ^{13}C NMR (125 MHz, $CDCl_3$) 2-(4-Butylbenzyl)-4,4,5,5-tetramethyl-1,3,2-dioxaborolane

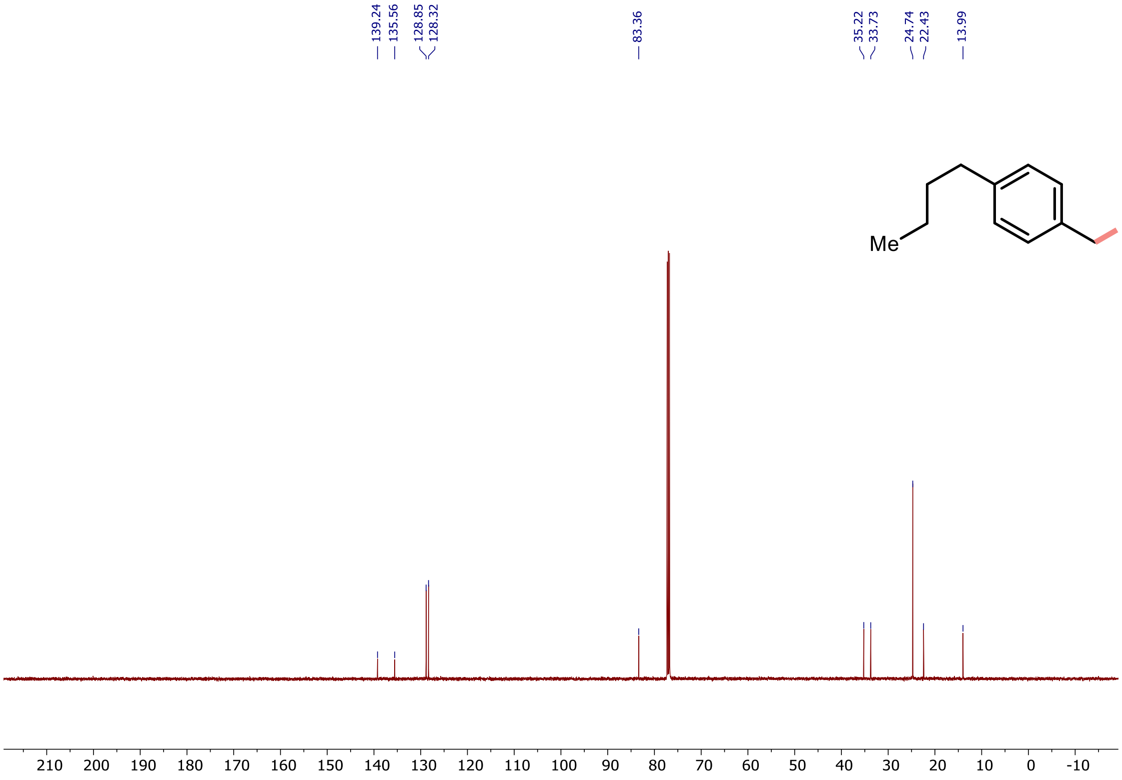

Supplementary Figure 5b-3 ^{11}B NMR (161 MHz, $CDCl_3$) 2-(4-Butylbenzyl)-4,4,5,5-tetramethyl-1,3,2-dioxaborolane

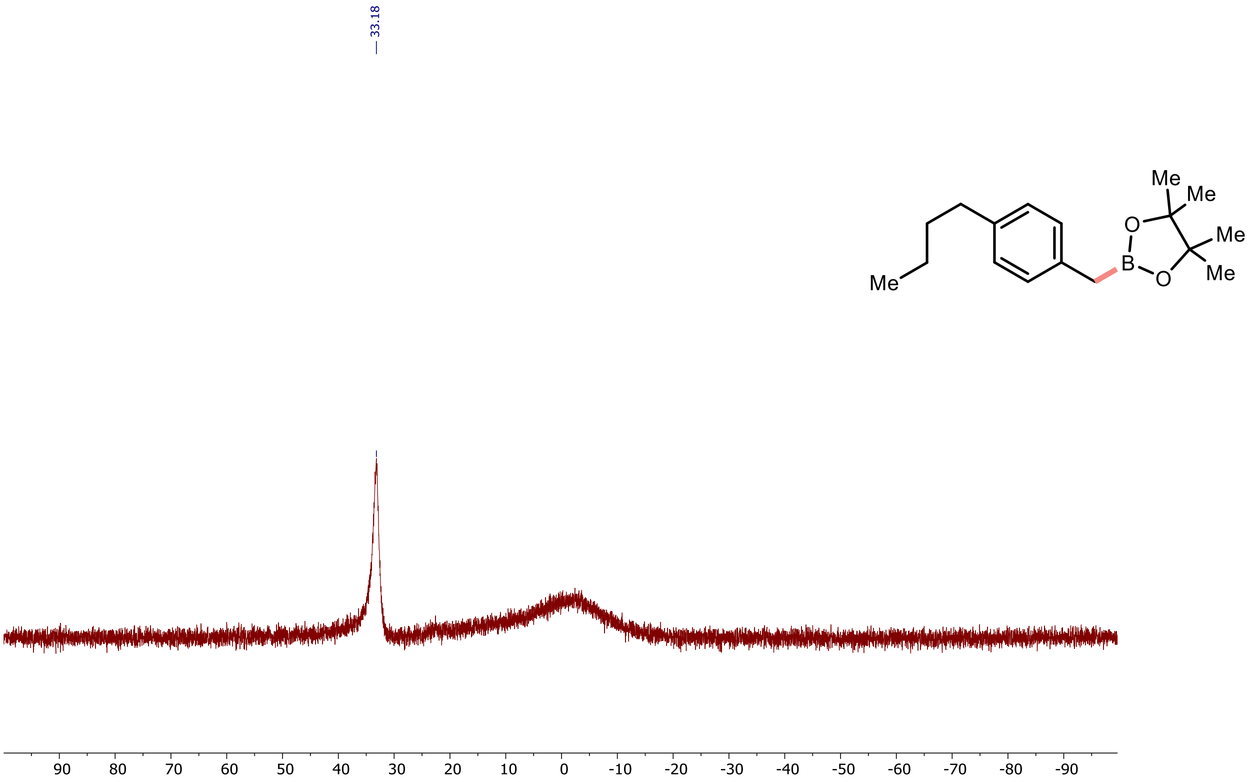

Supplementary Figure 6b-1 ^{1}H NMR (500 MHz, $CDCl_3$) 2-(4-(*Tert*-butyl)benzyl)-4,4,5,5-tetramethyl-1,3,2-dioxaborolane

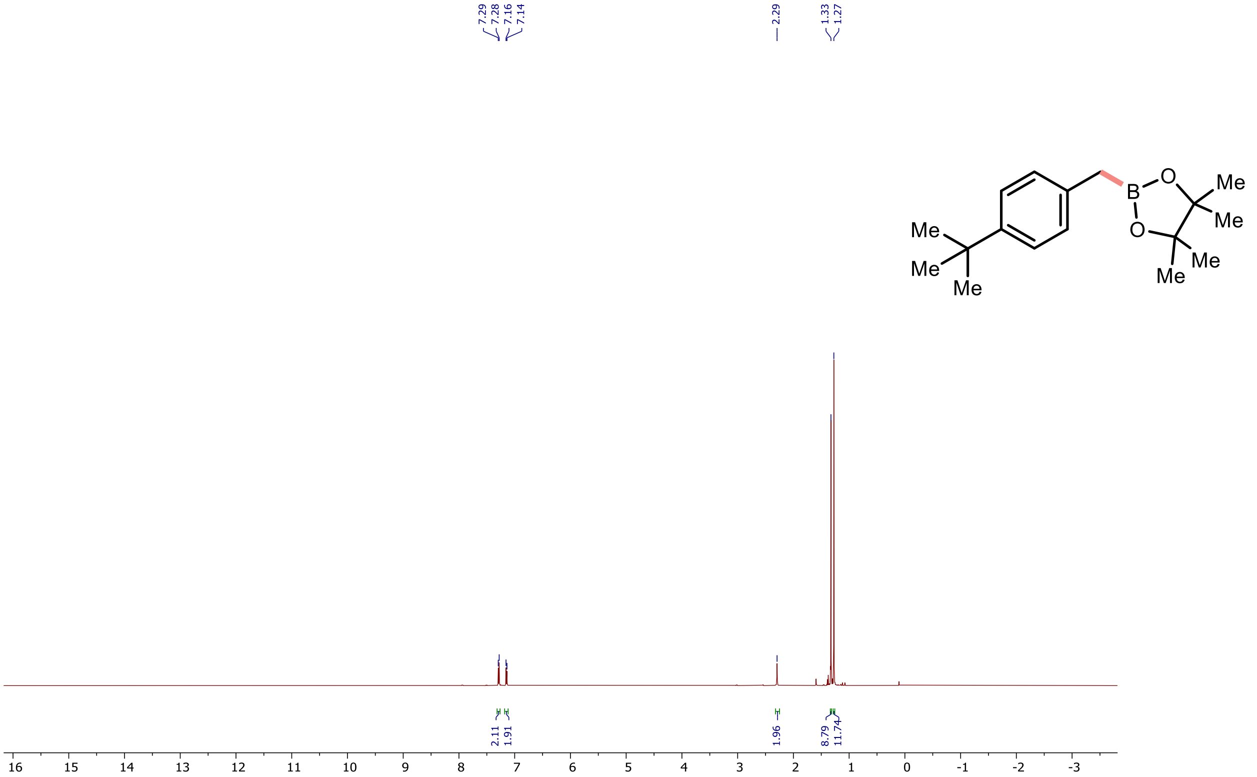

Supplementary Figure 6b-2 ^{13}C NMR (125 MHz, $CDCl_3$) 2-(4-(*Tert*-butyl)benzyl)-4,4,5,5-tetramethyl-1,3,2-dioxaborolane

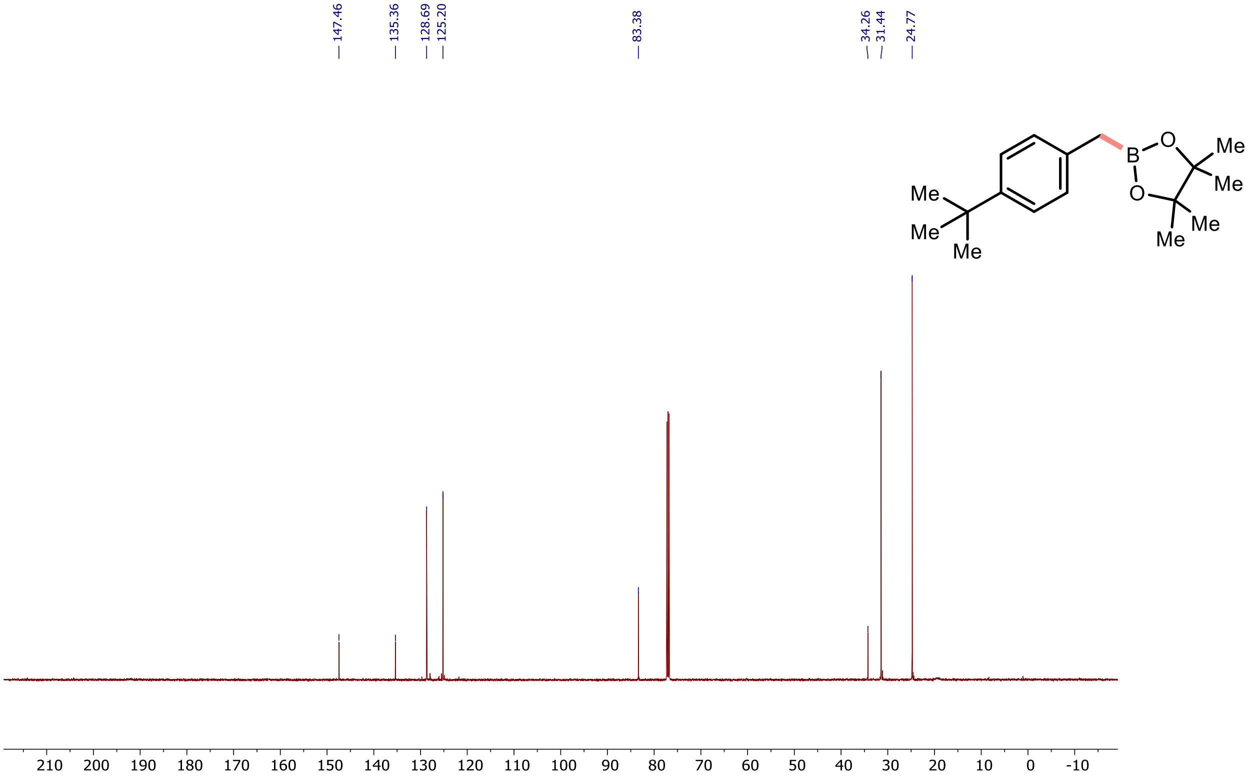

Supplementary Figure 6b-3 ^{11}B NMR (161 MHz, $CDCl_3$) 2-(4-(*Tert*-butyl)benzyl)-4,4,5,5-tetramethyl-1,3,2-dioxaborolane

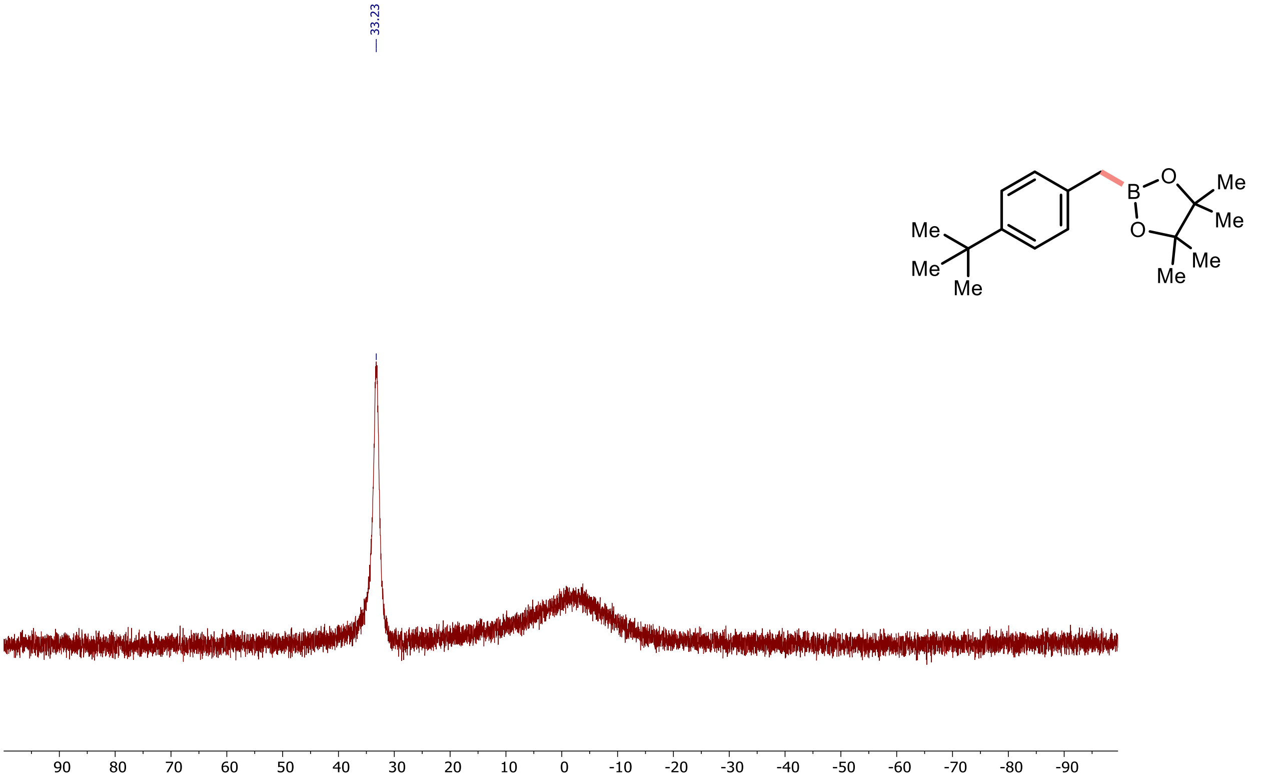

Supplementary Figure 7b-1 ^1H NMR (500 mHz, $CDCl_3$) 4,4,5,5-Tetramethyl-2-(3-methylbenzyl)-1,3,2-dioxaborolane

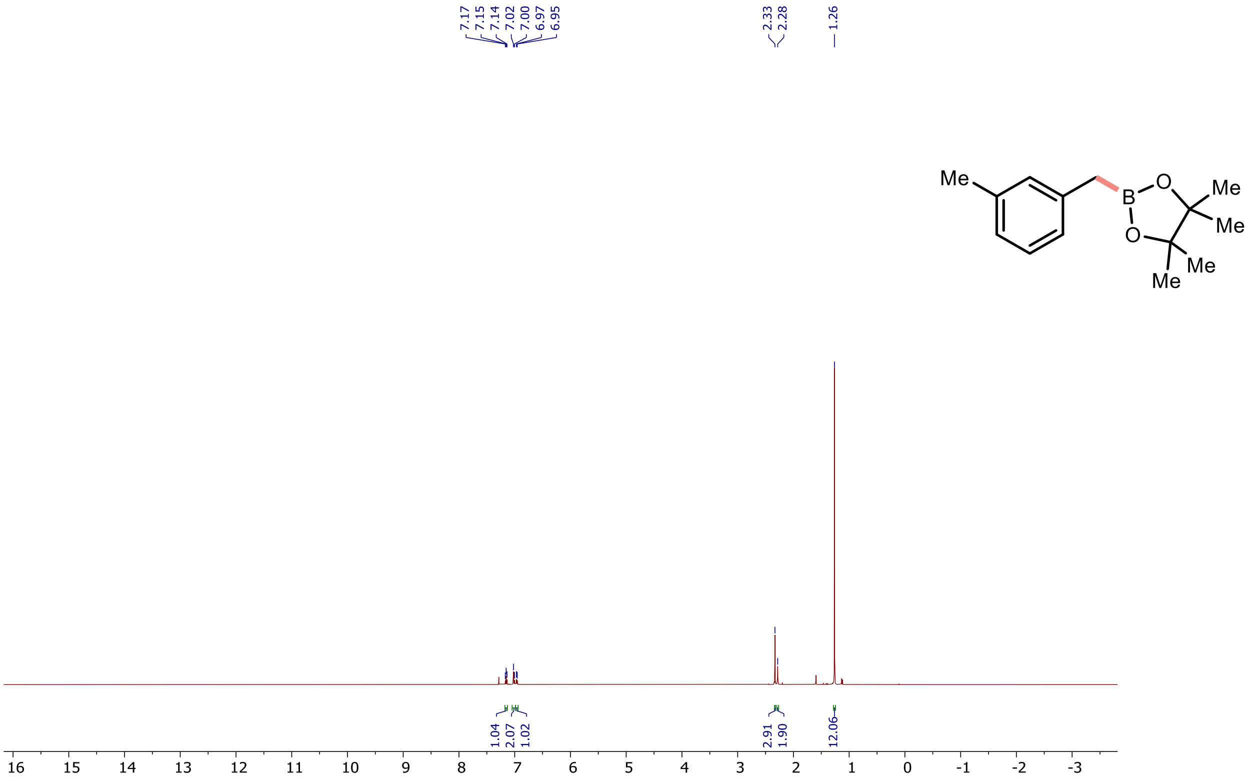

Supplementary Figure 7b-2 ^{13}C NMR (125 mHz, $CDCl_3$) 4,4,5,5-Tetramethyl-2-(3-methylbenzyl)-1,3,2-dioxaborolane

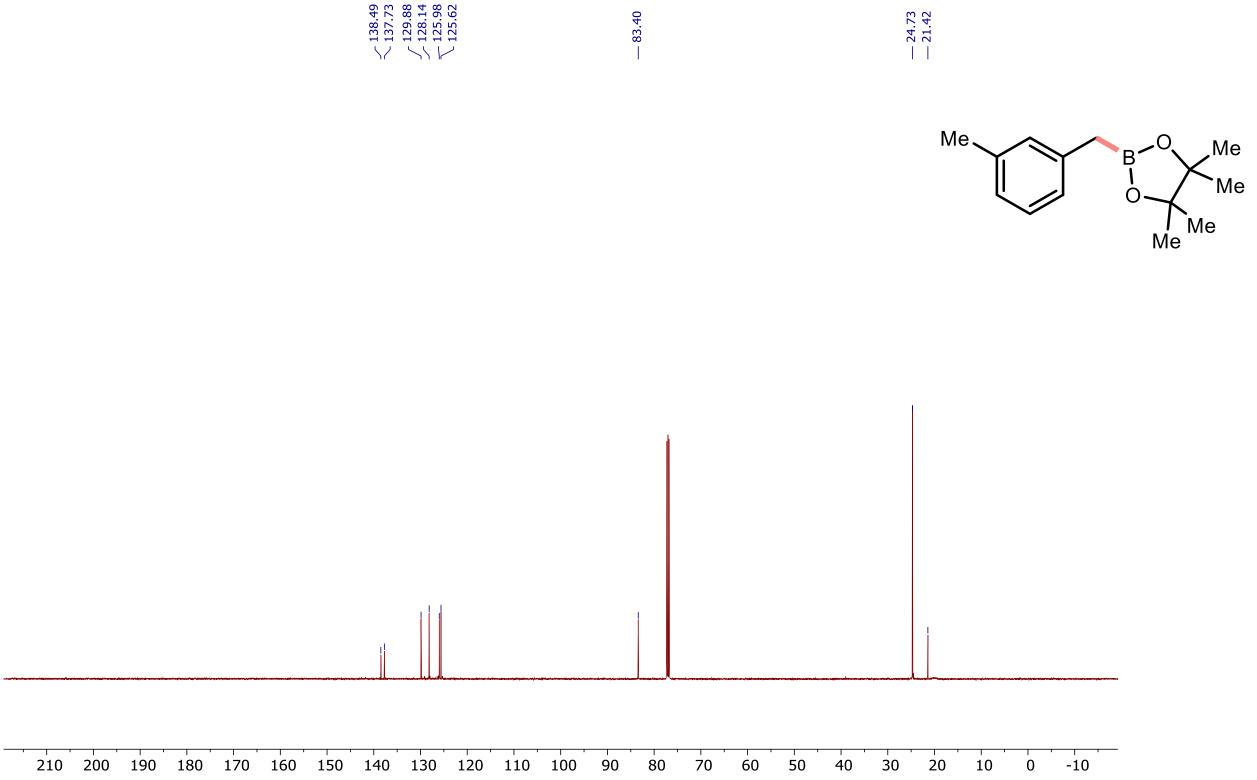

Supplementary Figure 7b-3 ^{11}B NMR (161 mHz, $CDCl_3$) 4,4,5,5-Tetramethyl-2-(3-methylbenzyl)-1,3,2-dioxaborolane

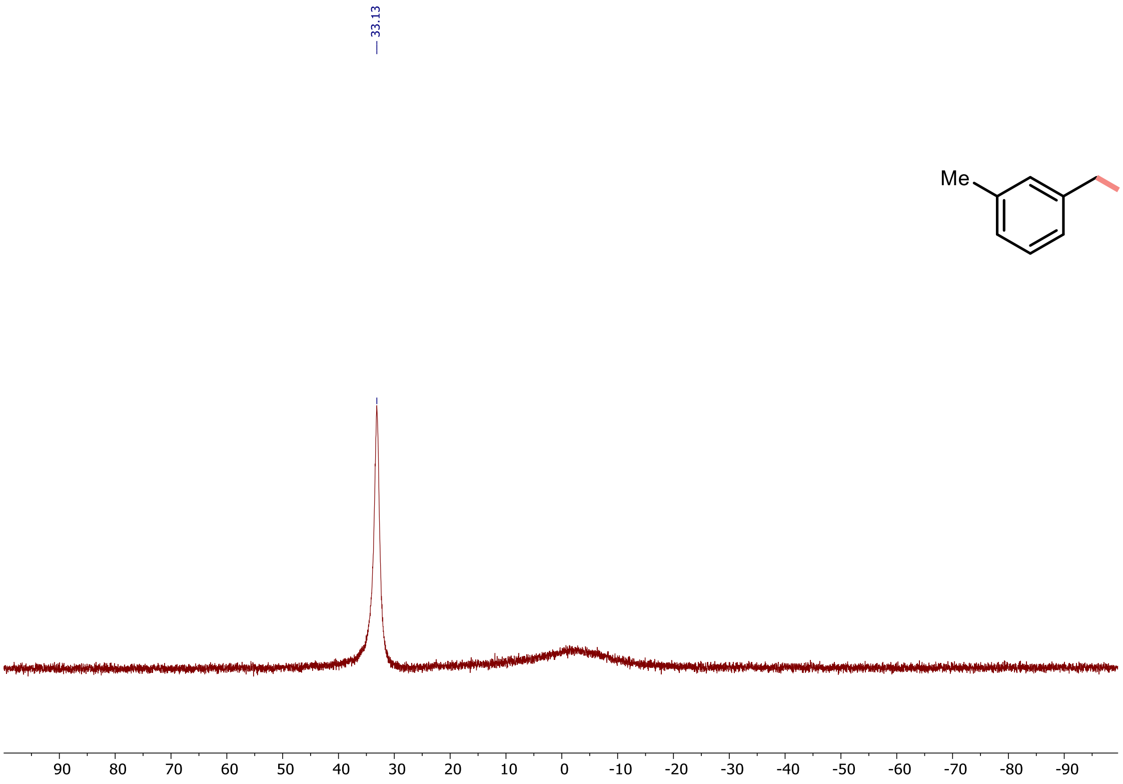

Supplementary Figure 8b-1 ^{1}H NMR (500 MHz, $CDCl_3$) 2-(3,5-Dimethylbenzyl)-4,4,5,5-tetramethyl-1,3,2-dioxaborolane

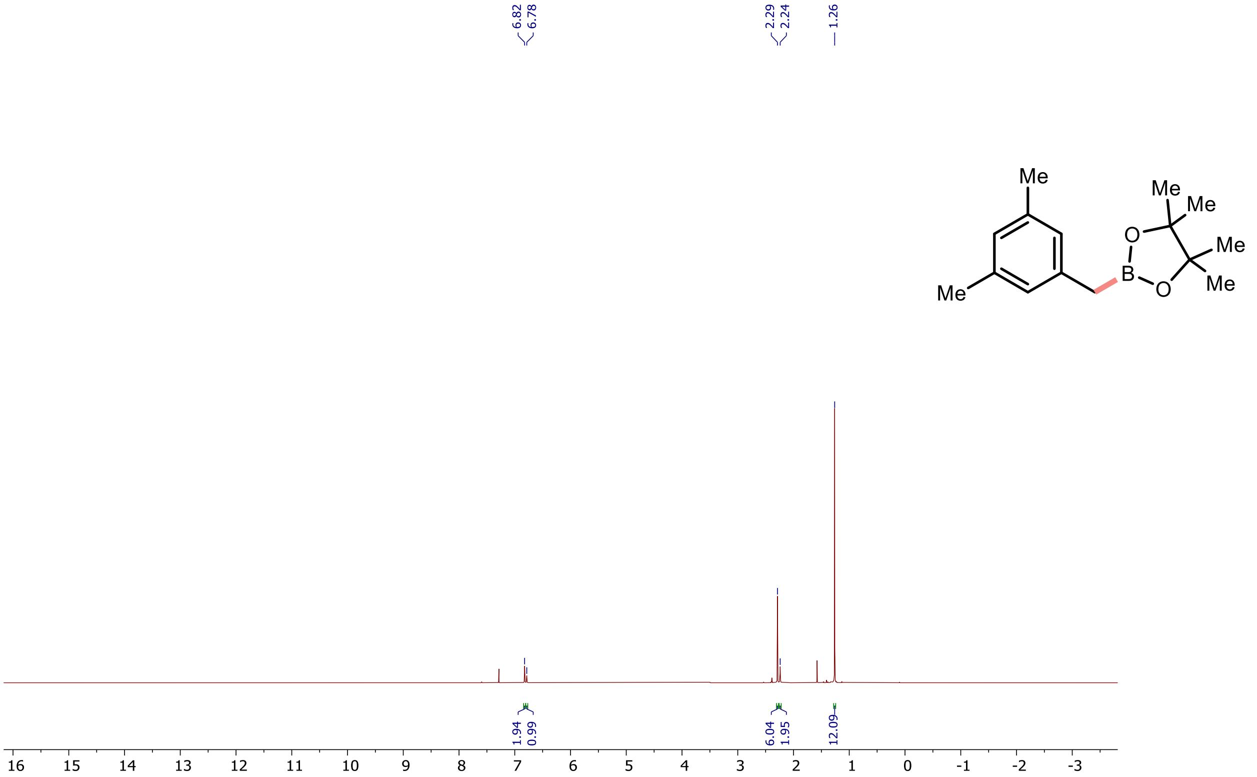

Supplementary Figure 8b-2 ^{13}C NMR (125 MHz, $CDCl_3$) 2-(3,5-Dimethylbenzyl)-4,4,5,5-tetramethyl-1,3,2-dioxaborolane

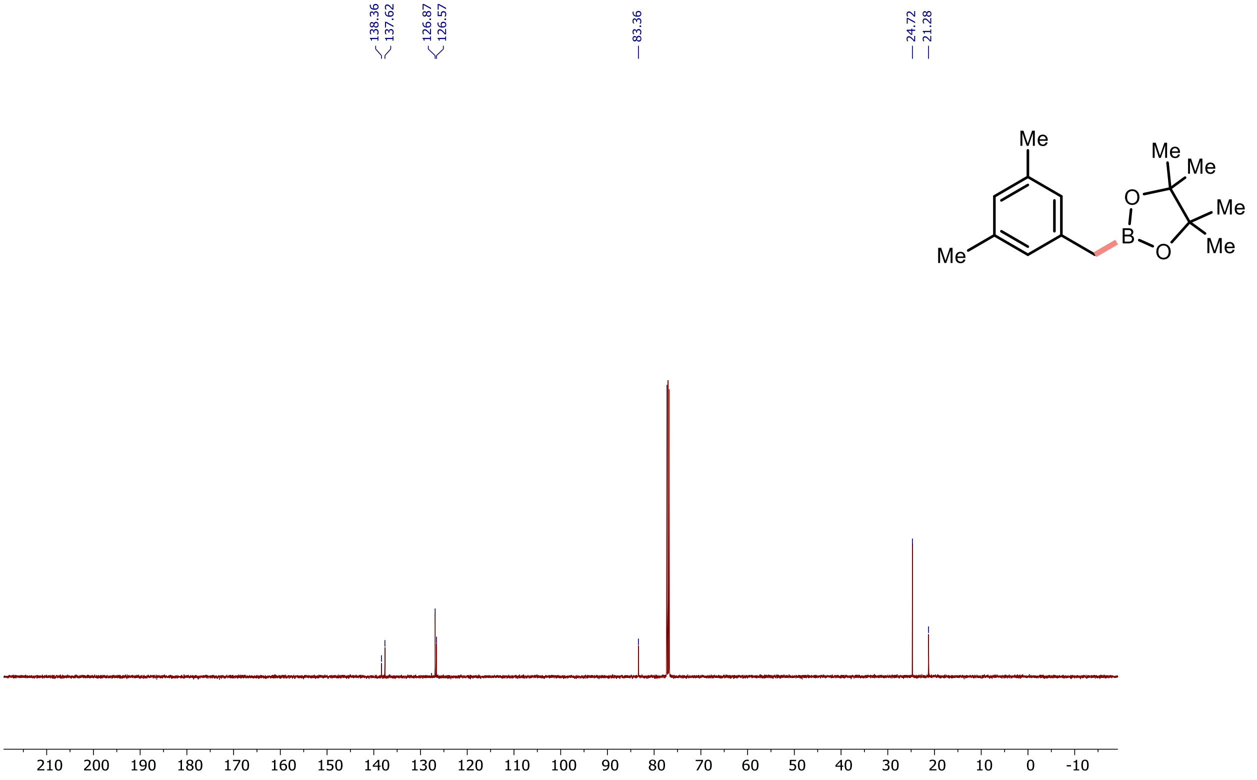

Supplementary Figure 8b-3 ^{11}B NMR (161 MHz, $CDCl_3$) 2-(3,5-Dimethylbenzyl)-4,4,5,5-tetramethyl-1,3,2-dioxaborolane

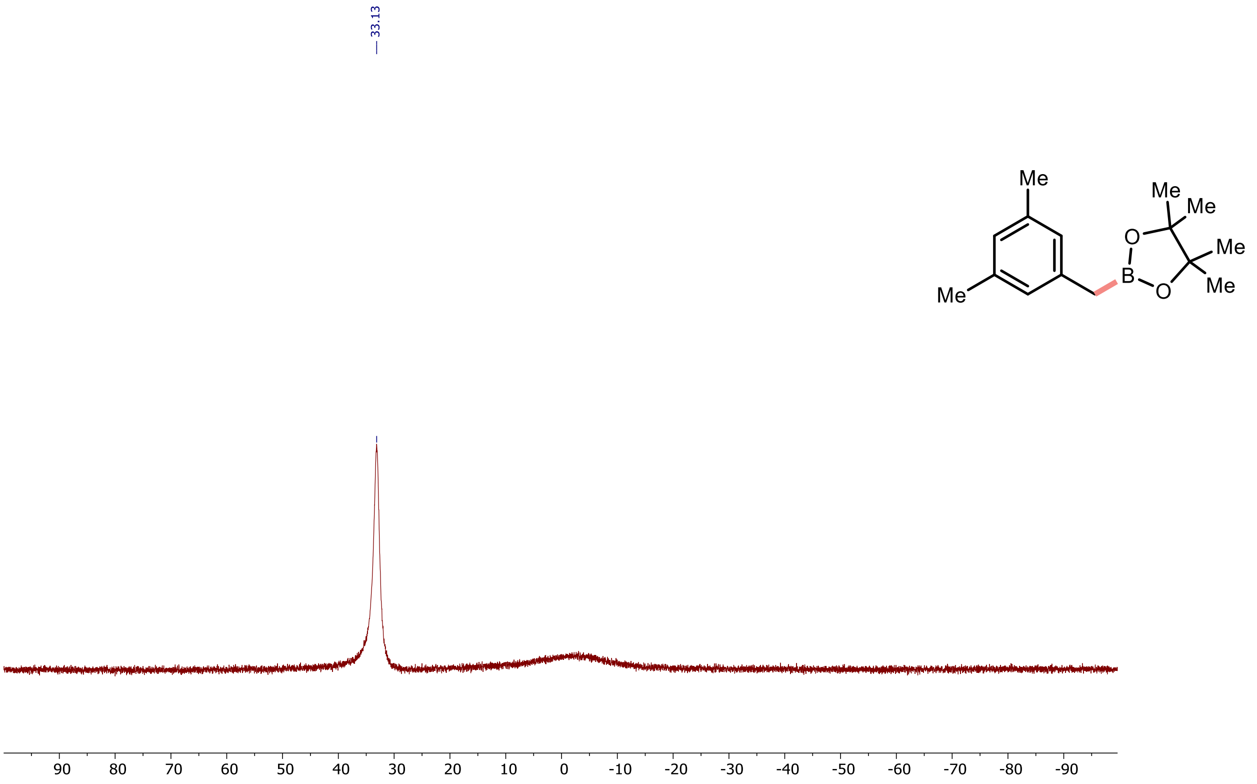

Supplementary Figure 9b-1 ^{1}H NMR (500 MHz, $CDCl_3$) 4,4,5,5-Tetramethyl-2-(2-methylbenzyl)-1,3,2-dioxaborolane

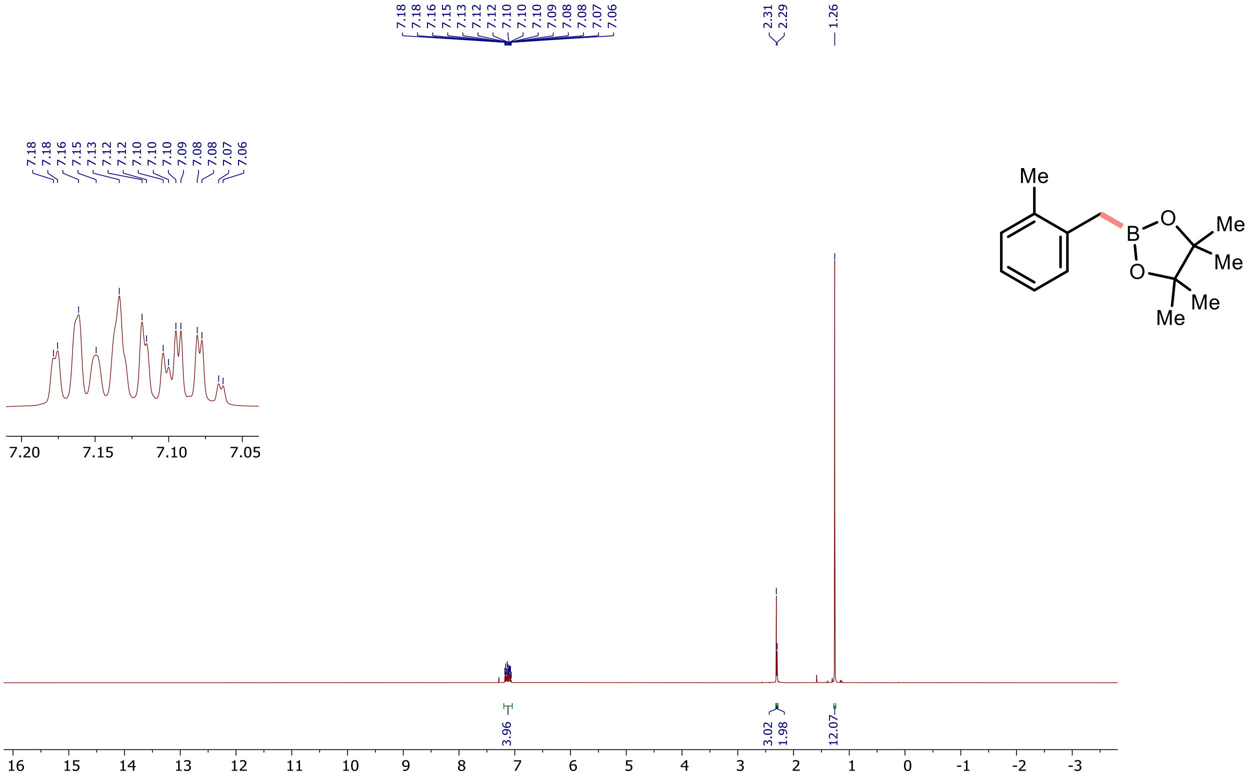

Supplementary Figure 9b-2 ^{13}C NMR (125 MHz, $CDCl_3$) 4,4,5,5-Tetramethyl-2-(2-methylbenzyl)-1,3,2-dioxaborolane

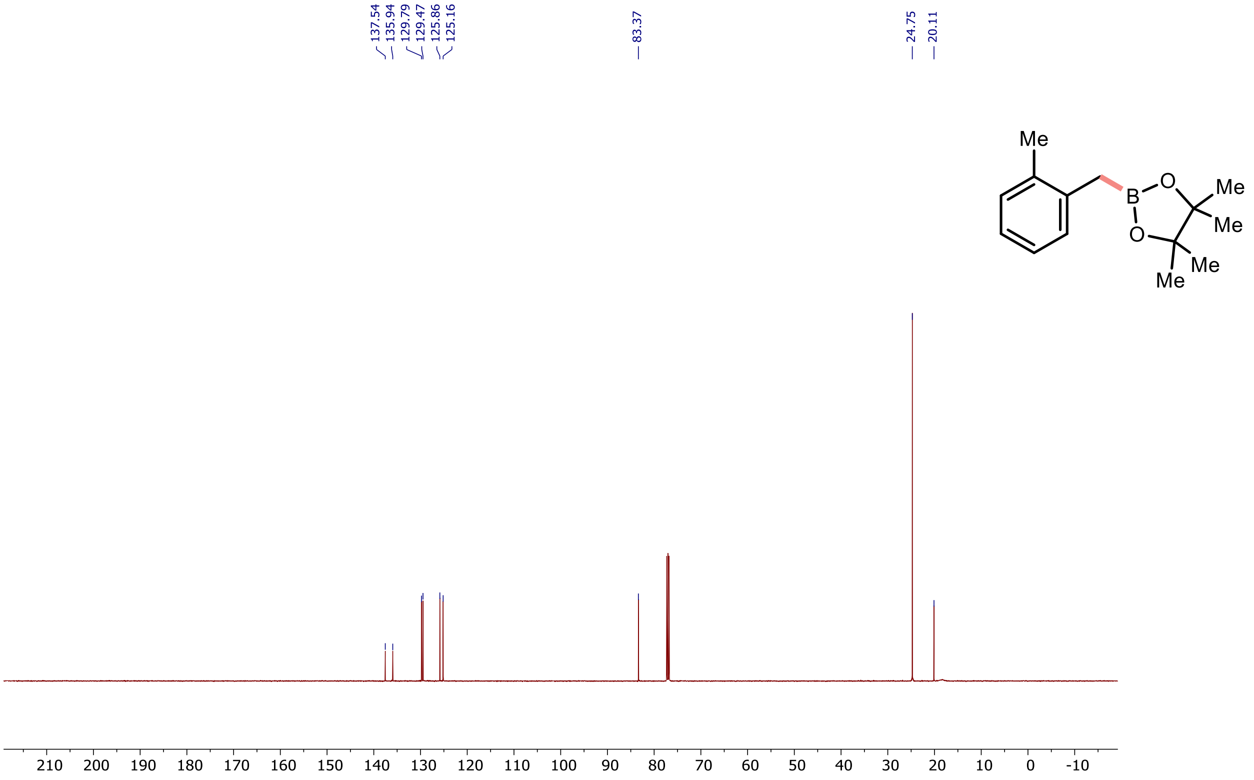

Supplementary Figure 9b-3 ^{11}B NMR (161 MHz, $CDCl_3$) 4,4,5,5-Tetramethyl-2-(2-methylbenzyl)-1,3,2-dioxaborolane

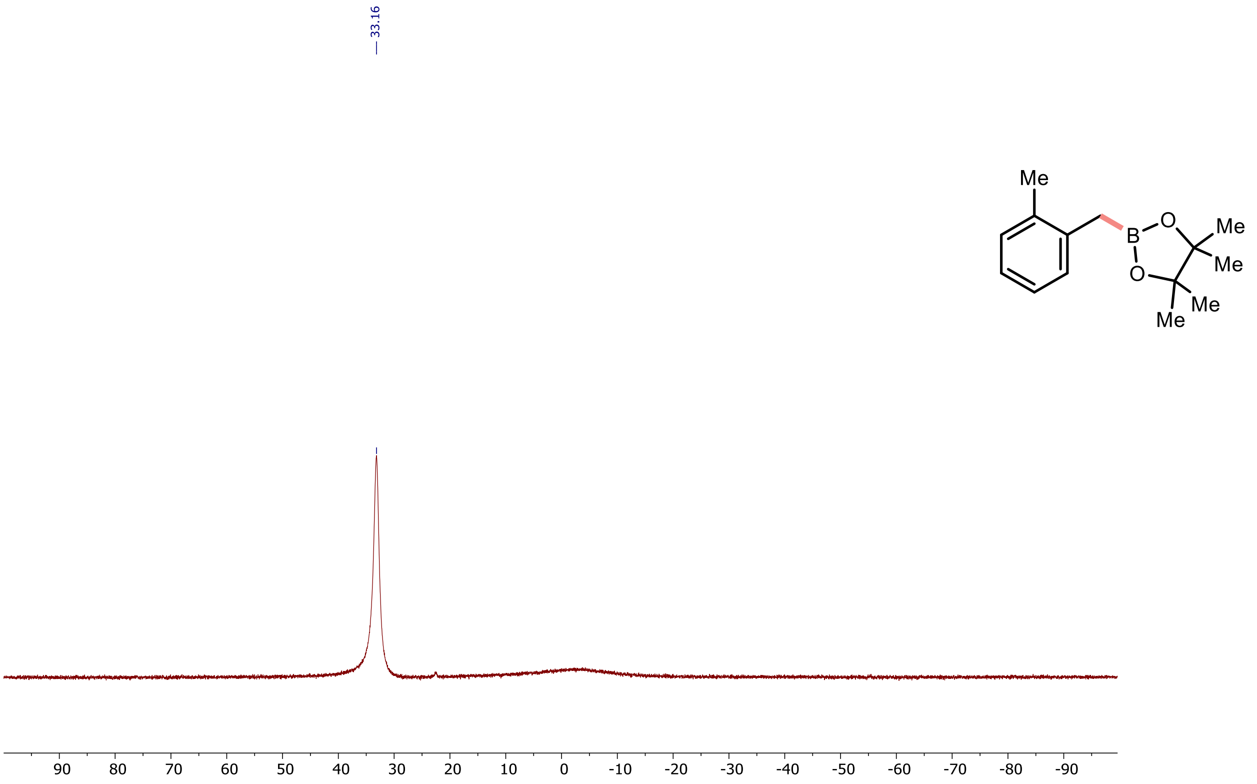

Supplementary Figure 10b-1 ^{1}H NMR (500 MHz, $CDCl_3$) 2-([1,1'-Biphenyl]-3-ylmethyl)-4,4,5,5-tetramethyl-1,3,2-dioxaborolane

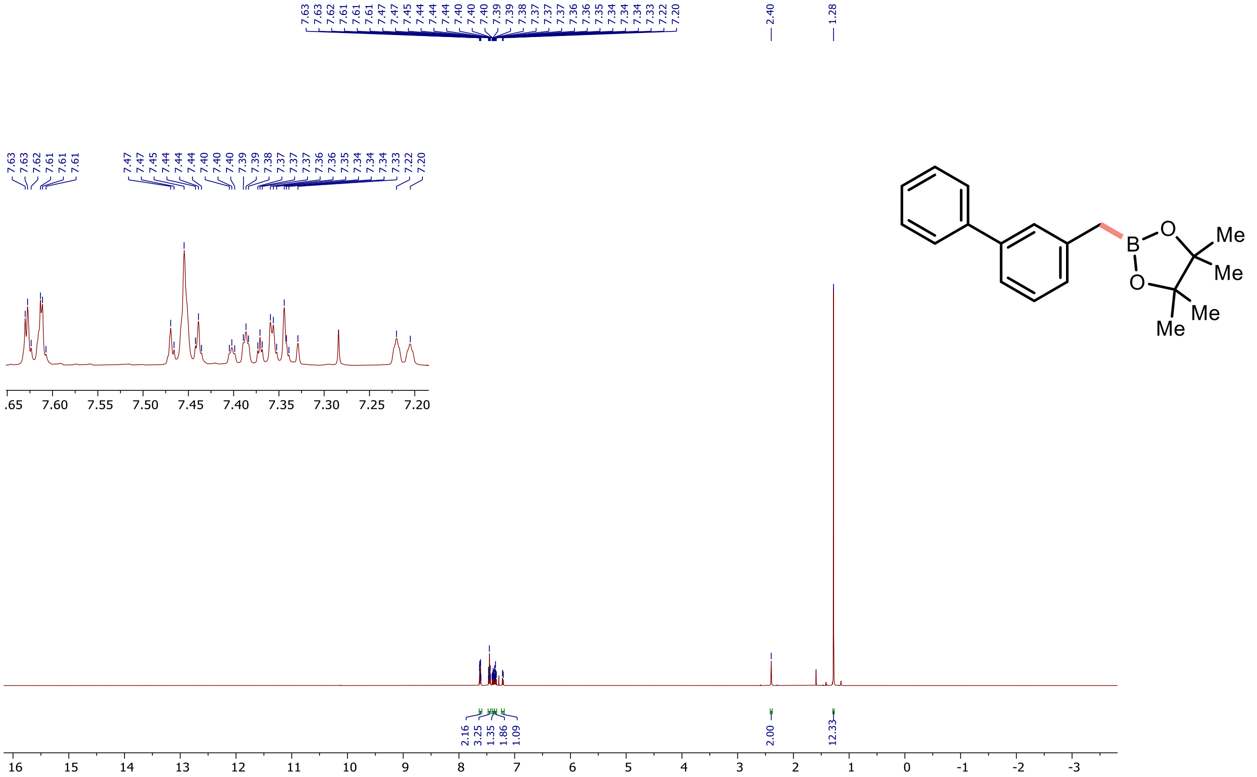

Supplementary Figure 10b-2 ^{13}C NMR (125 MHz, $CDCl_3$) 2-([1,1'-Biphenyl]-3-ylmethyl)-4,4,5,5-tetramethyl-1,3,2-dioxaborolane

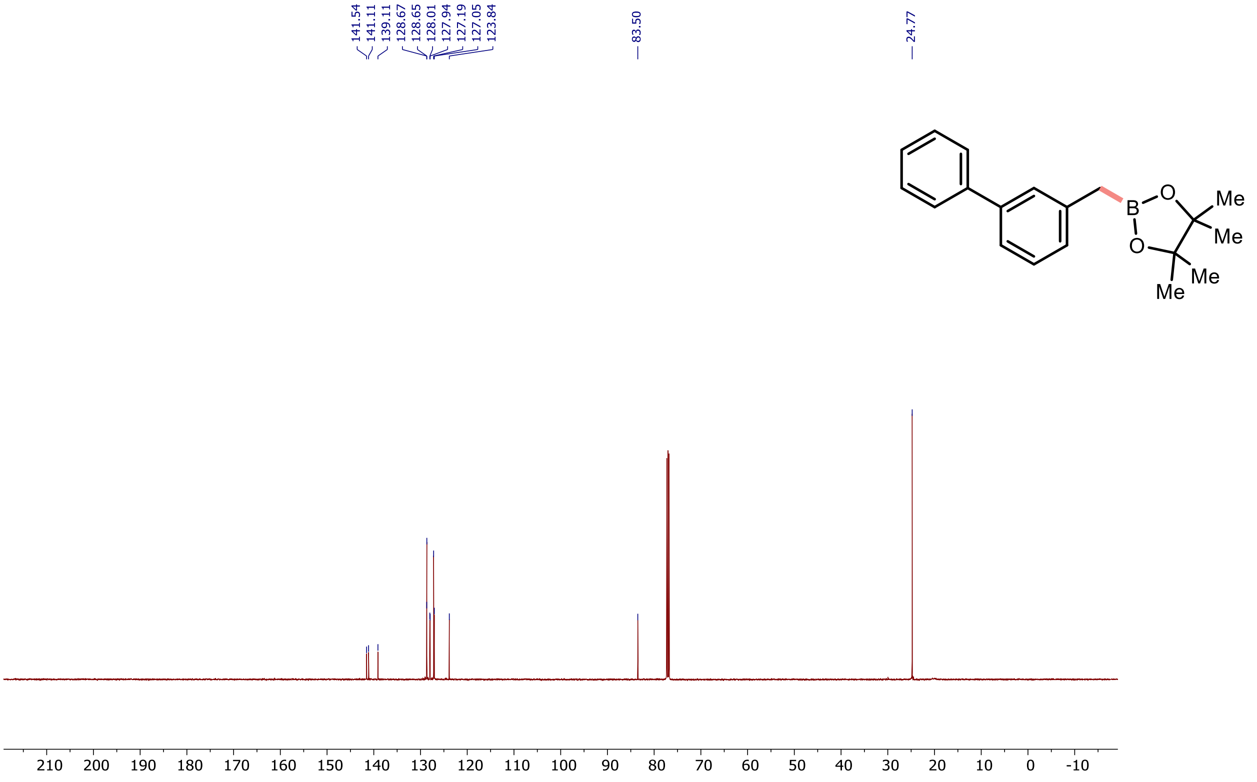

Supplementary Figure 10b-3 ^{11}B NMR (161 MHz, $CDCl_3$) 2-([1,1'-Biphenyl]-3-ylmethyl)-4,4,5,5-tetramethyl-1,3,2-dioxaborolane

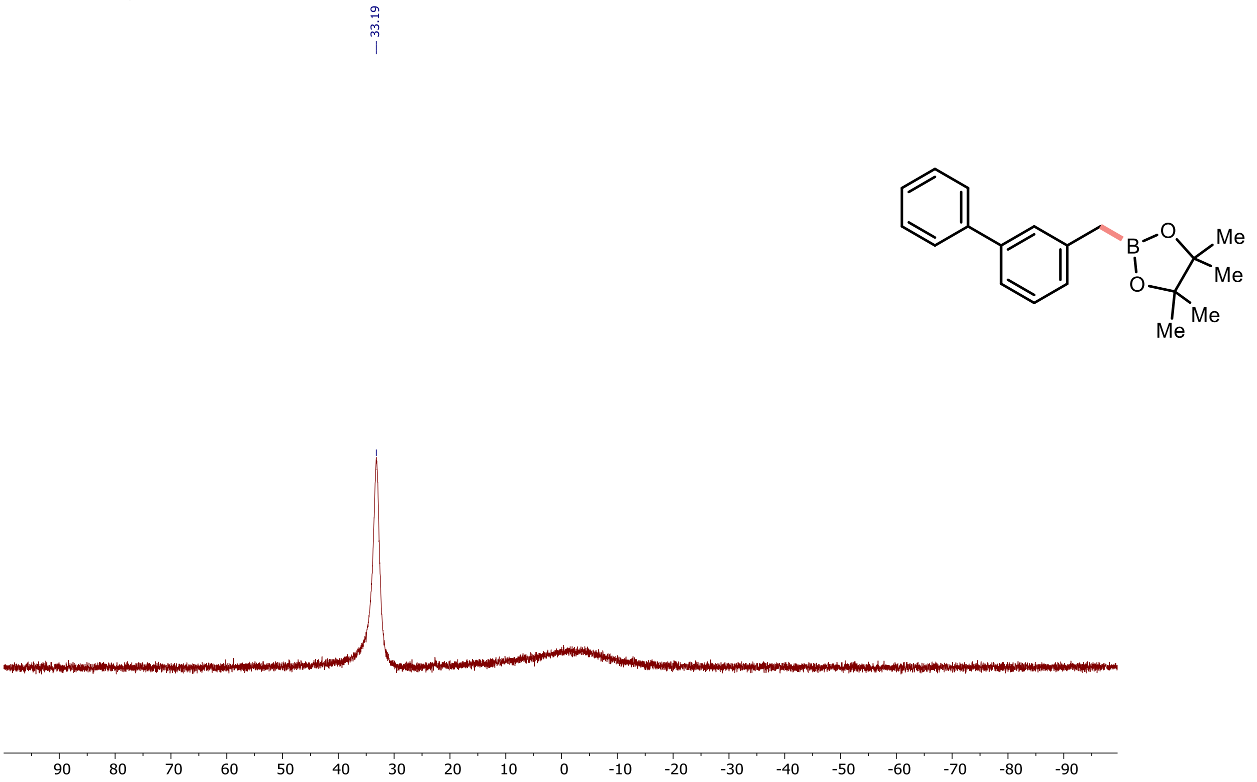

Supplementary Figure 11b-1 ^1H NMR (500 MHz, $CDCl_3$) 2-((9*H*-Fluoren-2-yl)methyl)-4,4,5,5-tetramethyl-1,3,2-dioxaborolane

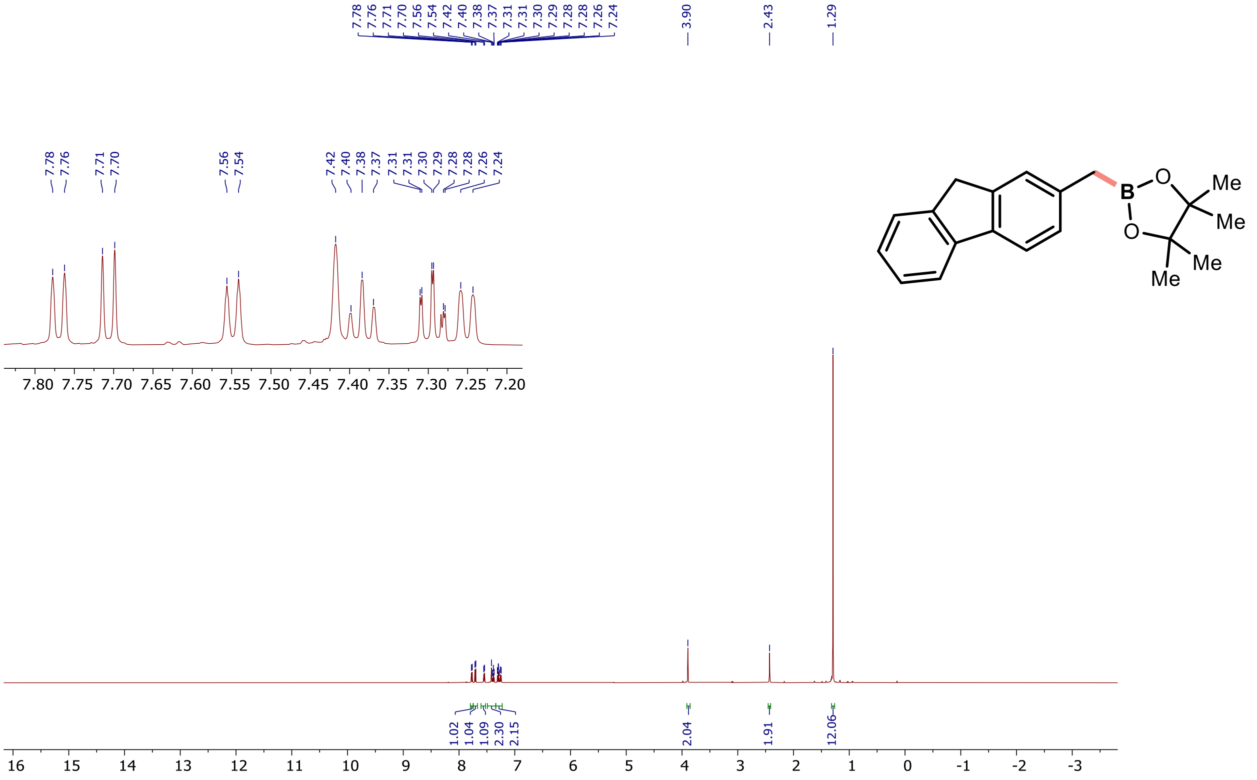

Supplementary Figure 11b-2 ^{13}C NMR (125 MHz, $CDCl_3$) 2-((9*H*-Fluoren-2-yl)methyl)-4,4,5,5-tetramethyl-1,3,2-dioxaborolane

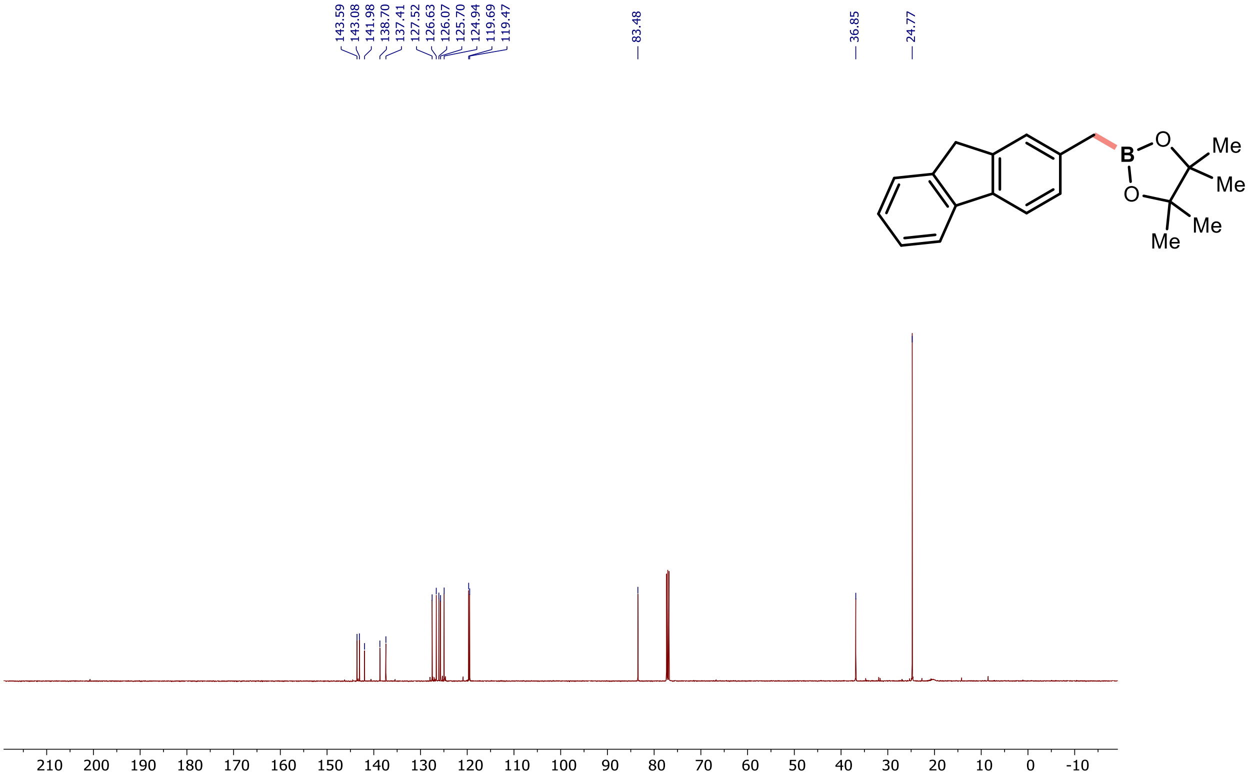

Supplementary Figure 11b-3 ^{11}B NMR (161 MHz, $CDCl_3$) 2-((9*H*-Fluoren-2-yl)methyl)-4,4,5,5-tetramethyl-1,3,2-dioxaborolane

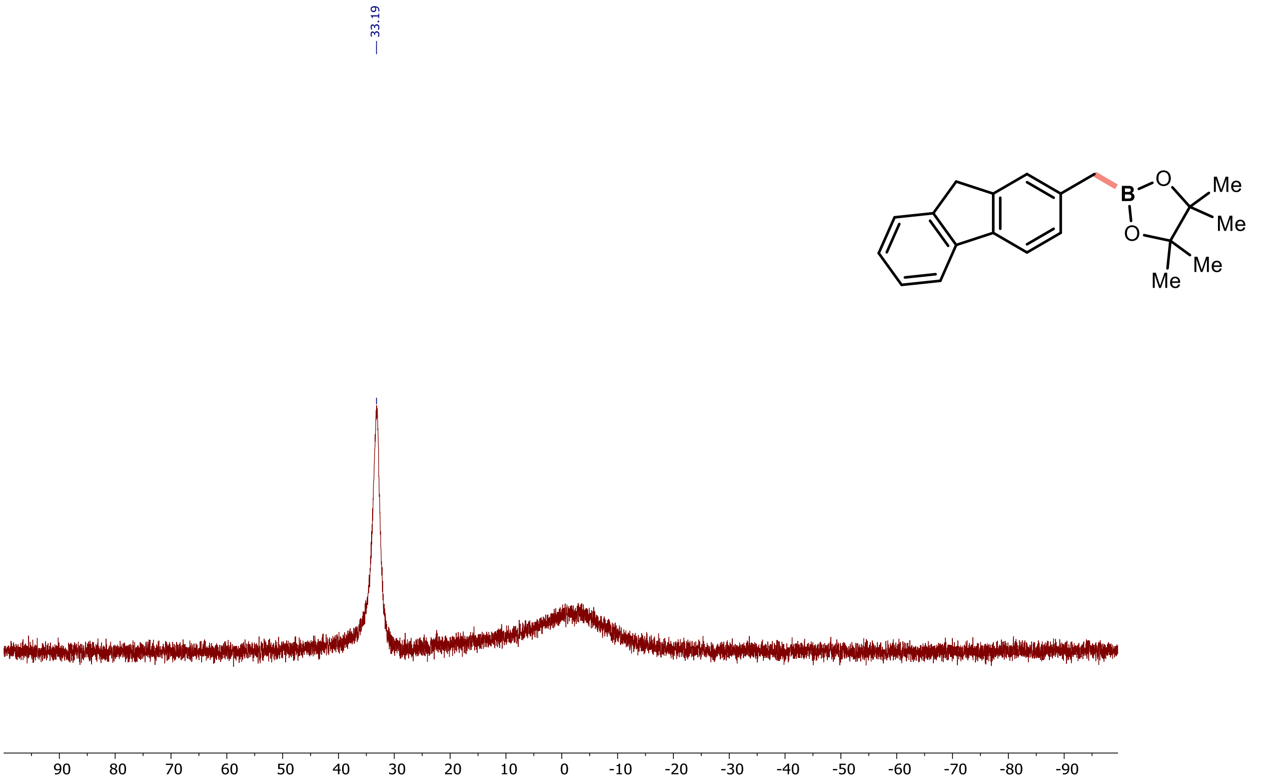

Supplementary Figure 12b-1 ^{1}H NMR (500 MHz, $CDCl_3$) 4,4,5,5-Tetramethyl-2-(naphthalen-1-ylmethyl)-1,3,2-dioxaborolane

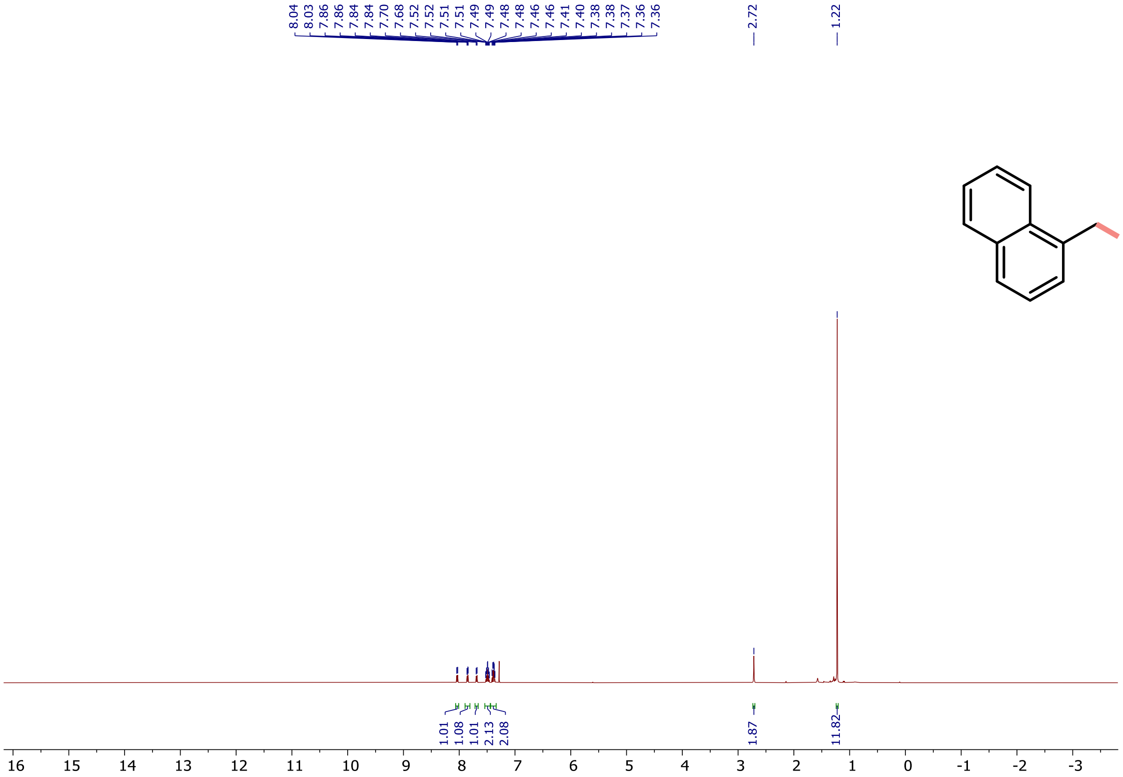

Supplementary Figure 12b-2 ^{13}C NMR (125 MHz, $CDCl_3$) 4,4,5,5-Tetramethyl-2-(naphthalen-1-ylmethyl)-1,3,2-dioxaborolane

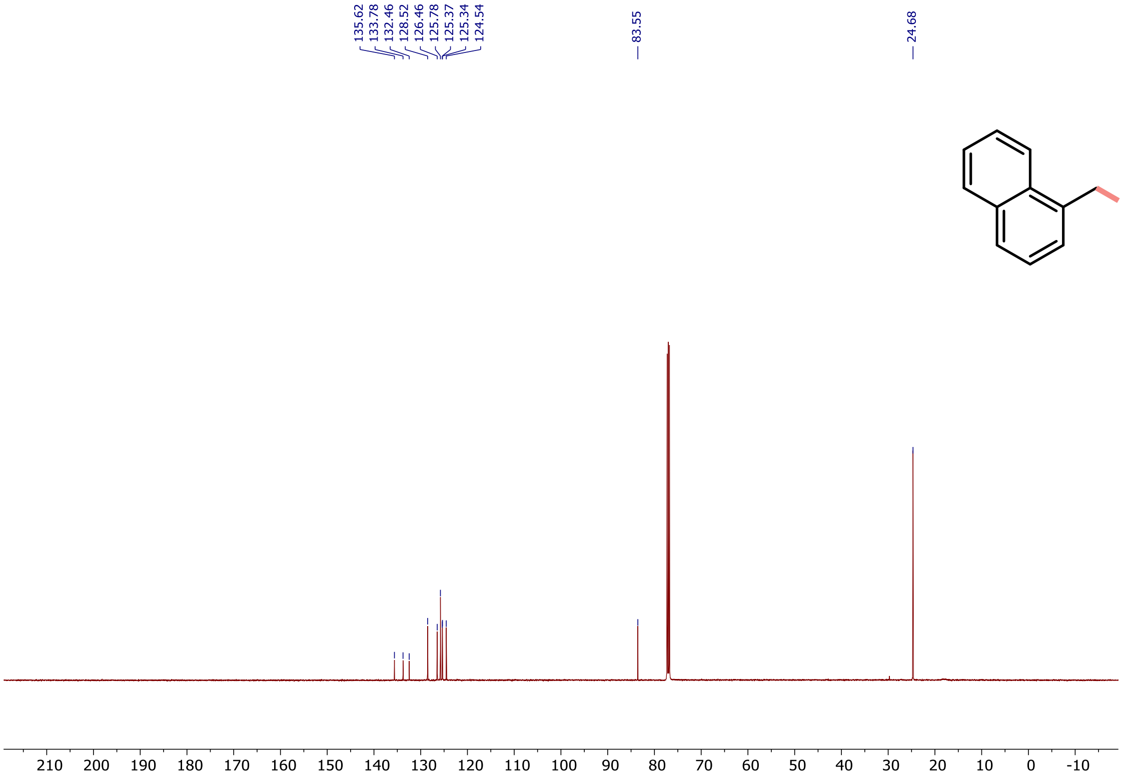

Supplementary Figure 12b-3 ^{11}B NMR (161 MHz, $CDCl_3$) 4,4,5,5-Tetramethyl-2-(naphthalen-1-ylmethyl)-1,3,2-dioxaborolane

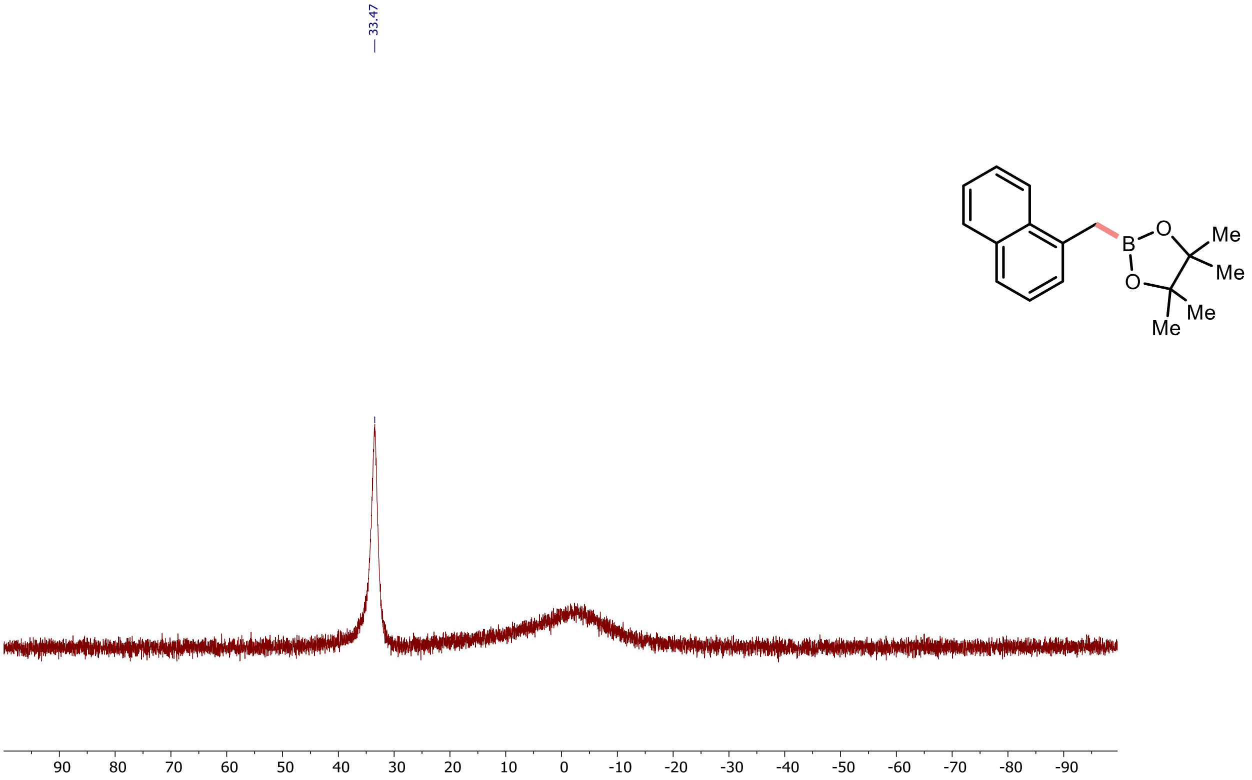

Supplementary Figure 13b-1 ^{1}H NMR (500 MHz, $CDCl_3$) 4,4,5,5-Tetramethyl-2-(naphthalen-2-ylmethyl)-1,3,2-dioxaborolane

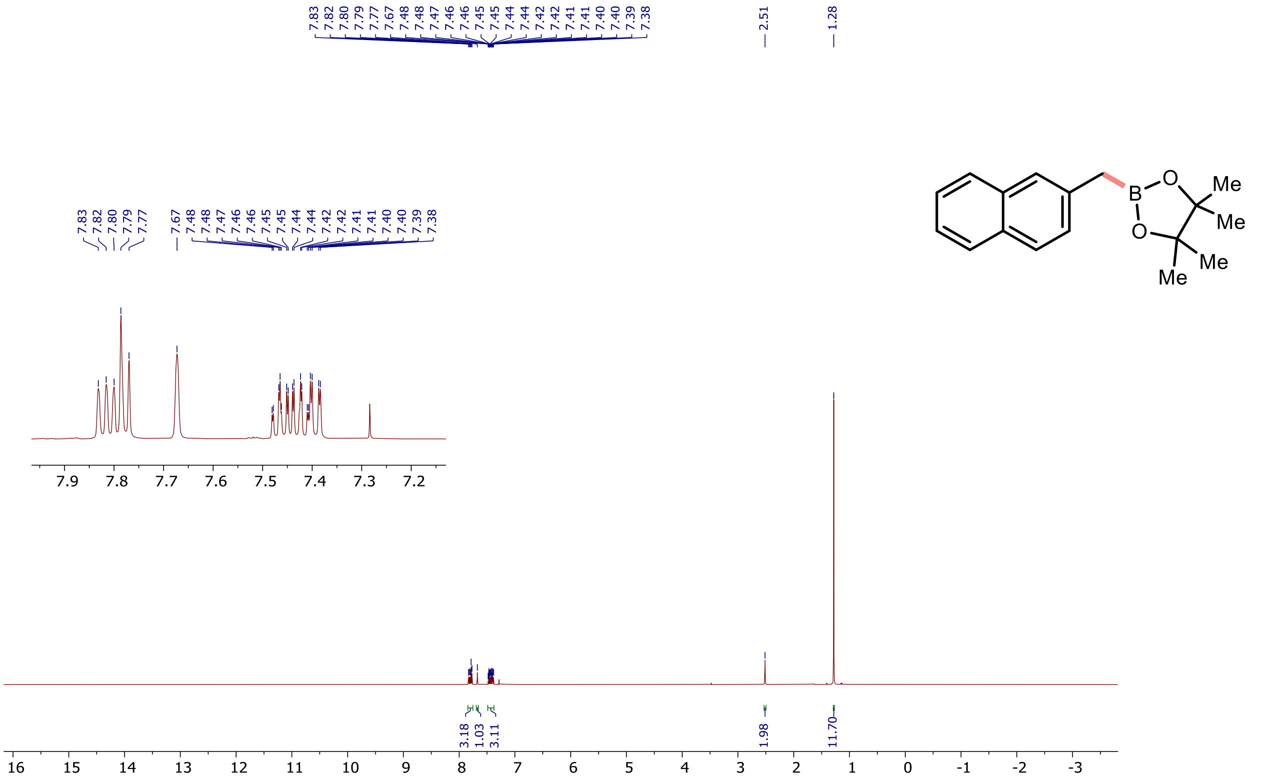

Supplementary Figure 13b-2 ^{13}C NMR (125 MHz, $CDCl_3$) 4,4,5,5-Tetramethyl-2-(naphthalen-2-ylmethyl)-1,3,2-dioxaborolane

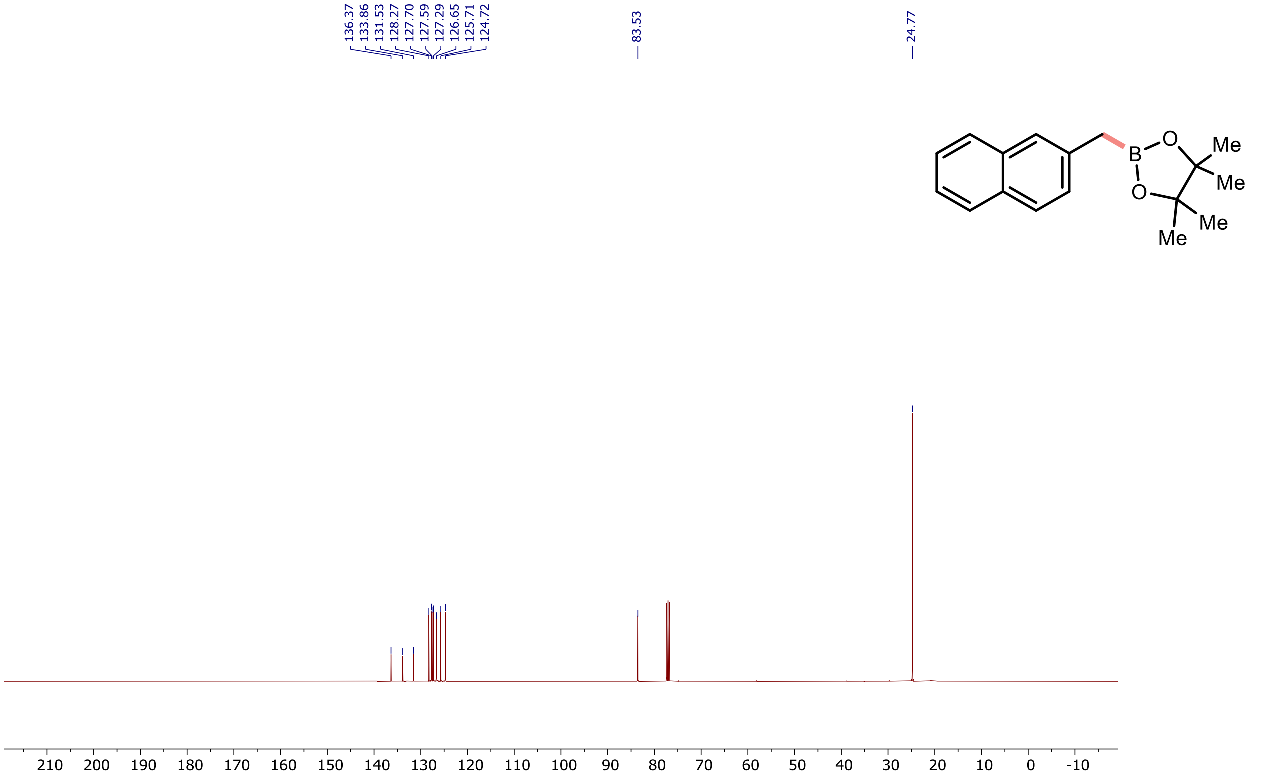

Supplementary Figure 13b-3 ^{11}B NMR (161 MHz, $CDCl_3$) 4,4,5,5-Tetramethyl-2-(naphthalen-2-ylmethyl)-1,3,2-dioxaborolane

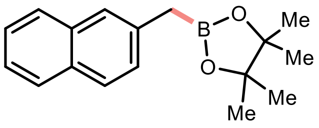

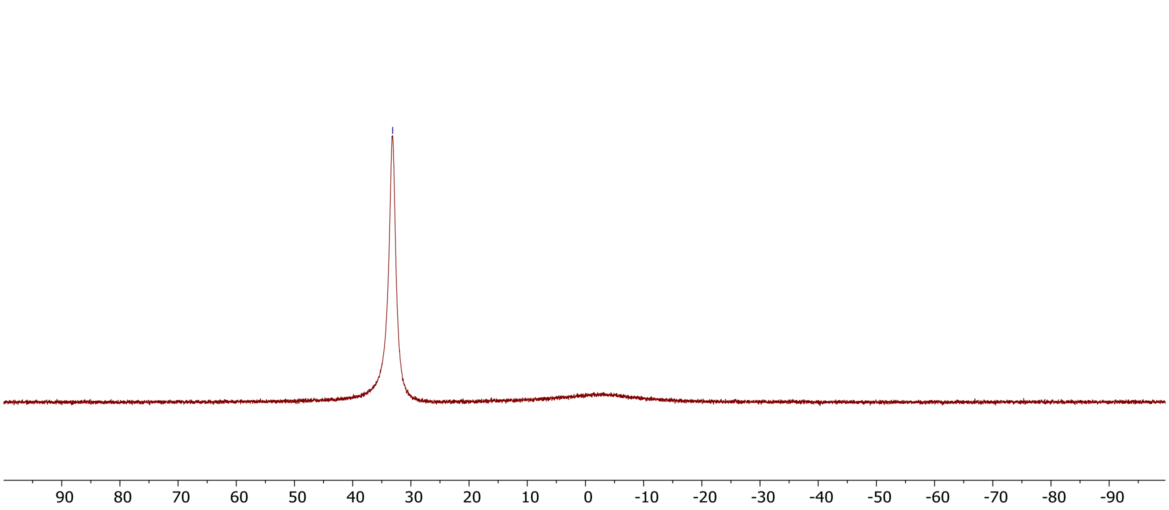

Supplementary Figure 14b-1 ^{1}H NMR (500 MHz, $CDCl_3$) 2-(4-Fluorobenzyl)-4,4,5,5-tetramethyl-1,3,2-dioxaborolane

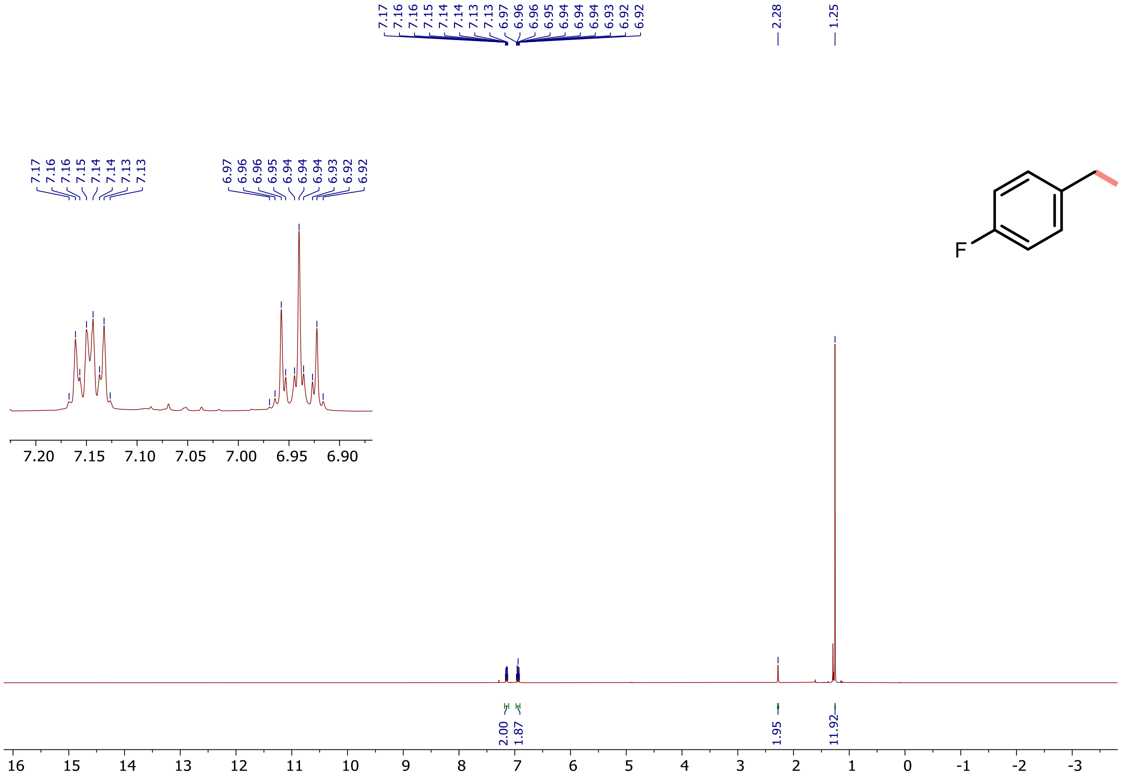

Supplementary Figure 14b-2 ^{13}C NMR (125 MHz, $CDCl_3$) 2-(4-Fluorobenzyl)-4,4,5,5-tetramethyl-1,3,2-dioxaborolane

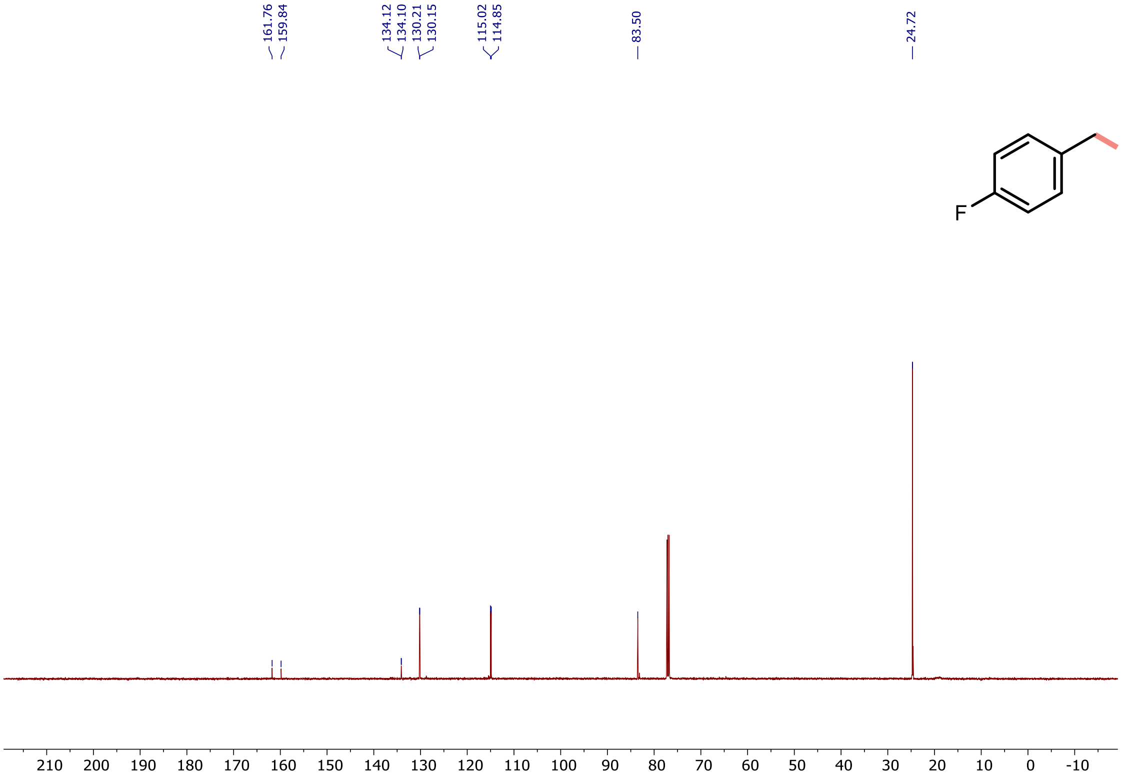

Supplementary Figure 14b-3 ^{19}F NMR (471 MHz, $CDCl_3$) 2-(4-Fluorobenzyl)-4,4,5,5-tetramethyl-1,3,2-dioxaborolane

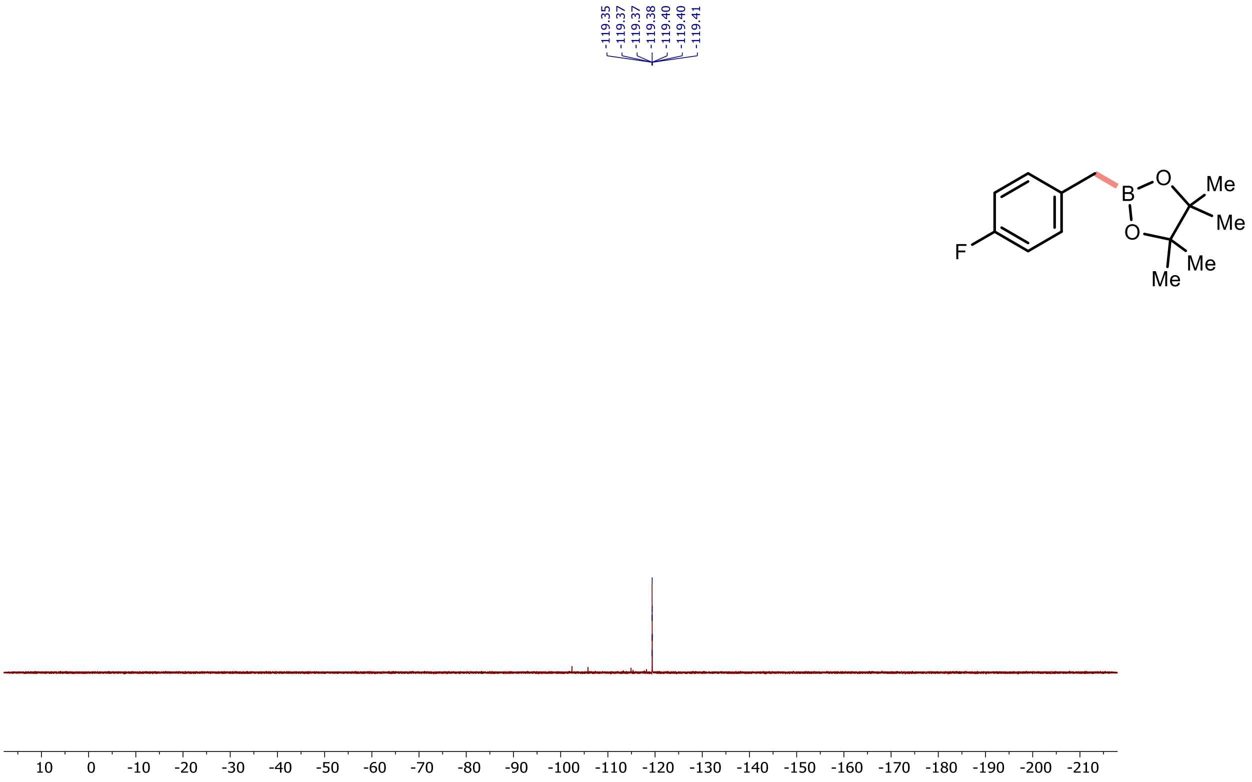

Supplementary Figure 14b-4 ^{11}B NMR (161 MHz, $CDCl_3$) 2-(4-Fluorobenzyl)-4,4,5,5-tetramethyl-1,3,2-dioxaborolane

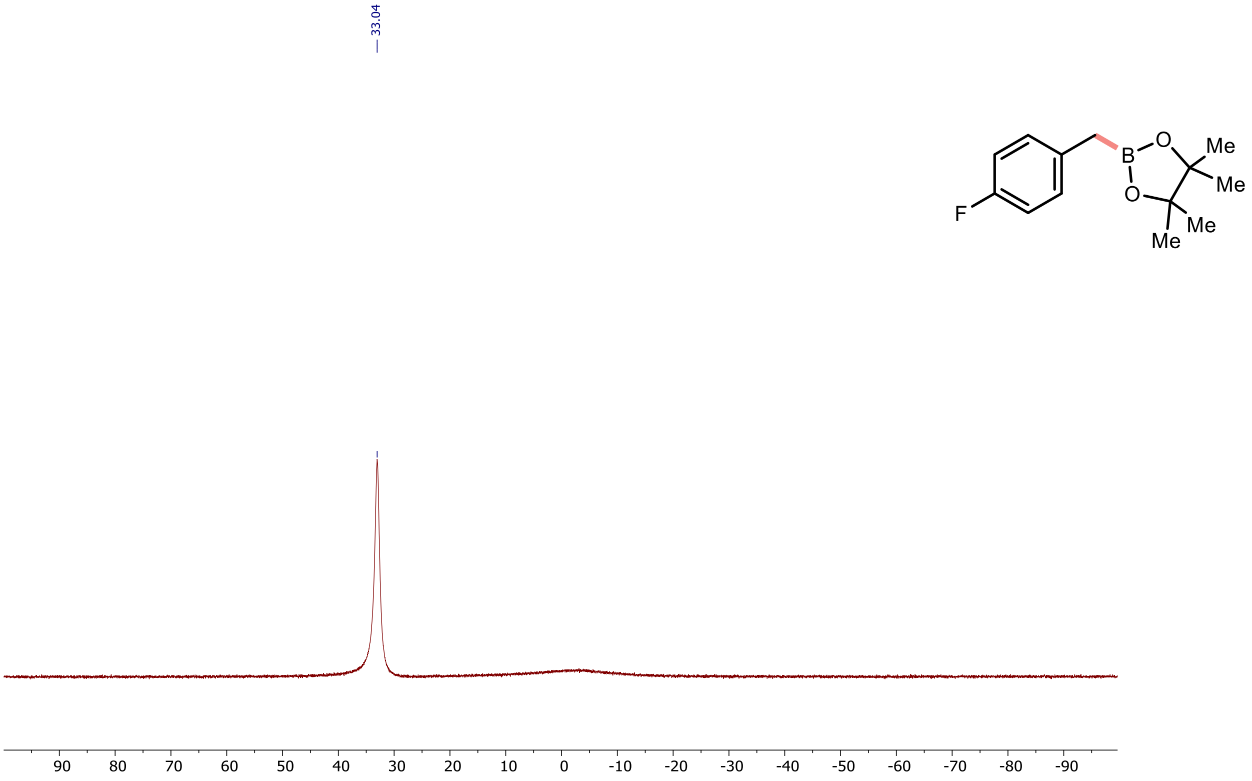

Supplementary Figure 15b-1 ^{1}H NMR (500 MHz, $CDCl_3$) 2-(2-Fluorobenzyl)-4,4,5,5-tetramethyl-1,3,2-dioxaborolane

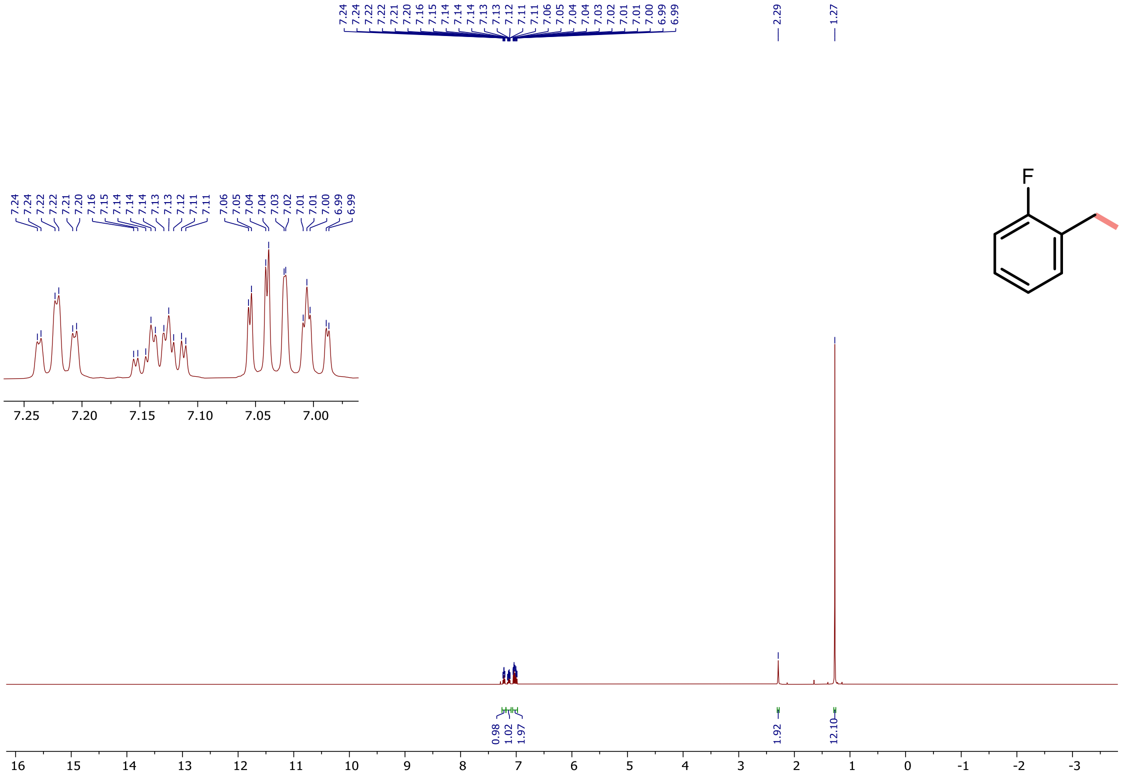

Supplementary Figure 15b-2 ^{13}C NMR (125 MHz, $CDCl_3$) 2-(2-Fluorobenzyl)-4,4,5,5-tetramethyl-1,3,2-dioxaborolane

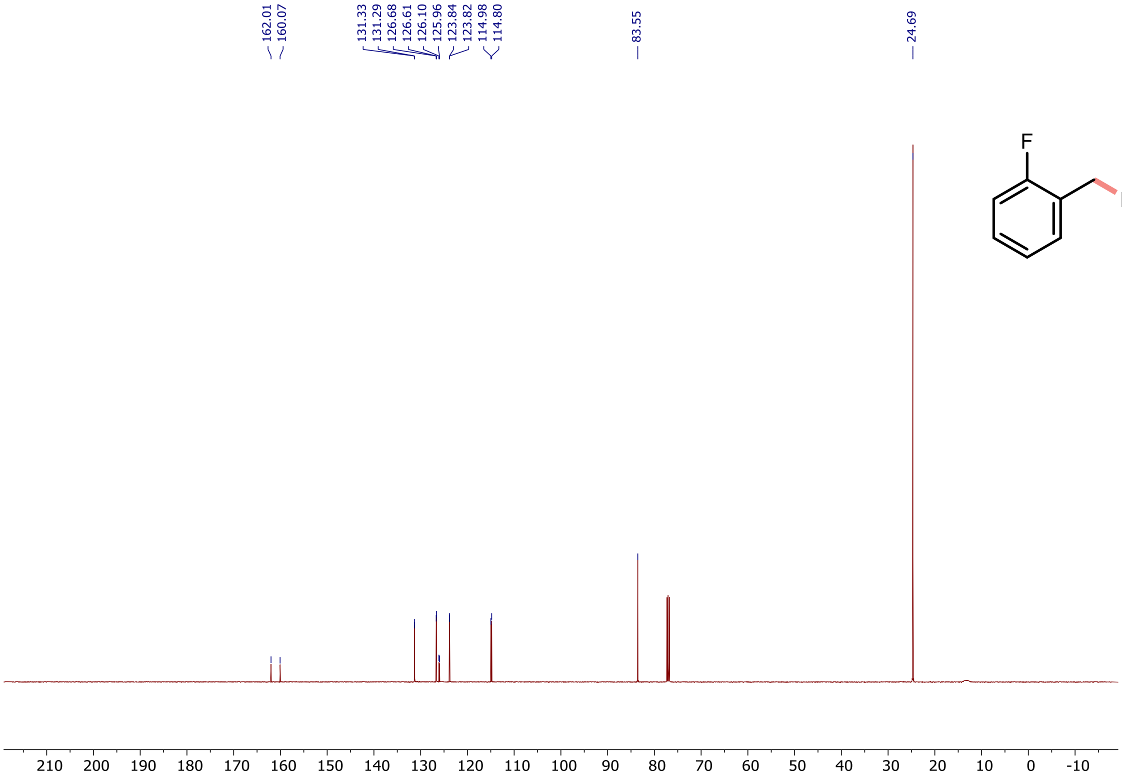

Supplementary Figure 15b-3 ^{19}F NMR (471 MHz, $CDCl_3$) 2-(2-Fluorobenzyl)-4,4,5,5-tetramethyl-1,3,2-dioxaborolane

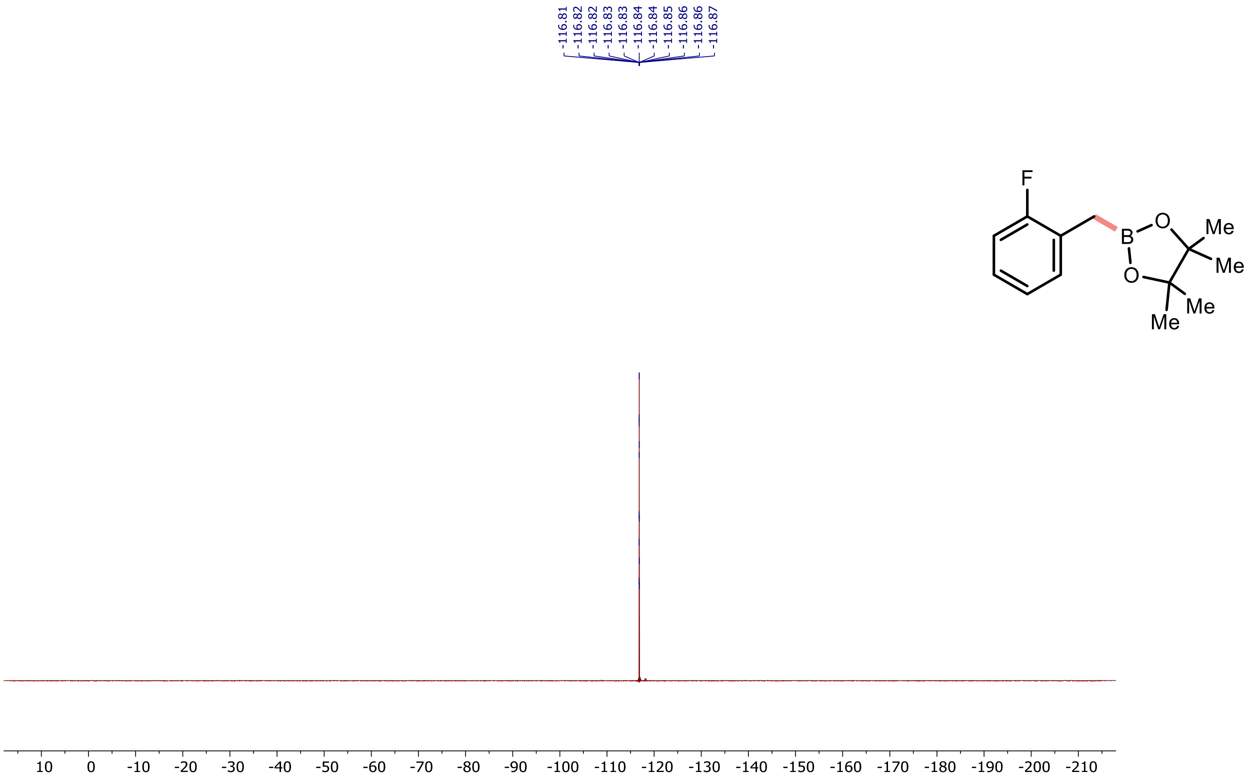

Supplementary Figure 15b-4 ^{11}B NMR (161 MHz, $CDCl_3$) 2-(2-Fluorobenzyl)-4,4,5,5-tetramethyl-1,3,2-dioxaborolane

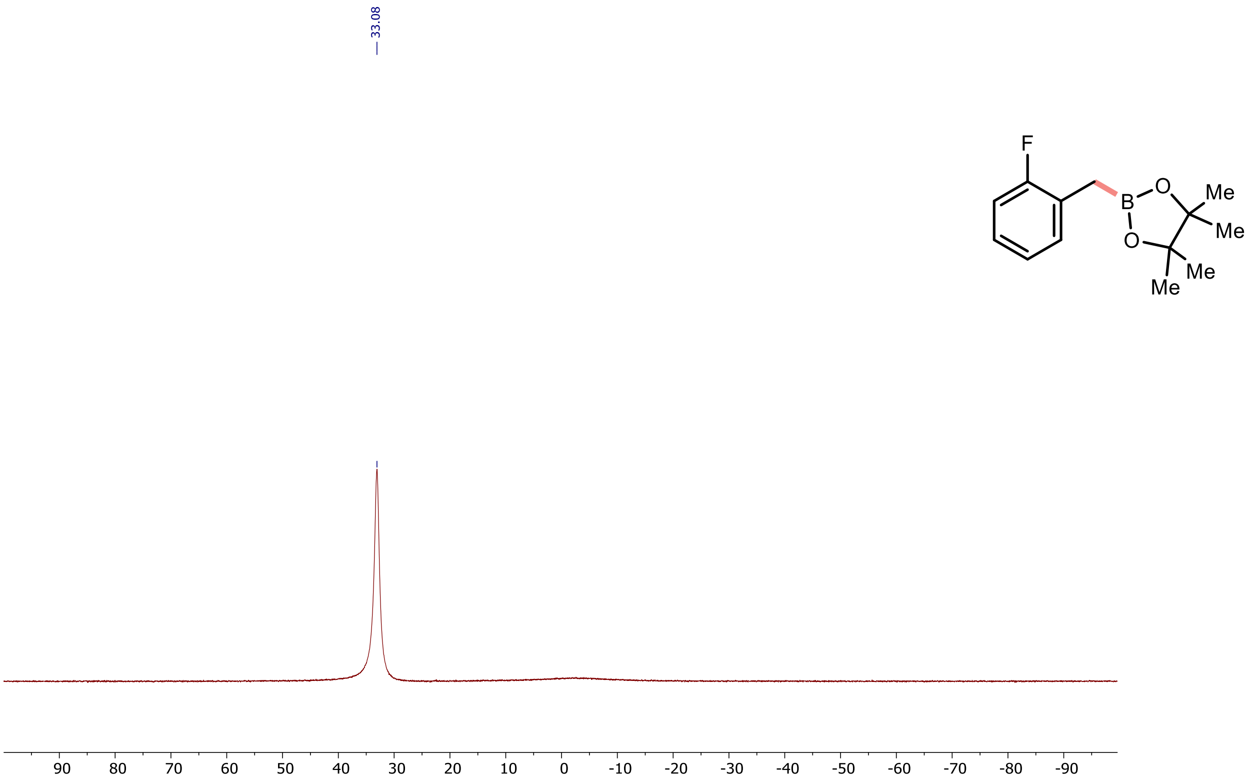

Supplementary Figure 16b-1 ^{1}H NMR (500 MHz, $CDCl_3$) 2-(4-Chlorobenzyl)-4,4,5,5-tetramethyl-1,3,2-dioxaborolane

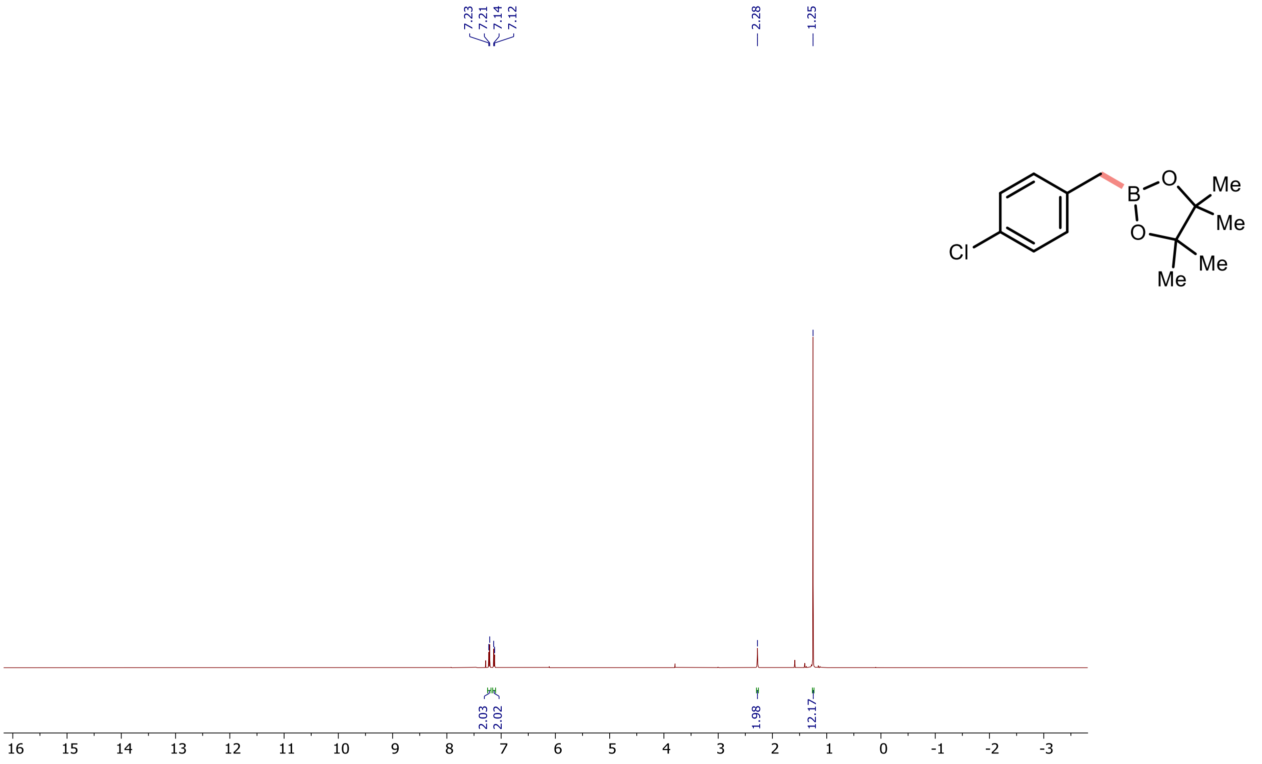

Supplementary Figure 16b-2 ^{13}C NMR (125 MHz, $CDCl_3$) 2-(4-Chlorobenzyl)-4,4,5,5-tetramethyl-1,3,2-dioxaborolane

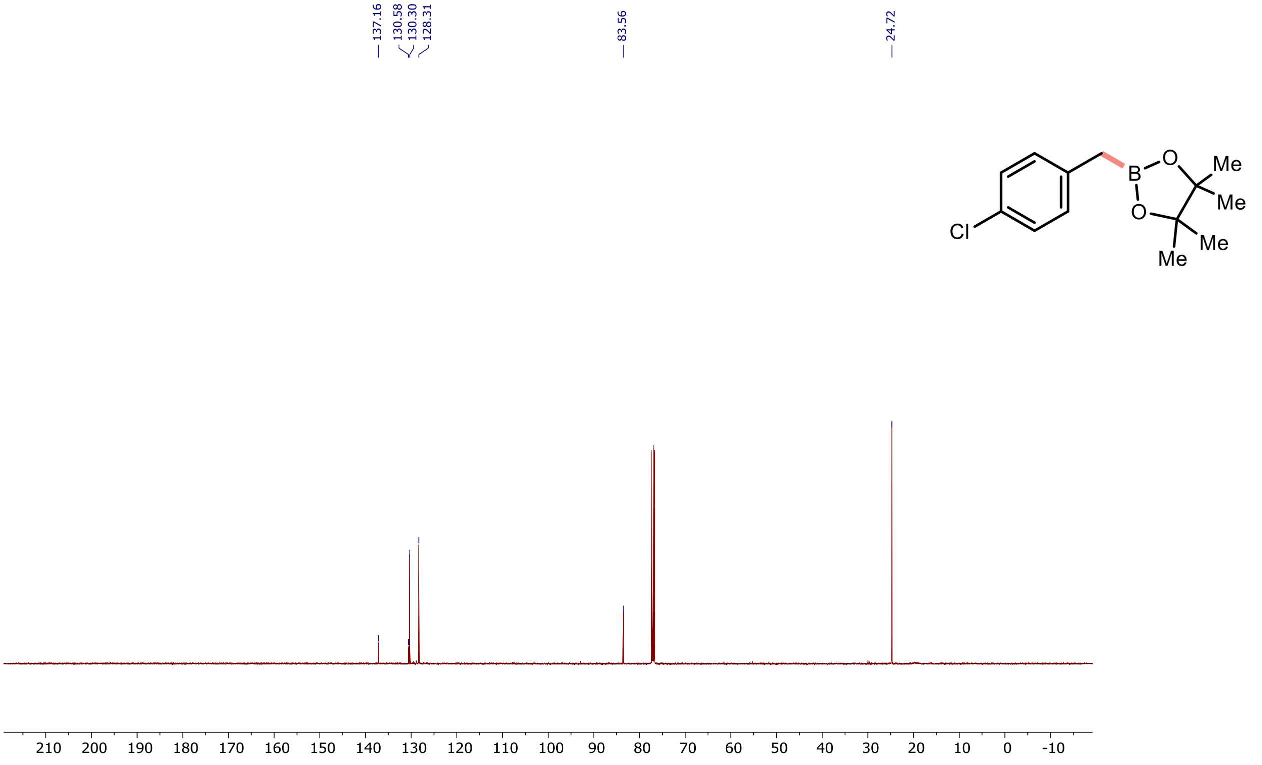

Supplementary Figure 16b-3 ^{11}B NMR (161 MHz, $CDCl_3$) 2-(4-Chlorobenzyl)-4,4,5,5-tetramethyl-1,3,2-dioxaborolane

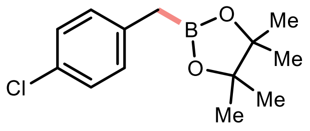

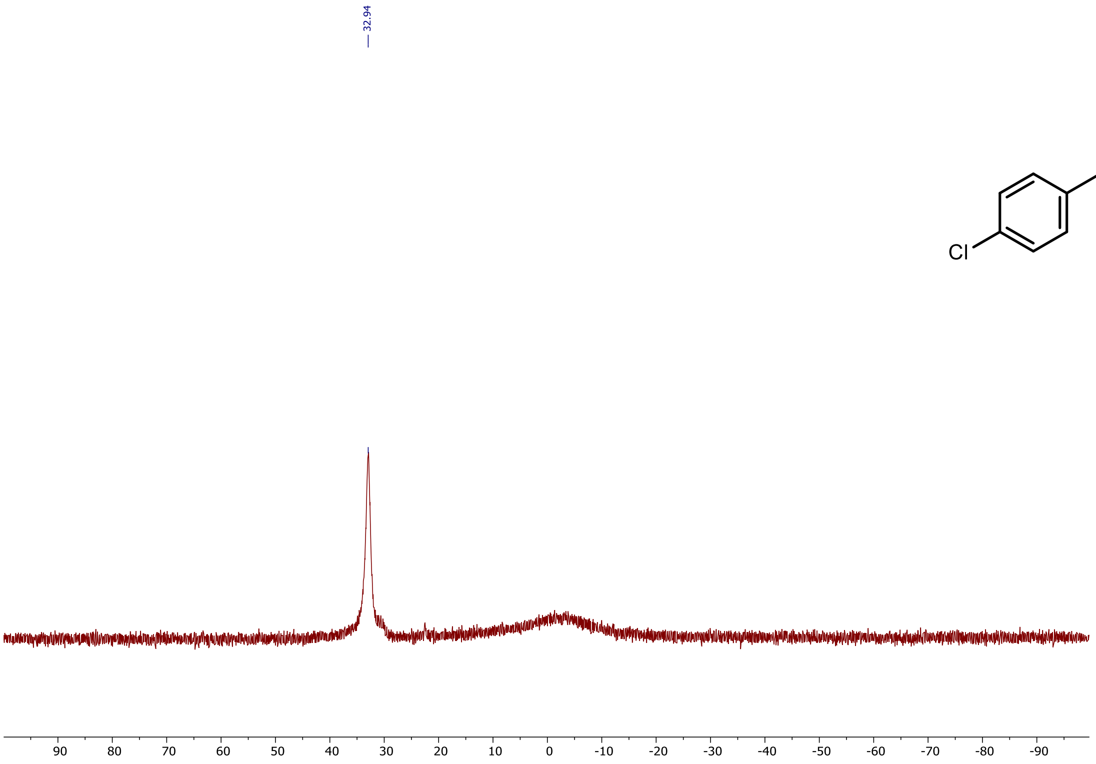

Supplementary Figure 17b-1 ^{1}H NMR (500 MHz, $CDCl_3$) 2-(3-Chlorobenzyl)-4,4,5,5-tetramethyl-1,3,2-dioxaborolane

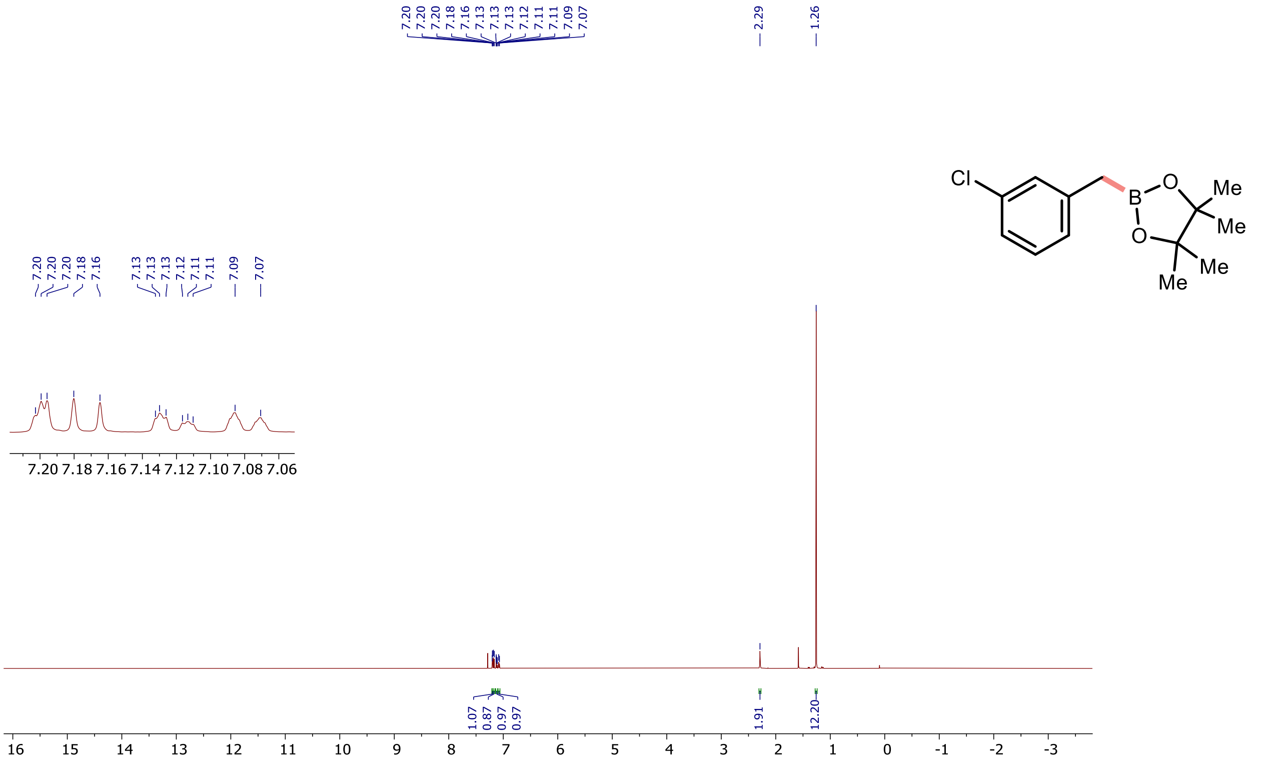

Supplementary Figure 17b-2 ^{13}C NMR (125 MHz, $CDCl_3$) 2-(3-Chlorobenzyl)-4,4,5,5-tetramethyl-1,3,2-dioxaborolane

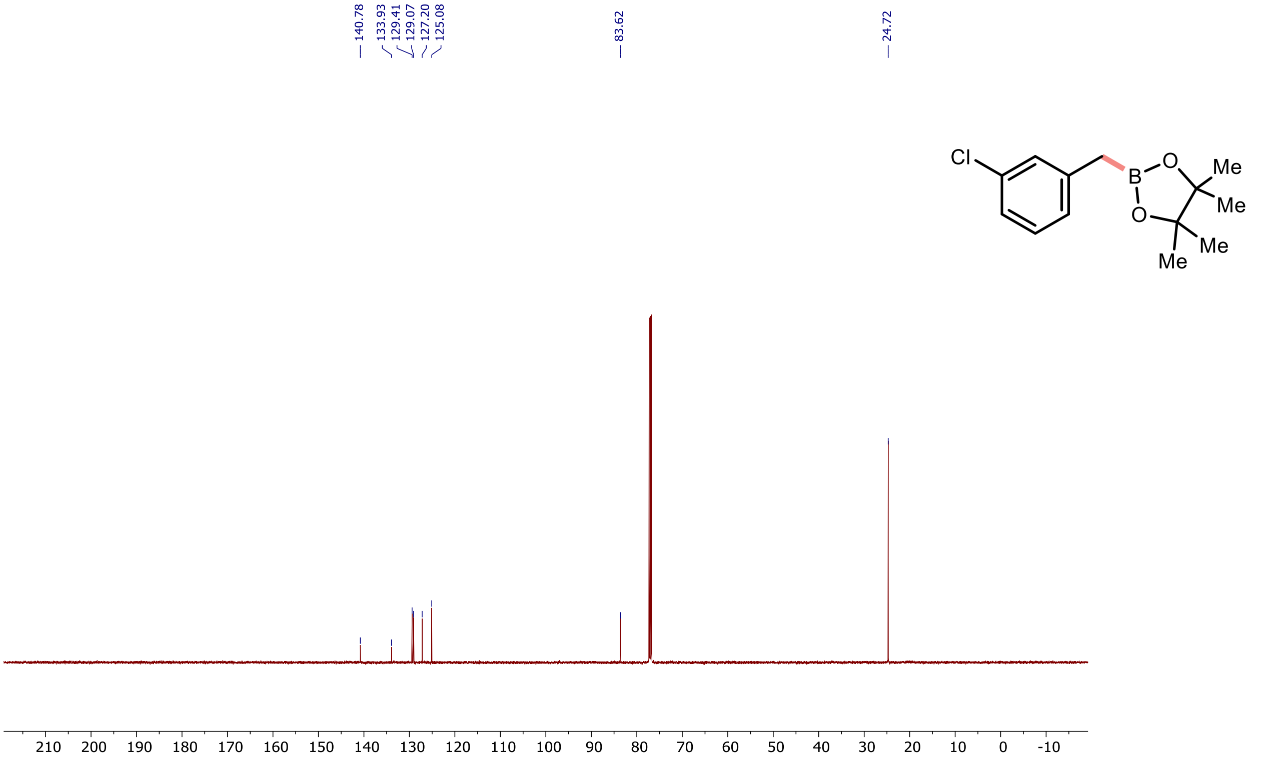

Supplementary Figure 17b-3 ^{11}B NMR (161 MHz, $CDCl_3$) 2-(3-Chlorobenzyl)-4,4,5,5-tetramethyl-1,3,2-dioxaborolane

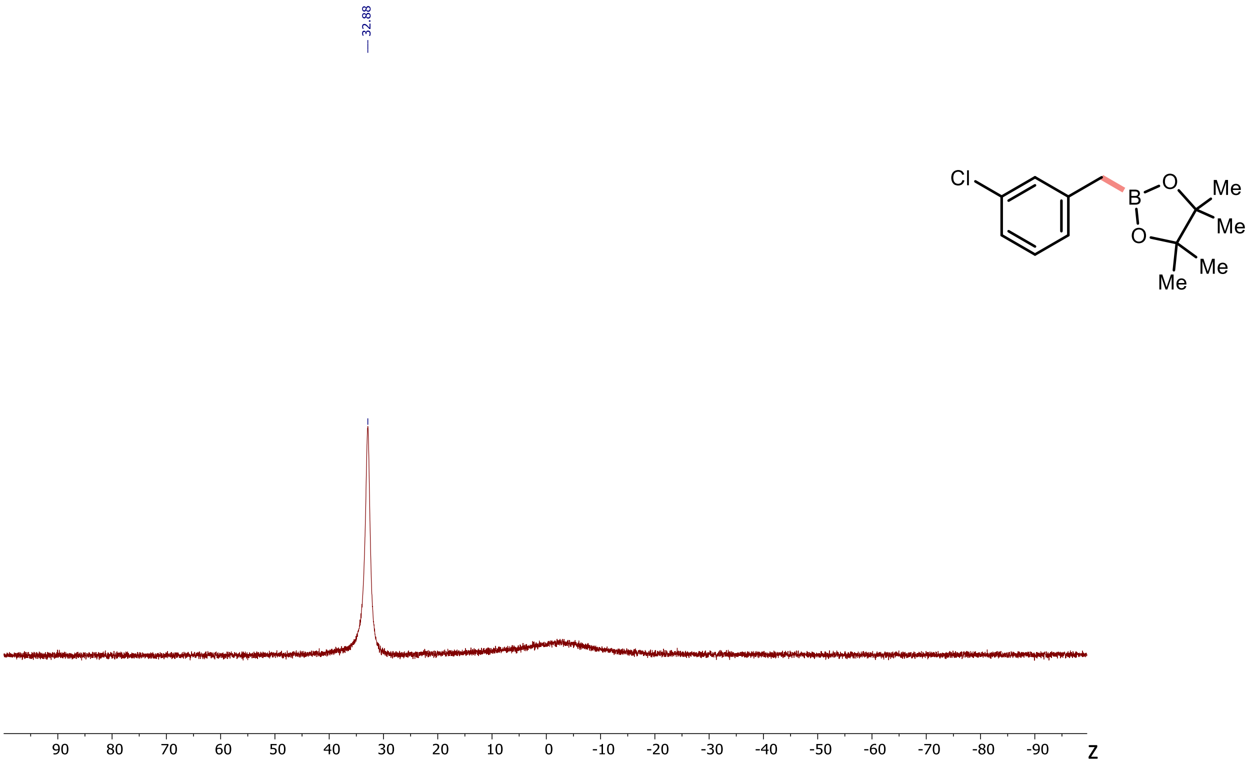

Supplementary Figure 18b-1 ^{1}H NMR (500 MHz, $CDCl_3$) 2-(4-Bromobenzyl)-4,4,5,5-tetramethyl-1,3,2-dioxaborolane

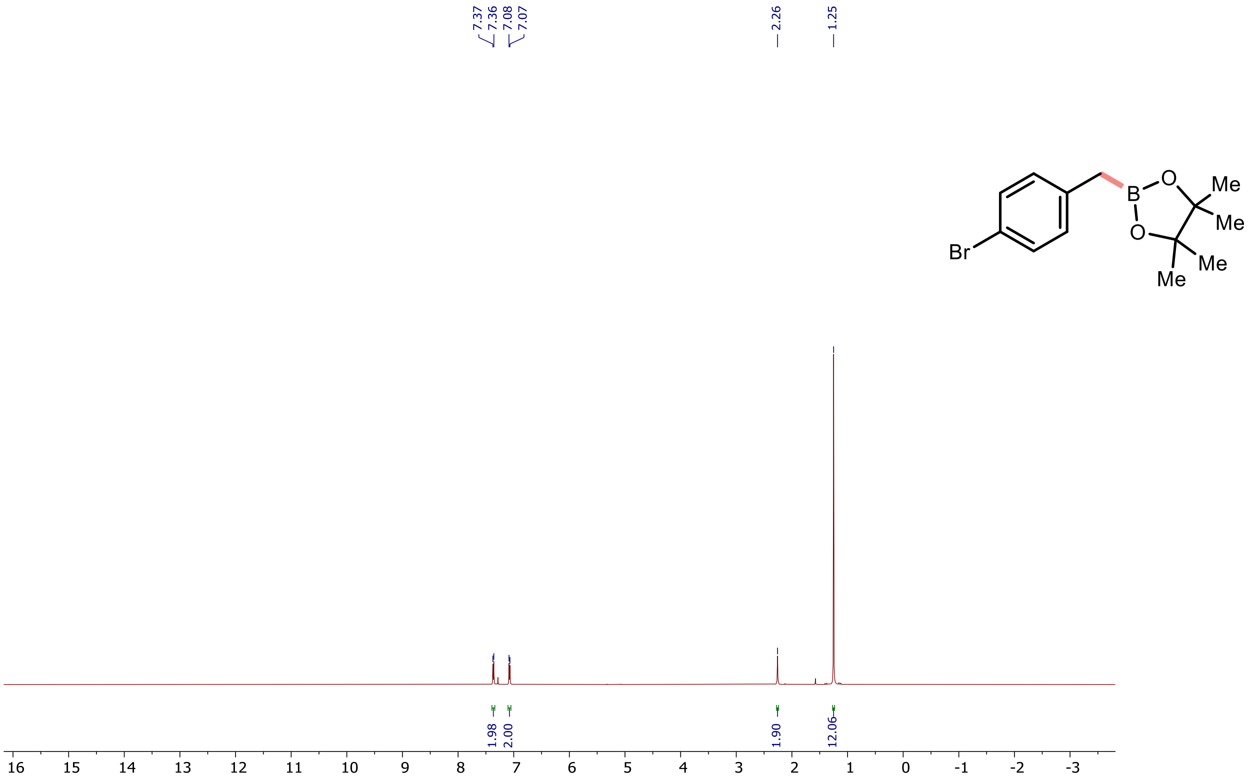

Supplementary Figure 18b-2 ^{13}C NMR (125 MHz, $CDCl_3$) 2-(4-Bromobenzyl)-4,4,5,5-tetramethyl-1,3,2-dioxaborolane

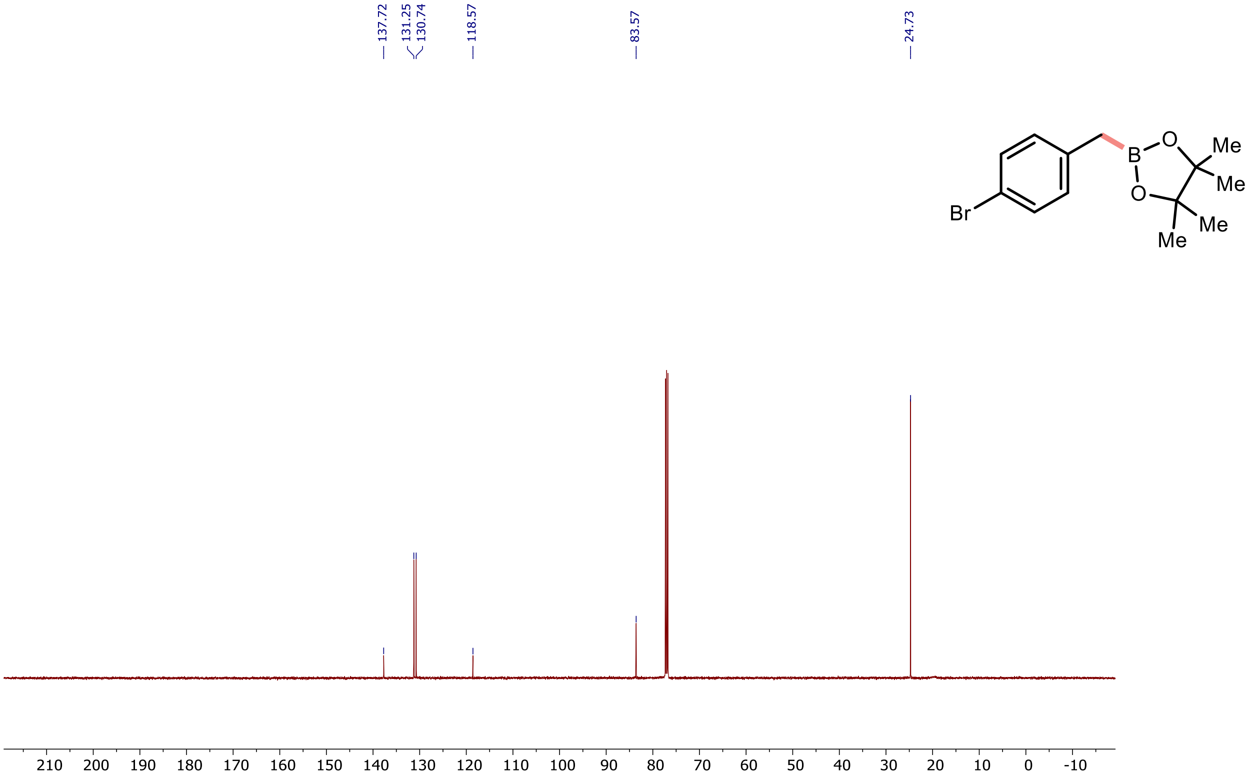

Supplementary Figure 18b-3 ^{11}B NMR (161 MHz, $CDCl_3$) 2-(4-Bromobenzyl)-4,4,5,5-tetramethyl-1,3,2-dioxaborolane

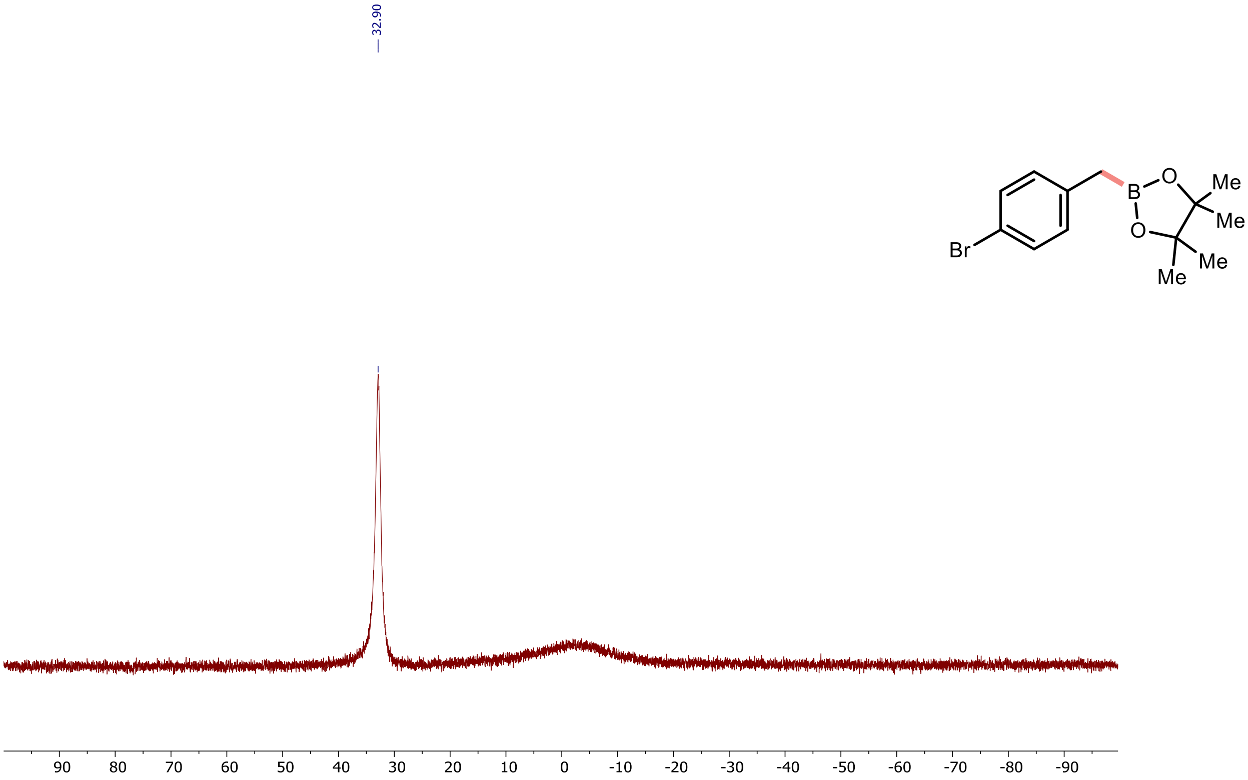

Supplementary Figure 19b-1 ^{1}H NMR (500 MHz, $CDCl_3$) 2-(3-Iodobenzyl)-4,4,5,5-tetramethyl-1,3,2-dioxaborolane

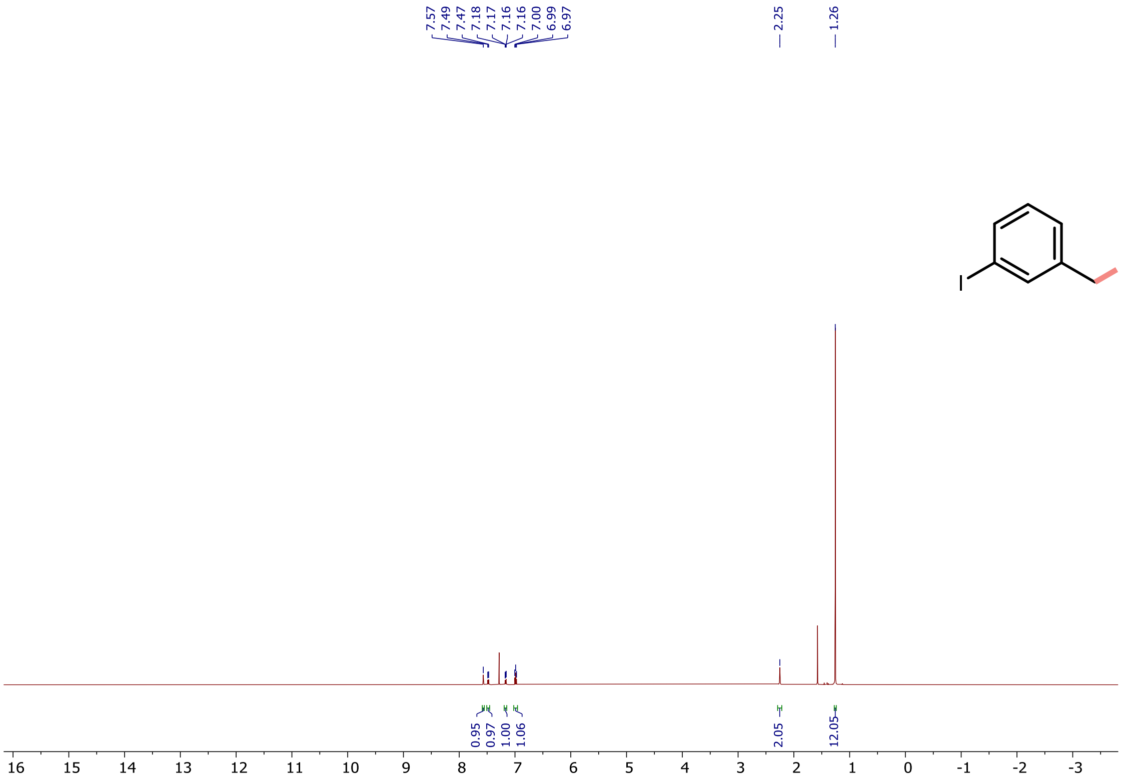

Supplementary Figure 19b-2 ^{13}C NMR (125 MHz, $CDCl_3$) 2-(3-Iodobenzyl)-4,4,5,5-tetramethyl-1,3,2-dioxaborolane

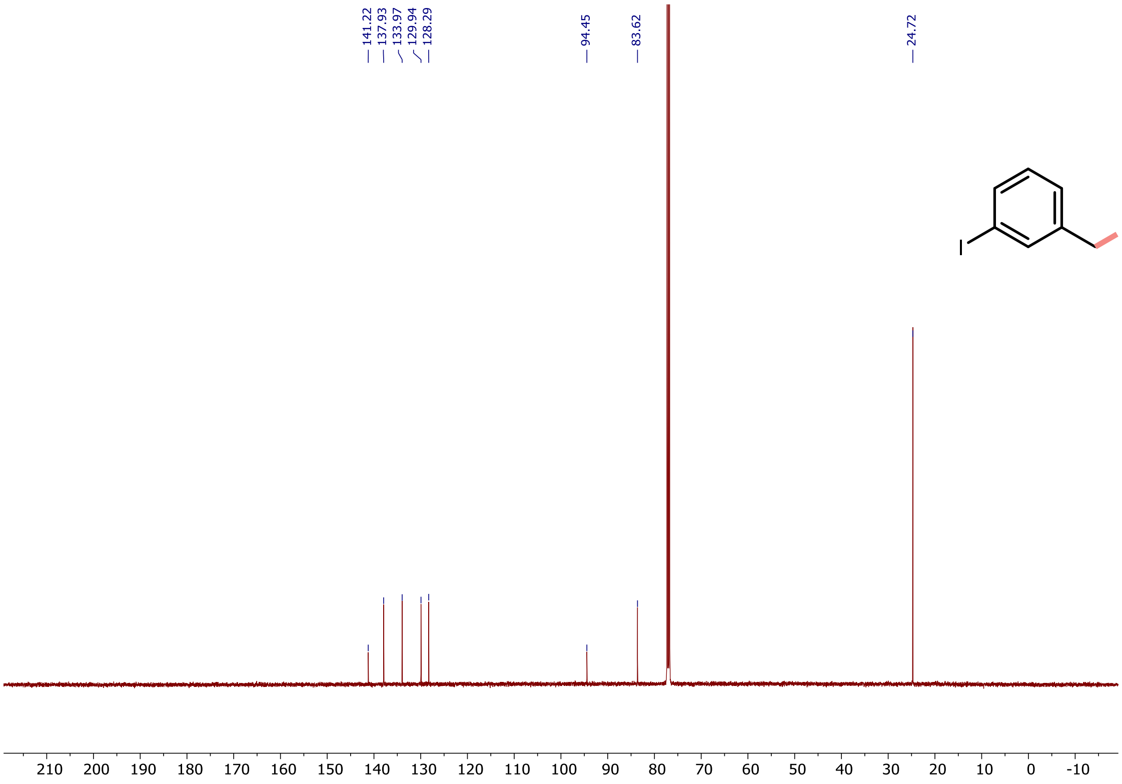

Supplementary Figure 19b-3 ^{11}B NMR (161 MHz, $CDCl_3$) 2-(3-Iodobenzyl)-4,4,5,5-tetramethyl-1,3,2-dioxaborolane

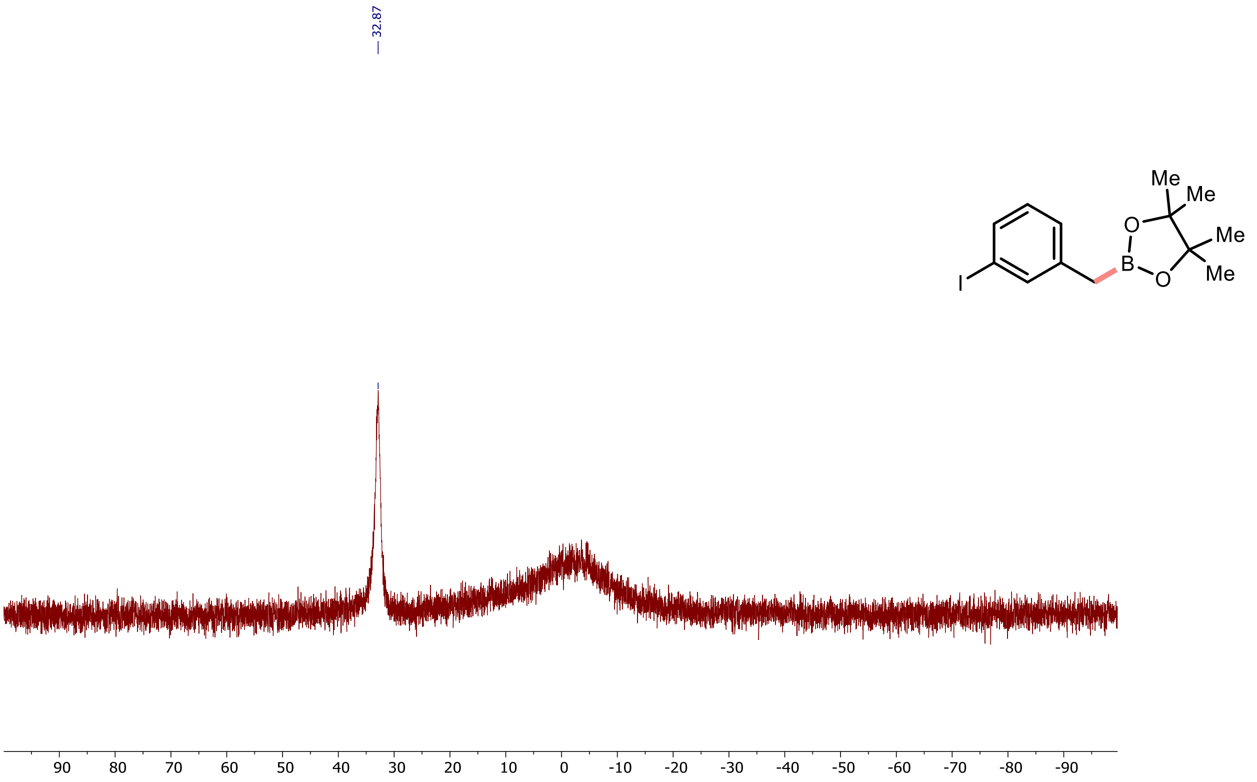

Supplementary Figure 20b-1 ^{1}H NMR (500 MHz, $CDCl_3$) 4-((4,4,5,5-Tetramethyl-1,3,2-dioxaborolan-2-yl)methyl)phenyl acetate

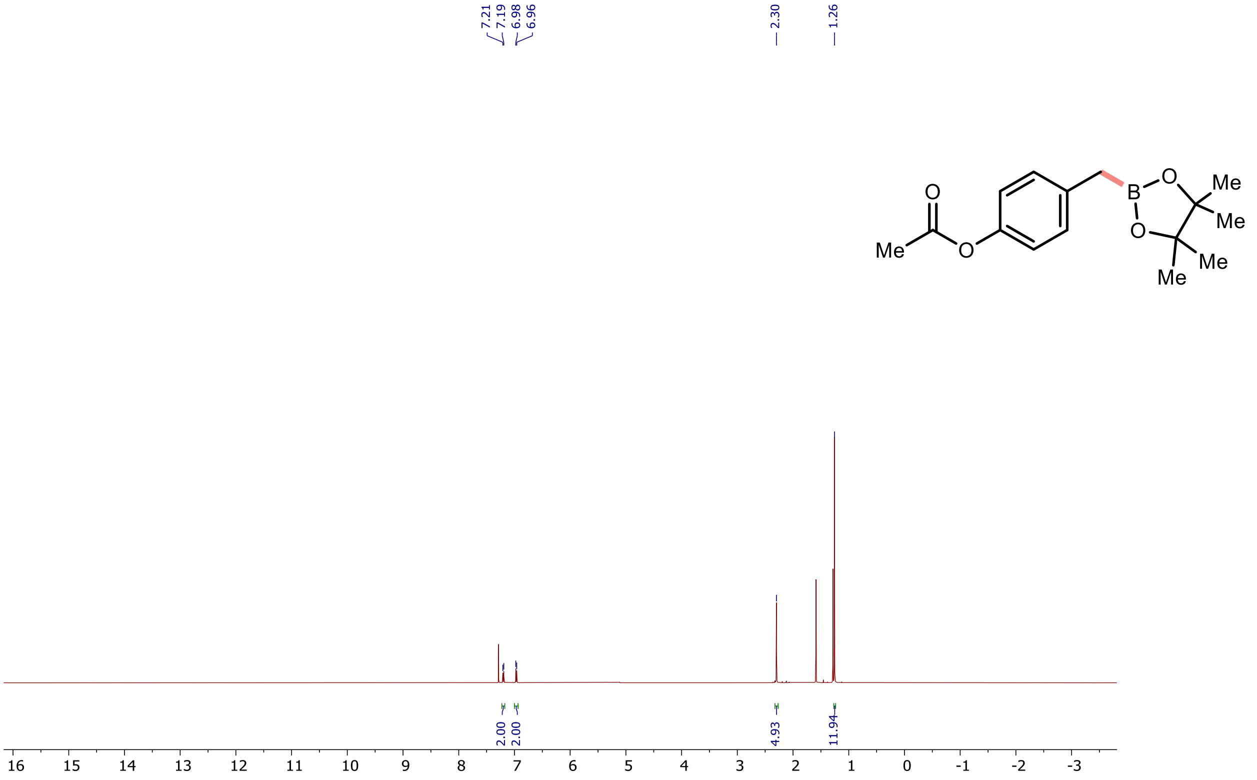

Supplementary Figure 20b-2 ^{13}C NMR (125 MHz, $CDCl_3$) 4-((4,4,5,5-Tetramethyl-1,3,2-dioxaborolan-2-yl)methyl)phenyl acetate

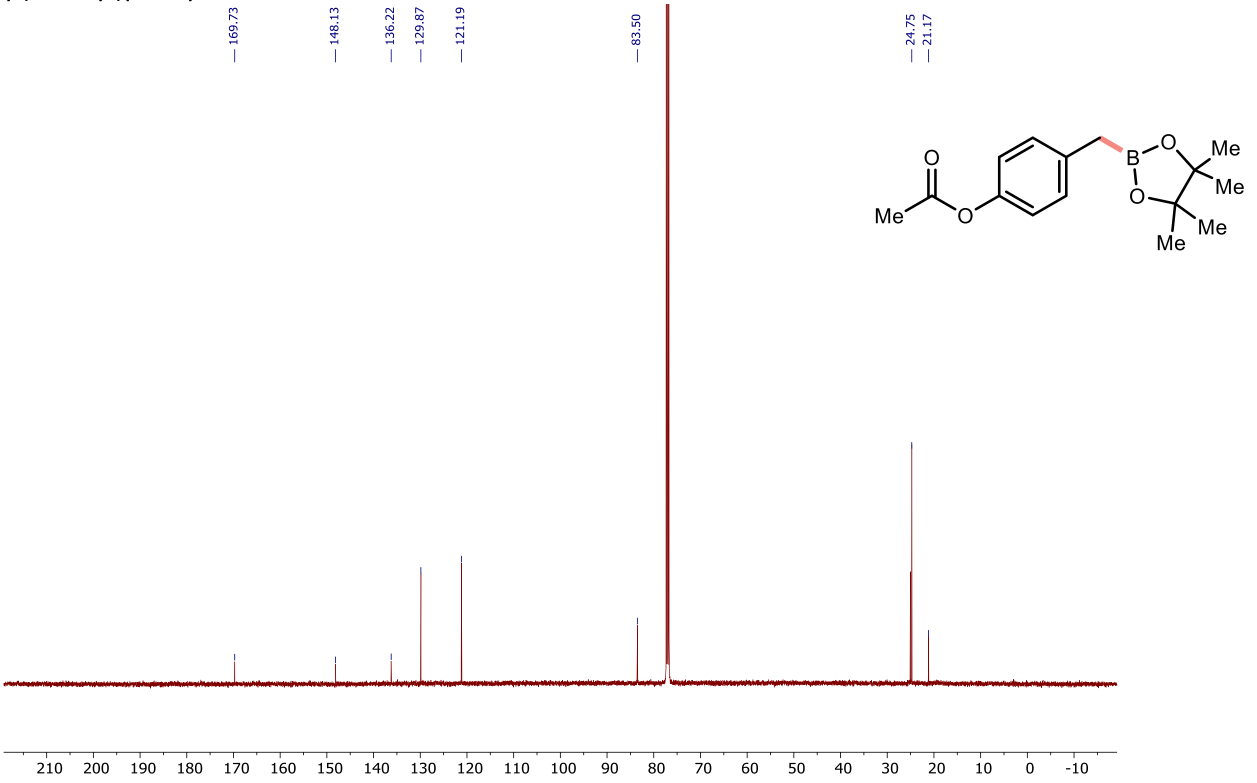

Supplementary Figure 20b-3 ^{11}B NMR (161 MHz, $CDCl_3$) 4-((4,4,5,5-Tetramethyl-1,3,2-dioxaborolan-2-yl)methyl)phenyl acetate

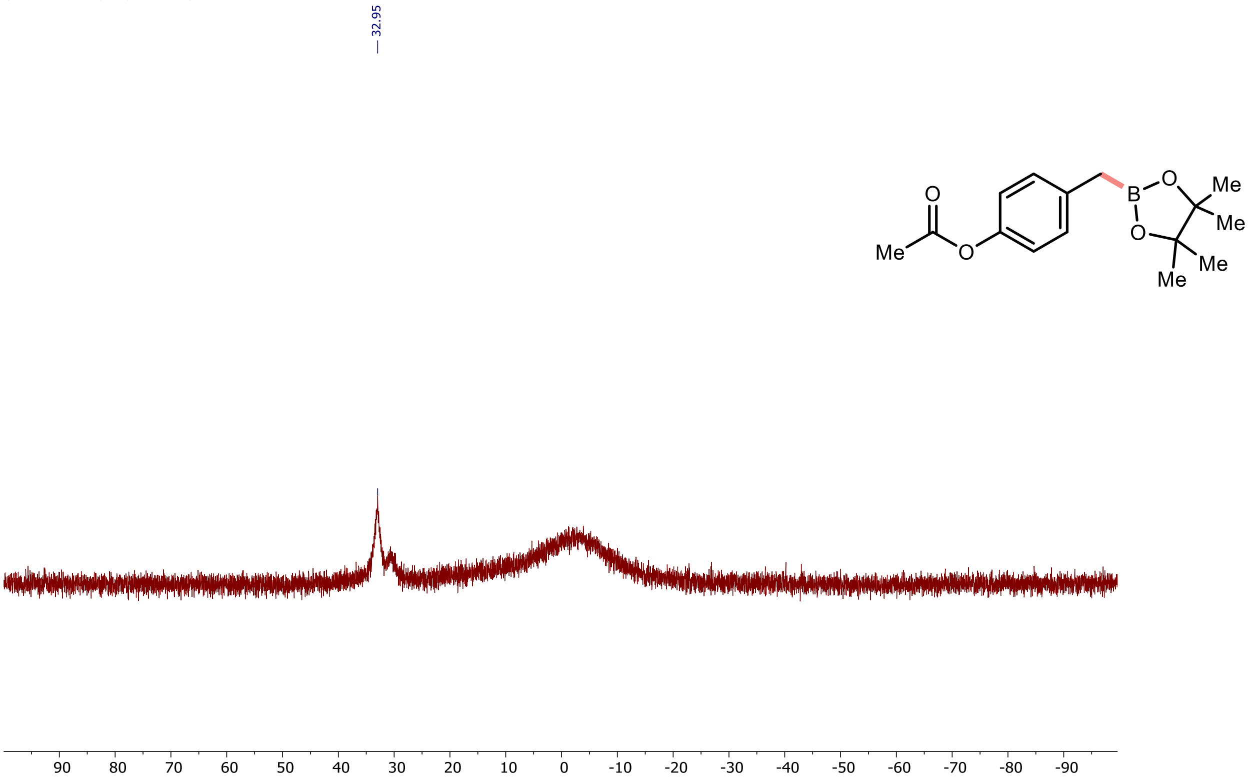

Supplementary Figure 21b-1 ^{1}H NMR (500 MHz, $CDCl_3$) 4,4,5,5-Tetramethyl-2-(4-(4,4,5,5-tetramethyl-1,3,2-dioxaborolan-2-yl)benzyl)-1,3,2-dioxaborolane

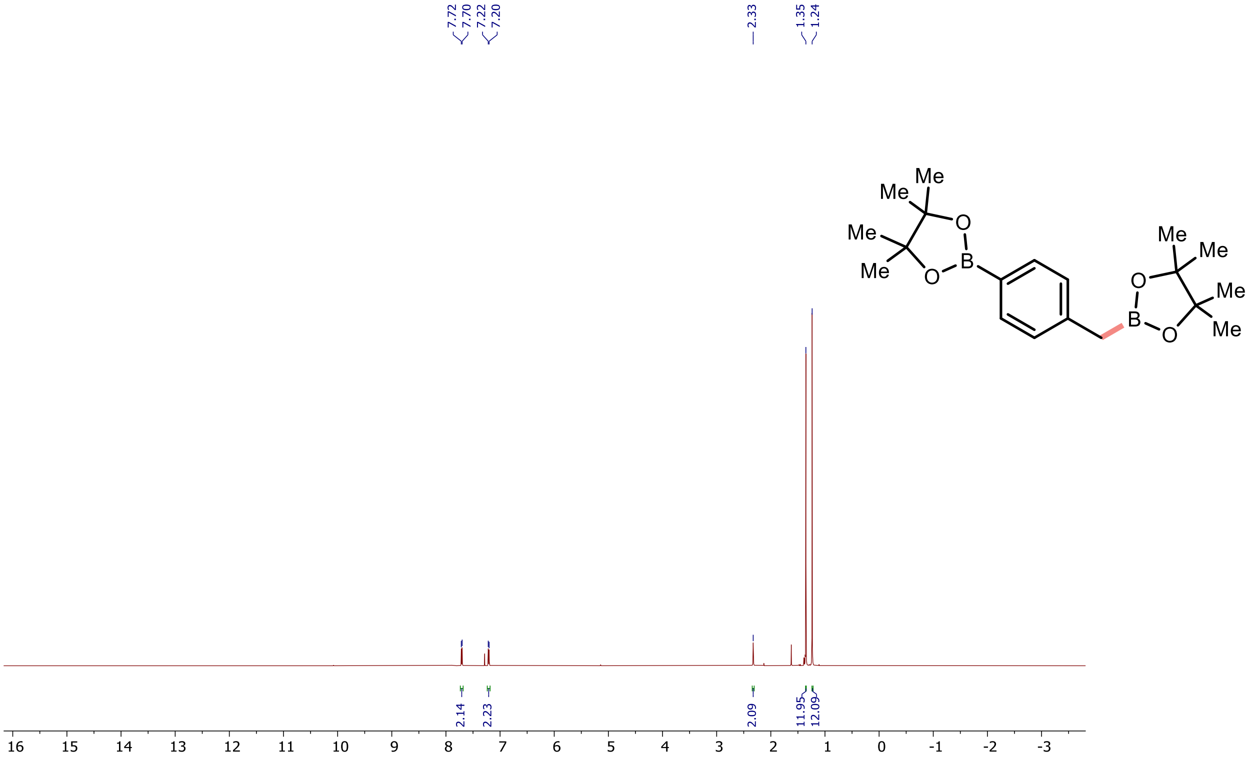

Supplementary Figure 21b-2 ^{13}C NMR (125 MHz, $CDCl_3$) 4,4,5,5-Tetramethyl-2-(4-(4,4,5,5-tetramethyl-1,3,2-dioxaborolan-2-yl)benzyl)-1,3,2-dioxaborolane

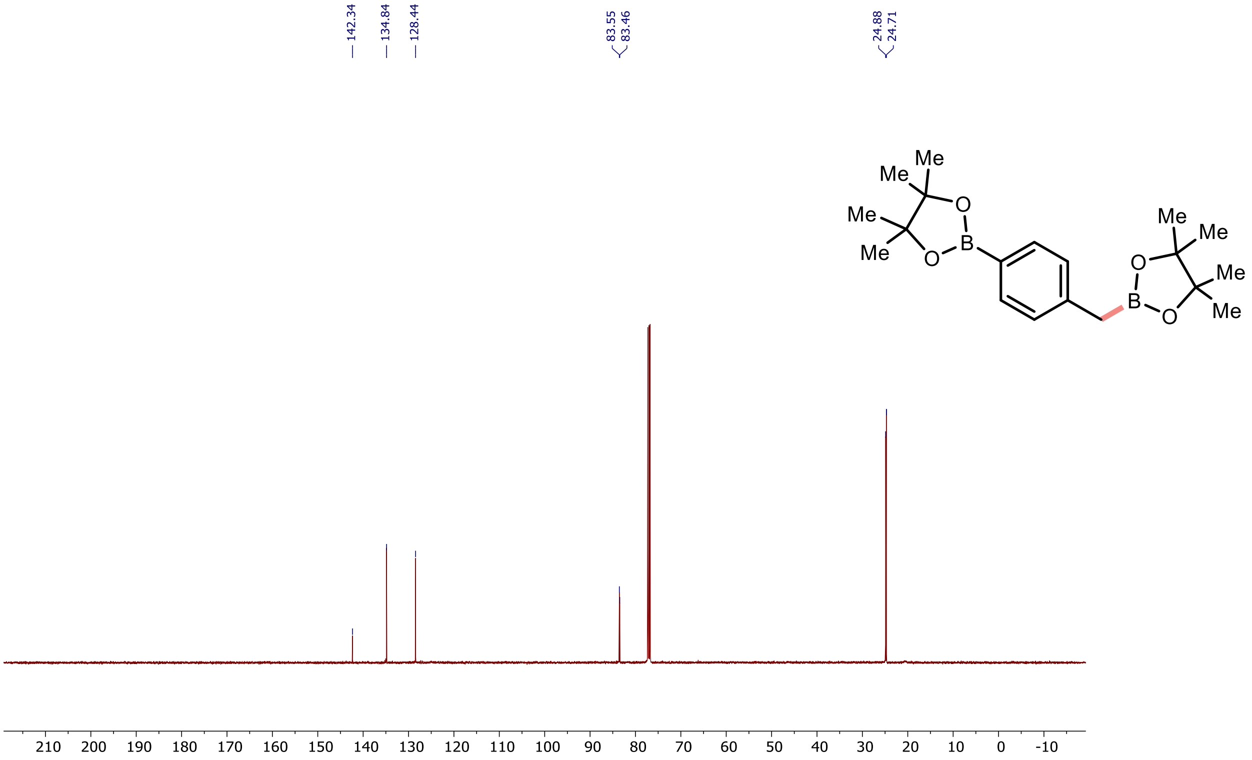

Supplementary Figure 21b-3 ^{11}B NMR (161 MHz, $CDCl_3$) 4,4,5,5-Tetramethyl-2-(4-(4,4,5,5-tetramethyl-1,3,2-dioxaborolan-2-yl)benzyl)-1,3,2-dioxaborolane

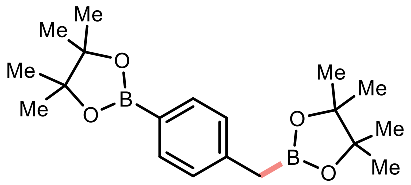

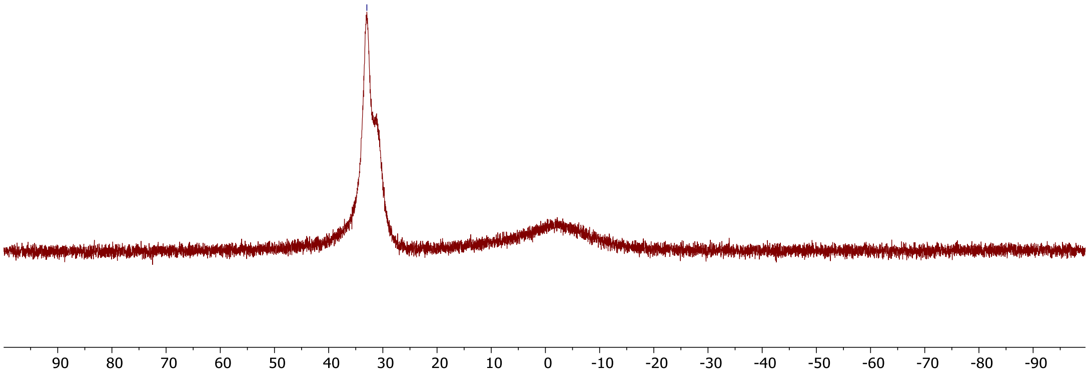

Supplementary Figure 22b-1 ^{1}H NMR (500 MHz, $CDCl_3$) 2-(4-Methoxybenzyl)-4,4,5,5-tetramethyl-1,3,2-dioxaborolane

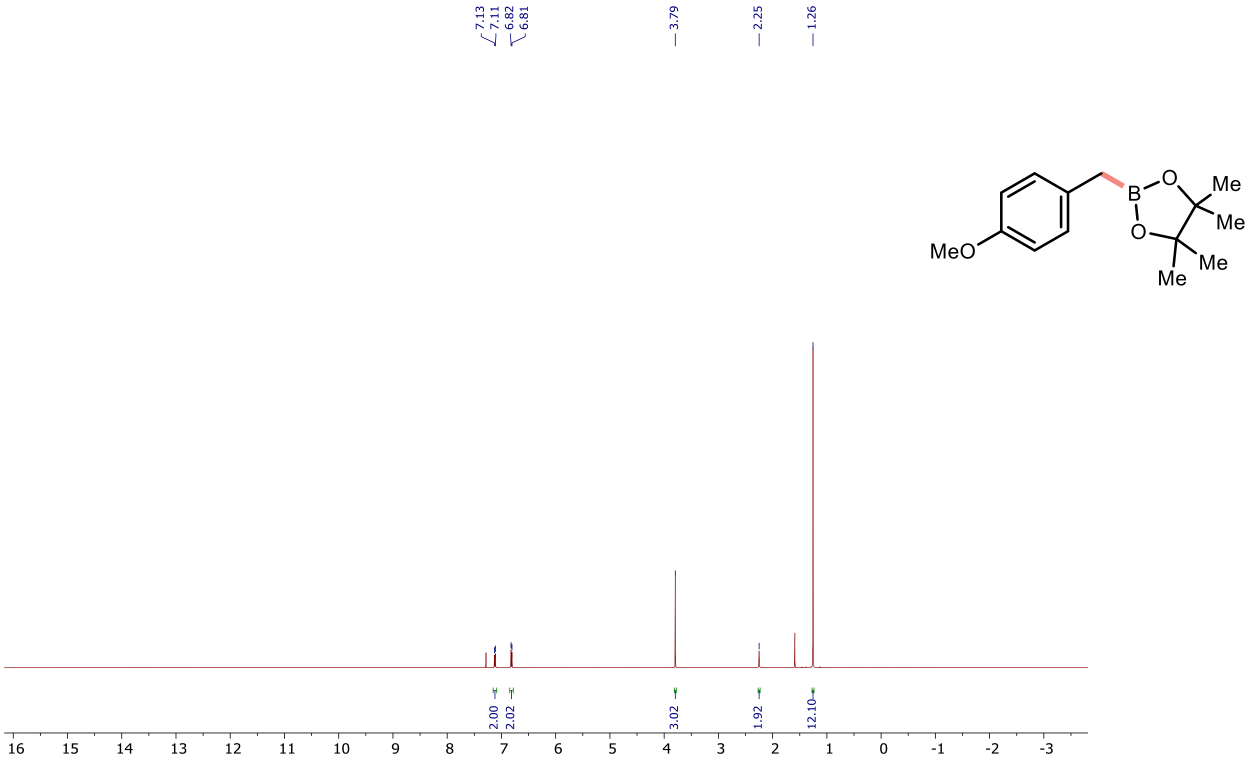

Supplementary Figure 22b-2^{13}C NMR (125 MHz, $CDCl_3$) 2-(4-Methoxybenzyl)-4,4,5,5-tetramethyl-1,3,2-dioxaborolane

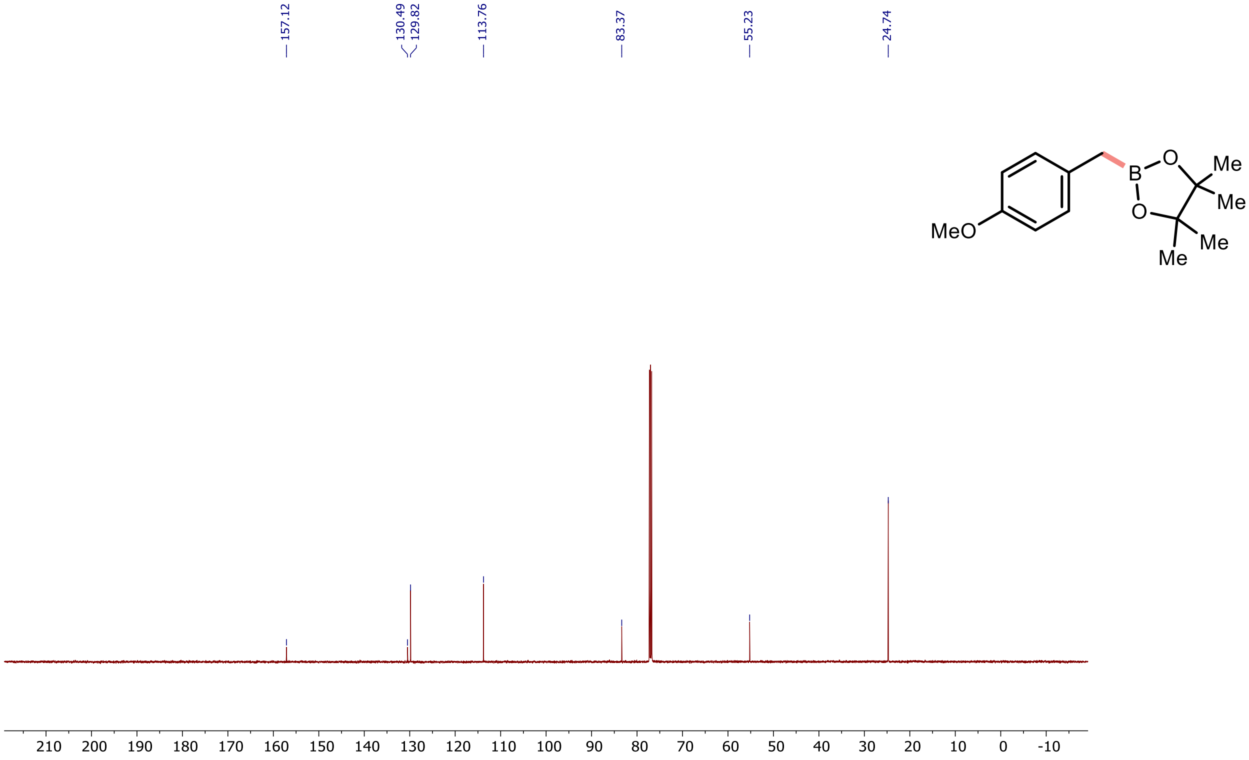

Supplementary Figure 22b-3 ^{11}B NMR (161 MHz, $CDCl_3$) 2-(4-Methoxybenzyl)-4,4,5,5-tetramethyl-1,3,2-dioxaborolane

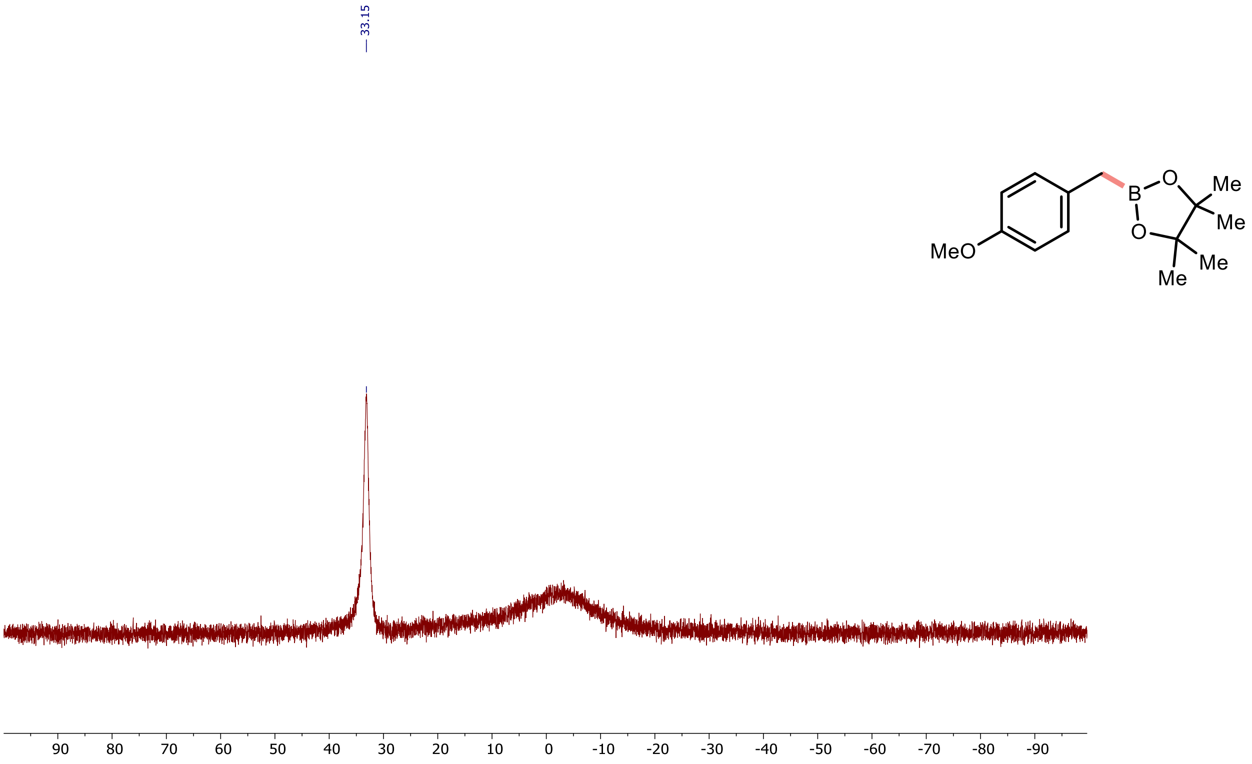

Supplementary Figure 23b-1 ^{1}H NMR (500 MHz, $CDCl_3$) 2-(3-Methoxybenzyl)-4,4,5,5-tetramethyl-1,3,2-dioxaborolane

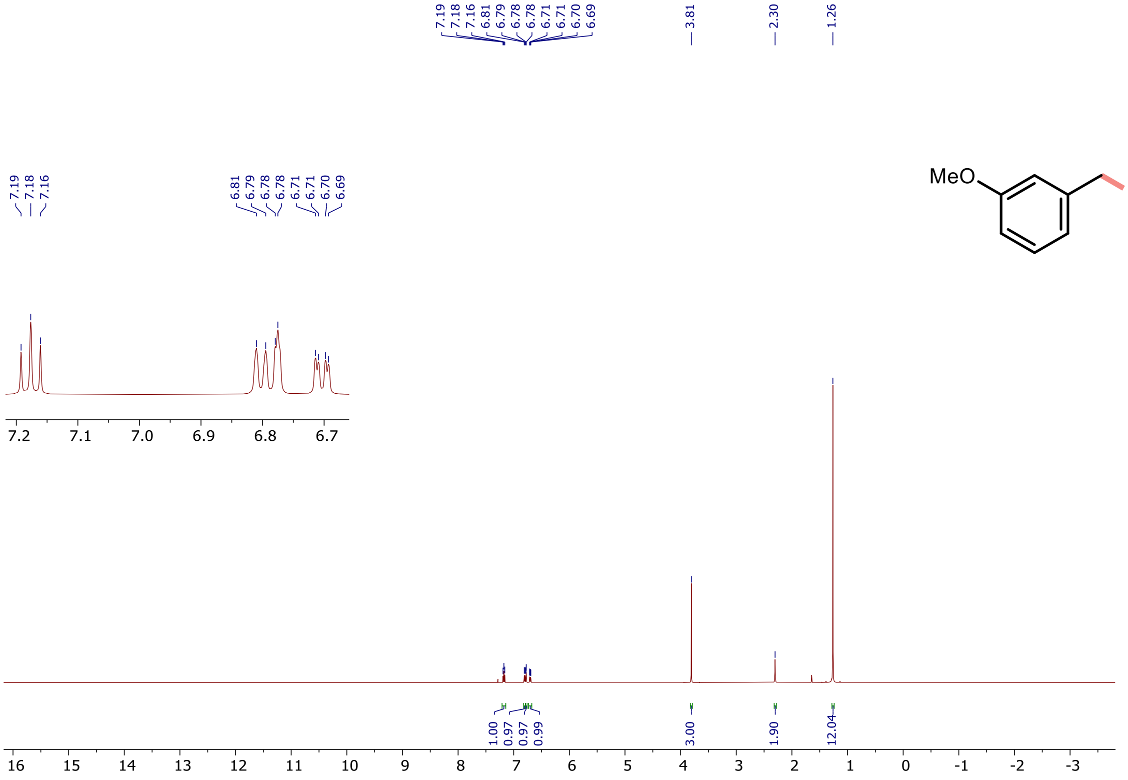

Supplementary Figure 23b-2 ^{13}C NMR (125 MHz, $CDCl_3$) 2-(3-Methoxybenzyl)-4,4,5,5-tetramethyl-1,3,2-dioxaborolane

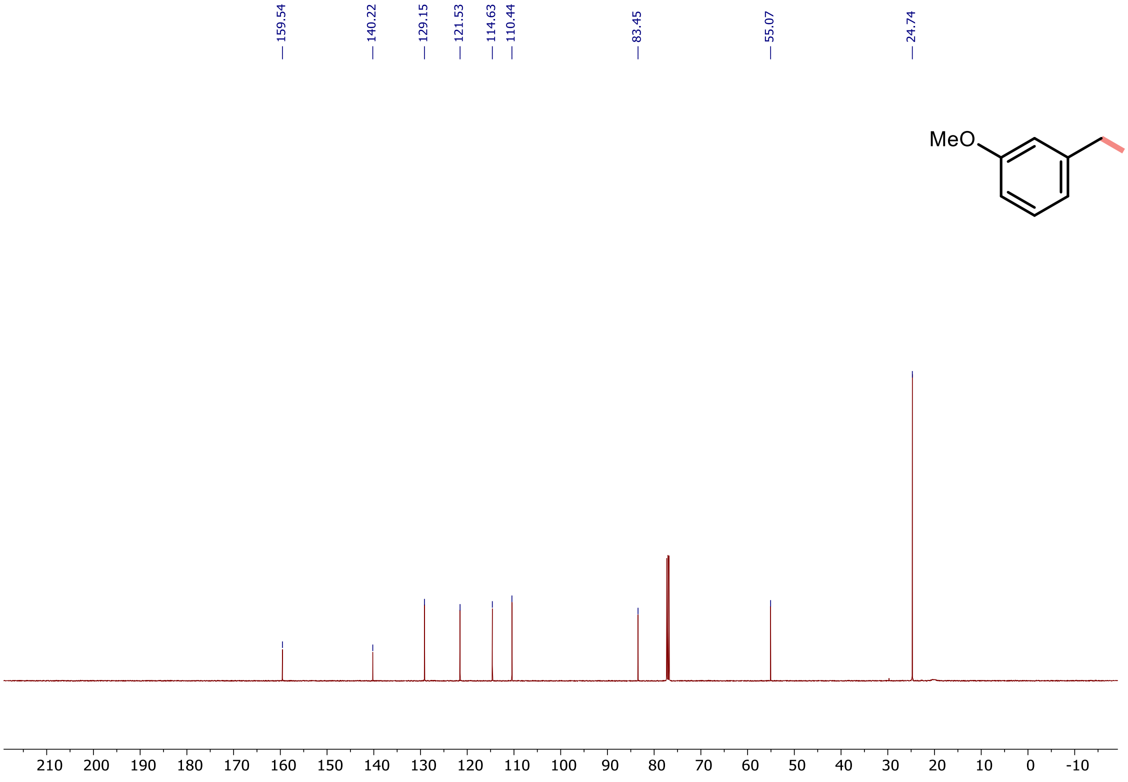

Supplementary Figure 23b-3 ^{11}B NMR (161 MHz, $CDCl_3$) 2-(3-Methoxybenzyl)-4,4,5,5-tetramethyl-1,3,2-dioxaborolane

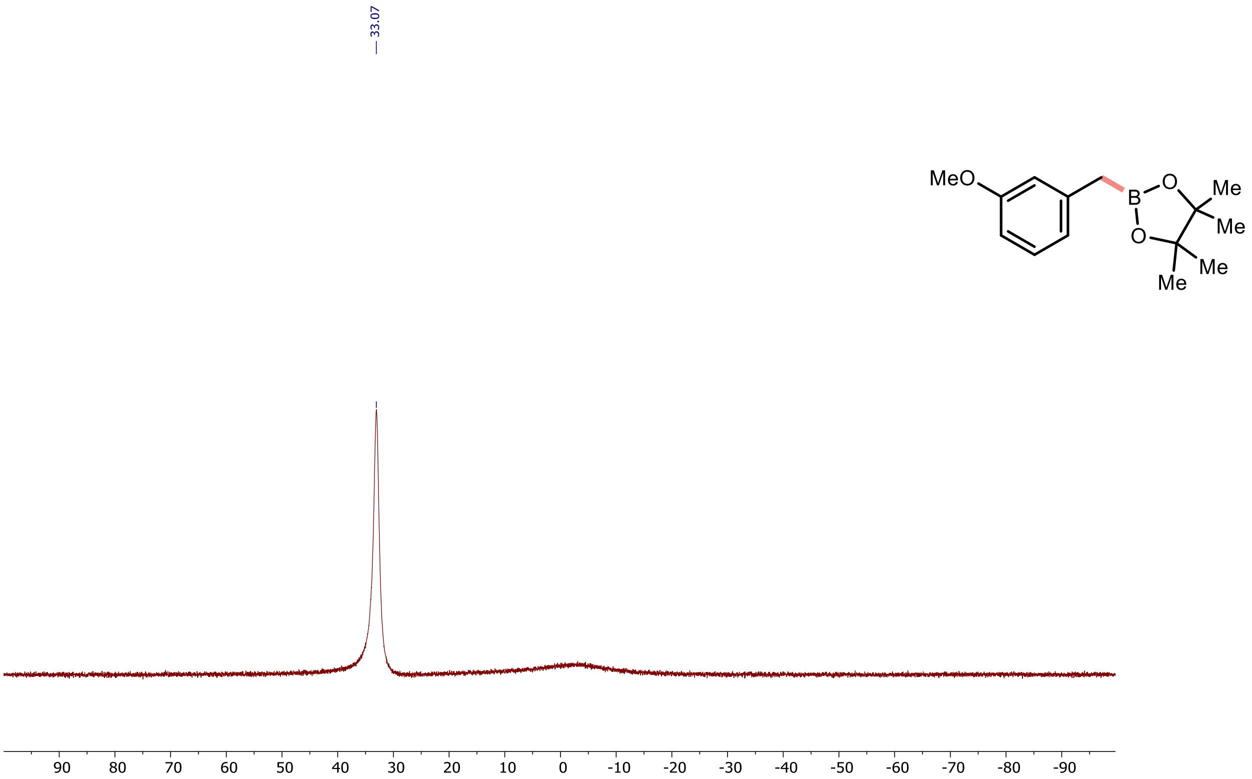

Supplementary Figure 24b-1 ^{1}H NMR (500 MHz, $CDCl_3$) 2-(4-(Benzyloxy)benzyl)-4,4,5,5-tetramethyl-1,3,2-dioxaborolane

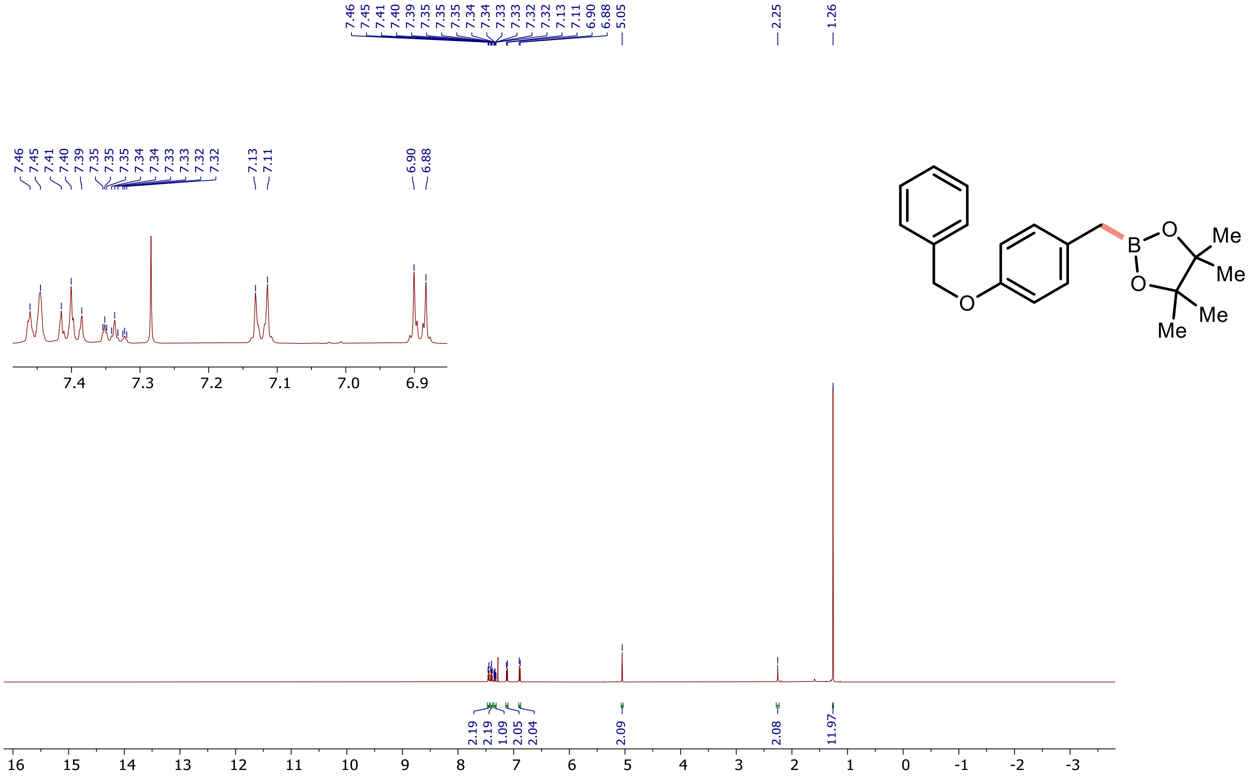

Supplementary Figure 24b-2 ^{13}C NMR (125 MHz, $CDCl_3$) 2-(4-(Benzyloxy)benzyl)-4,4,5,5-tetramethyl-1,3,2-dioxaborolane

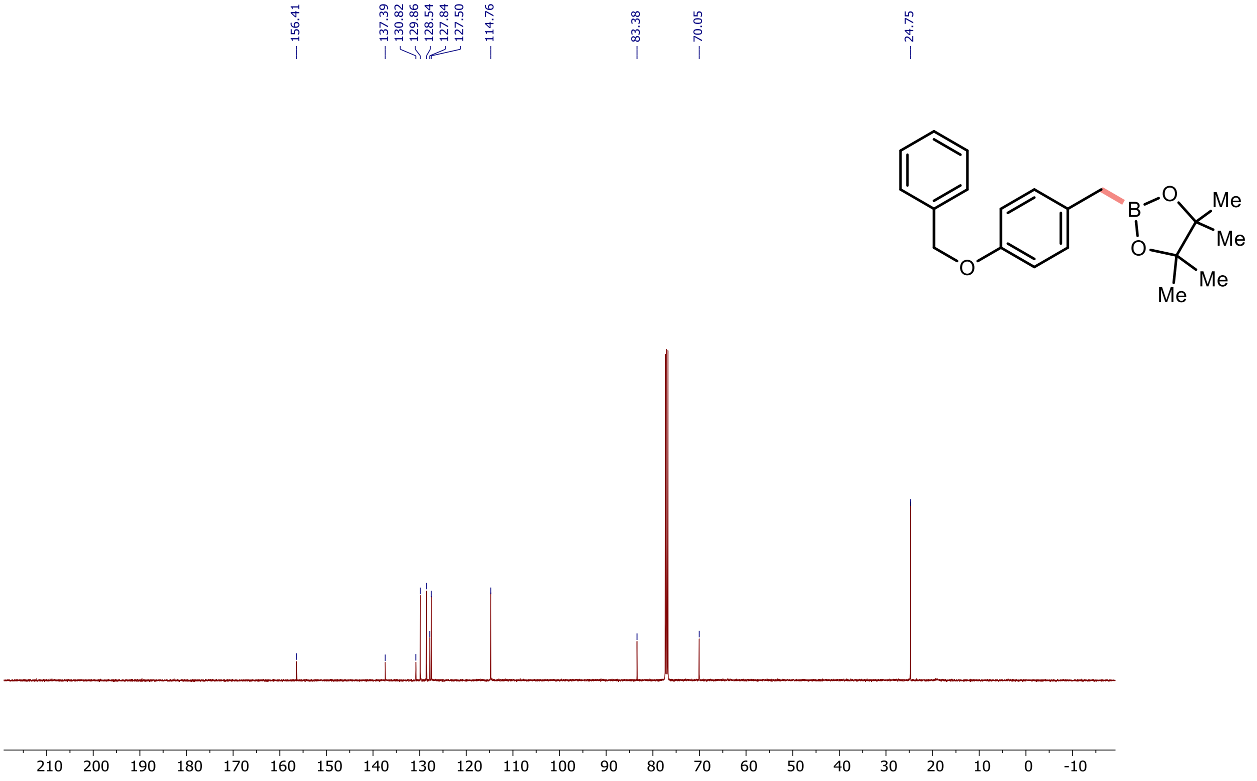

Supplementary Figure 24b-3 ^{11}B NMR (161 MHz, $CDCl_3$) 2-(4-(Benzyloxy)benzyl)-4,4,5,5-tetramethyl-1,3,2-dioxaborolane

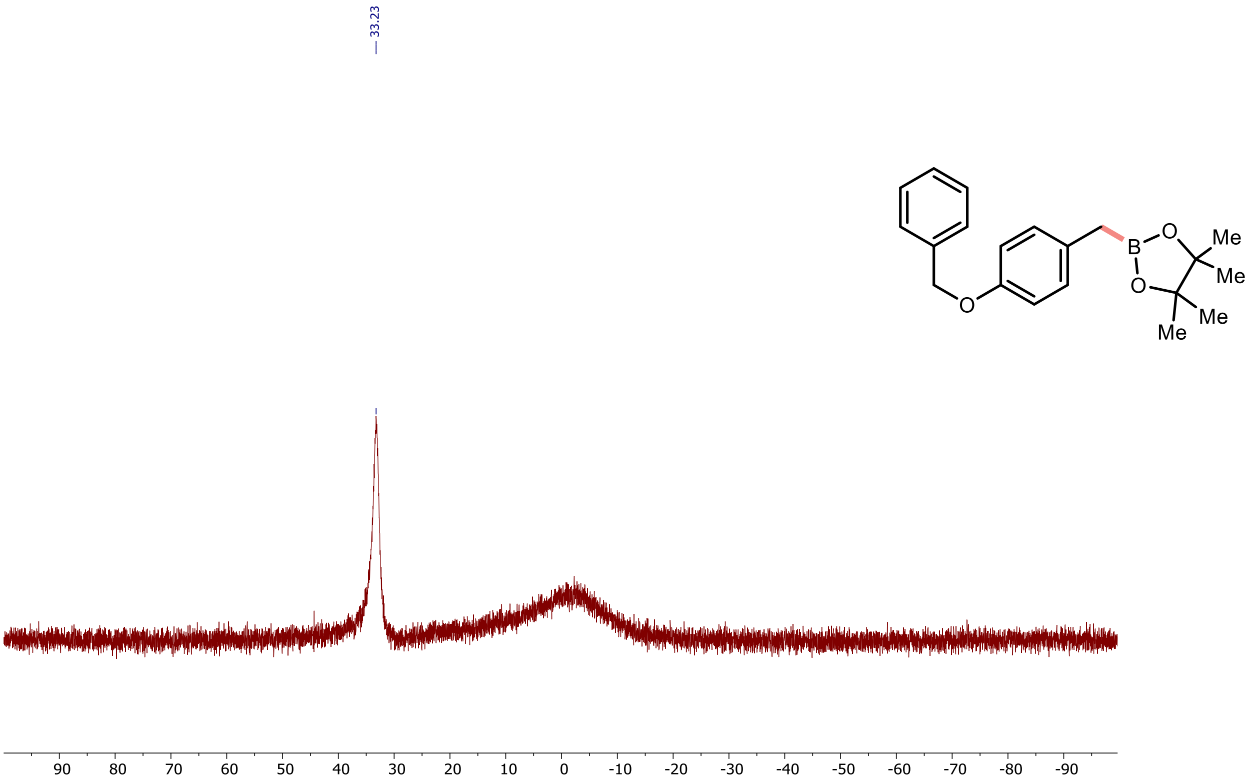

Supplementary Figure 25b-1 ^{1}H NMR (500 MHz, $CDCl_3$) 2-(4-(Dodecyloxy)benzyl)-4,4,5,5-tetramethyl-1,3,2-dioxaborolane

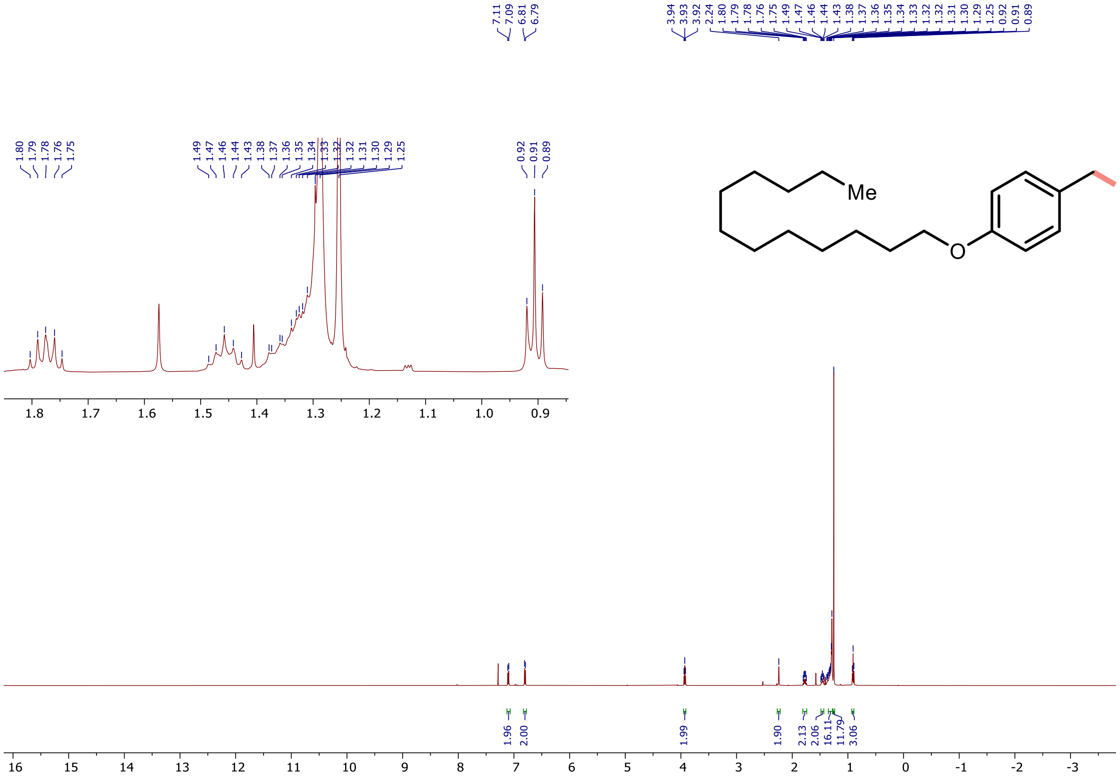

Supplementary Figure 25b-2 ^{13}C NMR (125 MHz, $CDCl_3$) 2-(4-(Dodecyloxy)benzyl)-4,4,5,5-tetramethyl-1,3,2-dioxaborolane

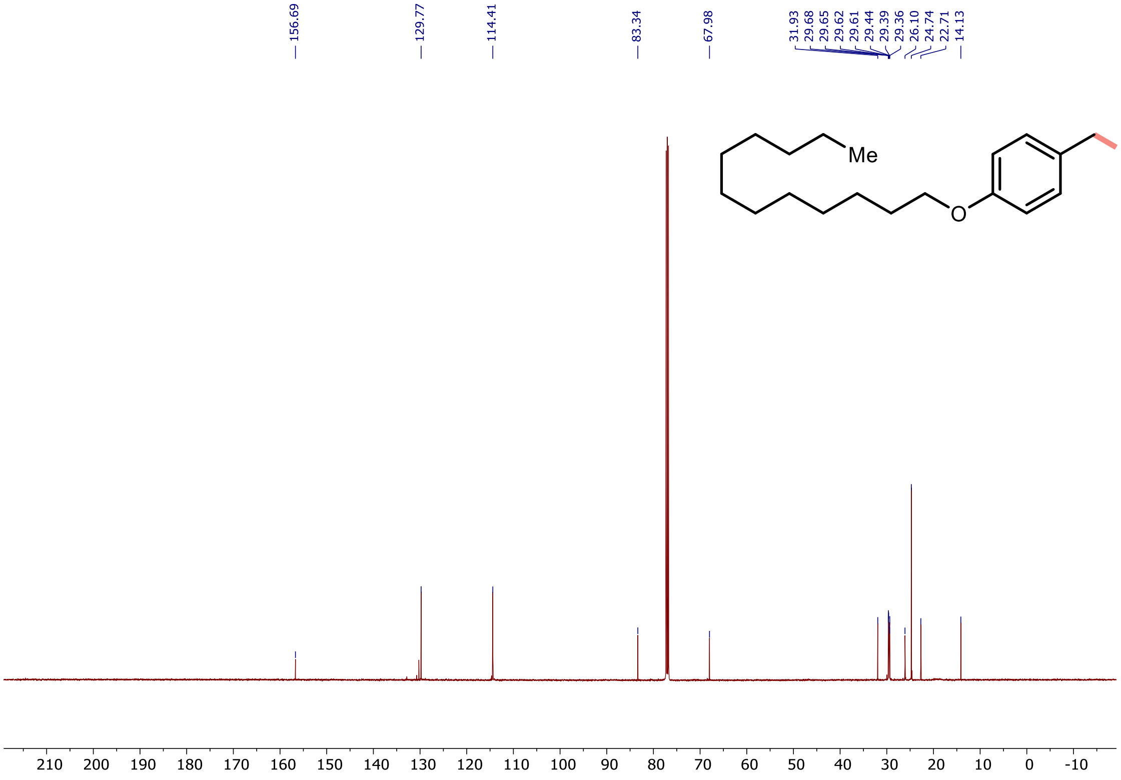

Supplementary Figure 25b-3 ^{11}B NMR (161 MHz, $CDCl_3$) 2-(4-(Dodecyloxy)benzyl)-4,4,5,5-tetramethyl-1,3,2-dioxaborolane

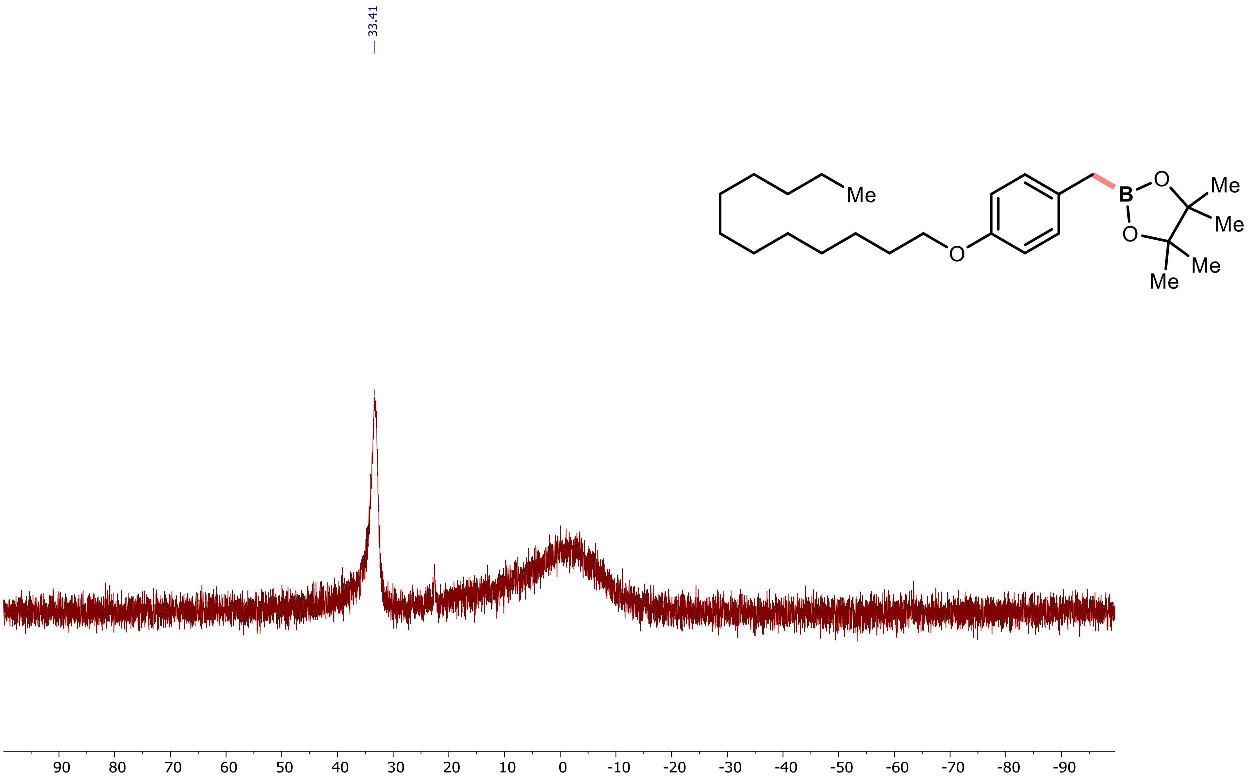

Supplementary Figure 26b-1 ^{1}H NMR (500 MHz, $CDCl_3$) 2-(Benzo[*d*][1,3]dioxol-5-ylmethyl)-4,4,5,5-tetramethyl-1,3,2-dioxaborolane

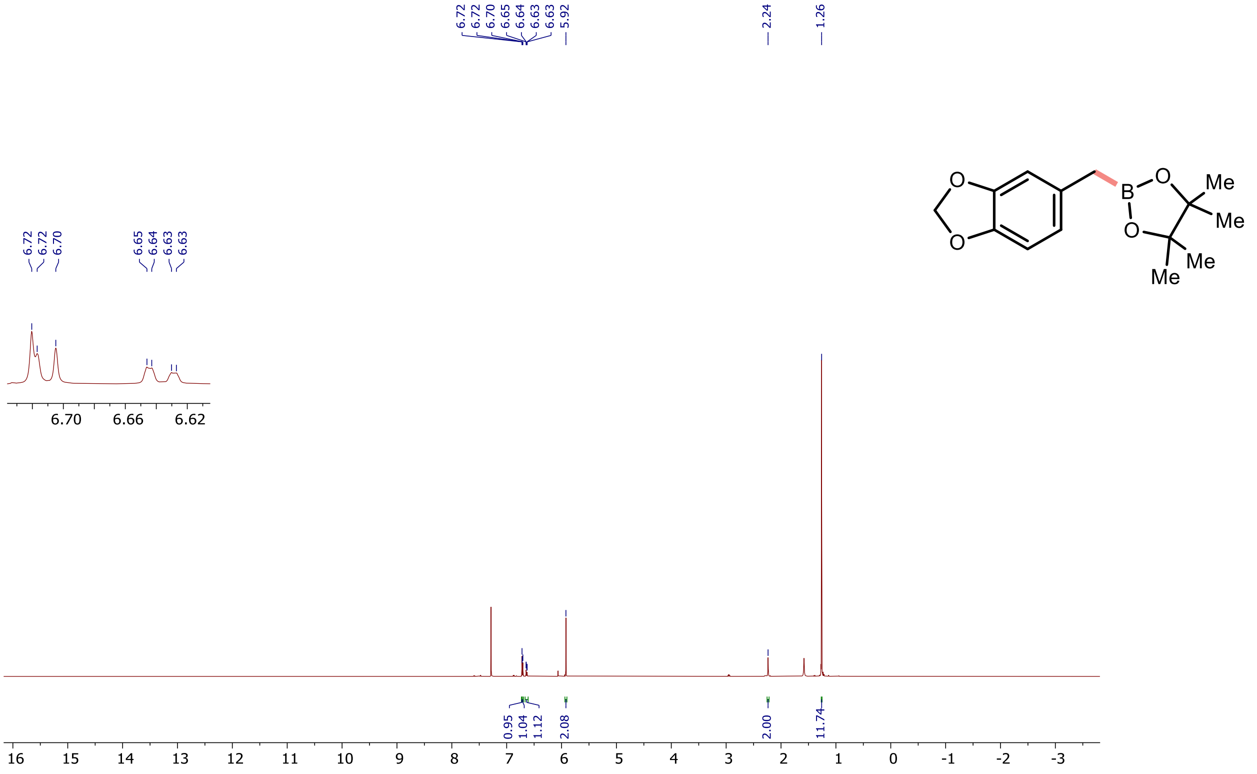

Supplementary Figure 26b-2 ^{13}C NMR (125 MHz, $CDCl_3$) 2-(Benzo[*d*][1,3]dioxol-5-ylmethyl)-4,4,5,5-tetramethyl-1,3,2-dioxaborolane

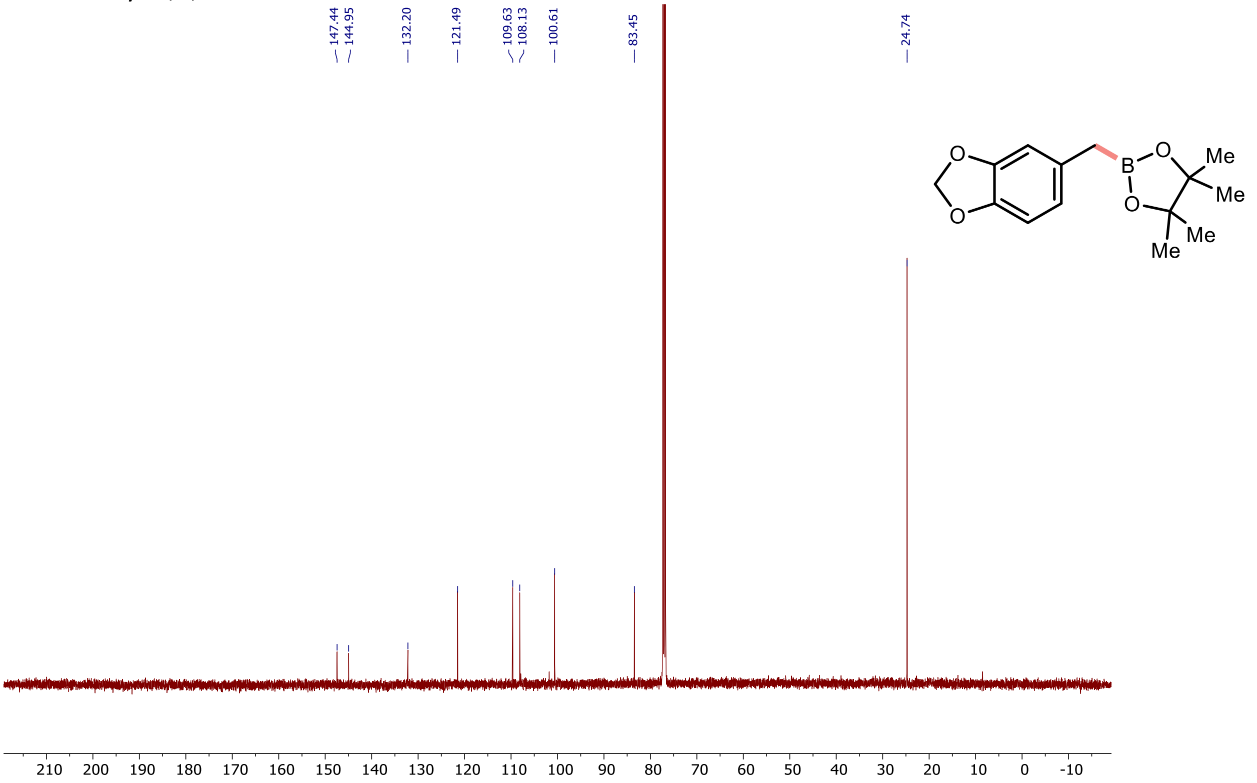

Supplementary Figure 26b-3 ^{11}B NMR (161 MHz, $CDCl_3$) 2-(Benzo[*d*][1,3]dioxol-5-ylmethyl)-4,4,5,5-tetramethyl-1,3,2-dioxaborolane

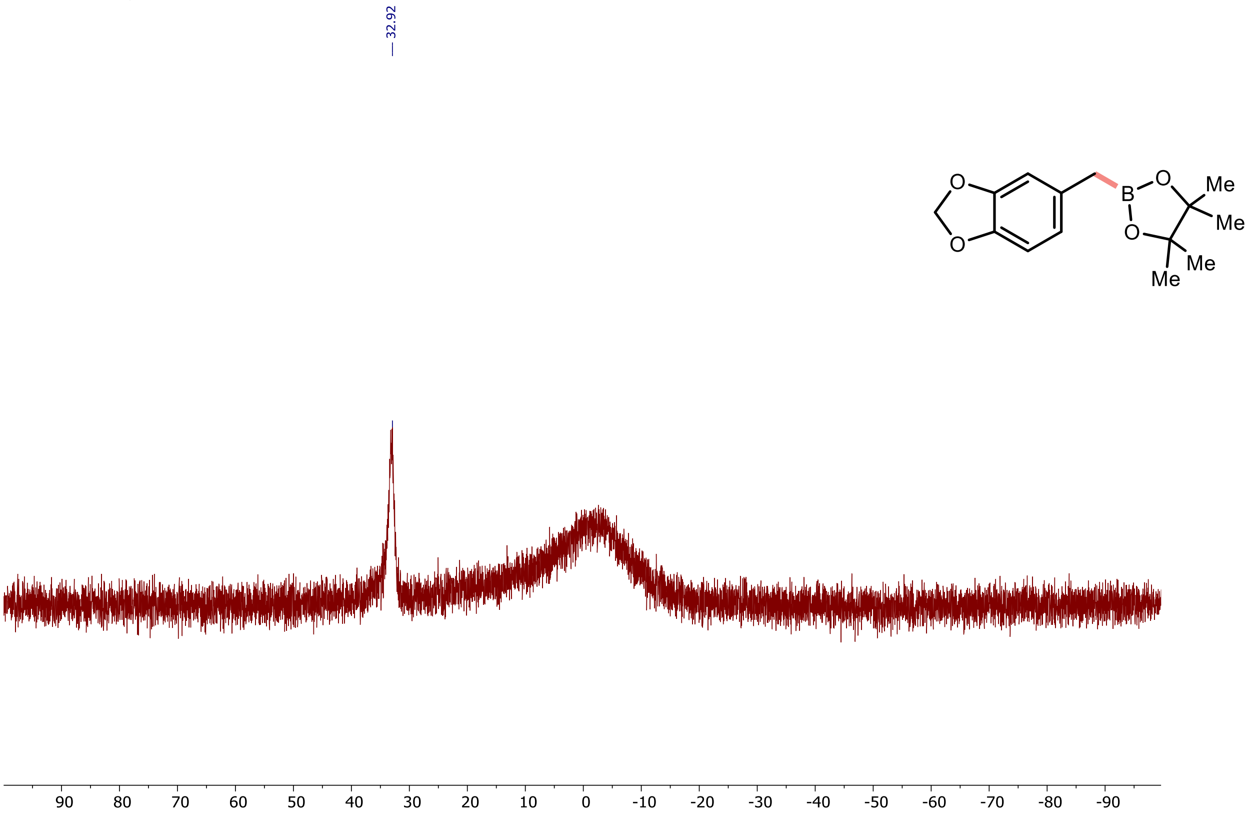

Supplementary Figure 27d-1 ^{1}H NMR (500 MHz, $CDCl_3$) (3,4,5-Trimethoxyphenyl)methanol

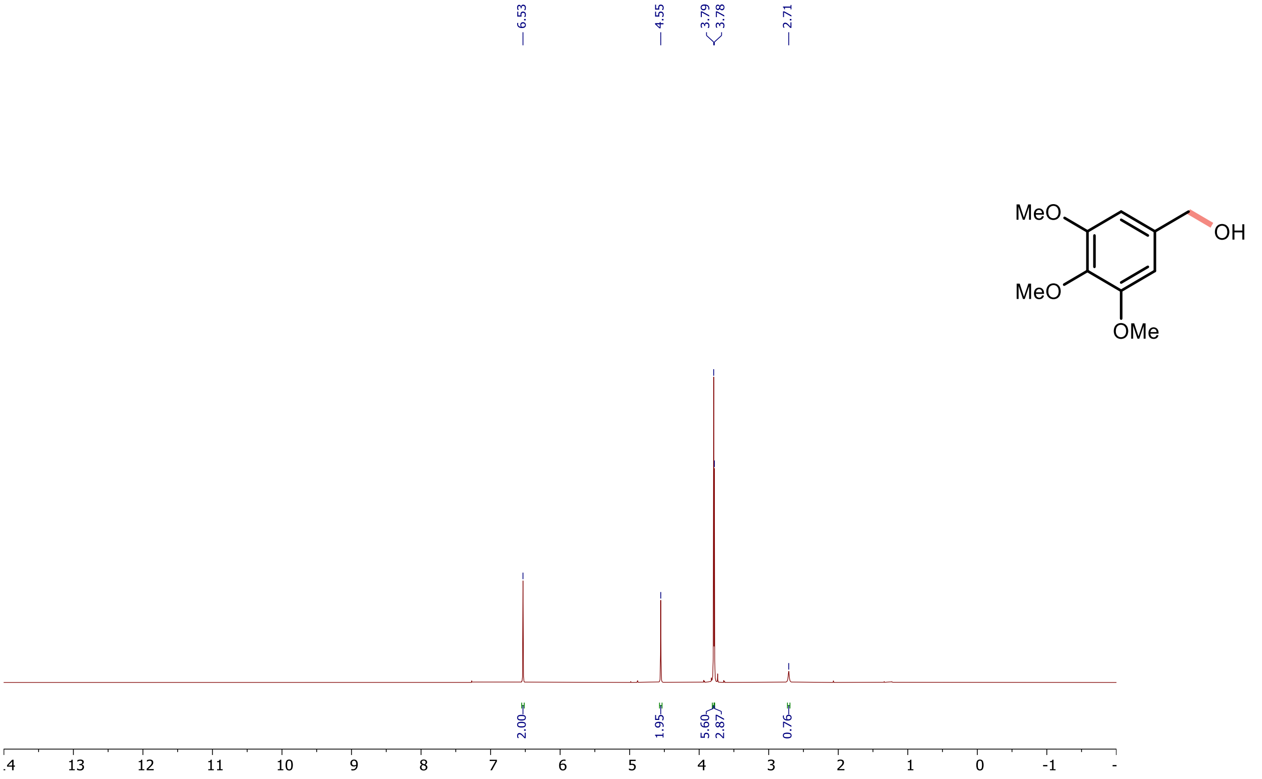

Supplementary Figure 27d-2 ^{13}C NMR (125 MHz, $CDCl_3$) (3,4,5-Trimethoxyphenyl)methanol

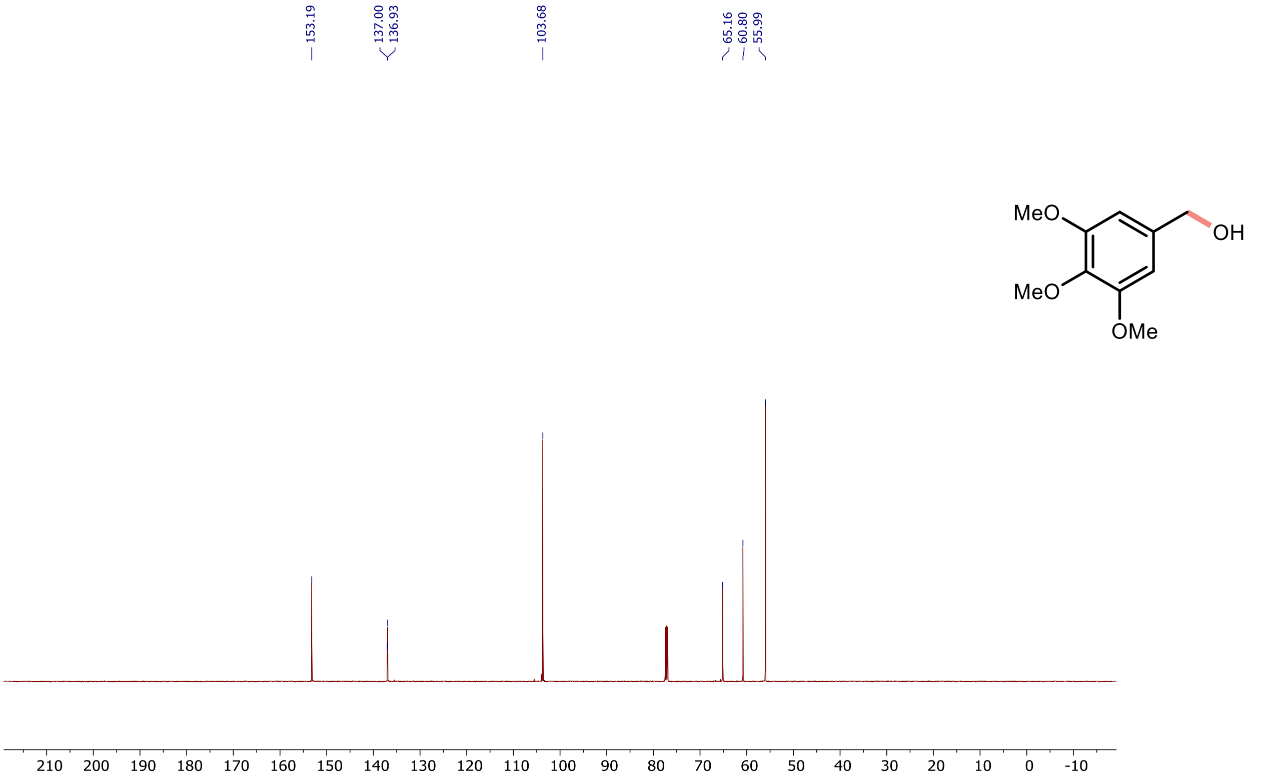

Supplementary Figure 28d-1 ^{1}H NMR (500 MHz, DMSO-d_6) 4-(Hydroxymethyl)phenol

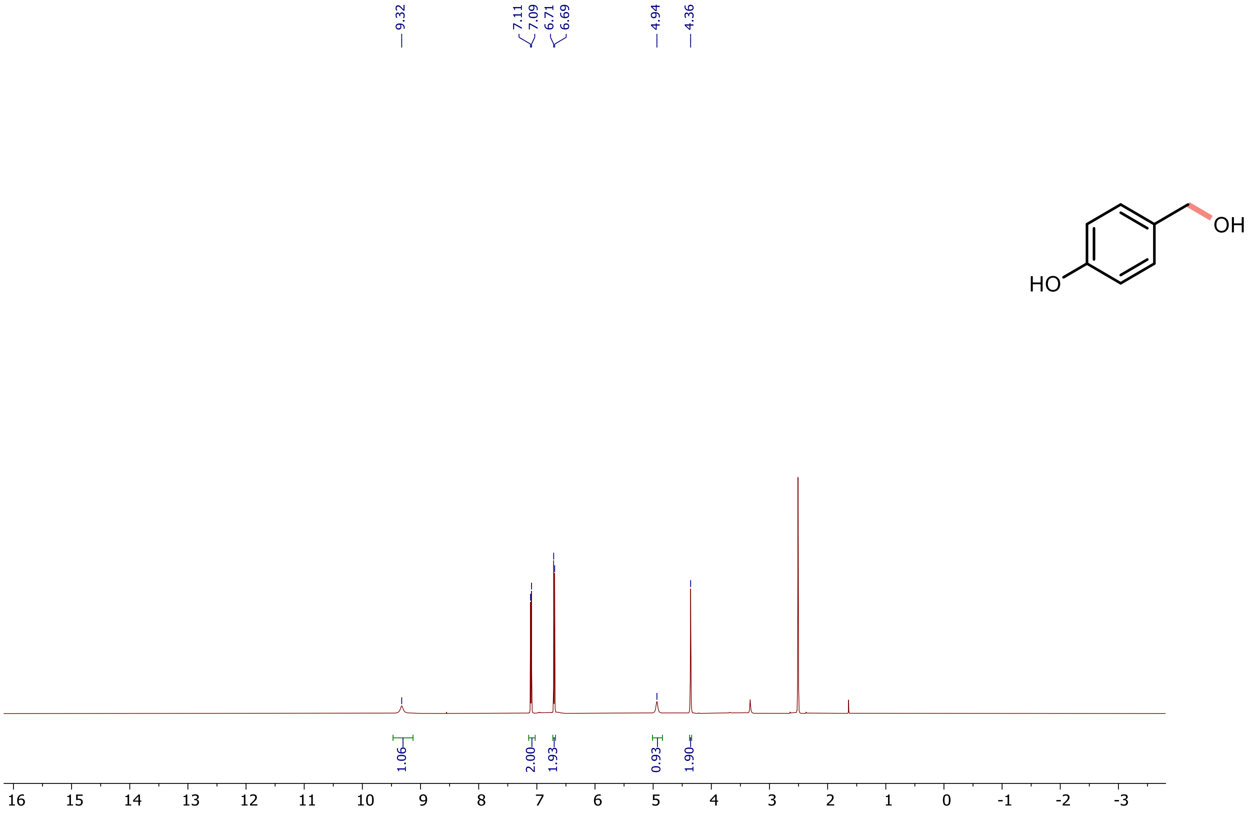

Supplementary Figure 28d-2 ^{13}C NMR (125 MHz, DMSO-d_6) 4-(Hydroxymethyl)phenol

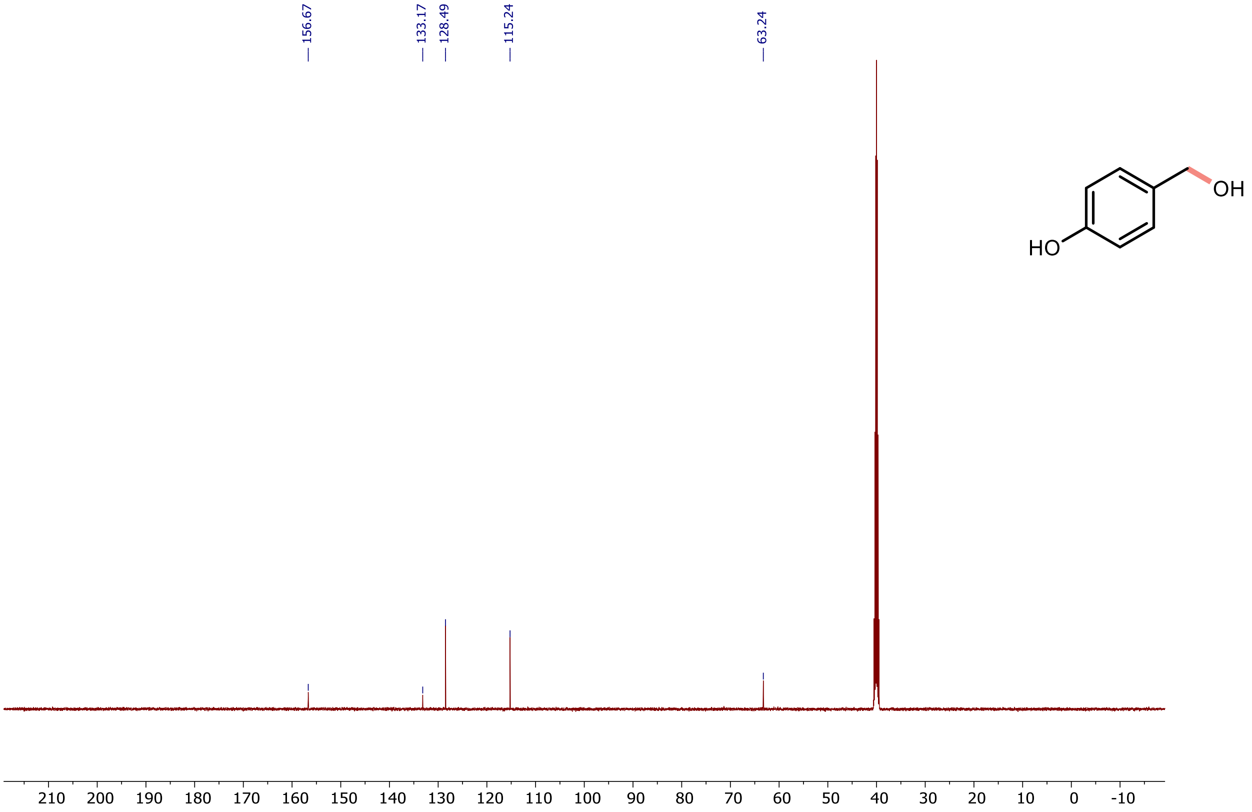

Supplementary Figure 29b-1 ^1H NMR (500 MHz, $CDCl_3$) 4,4,5,5-Tetramethyl-2-(4-(trifluoromethoxy)benzyl)-1,3,2-dioxaborolane

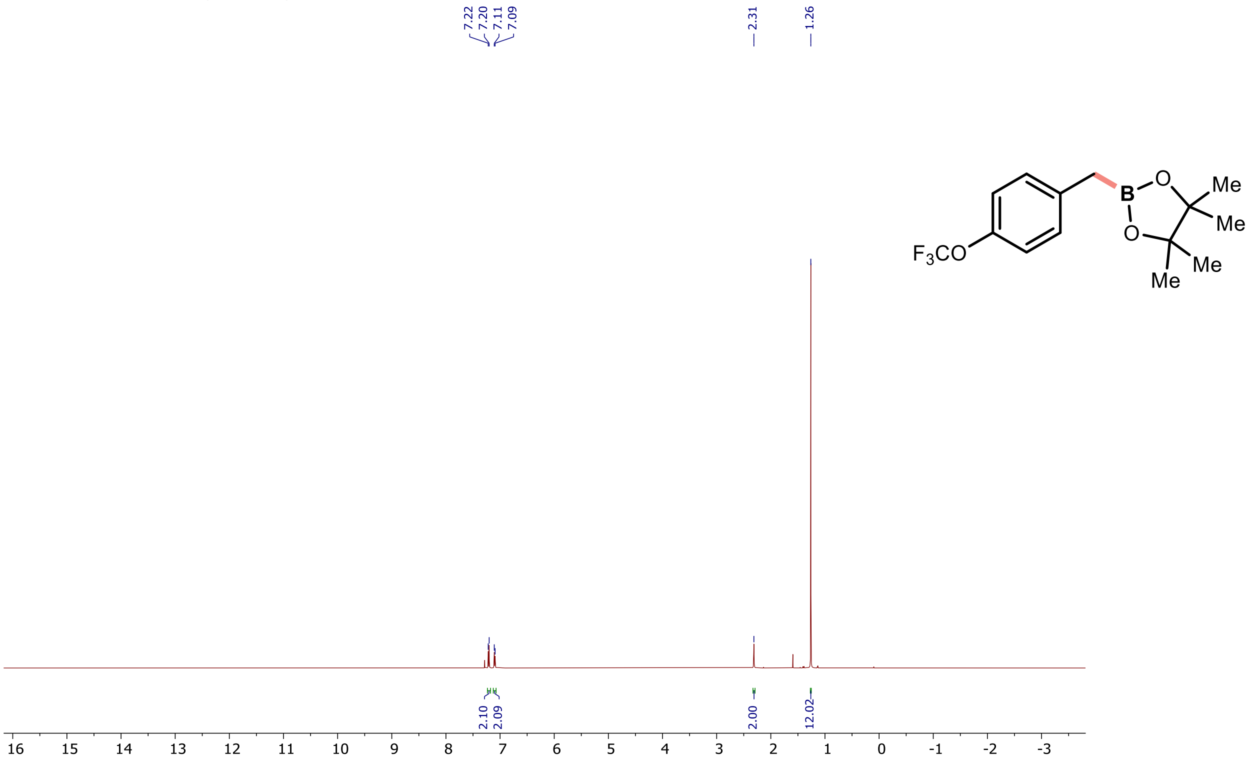

Supplementary Figure 29b-2 ^{13}C NMR (125 MHz, $CDCl_3$) 4,4,5,5-Tetramethyl-2-(4-(trifluoromethoxy)benzyl)-1,3,2-dioxaborolane

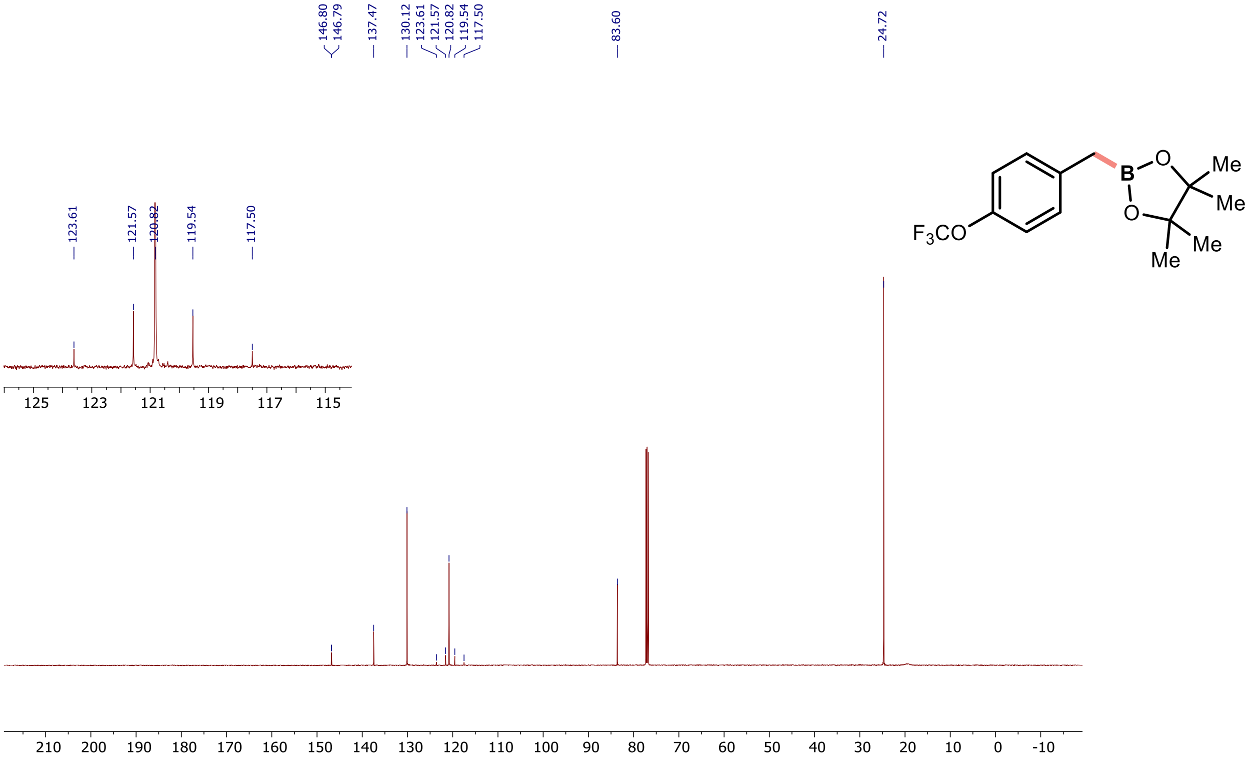

Supplementary Figure 29b-3 ^{19}F NMR (471 MHz, $CDCl_3$) 4,4,5,5-Tetramethyl-2-(4-(trifluoromethoxy)benzyl)-1,3,2-dioxaborolane

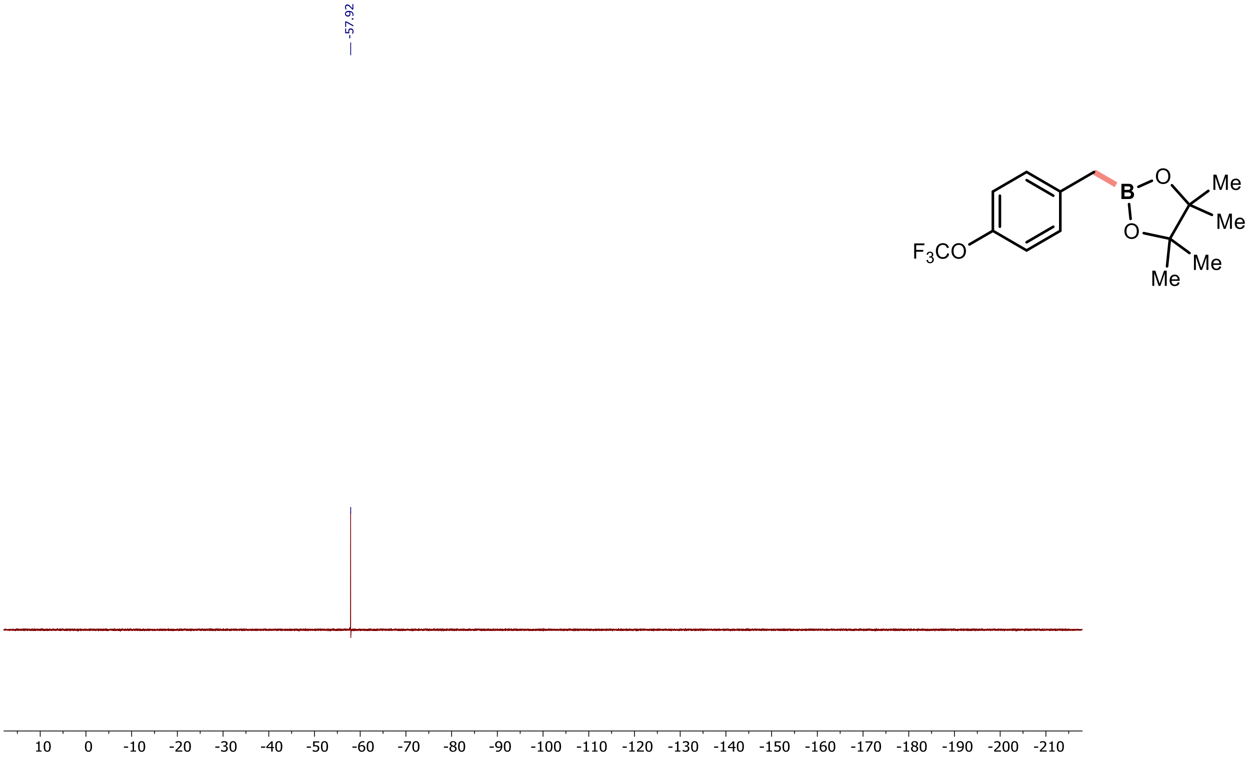

Supplementary Figure 29b-4 ^{11}B NMR (161 MHz, $CDCl_3$) 4,4,5,5-Tetramethyl-2-(4-(trifluoromethoxy)benzyl)-1,3,2-dioxaborolane

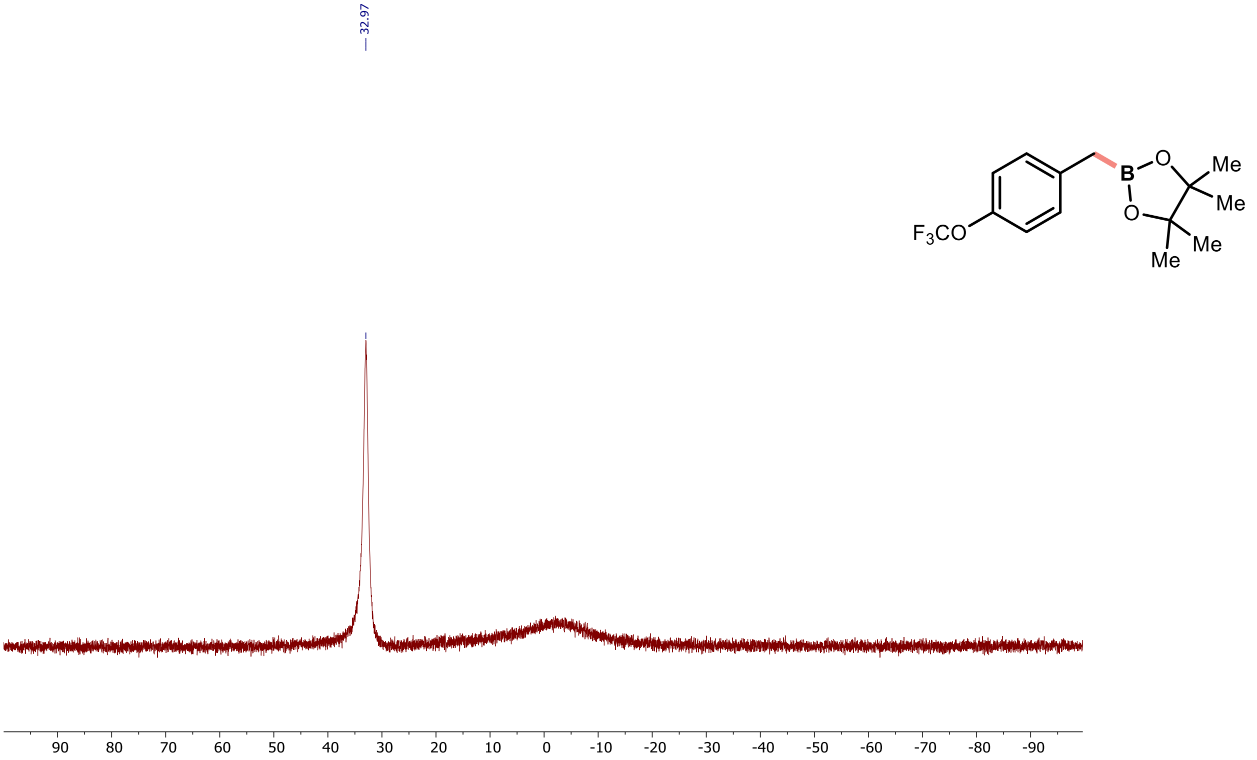

Supplementary Figure 30d-1 ^{1}H NMR (500 MHz, $CDCl_3$) (4-(Dimethylamino)phenyl)methanol

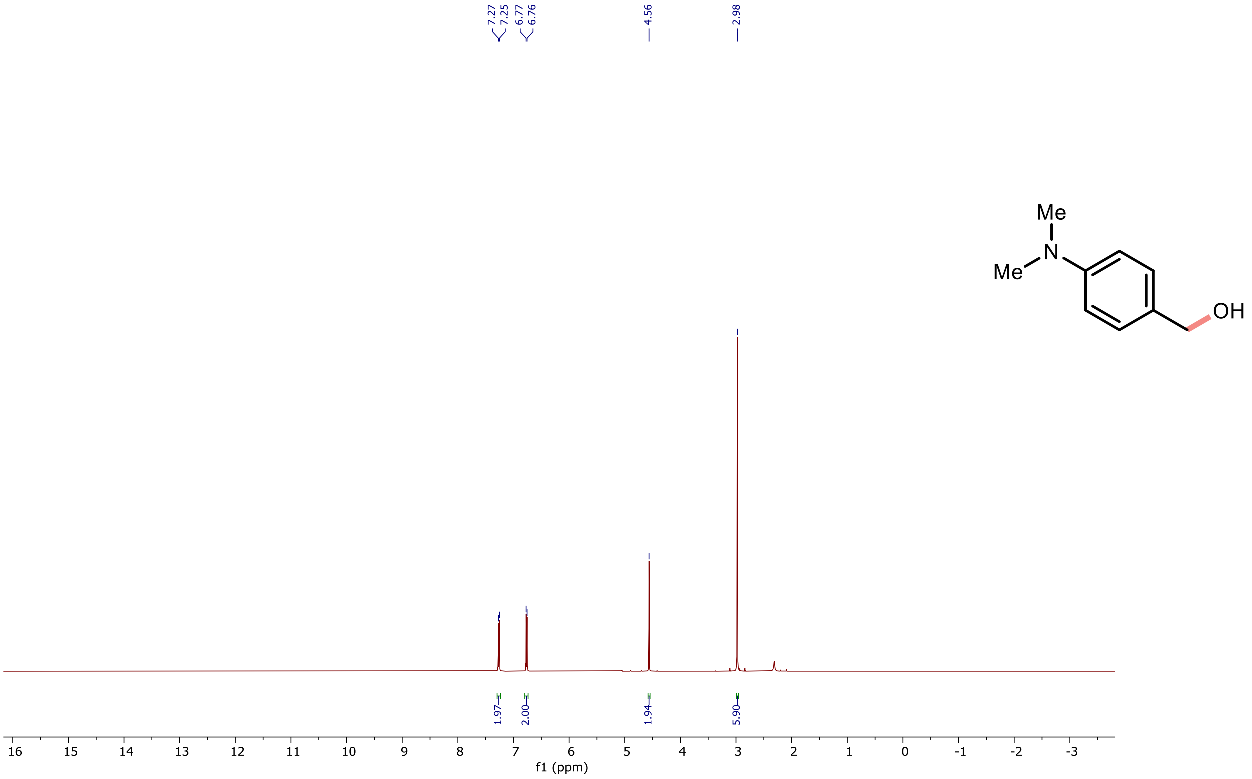

Supplementary Figure 30d-2 ^{13}C NMR (125 MHz, $CDCl_3$) (4-(Dimethylamino)phenyl)methanol

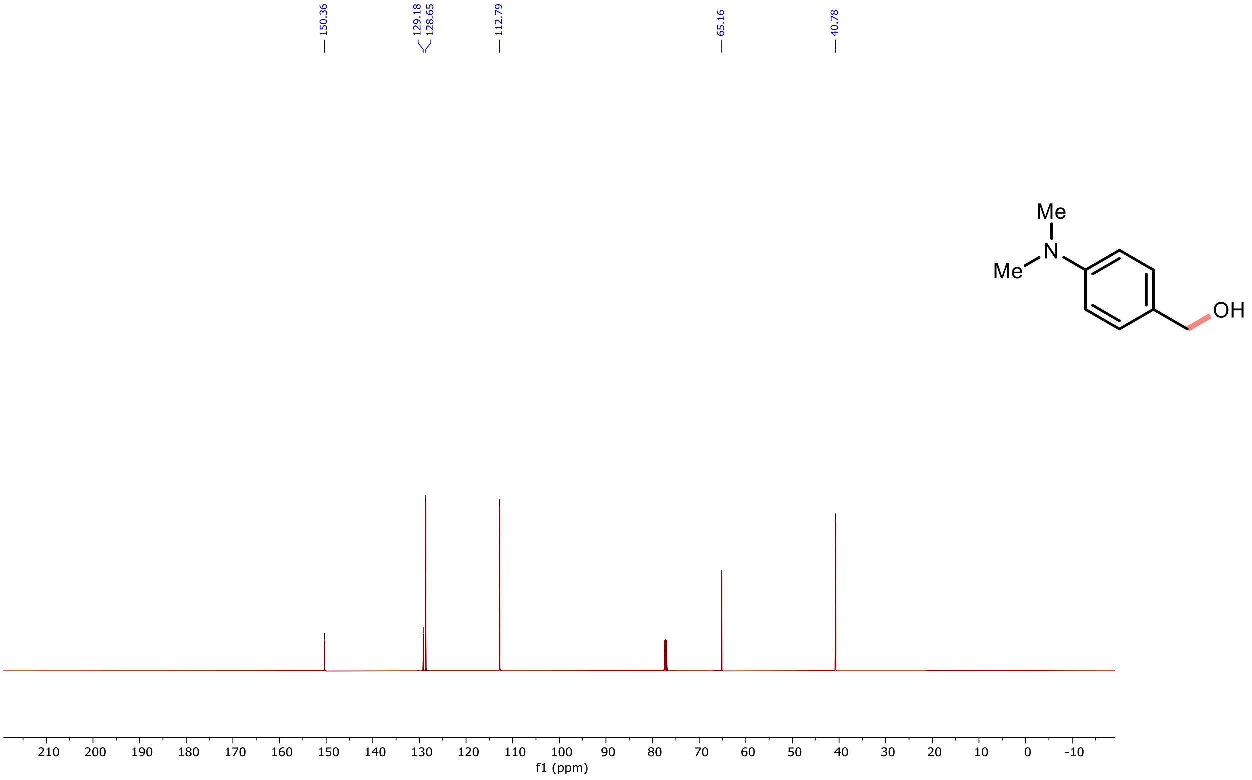

Supplementary Figure 31b-1 ^{1}H NMR (500 MHz, $CDCl_3$) 4,4,5,5-Tetramethyl-2-(4-(methylthio)benzyl)-1,3,2-dioxaborolane

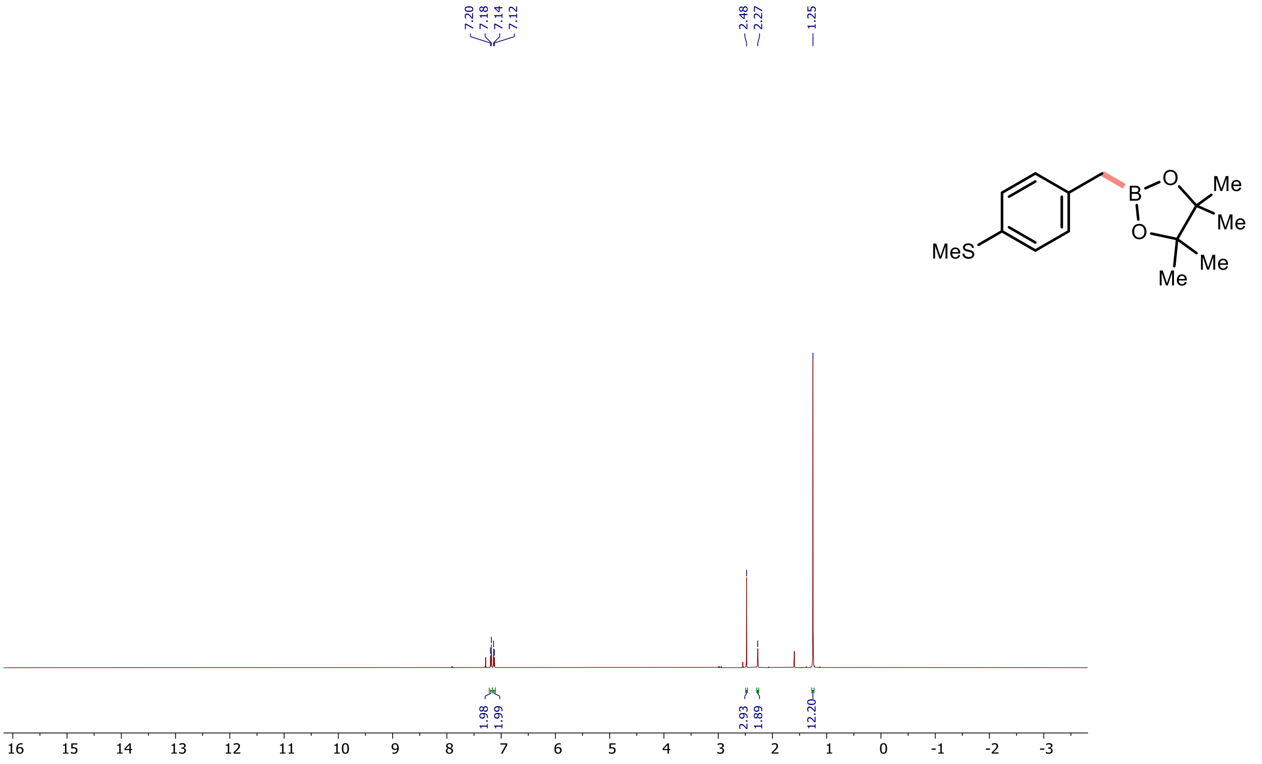

Supplementary Figure 31b-2 ^{13}C NMR (125 MHz, $CDCl_3$) 4,4,5,5-Tetramethyl-2-(4-(methylthio)benzyl)-1,3,2-dioxaborolane

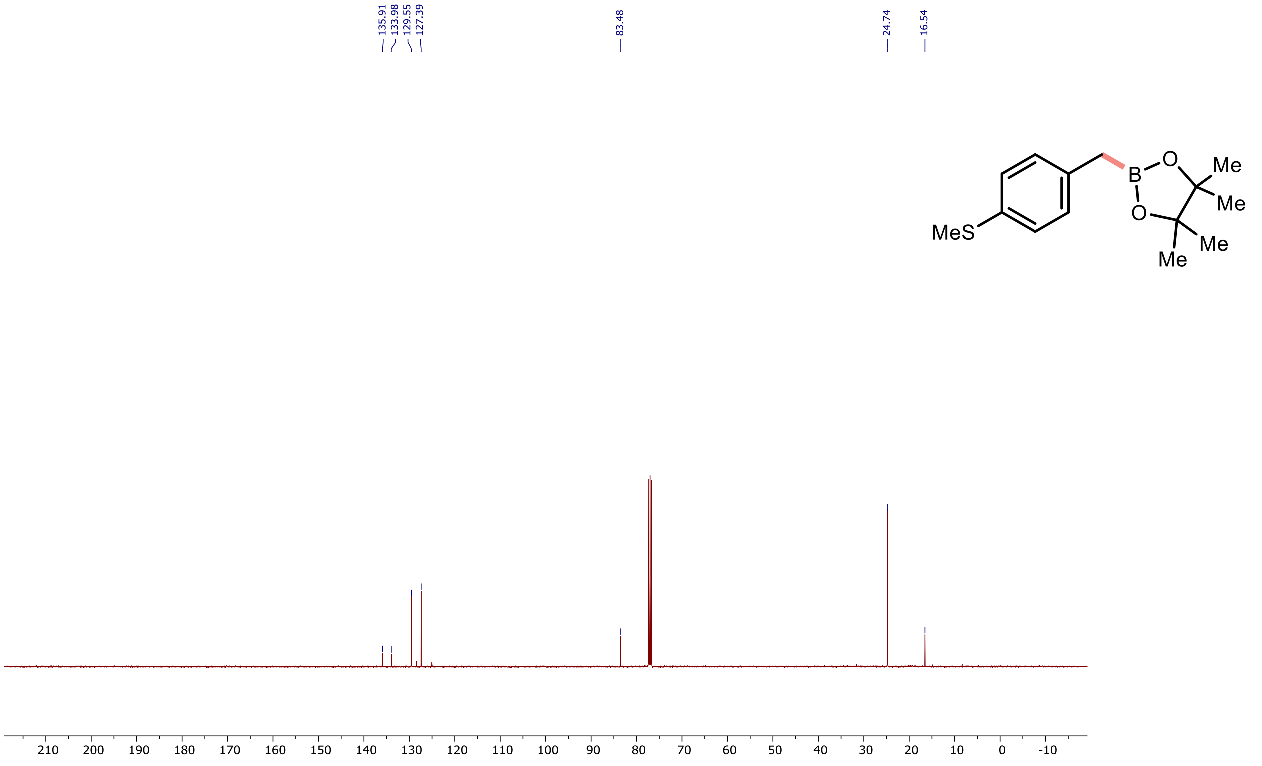

Supplementary Figure 31b-3 ^{11}B NMR (161 MHz, $CDCl_3$) 4,4,5,5-Tetramethyl-2-(4-(methylthio)benzyl)-1,3,2-dioxaborolane

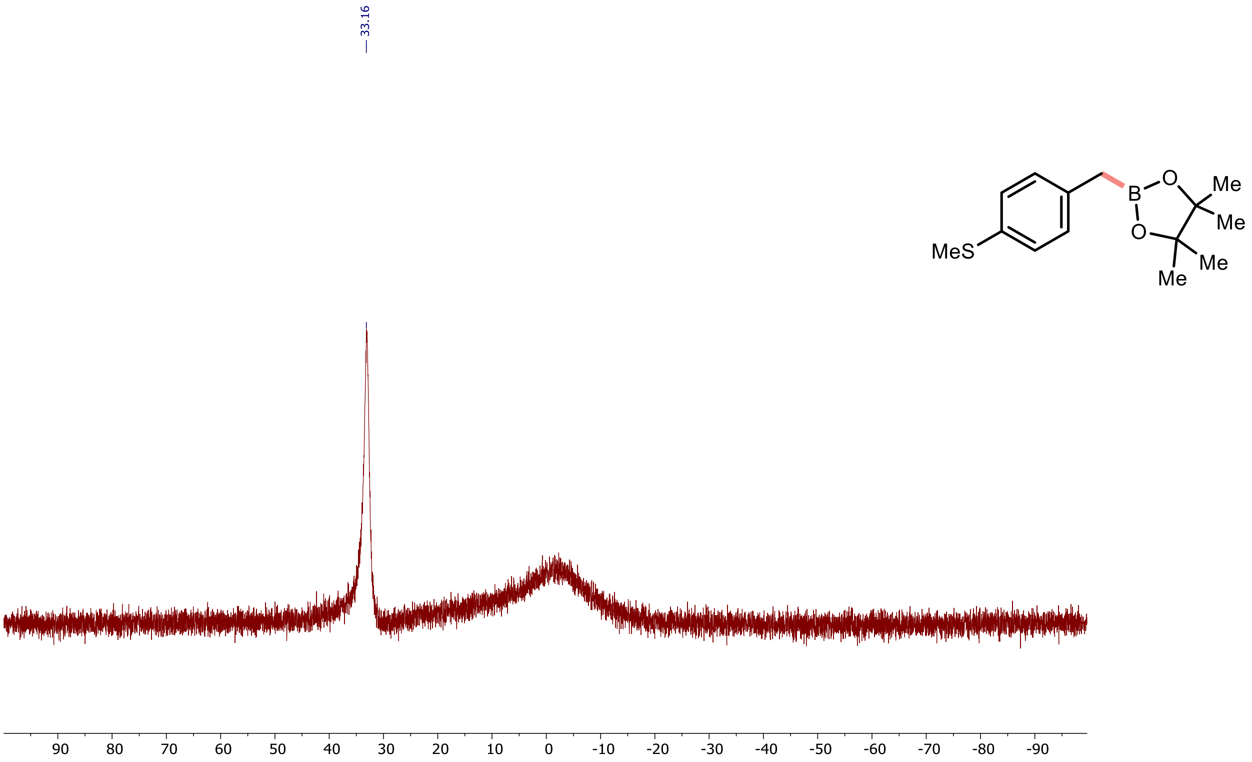

Supplementary Figure 32b-1 ^{1}H NMR (500 MHz, $CDCl_3$) 9-Ethyl-3-((4,4,5,5-tetramethyl-1,3,2-dioxaborolan-2-yl)methyl)-9*H*-carbazole

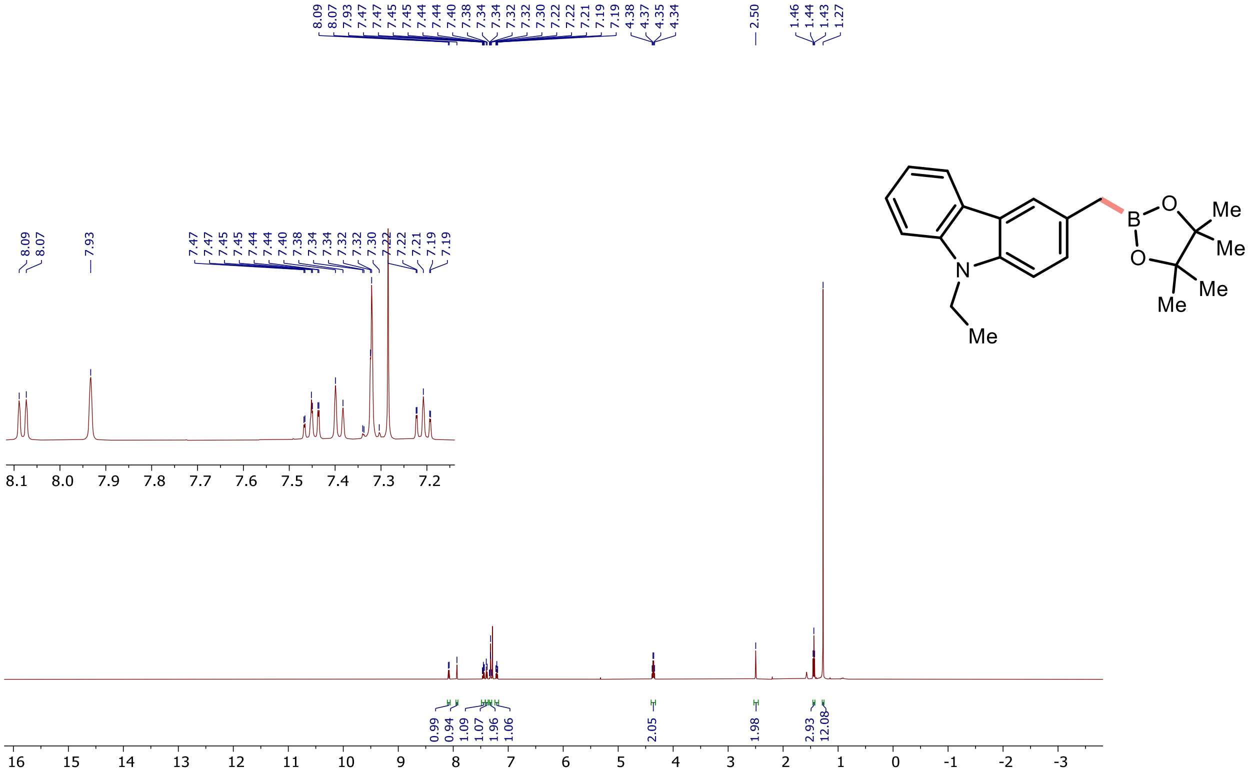

Supplementary Figure 31b-2 ^{13}C NMR (125 MHz, $CDCl_3$) 9-Ethyl-3-((4,4,5,5-tetramethyl-1,3,2-dioxaborolan-2-yl)methyl)-9*H*-carbazole

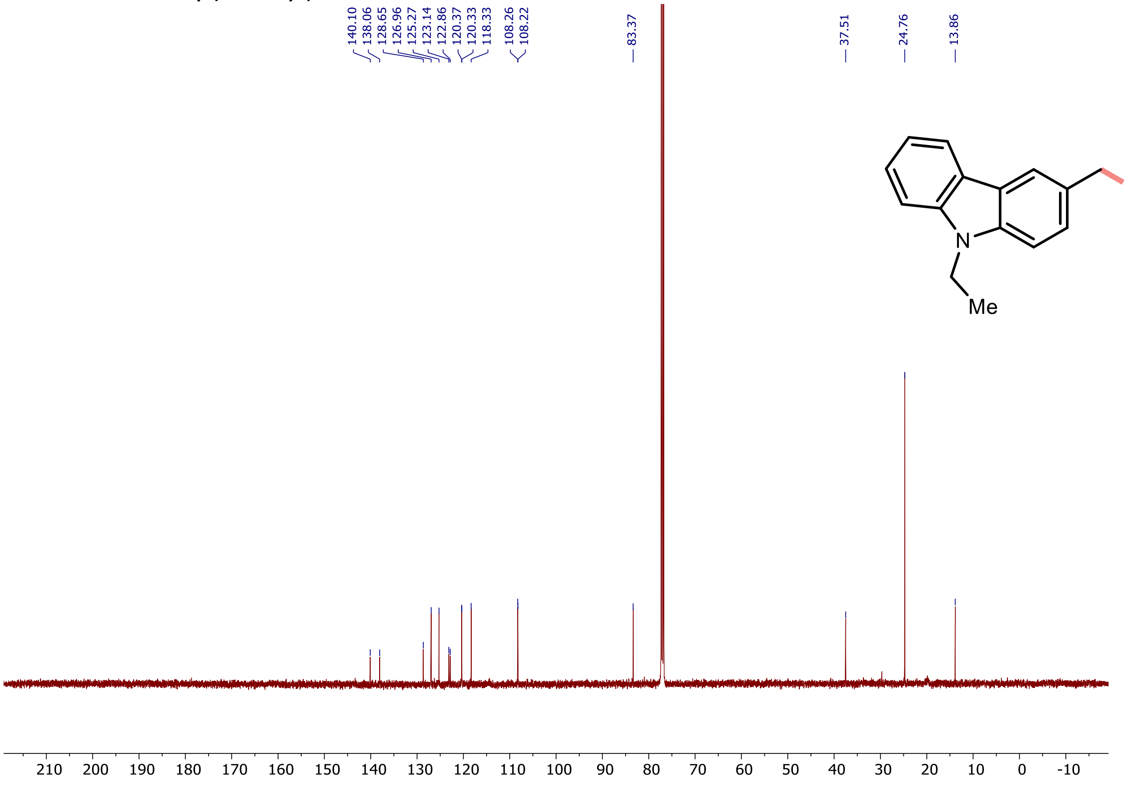

Supplementary Figure 32b-3 ^{11}B NMR (161 MHz, $CDCl_3$) 9-Ethyl-3-((4,4,5,5-tetramethyl-1,3,2-dioxaborolan-2-yl)methyl)-9*H*-carbazole

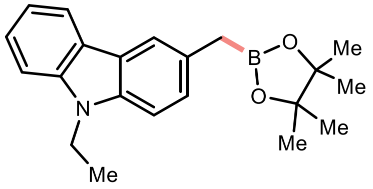

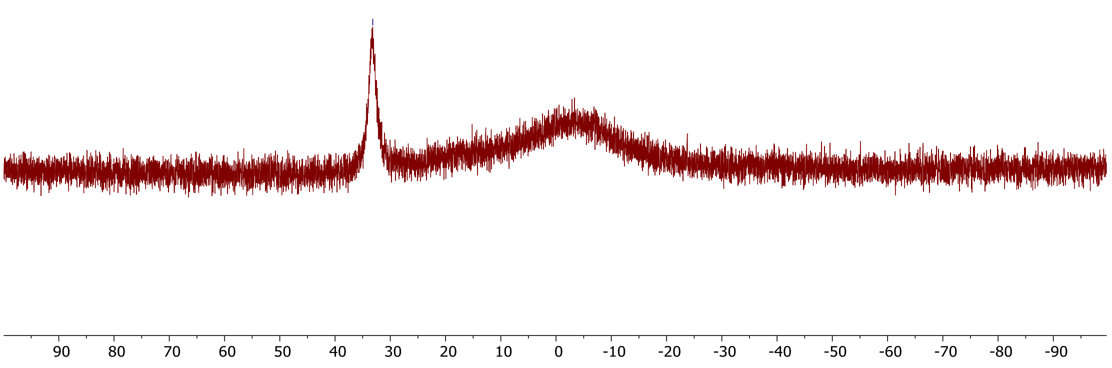

Supplementary Figure 33b-1 ^{1}H NMR (500 MHz, $CDCl_3$) 2-(Benzofuran-5-ylmethyl)-4,4,5,5-tetramethyl-1,3,2-dioxaborolane

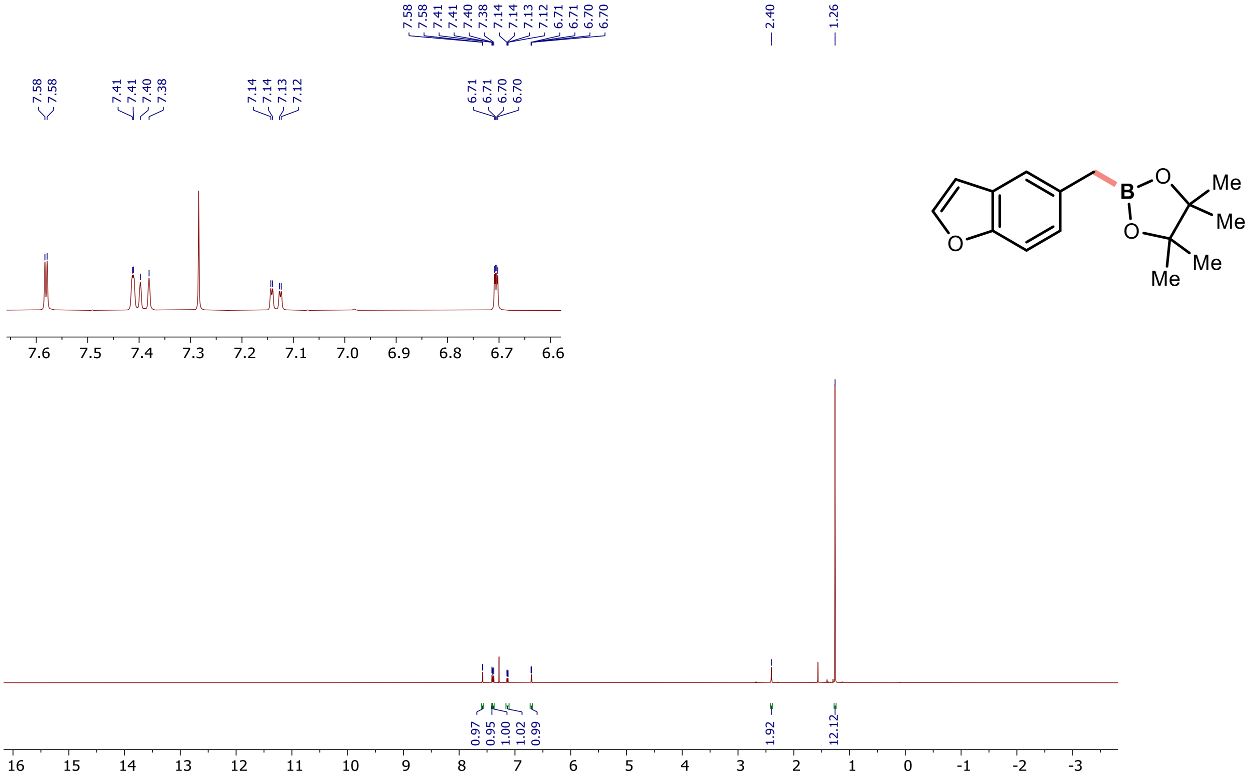

Supplementary Figure 33b-2 ^{13}C NMR (125 MHz, $CDCl_3$) 2-(Benzofuran-5-ylmethyl)-4,4,5,5-tetramethyl-1,3,2-dioxaborolane

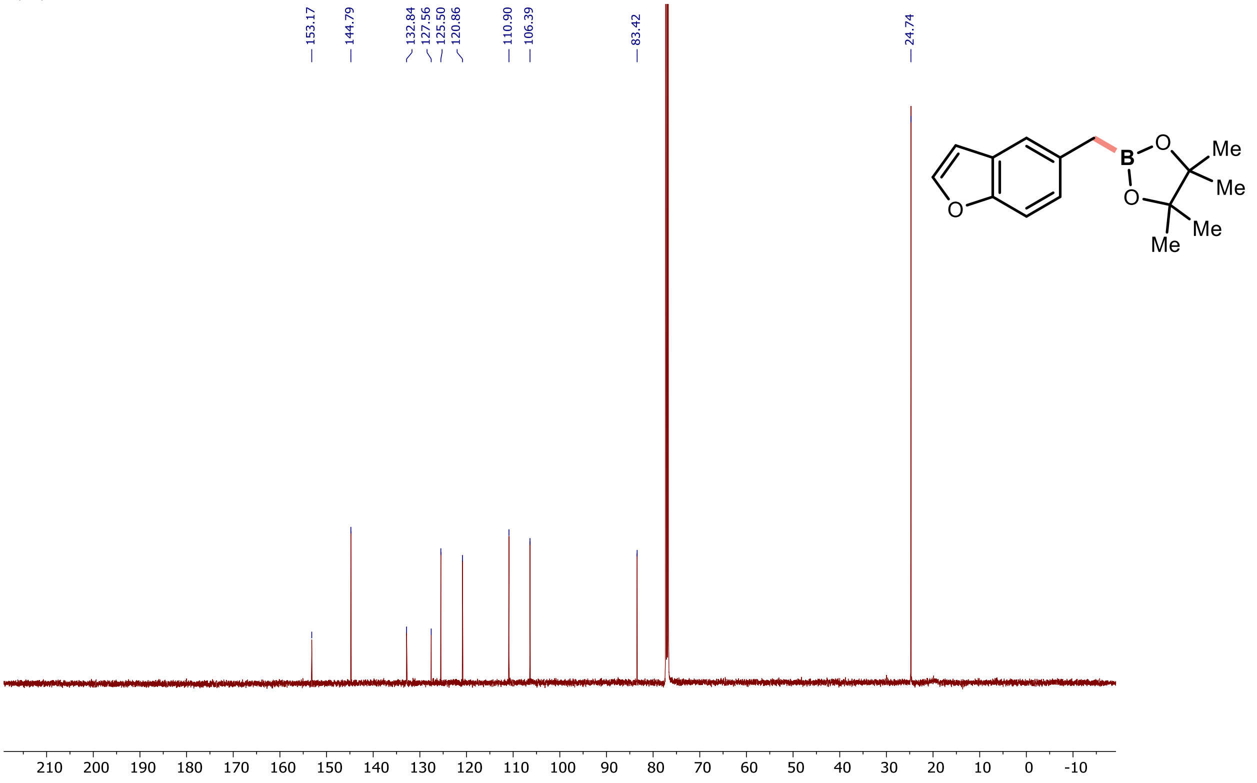

Supplementary Figure 33b-3 ^{11}B NMR (161 MHz, $CDCl_3$) 2-(Benzofuran-5-ylmethyl)-4,4,5,5-tetramethyl-1,3,2-dioxaborolane

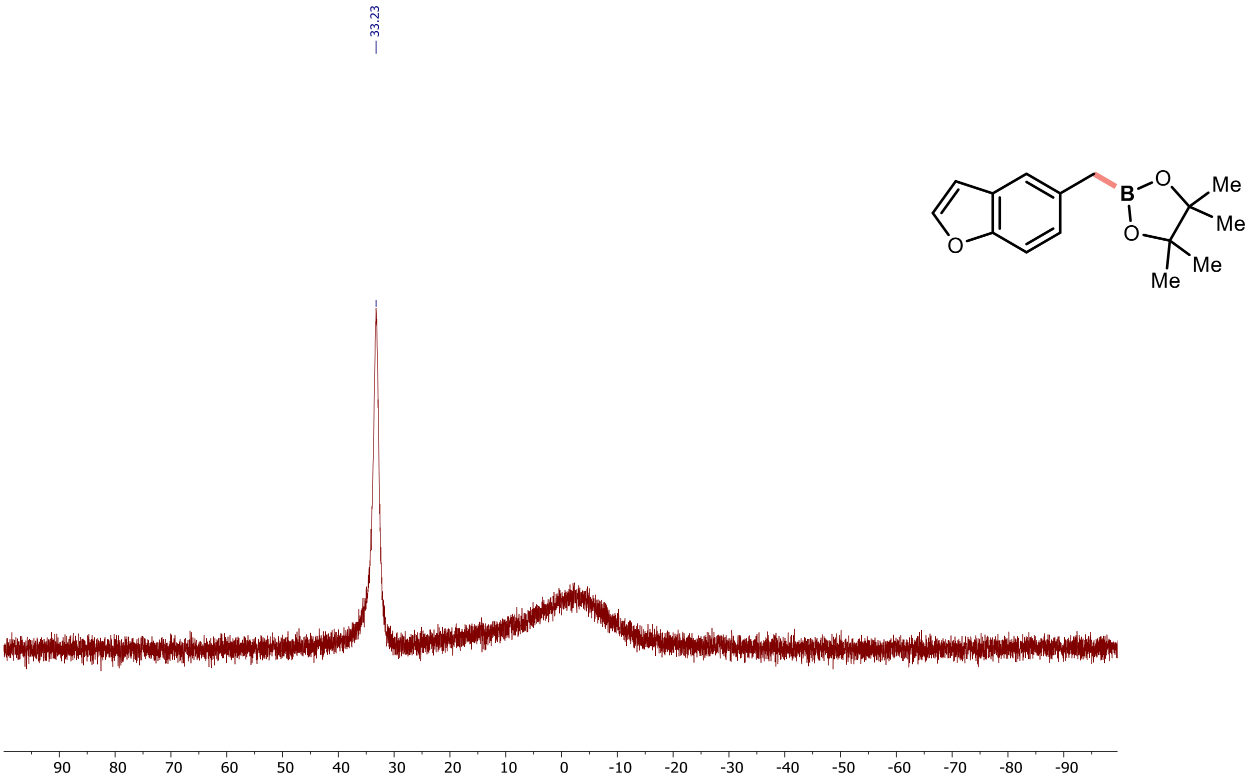

Supplementary Figure 34b-1 ^{1}H NMR (500 MHz, $CDCl_3$) 2-(Benzo[*b*]thiophen-5-ylmethyl)-4,4,5,5-tetramethyl-1,3,2-dioxaborolane

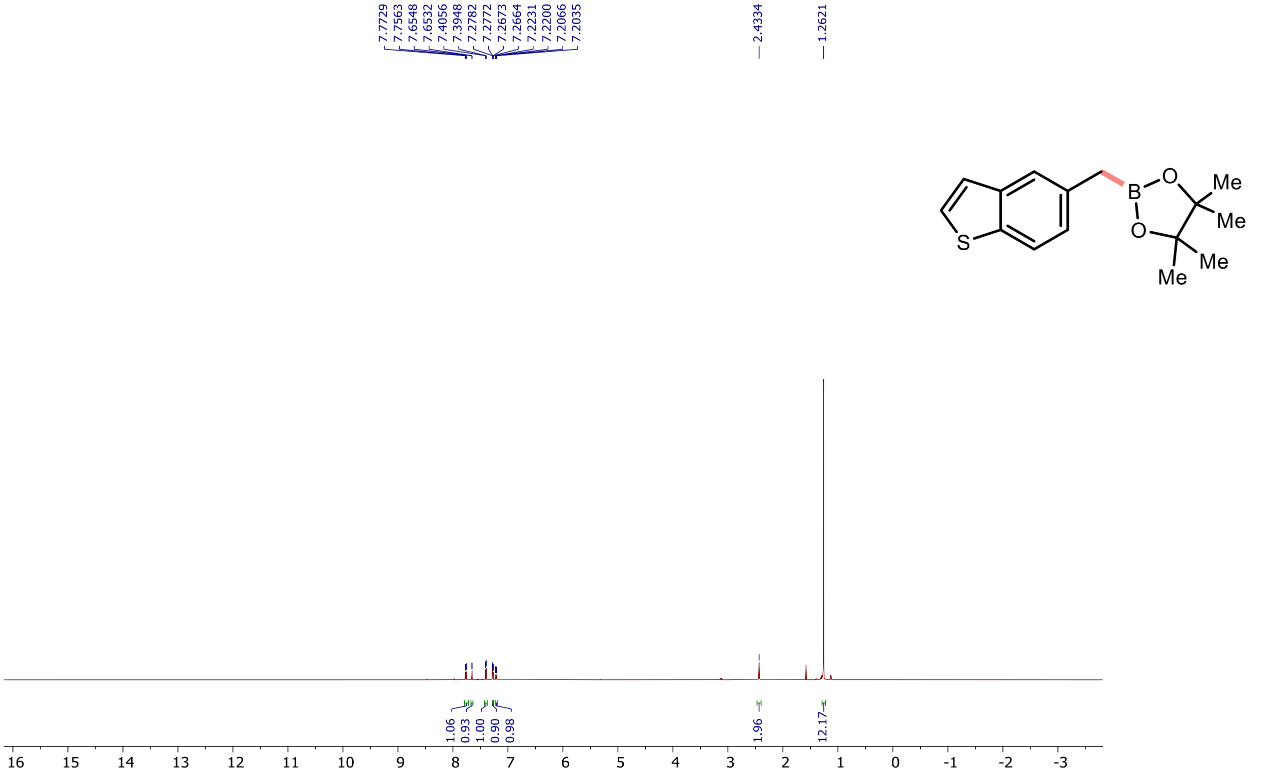

Supplementary Figure 34b-2 ^{13}C NMR (125 MHz, $CDCl_3$) 2-(Benzo[*b*]thiophen-5-ylmethyl)-4,4,5,5-tetramethyl-1,3,2-dioxaborolane

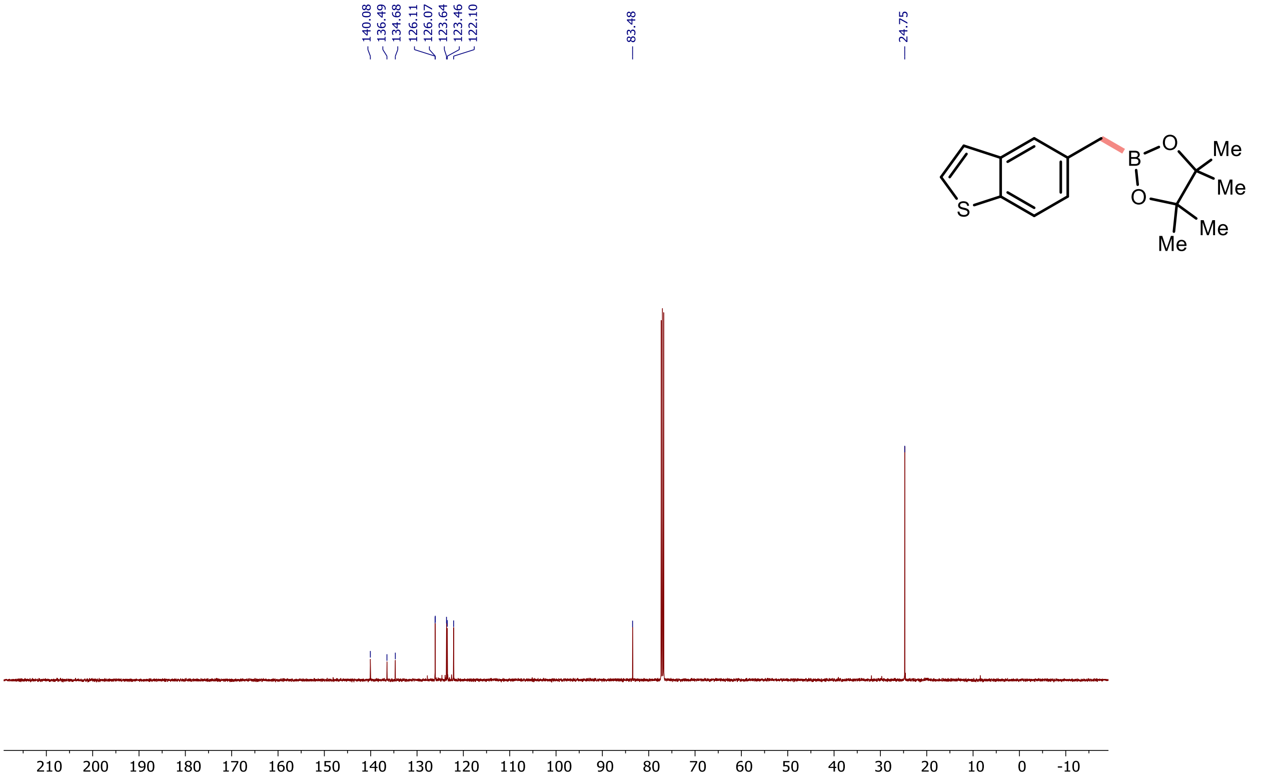

Supplementary Figure 34b-3 ^{11}B NMR (161 MHz, $CDCl_3$) 2-(Benzo[*b*]thiophen-5-ylmethyl)-4,4,5,5-tetramethyl-1,3,2-dioxaborolane

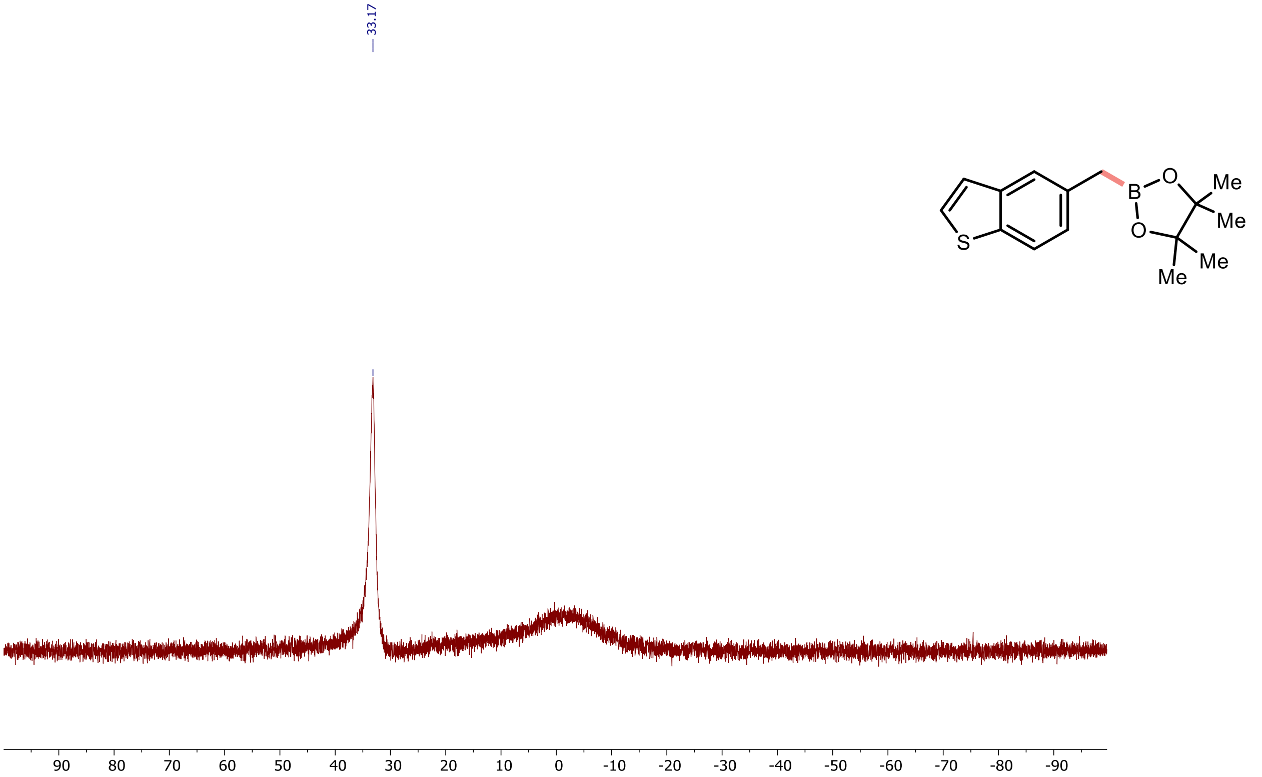

Supplementary Figure 35b-2 ^{1}H NMR (500 MHz, $CDCl_3$) 2,2',2''-(Ethane-1,1,2-triyl)tris(4,4,5,5-tetramethyl-1,3,2-dioxaborolane)

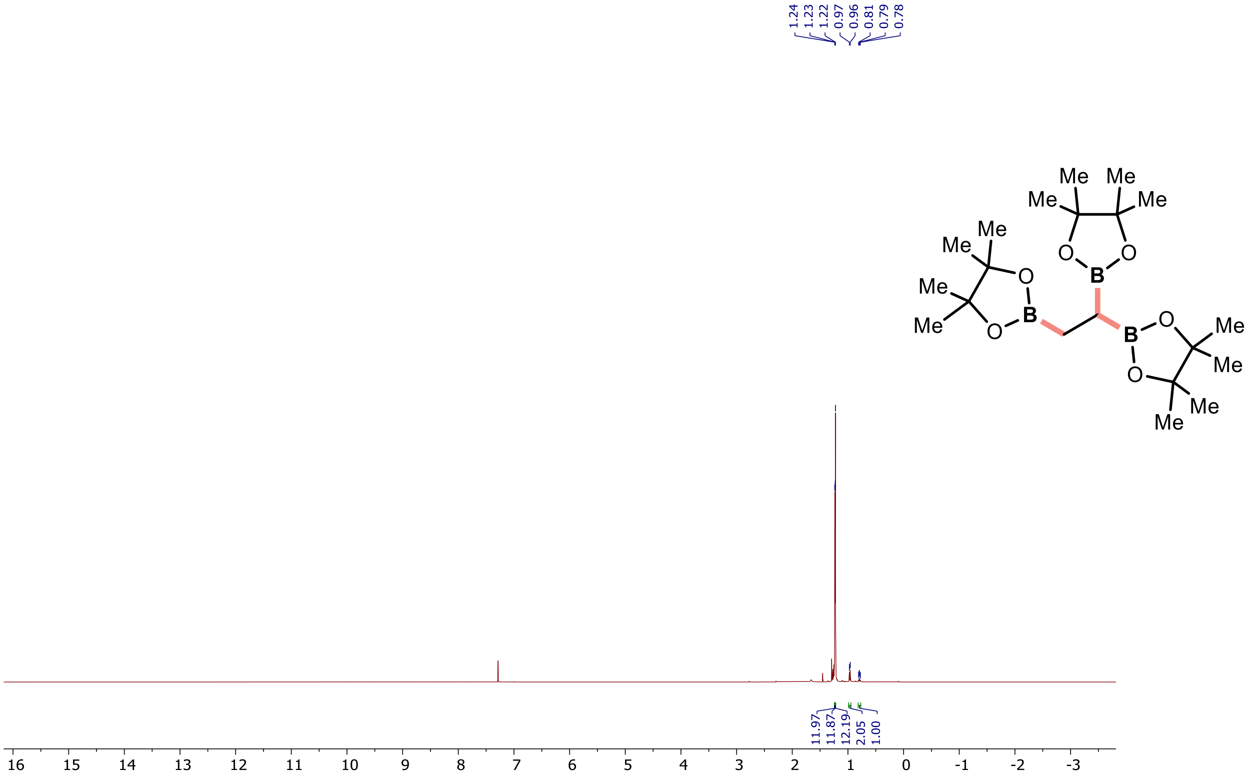

Supplementary Figure 35b-2 ^{13}C NMR (125 MHz, $CDCl_3$) 2,2',2''-(Ethane-1,1,2-triyl)tris(4,4,5,5-tetramethyl-1,3,2-dioxaborolane)

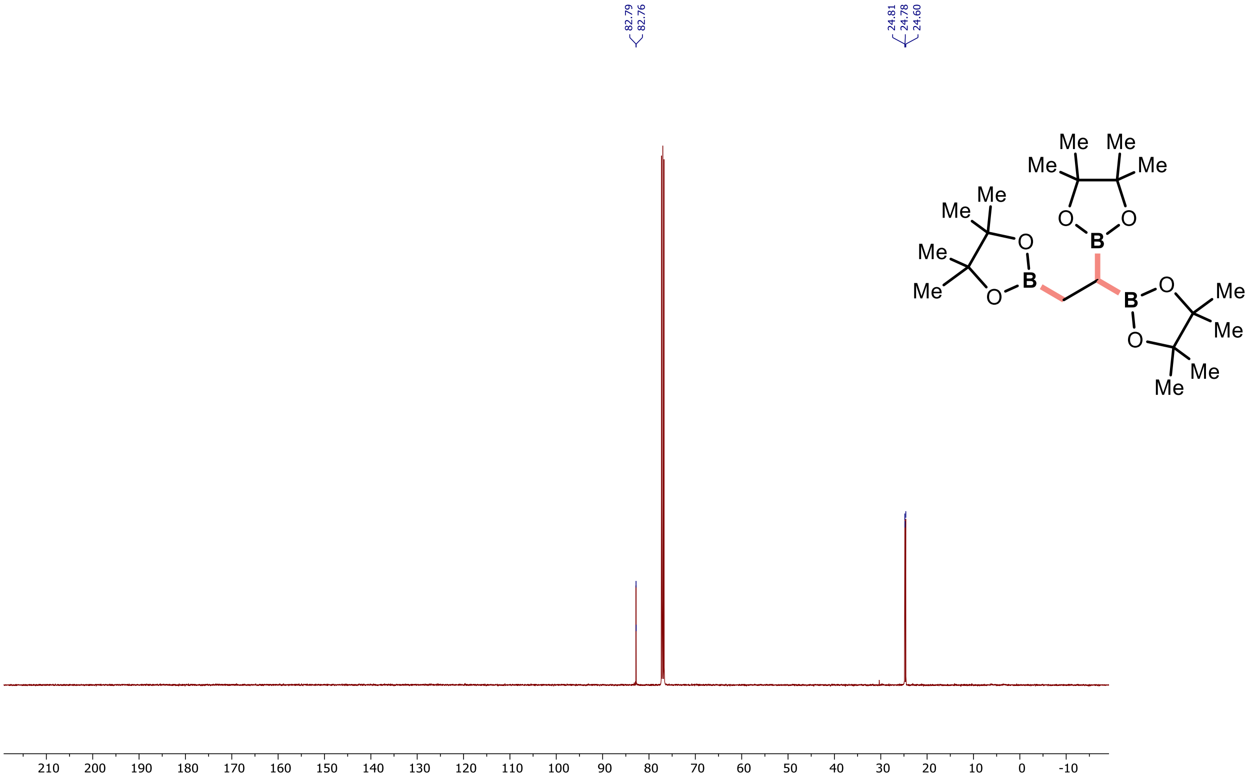

Supplementary Figure 35b-3 ^{11}B NMR (161 MHz, $CDCl_3$) 2,2',2''-(Ethane-1,1,2-triyl)tris(4,4,5,5-tetramethyl-1,3,2-dioxaborolane)

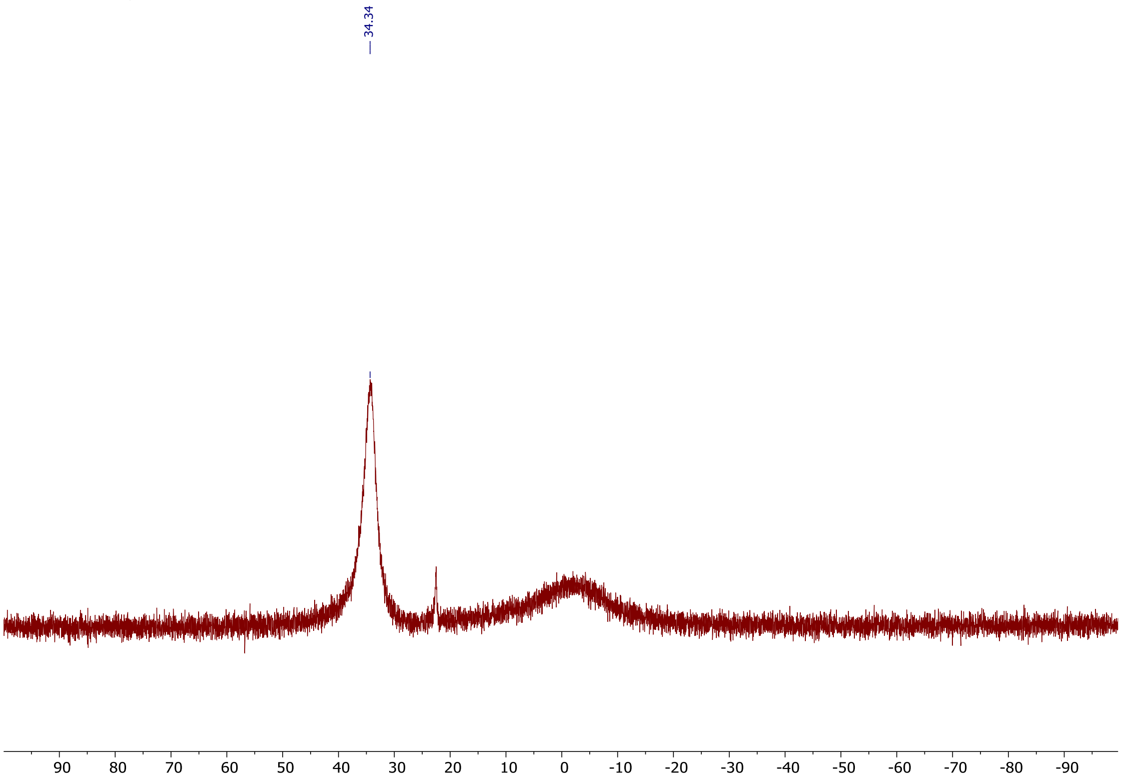

Supplementary Figure 35b-$^{13}C_1$-1 ^{1}H NMR (500 MHz, $CDCl_3$) 2,2',2''-(1λ^3-ethane-1,1,2-triyl-1-^{13}C)tris(4,4,5,5-tetramethyl-1,3,2-dioxaborolane)

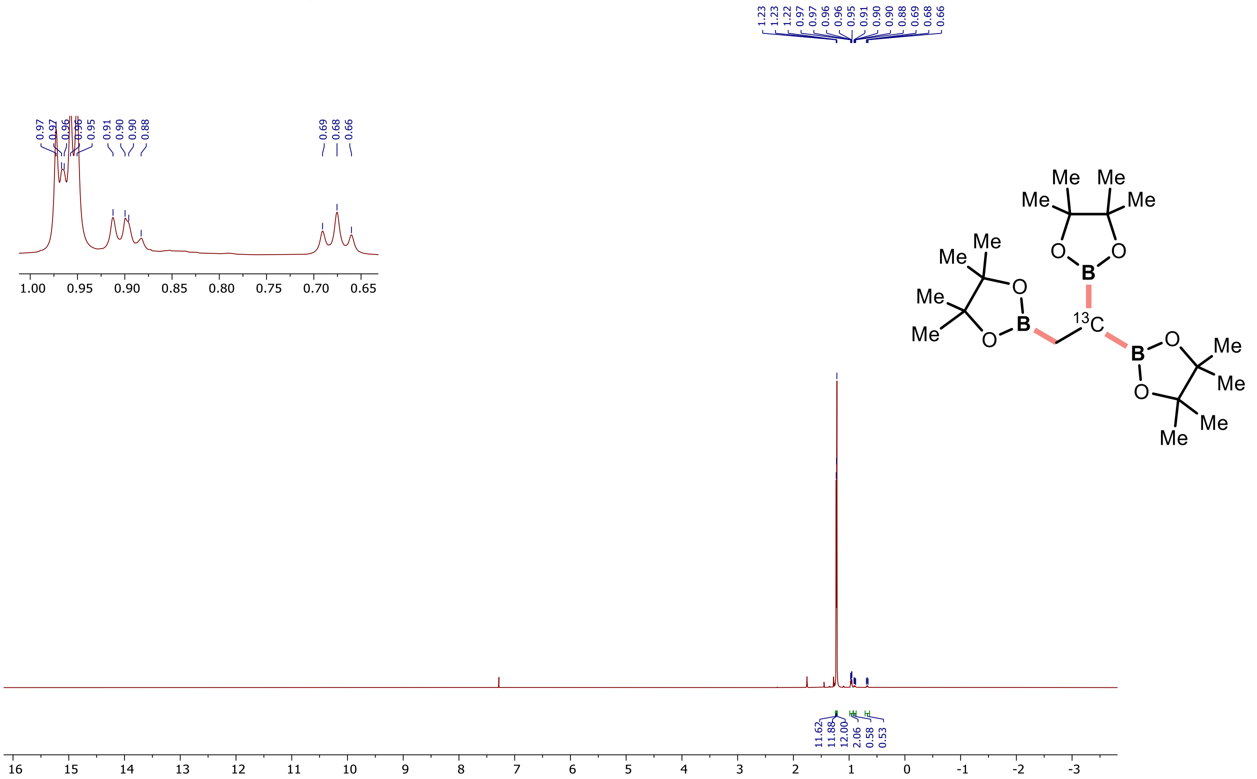

Supplementary Figure 35b-$^{13}C_1$-2 ^{13}C NMR (125 MHz, $CDCl_3$) 2,2',2''-(1λ^3-ethane-1,1,2-triyl-1-^{13}C)tris(4,4,5,5-tetramethyl-1,3,2-dioxaborolane)

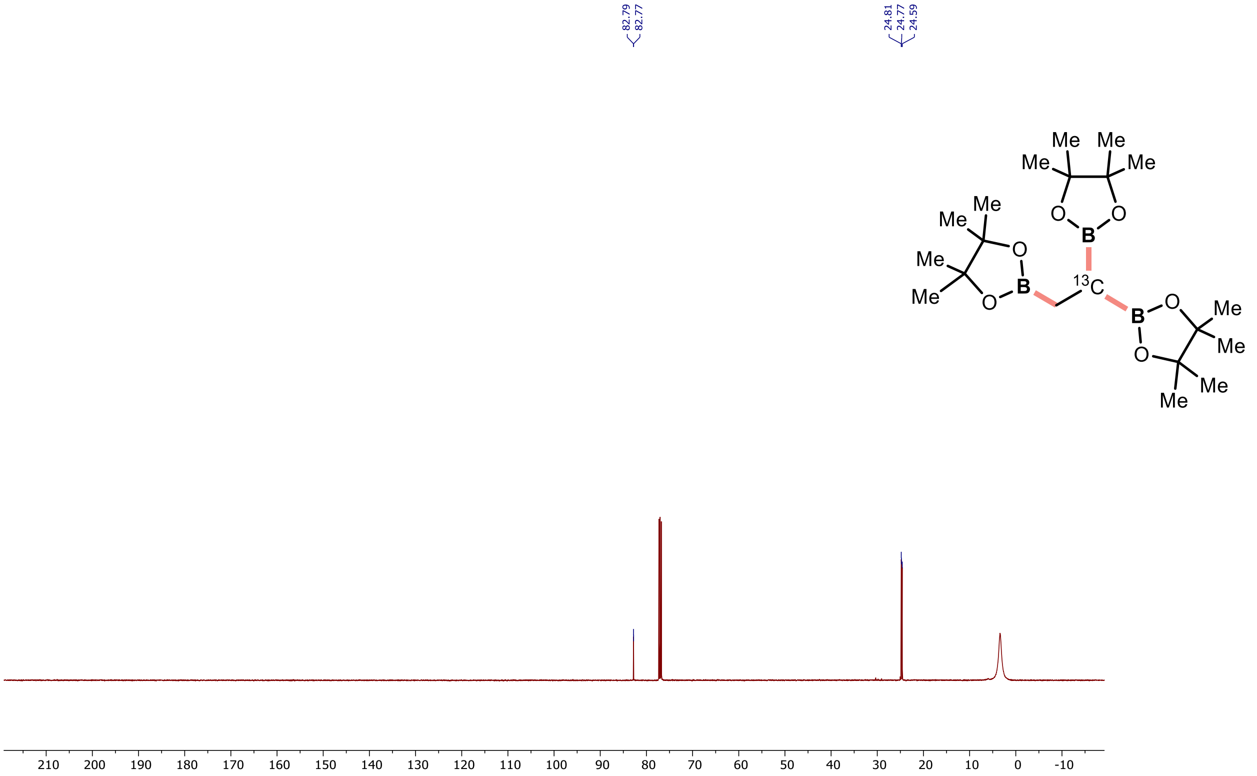

Supplementary Figure 35b-$^{13}C_1$-3 ^{11}B NMR (161 MHz, $CDCl_3$) 2,2',2''-(1λ^3-ethane-1,1,2-triyl-1-^{13}C)tris(4,4,5,5-tetramethyl-1,3,2-dioxaborolane)

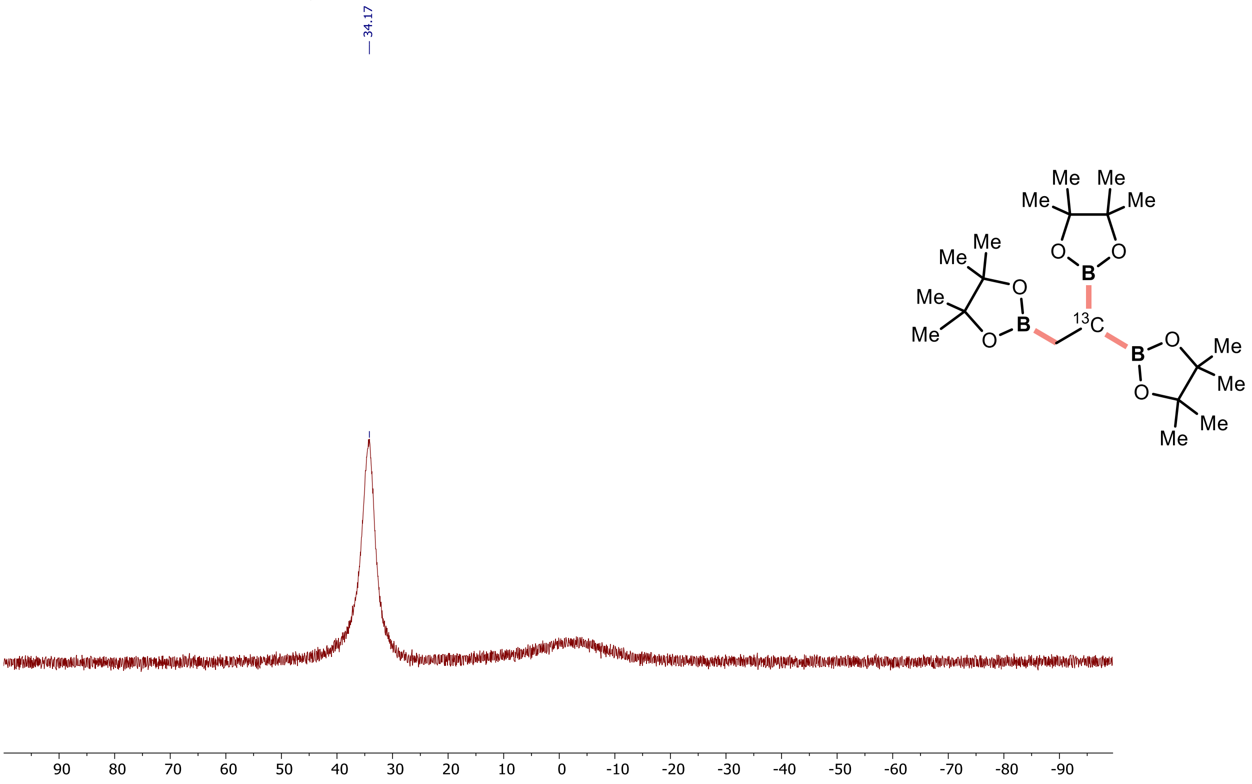

Supplementary Figure 35b-$^{13}C_2$-1 ^{1}H NMR (500 MHz, $CDCl_3$) 2,2',2''-(1λ^3,2λ^2-ethane-1,1,2-triyl-$^{13}C_2$)tris(4,4,5,5-tetramethyl-1,3,2-dioxaborolane)

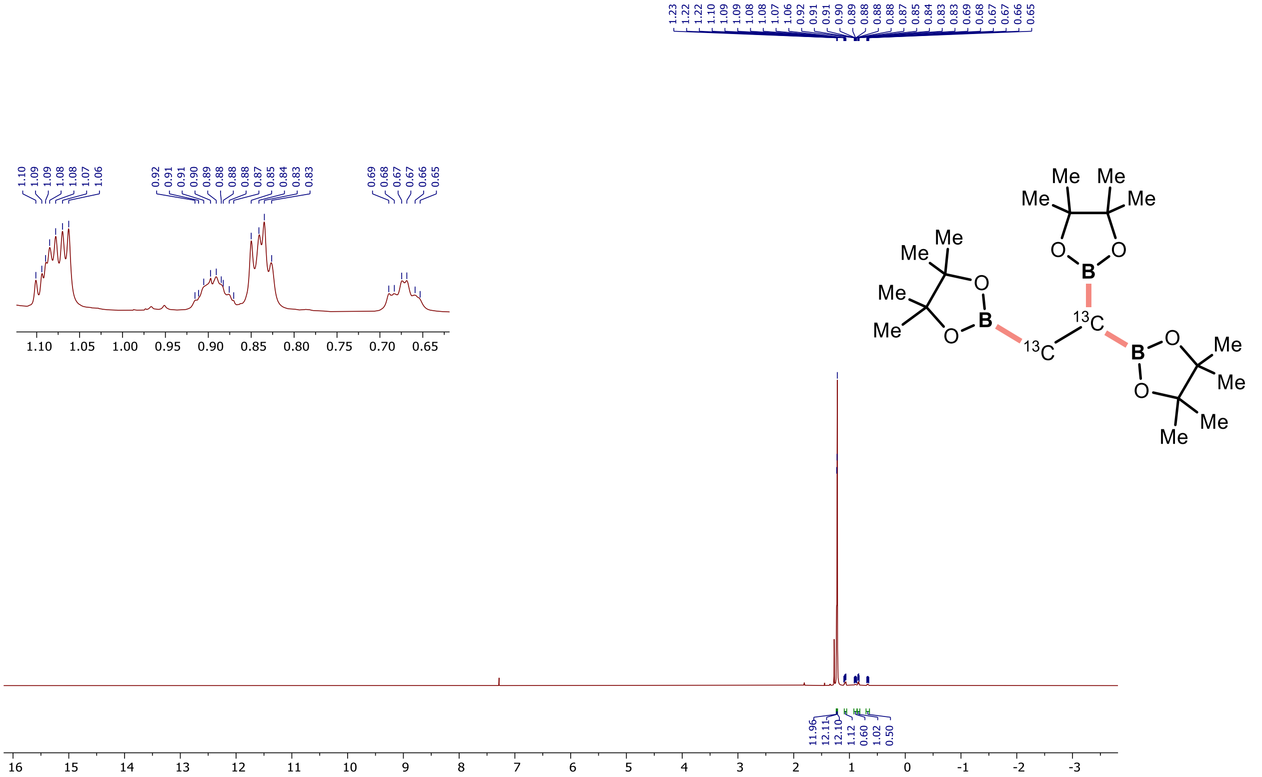

Supplementary Figure 35b-$^{13}C_2$-2 ^{13}C NMR (125 MHz, $CDCl_3$) 2,2',2''-(1λ^3,2λ^2-ethane-1,1,2-triyl-$^{13}C_2$)tris(4,4,5,5-tetramethyl-1,3,2-dioxaborolane)

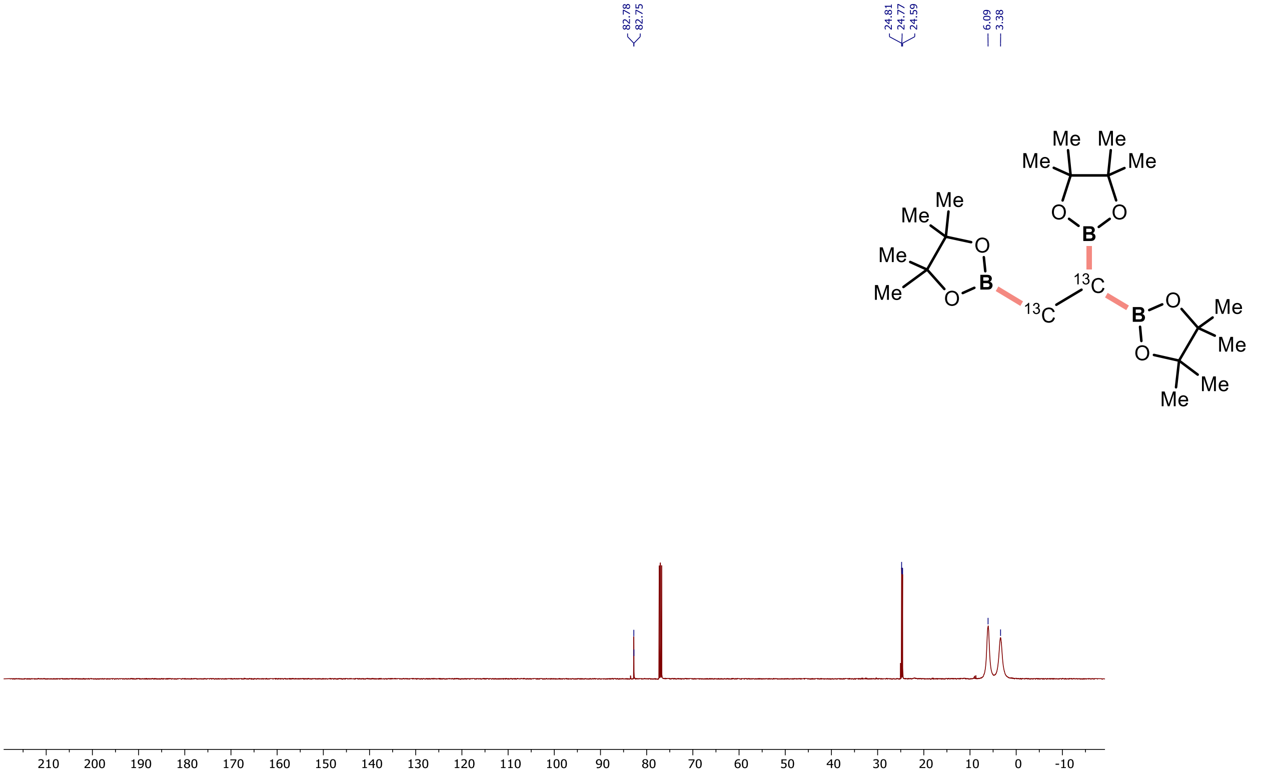

Supplementary Figure 35b-$^{13}C_2$-3 ^{11}B NMR (161 MHz, $CDCl_3$) 2,2',2''-(1λ^3,2λ^2-ethane-1,1,2-triyl-$^{13}C_2$)tris(4,4,5,5-tetramethyl-1,3,2-dioxaborolane)

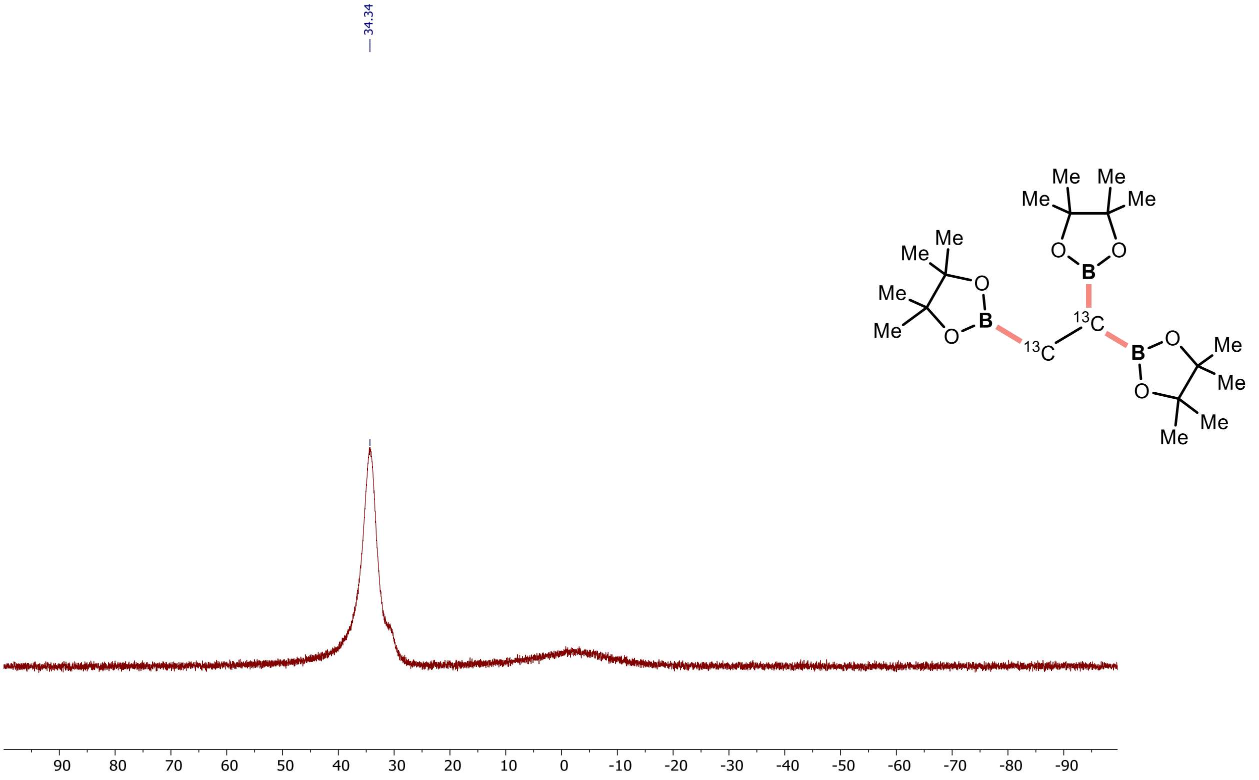

Supplementary Figure 36b-1 ^{1}H NMR (500 MHz, $CDCl_3$) 2,2',2''-(Butane-1,1,2-triyl)tris(4,4,5,5-tetramethyl-1,3,2-dioxaborolane)

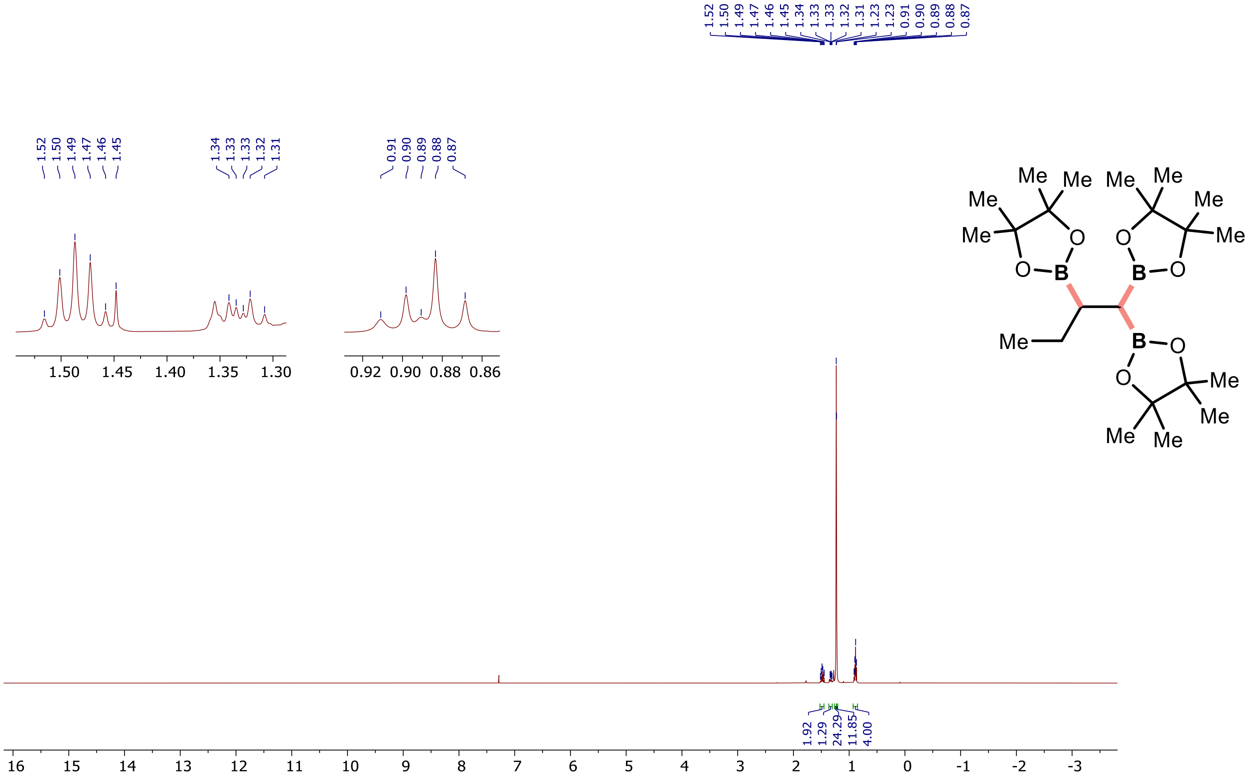

Supplementary Figure 36b-2 ^{13}C NMR (125 MHz, $CDCl_3$) 2,2',2''-(Butane-1,1,2-triyl)tris(4,4,5,5-tetramethyl-1,3,2-dioxaborolane)

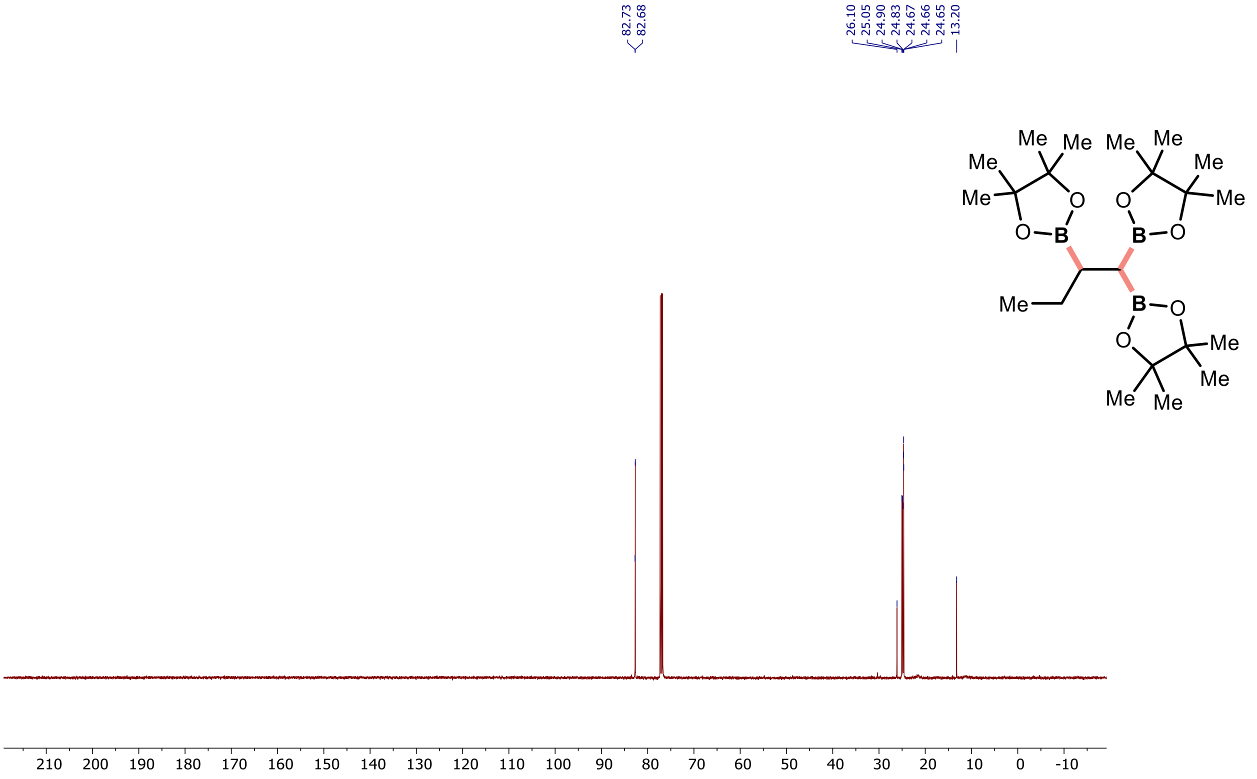

Supplementary Figure 36b-3 ^{11}B NMR (161 MHz, $CDCl_3$) 2,2',2''-(Butane-1,1,2-triyl)tris(4,4,5,5-tetramethyl-1,3,2-dioxaborolane)

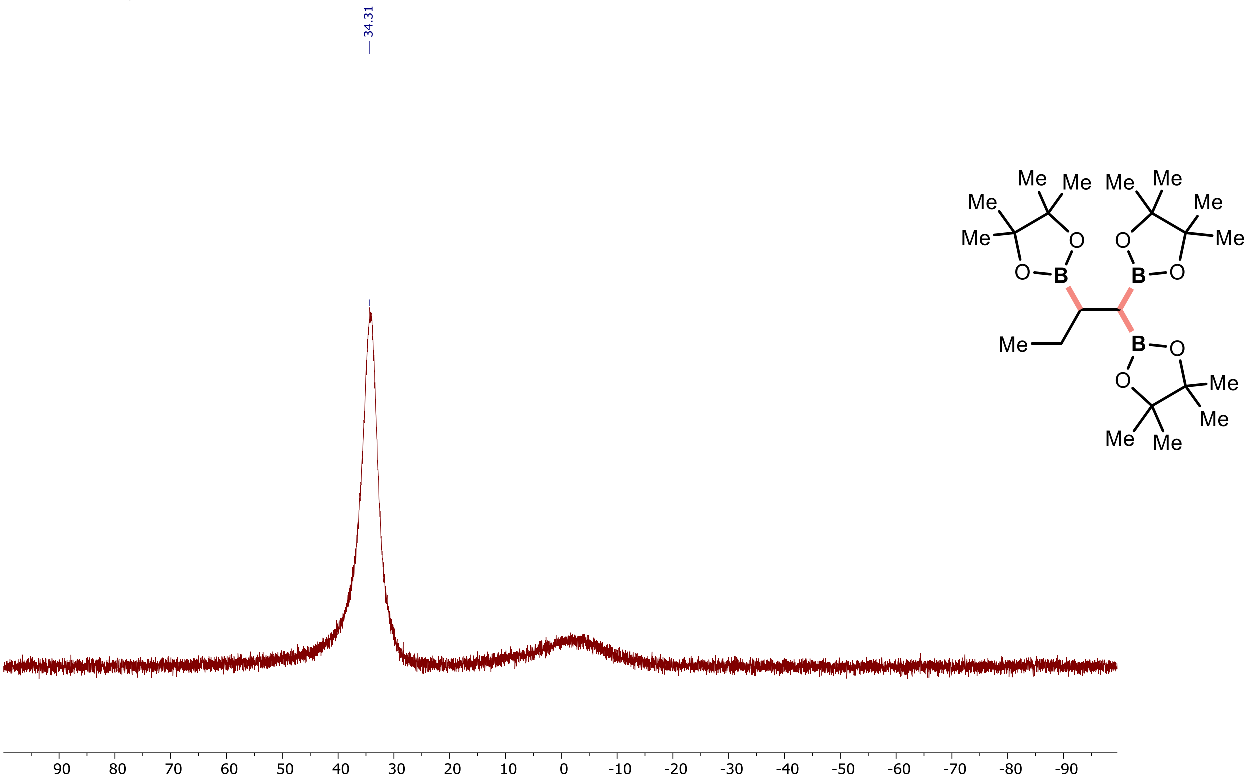

Supplementary Figure 37b-1 ^{1}H NMR (500 MHz, $CDCl_3$) 2,2',2''-(Octadecane-1,1,2-triyl)tris(4,4,5,5-tetramethyl-1,3,2-dioxaborolane)

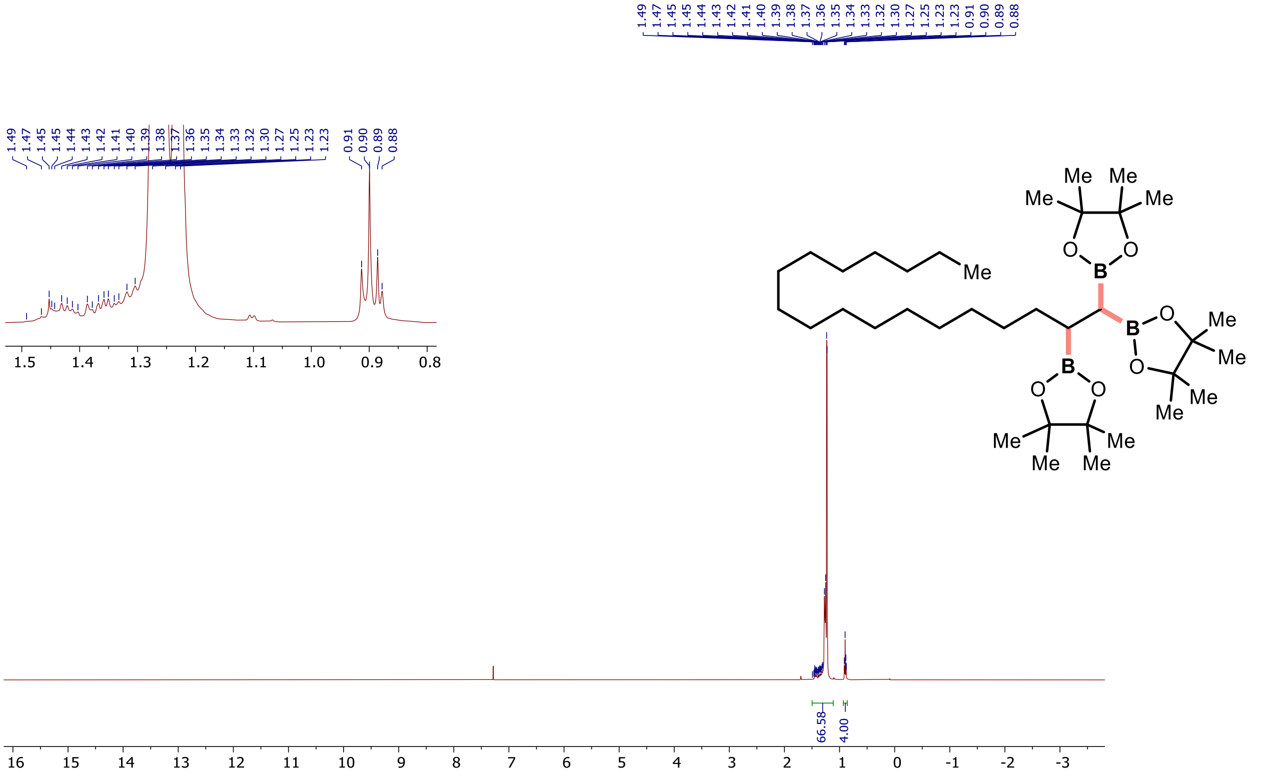

Supplementary Figure 37b-2 ^{13}C NMR (125 MHz, $CDCl_3$) 2,2',2''-(Octadecane-1,1,2-triyl)tris(4,4,5,5-tetramethyl-1,3,2-dioxaborolane)

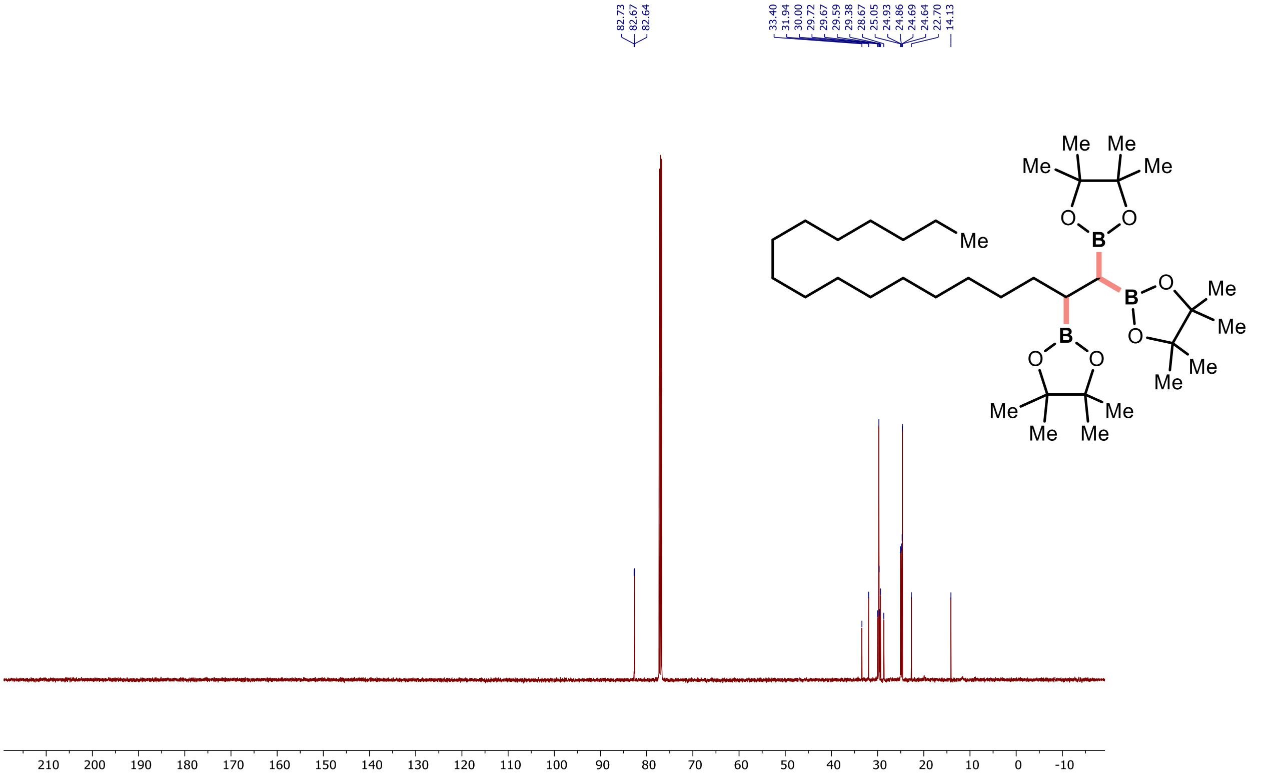

Supplementary Figure 37b-3 ^{11}B NMR (161 MHz, $CDCl_3$) 2,2',2''-(Octadecane-1,1,2-triyl)tris(4,4,5,5-tetramethyl-1,3,2-dioxaborolane)

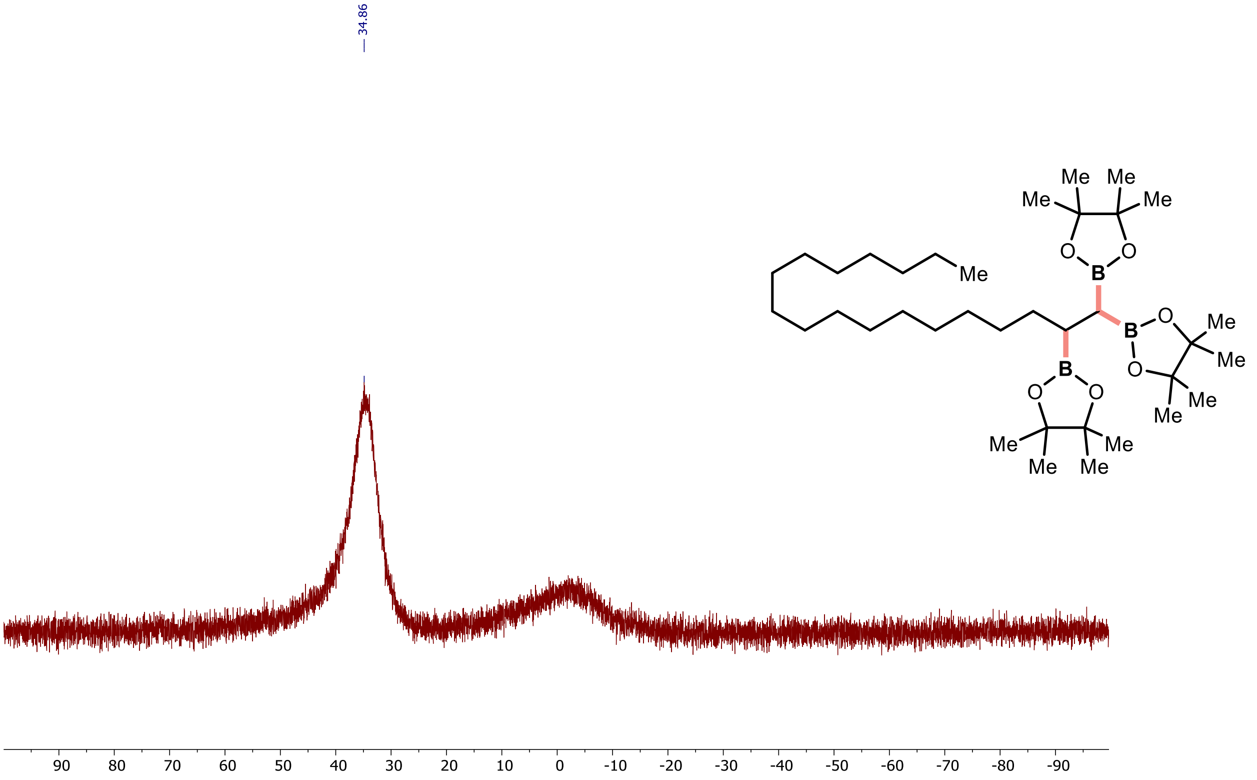

Supplementary Figure 37b-*d*-1 ^{1}H NMR (500 MHz, $CDCl_3$) 2,2',2''-(Octadecane-1,1,2-triyl-3,3,4,4,5,5,6,6,7,7,8,8,9,9,10,10,11,11,12,12,13,13,14,14,15,15,16,16,17,17,18,18,18-d_{33})tris(4,4,5,5-tetramethyl-1,3,2-dioxaborolane) (37b-*d*)

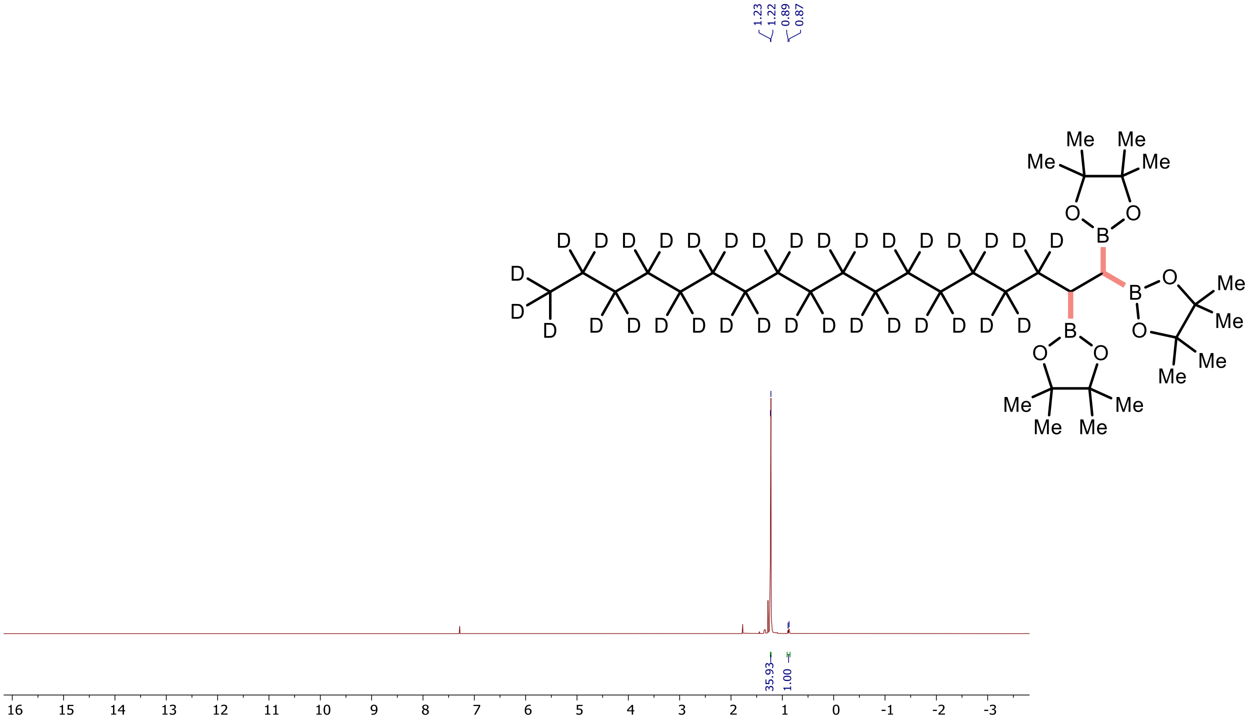

Supplementary Figure 37b-*d*-2 ^{13}C NMR (200 MHz, $CDCl_3$) 2,2',2''-(Octadecane-1,1,2-triyl-3,3,4,4,5,5,6,6,7,7,8,8,9,9,10,10,11,11,12,12,13,13,14,14,15,15,16,16,17,17,18,18,18-d_{33})tris(4,4,5,5-tetramethyl-1,3,2-dioxaborolane) (37b-*d*)

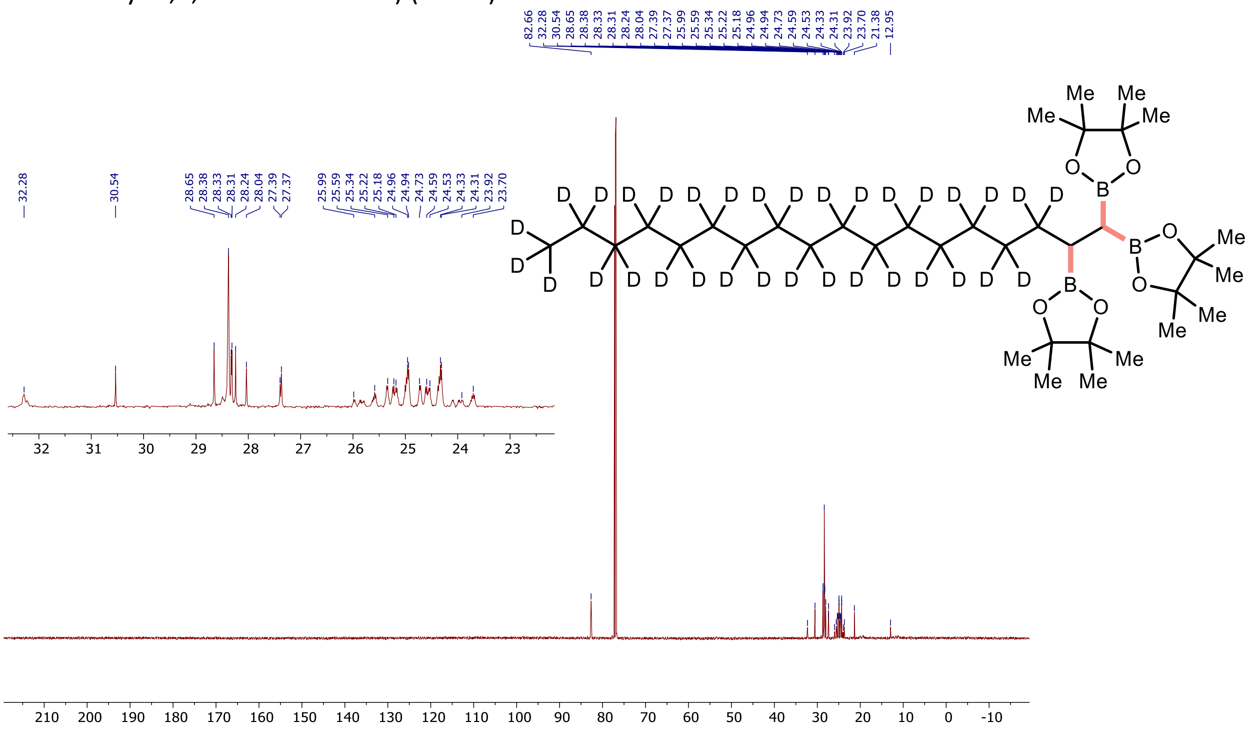

Supplementary Figure 35b-*d*-3 ^{11}B NMR (161 MHz, $CDCl_3$) 2,2',2''-(Octadecane-1,1,2-triyl-3,3,4,4,5,5,6,6,7,7,8,8,9,9,10,10,11,11,12,12,13,13,14,14,15,15,16,16,17,17,18,18,18-*d*$_{33}$)tris(4,4,5,5-tetramethyl-1,3,2-dioxaborolane) (37b-*d*)

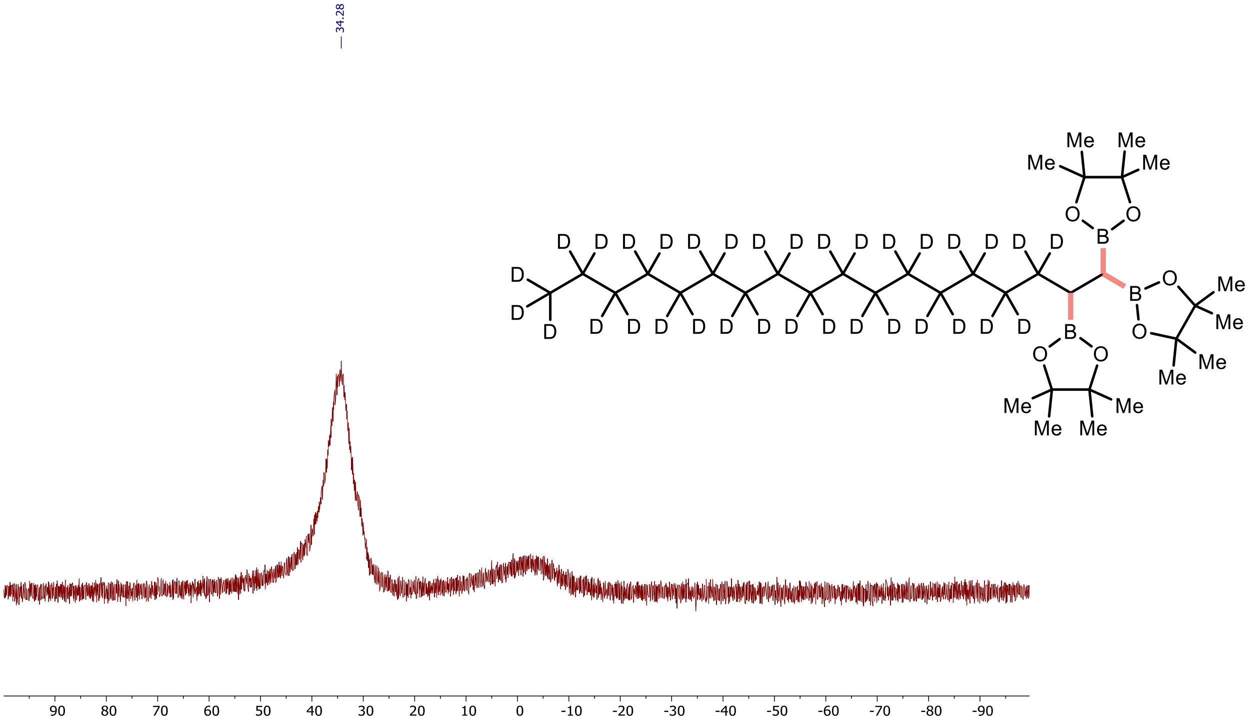

Supplementary Figure 35b-*d*-4 ^{2}H NMR (77 MHz, $CDCl_3$) 2,2',2''-(Octadecane-1,1,2-triyl-3,3,4,4,5,5,6,6,7,7,8,8,9,9,10,10,11,11,12,12,13,13,14,14,15,15,16,16,17,17,18,18,18-*d*$_{33}$)tris(4,4,5,5-tetramethyl-1,3,2-dioxaborolane) (37b-*d*)

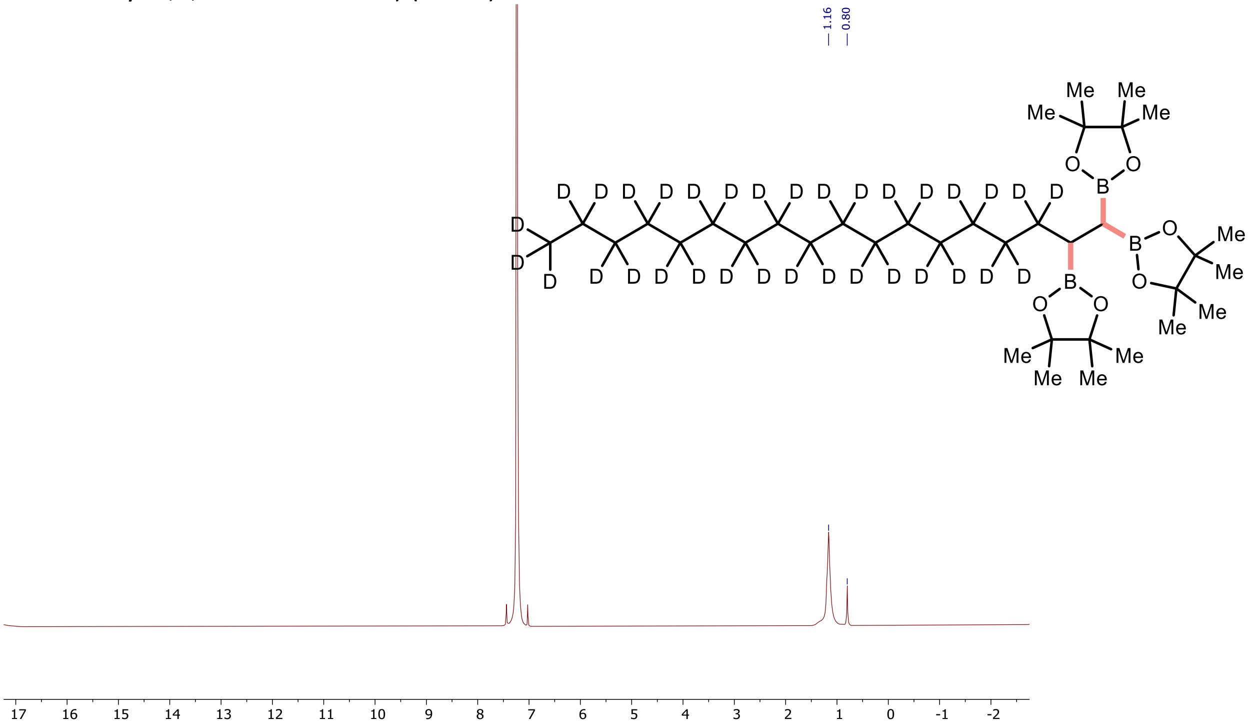

Supplementary Figure 37b-^{13}C-1 ^{1}H NMR (500 MHz, $CDCl_3$) 2,2',2''-(Octadecane-1,1,2-triyl-1-^{13}C)tris(4,4,5,5-tetramethyl-1,3,2-dioxaborolane)

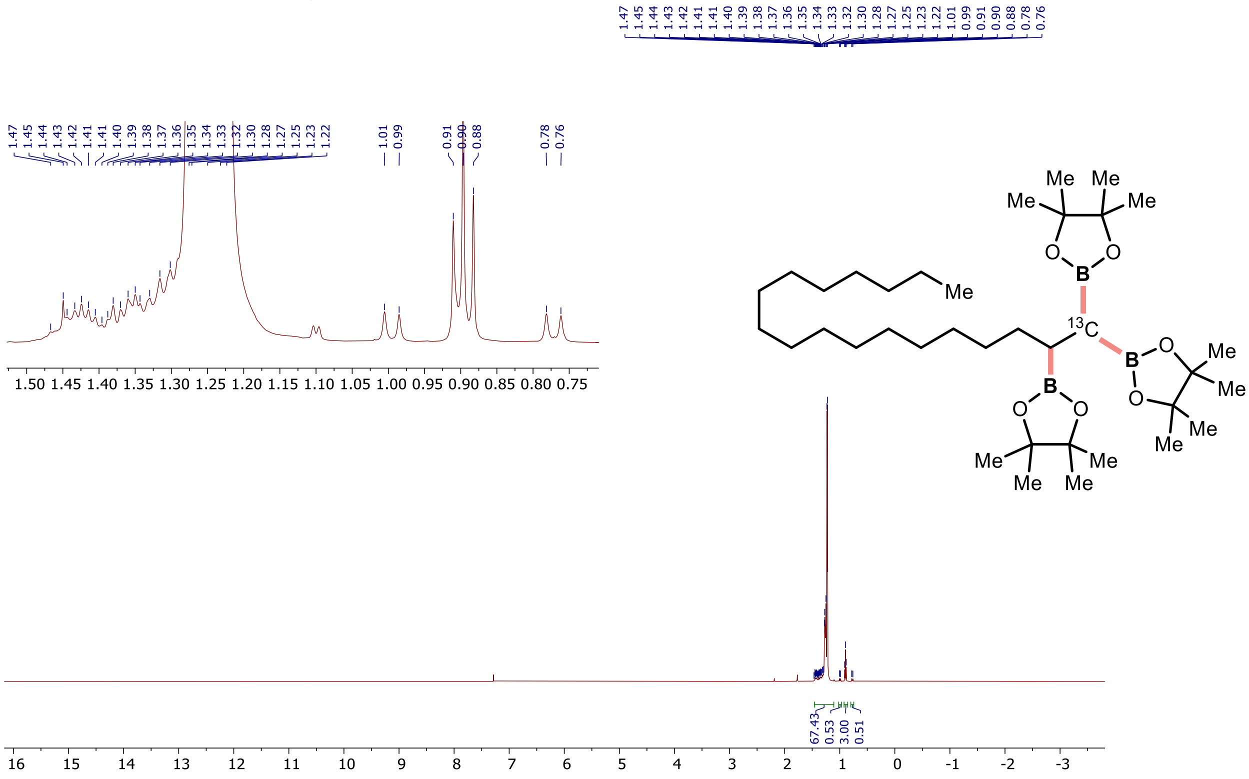

Supplementary Figure 37b-^{13}C-2 ^{13}C NMR (125 MHz, $CDCl_3$) 2,2',2''-(Octadecane-1,1,2-triyl-1-^{13}C)tris(4,4,5,5-tetramethyl-1,3,2-dioxaborolane)

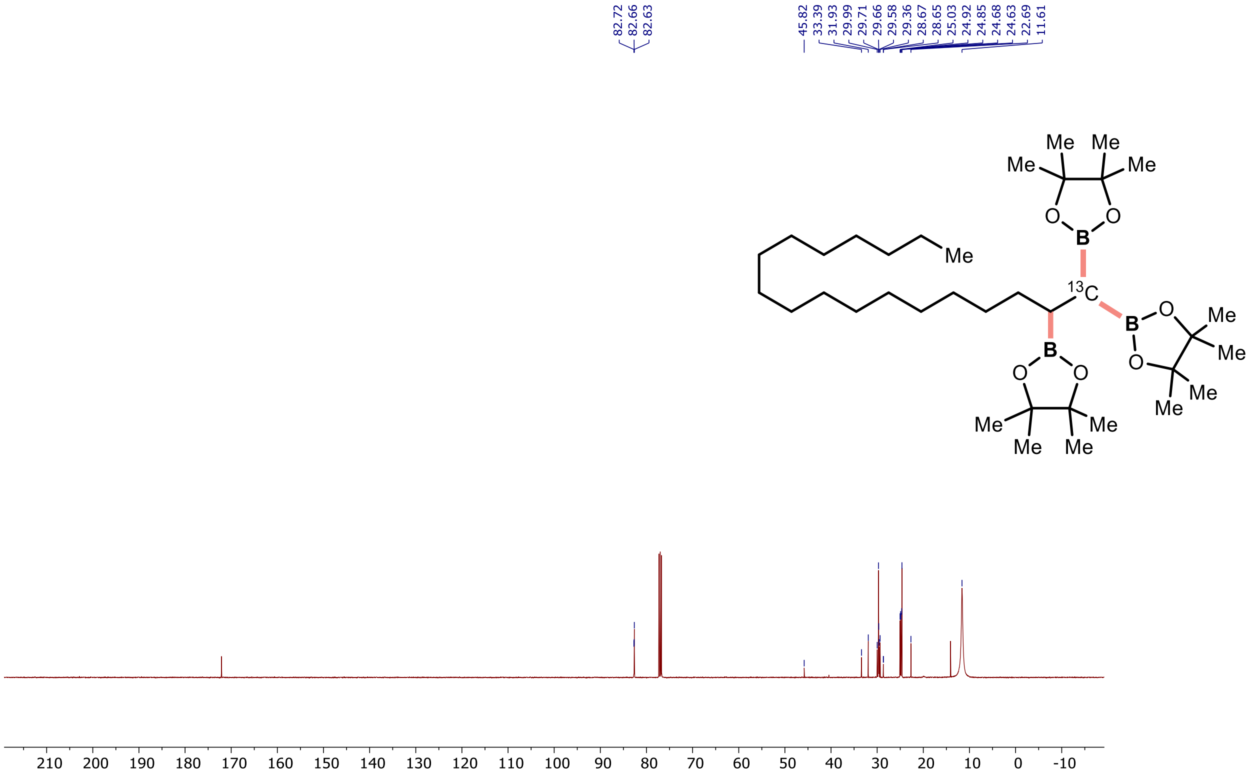

Supplementary Figure 37b-^{13}C-3 ^{11}B NMR (161 MHz, $CDCl_3$) 2,2',2''-(Octadecane-1,1,2-triyl-1-^{13}C)tris(4,4,5,5-tetramethyl-1,3,2-dioxaborolane)

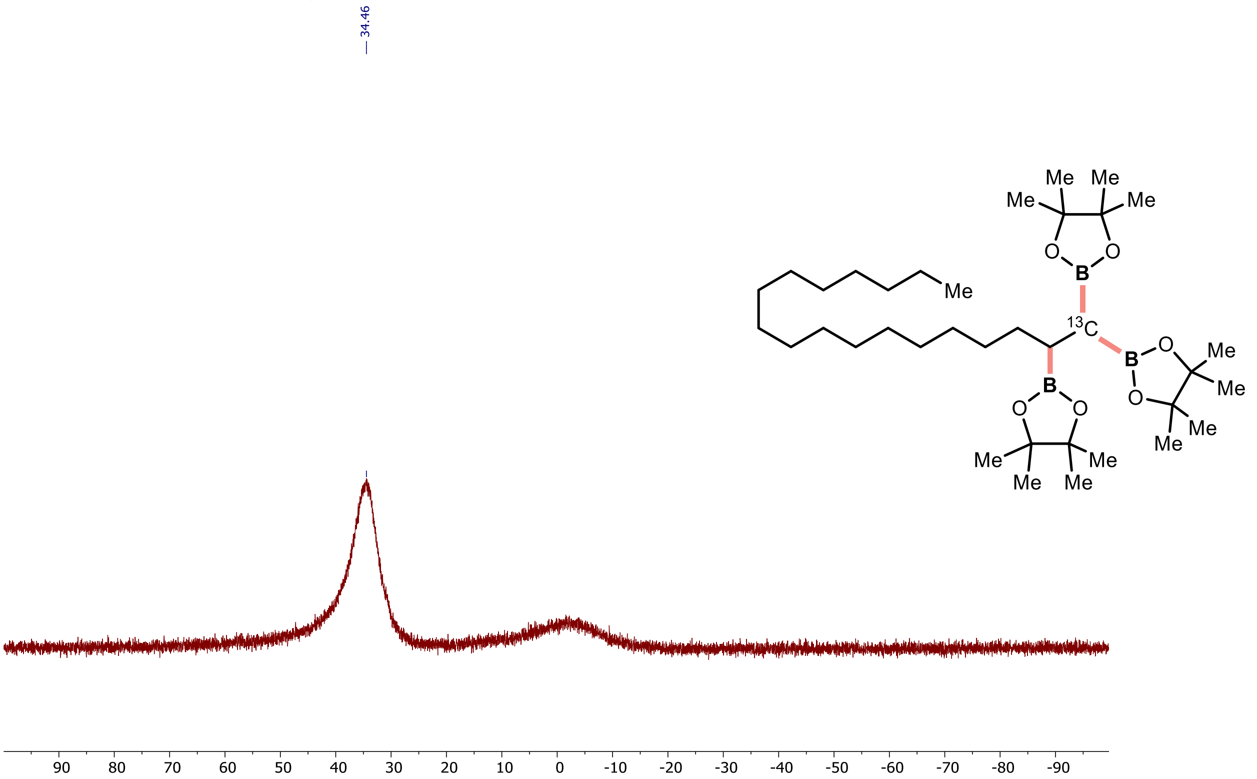

Supplementary Figure 38b-1 ^{1}H NMR (500 MHz, $CDCl_3$) 2,2',2''-(2-Phenylethane-1,1,2-triyl)tris(4,4,5,5-tetramethyl-1,3,2-dioxaborolane)

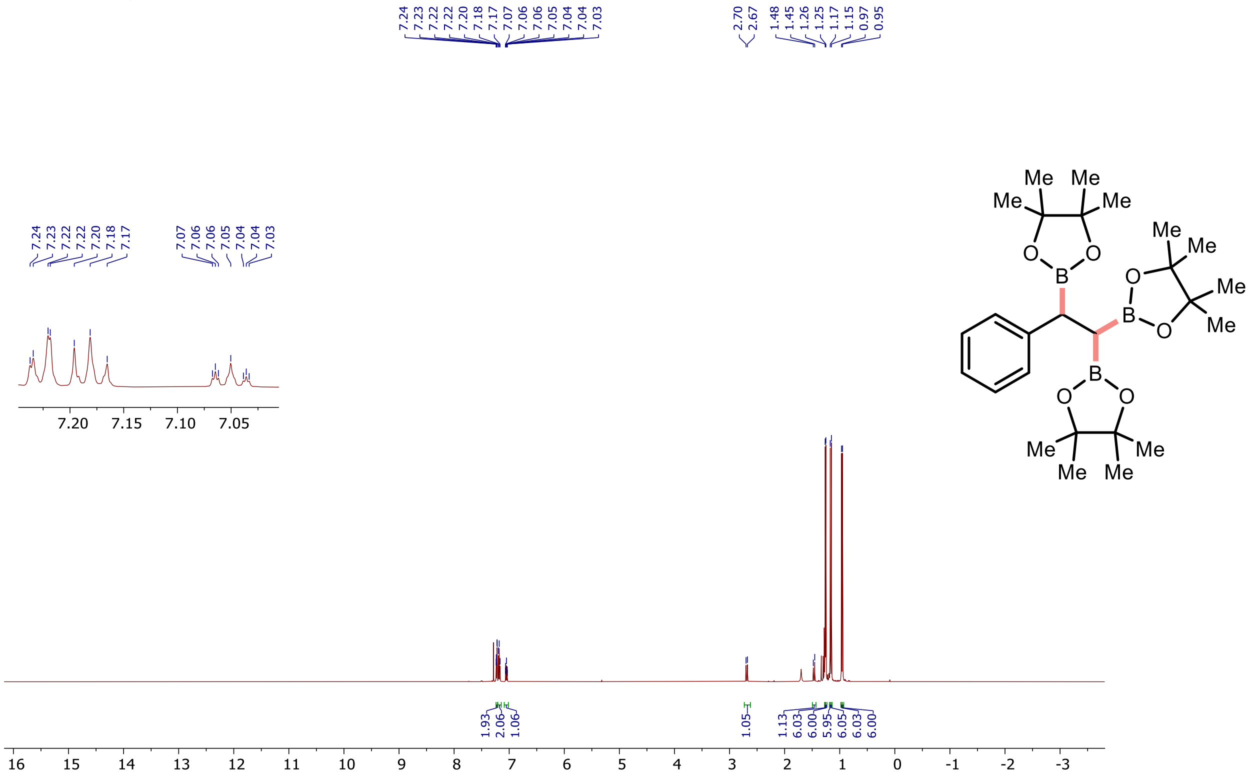

Supplementary Figure 38b-2 ^{13}C NMR (125 MHz, $CDCl_3$) 2,2',2''-(2-Phenylethane-1,1,2-triyl)tris(4,4,5,5-tetramethyl-1,3,2-dioxaborolane)

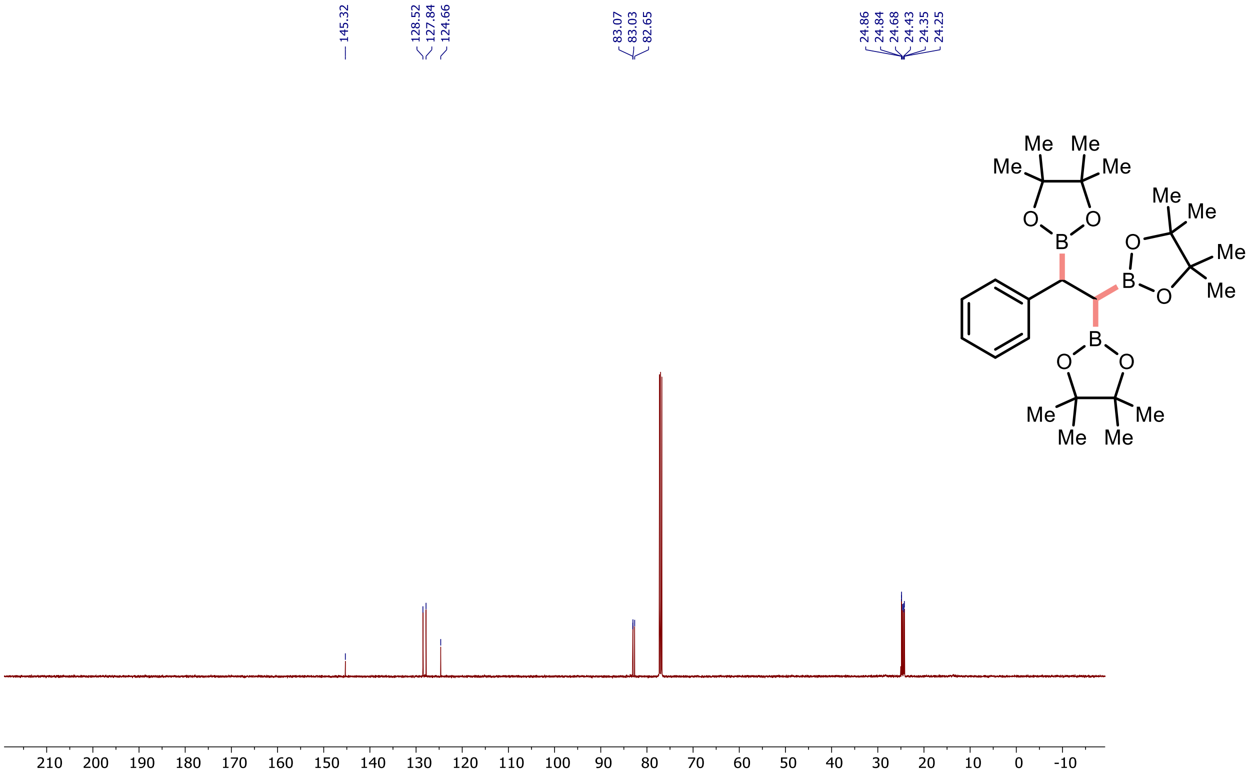

Supplementary Figure 38b-3 ^{11}B NMR (161 MHz, $CDCl_3$) 2,2',2''-(2-Phenylethane-1,1,2-triyl)tris(4,4,5,5-tetramethyl-1,3,2-dioxaborolane)

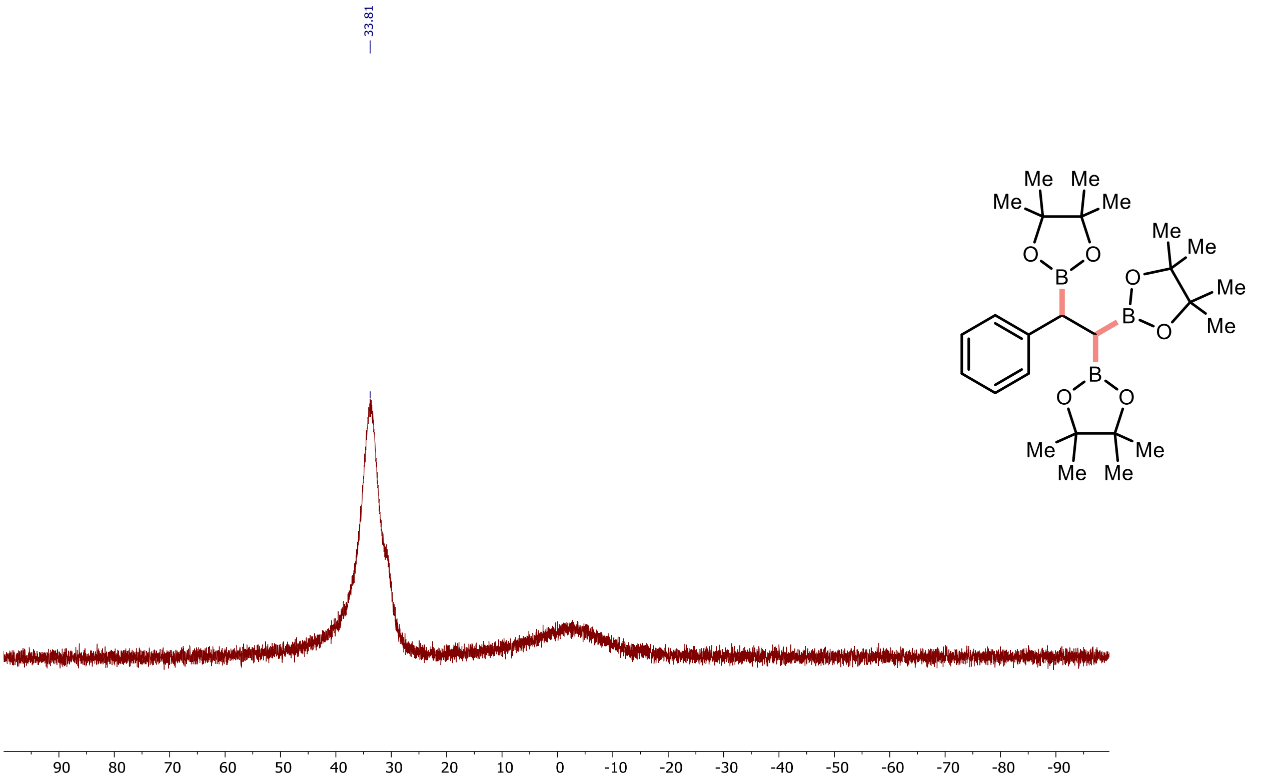

Supplementary Figure 38b'-H$_2$-1 ^{1}H NMR (500 MHz, $CDCl_3$) 4,4,5,5-Tetramethyl-2-phenethyl-1,3,2-dioxaborolane

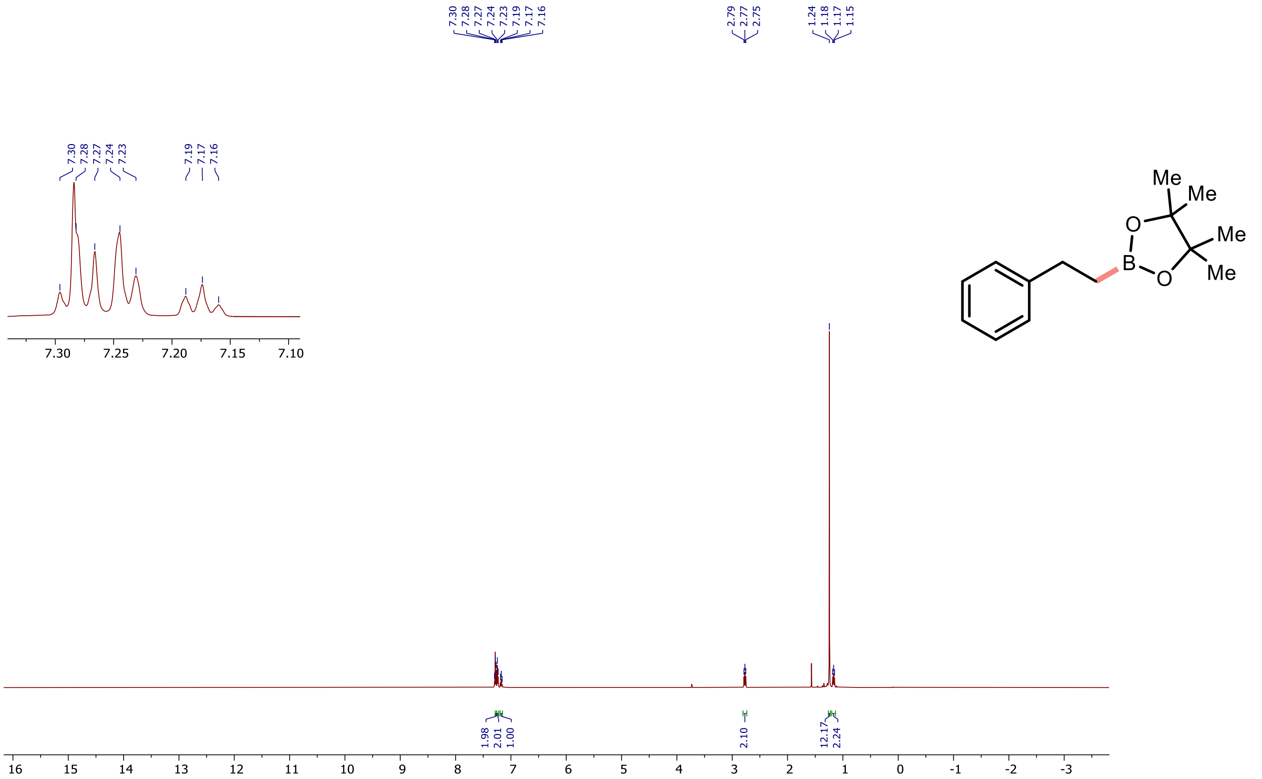

Supplementary Figure 38b'-H$_2$-2 ^{13}C NMR (125 MHz, $CDCl_3$) 4,4,5,5-Tetramethyl-2-phenethyl-1,3,2-dioxaborolane

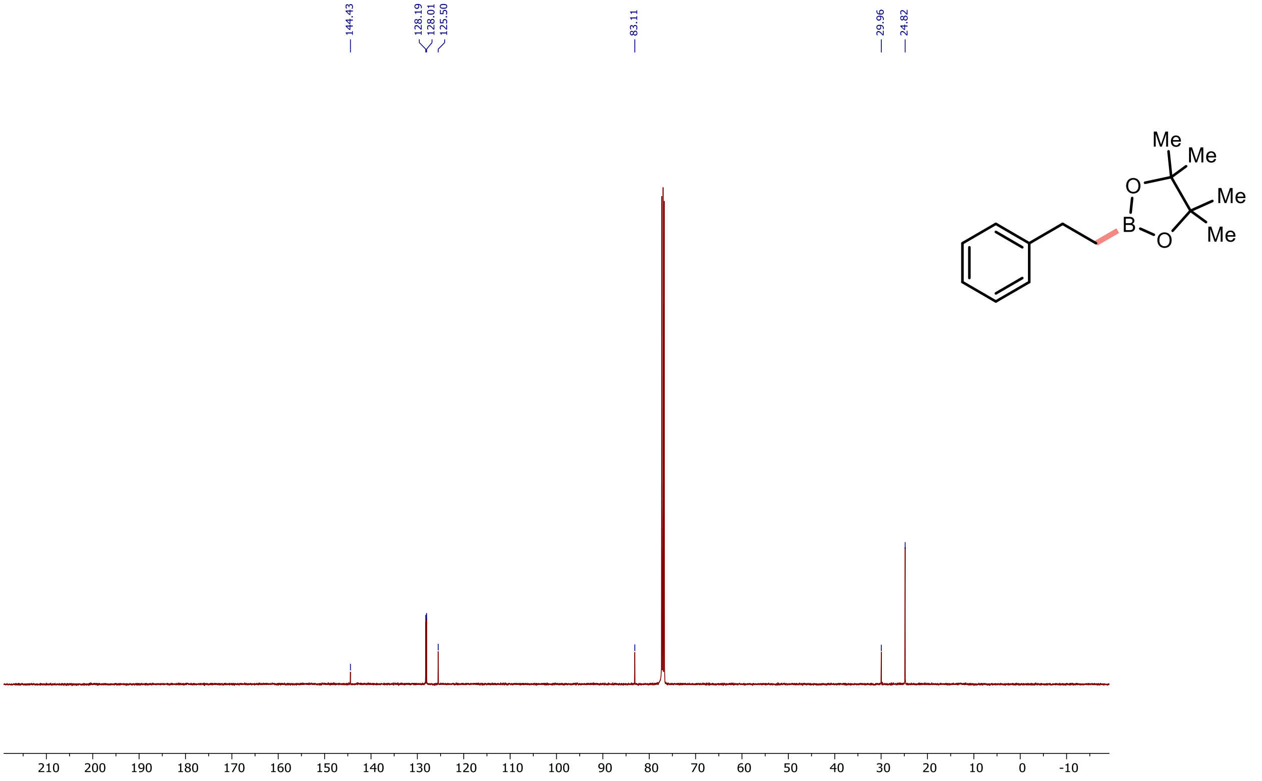

Supplementary Figure 38b'-H$_2$-3 ^{11}B NMR (161 MHz, $CDCl_3$) 4,4,5,5-Tetramethyl-2-phenethyl-1,3,2-dioxaborolane

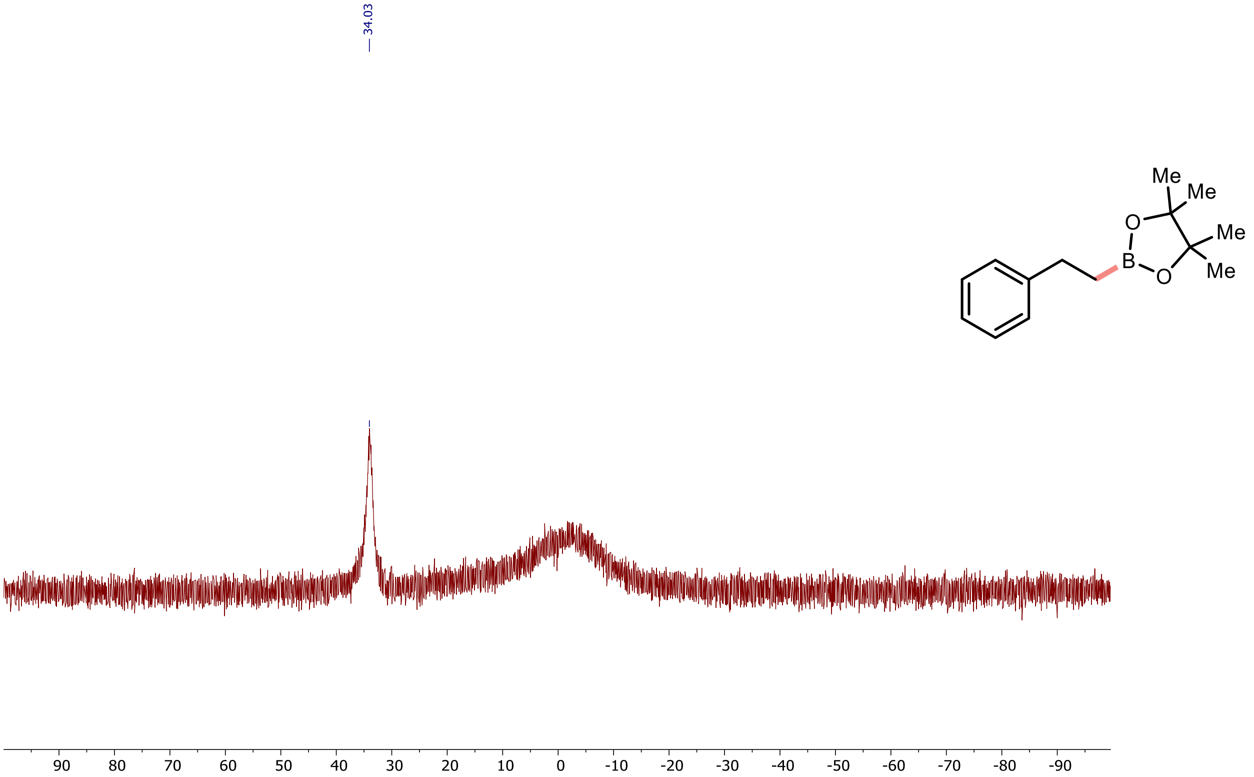

Supplementary Figure 39b-1 ^{1}H NMR (500 MHz, $CDCl_3$) 2,2',2''-(3-Phenylpropane-1,1,2-triyl)tris(4,4,5,5-tetramethyl-1,3,2-dioxaborolane)

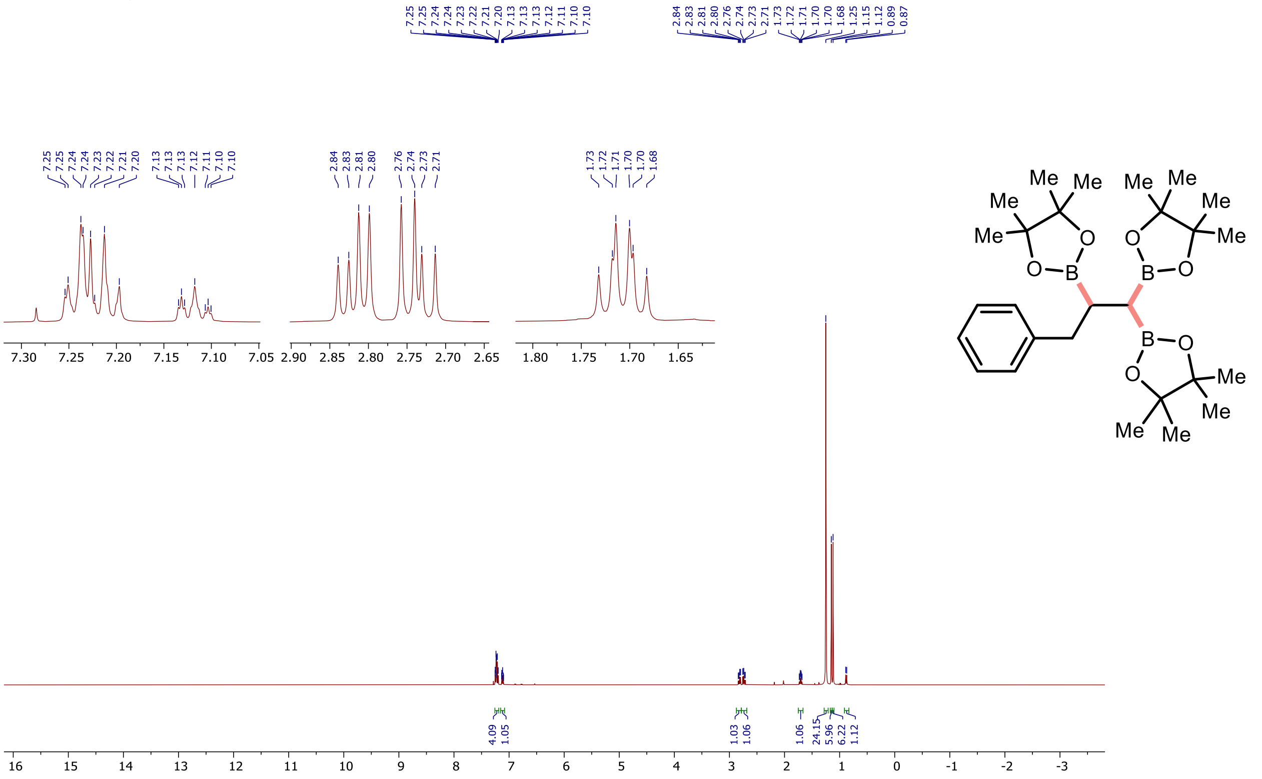

Supplementary Figure 39b-2 ^{13}C NMR (125 MHz, $CDCl_3$) 2,2',2''-(3-Phenylpropane-1,1,2-triyl)tris(4,4,5,5-tetramethyl-1,3,2-dioxaborolane)

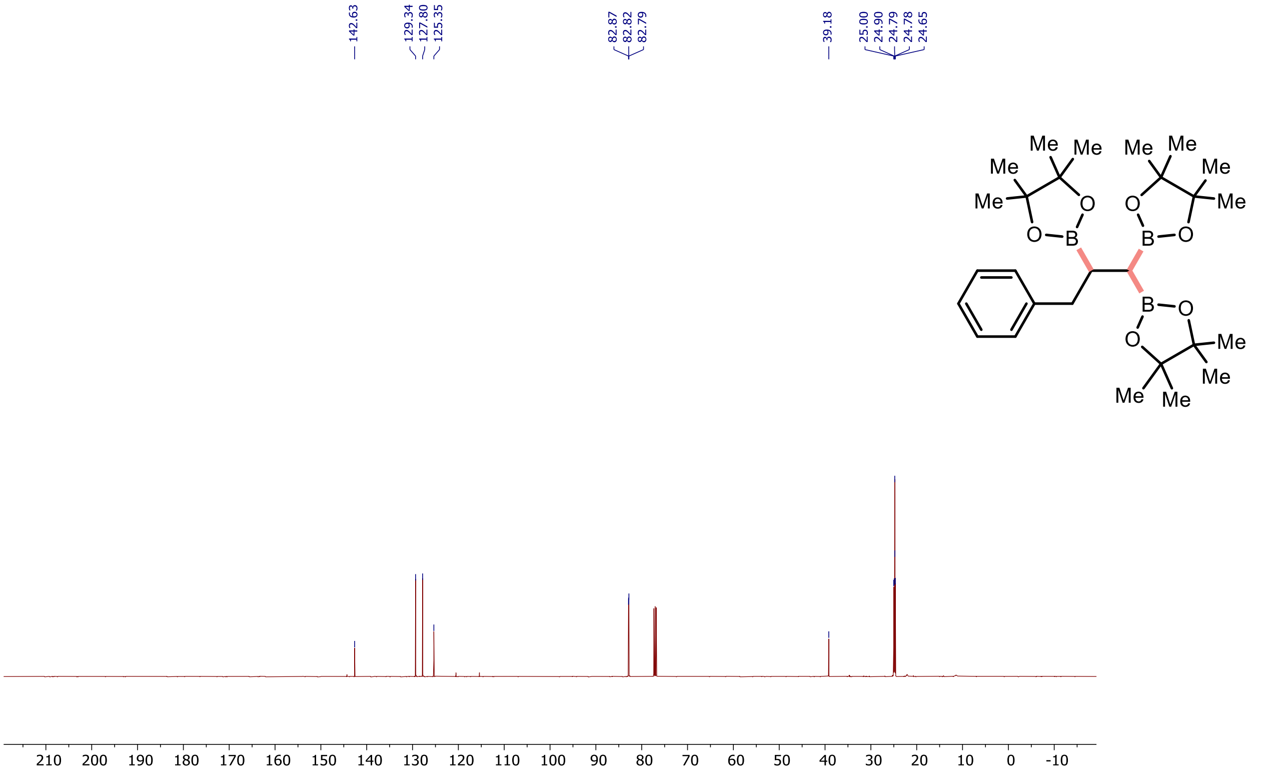

Supplementary Figure 39b-3 ^{11}B NMR (161 MHz, $CDCl_3$) 2,2',2''-(3-Phenylpropane-1,1,2-triyl)tris(4,4,5,5-tetramethyl-1,3,2-dioxaborolane)

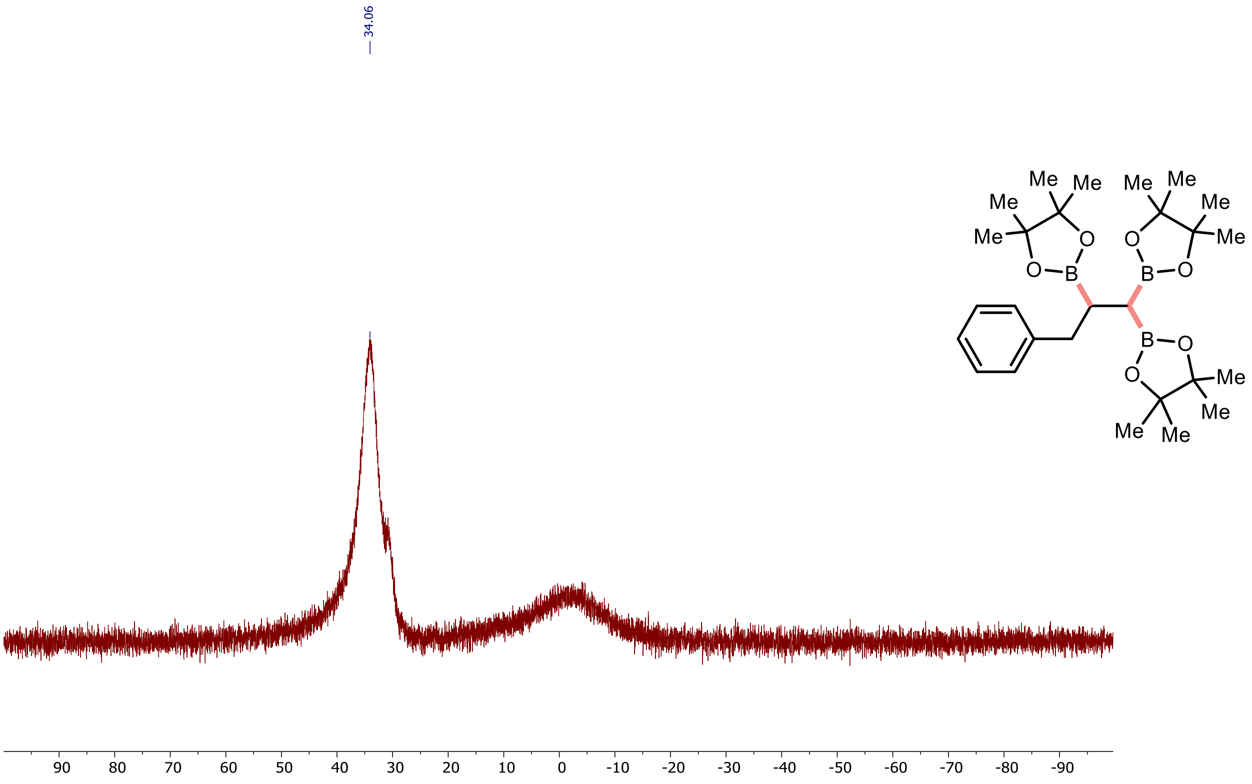

Supplementary Figure 39b-^{13}C-1 ^{1}H NMR (500 MHz, $CDCl_3$) 2,2',2''-(3-Phenylpropane-1,1,2-triyl-2-^{13}*C*)tris(4,4,5,5-tetramethyl-1,3,2-dioxaborolane)

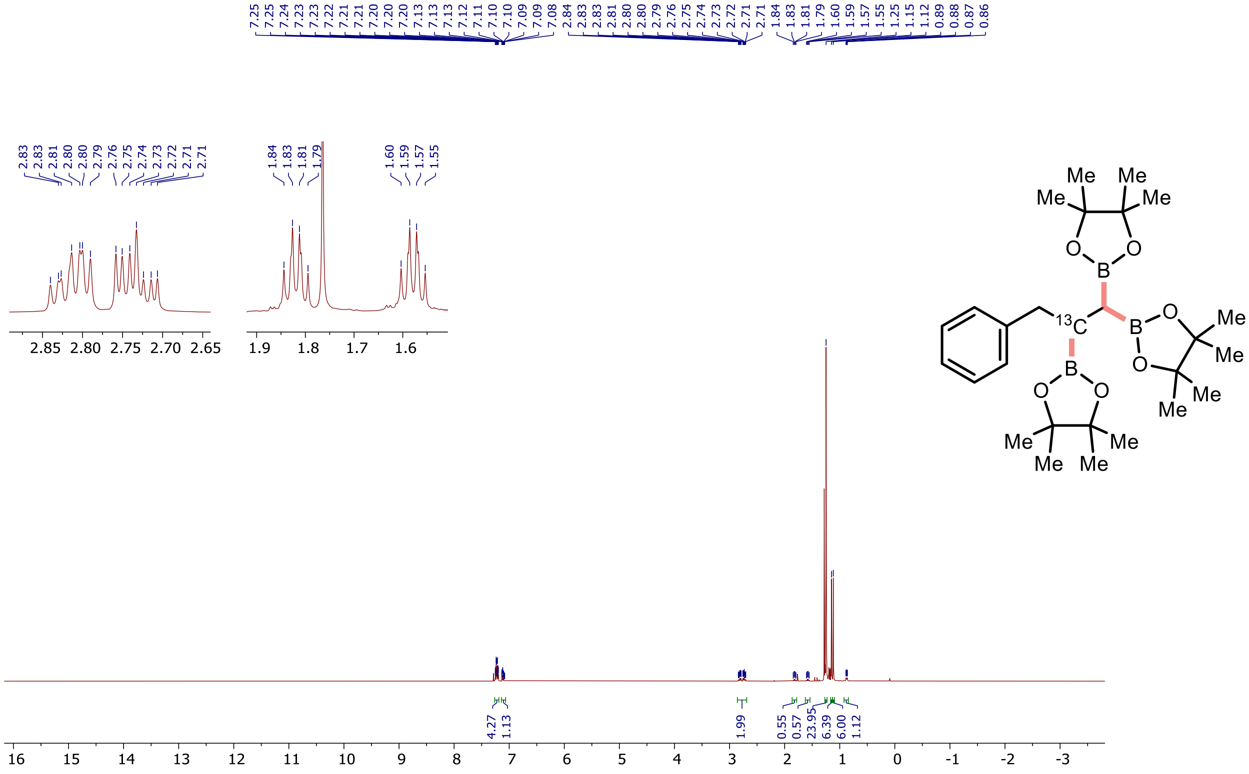

Supplementary Figure 39b-^{13}C-2 ^{13}C NMR (125 MHz, $CDCl_3$) 2,2',2''-(3-Phenylpropane-1,1,2-triyl-2-^{13}*C*)tris(4,4,5,5-tetramethyl-1,3,2-dioxaborolane)

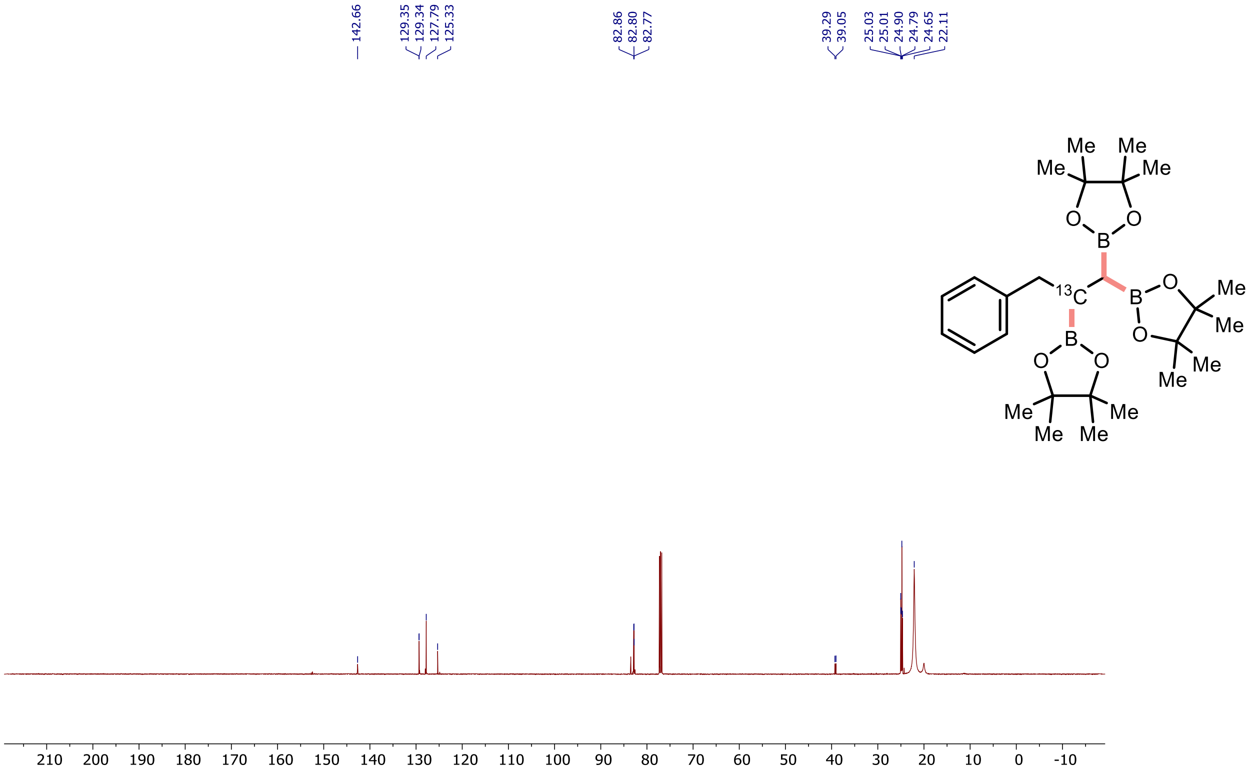

Supplementary Figure 39b-^{13}C-3 ^{11}B NMR (161 MHz, $CDCl_3$) 2,2',2''-(3-Phenylpropane-1,1,2-triyl-2-^{13}*C*)tris(4,4,5,5-tetramethyl-1,3,2-dioxaborolane)

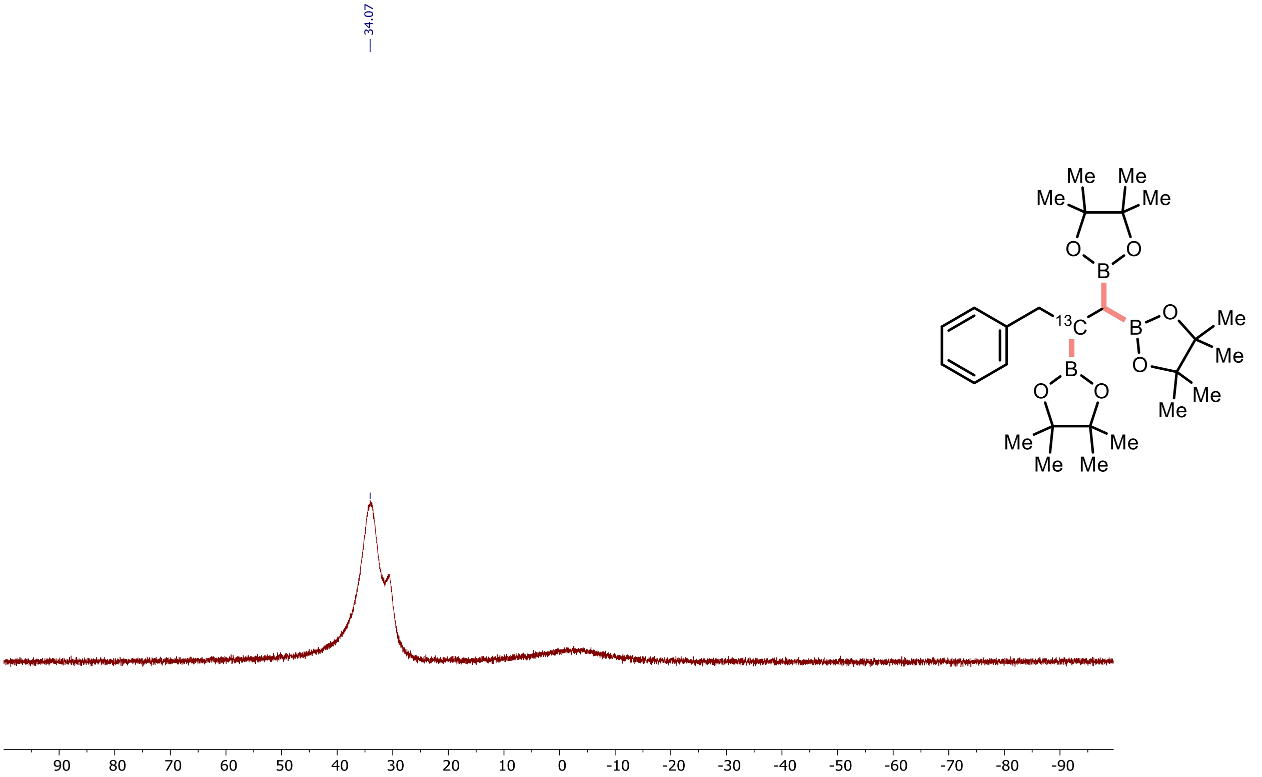

Supplementary Figure 40b-1 ^{1}H NMR (500 MHz, $CDCl_3$) 2,2',2''-(4-Phenylbutane-1,1,2-triyl)tris(4,4,5,5-tetramethyl-1,3,2-dioxaborolane)

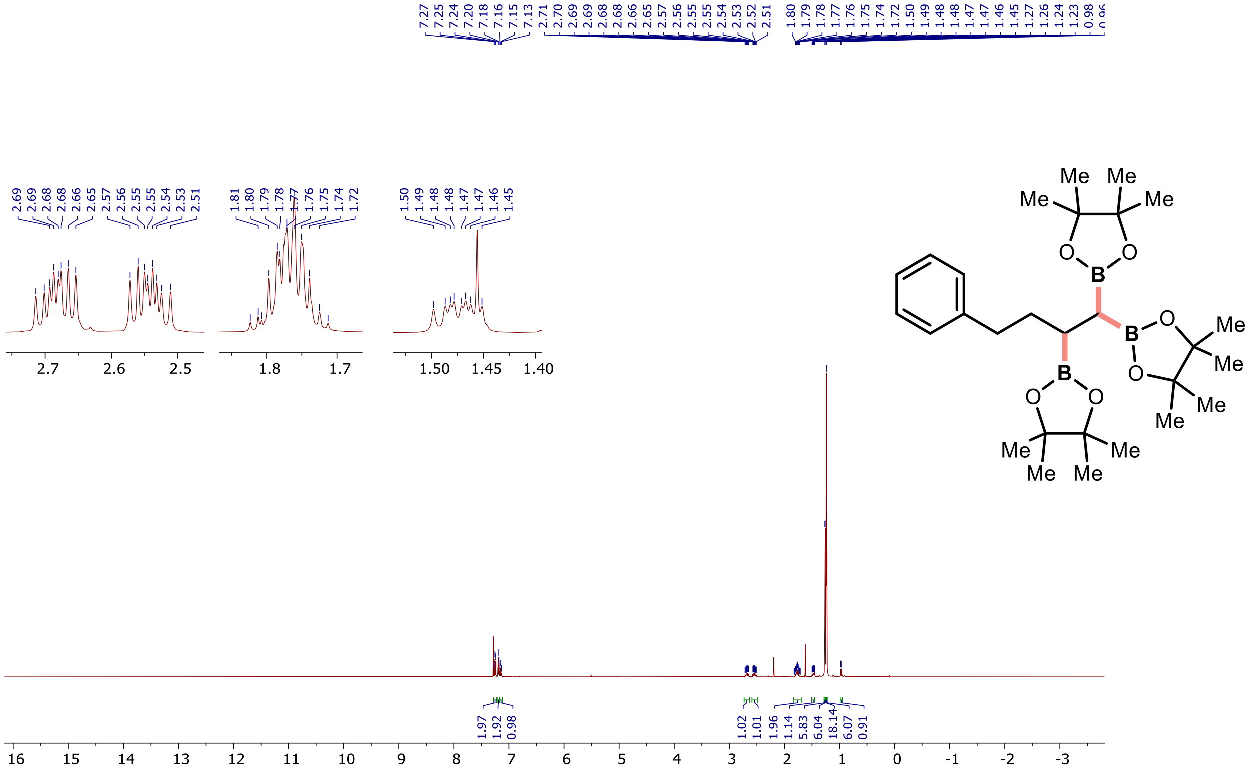

Supplementary Figure 40b-2 ^{13}C NMR (125 MHz, $CDCl_3$) 2,2',2''-(4-Phenylbutane-1,1,2-triyl)tris(4,4,5,5-tetramethyl-1,3,2-dioxaborolane)

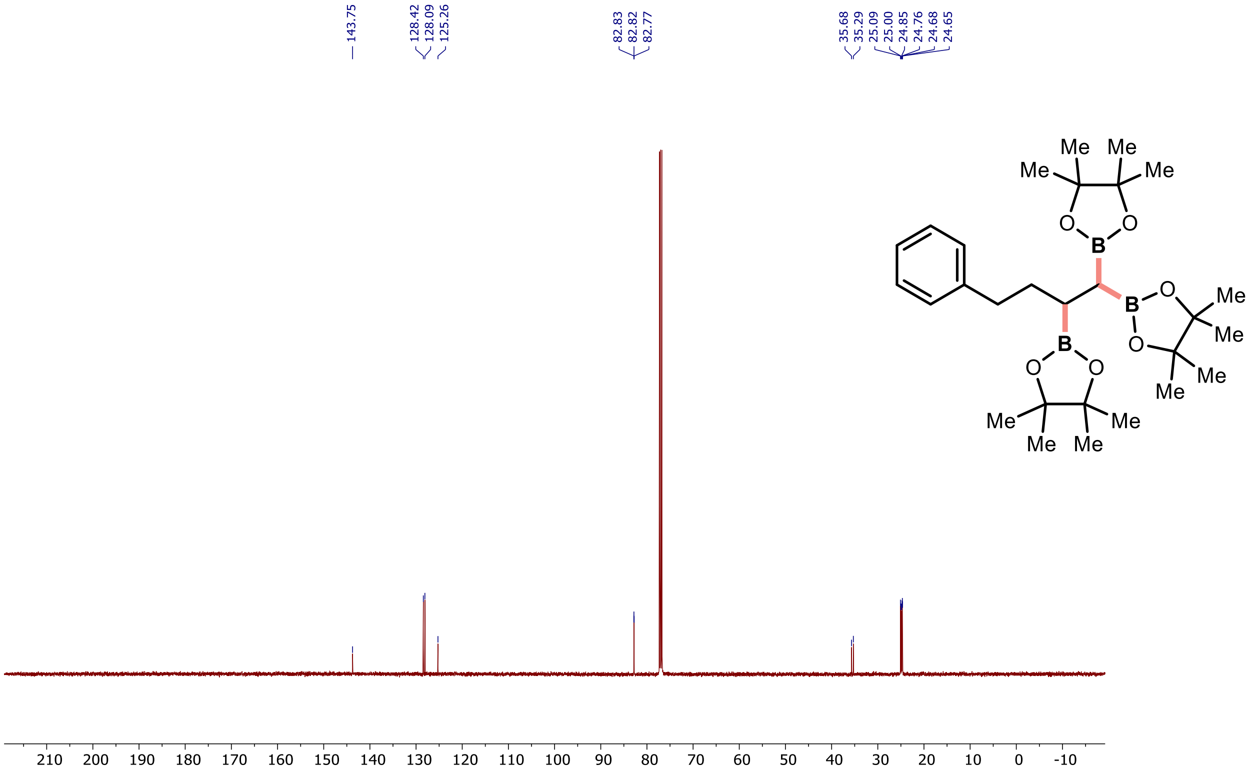

Supplementary Figure 40b-3 ^{11}B NMR (161 MHz, $CDCl_3$) 2,2',2''-(4-Phenylbutane-1,1,2-triyl)tris(4,4,5,5-tetramethyl-1,3,2-dioxaborolane)

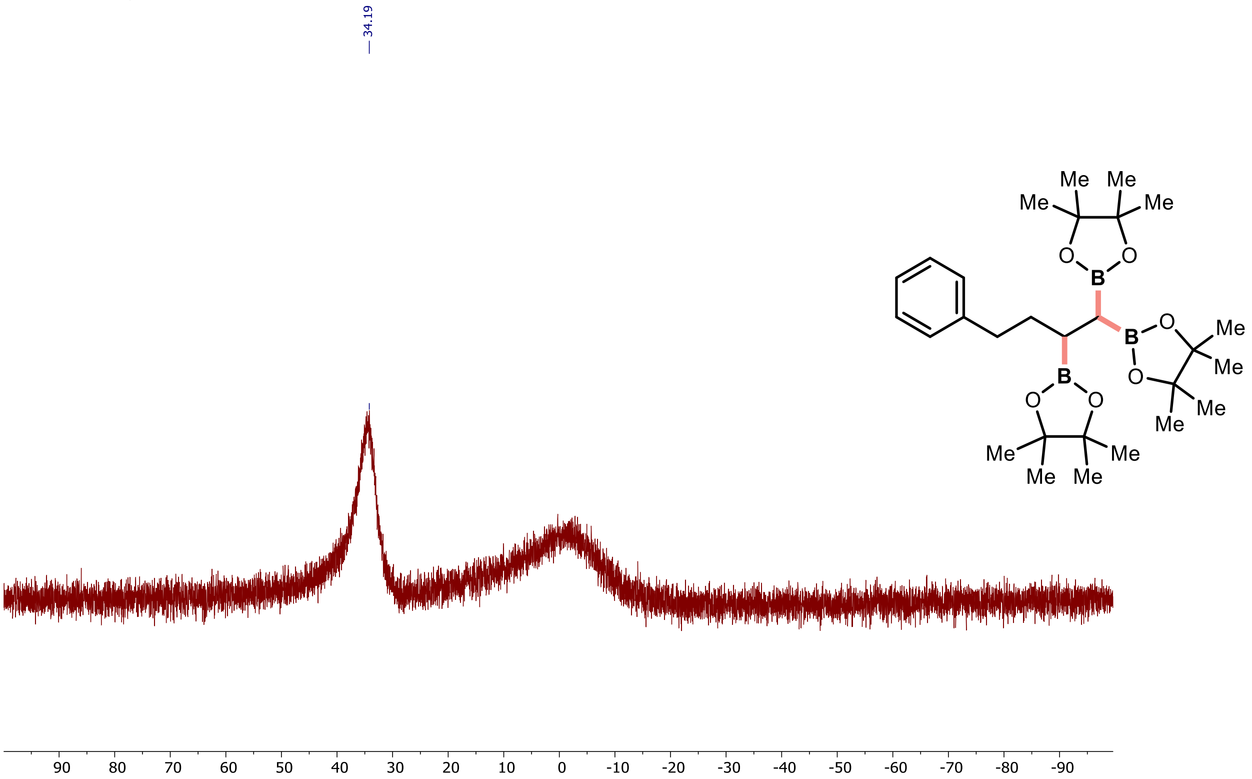

Supplementary Figure 43b'-1 ^{1}H NMR (500 MHz, $CDCl_3$) 2,2'-(((3*r*,5*r*,7*r*)-Adamantan-1-yl)methylene)bis(4,4,5,5-tetramethyl-1,3,2-dioxaborolane)

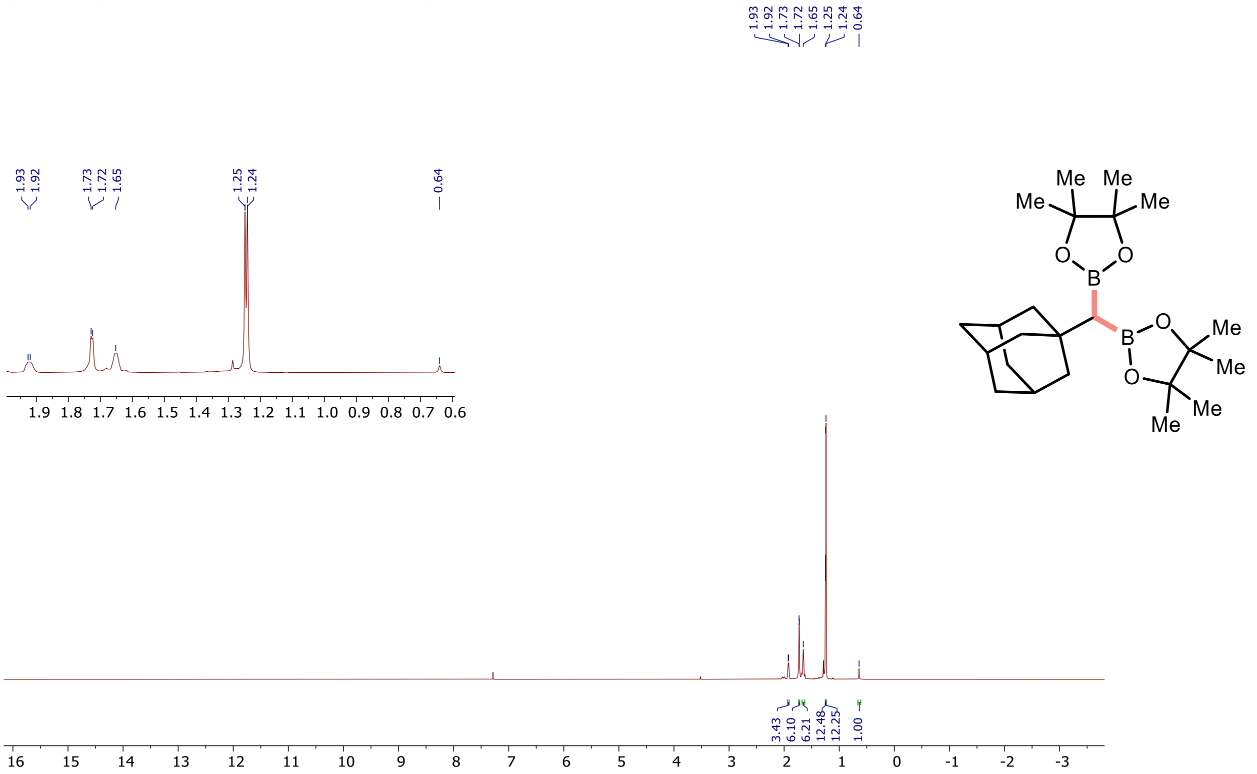

Supplementary Figure 43b'-2 ^{13}C NMR (125 MHz, $CDCl_3$) 2,2'-(((3*r*,5*r*,7*r*)-Adamantan-1-yl)methylene)bis(4,4,5,5-tetramethyl-1,3,2-dioxaborolane)

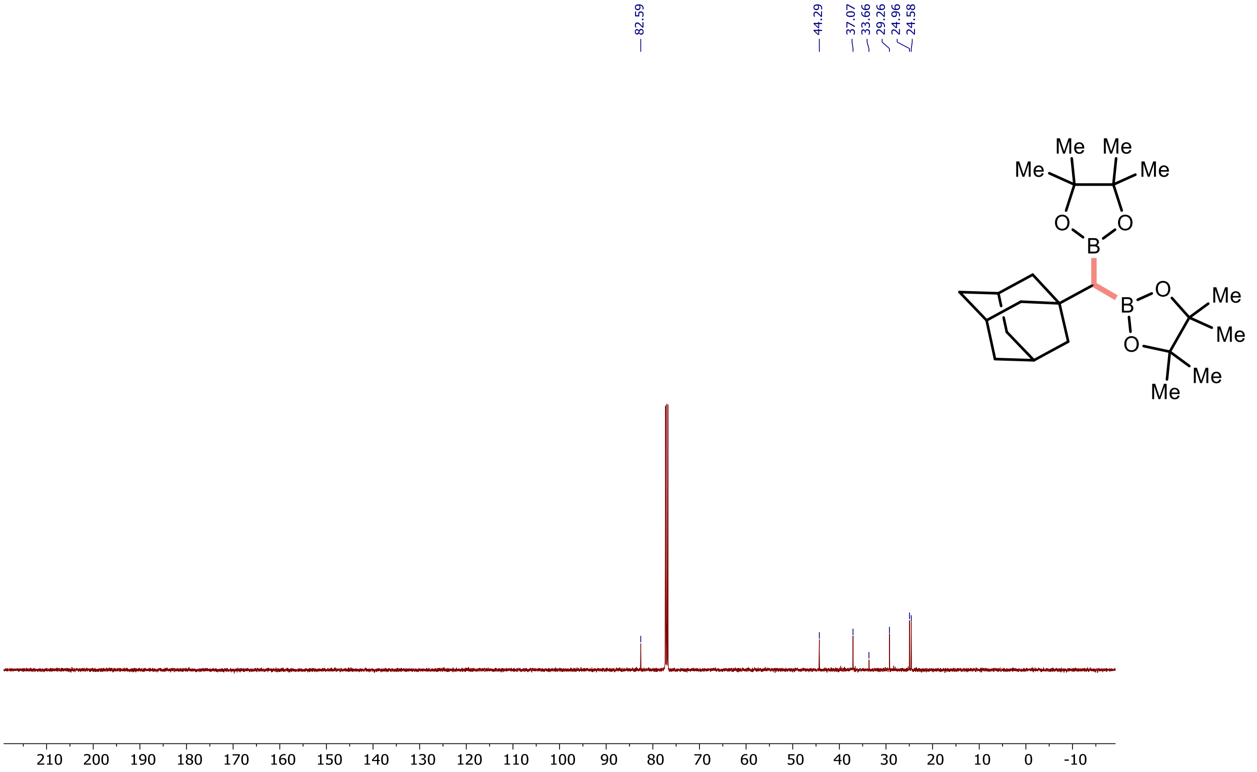

Supplementary Figure 43b'-3 ^{11}B NMR (161 MHz, $CDCl_3$) 2,2'-(((3*r*,5*r*,7*r*)-Adamantan-1-yl)methylene)bis(4,4,5,5-tetramethyl-1,3,2-dioxaborolane)

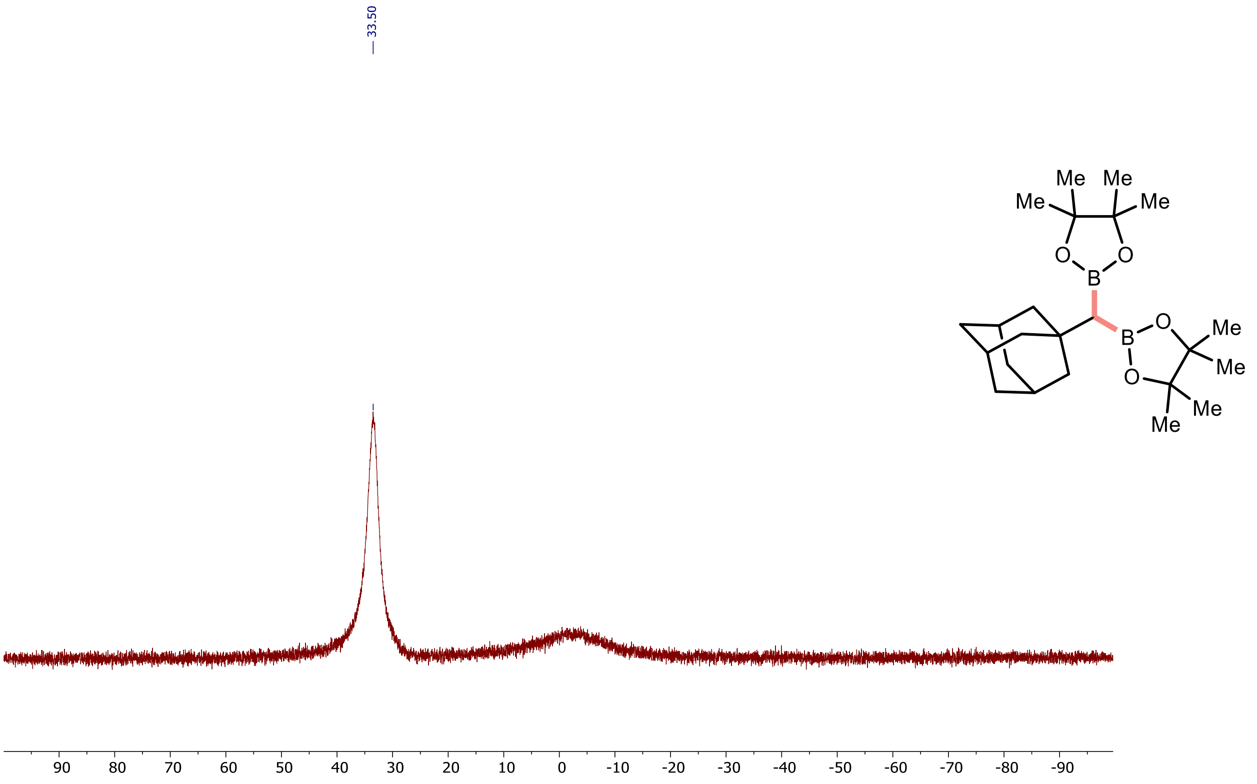

Supplementary Figure 44b'-1 ^{1}H NMR (500 MHz, $CDCl_3$) 2,2'-(2,2-Dimethylpropane-1,1-diyl)bis(4,4,5,5-tetramethyl-1,3,2-dioxaborolane)

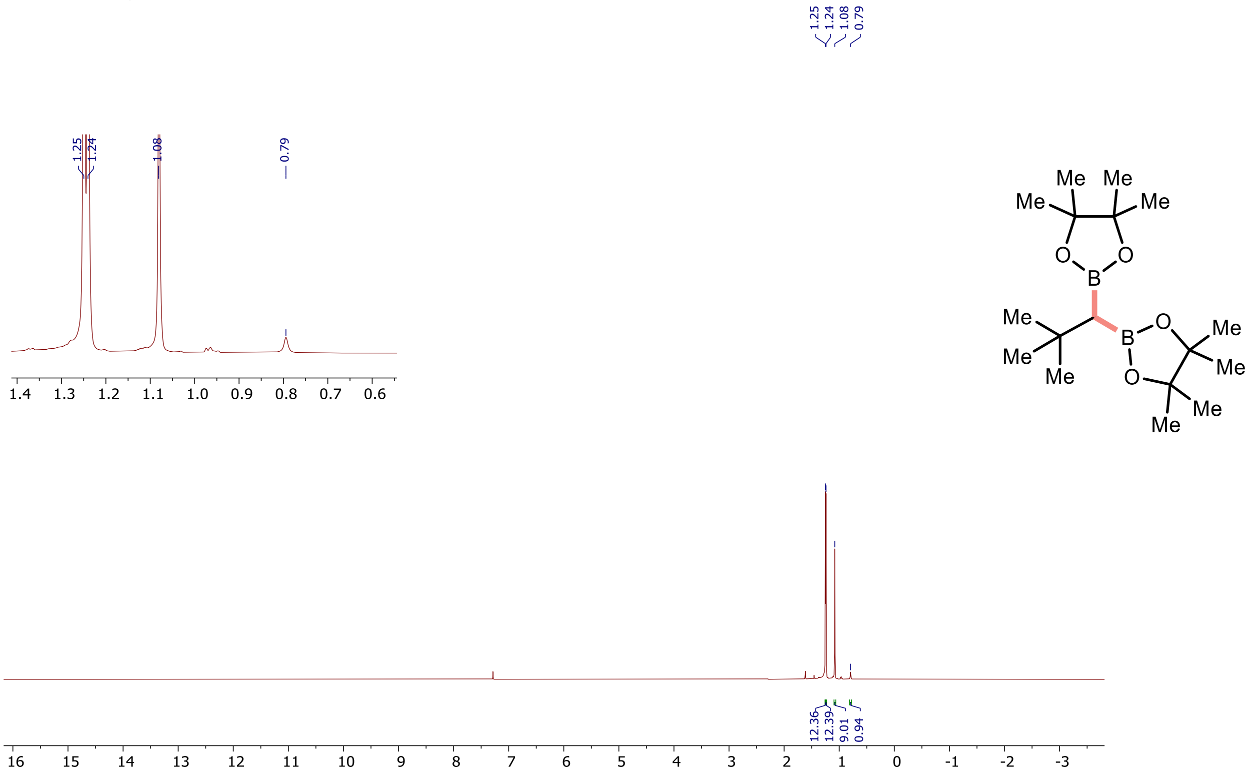

Supplementary Figure 44b'-2 ^{13}C NMR (125 MHz, $CDCl_3$) 2,2'-(2,2-Dimethylpropane-1,1-diyl)bis(4,4,5,5-tetramethyl-1,3,2-dioxaborolane)

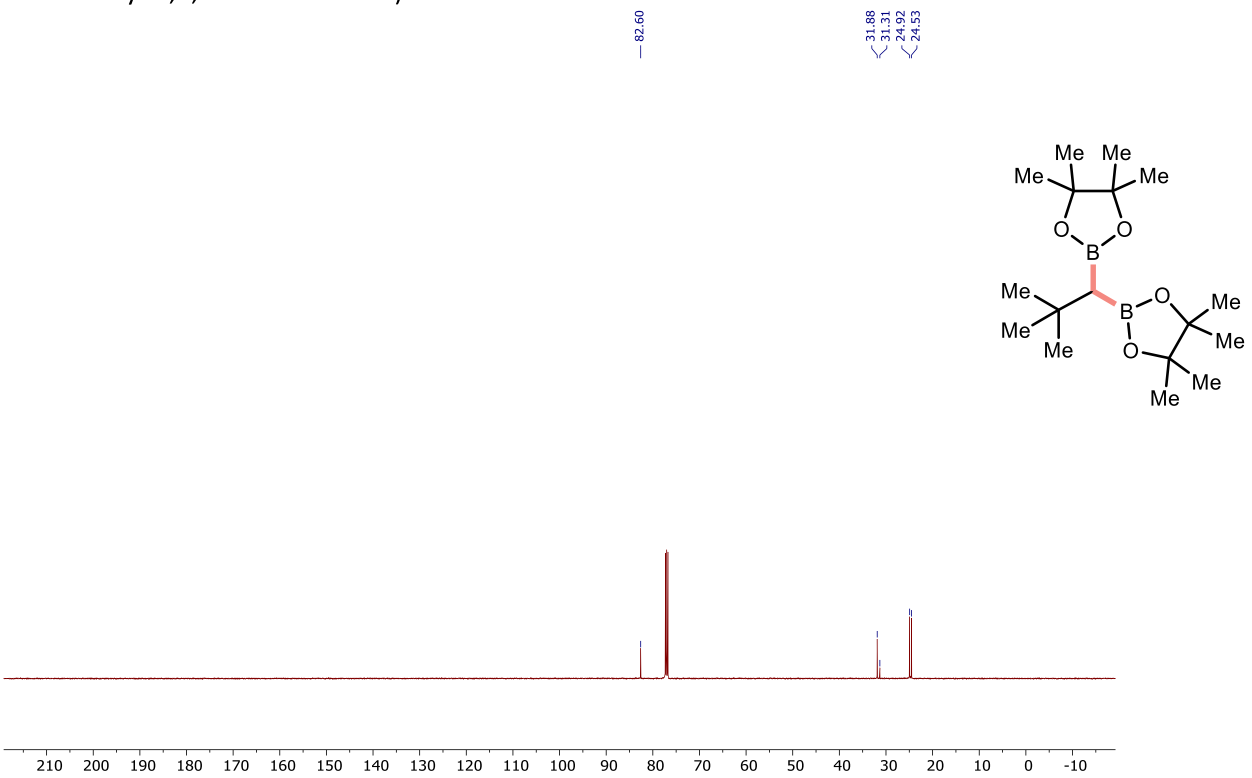

Supplementary Figure 44b'-3 ^{11}B NMR (161 MHz, $CDCl_3$) 2,2'-(2,2-Dimethylpropane-1,1-diyl)bis(4,4,5,5-tetramethyl-1,3,2-dioxaborolane)

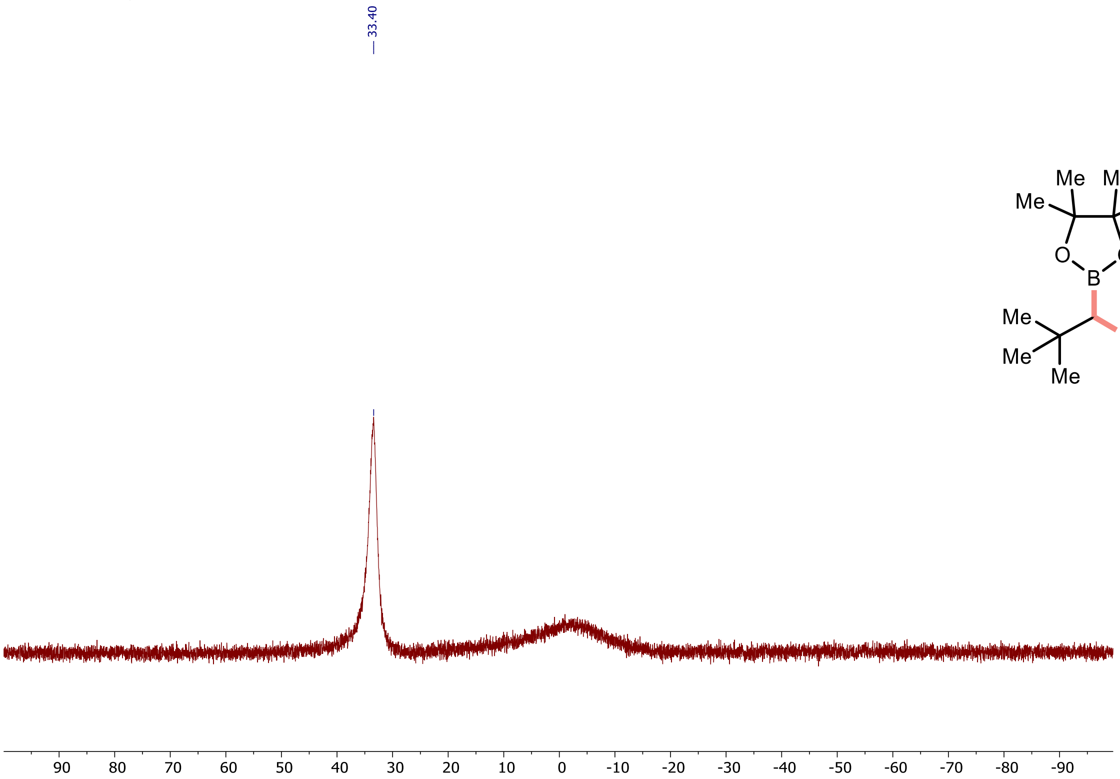

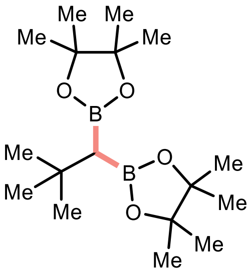

Supplementary Figure 45g-1 ^{1}H NMR (500 MHz, $CDCl_3$) (Diphenyl(4-(((3a*S*,4*S*,6*S*,7a*R*)-3a,5,5-trimethylhexahydro-4,6-methanobenzo[*d*][1,3,2]dioxaborol-2-yl)methyl)phenyl)phosphane

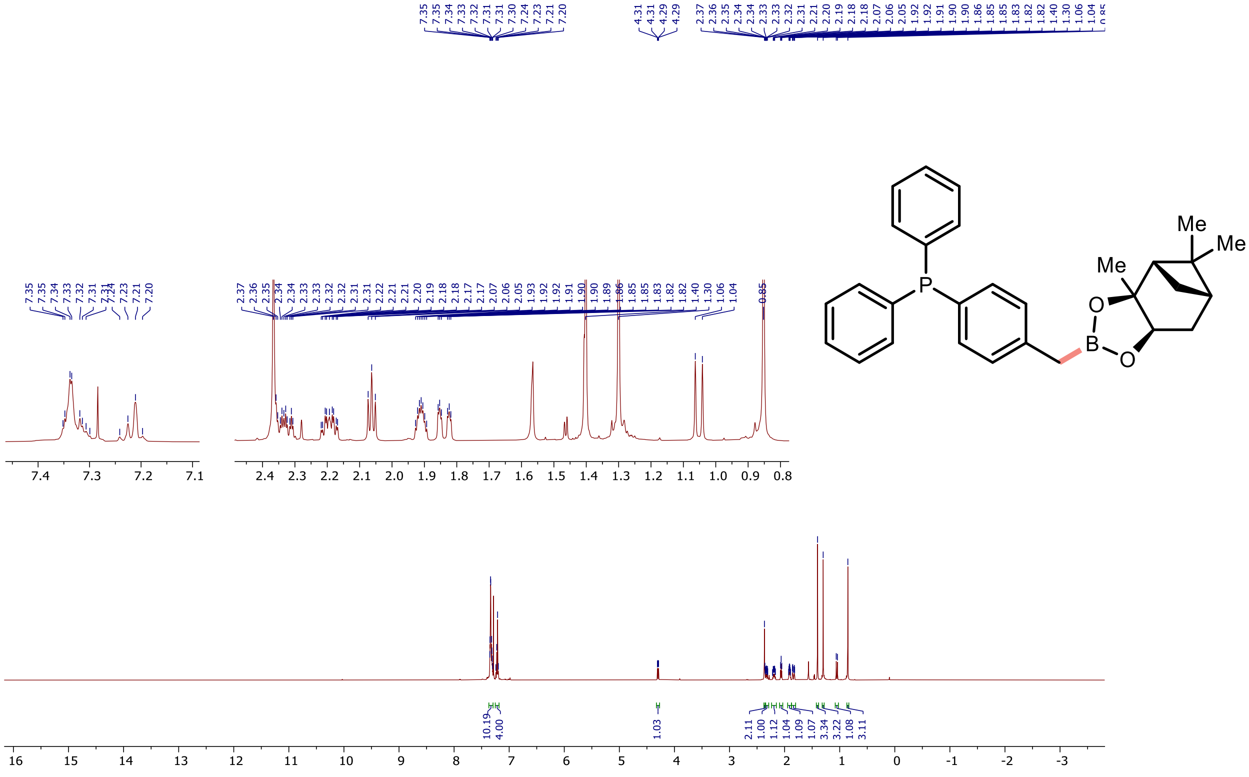

Supplementary Figure 45g-2 ^{13}C NMR (125 MHz, $CDCl_3$) (Diphenyl(4-(((3a*S*,4*S*,6*S*,7a*R*)-3a,5,5-trimethylhexahydro-4,6-methanobenzo[*d*][1,3,2]dioxaborol-2-yl)methyl)phenyl)phosphane

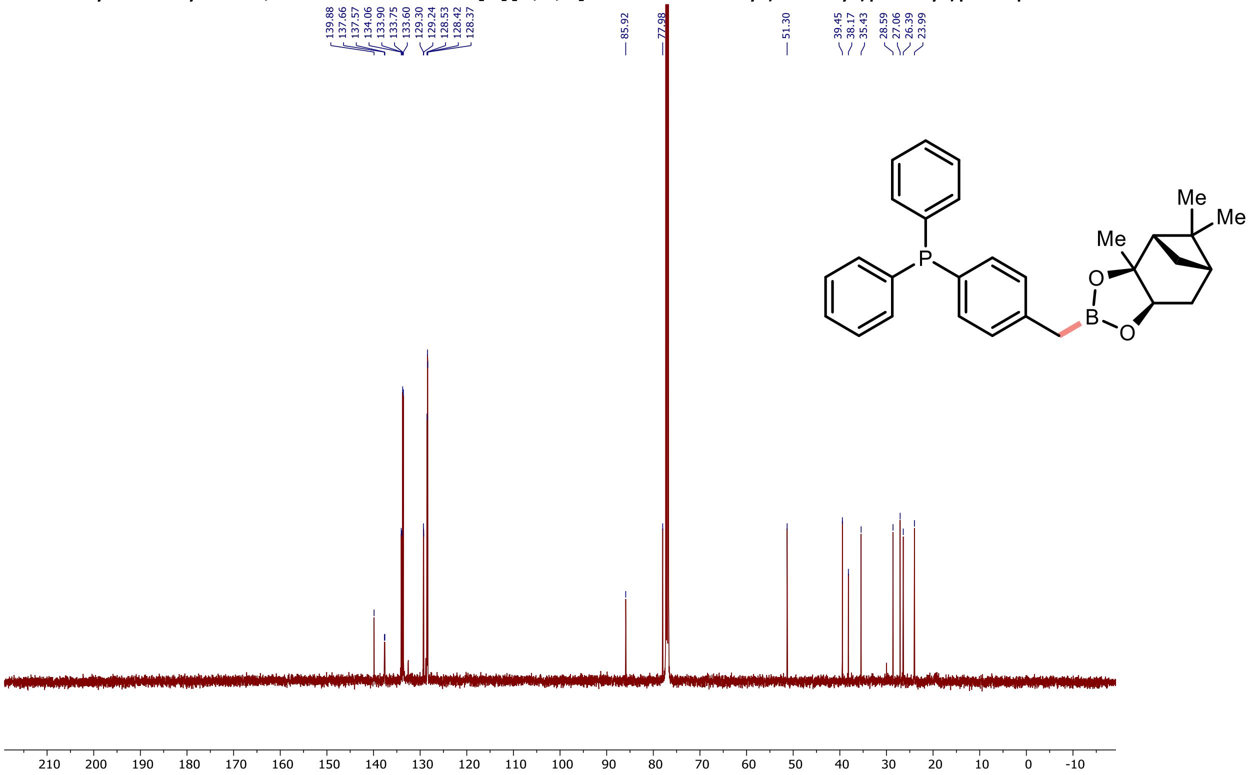

Supplementary Figure 45g-3 ^{31}P NMR (202 MHz, $CDCl_3$) (Diphenyl(4-(((3a*S*,4*S*,6*S*,7a*R*)-3a,5,5-trimethylhexahydro-4,6-methanobenzo[*d*][1,3,2]dioxaborol-2-yl)methyl)phenyl)phosphane

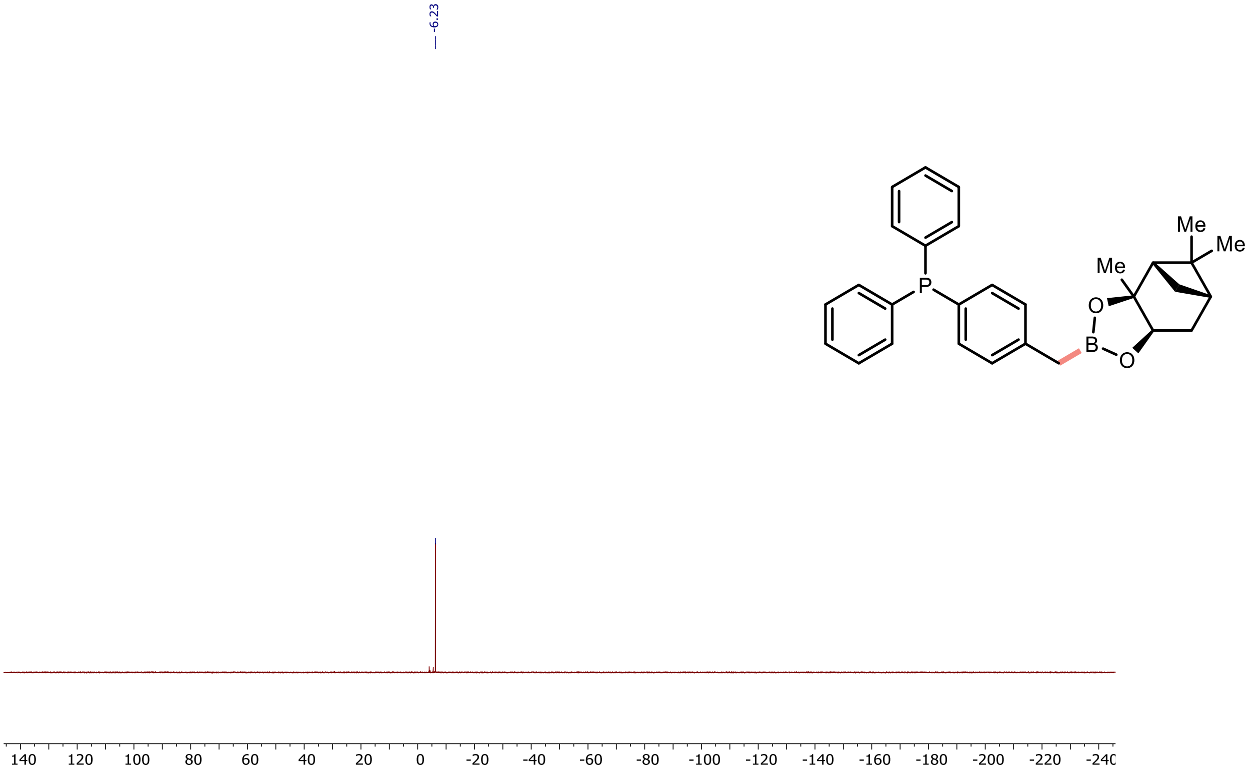

Supplementary Figure 45g-4 ^{11}B NMR (161 MHz, $CDCl_3$) (Diphenyl(4-(((3a*S*,4*S*,6*S*,7a*R*)-3a,5,5-trimethylhexahydro-4,6-methanobenzo[*d*][1,3,2]dioxaborol-2-yl)methyl)phenyl)phosphane

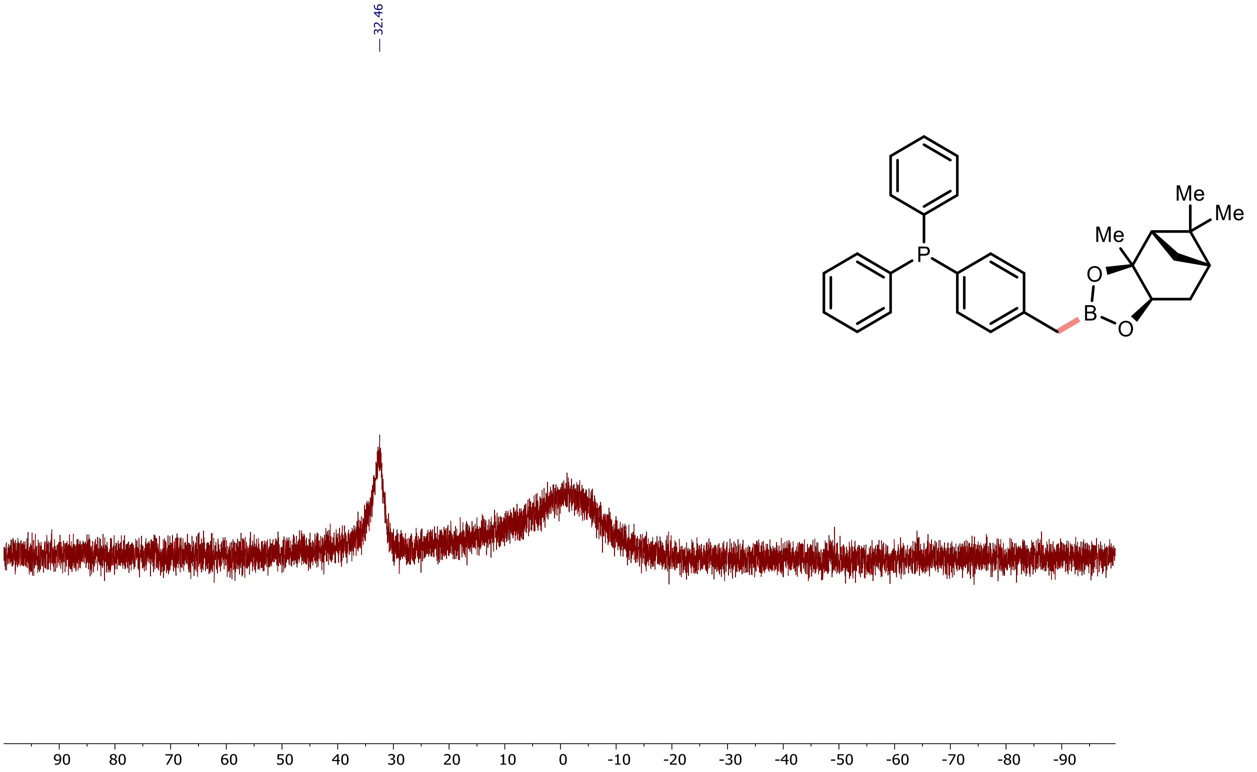

Supplementary Figure 46b-1 ^{1}H NMR (500 MHz, $CDCl_3$) 2-(4-((((1*R*,2*S*,5*R*)-2-Isopropyl-5-methylcyclohexyl)oxy)methyl)benzyl)-4,4,5,5-tetramethyl-1,3,2-dioxaborolane

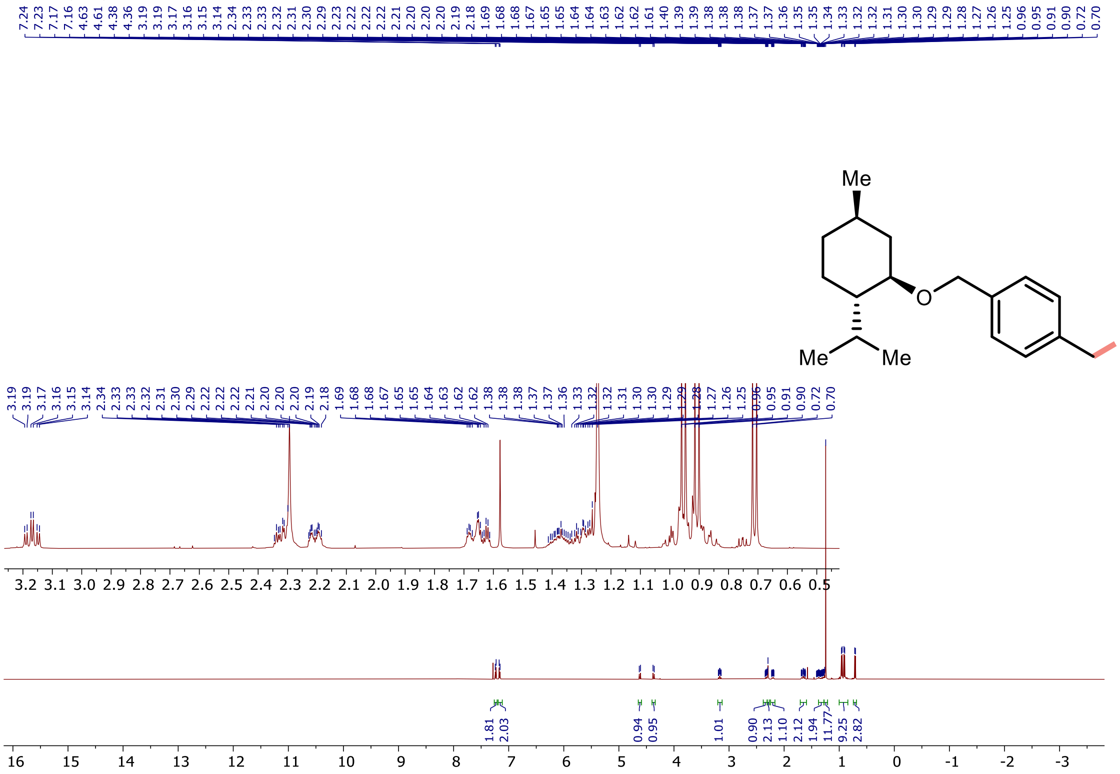

Supplementary Figure 46b-2 ^{13}C NMR (MHz, $CDCl_3$) 2-(4-((((1*R*,2*S*,5*R*)-2-Isopropyl-5-methylcyclohexyl)oxy)methyl)benzyl)-4,4,5,5-tetramethyl-1,3,2-dioxaborolane

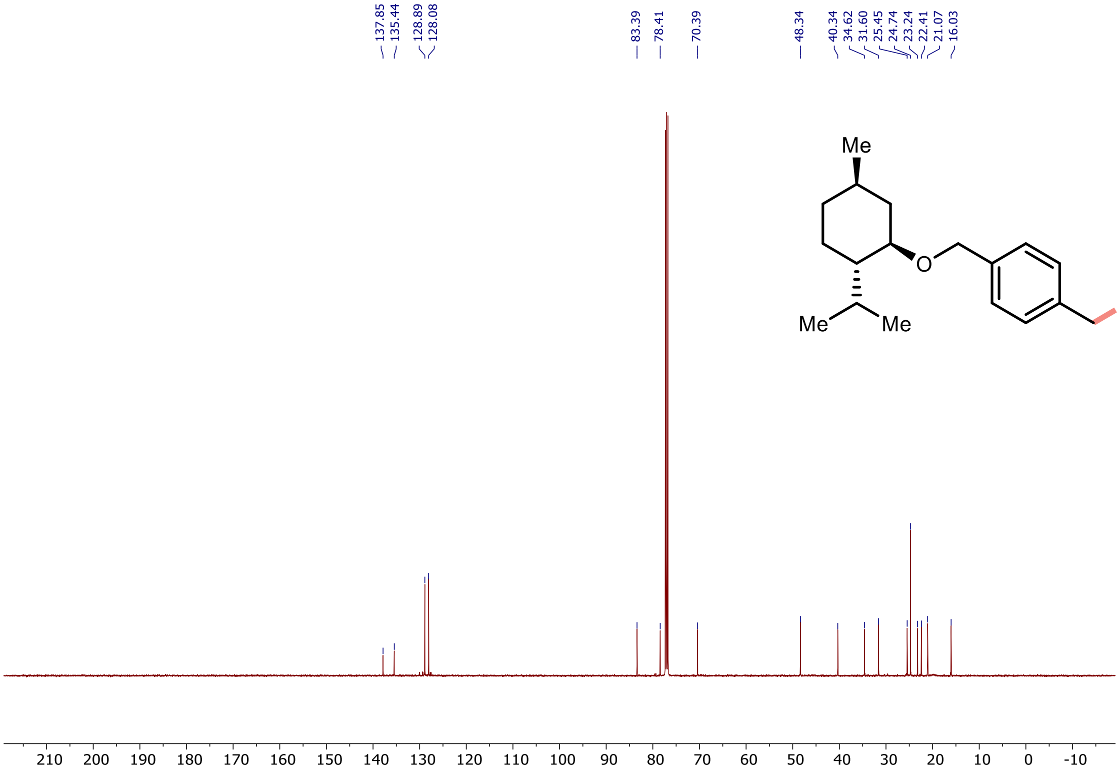

Supplementary Figure 46b-3 ^{11}B NMR (161 MHz, $CDCl_3$) 2-(4-((((1*R*,2*S*,5*R*)-2-Isopropyl-5-methylcyclohexyl)oxy)methyl)benzyl)-4,4,5,5-tetramethyl-1,3,2-dioxaborolane

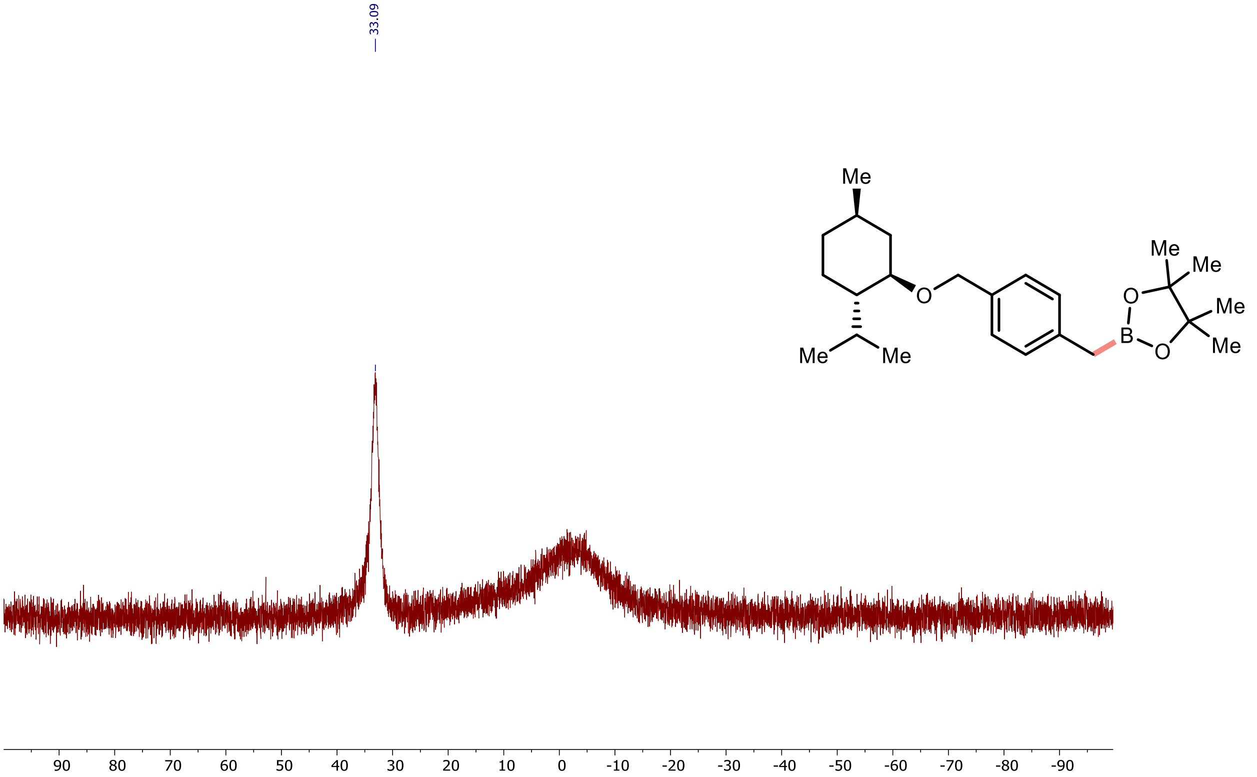

Supplementary Figure 47b-1 ^{1}H NMR (500 MHz, $CDCl_3$) 2-((6-(3-((3*r*,5*r*,7*r*)-Adamantan-1-yl)-4-methoxyphenyl)naphthalen-2-yl)methyl)-4,4,5,5-tetramethyl-1,3,2-dioxaborolane

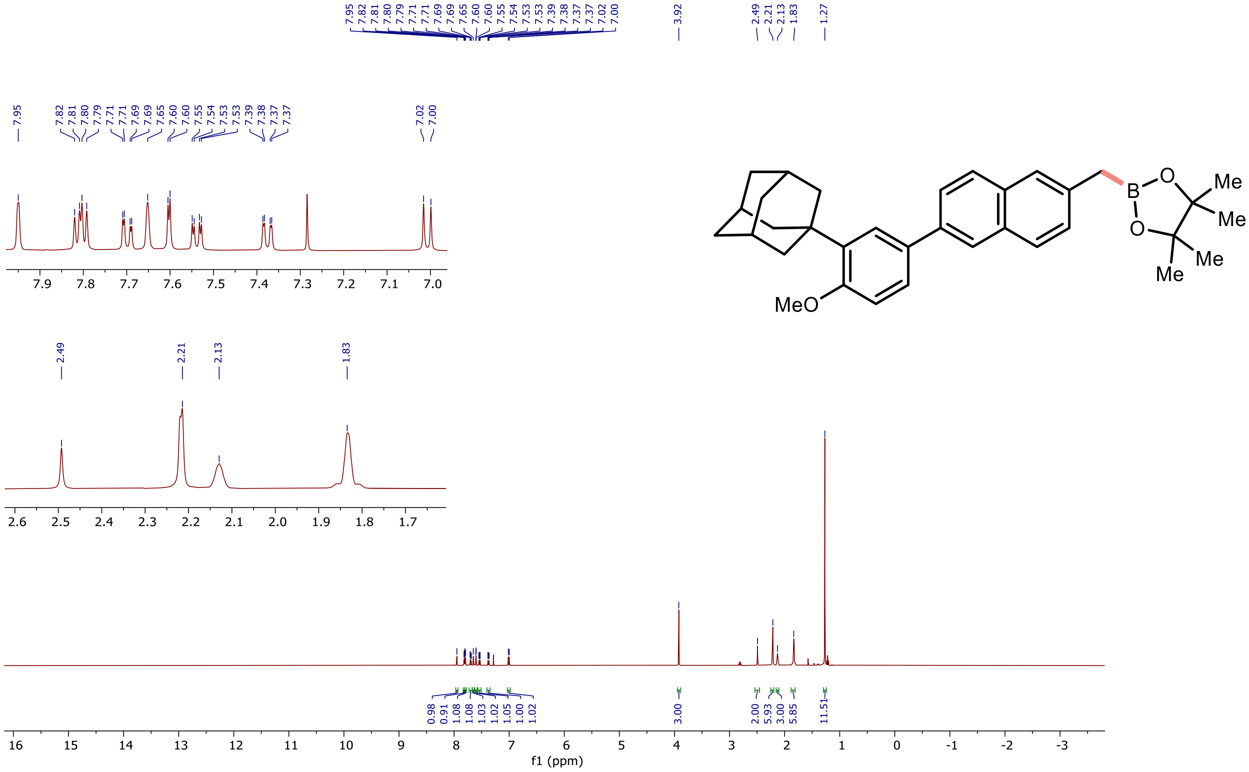

Supplementary Figure 47b-2 ^{13}C NMR (125 MHz, $CDCl_3$) 2-((6-(3-((3*r*,5*r*,7*r*)-Adamantan-1-yl)-4-methoxyphenyl)naphthalen-2-yl)methyl)-4,4,5,5-tetramethyl-1,3,2-dioxaborolane

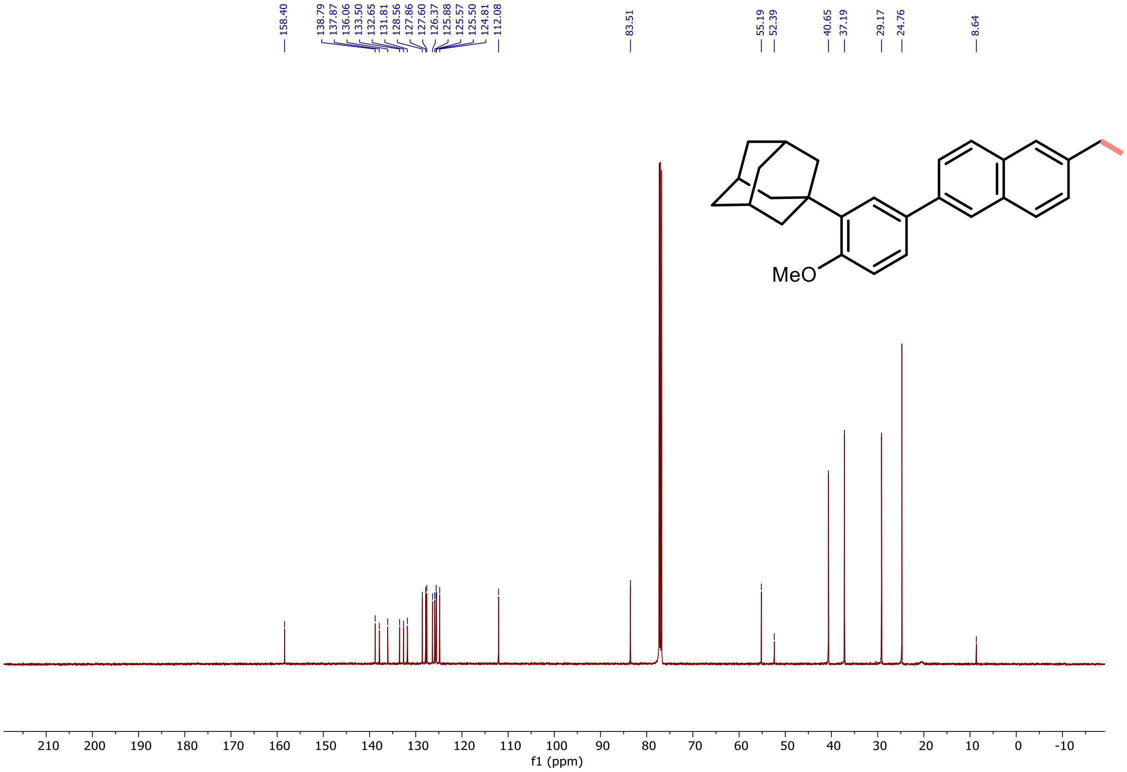

Supplementary Figure 47b-3 ^{11}B NMR (161 MHz, $CDCl_3$) 2-((6-(3-((3*r*,5*r*,7*r*)-Adamantan-1-yl)-4-methoxyphenyl)naphthalen-2-yl)methyl)-4,4,5,5-tetramethyl-1,3,2-dioxaborolane

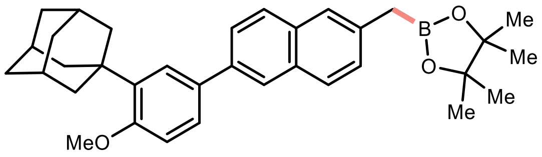

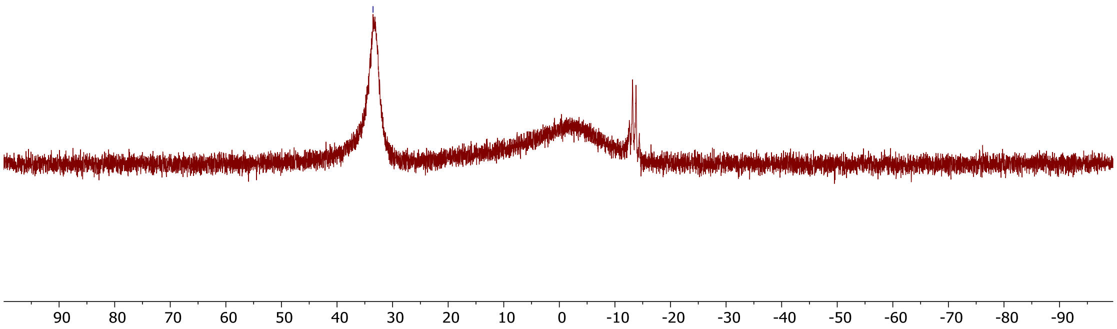

Supplementary Figure 48b-1 ^{1}H NMR (500 MHz, $CDCl_3$) 2-(4-((1*s*,4*r*)-4-Butylcyclohexyl)benzyl)-4,4,5,5-tetramethyl-1,3,2-dioxaborolane

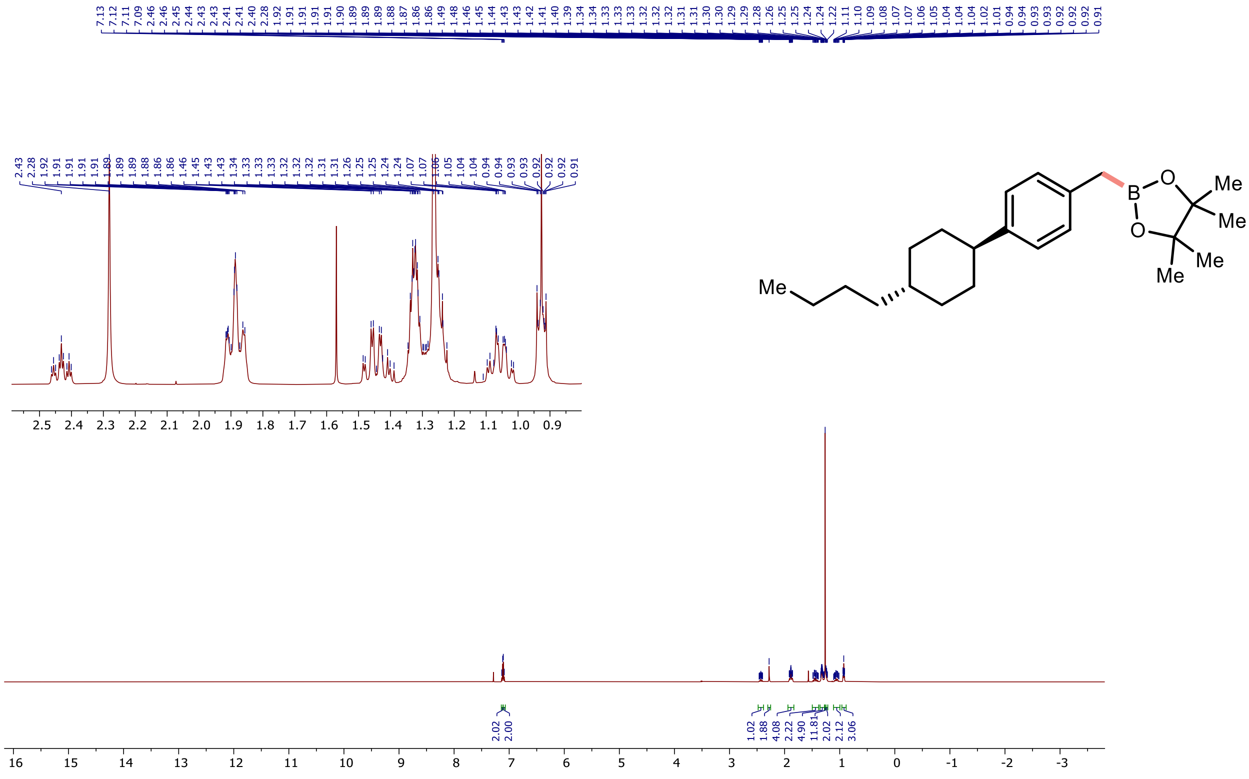

Supplementary Figure 48b-2 ^{13}C NMR (125 MHz, $CDCl_3$) 2-(4-((1*s*,4*r*)-4-Butylcyclohexyl)benzyl)-4,4,5,5-tetramethyl-1,3,2-dioxaborolane

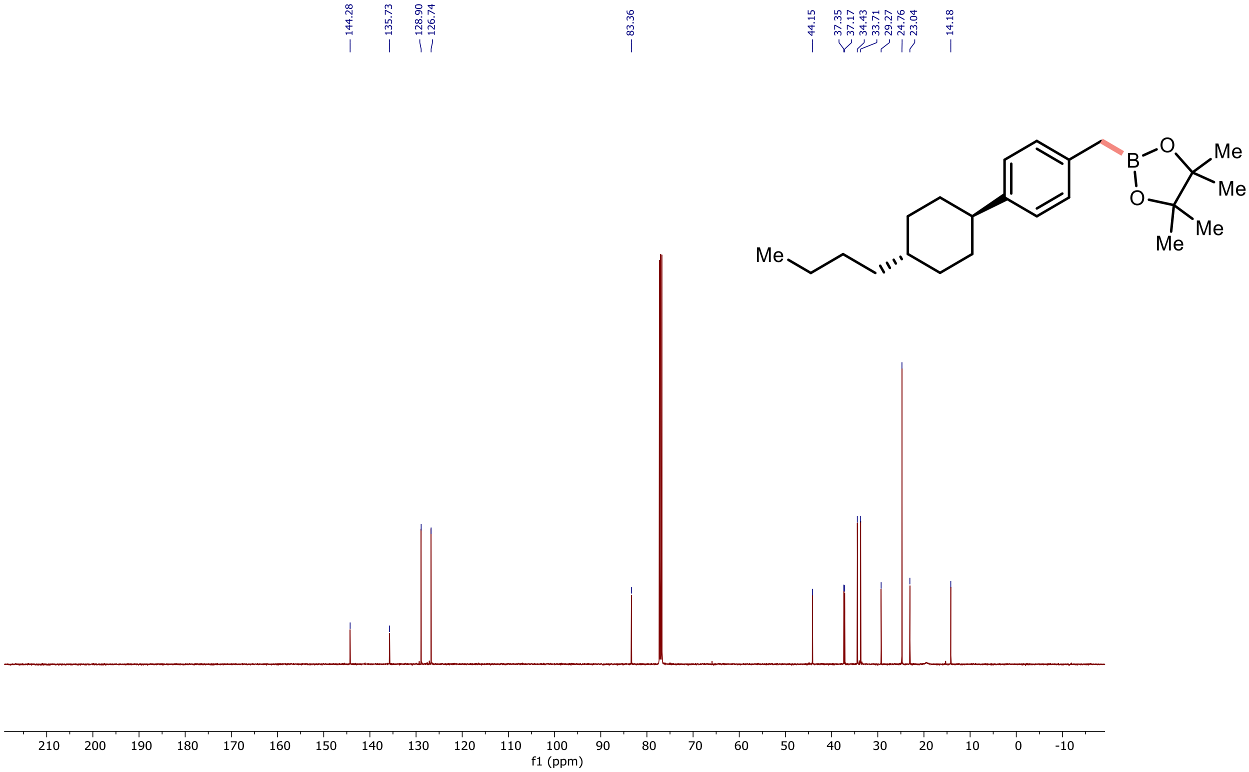

Supplementary Figure 48b-3 ^{11}B NMR (161 MHz, $CDCl_3$) 2-(4-((1*s*,4*r*)-4-Butylcyclohexyl)benzyl)-4,4,5,5-tetramethyl-1,3,2-dioxaborolane

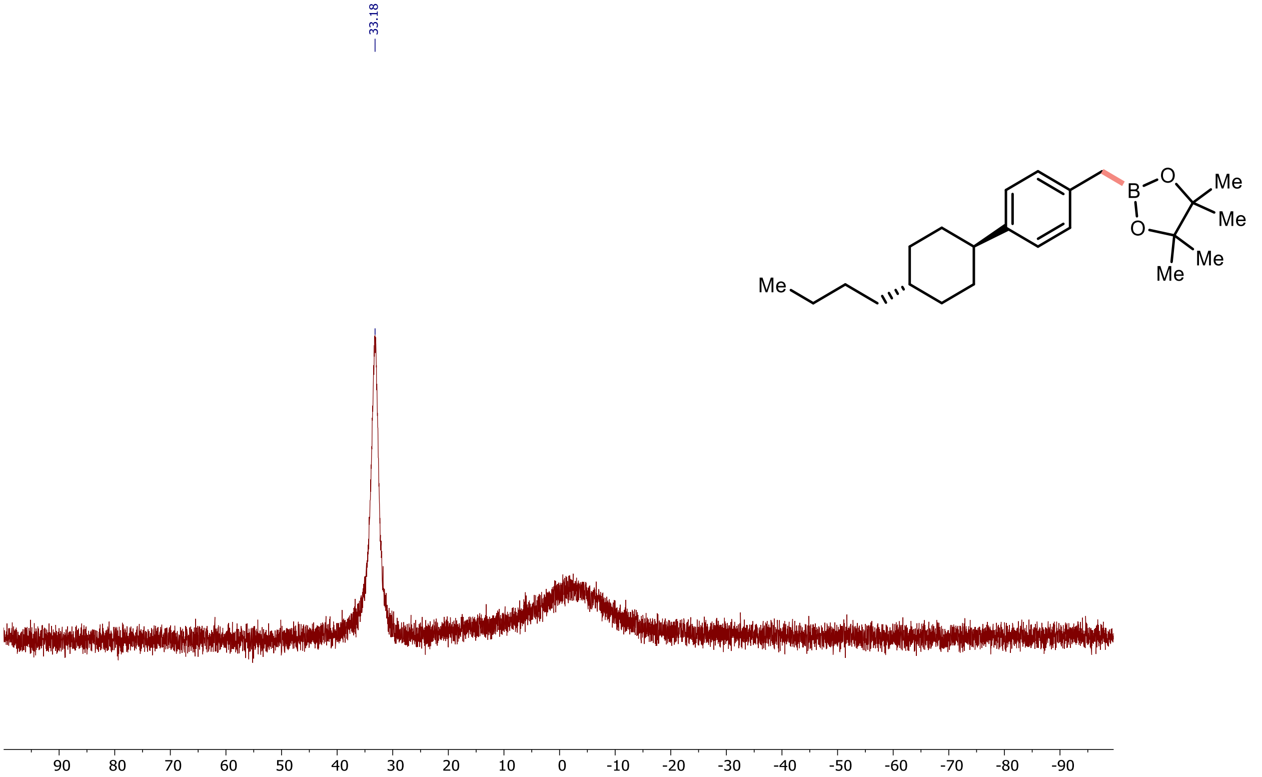

Supplementary Figure 49d-1 ^{1}H NMR (500 MHz, $CDCl_3$) 4-(Hydroxymethyl)-*N*,*N*-dipropylbenzenesulfonamide

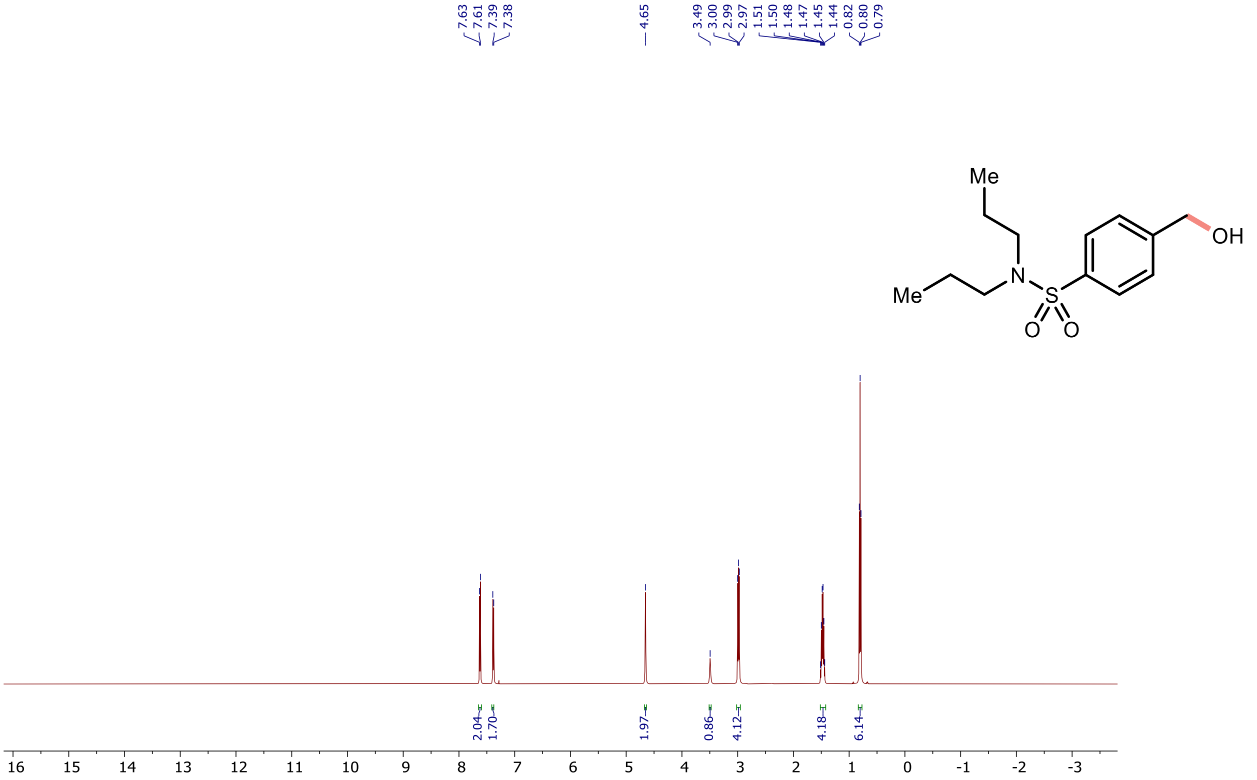

Supplementary Figure 49d-2 ^{13}C NMR (125 MHz, $CDCl_3$) 4-(Hydroxymethyl)-*N*,*N*-dipropylbenzenesulfonamide

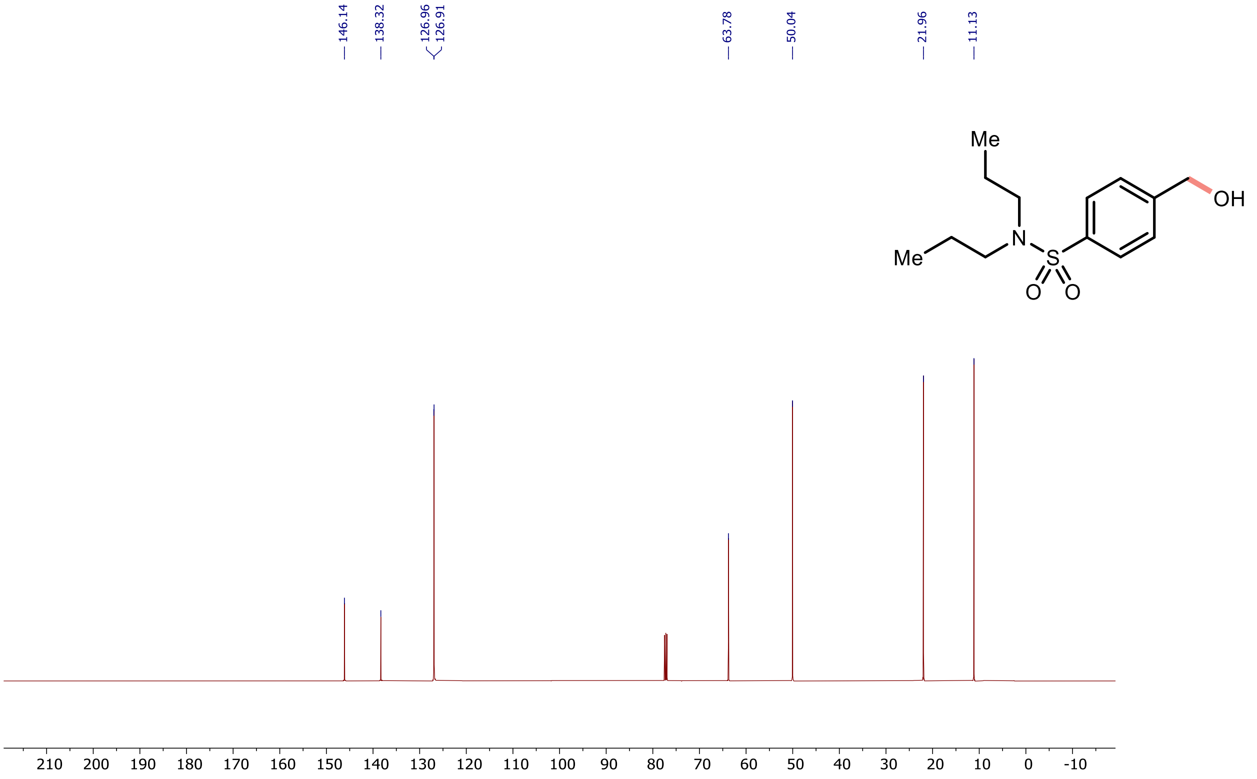

Supplementary Figure 50b-1 ^{1}H NMR (500 MHz, $CDCl_3$) 2,2',2''-((4*R*)-4-((5*S*,8*R*,9*S*,10*S*,13*R*,14*S*,17*R*)-10,13-Dimethylhexadecahydro-1*H*-cyclopenta[a]phenanthren-17-yl)pentane-1,1,2-triyl)tris(4,4,5,5-tetramethyl-1,3,2-dioxaborolane)

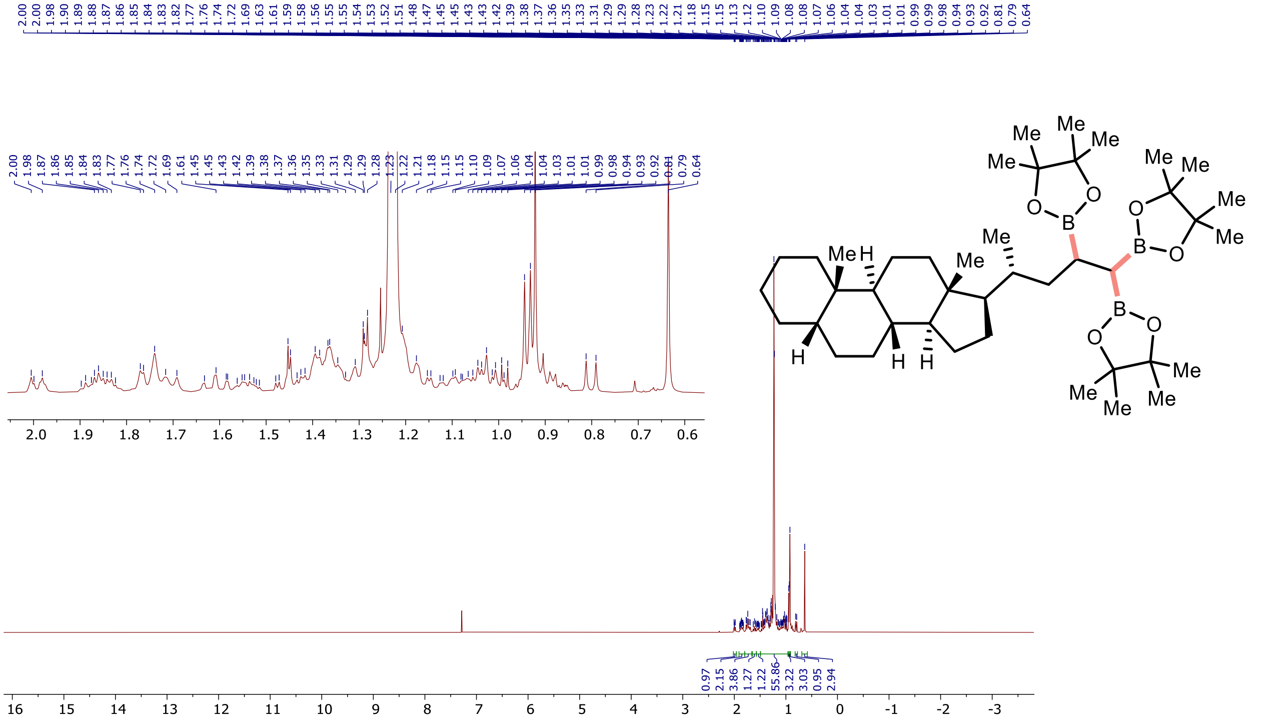

Supplementary Figure 50b-2 ^{13}C NMR (125 MHz, $CDCl_3$) 2,2',2''-((4*R*)-4-((5*S*,8*R*,9*S*,10*S*,13*R*,14*S*,17*R*)-10,13-Dimethylhexadecahydro-1*H*-cyclopenta[a]phenanthren-17-yl)pentane-1,1,2-triyl)tris(4,4,5,5-tetramethyl-1,3,2-dioxaborolane)

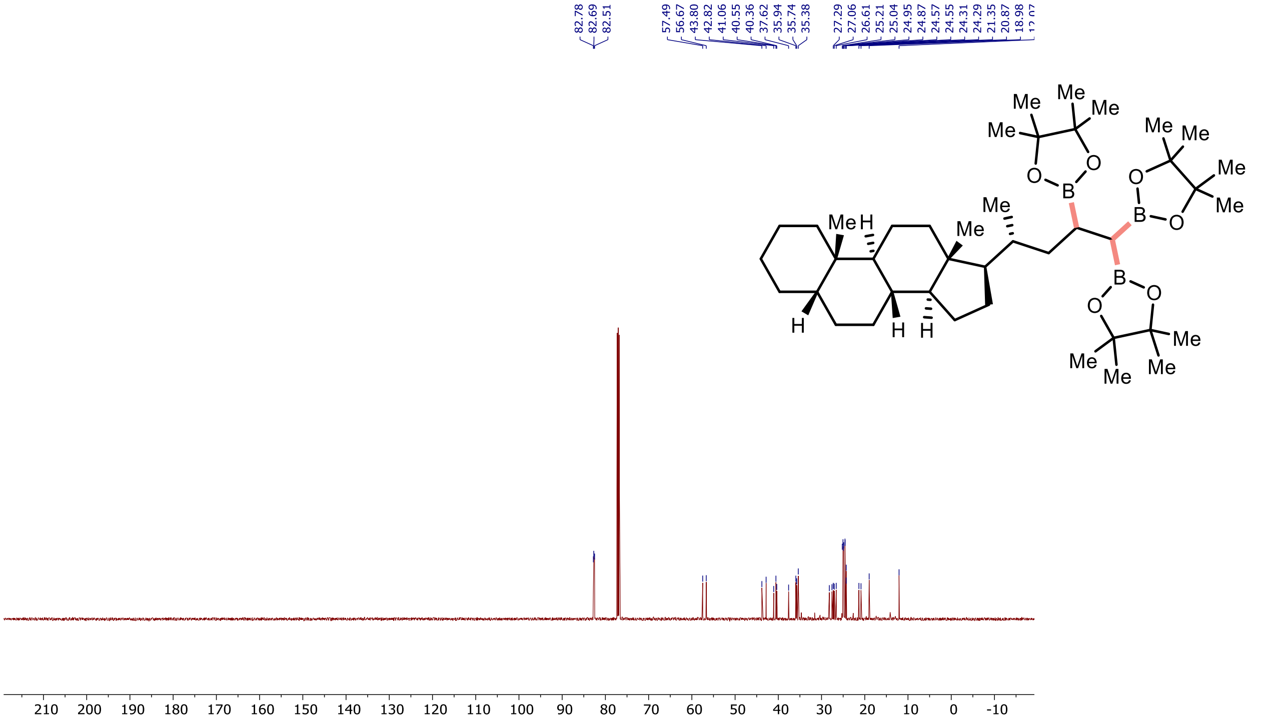

Supplementary Figure 50b-3 ^{11}B NMR (161 MHz, $CDCl_3$) 2,2',2''-((4*R*)-4-((5*S*,8*R*,9*S*,10*S*,13*R*,14*S*,17*R*)-10,13-Dimethylhexadecahydro-1*H*-cyclopenta[a]phenanthren-17-yl)pentane-1,1,2-triyl)tris(4,4,5,5-tetramethyl-1,3,2-dioxaborolane)

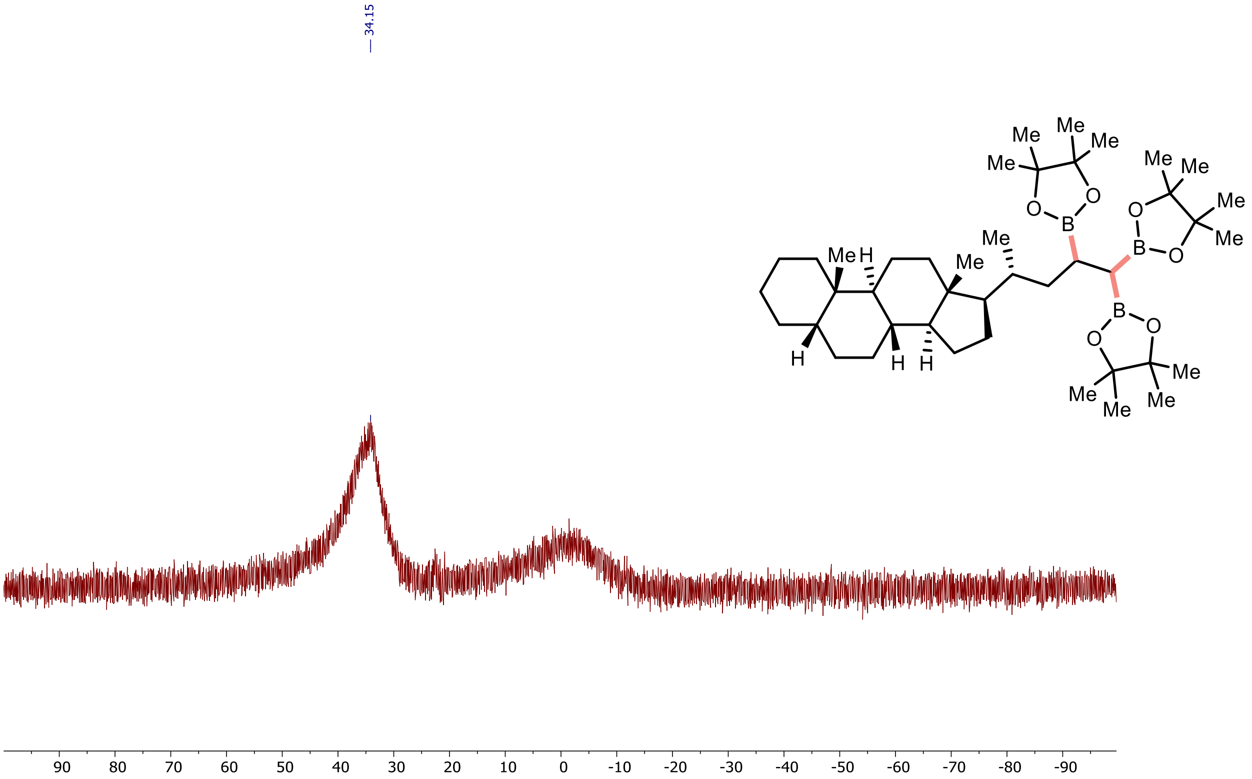

Supplementary Figure 51b-1 ^{1}H NMR (500 MHz, $CDCl_3$) 2,2',2''-(2-(4-Isobutylphenyl)ethane-1,1,2-triyl)tris(4,4,5,5-tetramethyl-1,3,2-dioxaborolane)

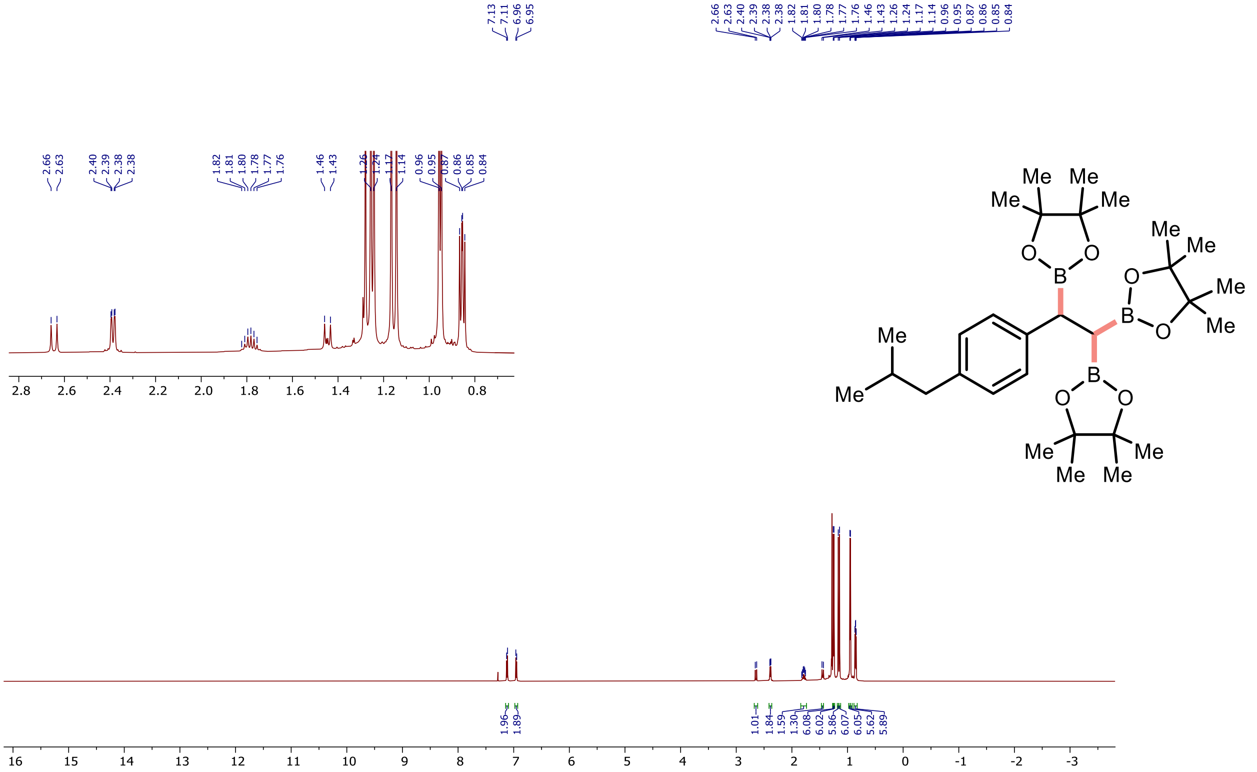

Supplementary Figure 51b-2 ^{13}C NMR (125 MHz, $CDCl_3$) 2,2',2''-(2-(4-Isobutylphenyl)ethane-1,1,2-triyl)tris(4,4,5,5-tetramethyl-1,3,2-dioxaborolane)

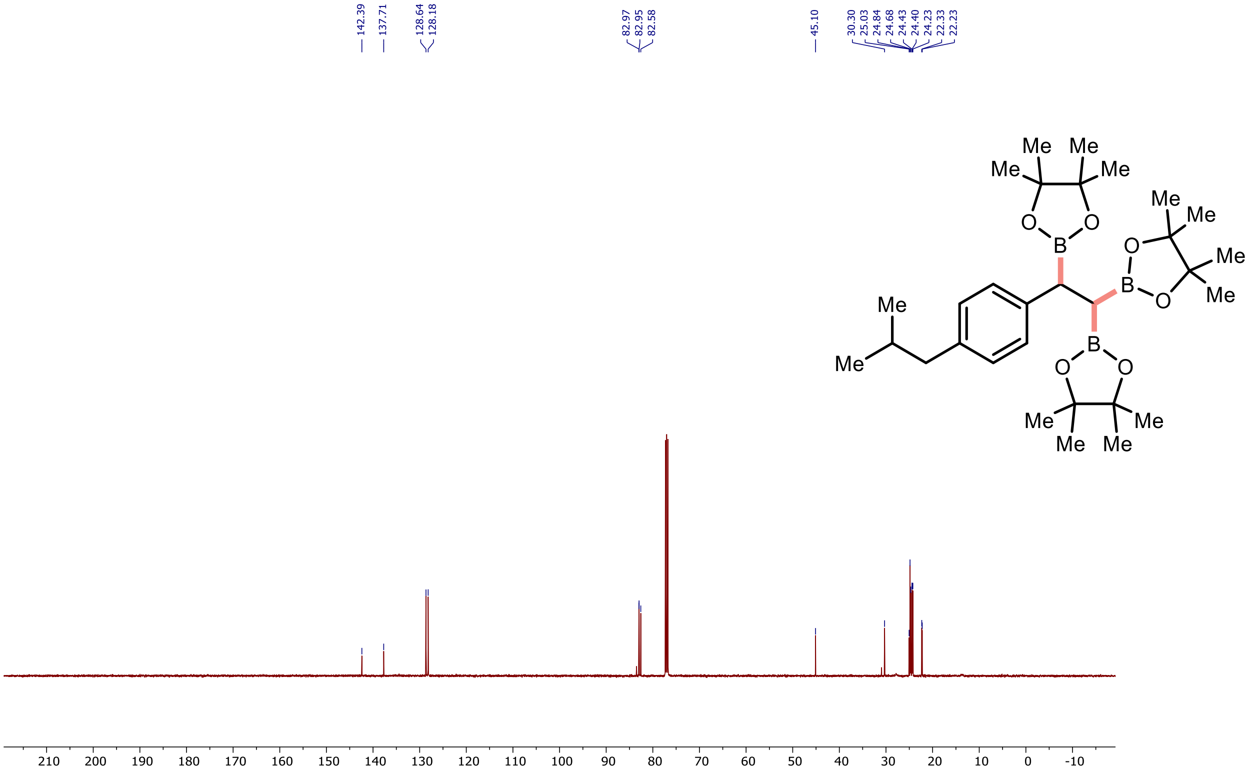

Supplementary Figure 51b-3 ^{11}B NMR (161 MHz, $CDCl_3$) 2,2',2''-(2-(4-Isobutylphenyl)ethane-1,1,2-triyl)tris(4,4,5,5-tetramethyl-1,3,2-dioxaborolane)

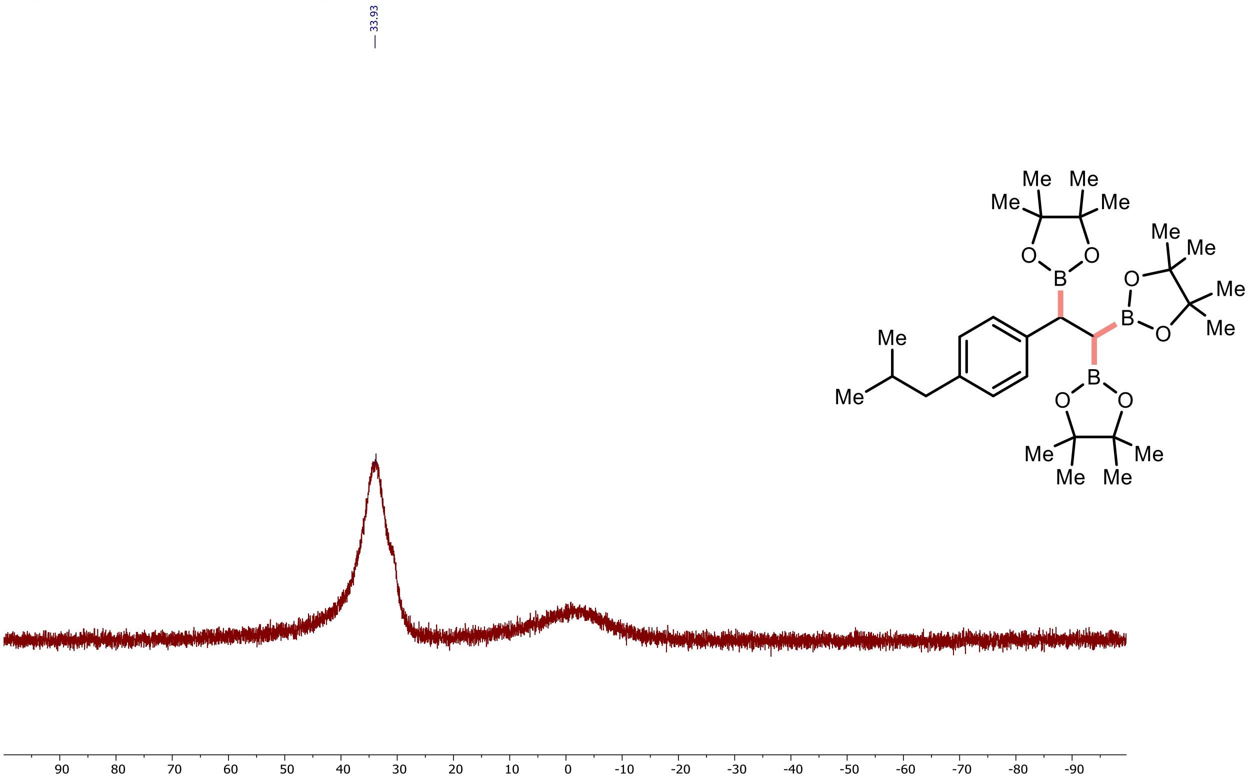

Supplementary Figure 52a-1 ^{1}H NMR (500 MHz, CD_3OD) 4-Benzylbenzoic acid

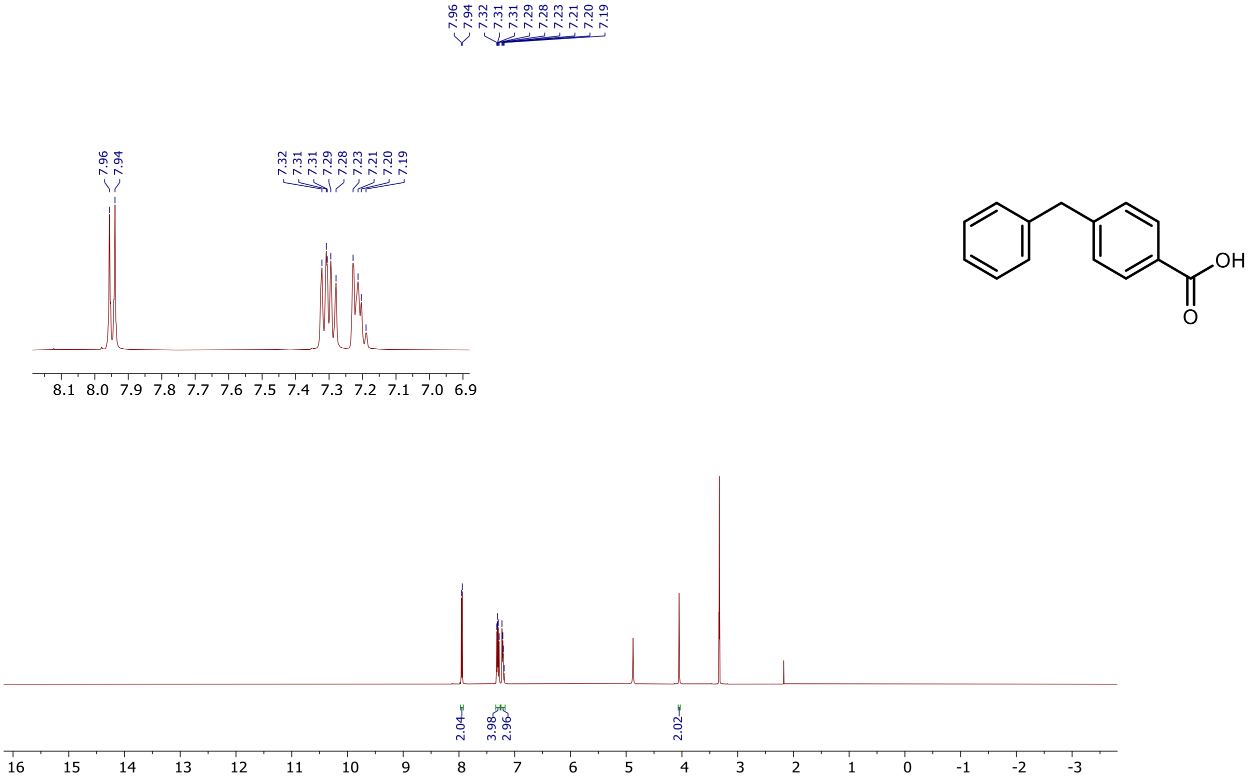

Supplementary Figure 52a-2 ^{13}C NMR (125 MHz, CD_3OD) 4-Benzylbenzoic acid

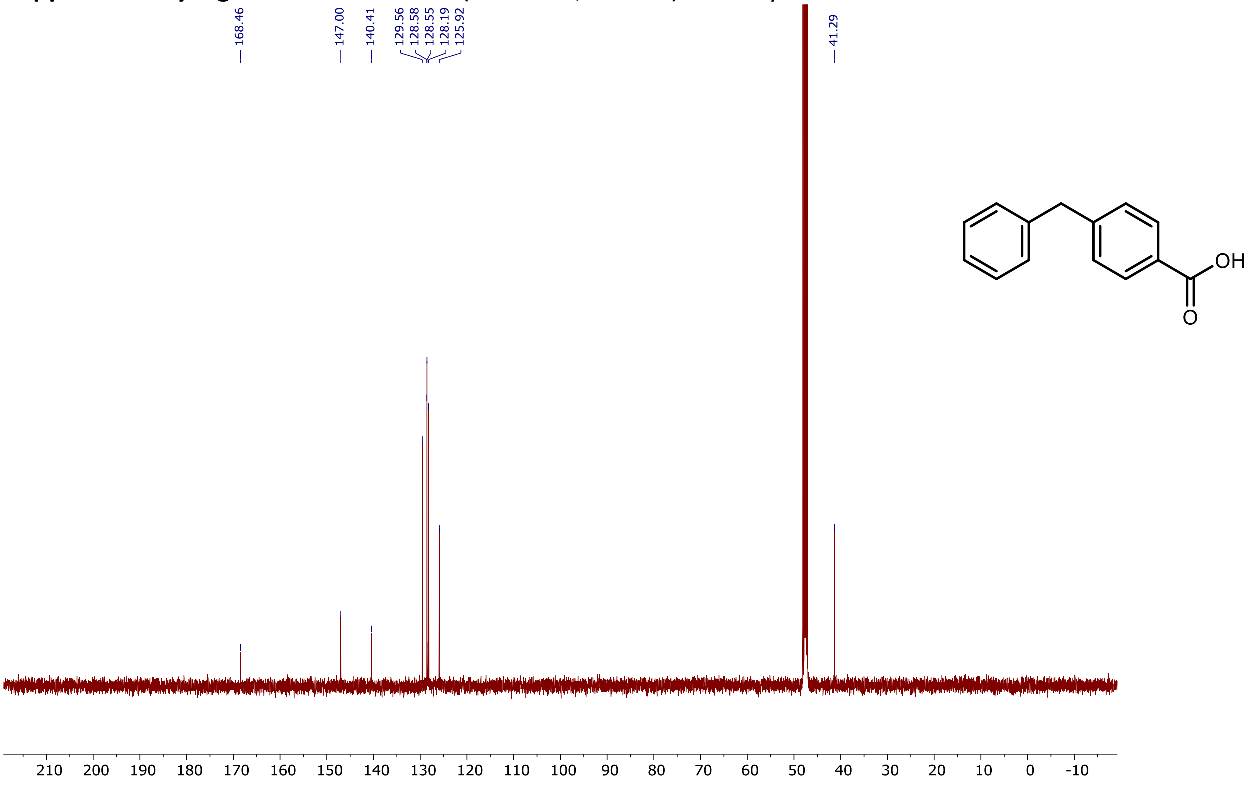

Supplementary Figure 52b-1 ^{1}H NMR (500 MHz, $CDCl_3$) 2-(4-Benzylbenzyl)-4,4,5,5-tetramethyl-1,3,2-dioxaborolane

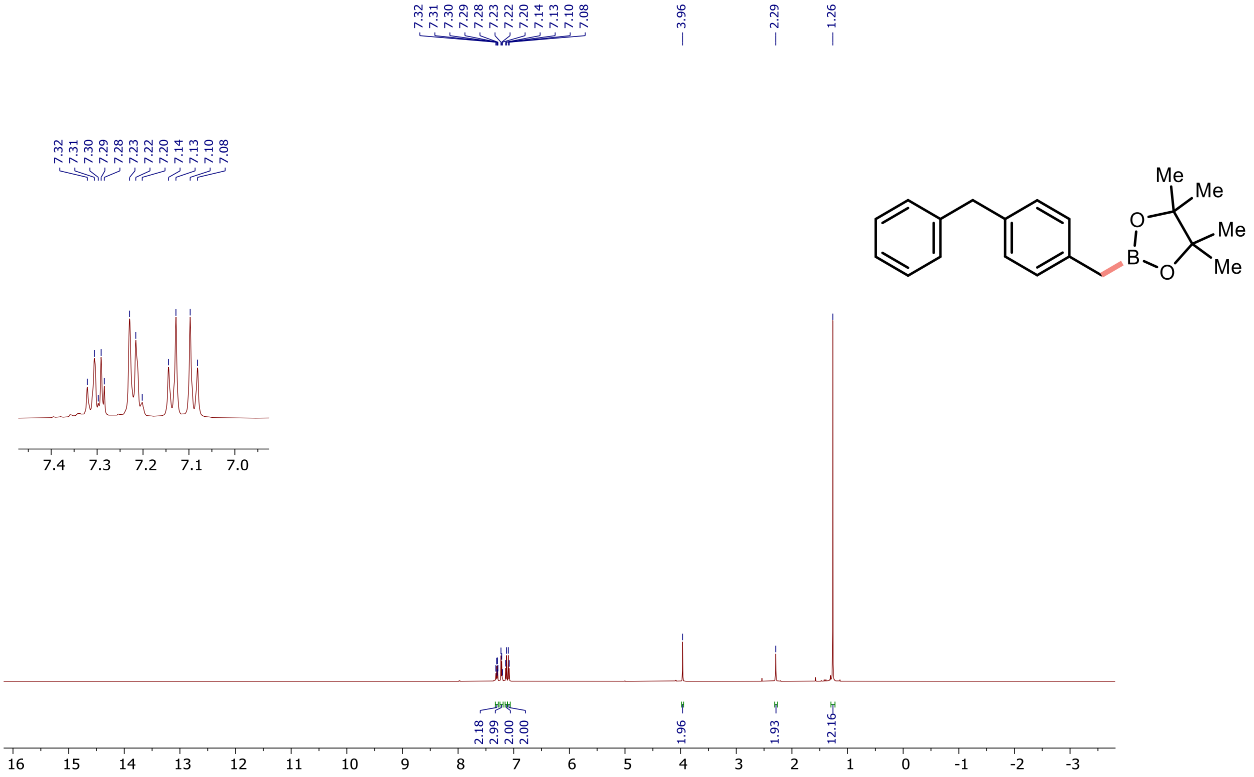

Supplementary Figure 52b-2 ^{13}C NMR (125 MHz, $CDCl_3$) 2-(4-Benzylbenzyl)-4,4,5,5-tetramethyl-1,3,2-dioxaborolane

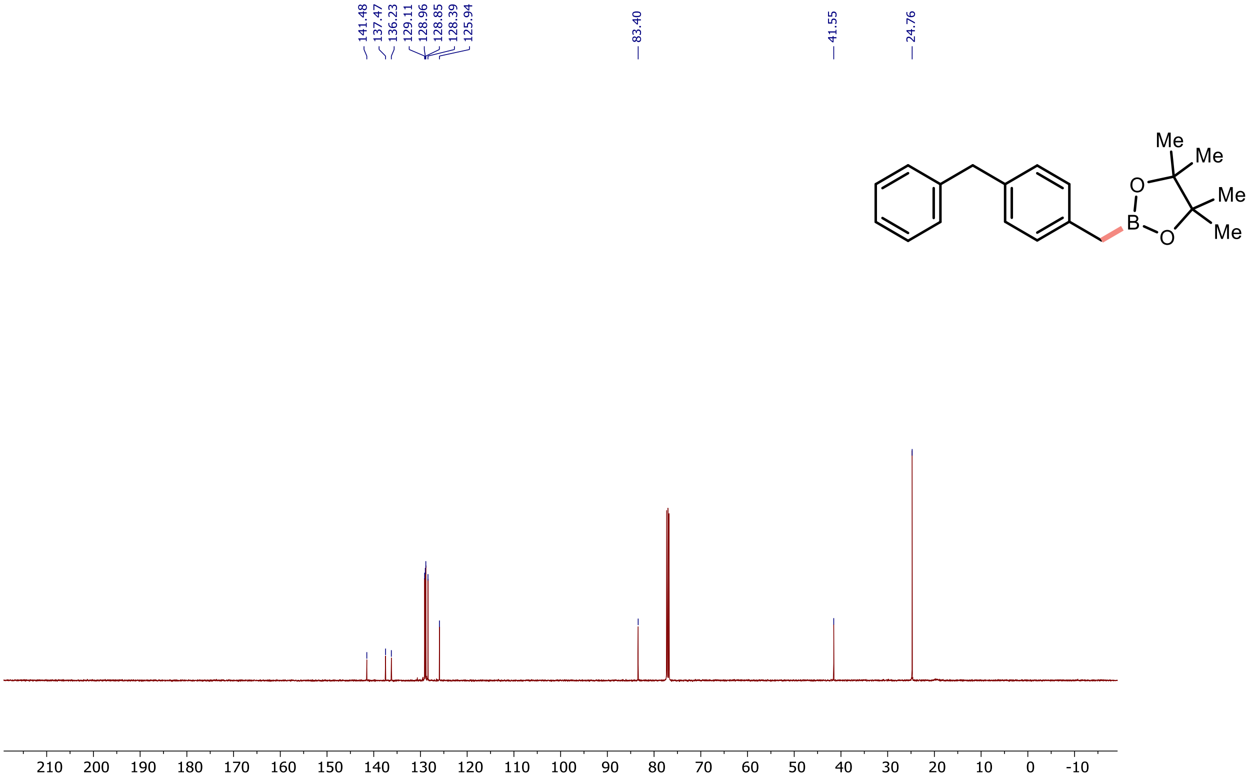

Supplementary Figure 52b-3 ^{11}B NMR (161 MHz, $CDCl_3$) 2-(4-Benzylbenzyl)-4,4,5,5-tetramethyl-1,3,2-dioxaborolane

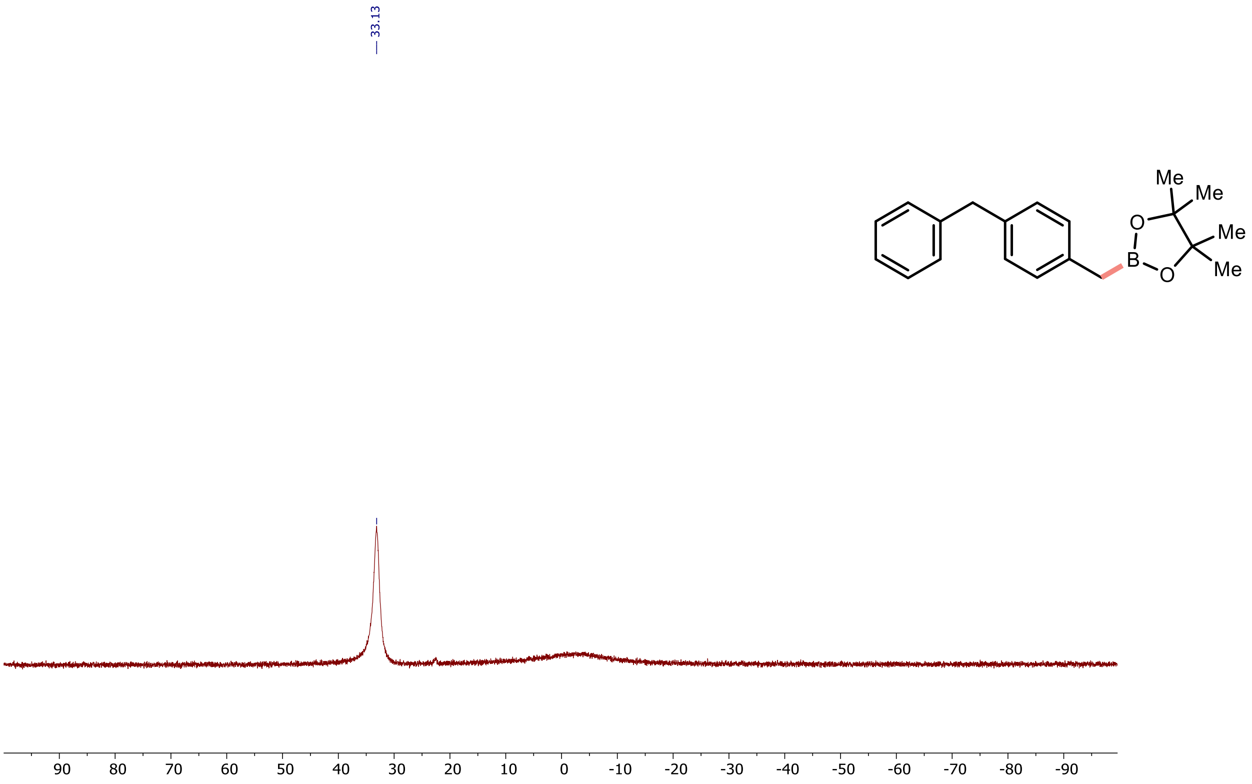

Supplementary Figure 52l-1 ^{1}H NMR (500 MHz, DMSO-d_6) (4-Benzylbenzyl)trifluoro-λ^4-borane, potassium salt

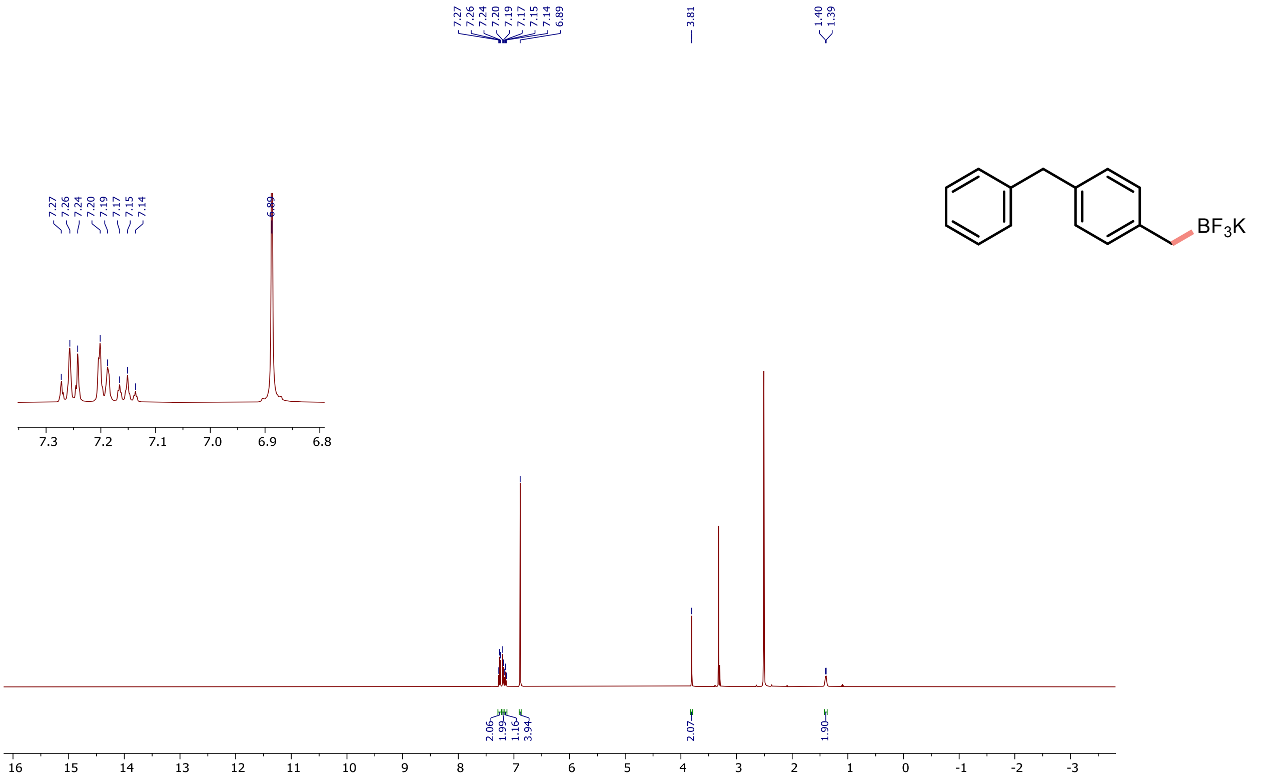

Supplementary Figure 52l-2 ^{13}C NMR (125 MHz, DMSO-d_6) (4-Benzylbenzyl)trifluoro-λ^4-borane, potassium salt

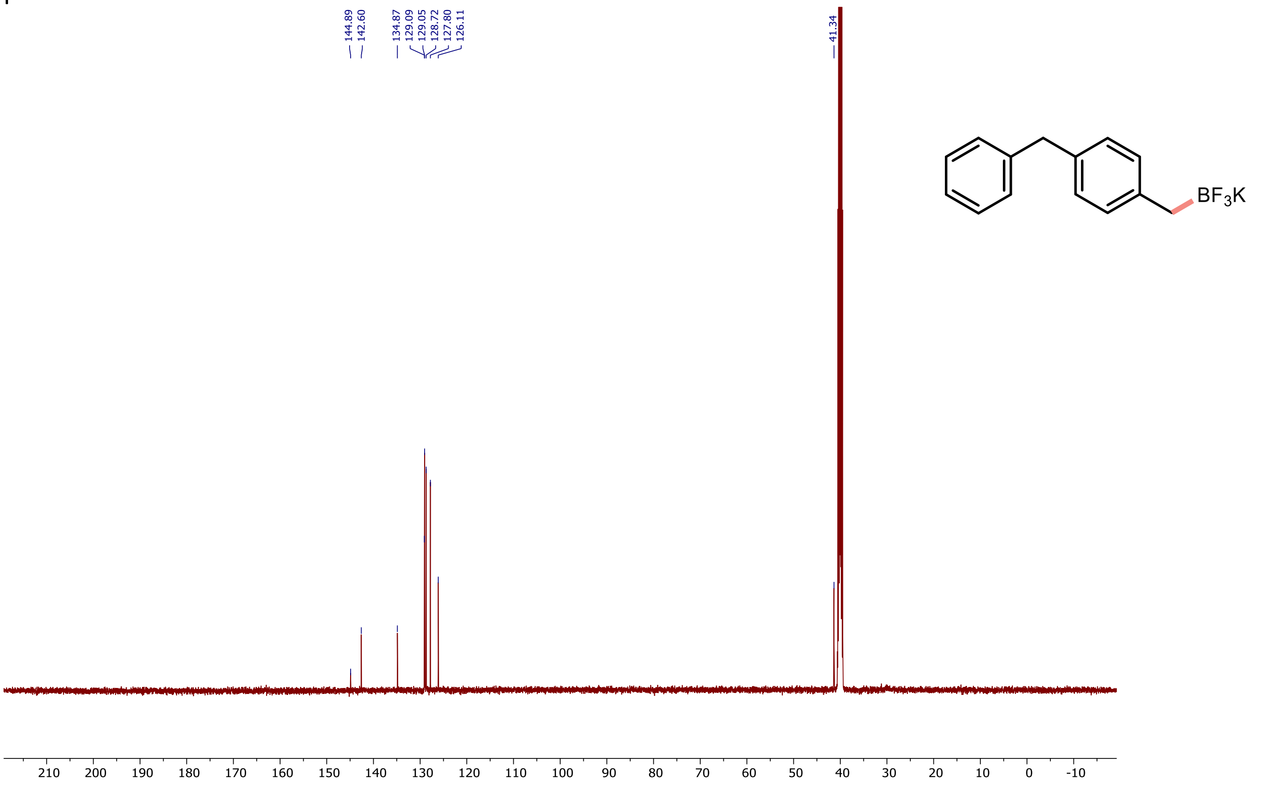

Supplementary Figure 52l-3 ^{19}F NMR (471 MHz, DMSO-d_6) (4-Benzylbenzyl)trifluoro-λ^4-borane, potassium salt

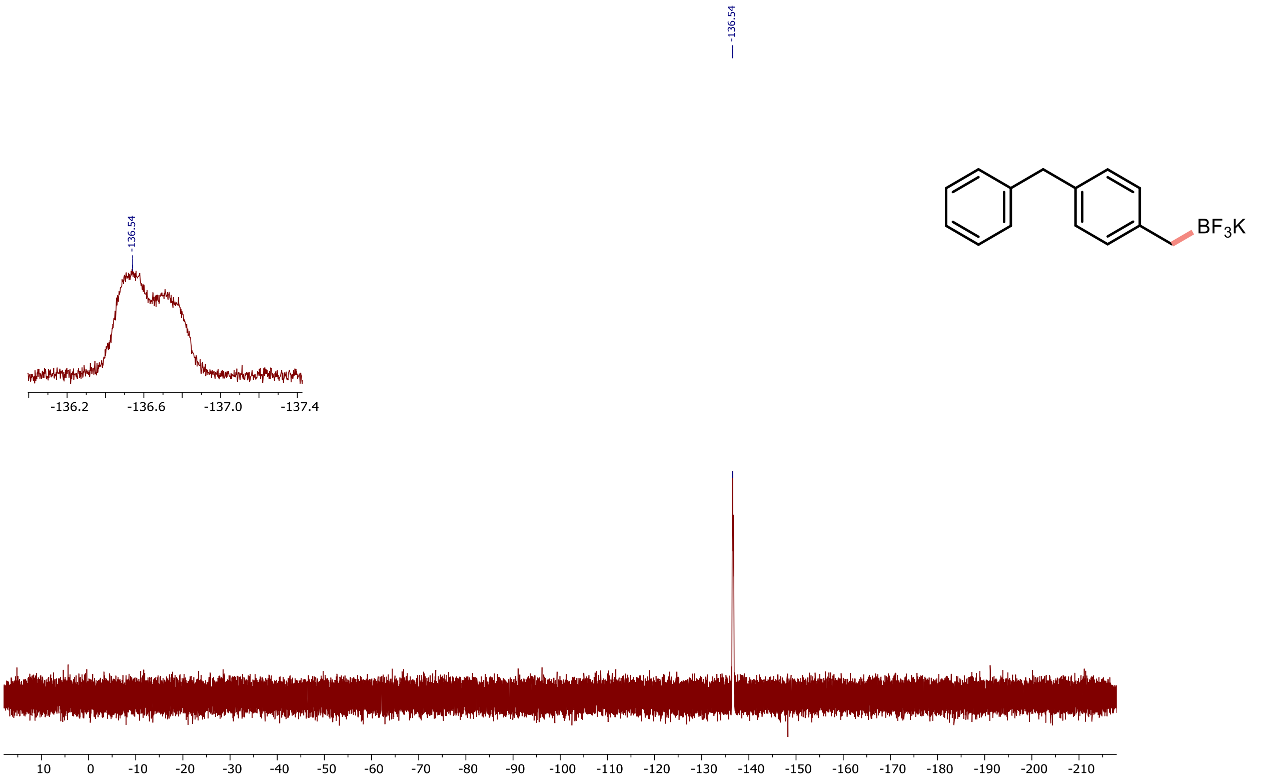

Supplementary Figure 52l-4 ^{11}B NMR (161 MHz, DMSO-d_6) (4-Benzylbenzyl)trifluoro-λ^4-borane, potassium salt

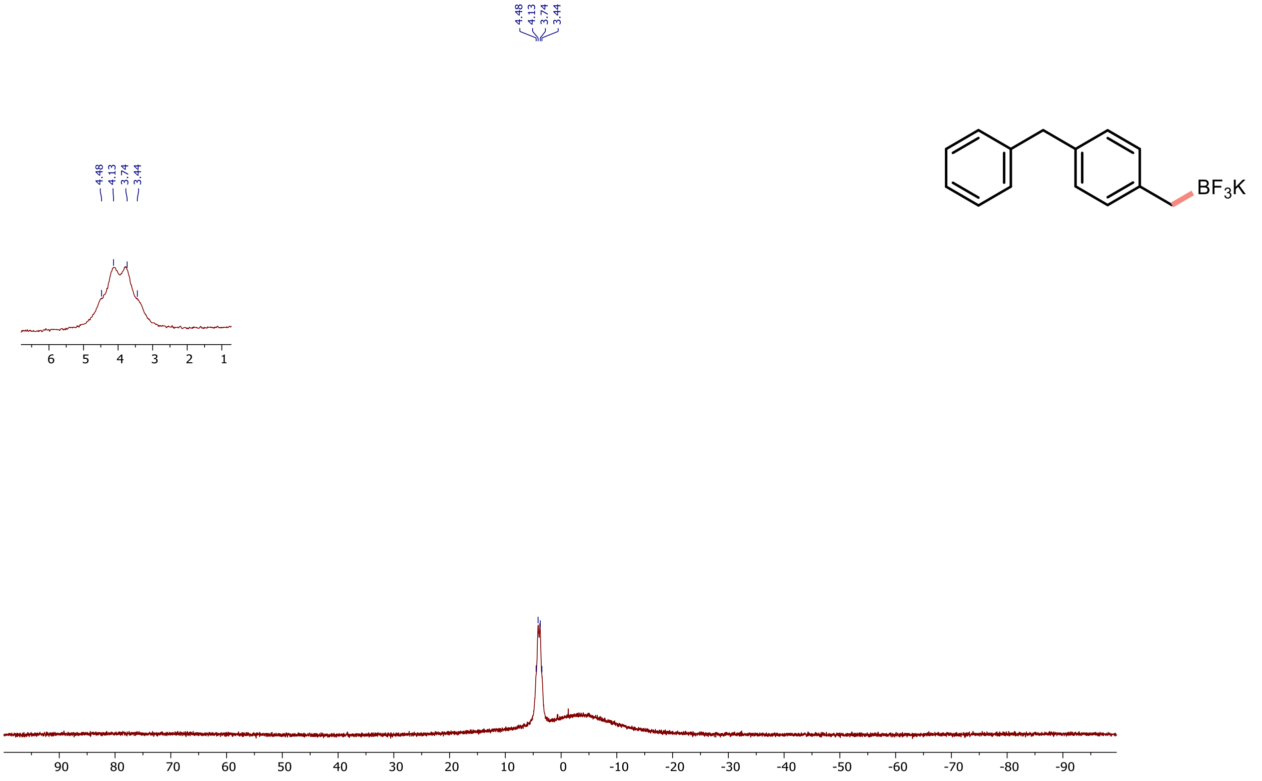

Supplementary Figure 53c-1 ^{1}H NMR (500 MHz, $CDCl_3$) 4-(4-Benzylbenzyl)benzaldehyde

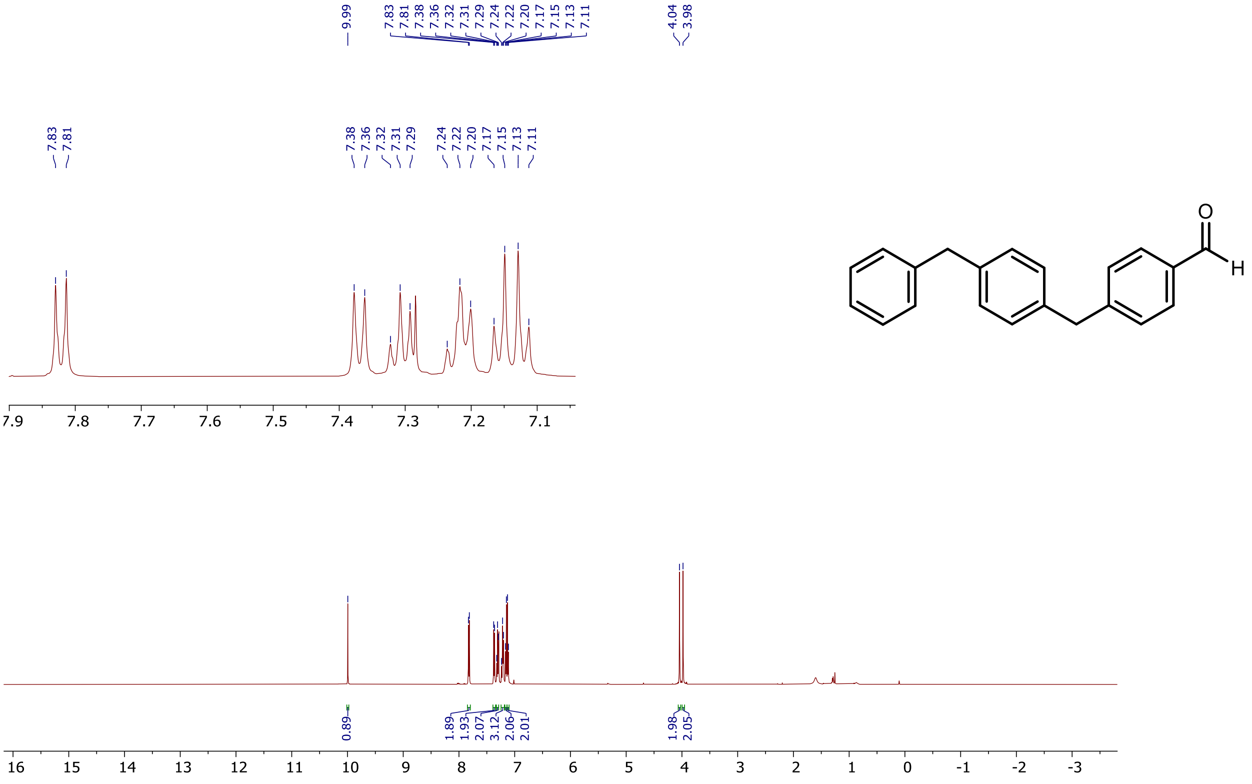

Supplementary Figure 53c-2 ^{13}C NMR (125 MHz, $CDCl_3$) 4-(4-Benzylbenzyl)benzaldehyde

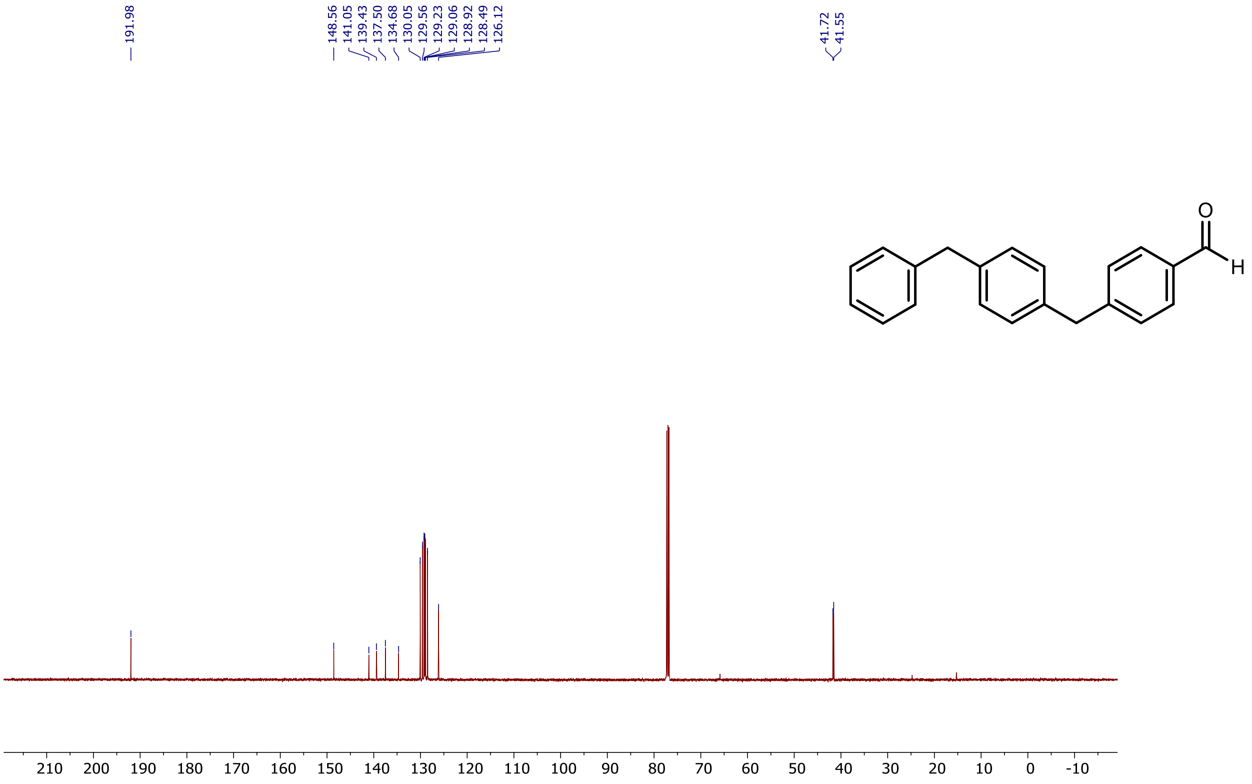

Supplementary Figure 57f-1 ^{1}H NMR (500 MHz, $CDCl_3$) 6-Benzyl-1-(4-methoxyphenyl)-1*H*-benzo[*d*]imidazole

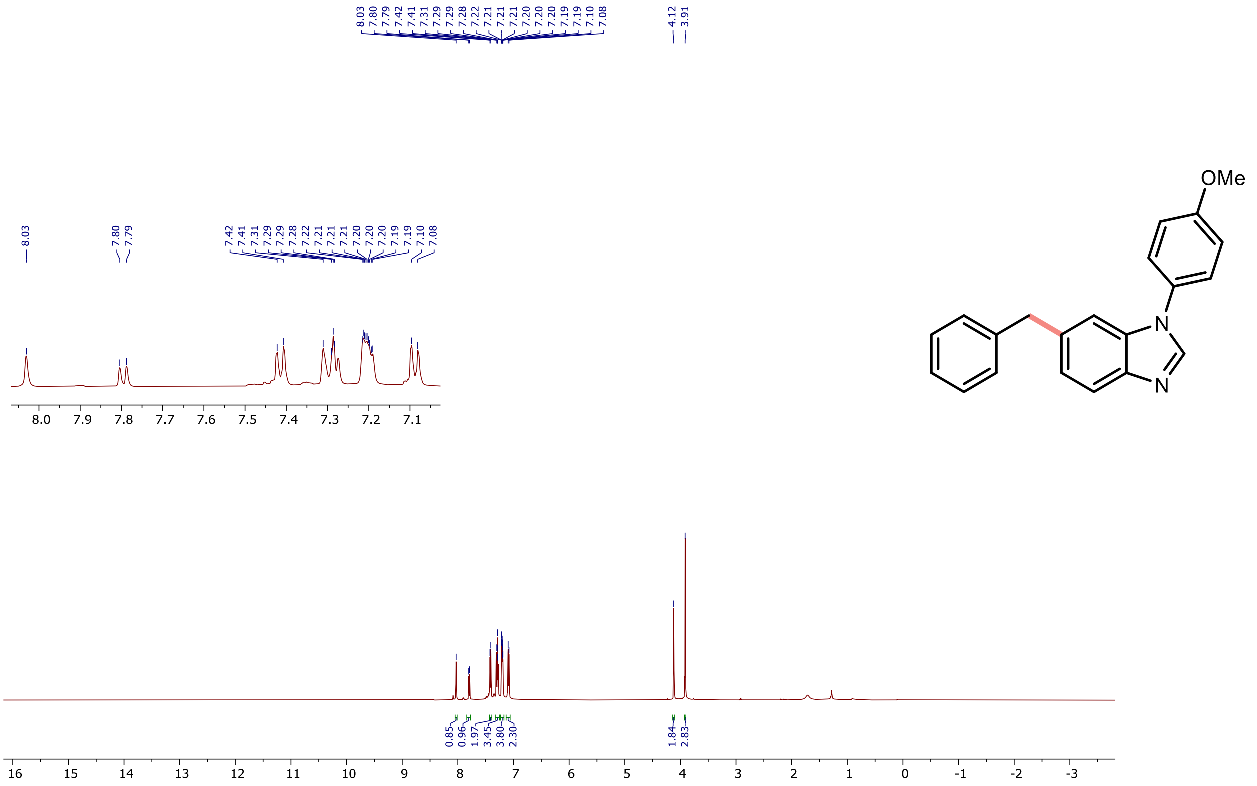

Supplementary Figure 5f-2 ^{13}C NMR (125 MHz, $CDCl_3$) 6-Benzyl-1-(4-methoxyphenyl)-1*H*-benzo[*d*]imidazole

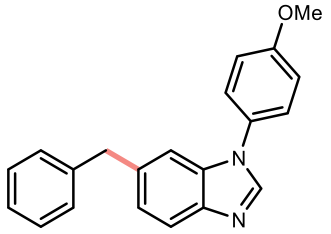

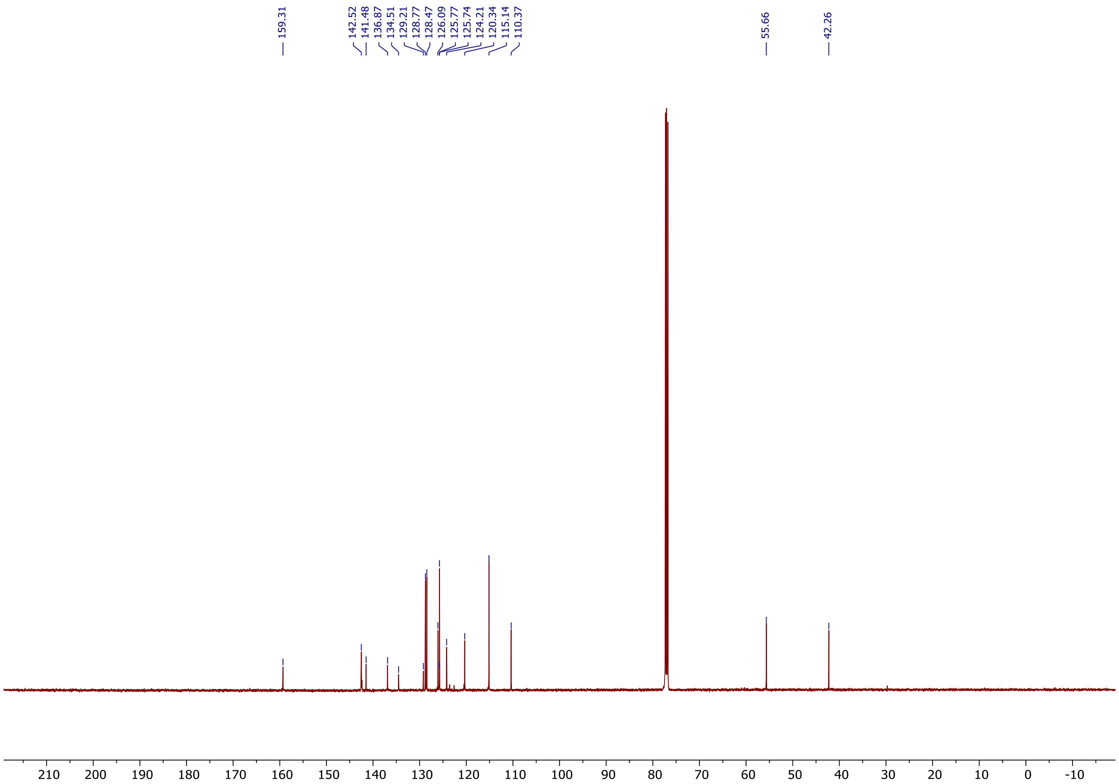
159.31
142.52
141.48
136.87
134.51
129.21
128.77
128.47
126.09
125.77
125.74
124.21
120.34
115.14
110.37
55.66
42.26
210 200 190 180 170 160 150 140 130 120 110 100 90 80 70 60 50 40 30 20 10 0 -10

Supplementary Figure 58f-1 ^{1}H NMR (500 MHz, $CDCl_3$) 1-(4-Methoxyphenyl)-6-(4-methylbenzyl)-1*H*-benzo[*d*]imidazole

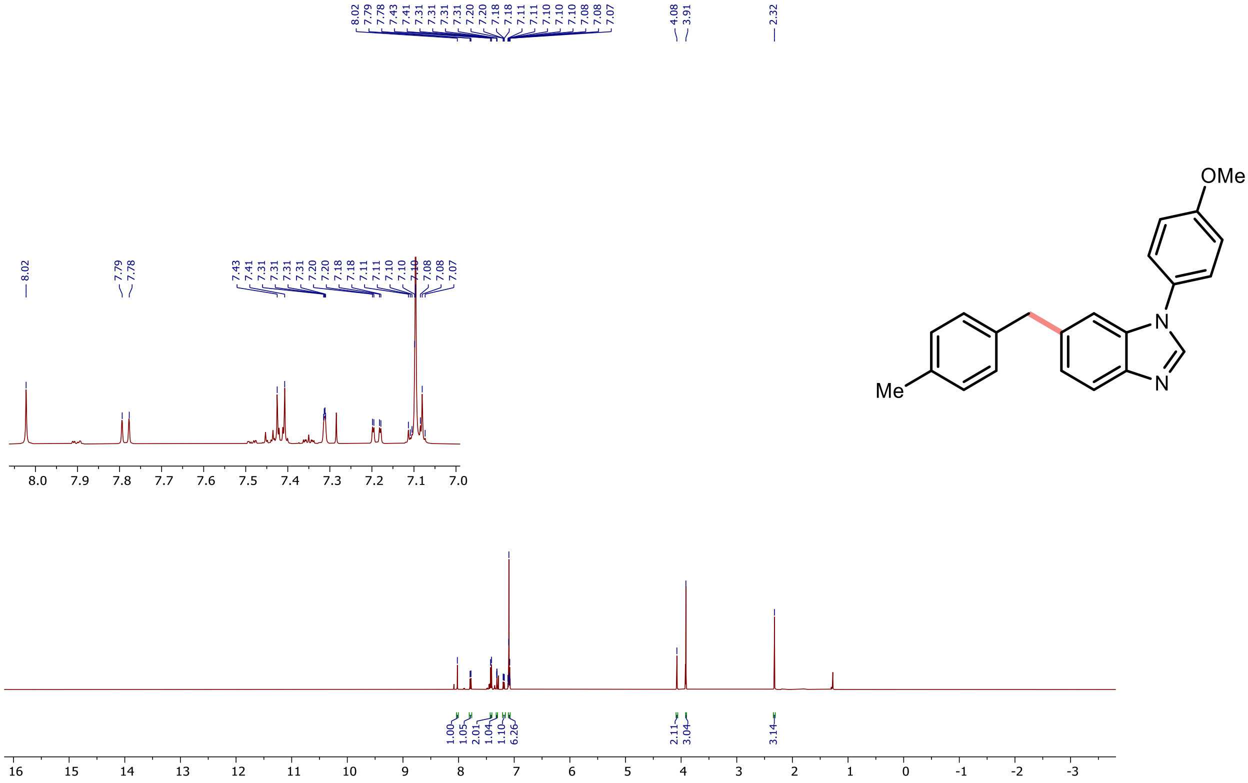

Supplementary Figure 58f-2 ^{13}C NMR (125 MHz, $CDCl_3$) 1-(4-Methoxyphenyl)-6-(4-methylbenzyl)-1*H*-benzo[*d*]imidazole

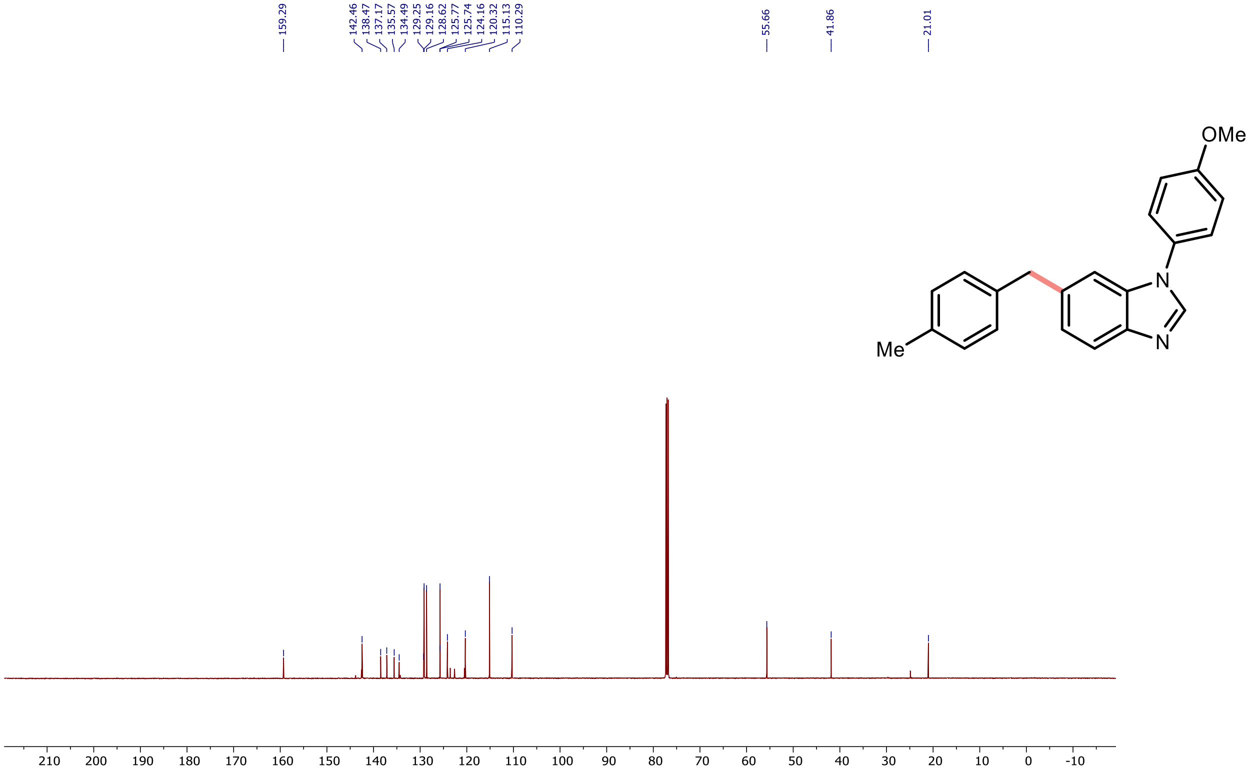

Supplementary Figure 1b-*d*$_{73\%}$-1 ^{1}H NMR (500 MHz, $CDCl_3$) 2-([1,1'-Biphenyl]-4-ylmethyl)-4,4,5,5-tetramethyl-1,3,2-dioxaborolane-*d*$_{73\%}$

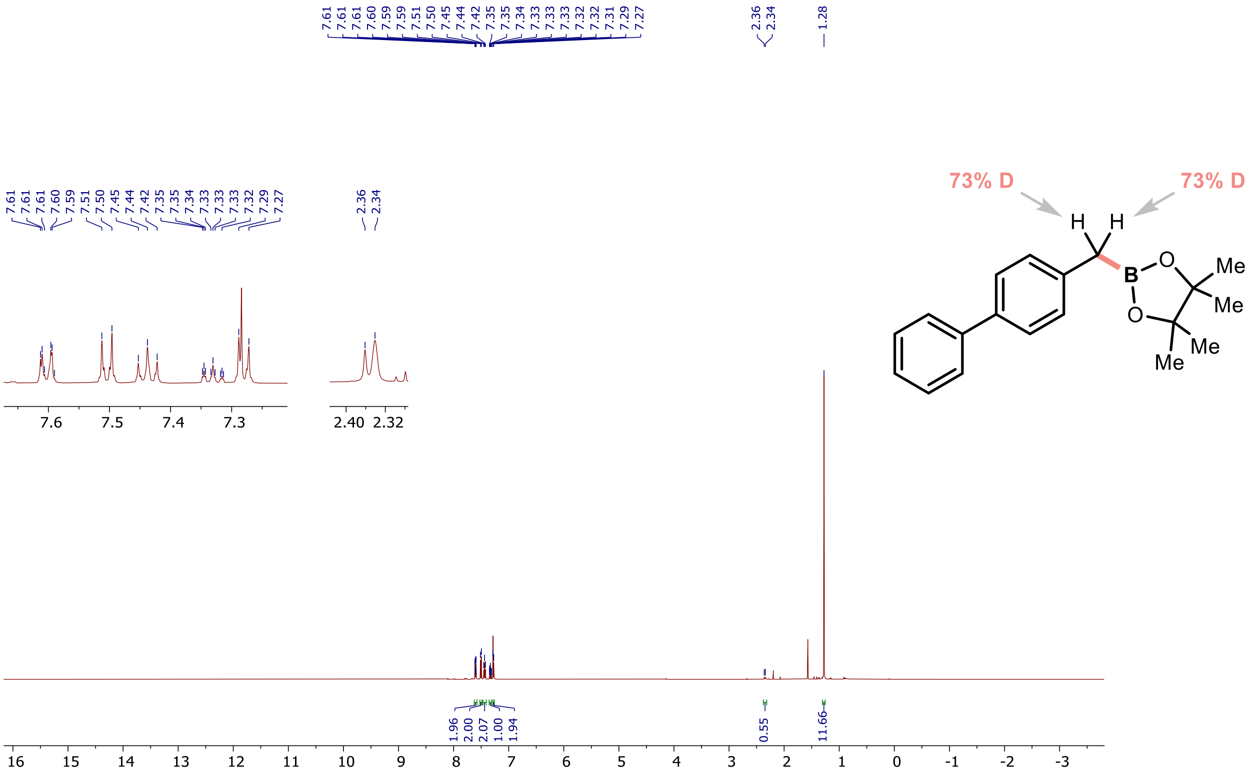

Supplementary Figure 1b-*d*$_{73\%}$-2 ^{13}C NMR (125 MHz, $CDCl_3$) 2-([1,1'-Biphenyl]-4-ylmethyl)-4,4,5,5-tetramethyl-1,3,2-dioxaborolane-*d*$_{73\%}$

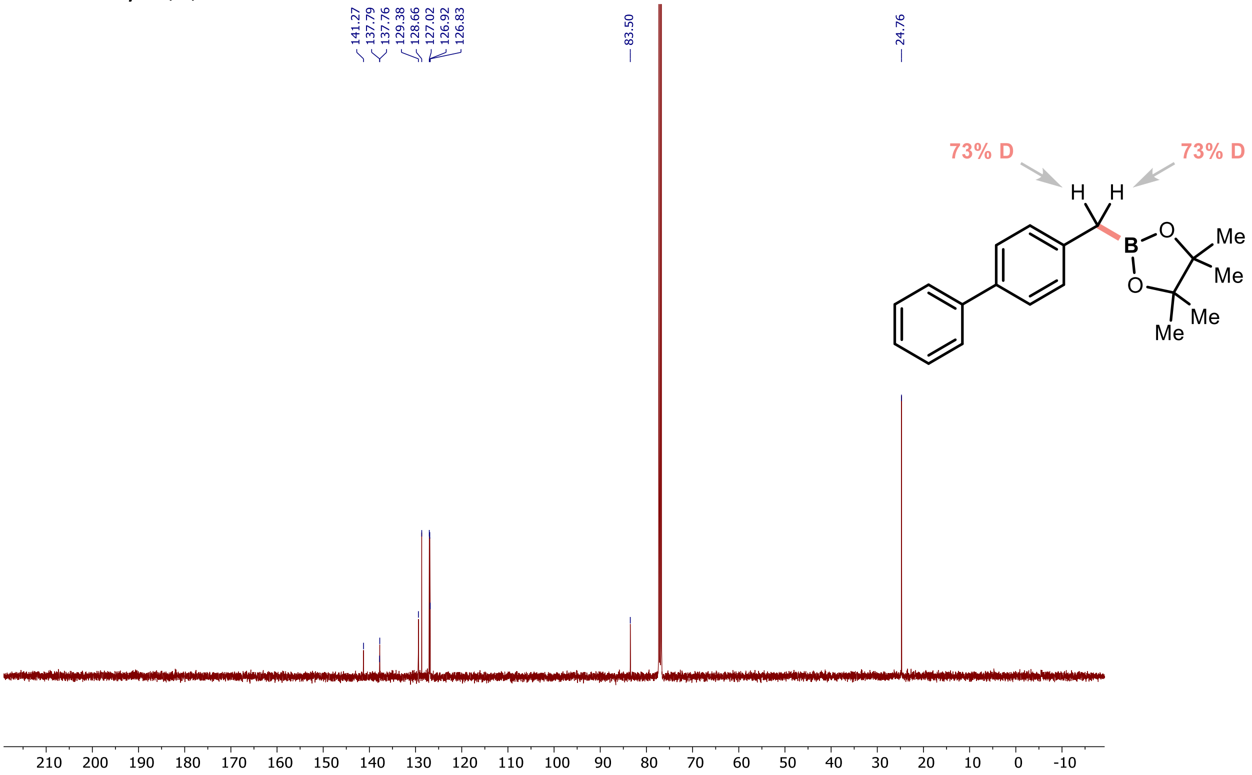

Supplementary Figure 1b-*d*$_{73\%}$-3 ^{2}H NMR (77 MHz, $CDCl_3$) 2-([1,1'-Biphenyl]-4-ylmethyl)-4,4,5,5-tetramethyl-1,3,2-dioxaborolane-*d*$_{73\%}$

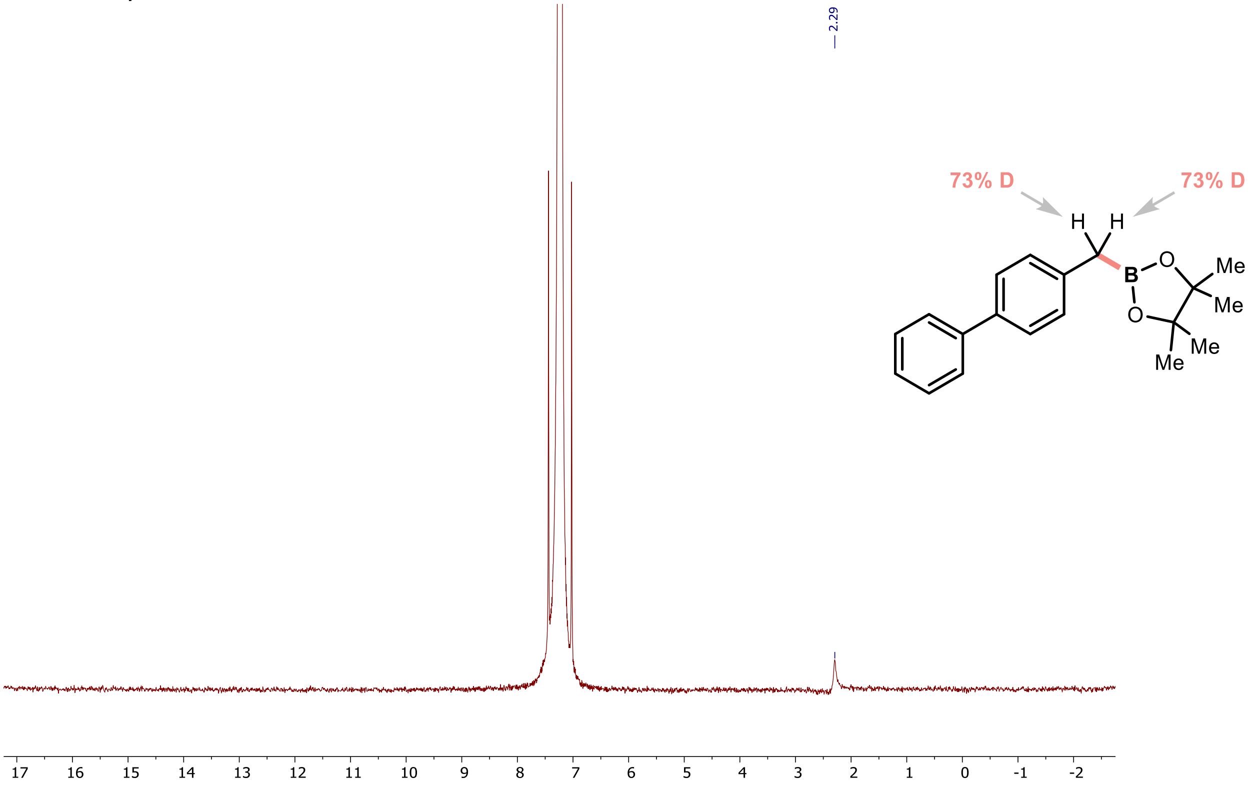

Supplementary Figure 1b-*d*$_{73\%}$-4 ^{11}B NMR (161 MHz, $CDCl_3$) 2-([1,1'-Biphenyl]-4-ylmethyl)-4,4,5,5-tetramethyl-1,3,2-dioxaborolane-*d*$_{73\%}$

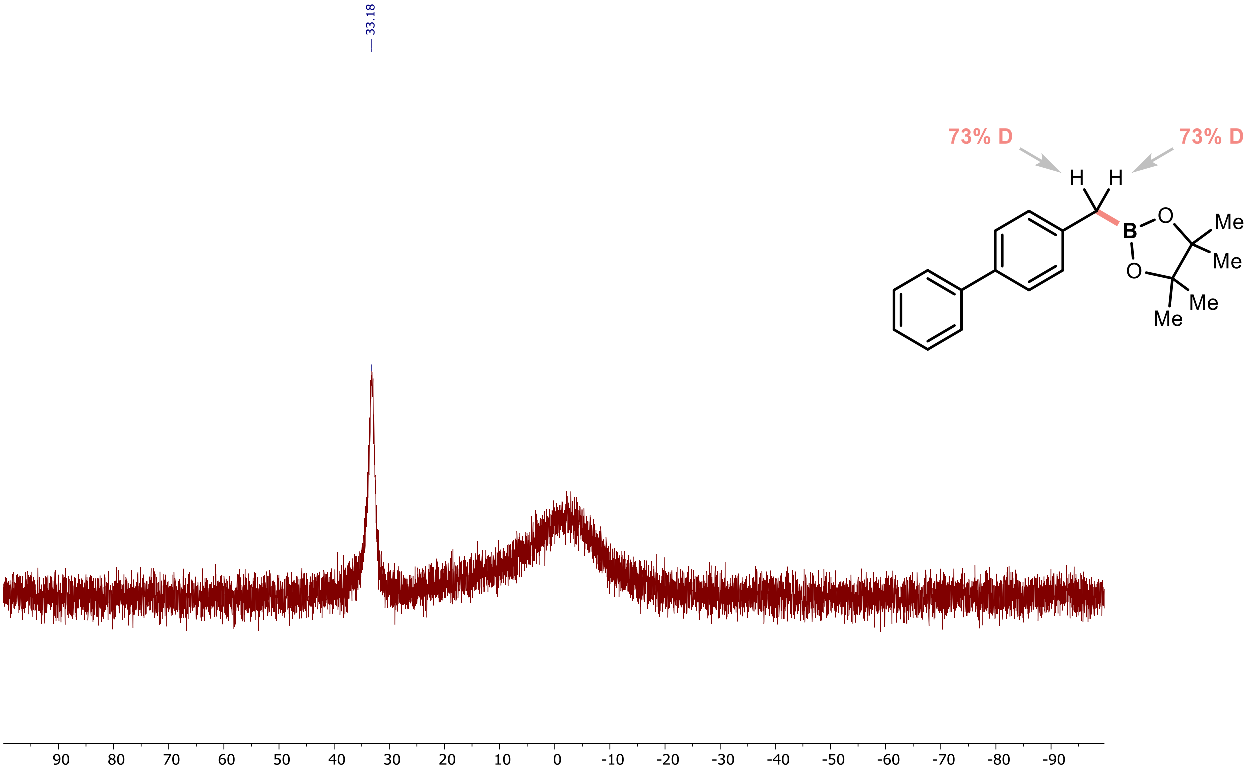

Supplementary Figure 1b-*d*$_{64\%}$-1 ^{1}H NMR (500 MHz, $CDCl_3$) 2-([1,1'-Biphenyl]-4-ylmethyl)-4,4,5,5-tetramethyl-1,3,2-dioxaborolane-*d*$_{64\%}$

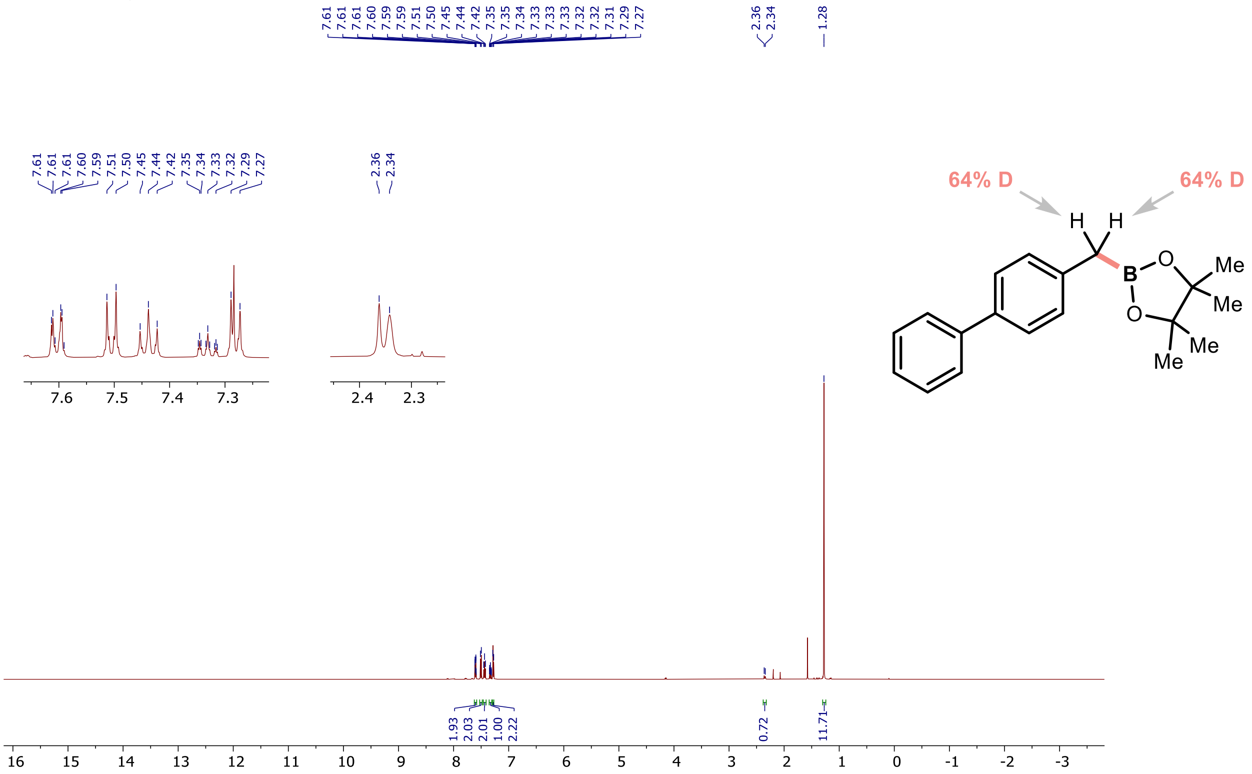

Supplementary Figure 1b-*d*$_{64\%}$-2 ^{13}C NMR (125 MHz, $CDCl_3$) 2-([1,1'-Biphenyl]-4-ylmethyl)-4,4,5,5-tetramethyl-1,3,2-dioxaborolane-*d*$_{64\%}$

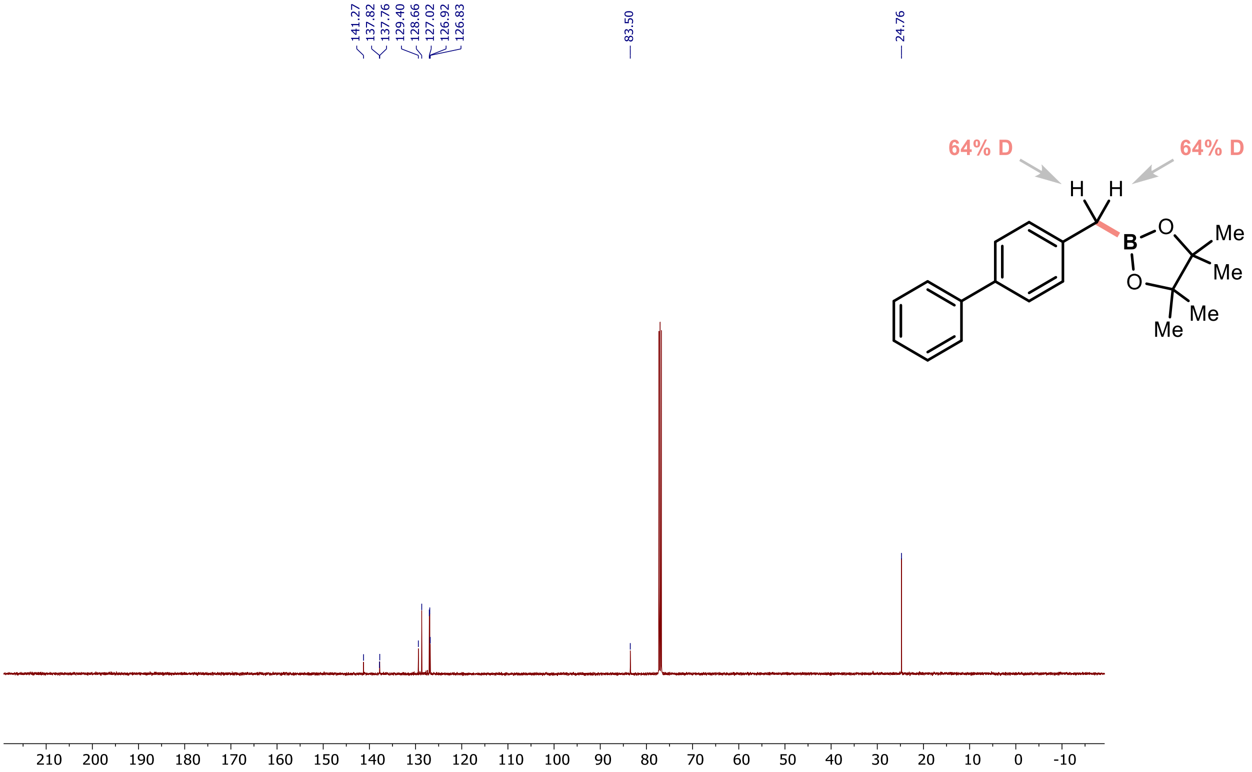

Supplementary Figure 1b-$d_{64\%}$-3 ^{2}H NMR (77 MHz, $CDCl_3$) 2-([1,1'-Biphenyl]-4-ylmethyl)-4,4,5,5-tetramethyl-1,3,2-dioxaborolane-$d_{64\%}$

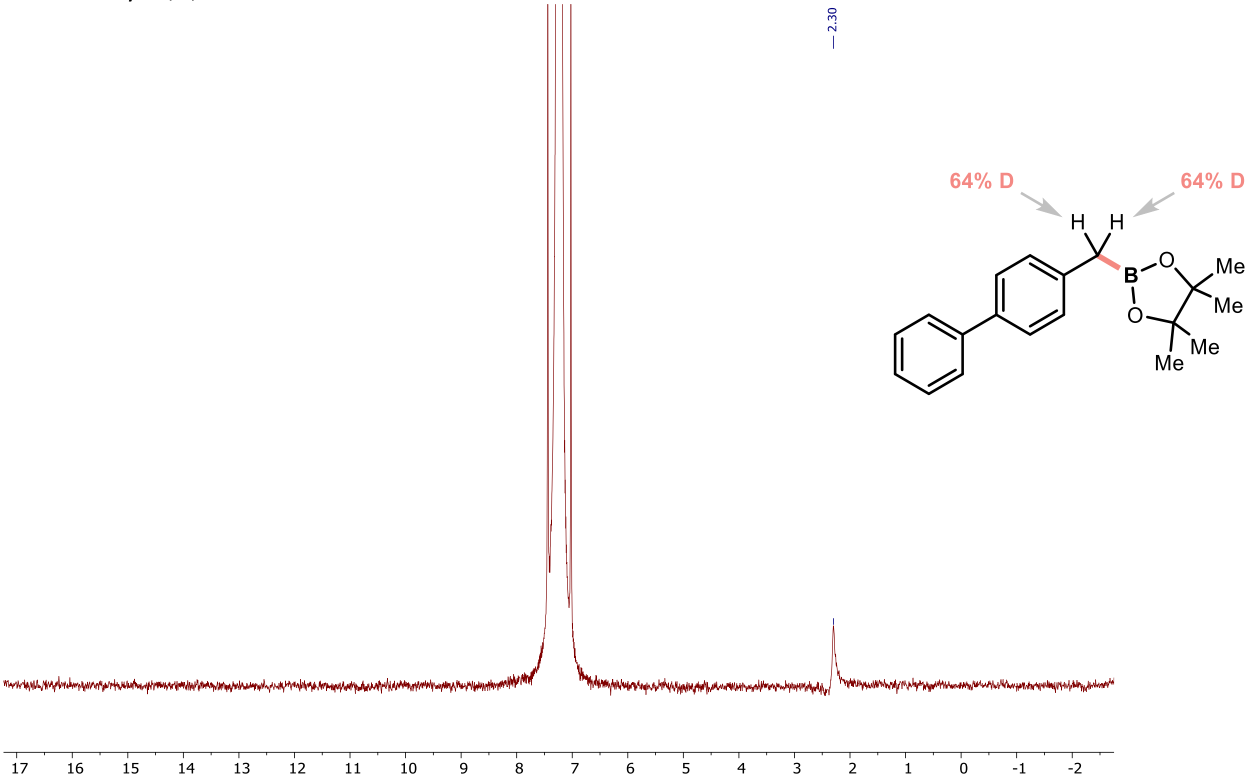

Supplementary Figure 1b-$d_{64\%}$-4 ^{11}B NMR (161 MHz, $CDCl_3$) 2-([1,1'-Biphenyl]-4-ylmethyl)-4,4,5,5-tetramethyl-1,3,2-dioxaborolane-$d_{64\%}$

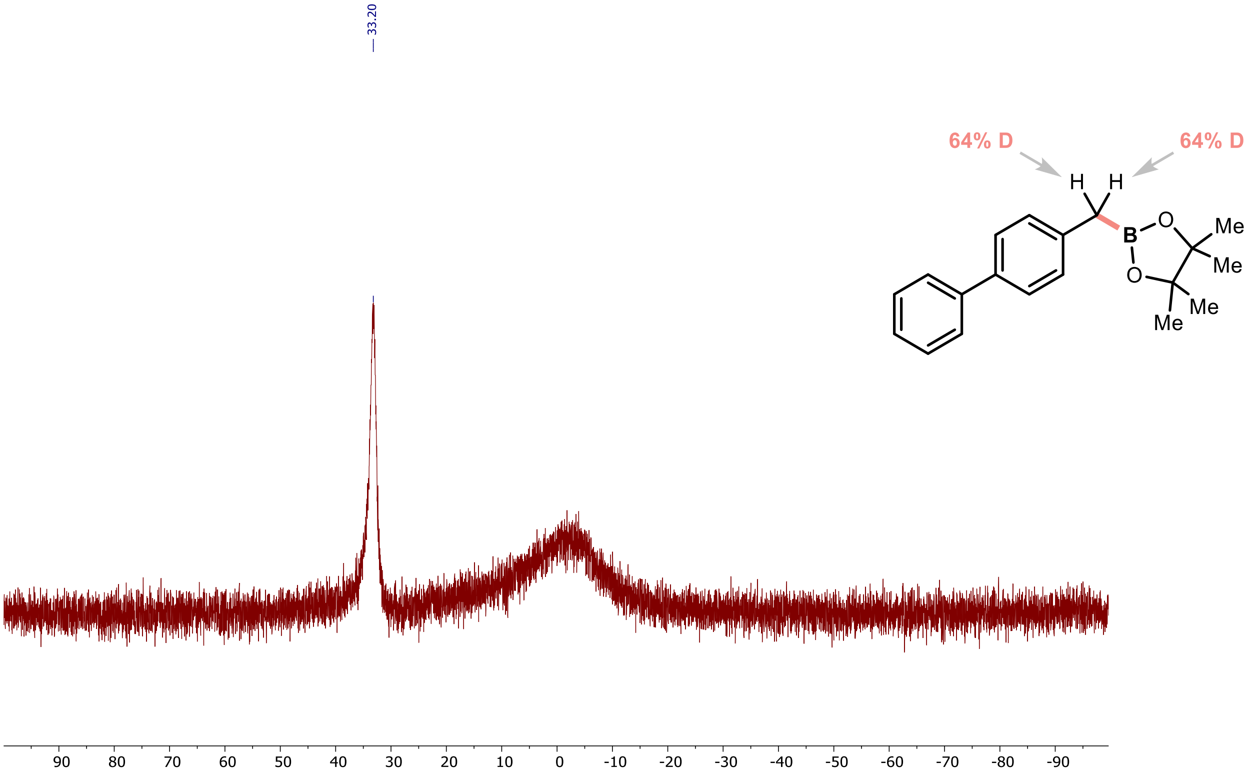

Supplementary Figure 1b-*d*48%-1 ^{1}H NMR (500 MHz, $CDCl_3$) 2-([1,1'-Biphenyl]-4-ylmethyl)-4,4,5,5-tetramethyl-1,3,2-dioxaborolane-*d*48%

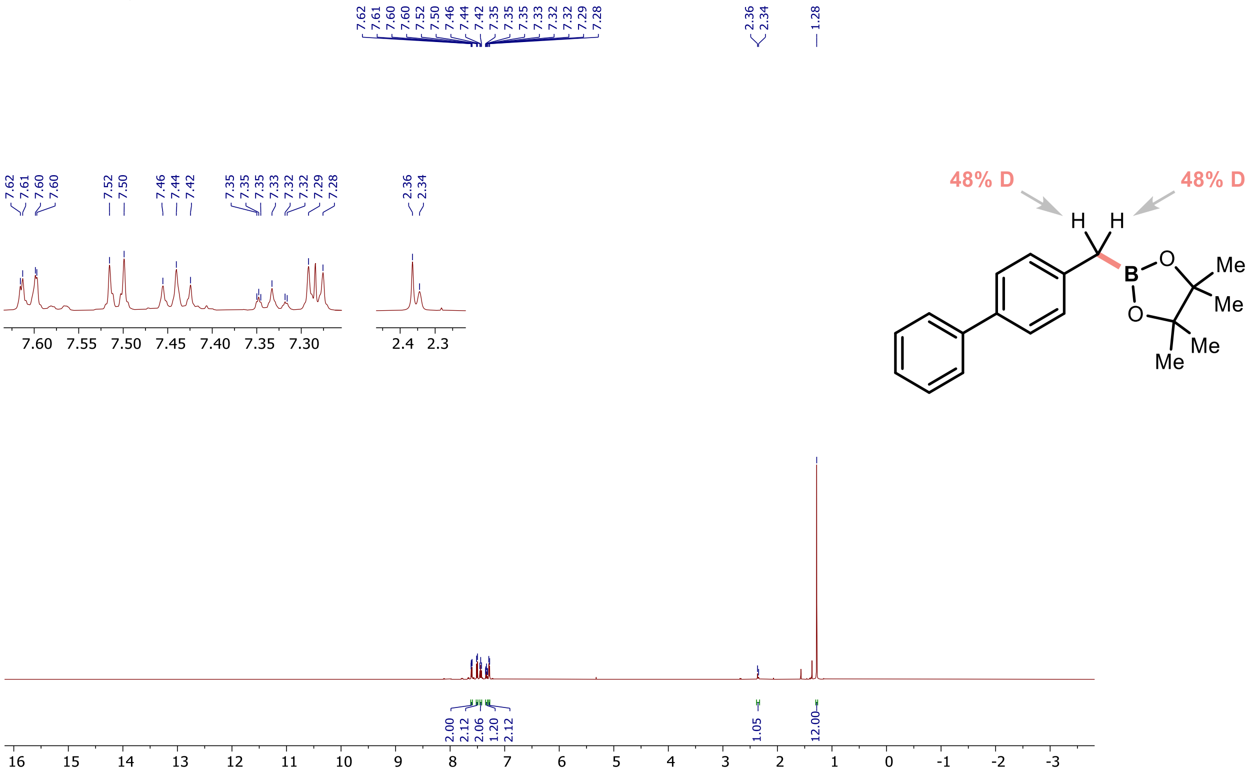

Supplementary Figure 1b-*d*48%-2 ^{13}C NMR (125 MHz, $CDCl_3$) 2-([1,1'-Biphenyl]-4-ylmethyl)-4,4,5,5-tetramethyl-1,3,2-dioxaborolane-*d*48%

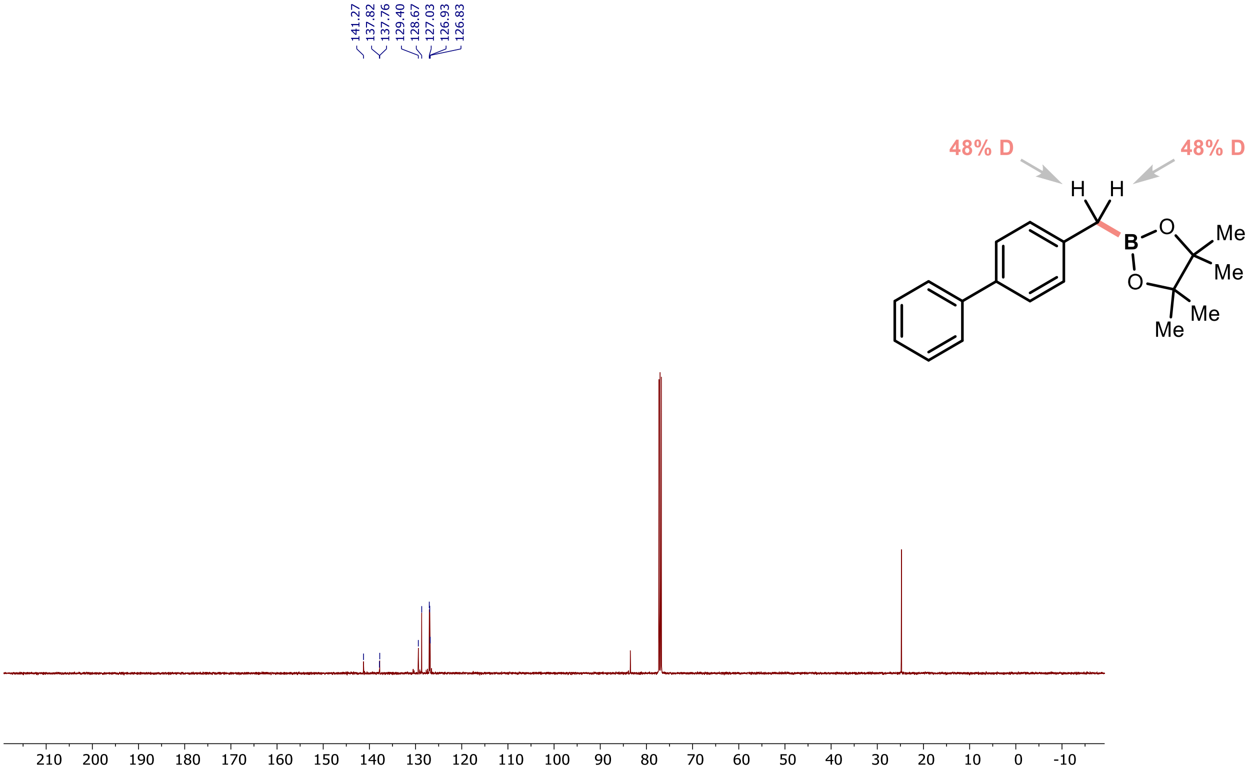

Supplementary Figure 1b-$d_{48\%}$-3 ^{2}H NMR (77 MHz, $CDCl_3$) 2-([1,1'-Biphenyl]-4-ylmethyl)-4,4,5,5-tetramethyl-1,3,2-dioxaborolane-$d_{48\%}$

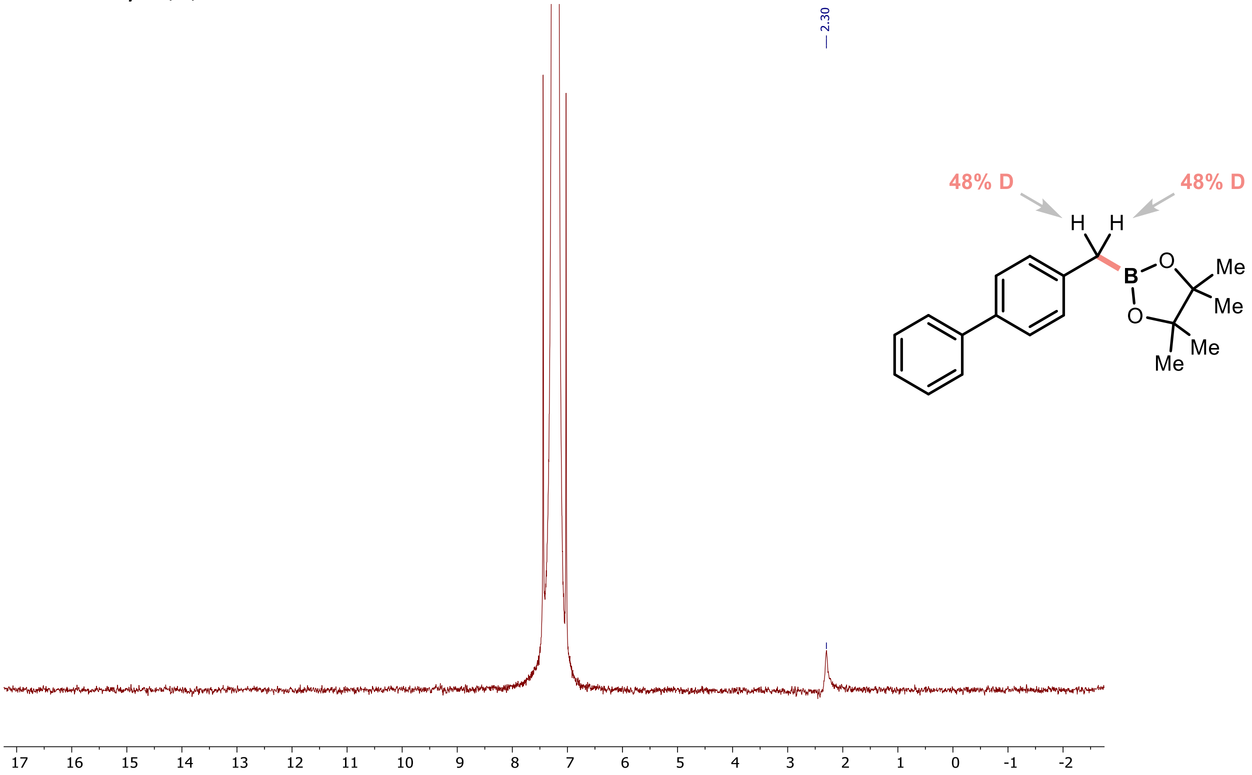

Supplementary Figure 1b-$d_{48\%}$-4 ^{11}B NMR (161 MHz, $CDCl_3$) 2-([1,1'-Biphenyl]-4-ylmethyl)-4,4,5,5-tetramethyl-1,3,2-dioxaborolane-$d_{48\%}$

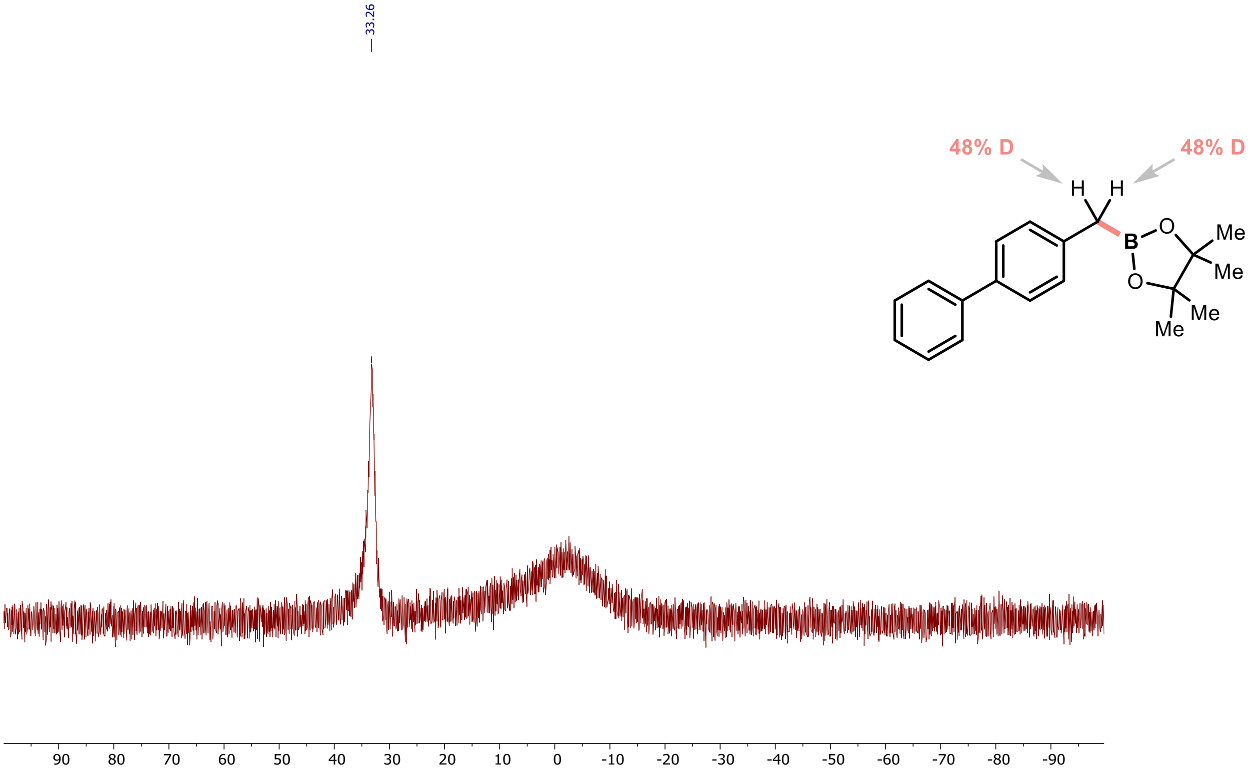

Supplementary Figure 1a-*d*-1 ^{1}H NMR (500 MHz, $CDCl_3$) [1,1'-Biphenyl]-4-carboxylic acid-*d*

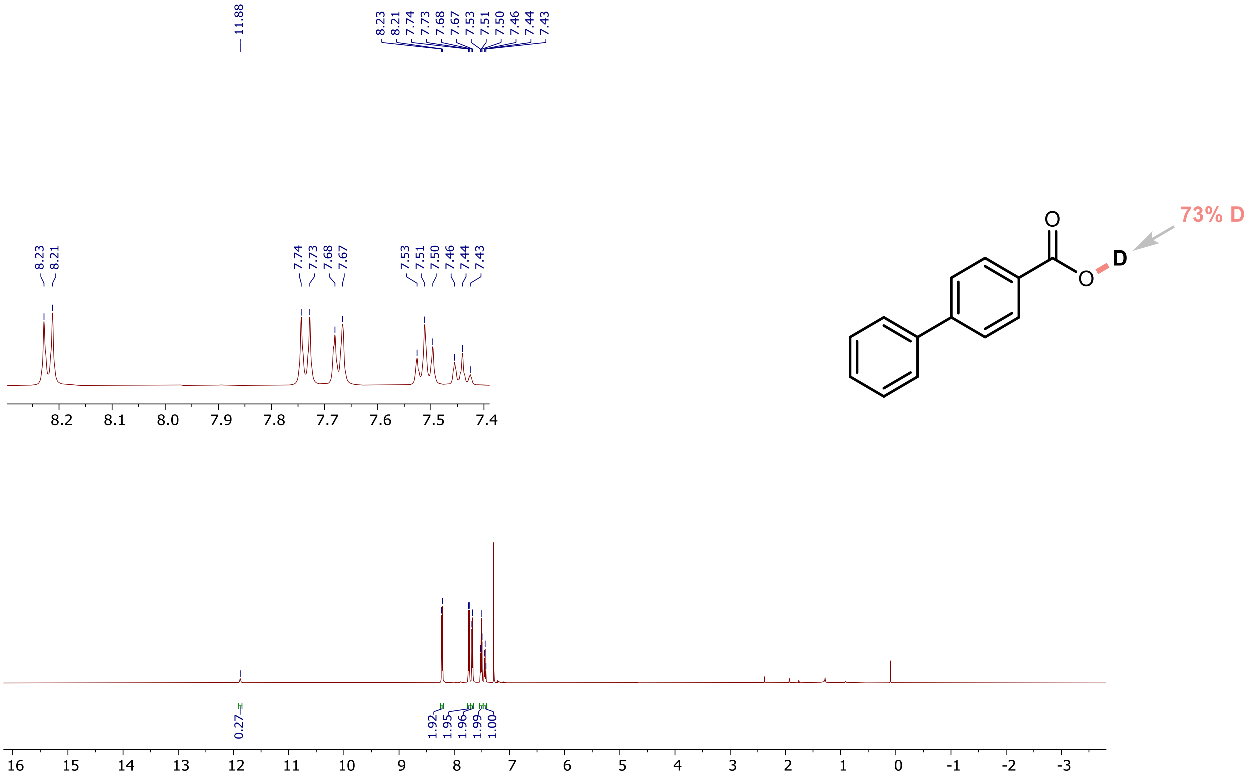

Supplementary Figure 1a-*d*-2 ^{13}C NMR (125 MHz, $CDCl_3$) [1,1'-Biphenyl]-4-carboxylic acid-*d*

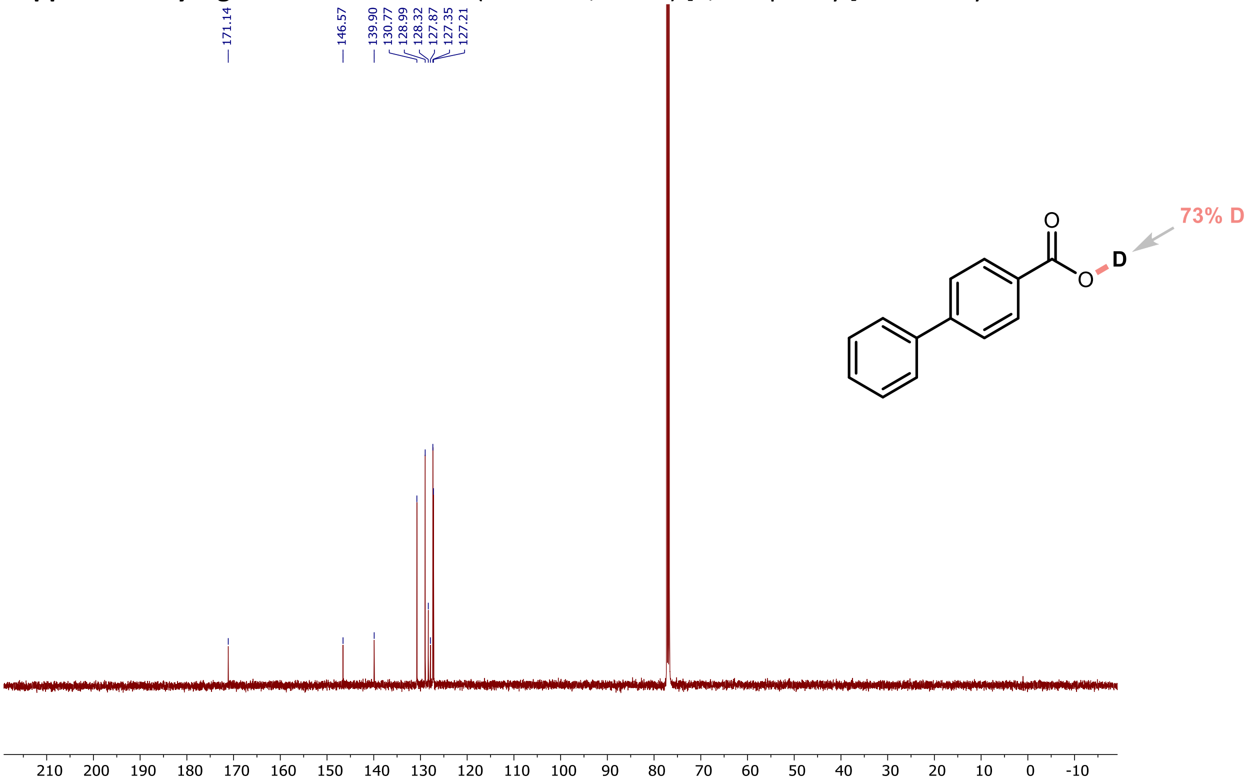

Supplementary Figure 1a-*d*-3 ^{2}H NMR (77 MHz, $CDCl_3$) [1,1'-Biphenyl]-4-carboxylic acid-*d*

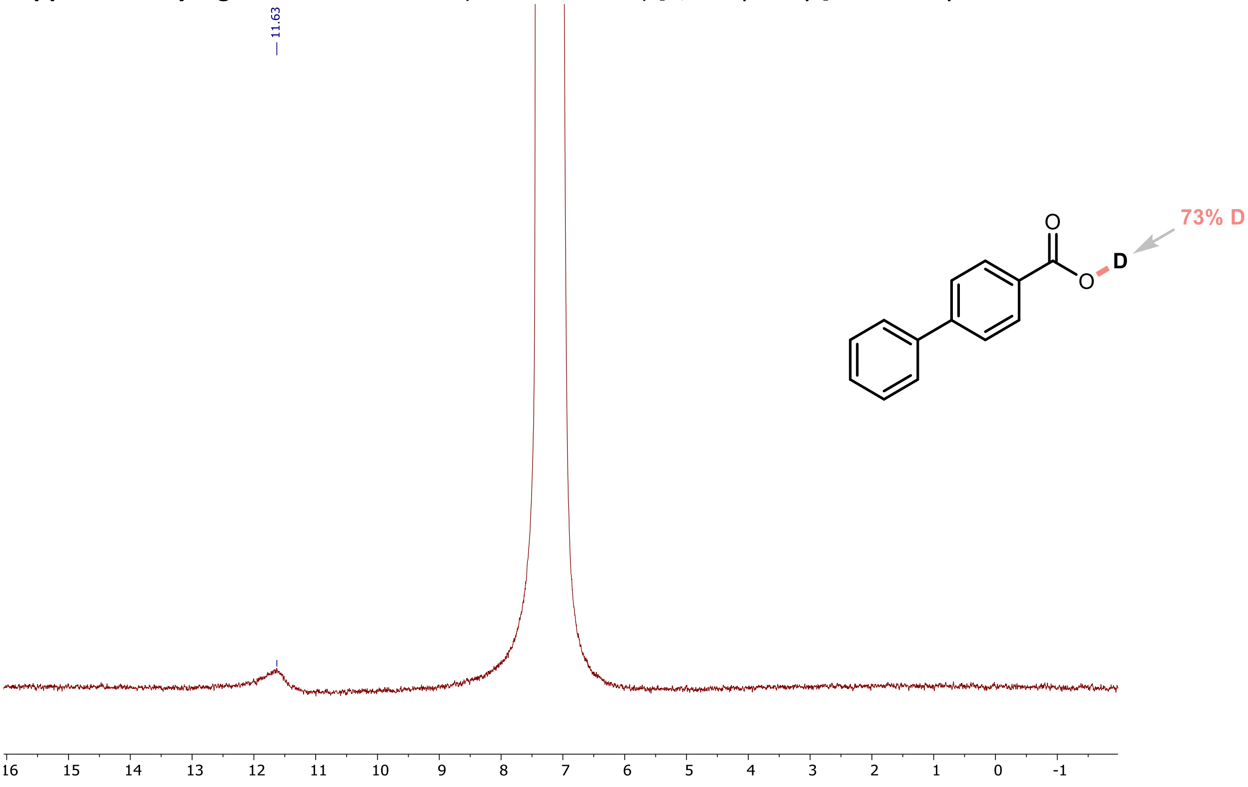

7. Supplementary References

1. Fujinaga, T., Izutsu, K., Sakura, S. Hexamethylphosphoramide: Purification and tests for purity. *Pure Appl. Chem.* **44**, 115-124 (1975).
2. Wang, L., *et al.* C-O functionalization of α-oxyboronates: A deoxygenative *gem*-diborylation and *gem*-silylborylation of aldehydes and ketones. *J. Am. Chem. Soc.* **139**, 5257-5264 (2017).
3. He, Z., *et al.* Cooperation between an alcoholic proton and boryl species in the catalytic gem-hydrodiborylation of carboxylic esters to access 1,1-diborylalkanes. *Org. Chem. Front.* **6**, 900-907 (2019).
4. Su, W., Deng, X., Guo, J., Zhang, X., Wang, X. Activation of aryl carboxylic acids by diboron reagents towards nickel-catalyzed direct decarbonylative borylation. *Angew. Chem., Int. Ed.*, http://doi.org/10.1002/anie.202106356 (2021).
5. Baran, P., Blackmond, D., Chen, L., Barton, L. M. Electrochemical borylation of carboxylic acids. *ChemRxiv*, http://doi.org/10.26434/chemrxiv.14210963.v14210961 (2021).
6. Yang, K., Song, Q. L. Transition-metal-free regioselective synthesis of alkylboronates from arylacetylenes and vinyl arenes. *Green Chem.* **18**, 932-936 (2016).
7. Jiang, X., Zhang, J., Ma, S. Iron catalysis for room-temperature aerobic oxidation of alcohols to carboxylic acids. *J. Am. Chem. Soc.* **138**, 8344-8347 (2016).
8. Zhu, X. J., Liu, Y., Liu, C., Yang, H. J., Fu, H. Light and oxygen-enabled sodium trifluoromethanesulfinate-mediated selective oxidation of C-H bonds. *Green Chem.* **22**, 4357-4363 (2020).
9. Hu, J., Wang, G., Li, S., Shi, Z. Selective C-N borylation of alkyl amines promoted by lewis base. *Angew. Chem., Int. Ed.* **57**, 15227-15231 (2018).
10. Churches, Q. I., Hooper, J. F., Hutton, C. A. A general method for interconversion of boronic acid protecting groups: trifluoroborates as common intermediates. *J. Org. Chem.* **80**, 5428-5435 (2015).
11. Fawcett, A., *et al.* Photoinduced decarboxylative borylation of carboxylic acids. *Science* **357**, 283-286 (2017).
12. Zhang, J. J., Duan, X. H., Wu, Y., Yang, J. C., Guo, L. N. Transition-metal free C-C bond cleavage/borylation of cycloketone oxime esters. *Chem. Sci.* **10**, 161-166 (2019).
13. Wei, D., Liu, T. M., Zhou, B., Han, B. Decarboxylative borylation of *m*CPBA-activated aliphatic acids. *Org. Lett.* **22**, 234-238 (2020).
14. Sun, W., Wang, L., Xia, C., Liu, C. Dual functionalization of α-monoboryl carbanions through deoxygenative enolization with carboxylic acids. *Angew. Chem., Int. Ed.* **57**, 5501-5505 (2018).
15. Stachowiak, H., Kazmierczak, J., Kucinski, K., Hreczycho, G. Catalyst-free and solvent-free hydroboration of aldehydes. *Green Chem.* **20**, 1738-1742 (2018).
16. Wang, W., *et al.* Green hydroboration of carboxylic acids and mechanism investigation. *Org. Biomol. Chem.* **17**, 3604-3608 (2019).
17. Lepage, M. L., *et al.* Direct access to MIDA acylboronates through mild oxidation of MIDA vinylboronates. *Angew. Chem., Int. Ed.* **56**, 15257-15261 (2017).
18. Li, H., *et al.* Formal carbon insertion of *N*-tosylhydrazone into B-B and B-Si bonds: *Gem*-diborylation and *gem*-silylborylation of sp^3 carbon. *Org. Lett.* **16**, 448-451 (2014).
19. Gao, G. L., Yan, J. X., Yang, K., Chen, F. E., Song, Q. L. Base-controlled highly selective synthesis of alkyl 1,2-bis(boronates) or 1,1,2-tris(boronates) from terminal alkynes. *Green Chem.* **19**, 3997-4001 (2017).
20. Coombs, J. R., Zhang, L., Morken, J. P. Enantiomerically enriched tris(boronates): Readily accessible conjunctive reagents for asymmetric synthesis. *J. Am. Chem. Soc.* **136**, 16140-16143 (2014).

21. Antony, J., Grimme, S. Density functional theory including dispersion corrections for intermolecular interactions in a large benchmark set of biologically relevant molecules. *Phys. Chem. Chem. Phys.* **8**, 5287-5293 (2006).
22. Gobato, R., Heidari, A. Calculations using quantum chemistry for inorganic molecule simulation $BeLi_2SeSi$. *Am. J. Quantum Chem. Mol. Spectrosc.* **2**, 37-46 (2017).
23. Grimme, S., Antony, J., Ehrlich, S., Krieg, H. A consistent and accurate *ab initio* parametrization of density functional dispersion correction (DFT-D) for the 94 elements H-Pu. *J. Chem. Phys.* **132**, 154104-154101-154104-154119 (2010).
24. Scalmani, G., Frisch, M. J. Continuous surface charge polarizable continuum models of solvation. I. General formalism. *J. Chem. Phys.* **132**, 114110-114111-114110-114114 (2010).
25. Li, J., Wang, H., Qiu, Z., Huang, C. Y., Li, C. J. Metal-free direct deoxygenative borylation of aldehydes and ketones. *J. Am. Chem. Soc.* **142**, 13011-13020 (2020).
26. Molander, G. A., Ito, T. Cross-coupling reactions of potassium alkyltrifluoroborates with aryl and 1-alkenyl trifluoromethanesulfonates. *Org. Lett.* **3**, 393-396 (2001).
27. Stache, E. E., Rovis, T., Doyle, A. G. Dual nickel- and photoredox-catalyzed enantioselective desymmetrization of cyclic *meso*-anhydrides. *Angew. Chem., Int. Ed.* **56**, 3679-3683 (2017).
28. Cui, L. C., Zhang, Z. Q., Lu, X., Xiao, B., Fu, Y. Pd-catalyzed cross-coupling of 1,1-diborylalkanes with aryl triflates. *RSC Adv.* **6**, 51932-51935 (2016).
29. Wang, L., Sun, W., Liu, C. Cu-catalyzed deoxygenative *gem*-hydroborylation of aromatic aldehydes and ketones to access benzylboronic esters. *Chinese J. Catal.* **39**, 1725-1729 (2018).
30. Larsen, M. A., Wilson, C. V., Hartwig, J. F. Iridium-catalyzed borylation of primary benzylic C-H bonds without a directing group: Scope, mechanism, and origins of selectivity. *J. Am. Chem. Soc.* **137**, 8633-8643 (2015).
31. Li, H., Wang, L., Zhang, Y., Wang, J. Transition-metal-free synthesis of pinacol alkylboronates from tosylhydrazones. *Angew. Chem., Int. Ed.* **51**, 2943-2946 (2012).
32. Mao, L., Szabo, K. J., Marder, T. B. Synthesis of benzyl-, allyl-, and allenyl-boronates via copper-catalyzed borylation of alcohols. *Org. Lett.* **19**, 1204-1207 (2017).
33. Liu, Y.*, et al.* Mild palladium-catalysed highly efficient hydrogenation of C≡N, $C–NO_2$, and C=O bonds using H_2 of 1 atm in H_2O. *Green Chem.* **21**, 830-838 (2019).
34. Koren-Selfridge, L.*, et al.* A boron-substituted analogue of the Shvo hydrogenation catalyst: Catalytic hydroboration of aldehydes, imines, and ketones. *Organometallics* **28**, 2085-2090 (2009).
35. Nielsen, M. K., Ahneman, D. T., Riera, O., Doyle, A. G. Deoxyfluorination with sulfonyl fluorides: Navigating reaction space with machine learning. *J. Am. Chem. Soc.* **140**, 5004-5008 (2018).
36. Fujii, I., Semba, K., Li, Q.-Z., Sakaki, S., Nakao, Y. Magnesiation of aryl fluorides catalyzed by a rhodium–aluminum complex. *J. Am. Chem. Soc.* **142**, 11647-11652 (2020).
37. Gribble, G. W., Nutaitis, C. F. [1.1.1.1.1]Paracyclophane and [1.1.1.1.1.1]paracyclophane. *Tetrahedron Lett.* **26**, 6023-6026 (1985).
38. Ahlmark, M.*, et al.* Catechol *O*-methyltransferase activity inhibiting compounds (2016).
39. Plewe, M.*, et al.* Heterocyclic compounds for the treatment of arenavirus infection and their preparation Patent WO2018013430A2 (2018).
